# Cyclic Alkenylsulfonyl Fluorides: Palladium‐Catalyzed Synthesis and Functionalization of Compact Multifunctional Reagents

**DOI:** 10.1002/anie.201910871

**Published:** 2019-11-06

**Authors:** Terry Shing‐Bong Lou, Scott W. Bagley, Michael C. Willis

**Affiliations:** ^1^ Department of Chemistry University of Oxford Chemistry Research Laboratory Mansfield Road Oxford OX1 3TA UK; ^2^ Global Medicine Design Pfizer Inc. Eastern Point Road Groton CT 06340 USA

**Keywords:** homogeneous catalysis, fluoride, multifunctional reagents, palladium, sulfur

## Abstract

A series of low‐molecular‐weight, compact, and multifunctional cyclic alkenylsulfonyl fluorides were efficiently prepared from the corresponding alkenyl triflates. Palladium‐catalyzed sulfur dioxide insertion using the surrogate reagent DABSO effects sulfinate formation, before trapping with an F electrophile delivers the sulfonyl fluorides. A broad range of functional groups are tolerated, and a correspondingly large collection of derivatization reactions are possible on the products, including substitution at sulfur, conjugate addition, and N‐functionalization. Together, these attributes suggest that this method could find new applications in chemical biology.

## Graphical abstract

**A family of compact** low‐molecular‐weight cyclic alkenylsulfonyl fluorides can be readily prepared from the corresponding alkenyl triflates using palladium catalysis. These densely functionalized reagents undergo a diverse range of derivatization reactions, including substitution at sulfur, conjugate addition, and N‐functionalization.

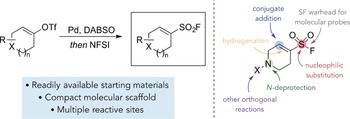

The attractive balance of reactivity and stability that is harnessed in sulfonyl fluorides has propelled these functional groups to the vanguard of new applications in medicinal chemistry and chemical biology.^1^ While they also continue to be of interest as intermediates in synthetic chemistry, it is their tolerance to aqueous media and physiological conditions that is responsible for their popularity in biological contexts.^2^, ^3^, ^4^ Classically, sulfonyl fluorides are prepared from the corresponding sulfonyl chlorides by way of chloride‐fluoride exchange, achieved using potassium bifluoride (KHF_2_)^5^ or the combination of KF with 18‐crown‐6, which is inconvenient to handle due to hygroscopicity.^6^ The required sulfonyl chlorides, typically prepared through the chlorosulfonation of arenes,^7^ are moisture‐sensitive electrophiles and as such are inherently limited by their stability and availability, which is a particular concern in discovery chemistry (Scheme 1 a). The groups of Hyatt,^8^ Sharpless, and others have reported the use of ethenesulfonyl fluoride (ESF) for incorporating sulfonyl fluoride groups through conjugate addition or Pd‐catalyzed Heck‐type coupling reactions using aryl iodides, boronic acids or diazonium salts (Scheme 1 b).^9^ These reactions work well, but are fundamentally limited to the synthesis of sulfonyl fluorides with only ethyl or ethylene linkers. The Willis group has reported a Pd‐catalyzed synthesis of (hetero)arylsulfonyl fluorides from the corresponding aryl bromides, DABSO and NFSI,^10^ and the Ball group has also reported a closely related method (Scheme 1 c).^11^ Unsurprisingly, these latter two reports focus exclusively on planar arene and heteroarene substrates. A recent report has described the electrochemical coupling of thiols and potassium fluoride as a route to sulfonyl fluorides.^12^

**Scheme 1:**
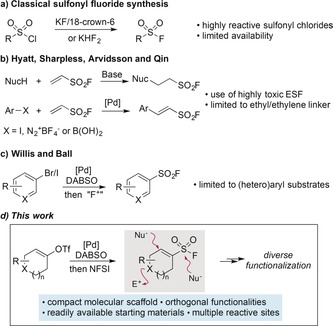
Common strategies for sulfonyl fluoride synthesis and the approach and reagents reported here.

There is an increasing demand for functionalized sp^3^‐rich molecules for use in medical chemistry and chemical biology applications,^13^ and while there are isolated reports of 3D sulfonyl fluorides, their synthesis involves multistep routes and proceeds via unstable sulfonyl chloride intermediates.3e We conceived of a family of small, densely functionalized alkenylsulfonyl fluorides as attractive reagents for possible applications in medical chemistry and chemical biology (Scheme 1 d). Such molecules would contain multiple sp^3^ centers and numerous sites for further functionalization, adding a valuable application to the SuFEx click chemistry toolkit.^14^ Importantly, the excellent functional‐group tolerance achieved in Pd‐catalyzed arylsulfonyl fluoride syntheses suggested that these reactions could be engineered for the preparation of alkenylsulfonyl fluorides from suitable alkenyl (pseudo)halide precursors. Herein, we report that this is the case, and detail the efficient preparation of a broad range of low‐molecular‐weight functionalized alkenylsulfonyl fluorides. We also demonstrate the diverse derivatization reactions that are possible with these new reagents.

Considering substrate availability, we selected alkenyl halides^10^, ^15^ or pseudohalides as the starting materials, in preference to alkenyl boronic acids^16^ or preformed organometallic reagents.^17^ We employed cycloheptenyl iodide (**1**, Table 1) as our test substrate and adopted our previously reported method for the sulfination of aryl iodides,15a followed by electrophilic fluorination using NFSI, as the initial reaction conditions. Using these conditions only a low 17 % yield of the cycloheptenylsulfonyl fluoride **5 a** was obtained (Table 1, entry 1). Reaction monitoring established that alkenyl iodide **1** was fully consumed and alkenylsulfinate **4 a** was formed in the first hour of reaction, with the concentration of **4 a** decreasing over time. Prolonged reaction time for the fluorination step was also found to diminish the yield of alkenylsulfonyl fluoride **5 a**. We speculated that the excess base and alcoholic solvent in use was leading to the decomposition of alkenylsulfinate **4** and alkenylsulfonyl fluoride **5** under these reaction conditions. By limiting the reaction time for both steps to one hour, and performing a solvent switch to CH_3_CN for the fluorination, the yield was improved to 56 % (Table 1, entries 2–3). Substrates with different leaving groups were then examined, with alkenyl bromide **2** and alkenyl triflate **3 a** providing similar yields when PdCl_2_(AmPhos)_2_ was used as catalyst (Table 1, entries 4–6). Alkenyl triflates were chosen for further investigation due to their ease of preparation from the corresponding readily available ketones. Finally, several solvents were evaluated for the fluorination step (Table 1, entries 6–8), with ethyl acetate providing the highest yield (70 %).

**Table 1:** Selected optimization studies.^[a]^ 
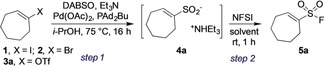

Entry	X	Variation in step 1	Solvent for step 2	Yield of **5 a**
1	I	as above	*i*‐PrOH	17 %
2	I	1 h	*i*‐PrOH	37 %
3	I	1 h	MeCN	56 %
4	Br	PdCl_2_(AmPhos)_2_, 1 h	MeCN	52 %
5	OTf	1 h	MeCN	36 %
6	OTf	PdCl_2_(AmPhos)_2_, 1 h	MeCN	56 %
7	OTf	PdCl_2_(AmPhos)_2_, 1 h	EtOAc	67 % (70 %)^[b]^
8	OTf	PdCl_2_(AmPhos)_2_, 1 h	*i*‐PrOH	54 %
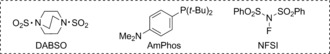				

[a] Reaction conditions: (step 1) Alkenyl (pseudo)halide (0.3 mmol, 1 equiv), DABSO (0.6 equiv), Et_3_N (3 equiv), Pd(OAc)_2_ (5 mol %), PAd_2_(*n*‐Bu) (7.5 mol %), *i*‐PrOH [0.25 m], 75 °C, 16 h; (step 2) NFSI (1.5 equiv), solvent [0.25 m], rt, 1 h. Yields determined by ^19^F‐NMR spectroscopy with internal standard. [b] Yield of isolated product.

With alkenyl triflates selected as the substrates and optimized conditions identified, we next examined the substrate scope for the reaction (Table 2). While the parent cyclohexenylsulfonyl fluoride proved to be too volatile for straightforward isolation, a broad range of cyclohexenyl triflate derivatives were examined in this reaction. A variety of functional groups at the 4‐position were tolerated, including phenyl (**5 b**), *tert*‐butyl (**5 c**), geminal difluoro (**5 d**), ethyl ester (**5 e**), methoxy ether (**5 f**), Boc‐protected amine (**5 g**) and spirodioxolane (**5 h**) groups. Viability for scale‐up was illustrated by the gram‐scale reaction to form sulfonyl fluoride **5 h**; one gram of alkenyl triflate **3 d** (3.5 mmol) delivered 0.57 g (2.57 mmol) of **5 h** in 73 % yield, which is comparable to the 74 % yield obtained for a 0.3 mmol scale reaction. Substrates with substituents at the 3‐position were also converted into alkenylsulfonyl fluorides efficiently, with an electron‐rich heteroaromatic 1‐methylindole (**5 i**) and 2‐methylfuran (**5 k**) tolerated to give alkenylsulfonyl fluorides in 52 % and 68 % yield, respectively. A substrate with a 6‐methyl substituent was slower to react and required heating to 95 °C for the sulfination step, ultimately providing sulfonyl fluoride **5 l** in 43 % yield. Heterocyclic substrates were then explored, with sulfone‐ and dihydropyran‐containing products being efficiently obtained (**5 m**, **n**). Protected amines at both the 4‐ and 5‐positions could also be incorporated (**5 o–s**). Of these, the substrates featuring carbamate (**5 o–q**) and sulfonamide (**5 s**) groups were most efficient, with the basic N‐benzyl derivative requiring the addition of Hünig's base in the fluorination step (**5 r**). The X‐ray structure of sulfonyl fluoride **5 s**, which features a sulfonamide protected amine, is shown, and illustrates the expected half‐chair conformation common to cyclohexenes as well as the relatively small C‐SO_2_‐F bond‐angle of 100.3°.^18^, ^19^ Tetralone‐derived alkenyl triflates were converted into alkenylsulfonyl fluorides (**5 u–x**) in moderate to good yields. Interestingly, whilst substrate **3 x** bears both an alkenyl and an aryl triflate, only the alkenyl triflate was converted with the aryl triflate being preserved. It followed that aryl triflates are inert to the present reaction conditions, with 96 % recovery of the biphenyl triflate and no formation of sulfonyl fluoride **5 y** occurring. Five‐membered‐ring alkenyl triflates (**3 j**,**t**) were poor substrates, as was an acyclic example (**3 z**), with the latter undergoing decomposition under the reactions conditions.

**Table 2:** Scope with respect to the cyclic alkenyl sulfonyl fluorides.^[a]^ 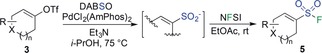

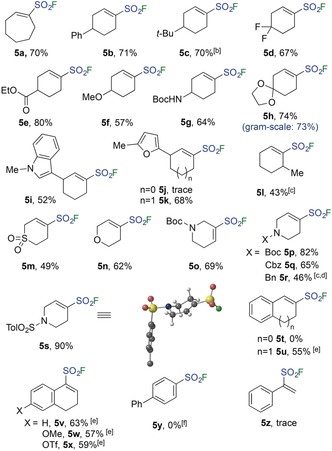

[a] Reaction conditions: Alkenyl triflate **3** (0.3 mmol, 1 equiv), DABSO (0.6 equiv), Et_3_N (3 equiv), PdCl_2_(AmPhos)_2_ (5 mol %), *i*‐PrOH [0.25 m], 75 °C, 0.5–10 h; then NFSI (1.5 equiv), EtOAc [0.25 m], rt, 1 h. [b] Step 1: 80 °C. [c] Step 1: μW, 95 °C. [d] Step 2: DIPEA (3 equiv) was added after solvent switch and prior to the addition of NFSI. [e] Step 1: PdCl_2_(AmPhos)_2_ (5 mol %), DABSO (1 equiv), Et_3_N (3 equiv), *i*‐PrOH/1,4‐dioxane (2:1) [0.25 m], 80 °C, 0.5–1.5 h. [f] 96 % recovery of biphenyl triflate.

With success in the preparation of a broad range of functionalized cyclic alkenylsulfonyl fluorides achieved, we then investigated derivatization reactions. As shown in Scheme 2, nucleophilic substitution at sulfur was achieved through the reaction of alkenylsulfonyl fluoride **5 b** with *p*‐methoxyphenol to form sulfonate ester **6** in 84 % yield, or with pyrrolidine to form sulfonamide **7** in 51 % yield. With the aid of stoichiometric Ca(NTf_2_)_2_ as a Lewis acid,3d anilines could also be used as nucleophiles to form alkenyl sulfonamides **8 a** and **8 b** in good yields. Alternatively, softer sulfur nucleophiles such as thiols and thiophenols underwent base‐catalyzed conjugate addition to the electron‐poor alkene in quantitative yields (**9 a**–**c**). Pd‐catalyzed hydrogenation of the alkene was also viable using balloon pressure H_2_, forming the saturated sulfonyl fluoride **10** in 71 % yield, thus demonstrating an efficient method to access saturated derivatives. Alkenylsulfonyl fluoride **5 r**, which features an N‐Bn group, could undergo an efficient one‐pot debenzylation/acylation using propargyl chloroformate^20^ in quantitative yield. The resultant alkyne‐bearing alkenylsulfonyl fluoride **11** could then undergo “click” copper(I)‐catalyzed azide–alkyne cycloaddition (CuAAC) with benzyl azide, providing triazole **12**. The Boc protecting group of sulfonyl fluoride **5 p** could be cleaved using 20 % (v/v) trifluoroacetic acid (TFA) in dichloromethane, forming ammonium salt **14**, which could then be further derivatized. Alternatively, by treating the same substrate with 4 n hydrochloric acid in dioxane, a hydrochloride salt of the deprotected amine **13** was isolated in 92 % yield as a bench‐stable white solid. The secondary ammonium salts **13** and **14** were able to react with various electrophiles; for example, reaction with phenyl isocyanate formed urea **17** in 74 % yield, and reaction with phenyl isothiocyanate generated thiourea **18** in 84 % yield. Notably, using this strategy, a fluorophore could be attached to the alkenylsulfonyl fluoride by the reaction with fluorescein isothiocyanate (FITC) isomer I, giving **19** in 65 % yield. Ammonium salt **13** underwent reductive amination with nicotinaldehyde to form tertiary amine **15** in 78 % yield, thus illustrating the tolerance of alkenylsulfonyl fluoride to such reductive conditions. In addition, ammonium salt **14** could be combined with an amino acid using T3P as coupling agent to form amide **20**; biotin was conjugated to ammonium **13** using HATU to form the biotinylated alkenylsulfonyl fluoride **16**. Together, the alkyne‐, fluorophore‐, and biotin‐derivatized alkenylsulfonyl fluorides are examples of multifunctional reagents primed for application to biological problems.

**Scheme 2:**
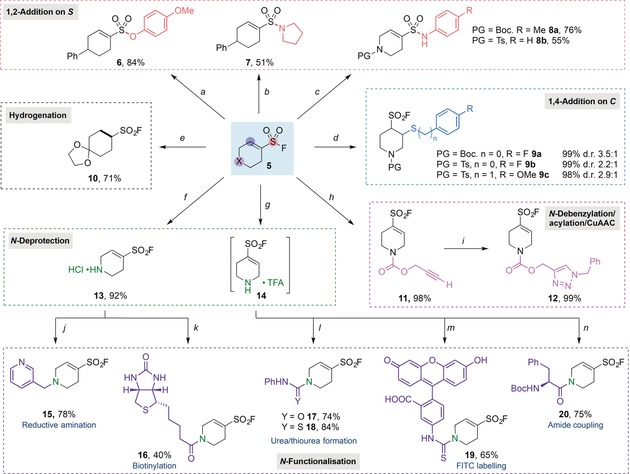
Functionalization of alkenylsulfonyl fluorides. Reaction conditions: Unless specified, 1 equiv of alkenylsulfonyl fluoride **5** was used. [a] X=CHPh; phenol (1.1 equiv), Cs_2_CO_3_ (2 equiv), CH_3_CN [0.2 m], rt, air, 1 h. [b] X=CHPh; pyrrolidine (5 equiv), CH_3_CN [0.1 m], 70 °C, air, 3 h. [c] X=NPG; aniline (2.2 equiv), Ca(NTf_2_)_2_ (1.1–2.0 equiv), *t*‐amyl‐OH [0.2 m], 60 °C, 16 h. [d] X=NPG; thiol (1.1 equiv), DBU (10 mol %), DCM [0.2 m], air, rt, 1–2 h. [e] X=C(OCH_2_CH_2_O); Pd/C (30 mol %), EtOAc [0.2 m], H_2_ (balloon), 40 °C, 24 h. [f] X=NBoc; HCl (4 N in dioxane) (10 equiv), 1,4‐dioxane [0.2 m], rt, 16 h. [g] X=NBoc; 20 % (v/v) TFA in DCM [0.1 m], rt, 30 min. [h] X=NBn; propargyl chloroformate (1.8 equiv), CHCl_3_ [0.33 m], −20 °C to 60 °C, 2 h. [i] Benzyl azide (1.1 equiv), CuSO_4_⋅5 H_2_O (5 mol %), sodium ascorbate (10 mol %), *t*‐BuOH/H_2_O (1:1) [0.23 m], air, 16 h. [j] Aldehyde (3 equiv), Et_3_N (1 equiv), NaBH(OAc)_3_ (3 equiv), DCM [0.2 m], rt, 16 h. [k] **13** (1.1 equiv), Biotin (1.0 equiv), HATU (1.05 equiv), Et_3_N (2.5 equiv), DMF [0.08 m], N_2_, rt, 16 h. [l] Et_3_N (1 equiv), PhNCO or PhNCS (1 equiv), THF [0.2 m], rt, 16 h. [m] Et_3_N (1 equiv), FITC isomer I (1 equiv), THF [0.2 m], rt, 16 h. [n] Boc‐Phe‐OH (1 equiv), Et_3_N (3 equiv), T3P (50 % in EtOAc) (2 equiv), DMF [0.1 m], 0 °C, 16 h. PG=protecting group, DBU=1,8‐diazabicyclo[5.4.0]undec‐7‐ene, Boc=*tert*‐butyloxycarbonyl, DCM=dichloromethane, TFA=trfluoroacetic acid, DMF=dimethylformamide, THF=tetrahydrofuran

In conclusion, we have reported an efficient and general synthesis of multifunctional alkenylsulfonyl fluorides. These Pd‐catalyzed reactions proceed from alkenyl triflates that are readily formed from commonly available ketones, and display good functional‐group tolerance. We have shown that templates in this new class of sulfonyl fluoride are able to undergo a variety of orthogonal derivatization processes, including nucleophilic substitution at sulfur, conjugate addition to the alkene, Pd‐catalyzed hydrogenation of the olefin, N‐functionalization, and a CuAAC click reaction. We anticipate that these attributes will result in these compact low‐molecular‐weight densely functionalized reagents being exploited in a variety of chemical biology, synthetic chemistry, and medicinal chemistry applications.

## Conflict of interest

The authors declare no conflict of interest.

## Supporting information

As a service to our authors and readers, this journal provides supporting information supplied by the authors. Such materials are peer reviewed and may be re‐organized for online delivery, but are not copy‐edited or typeset. Technical support issues arising from supporting information (other than missing files) should be addressed to the authors.

Supplementary
