# Supplementary material for: Cyclic Alkenylsulfonyl Fluorides: Palladium‐Catalyzed Synthesis and Functionalization of Compact Multifunctional Reagents
Source: Angew Chem Int Ed Engl. 2019 Nov 6;58(52):18859–63. doi: 10.1002/anie.201910871 (PMC6972694; doi:10.1002/anie.201910871)
Supplement: Supplementary file 1 — Supplementary [file ANIE-58-18859-s001.pdf]

Angewandte Chemie
Eine Zeitschrift der Gesellschaft Deutscher Chemiker

Supporting Information

Cyclic Alkenylsulfonyl Fluorides: Palladium-Catalyzed Synthesis and Functionalization of Compact Multifunctional Reagents

*Terry Shing-Bong Lou, Scott W. Bagley, and Michael C. Willis**

anie_201910871_sm_miscellaneous_information.pdf

Supporting Information

1 General Considerations S2

2 Table of Optimisation S3

3 Synthetic Procedures and Characterisation Data S5

3.1 Preparation of 1-iodocyclohept-1-ene (**1**) S5

3.2 Preparation of 1-bromocyclohept-1-ene (**2**) S6

3.3 Preparation of Alkenyl Triflates S7

3.4 Palladium-Catalysed Synthesis of Alkenylsulfonyl Fluorides S19

3.5 Derivatisation of Alkenylsulfonyl Fluorides S33

4 NMR Spectra of Alkenyl Triflates S53

5 NMR Spectra of Alkenylsulfonyl Fluorides Synthesized by Palladium Catalysis S88

6 Chiral HPLC Traces S157

7 Reference S158

1 General Considerations

Chemicals were purchased from Sigma-Aldrich, Alfa Aesar, Fluorochem or Acros and used without further purification, except TMSCl which was distilled, NBS which was recrystallised from hot water prior to use, and DABSO which was prepared by literature method.[1] $PdCl_2(AmPhos)_2$ pre-catalyst (dichlorobis[di-*tert*-butyl(4-dimethylaminophenyl) phosphine] palladium(II)) was purchased from Alfa Aesar (CAS# 887919-35-9, catalog number 45511). Solvents were purchased from Sigma Aldrich, Fisher Scientific or Rathburn and used without further purification, with the exception of isopropanol (IPA) which was distilled from Drierite® (with indicator, 8 mesh) onto 4 Å molecular sieves which had been activated by heating under vacuum (0.1 mbar) at 250 °C for 12 h. "Petroleum ether" or "petrol" refers to the fraction of petroleum ether boiling in the range 40–60 °C.

Reactions were performed with continuous magnetic stirring, under an atmosphere of nitrogen (passed through a Drierite® filled tube), unless otherwise specified. All glassware was dried in an oven (150 °C, overnight) and allowed to cool under a flow of nitrogen prior to use. "Reaction tube" refers to a 10 mL CEM microwave reaction vial. Cooling between –20 °C and –78 °C was achieved using a dry ice/acetone bath. Flash column chromatography was performed using Fluorochem Silicagel 60Å 40-63µ (particle size 0.040–0.063 nm) with the indicated eluents. Thin layer chromatography (TLC) was carried out on Merck Kieselgel 60 PF254 pre-coated aluminum backed sheets and visualised either by UV fluorescence (254 nm) and/or staining with potassium permanganate solution.

NMR spectra were recorded, unless specified, at ambient temperature on a Bruker AVIIIHD 400 MHz or AVII 500 MHz spectrometer. Proton-decoupled spectra are denoted as $\{^1H\}$. Chemical shifts (δ) are reported in parts per million (ppm) and referenced to the residual solvent peak(s). Coupling constants (J) are reported in Hertz (Hz). Assignments were made on the basis of chemical shifts, coupling constants, COSY, HSQC and comparison with spectra of related compounds. Signal multiplicities are denoted as: s, singlet; d, doublet; t, triplet; q, quartet; quint, quintet; sext, sextet; dd, doublet of doublets; dt, doublet of triplets; m, multiplet; br, broad; app., apparent.

Melting points were measured on a Leica Gallen III hot-stage microscope. Low resolution mass spectrometry (LRMS) was performed on a Fisons Platform spectrometer (ESI). High resolution mass spectrometry (HRMS) was performed *via* atmospheric pressure chemical ionisation (APCI) or electrospray ionisation (ESI) sources. *m/z* ratios are reported in Daltons; high resolution values are calculated to four decimal places from the molecular formula. Infrared (IR) spectra were recorded as thin films on a Bruker Tensor 27 FT-IR spectrometer with an internal range of 600–4000 cm^{-1}. Optical rotations were measured on a Schmidt Haensch UniPol L2000 polarimeter at 589 nm, 25 °C. $[\alpha]_D^T$ is expressed in deg cm^3 g^{-1} dm^{-1} and c is expressed in g/100 cm^3. The enantiomeric excess (ee) of the products was determined by chiral stationary phase HPLC in a Dionex P680 chromatograph with a Dionex UVD170U detector (Daicel Chiralpak IC column).

2 Table of Optimisation

Table S1. Initial optimisation of palladium-catalysed alkenylsulfonyl fluoride synthesis.[a]

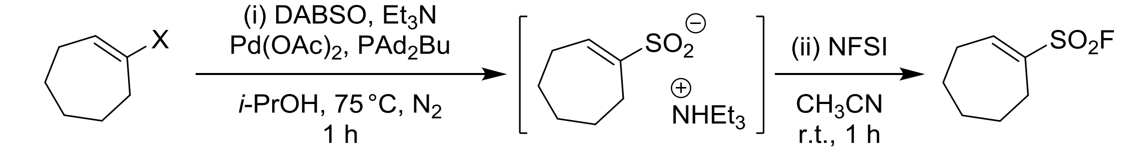

1, X = I; **2**, X = Br
3a, X = OTf

4a

5a

Entry	X	Variation	% Yield of **4a**[b]	% Yield of **5a**[c]
1	I	-	77	56
2	I	(ii) *i*-PrOH	-	37
3	I	(i) 16 h; (ii) *i*-PrOH	56	17[d]
4	I	(i) 2 h	-	50
5	I	(i) 24 h	0	-
6	I	(i) 50 °C, 8 h	-	39
7	I	(i) NMMSO (1.2 equiv.), 3 h	-	50
8	I	(i) TIMSO (1.2 equiv.), 1 h	-	33
9	I	(i) DABSO (1 equiv.), 1 h	-	53
10	I	(i) *t*-BuOH, Na(HCO_2), 24 h	10	-
11	I	(i) HFIP, 48 h	2	-
12	I	(i) SPhos instead of PAd_2Bu, 24 h	9	-
13	I	(i) MePhos instead of PAd_2Bu, 24 h	11	-
14	I	(i) $PdCl_2$(AmPhos)$_2$, 24 h	-	31
15	Br	(i) $PdCl_2$(AmPhos)$_2$, 1 h	-	52[d]
16	Br	(i) $PdCl_2$(AmPhos)$_2$, NMMSO (1.5 equiv.), 4 h	-	56[d]
17	OTf	-	-	36
**18**	**OTf**	**(i) $PdCl_2$(AmPhos)$_2$, 1 h**	**-**	**56 (56)[d]**

[a] Reaction conditions: (i) Alkenyl (pseudo)halide (0.3 mmol, 1 equiv.), DABSO (0.6 equiv.), Et_3N (3 equiv.), $Pd(OAc)_2$ (5 mol%), PAd_2(*n*-Bu) (7.5 mol%), *i*-PrOH [0.25 M], 75 °C, N_2, 1 h; (ii) NFSI (1.5 equiv.), CH_3CN [0.25 M], r.t., 1 h. [b] HPLC yield. [c] NMR yield. [d] Isolated yield. Ad = adamantyl. AmPhos = di-*tert*-butyl(4-dimethylaminophenyl)phosphine. HFIP = Hexafluoroisopropanol. NMMSO = *N*-methylmorpholine-SO_2 adduct. TIMSO = *N*-methylpyrrolidine-SO_2 adduct.

Ligand and Chemical Structures:

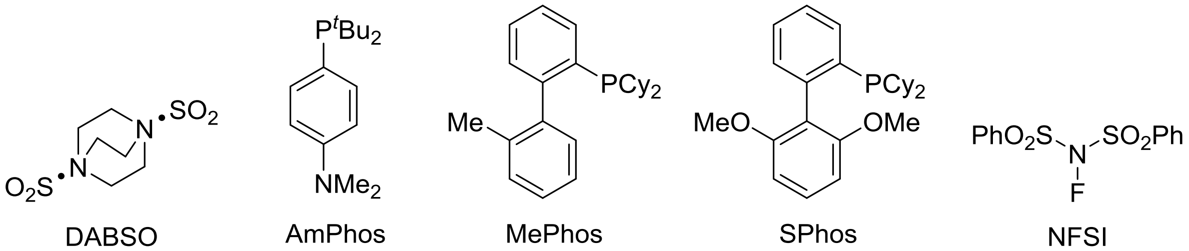

DABSO AmPhos MePhos SPhos NFSI

Table S2. Optimisation of palladium-catalysed alkenylsulfonyl fluoride synthesis using alkenyl triflate.[a]

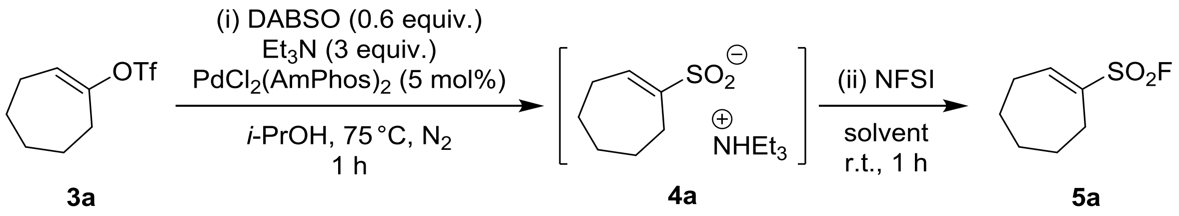

Entry	Variation	Solvent for (ii)	% Yield of **5a**[b]
1	-	CH_3CN	56
2	(i) NMMSO (1.2 equiv.), 6 h	CH_3CN	41
3	(i) Cy_2NMe	CH_3CN	51
4	(i) Cy_2NMe	*i*-PrOH/CH_3CN (1:1)	49
5	(i) 80 °C, 30 min	CH_3CN	53
6	-	*i*-PrOH	54
7	-	*i*-PrOH/CH_3CN (1:1)	46
8	-	CH_2Cl_2	48
9	-	THF	53
10	**-**	**EtOAc**	**67 (70)[c]**
**11**	-	DMF	61
12	-	Et_2O	54
13	-	Acetone	59
14	(i) 80 °C, 30 min	EtOAc	67
15	- [d]	EtOAc	67[c]
16	- [d]	CH_3CN	63
17	(i) DABSO (1 equiv.)[d]	EtOAc	65
18	(i) DABSO (2 equiv.)[d]	EtOAc	40
19	(i) Cy_2NMe	EtOAc	55
20	(i) Cy_2NMe[d]	EtOAc	57
21	(i) DIPEA	EtOAc	68
22	(i) DIPEA[d]	EtOAc	52

[a] Reaction conditions: (i) Alkenyl triflate **3a** (0.3 mmol, 1 equiv.), DABSO (0.6 equiv.), Et_3N (3 equiv.), $PdCl_2$(AmPhos) (5 mol%), *i*-PrOH [0.25 M], 75 °C, N_2, 1 h; (ii) NFSI (1.5 equiv.), solvent [0.25 M], r.t., 1 h. [b] NMR yield. [c] Isolated yield. [d] An *i*-PrOH solution of Pd catalyst, Et_3N and DABSO was heated to 75 °C for 15 min prior to the addition of **3a**. AmPhos = di-*tert*-butyl(4-dimethylamino-phenyl)-phosphine. Cy = cyclohexyl. DIPEA = *N*,*N*-diisopropylethylamine. DMF = dimethylformamide. NMMSO = *N*-methylmorpholine-SO_2 adduct. THF = tetrahydrofuran.

3 Synthetic Procedures and Characterisation Data

3.1 Preparation of 1-iodocyclohept-1-ene (1)[2]

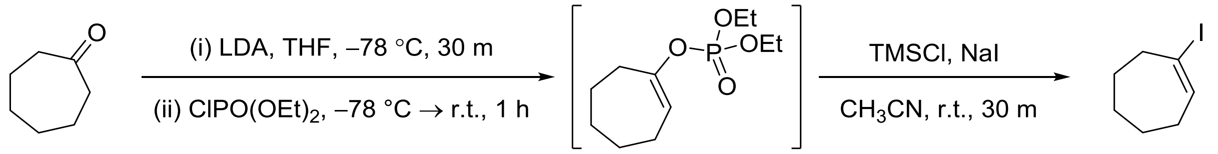

1

According to the procedure of Lee and Wiemer,[3] a two-neck round bottom flask was charged with cycloheptanone (1.2 mL, 10 mmol, 1.0 equiv.), and was evacuated and filled with N_2 three times. After the addition of anhydrous THF (10 mL), the mixture was cooled to −78 °C, followed by the addition of LDA (1.0 M in THF, 11 mL, 11 mmol, 1.1 equiv.). The reaction was stirred at −78 °C for 30 min under a positive pressure of N_2. A solution of diethyl chlorophosphate (2.9 mL, 20 mmol, 2.0 equiv.) in anhydrous THF (10 mL) was then added to the reaction. The reaction mixture was stirred at −78 °C for 30 min, then warmed up to ambient temperature and further stirred for a further hour. To the reaction mixture, sat. aq. NH_4Cl (30 mL) was added dropwise at 0 °C, and the THF was removed *in vacuo*, then the crude mixture extracted with ethyl acetate for three times. The combined organic layers were then dried with anhydrous $MgSO_4$, filtered and concentrated *in vacuo*. The crude product was used in the next step without further purification.

To the crude alkenyl phosphate was added NaI (4.5 g, 30 mmol, 3.0 equiv.), then dried under vacuum (1 mbar) for an hour. The mixture was dissolved in anhydrous acetonitrile (25 mL), and TMSCl (3.8 mL, 30 mmol, 3.0 equiv.) was added dropwise at room temperature. The reaction was stirred for 30 min under a positive pressure of N_2, which was then cooled to 0 °C and quenched by slow addition of sat. aq. NH_4Cl (2 mL). The mixture was diluted with Et_2O and filtered. The filtrate was then washed subsequently with sat. aq. $NaHCO_3$ and 10% (w/w) aq. $Na_2S_2O_3$, dried with anhydrous $MgSO_4$, filtered and concentrated *in vacuo*. The crude product was then purified by flash column chromatography (petrol), affording the corresponding alkenyl iodide **1** as a colourless oil (0.97 g, 44%) with spectroscopic data in accordance with the literature;

^{1}H NMR (400 MHz, $CDCl_3$) δ 6.51 (t, J = 6.6 Hz, 1H, C=CH), 2.78–2.72 (m, 2H, CHCH_2), 2.09–2.01 (m, 2H, CCH_2), 1.76–1.68 (m, 2H, Alk-CH_2), 1.58–1.50 (m, 4H, 2 × Alk-CH_2);

^{13}C {^{1}H} NMR (101 MHz, $CDCl_3$) δ 143.0, 100.8, 45.1, 31.5, 31.1, 26.8, 26.5;

IR ν_{max} (neat)/cm^{-1} 2922, 2850, 1453, 1279, 1216, 1105, 1031.

3.2 Preparation of 1-bromocyclohept-1-ene (2)[4]

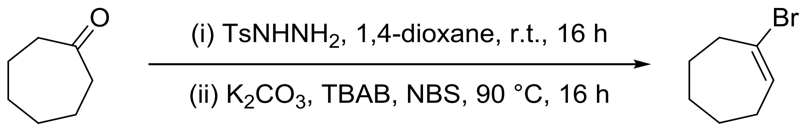

2

According to the procedure of Ojha and Prabhu,[5] a solution of cycloheptanone (0.59 mL, 5.0 mmol, 1.0 equiv.) and *p*-toluenesulfonyl hydrazide (0.93 g, 5.0 mmol, 1.0 equiv.) in 1,4-dioxane (10 mL) was stirred at ambient temperature for 16 h, followed by the addition of K_2CO_3 (2.1 g, 15 mmol, 3.0 equiv.), TBAB (4.8 g, 15 mmol, 3.0 equiv.) and NBS (1.34 g, 7.5 mmol, 1.5 equiv.). The mixture was heated to 90 °C and stirred for 16 h under a positive pressure of N_2. The reaction mixture was then cooled to room temperature and poured into a 1:1 mixture of petrol and EtOAc (100 mL). The solution was filtered through a plug of silica and concentrated *in vacuo* to yield the crude product which was then purified by flash column chromatography (petrol), affording the alkenyl bromide **2** as a colourless oil (237 mg, 27%) with spectroscopic data in accordance with the literature;

^{1}H NMR (400 MHz, $CDCl_3$) δ 6.19 (t, J = 6.5 Hz, 1H, C=C*H*), 2.71–2.64 (m, 2H, CHCH_2), 2.11–2.03 (m, 2H, CCH_2), 1.75–1.66 (m, 2H, Alk-CH_2), 1.66–1.52 (m, 4H, 2 × Alk-CH_2);

^{13}C {^{1}H} NMR (101 MHz, $CDCl_3$) δ 133.5, 126.2, 40.7, 30.7, 29.2, 26.4, 26.3;

IR ν_{max} (neat)/cm^{-1} 2929, 2851, 1723, 1447, 1265, 1100, 1018, 730.

3.3 Preparation of Alkenyl Triflates

General Procedure A: Synthesis of alkenyl triflates from the corresponding ketone, LiHMDS and $PhNTf_2$ as exemplified by the preparation of 1-cyclohept-1-enyl trifluoromethanesulfonate (3a)[6]

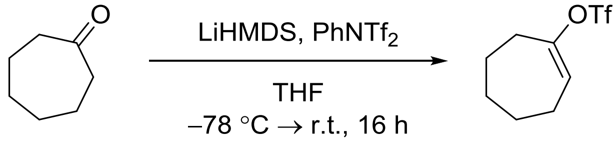

3a

A two-neck round bottom flask was charged with cycloheptanone (0.70 mL, 6.0 mmol, 1.0 equiv.) and $PhNTf_2$ (2.36 g, 6.6 mmol, 1.1 equiv.), then evacuated and back-filled with N_2 three times, followed by the addition of anhydrous THF (25 mL). The mixture was cooled to −78 °C, and LiHMDS (1 M in THF) (6.6 mL, 6.6 mmol, 1.1 equiv.) was added slowly. The reaction was allowed to warm up gradually to ambient temperature over 16 h. To the reaction mixture, sat. aq. NH_4Cl solution (2 mL) was added, and was concentrated *in vacuo*. To the mixture was added Et_2O (20 mL) and 2 M aq. NaOH solution (10 mL), and the mixture was stirred vigorously for 15 minutes. The organic layer was separated and washed with 2 M aq. NaOH solution and brine, then dried with anhydrous $MgSO_4$, filtered and concentrated *in vacuo* to leave the crude product, which was then purified by flash column chromatography (petrol), affording the corresponding alkenyl triflate **3a** as a colourless oil (1.16 g, 79%) with spectroscopic data in accordance with the literature;[6]

^{1}H NMR (400 MHz, $CDCl_3$) δ 5.88 (t, J = 6.4 Hz, 1H, OC=CH), 2.55–2.48 (m, 2H, OCCH_2), 2.20–2.12 (m, 2H, CHCH_2), 1.76–1.59 (m, 6H, 3 × Alk-CH_2);

^{13}C {^{1}H} NMR (101 MHz, $CDCl_3$) δ 153.2, 123.2, 118.7 (q, $^1J_{CF}$ = 320.3 Hz), 33.3, 30.0, 26.5, 24.92, 24.87;

^{19}F {^{1}H} NMR (377 MHz, $CDCl_3$) δ −74.0;

IR ν_{max} (neat)/cm^{-1} 2889, 1419, 1205, 1141, 990, 869.

4-Phenyl-1-cyclohexen-1-yl trifluoromethanesulfonate (3b)[7]

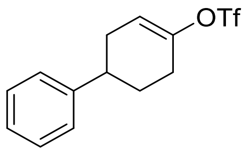

Prepared according to General Procedure A using 4-phenylcyclohexan-1-one (1.74 g, 10 mmol). The crude product was purified by flash column chromatography (petrol) to afford the corresponding alkenyl triflate **3b** as a white crystalline solid (1.92 g, 63%) with spectroscopic data in accordance with the literature;

mp 27–28 °C (hexane);

^{1}H NMR (400 MHz, $CDCl_3$) δ 7.33 (t, J = 7.3 Hz, 2H, 2 × Ar*H*), 7.26–7.19 (m, 3H, 3 × Ar*H*), 5.86 (dt, J = 4.8, 2.1 Hz, 1H, OC=C*H*), 2.90–2.80 (m, 1H, ArC*H*), 2.62–2.27 (m, 4H, CH_2 + C=CHCH_2), 2.14–2.02 (m, 1H, $CCH_2CH_aH_b$), 2.04–1.89 (m, 1H, $CCH_2CH_aH_b$);

^{13}C {^{1}H} NMR (101 MHz, $CDCl_3$) δ 149.1, 144.7, 128.8, 126.9, 126.8, 118.7 (q, $^1J_{CF}$ = 320.0 Hz), 118.3, 38.9, 31.7, 29.8, 28.0;

^{19}F {^{1}H} NMR (377 MHz, $CDCl_3$) δ −74.0;

IR ν_{max} (neat)/cm^{-1} 3031, 2929, 1692, 1416, 1246, 1208, 1142, 1054, 1025.

4-(*tert*-Butyl)cyclohex-1-en-1-yl trifluoromethanesulfonate (3c)[8]

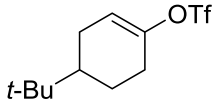

Prepared according to General Procedure A using 4-*tert*-butylcyclohexanone (0.46 g, 3.0 mmol). The crude product was purified by flash column chromatography (petrol) to afford the corresponding alkenyl triflate **3c** as a colourless oil (0.75 g, 87%) which solidified at -20 °C, with spectroscopic data in accordance with the literature;

^{1}H NMR (400 MHz, $CDCl_3$) δ 5.74 (dt, J = 5.9, 2.4 Hz, 1H, OC=C*H*), 2.45–2.27 (m, 2H, CCH_2), 2.25–2.16 (m, 1H, $C(CH_3)_3CH$), 2.00–1.89 (m, 2H, C=CHCH_2), 1.44–1.25 (m, 2H, CCH_2CH_2), 0.89 (s, 9H, $C(CH_3)_3$);

^{13}C {^{1}H} NMR (101 MHz, $CDCl_3$) δ 149.4, 118.7 (q, $^1J_{CF}$ = 320.5 Hz), 118.6, 43.1, 32.2, 28.7, 27.3, 25.5, 24.2;

^{19}F {^{1}H} NMR (377 MHz, $CDCl_3$) δ −74.1.

4,4-Difluorocyclohex-1-en-1-yl trifluoromethanesulfonate (3d)

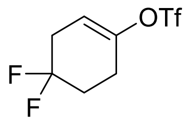

Prepared according to General Procedure A using 4,4-difluorocyclohexan-1-one (0.67 g, 5.0 mmol). The crude product was purified by flash column chromatography (0–5% Et_2O in petrol) to afford the corresponding *alkenyl triflate* **3d** as a light yellow oil (0.96 g, 72%);

^{1}H NMR (400 MHz, $CDCl_3$) δ 5.70–5.65 (m, 1H, OC=C*H*), 2.70 (td, *J* = 13.4, 3.3 Hz, 2H, $CHCH_2$), 2.60 (t, *J* = 6.6 Hz, 2H, $CF_2CH_2CH_2$), 2.21 (tt, *J* = 13.4, 6.7 Hz, 2H, $CF_2CH_2CH_2$);

^{13}C {^{1}H} NMR (101 MHz, $CDCl_3$) δ 147.2, 120.5 (t, $^1J_{CF}$ = 241.1 Hz), 118.5 (q, $^1J_{CF}$ = 320.5 Hz), 113.8 (t, $^3J_{CF}$ = 5.9 Hz), 33.1 (t, $^2J_{CF}$ = 28.7 Hz), 30.1 (t, $^2J_{CF}$ = 25.8 Hz), 25.4 (t, $^3J_{CF}$ = 5.6 Hz);

^{19}F NMR (377 MHz, $CDCl_3$) δ −73.8 (s, 3F, CF_3), −98.6 (quint, $^3J_{H\text{-}F}$ = 13.2 Hz, 2F, CF_2);

IR ν_{max} (neat)/cm^{-1} 1694, 1418, 1382, 1247, 1206, 1141, 1122, 1086, 1066, 1041, 899, 863;

HRMS (APCI$^+$) found *m/z* 247.0034 $[M-F]^+$, $C_7H_7F_4O_3S$ requires *m/z* 247.0047.

Ethyl 4-(((trifluoromethyl)sulfonyl)oxy)cyclohex-3-ene-1-carboxylate (3e)[9]

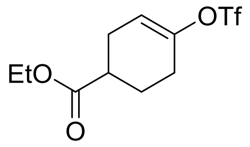

Prepared according to General Procedure A using ethyl cyclohexanone-4-carboxylate (0.48 mL, 3.0 mmol). The crude product was purified by flash column chromatography (0–10% EtOAc in petrol) to afford the corresponding alkenyl triflate **3e** as a colourless oil (0.85 g, 94%) with spectroscopic data in accordance with the literature;

^{1}H NMR (400 MHz, $CDCl_3$) δ 5.79 (t, *J* = 3.6 Hz, 1H, OC=C*H*), 4.18 (q, *J* = 7.2 Hz, 2H, C(=O)OCH_2), 2.67–2.55 (m, 1H, C(=O)C*H*), 2.51–2.38 (m, 4H, C=CHCH_2 + CH=CCH_2), 2.21–2.10 (m, 1H, $SCCH_2CH_aH_b$), 2.01–1.86 (m, 1H, $SCCH_2CH_aH_b$), 1.28 (td, *J* = 7.2, 1.4 Hz, 3H, CH_3);

^{13}C {^{1}H} NMR (101 MHz, $CDCl_3$) δ 173.9, 148.4, 118.5 (q, $^1J_{CF}$ = 319.2 Hz), 116.9, 60.8, 37.8, 26.6, 26.1, 25.0, 14.2;

^{19}F {^{1}H} NMR (377 MHz, $CDCl_3$) δ −74.0;

IR ν_{max} (neat)/cm^{-1} 2985, 2941, 1731, 1416, 1247, 1203, 1179, 1140, 1054, 1033, 872.

4-Methoxycyclohex-1-en-1-yl trifluoromethanesulfonate (3f)[10]

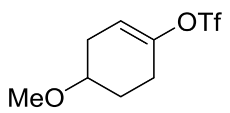

Prepared according to General Procedure A using 4-methoxycyclohexan-1-one (384 mg, 3.0 mmol). The crude product was purified by flash column chromatography (10–20% Et_2O in petrol) to afford the corresponding alkenyl triflate **3f** as a colourless oil (605 mg, 78%) with spectroscopic data in accordance with the literature;

^{1}H NMR (400 MHz, $CDCl_3$) δ 5.64 (q, J = 3.0, 2.3 Hz, 1H, OC=CH), 3.54–3.47 (m, 1H, OCH), 3.36 (s, 3H, OCH_3), 2.52–2.39 (m, 2H, CCH_2), 2.41–2.28 (m, 1H, C=CHCH_aH$_b$), 2.29–2.16 (m, 1H, C=CHCH$_a$$H_b$), 2.02–1.81 (m, 2H, CCH$_2CH_2$);

^{13}C {^{1}H} NMR (101 MHz, $CDCl_3$) δ 148.5, 118.6 (q, $^{1}J_{CF}$ = 319.8 Hz), 115.6, 73.2, 56.3, 29.5, 26.9, 25.1;

^{19}F {^{1}H} NMR (377 MHz, $CDCl_3$) δ −74.0;

4-((*tert*-Butoxycarbonyl)amino)cyclohex-1-en-1-yl trifluoromethanesulfonate (3g)[11]

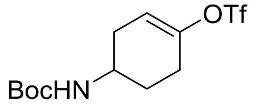

Prepared according to General Procedure A using *tert*-butyl (4-oxocyclohexyl)carbamate (639 mg, 3.0 mmol). The crude product was purified by flash column chromatography (10–20% EtOAc in petrol) to afford a creamy yellow solid which was then recrystallised from hot hexane to yield the corresponding alkenyl triflate **3g** as a white solid (336 mg, 32%) with spectroscopic data in accordance with the literature;

mp 75–77 °C (hexane);

^{1}H NMR (400 MHz, $CDCl_3$) δ 5.71–5.67 (m, 1H, OC=CH), 4.55–4.46 (m, 1H, NH), 3.87–3.79 (m, 1H, NHCH), 2.59–2.35 (m, 3H, CCH_2 + C=CHCH_aH$_b$), 2.11–1.96 (m, 2H, C=CHCH$_a$$H_b$ + CCH$_2$CH_cH$_d$), 1.83–1.73 (m, 1H, CCH$_2$CH$_c$$H_d$), 1.45 (s, 9H, C(C$H_3$)$_3$);

^{13}C {^{1}H} NMR (101 MHz, $CDCl_3$) δ 155.3, 148.4, 118.6 (q, $^{1}J_{CF}$ = 319.8 Hz), 116.3, 79.9, 44.4, 30.5, 28.5, 28.4, 25.9;

^{19}F {^{1}H} NMR (377 MHz, $CDCl_3$) δ −74.0;

1,4-Dioxaspiro[4.5]dec-7-en-8-yl trifluoromethanesulfonate (3h)[12]

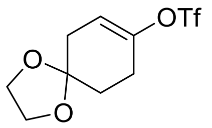

Prepared according to General Procedure A using 1,4-dioxaspiro[4.5]decan-8-one (2.0 g, 13 mmol). The crude product was purified by flash column chromatography (5–20% Et_2O in petrol) to afford the corresponding alkenyl triflate **3h** as a yellow oil (2.84 g, 76%) with spectroscopic data in accordance with the literature;

^{1}H NMR (400 MHz, $CDCl_3$) δ 5.69–5.63 (m, 1H, OC=C*H*), 4.25–3.69 (m, 4H, 2 × OCH_2), 2.57–2.51 (m, 2H, CH=CCH_2), 2.42–2.38 (m, 2H, CHCH_2), 1.90 (tt, J = 6.6, 0.8 Hz, 2H, CH=CCH_2CH_2);

^{13}C {^{1}H} NMR (101 MHz, $CDCl_3$) δ 148.3, 118.6 (q, $^1J_{CF}$ = 320.2 Hz), 116.0, 106.2, 64.8, 34.3, 31.1, 26.5;

^{19}F {^{1}H} NMR (377 MHz, $CDCl_3$) δ −76.0;

IR ν_{max} (neat)/cm^{-1} 2889, 1692, 1416, 1375, 1247, 1205, 1141, 1068, 1041, 1014.

3-(1-Methyl-1*H*-indol-3-yl)cyclohex-1-en-1-yl trifluoromethanesulfonate (3i)[13]

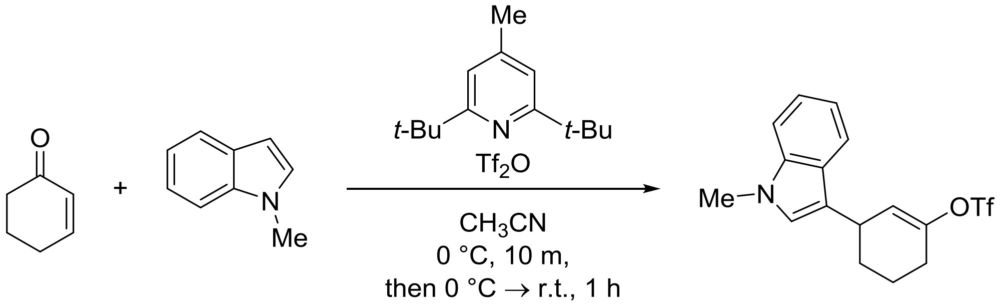

Prepared according to a modified literature procedure: 2,6-Di-*tert*-butyl-4-methylpyridine (0.74 g, 3.6 mmol, 1.2 equiv.) was dissolved in acetonitrile (15 mL), followed by the addition of cyclohex-2-en-1-one (0.29 mL, 3.0 mmol, 1.0 equiv.) and 1-methylindole (1.12 mL, 9.0 mmol, 3.0 equiv.). The mixture was cooled to 0 °C and triflic anhydride (0.56 mL, 3.3 mmol, 1.1 equiv.) was added with vigorous stirring. The reaction was stirred for 10 min at 0 °C, and sat. aq. $NaHCO_3$ solution (6 mL) was added. The mixture was stirred and allowed to warm up to ambient temperature over an hour, then diluted with water and extracted with CH_2Cl_2 three times. The combined organic extract was dried over anhydrous $MgSO_4$, filtered and concentrated *in vacuo* to leave the crude product, which was then purified by flash column chromatography (10–20% CH_2Cl_2 in petrol) without delay to afford the corresponding alkenyl triflate **3i** as a colourless oil (644 mg, 60%) with spectroscopic data in accordance with the literature;

^{1}H NMR (400 MHz, $CDCl_3$) δ 7.60 (d, J = 7.9 Hz, 1H, Ar*H*), 7.32 (d, J = 8.2 Hz, 1H, Ar*H*), 7.25 (t, J = 7.1 Hz, 1H, Ar*H*), 7.13 (t, J = 7.0 Hz, 1H, Ar*H*), 6.82 (s, 1H, NC*H*), 5.98–5.94 (m, 1H, OC=C*H*), 3.99–3.92 (m, 1H, C=CHC*H*), 3.76 (s, 3H, NCH_3), 2.45–2.40 (m, 2H, CH=CCH_2), 2.05–1.96 (m, 1H, C=CHCHCH_aH$_b$), 1.92–1.83 (m, 1H, C=CHCHCH$_a$$H_b$), 1.82–1.75 (m, 2H, CCH$_2CH_2$);

^{13}C {^{1}H} NMR (101 MHz, $CDCl_3$) δ 150.0, 137.4, 126.9, 126.6, 122.0, 121.8, 119.1, 118.9, 118.7 (app. d, $^1J_{CF}$ = 319.6 Hz), 116.8, 109.6, 32.9, 32.3, 29.0, 27.9, 20.6;

^{19}F {^{1}H} NMR (377 MHz, $CDCl_3$) δ −74.1;

3-(5-Methylfuran-2-yl)cyclohex-1-en-1-yl trifluoromethanesulfonate (3k)[13]

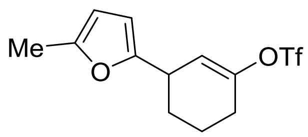

Prepared according to the procedure for compound **3i** as mentioned above using cyclohex-2-en-1-one (0.29 mL, 3.0 mmol), 2-methylfuran (0.81 mL, 9.0 mmol), 2,6-di-*tert*-butyl-4-methylpyridine (0.74 g, 3.6 mmol), triflic anhydride (0.56 mL, 3.3 mmol) and acetonitrile (15 mL). The crude product was purified by flash column chromatography (10–20% CH_2Cl_2 in petrol) without delay to afford the corresponding alkenyl triflate **3s** as a yellow oil (196 mg, 21%) with spectroscopic data in accordance with the literature;

^{1}H NMR (400 MHz, $CDCl_3$) δ 5.89 (dd, J = 3.1, 0.8 Hz, 1H, ArH), 5.87–5.85 (m, 2H, ArH + OC=CH), 3.67–3.62 (m, 1H, C=CHCH), 2.39–2.34 (m, 2H, CH=CCH_2), 2.26 (s, 3H, CH_3), 1.96–1.81 (m, 2H, CHCH_2), 1.80–1.72 (m, 2H, CCH_2CH_2);

^{13}C {^{1}H} NMR (101 MHz, $CDCl_3$) δ 154.1, 151.4, 150.7, 119.0, 118.7 (q, $^1J_{CF}$ = 320.3 Hz), 106.5, 106.1, 34.6, 27.7, 27.0, 20.4, 13.7;

^{19}F {^{1}H} NMR (377 MHz, $CDCl_3$) δ −73.9;

6-Methylcyclohex-1-en-1-yl trifluoromethanesulfonate (3l)[9]

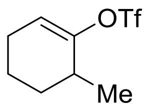

Prepared according to General Procedure A using 2-methylcyclohexan-1-one (0.37 mL, 3.0 mmol). The crude product was purified by flash column chromatography (petrol) to afford the corresponding alkenyl triflate **3l** as a colourless oil (388 mg, 53%) with spectroscopic data in accordance with the literature;

^{1}H NMR (400 MHz, $CDCl_3$) δ 5.73 (td, J = 4.1, 1.5 Hz, 1H, OC=CH), 2.59–2.49 (m, 1H, CH_3CH), 2.20–2.14 (m, 2H, C=CHCH_2), 1.97–1.89 (m, 1H, $CH_3CHCH_aH_b$), 1.71–1.61 (m, 1H, $CHCH_2CH_cH_d$), 1.61–1.52 (m, 1H, $CHCH_2CH_cH_d$), 1.51–1.42 (m, 1H, $CH_3CHCH_aH_b$), 1.14 (d, J = 6.9 Hz, 3H, CH_3);

^{13}C {^{1}H} NMR (101 MHz, $CDCl_3$) δ 153.5, 118.5 (q, $^1J_{CF}$ = 319.9 Hz), 118.3, 32.5, 31.6, 24.6, 19.4, 18.0;

^{19}F {^{1}H} NMR (377 MHz, $CDCl_3$) δ −74.3.

1,1-Dioxido-3,6-dihydro-2*H*-thiopyran-4-yl trifluoromethanesulfonate (3m)

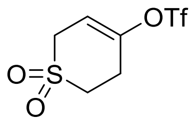

Prepared according to General Procedure A using tetrahydro-4*H*-thiopyran-4-one 1,1-dioxide (296 mg, 2.0 mmol). The crude product was purified by flash column chromatography (20–30% EtOAc in petrol) to afford the corresponding *alkenyl triflate* **3m** as a white solid (160 mg, 29%).

mp 81–83 °C (EtOAc);

^{1}H NMR (400 MHz, $CDCl_3$) δ 5.82 (tt, *J* = 4.5, 1.2 Hz, 1H, OC=C*H*), 3.79–3.76 (m, 2H, C=CHCH_2), 3.26 (tt, *J* = 6.7, 1.3 Hz, 2H, SCH_2CH$_2$), 3.10–3.01 (m, 2H, SCH$_2$CH_2);

^{13}C {^{1}H} NMR (101 MHz, $CDCl_3$) δ 147.1, 118.5 (app. d, $^{1}J_{CF}$ = 320.6 Hz), 112.7, 48.7, 47.0, 28.2;

^{19}F {^{1}H} NMR (377 MHz, $CDCl_3$) δ −73.4;

IR ν_{max} (neat)/cm^{-1} 2930, 2852, 1415, 1318, 1300, 1290, 1204, 1125, 1064, 1038;

HRMS (ESI/APCI) not found.

3,6-Dihydro-2*H*-pyran-4-yl trifluoromethanesulfonate (3n)[14]

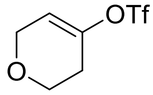

Prepared according to General Procedure A using tetrahydro-4*H*-pyran-4-one (0.46 mL, 5.0 mmol). The crude product was purified by flash column chromatography (5–20% Et_2O in pentane) to afford the corresponding alkenyl triflate **3n** as a colourless oil (678 mg, 58%) with spectroscopic data in accordance with the literature;

^{1}H NMR (400 MHz, $CDCl_3$) δ 5.86–5.78 (m, 1H, OC=C*H*), 4.26 (app. q, *J* = 2.9 Hz, 2H, CHCH_2), 3.89 (t, *J* = 5.5 Hz, 2H, OCH_2CH$_2$), 2.49–2.42 (m, 2H, OCH$_2$CH_2);

^{13}C {^{1}H} NMR (101 MHz, $CDCl_3$) δ 145.9, 118.6 (q, $^{1}J_{CF}$ = 320.3 Hz), 117.1, 64.3, 64.1, 28.5;

^{19}F {^{1}H} NMR (377 MHz, $CDCl_3$) δ −73.9;

IR ν_{max} (neat)/cm^{-1} 2846, 1695, 1416, 1357, 1248, 1203, 1138, 1064, 1008, 872.

Triflate 3o and 3o'[15]

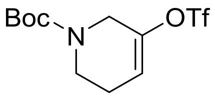

3l

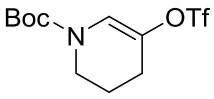

3l'

Prepared according to General Procedure A using *tert*-butyl 3-oxopiperidine-1-carboxylate (0.60 g, 3.0 mmol). The crude product was purified by flash column chromatography (5–15% Et_2O in petrol) to afford the corresponding alkenyl triflate **3o** as a yellow oil (223 mg, 22%) and its regioisomer **3o'** as a yellow oil (416 mg, 42%) with spectroscopic data in accordance with the literature;

***tert*-Butyl 5-(((trifluoromethyl)sulfonyl)oxy)-3,6-dihydropyridine-1(2*H*)-carboxylate (3o)**

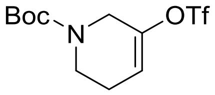

^{1}H NMR (400 MHz, $CDCl_3$) δ 5.92 (t, J = 4.6 Hz, 1H, OC=C*H*), 4.08–3.99 (m, 2H, CC*H*$_2$N), 3.49 (t, J = 5.7 Hz, 2H, NC*H*$_2$CH$_2$), 2.34–2.26 (m, 2H, NCH$_2$C*H*$_2$), 1.47 (d, J = 1.5 Hz, 9H, C(C*H*$_3$)$_3$);

^{13}C {^{1}H} NMR (101 MHz, $CDCl_3$) δ 154.4, 144.9, 118.6 (app. d, $^1J_{CF}$ = 320.4 Hz), 117.4, 80.9, 44.4, 39.0, 28.5, 23.9;

^{19}F {^{1}H} NMR (377 MHz, $CDCl_3$) δ −73.5;

***tert*-Butyl 5-(((trifluoromethyl)sulfonyl)oxy)-3,4-dihydropyridine-1(2*H*)-carboxylate (3o')**

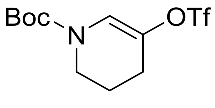

^{1}H NMR (400 MHz, $CDCl_3$) δ 7.24 (br. s, 0.4H, OC=CH^{rot1}), 7.06 (br. s, 0.6H, OC=CH^{rot2}), 3.56–3.47 (m, 2H, NC*H*$_2$), 2.43 (t, J = 6.4 Hz, 2H, CC*H*$_2$), 1.93 (quint, J = 6.1 Hz, 2H, NCH$_2$C*H*$_2$), 1.49 (app. d, J = 1.4 Hz, 9H, C(C*H*$_3$)$_3$) (recorded as a pair of rotamers);

^{13}C {^{1}H} NMR (101 MHz, $CDCl_3$) δ 151.5, {134.3, 133.4}, {122.2, 121.8}, 118.7 (app. d, $^1J_{CF}$ = 321.0 Hz), 82.3, {41.8, 40.6}, 28.3, 25.4, {21.1, 20.8}(recorded as a pair of rotamers);

^{19}F {^{1}H} NMR (377 MHz, $CDCl_3$) δ −73.6;

tert-Butyl 4-(((trifluoromethyl)sulfonyl)oxy)-3,6-dihydropyridine-1(2*H*)-carboxylate (3p)[16]

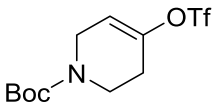

Prepared according to General Procedure A using *tert*-butyl 4-oxopiperidine-1-carboxylate (0.60 g, 3.0 mmol). The crude product was purified by flash column chromatography (10–20% Et_2O in petrol) to afford the corresponding alkenyl triflate **3p** as a low-melting white solid (0.59 g, 60%) with spectroscopic data in accordance with the literature;

mp 27-28 °C (CH_2Cl_2);

^{1}H NMR (400 MHz, $CDCl_3$) δ 5.76 (br. s, 1H, OC=C*H*), 4.08–4.00 (m, 2H, $CHCH_2$), 3.62 (t, J = 5.8 Hz, 2H, CCH_2CH_2), 2.48–2.39 (m, 2H, CCH_2), 1.47 (app. d, J = 1.7 Hz, 9H, $C(CH_3)_3$) (recorded as a pair of rotamers);

^{13}C {^{1}H} NMR (101 MHz, $CDCl_3$) δ 154.4, 147.0, 118.6 (q, $^1J_{CF}$ = 320.2 Hz), 115.6, 80.7, 41.9, 40.9, 39.9, {28.5, 28.2} (recorded as a pair of rotamers);

^{19}F {^{1}H} NMR (377 MHz, $CDCl_3$) δ −73.9;

1-Benzyloxycarbonyl-1,2,3,6-tetrahydropyridin-4-yl trifluoromethanesulfonate (3q)[17]

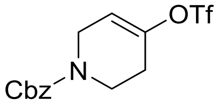

Prepared according to General Procedure A using 1-benzyloxycarbonylpiperidin-4-one (2.33 g, 10.0 mmol). The crude product was purified by flash column chromatography (5–20% EtOAc in petrol) to afford the corresponding alkenyl triflate **3q** as a light brown oil (1.87 g, 51%) with spectroscopic data in accordance with the literature;

^{1}H NMR (400 MHz, $CDCl_3$) δ 7.41–7.31 (m, 5H, 5 × Ar*H*), 5.80 (br. s, 0.5H, OC=CH^{rot1}), 5.75 (br. s, 0.5H, OC=CH^{rot2}), 5.16 (s, 2H, $ArCH_2$), 4.13 (s, 2H, $CHCH_2$), 3.72 (t, J = 5.5 Hz, 2H, NCH_2CH_2), 2.47 (app. s, 2H, NCH_2CH_2) (recorded as a pair of rotamers);

^{13}C {^{1}H} NMR (101 MHz, $CDCl_3$) δ 155.1, {147.0, 146.6}, 136.3, 128.7, 128.4, 128.2, 118.6 (q, $^1J_{CF}$ = 320.3 Hz), {115.9, 115.3}, 67.8, 42.0, 40.6, {28.3, 28.0} (recorded as a pair of rotamers);

^{19}F {^{1}H} NMR (377 MHz, $CDCl_3$) δ −73.7, −73.8 (recorded as a pair of rotamers);

HRMS (ESI^+) found *m/z* 366.0621 $[M+H]^+$, $C_{14}H_{15}F_3NO_5S^+$ requires *m/z* 366.0618.

1-Benzyl-1,2,3,6-tetrahydropyridin-4-yl trifluoromethanesulfonate (3r)[18]

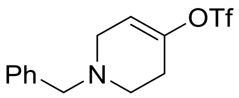

Prepared according to General Procedure A using 1-benzylpiperidin-4-one (1.13 g, 6.0 mmol). The crude product was purified by flash column chromatography (5–15% EtOAc in petrol) and filtered over basic alumina (10% EtOAc in petrol) to afford the corresponding alkenyl triflate **3r** as a yellow oil (1.81 g, 94%) with spectroscopic data in accordance with the literature;

^{1}H NMR (400 MHz, $CDCl_3$) δ 7.35–7.31 (m, 4H, 4 × Ar*H*), 7.31–7.26 (m, 1H, Ar*H*), 5.73 (td, *J* = 3.5, 1.7 Hz, 1H, OC=C*H*), 3.63 (s, 2H, ArCH_2), 3.13 (q, *J* = 3.1 Hz, 2H, C=CHCH_2), 2.73 (t, *J* = 5.7 Hz, 2H, CCH_2CH_2N), 2.49–2.42 (m, 2H, CCH_2CH_2N);

^{13}C {^{1}H} NMR (101 MHz, $CDCl_3$) δ 147.4, 137.8, 129.1, 128.6, 127.5, 118.7 (q, $^{1}J_{CF}$ = 320.7 Hz), 116.3, 61.4, 50.6, 49.2, 28.5;

^{19}F {^{1}H} NMR (377 MHz, $CDCl_3$) δ −73.9;

1-Tosyl-1,2,3,6-tetrahydropyridin-4-yl trifluoromethanesulfonate (3s)[19]

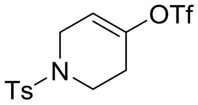

Prepared according to General Procedure A using 1-tosylpiperidin-4-one (0.76 g, 3.0 mmol). The crude product was purified by flash column chromatography (0–15% EtOAc in petrol) to afford the corresponding alkenyl triflate **3s** as a white solid (498 mg, 43%) with spectroscopic data in accordance with the literature;

mp 86–87 °C (EtOAc);

^{1}H NMR (400 MHz, $CDCl_3$) δ 7.67 (d, *J* = 8.1 Hz, 2H, 2 × Ar*H*), 7.34 (d, *J* = 8.1 Hz, 2H, 2 × Ar*H*), 5.76–5.70 (m, 1H, OC=C*H*), 3.78 (app. q, *J* = 3.0 Hz, 2H, CHCH_2), 3.35 (t, *J* = 5.7 Hz, 2H, NCH_2CH_2), 2.52–2.45 (m, 2H, NCH_2CH_2), 2.44 (s, 3H, CH_3);

^{13}C {^{1}H} NMR (101 MHz, $CDCl_3$) δ 146.5, 144.4, 133.4, 130.1, 127.6, 118.0 (q, $^{1}J_{CF}$ = 320.6 Hz), 114.6, 43.5, 42.8, 28.0, 21.7;

^{19}F {^{1}H} NMR (377 MHz, $CDCl_3$) δ −73.7;

LRMS (ESI^{+}) *m*/*z* 386.0 ([M+H]$^{+}$), 408.0 ([M+Na]$^{+}$).

3,4-Dihydronaphthalen-2-yl trifluoromethanesulfonate (3u)[12]

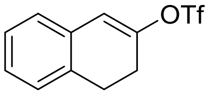

Prepared according to General Procedure A using 2-tetralone (0.40 mL, 3.0 mmol). The crude product was purified by flash column chromatography (0–0.25% Et_2O in petrol) to afford the corresponding alkenyl triflate **3u** as a colourless oil (670 mg, 80%) with spectroscopic data in accordance with the literature;

^{1}H NMR (400 MHz, $CDCl_3$) δ 7.24–7.21 (m, 2H, 2 × Ar*H*), 7.18–7.15 (m, 1H, Ar*H*), 7.12–7.08 (m, 1H, Ar*H*), 6.50 (s, 1H, OC=C*H*), 3.08 (t, *J* = 8.4 Hz, 2H, ArCH_2), 2.72 (t, *J* = 8.4 Hz, 2H, OCCH_2);

^{13}C {^{1}H} NMR (101 MHz, $CDCl_3$) δ 150.1, 133.1, 131.2, 128.6, 127.7, 127.5, 127.2, 118.8 (q, $^1J_{CF}$ = 320.4 Hz), 118.7, 28.7, 26.7;

^{19}F {^{1}H} NMR (377 MHz, $CDCl_3$) δ −73.6;

3,4-Dihydronaphthalen-1-yl trifluoromethanesulfonate (3v)[20]

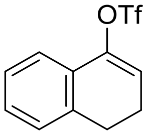

Prepared according to General Procedure A using 1-tetralone (1.46 g, 10.0 mmol) and $PhNTf_2$ (3.4 g, 9.5 mmol). The crude product was purified by vacuum distillation (***bp*** 100 °C, 5 mbar) using a Kugelrohr apparatus to afford the corresponding alkenyl triflate **3v** as a light yellow oil (2.06 g, 74%) with spectroscopic data in accordance with the literature;

^{1}H NMR (400 MHz, $CDCl_3$) δ 7.38–7.16 (m, 4H, 4 × Ar*H*), 6.03 (t, *J* = 4.8 Hz, 1H, OC=C*H*), 2.88 (t, *J* = 8.2 Hz, 2H, ArCH_2), 2.56–2.49 (m, 2H, OC=CHCH_2);

^{13}C {^{1}H} NMR (101 MHz, $CDCl_3$) δ 146.5, 136.3, 129.3, 128.8, 127.9, 127.1, 121.4, 118.7 (app. d, $^1J_{CF}$ = 320.3 Hz), 117.9, 27.0, 22.5;

^{19}F {^{1}H} NMR (377 MHz, $CDCl_3$) δ −73.7;

6-Methoxy-3,4-dihydronaphthalen-1-yl trifluoromethanesulfonate (3w)[21]

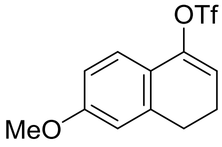

Prepared according to General Procedure A using 6-methoxy-1-tetralone (0.88 g, 5.0 mmol) and $PhNTf_2$ (1.78 g, 5.0 mmol). The crude product was purified by flash column chromatography (0–5% Et_2O in petrol) to afford the corresponding alkenyl triflate **3w** as a colourless oil (1.37 g, 89%) which solidified at -20 °C with spectroscopic data in accordance with the literature;

^{1}H NMR (400 MHz, $CDCl_3$) δ 7.28 (d, *J* = 8.4 Hz, 1H, Ar*H*), 6.77 (dd, *J* = 8.5, 2.6 Hz, 1H, Ar*H*), 6.74–6.73 (m, 1H, Ar*H*), 5.87 (t, *J* = 4.8 Hz, 1H, OC=C*H*), 3.82 (s, 3H, OCH_3), 2.84 (t, *J* = 8.1 Hz, 2H, ArCH_2), 2.51–2.45 (m, 2H, OC=CHCH_2);

^{13}C {^{1}H} NMR (101 MHz, $CDCl_3$) δ 160.4, 146.5, 138.5, 122.9, 121.8, 118.8 (q, $^1J_{CF}$ = 320.2 Hz), 114.9, 114.4, 111.4, 55.4, 27.5, 22.4.;

^{19}F {^{1}H} NMR (377 MHz, $CDCl_3$) δ −73.7;

3,4-Dihydronaphthalene-1,6-diyl bis(trifluoromethanesulfonate) (3x)[22]

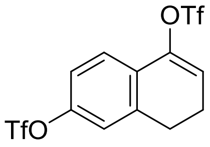

Prepared according to General Procedure A using 6-hydroxy-1-tetralone (486 mg, 3.0 mmol) and $PhNTf_2$ (2.07 g, 5.8 mmol). The crude product was purified by flash column chromatography (0–5% EtOAc in petrol) to afford the corresponding alkenyl triflate **3x** as a yellow oil (1.04 g, 84%) with spectroscopic data in accordance with the literature;

^{1}H NMR (400 MHz, $CDCl_3$) δ 7.41 (d, *J* = 8.5 Hz, 1H, Ar*H*), 7.17 (dd, *J* = 8.6, 2.4 Hz, 1H, Ar*H*), 7.12 (s, 1H, Ar*H*), 6.12 (t, *J* = 4.8 Hz, 1H, OC=C*H*), 2.92 (t, *J* = 8.2 Hz, 2H, ArCH_2), 2.57 (td, *J* = 8.2, 4.9 Hz, 2H, OC=CHCH_2);

^{13}C {^{1}H} NMR (101 MHz, $CDCl_3$) δ 149.5, 145.0, 139.1, 129.1, 123.2, 121.0, 119.9, 119.7, 118.8 (q, $^1J_{CF}$ = 320.7 Hz), 118.7 (q, $^1J_{CF}$ = 320.1 Hz), 26.9, 22.1;

^{19}F {^{1}H} NMR (377 MHz, $CDCl_3$) δ −72.8 (s, 3F), −73.6 (s, 3F).

3.4 Palladium-Catalysed Synthesis of Alkenylsulfonyl Fluorides

General Procedure B: Synthesis of alkenylsulfonyl fluorides from alkenyl triflates, DABSO and NFSI as exemplified by the preparation of cyclohept-1-ene-1-sulfonyl fluoride (5a)

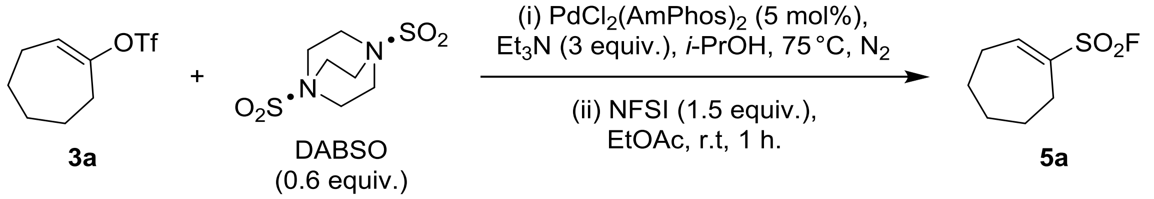

A reaction tube was charged with 1-cyclohept-1-enyl triflate (**3a**) (73.2 mg, 0.30 mmol, 1.0 equiv.), DABSO (43.2 mg, 0.18 mmol, 0.60 equiv.) and $PdCl_2(AmPhos)_2$ (10.6 mg, 0.015 mmol, 0.050 equiv.), sealed with a rubber septum, and evacuated and back-filled with N_2 for three times. Anhydrous degassed isopropanol (1.2 mL) and anhydrous triethylamine (0.13 mL, 0.90 mmol, 3.0 equiv.) were added subsequently, and the reaction mixture stirred under positive pressure of N_2 in a preheated aluminum heating block at 75 °C for 1 h. After cooling to ambient temperature, the reaction mixture was concentrated *in vacuo*, followed by the addition of EtOAc (1.2 mL), then NFSI (142 mg, 0.45 mmol, 1.5 equiv.). The reaction mixture was stirred at room temperature for 1 h until completion. The reaction mixture was then diluted with EtOAc, filtered through a plug of silica and concentrated *in vacuo* to yield the crude product which was then purified by flash column chromatography (5–20% CH_2Cl_2 in petrol), affording the *alkenylsulfonyl fluoride* **5a** as a colourless oil (37.5 mg, 70%);

R_f 0.41 (20% CH_2Cl_2 in hexane) [$KMnO_4$];

^{1}H NMR (400 MHz, $CDCl_3$) δ 7.32 (t, J = 6.5 Hz, 1H, SC=C*H*), 2.69–2.61 (m, 2H, CHCH_2), 2.47–2.38 (m, 2H, CCH_2), 1.88–1.78 (m, 2H, Alk-CH_2), 1.73–1.59 (m, 4H, 2 × Alk-CH_2);

^{13}C {^{1}H} NMR (101 MHz, $CDCl_3$) δ 148.6 (d, $^3J_{CF}$ = 2.6 Hz), 138.4 (d, $^2J_{CF}$ = 20.8 Hz), 30.9, 28.8, 27.9, 25.8, 25.0;

^{19}F {^{1}H} NMR (377 MHz, $CDCl_3$) δ 49.7;

IR ν_{max} (neat)/cm^{-1} 2930, 2858, 1449, 1394, 1227, 1194;

HRMS (APCI$^+$) found m/z 179.0531 $[M+H]^+$, $C_7H_{12}FO_2S^+$ requires m/z 179.0537.

Note: As a general procedure, the first step (sulfination) was monitored by TLC and HPLC for the consumption of alkenyl triflates and formation of alkenylsulfinates. The reaction was cooled to ambient temperature followed by solvent switch as soon as full consumption of alkenyl triflates was observed. The corresponding reaction time ranged from 0.5 to 10 h depending on the substrates, which are specified below.

1,2,3,6-Tetrahydro-[1,1'-biphenyl]-4-sulfonyl fluoride (5b)

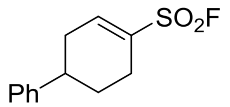

Prepared according to General Procedure B using alkenyl triflate **3b** (92 mg, 0.30 mmol), with the first step heated for 5.5 h. The crude product was purified by flash column chromatography (0–10% Et_2O in petrol) to afford the *alkenylsulfonyl fluoride* **5b** as a white crystalline solid (51.0 mg, 71%);

mp 115–116 °C (CH_2Cl_2);

R*f* 0.37 (15% Et_2O in hexane) [UV/$KMnO_4$];

^{1}H NMR (400 MHz, $CDCl_3$) δ 7.36 (t, *J* = 8.1, 6.7 Hz, 2H, 2 × Ar*H*), 7.30–7.18 (m, 4H, 3 × Ar*H* + SC=C*H*), 2.95–2.84 (m, 1H, ArC*H*), 2.76–2.53 (m, 3H, SCCH_2 + C=CHC$H_a$$H_b$), 2.52–2.39 (m, 1H, C=CHC$H_a$$H_b$), 2.23–2.14 (m, 1H, SC$CH_2CH_a$$H_b$), 1.98–1.83 (m, 1H, SC$CH_2CH_a$$H_b$);

^{13}C {^{1}H} NMR (101 MHz, $CDCl_3$) δ 144.2, 143.9 (d, $^3J_{CF}$ = 2.1 Hz), 133.8, 128.9, 127.1, 126.8, 38.4, 33.5, 28.9, 24.2;

^{19}F {^{1}H} NMR (377 MHz, $CDCl_3$) δ 50.7;

IR ν_{max} (neat)/cm^{-1} 2926, 1646, 1494, 1396, 1220, 1204;

HRMS (APCI$^+$) found *m/z* 241.0688 [M+H]$^+$, $C_{12}H_{14}FO_2S^+$ requires *m/z* 241.0693.

4-(*tert*-Butyl)cyclohex-1-ene-1-sulfonyl fluoride (5c)

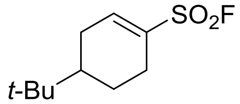

Prepared according to General Procedure B using alkenyl triflate **3c** (86 mg, 0.30 mmol), with the first step heated to 80 °C for 5 h. The crude product was purified by flash column chromatography (2% Et_2O in petrol) to afford the *alkenylsulfonyl fluoride* **5c** as a white solid (46.1 mg, 70%);

mp 48–50 °C (hexane);

R*f* 0.40 (5% Et_2O in hexane) [$KMnO_4$];

^{1}H NMR (400 MHz, $CDCl_3$) δ 7.19–7.11 (m, 1H, SC=C*H*), 2.69–2.58 (m, 1H, $C(CH_3)_3$C*H*), 2.49–2.32 (m, 2H, Alk-CH_2), 2.14–1.98 (m, 2H, Alk-CH_2), 1.42–1.21 (m, 2H, Alk-CH_2), 0.91 (s, 9H, C(CH_3)$_3$);

^{13}C {^{1}H} NMR (101 MHz, $CDCl_3$) δ 145.0, 133.7 (d, $^2J_{CF}$ = 22.6 Hz), 42.6, 32.3, 27.7, 27.1, 25.0, 23.3;

^{19}F {^{1}H} NMR (377 MHz, $CDCl_3$) δ 50.6;

IR ν_{max} (neat)/cm^{-1} 2968, 1649, 1391, 1369, 1201, 1055;

HRMS (ESI/APCI) not found.

4,4-Difluorocyclohex-1-ene-1-sulfonyl fluoride (5d)

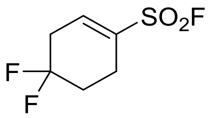

Prepared according to General Procedure B using alkenyl triflate **3d** (80 mg, 0.30 mmol), with the first step heated for 4 h. The crude product was purified by flash column chromatography (15–30% CH_2Cl_2 in petrol) to afford the *alkenylsulfonyl fluoride* **5d** as a light brown oil (40.2 mg, 67%);

R*f* 0.31 (33% CH_2Cl_2 in hexane) [$KMnO_4$];

^{1}H NMR (400 MHz, $CDCl_3$) δ 7.03–6.96 (m, 1H, SC=C*H*), 2.86 (tt, *J* = 13.4, 3.1 Hz, 2H, C=CHCH_2), 2.78 (t, *J* = 6.0 Hz, 2H, $CF_2CH_2CH_2$), 2.23 (tt, *J* = 13.3, 6.6 Hz, 2H, CF_2CH_2);

^{13}C {^{1}H} NMR (101 MHz, $CDCl_3$) δ 138.7 (td, $^3J_{CF}$ = 5.7, 2.5 Hz), 133.5 (d, $^2J_{CF}$ = 24.9 Hz), 120.4 (t, $^1J_{CF}$ = 241.7 Hz), 35.1 (t, $^2J_{CF}$ = 29.2 Hz), 29.6 (t, $^2J_{CF}$ = 25.4 Hz), 22.3 (t, $^3J_{CF}$ = 5.6 Hz);

^{19}F NMR (377 MHz, $CDCl_3$) δ 51.8 (s, 1F, SO_2F), −97.2 (quint d, *J* = 13.3, 2.6 Hz, 2F, CF_2);

IR ν_{max} (neat)/cm^{-1} 2953, 1654, 1403, 1378, 1265, 1205, 1150, 1121, 1086, 1059;

HRMS (APCI$^+$) found *m*/*z* 201.0189 $[M+H]^+$, $C_6H_8F_3O_2S^+$ requires *m*/*z* 201.0192.

Ethyl 4-(fluorosulfonyl)cyclohex-3-ene-1-carboxylate (5e)

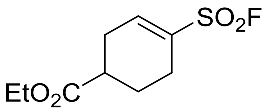

Prepared according to General Procedure B using alkenyl triflate **3e** (91 mg, 0.30 mmol), with the first step heated for 4.5 h. The crude product was purified by flash column chromatography (5–10% EtOAc in petrol) to afford the *alkenylsulfonyl fluoride* **5e** as a colourless oil (57.0 mg, 80%);

R*f* 0.27 (15% EtOAc in hexane) [$KMnO_4$];

^{1}H NMR (400 MHz, $CDCl_3$) δ 7.14 (t, *J* = 3.6 Hz, 1H, SC=C*H*), 4.17 (q, *J* = 7.1 Hz, 2H, OCH_2), 2.69–2.55 (m, 4H, C(=O)C*H* + C=CHCH_2 + SCCH_aH_b), 2.54–2.43 (m, 1H, SCCH_aH_b), 2.24–2.16 (m, 1H, SC$CH_2CH_cH_d$), 1.95–1.82 (m, 1H, SC$CH_2CH_cH_d$), 1.27 (t, *J* = 7.1 Hz, 3H, CH_3);

^{13}C {^{1}H} NMR (101 MHz, $CDCl_3$) δ 173.7, 142.5 (d, $^3J_{CF}$ = 3.0 Hz), 133.5 (d, $^2J_{CF}$ = 23.8 Hz), 61.2, 37.4, 27.8, 24.3, 22.8, 14.3;

^{19}F {^{1}H} NMR (377 MHz, $CDCl_3$) δ 50.8;

IR ν_{max} (neat)/cm^{-1} 2984, 1728, 1398, 1252, 1202, 1180, 1033;

LRMS (ESI$^+$) *m*/*z* 237.0 ($[M+H]^+$), 259.0 ($[M+Na]^+$);

HRMS (APCI$^+$) found *m*/*z* 236.0516 $[M]^+$, $C_9H_{13}FO_4S^+$ requires *m*/*z* 236.0513.

4-Methoxycyclohex-1-ene-1-sulfonyl fluoride (5f)

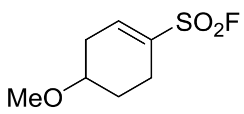

Prepared according to General Procedure B using alkenyl triflate **3f** (78 mg, 0.30 mmol), with the first step heated for 4 h. The crude product was purified by flash column chromatography (1:4:15 EtOAc/CH_2Cl_2/hexane mixture) to afford the *alkenylsulfonyl fluoride* **5f** as a colourless oil (33.3 mg, 57%);

R*f* 0.36 (1:4:8 EtOAc/CH_2Cl_2/hexane) [$KMnO_4$];

^{1}H NMR (400 MHz, $CDCl_3$) δ 7.03 (app. s, 1H, SC=C*H*), 3.66–3.51 (m, 1H, OC*H*), 3.37 (s, 3H, OC*H*$_3$), 2.67–2.53 (m, 2H, Alkyl-C*H*$_2$), 2.53–2.33 (m, 2H, Alkyl-C*H*$_2$), 2.05–1.84 (m, 2H, Alkyl-C*H*$_2$);

^{13}C {^{1}H} NMR (101 MHz, $CDCl_3$) δ 141.5 (d, $^3J_{CF}$ = 2.4 Hz), 133.6 (d, $^2J_{CF}$ = 23.8 Hz), 72.4, 56.2, 31.5, 25.7, 20.8;

^{19}F {^{1}H} NMR (377 MHz, $CDCl_3$) δ 50.8;

IR ν_{max} (neat)/cm^{-1} 2934, 2829, 1397, 1202, 1100, 1055, 769;

LRMS (ESI$^+$) *m/z* 217.0 ([M+Na]$^+$);

HRMS (APCI$^+$) found *m/z* 194.0412 [M]$^+$, $C_7H_{11}FO_3S^+$ requires *m/z* 194.0407.

***tert*-Butyl (4-(fluorosulfonyl)cyclohex-3-en-1-yl)carbamate (5g)**

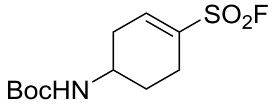

Prepared according to General Procedure B using alkenyl triflate **3g** (104 mg, 0.30 mmol), with the first step heated for 4 h. The crude product was purified by flash column chromatography (1:4:5 EtOAc/CH_2Cl_2/petrol mixture) to afford the *alkenylsulfonyl fluoride* **5g** as a white solid (53.5 mg, 64%);

mp 161–163 °C (CH_2Cl_2);

R*f* 0.31 (1:4:5 EtOAc/CH_2Cl_2/hexane) [$KMnO_4$];

^{1}H NMR (400 MHz, $CDCl_3$) δ 7.09–7.04 (m, 1H, SC=C*H*), 4.51 (br. s, 1H, N*H*), 3.85 (br. s, 1H, NC*H*), 2.76 (d, *J* = 19.2 Hz, 1H, SC=CHCH_aH$_b$), 2.67–2.50 (m, 2H, SCC*H*$_2$), 2.28–2.15 (m, 1H, SC=CHCH$_a$$H_b$), 2.12–2.01 (m, 1H, SCCH$_2$CH_cH$_d$), 1.81–1.67 (m, 1H, SCCH$_2$CH$_c$$H_d$), 1.45 (s, 9H, C(C*H*$_3$)$_3$);

^{13}C {^{1}H} NMR (101 MHz, $CDCl_3$) δ 155.2, 141.9, 133.6, 80.1, 44.2, 32.5, 28.5, 27.5, 22.3;

^{19}F {^{1}H} NMR (377 MHz, $CDCl_3$) δ 50.9;

IR ν_{max} (neat)/cm^{-1} 3359, 2981, 2938, 1676, 1529, 1398, 1370, 1316, 1301, 1178;

HRMS (APCI$^-$) found *m/z* 278.0870 [M–H]$^-$, $C_{11}H_{17}NO_4FS^-$ requires *m/z* 278.0868.

1,4-Dioxaspiro[4.5]dec-7-ene-8-sulfonyl fluoride (5h)

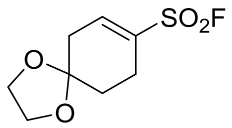

Prepared according to General Procedure B using alkenyl triflate **3h** (86 mg, 0.30 mmol), with the first step heated for 9 h. The crude product was purified by flash column chromatography (30–50% CH_2Cl_2 in petrol, then 10–30% Et_2O in petrol) to afford the *alkenylsulfonyl fluoride* **5h** as a white solid (49.5 mg, 74%);

Gram-scale synthesis:

An oven-dried two-neck round-bottom flask was charged with alkenyl triflate **3h** (1.01 g, 3.50 mmol, 1.0 equiv.), DABSO (504 mg, 2.10 mmol, 0.60 equiv.) and $PdCl_2(AmPhos)_2$ (124 mg, 0.175 mmol, 0.050 equiv.), evacuated and filled with N_2 for three times. Anhydrous degassed isopropanol (12 mL) and triethylamine (1.45 mL, 10.5 mmol, 3.0 equiv.) were added subsequently. The reaction mixture was heated under reflux under a N_2 atmosphere for 22 h or until completion. After cooling to ambient temperature, the reaction mixture was concentrated *in vacuo*, followed by the addition of EtOAc (14 mL), then NFSI (1.32 g, 4.20 mmol, 1.5 equiv.) was added in small portions. The reaction mixture was stirred at room temperature for 1 h. The reaction mixture was then diluted with Et_2O, filtered through a plug of silica and concentrated *in vacuo* to yield the crude product which was then purified by flash column chromatography as stated above to afford **5h** (0.57 g, 73%);

mp 62–64 °C (Et_2O);

$\mathbf{R_f}$ 0.07 (50% CH_2Cl_2 in hexane), 0.39 (50% Et_2O in hexane) [$KMnO_4$];

^{1}H NMR (400 MHz, $CDCl_3$) δ 7.02 (t, J = 3.2 Hz, 1H, SC=C*H*), 4.01 (s, 4H, 2 × OCH_2), 2.70 (t, J = 6.3 Hz, 2H, CH=CCH_2), 2.56 (t, J = 3.1 Hz, 2H, C=CHCH_2), 1.91 (t, J = 6.5 Hz, 2H, CH=CCH$_2$CH_2);

^{13}C {^{1}H} NMR (101 MHz, $CDCl_3$) δ 141.7 (d, $^3J_{CF}$ = 2.4 Hz), 133.5 (d, $^2J_{CF}$ = 23.7 Hz), 105.9, 64.9, 36.4, 30.6, 23.2;

^{19}F {^{1}H} NMR (377 MHz, $CDCl_3$) δ 51.5;

IR ν_{max} (neat)/cm^{-1} 2894, 1650, 1400, 1372, 1201, 1121;

HRMS (APCI$^+$) found *m/z* 223.0431 [M+H]$^+$, $C_8H_{12}FO_4S^+$ requires *m/z* 223.0435.

3-(1-Methyl-1*H*-indol-3-yl)cyclohex-1-ene-1-sulfonyl fluoride (5i)

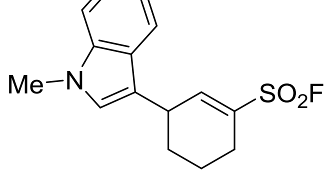

Prepared according to General Procedure B using alkenyl triflate **3i** (108 mg, 0.30 mmol), with the first step heated for 10 h. The crude product was purified by flash column chromatography (20–35% CH_2Cl_2 in petrol) to afford the *alkenylsulfonyl fluoride* **5i** as a light yellow oil (45.4 mg, 52%);

Alternatively, the first step was heated to 95 °C under microwave irradiation for 3 h, followed by fluorination and purification as described above to afford **5i** (46.0 mg, 52%).

R*f* 0.27 (40% CH_2Cl_2 in hexane) [$KMnO_4$/UV];

^{1}H NMR (400 MHz, $CDCl_3$) δ 7.55 (d, *J* = 8.0 Hz, 1H, Ar*H*), 7.32 (d, *J* = 8.3 Hz, 1H, Ar*H*), 7.28–7.23 (m, 2H, Ar*H* + SC=C*H*), 7.13 (t, *J* = 8.0 Hz, 1H, Ar*H*), 6.77 (s, 1H, NC*H*), 4.03–3.95 (m, 1H, SC=CHC*H*), 3.76 (s, 3H, NCH_3), 2.58–2.53 (m, 2H, SCCH_2), 2.15–2.03 (m, 1H, CHCH_aH$_b$), 1.97–1.77 (m, 3H, CHCH$_a$$H_b$ + SCCH$_2$CH_2);

^{13}C {^{1}H} NMR (101 MHz, $CDCl_3$) δ 146.3, 137.3, 134.0 (d, $^2J_{CF}$ = 21.8 Hz)., 126.7, 126.5, 122.3, 119.4, 118.7, 114.8, 109.7, 33.5, 32.9, 28.4, 23.8, 20.1;

^{19}F {^{1}H} NMR (377 MHz, $CDCl_3$) δ 50.8;

IR ν_{max} (neat)/cm^{-1} 3051, 2941, 2862, 1474, 1398, 1329, 1204, 738;

HRMS (ESI$^+$) found *m/z* 294.0960 [M+H]$^+$, $C_{15}H_{17}FNO_2S^+$ requires *m/z* 294.0959.

3-(5-Methylfuran-2-yl)cyclohex-1-ene-1-sulfonyl fluoride (5k)

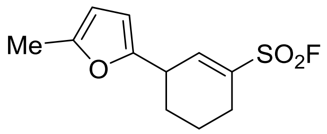

Prepared according to General Procedure B using alkenyl triflate **3k** (93 mg, 0.30 mmol), with the first step heated for 9 h. The crude product was purified by flash column chromatography (10–25% CH_2Cl_2 in petrol) to afford the *alkenylsulfonyl fluoride* **5k** as a colourless oil (50.3 mg, 69%);

R*f* 0.36 (25% CH_2Cl_2 in hexane) [$KMnO_4$/UV];

^{1}H NMR (400 MHz, $CDCl_3$) δ 7.21–7.14 (m, 1H, SC=C*H*), 5.93 (d, *J* = 3.2 Hz, 1H, Ar*H*), 5.92–5.85 (m, 1H, Ar*H*), 3.75–3.65 (m, 1H, C=CHC*H*), 2.58–2.44 (m, 2H, SCCH_2), 2.27 (s, 3H, CH_3), 2.08–1.93 (m, 2H, CHCH_2), 1.87–1.75 (m, 2H, SCCH$_2$CH_2);

^{13}C {^{1}H} NMR (101 MHz, $CDCl_3$) δ 152.0, 151.9, 143.3, 134.9 (d, $^2J_{CF}$ = 22.5 Hz), 106.9, 106.2, 35.9, 26.3, 23.6, 20.1, 13.7.

^{19}F {^{1}H} NMR (377 MHz, $CDCl_3$) δ 50.6;

IR ν_{max} (neat)/cm^{-1} 2951, 2868, 1402, 1206, 1020, 944, 778;

HRMS (ESI$^-$) found *m/z* 243.0494 [M−H]$^-$, $C_{11}H_{12}O_3FS^-$ requires *m/z* 243.0497.

6-Methylcyclohex-1-ene-1-sulfonyl fluoride (5l)

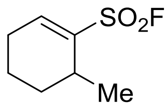

Prepared according to General Procedure B using alkenyl triflate **3l** (73 mg, 0.30 mmol), with the first step heated to 95 °C under microwave irradiation for 3 h. The crude product was purified by flash column chromatography (10–30% CH_2Cl_2 in petrol) to afford the *alkenylsulfonyl fluoride* **5l** as a colourless oil (22.8 mg, 43%);

R*f* 0.21 (20% CH_2Cl_2 in hexane) [$KMnO_4$];

^{1}H NMR (400 MHz, $CDCl_3$) δ 7.19 (t, *J* = 3.9 Hz, 1H, SC=C*H*), 2.85–2.77 (m, 1H, CH_3CH), 2.43–2.24 (m, 2H, C=CHCH_2), 1.86–1.61 (m, 4H, C=CHCH$_2$CH_2 + CH_3CHCH_2), 1.27 (dd, *J* = 7.0, 1.7 Hz, 3H, CH_3);

^{13}C {^{1}H} NMR (101 MHz, $CDCl_3$) δ 145.6 (d, $^3J_{CF}$ = 2.3 Hz), 138.7 (d, $^2J_{CF}$ = 19.0 Hz), 30.2, 28.8, 26.3, 19.7, 17.0;

^{19}F {^{1}H} NMR (377 MHz, $CDCl_3$) δ 58.2;

IR ν_{max} (neat)/cm^{-1} 2944, 2880, 1399, 1202, 761, 729, 625;

HRMS (ESI/APCI) not found.

3,6-Dihydro-2*H*-thiopyran-4-sulfonyl fluoride 1,1-dioxide (5m)

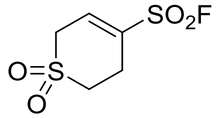

Prepared according to General Procedure B using alkenyl triflate **3m** (84 mg, 0.30 mmol), with the first step heated for 1 h. The crude product was purified by flash column chromatography (20–40% EtOAc in petrol) to afford the *alkenylsulfonyl fluoride* **5m** as a creamy yellow solid (31.5 mg, 49%);

mp 113–115 °C (EtOAc);

R*f* 0.34 (40% EtOAc in hexane) [$KMnO_4$];

^{1}H NMR (400 MHz, $CDCl_3$) δ 7.06 (t, *J* = 4.3 Hz, 1H, SC=C*H*), 3.96–3.88 (m, 2H, CHCH_2), 3.32–3.20 (m, 4H, 2 × CH_2);

^{13}C {^{1}H} NMR (101 MHz, $CDCl_3$) δ 135.2, 134.2 (d, $^2J_{CF}$ = 27.0 Hz), 50.0, 46.5, 24.8;

^{19}F {^{1}H} NMR (377 MHz, $CDCl_3$) δ 52.8;

IR ν_{max} (neat)/cm^{-1} 2964, 2922, 1406, 1326, 1292, 1206, 1170, 1126, 1059;

HRMS (ESI$^-$) found *m*/*z* 212.9697 [M−H]$^-$, $C_5H_6O_4FS_2^-$ requires *m*/*z* 212.9697.

3,6-Dihydro-2*H*-pyran-4-sulfonyl fluoride (5n)

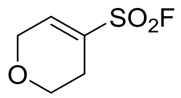

Prepared according to General Procedure B using alkenyl triflate **3n** (70 mg, 0.30 mmol), with the first step heated for 3 h. The crude product was purified by flash column chromatography (20–50% CH_2Cl_2 in pentane) to afford the *alkenylsulfonyl fluoride* **5n** as a light yellow oil (31.0 mg, 62%);

R*f* 0.36 (50% CH_2Cl_2 in pentrol) [$KMnO_4$];

^{1}H NMR (400 MHz, $CDCl_3$) δ 7.18–7.13 (m, 1H, SC=C*H*), 4.39 (app. quint, *J* = 2.8 Hz, 2H, CHCH_2), 3.90 (t, *J* = 5.4 Hz, 2H, OCH_2CH_2), 2.61–2.55 (m, 2H, OCH_2CH_2);

^{13}C {^{1}H} NMR (101 MHz, $CDCl_3$) δ 142.0 (d, $^3J_{CF}$ = 2.5 Hz), 131.6 (d, $^2J_{CF}$ = 25.7 Hz), 64.9, 63.3, 23.9;

^{19}F {^{1}H} NMR (377 MHz, $CDCl_3$) δ 50.9;

IR ν_{max} (neat)/cm^{-1} 2872, 1653, 1434, 1399, 1279, 1237, 1200, 1129;

HRMS (APCI$^+$) found *m/z* 167.0170 [M+H]$^+$, $C_5H_8FO_3S^+$ requires *m/z* 167.0173.

***tert*-Butyl 5-(fluorosulfonyl)-3,6-dihydropyridine-1(2*H*)-carboxylate (5o)**

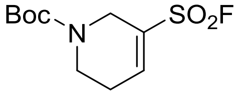

Prepared according to General Procedure B using alkenyl triflate **3o** (99 mg, 0.30 mmol), with the first step heated for 1.5 h. The crude product was purified by flash column chromatography (50% CH_2Cl_2 in petrol, then 10–30% EtOAc in petrol) to afford the *alkenylsulfonyl fluoride* **5o** as a colourless oil which solidified at -20 °C (55.0 mg, 69%);

mp 43–45 °C (EtOAc);

R*f* 0.36 (25% EtOAc in hexane) [$KMnO_4$/UV (weak)];

^{1}H NMR (400 MHz, $CDCl_3$) δ 7.25 (app. s, 1H, SC=C*H*), 4.28 (s, 2H, CCH_2), 3.55 (t, *J* = 5.7 Hz, 2H, NCH_2CH$_2$), 2.52–2.40 (m, 2H, CHCH_2), 1.48 (s, 9H, C(CH_3)$_3$);

^{13}C {^{1}H} NMR (101 MHz, $CDCl_3$) δ 154.2, 143.1, 131.6, 81.3, 41.8, 38.4, 28.4, 25.8;

^{19}F {^{1}H} NMR (377 MHz, $CDCl_3$) δ 54.2;

IR ν_{max} (neat)/cm^{-1} 2978, 2935, 1740, 1699, 1616, 1407, 1207, 1157;

LRMS (ESI$^+$) *m/z* 288.0 ([M+Na]$^+$);

HRMS (ESI$^+$) found *m/z* 288.0676 [M+Na]$^+$, $C_{10}H_{16}NO_4FSNa^+$ requires *m/z* 288.0676.

tert-Butyl 4-(fluorosulfonyl)-3,6-dihydropyridine-1(2_H_)-carboxylate (5p)

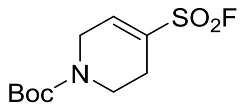

Prepared according to General Procedure B using alkenyl triflate **3p** (99 mg, 0.30 mmol), with the first step heated for 2 h. The crude product was purified by flash column chromatography (5–10% EtOAc in petrol) to afford the *alkenylsulfonyl fluoride* **5p** as a white solid (65.2 mg, 82%);

mp 56-58 °C (CH_2Cl_2);

$\mathbf{R}_{\boldsymbol{f}}$ 0.36 (20% EtOAc in hexane) [$KMnO_4$/UV];

^{1}H NMR (500 MHz, $CDCl_3$) δ 7.08 (br. s, 1H, SC=C*H*), 4.22 (app. t, *J* = 3.0 Hz, 2H, CHCH_2), 3.64 (t, *J* = 5.5 Hz, 2H, NCH_2CH$_2$), 2.61–2.54 (m, 2H, CCH_2), 1.48 (s, 9H, C(CH_3)$_3$);

^{13}C {^{1}H} NMR (126 MHz, $CDCl_3$) δ 154.2, {140.6, 140.0}, 132.7 (br.), 81.2, {43.7, 43.3}, {40.1, 38.9}, 28.5, 24.2 (recorded as a pair of rotamers);

^{19}F {^{1}H} NMR (377 MHz, $CDCl_3$) δ 51.2;

IR ν_{max} (neat)/cm^{-1} 2978, 1697, 1403, 1367, 1241, 1203, 1165, 1118;

HRMS (APCI$^-$) found *m*/*z* 264.0710 [M−H]$^-$, $C_{10}H_{15}FNO_4S^-$ requires *m*/*z* 264.0711.

Benzyl 4-(fluorosulfonyl)-3,6-dihydropyridine-1(2_H_)-carboxylate (5q)

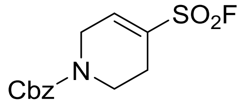

Prepared according to General Procedure B using alkenyl triflate **3q** (110 mg, 0.30 mmol), with the first step heated for 2 h. The crude product was purified by flash column chromatography (30–60% CH_2Cl_2 in petrol, followed by 4:2:1 petrol/CH_2Cl_2/Et_2O mixture) to afford the *alkenylsulfonyl fluoride* **5q** as a light yellow solid (58.0 mg, 65%);

mp 56–58 °C (CH_2Cl_2);

$\mathbf{R}_{\boldsymbol{f}}$ 0.13 (50% CH_2Cl_2 in hexane), 0.41 (4:2:1 hexane/CH_2Cl_2/Et_2O) [$KMnO_4$/ UV (weak)];

^{1}H NMR (400 MHz, $CDCl_3$) δ 7.41–7.32 (m, 5H, 5 × Ar*H*), 7.11 (br. s, 0.5H, SC=CH^{rot1}), 7.05 (br. s, 0.5H, SC=CH^{rot2}), 5.17 (s, 2H, ArCH_2), 4.30 (app. s, 2H, CHCH_2), 3.73 (t, *J* = 5.6 Hz, 2H, NCH_2CH$_2$), 2.60 (app. s, 2H, NCH$_2$CH_2) (recorded as a pair of rotamers);

^{13}C {^{1}H} NMR (101 MHz, $CDCl_3$) δ 154.9, {140.1, 139.4}, 136.1, 132.8, 128.8, 128.5, 128.3, 68.0, 43.6, {39.9, 39.5}, {24.2, 24.0}(recorded as a pair of rotamers);

^{19}F {^{1}H} NMR (377 MHz, $CDCl_3$) δ 51.41, 51.36 (recorded as a pair of rotamers);

IR ν_{max} (neat)/cm^{-1} 2925, 1700, 1655, 1427, 1401, 1234, 1200, 1112;

LRMS (ESI$^-$) *m*/*z* 298.0 ([M−H]$^-$);

HRMS (ESI$^-$) found *m*/*z* 298.0555 [M−H]$^-$, $C_{13}H_{13}FNO_4S^-$ requires *m*/*z* 298.0555.

1-Benzyl-1,2,3,6-tetrahydropyridine-4-sulfonyl fluoride (5r)

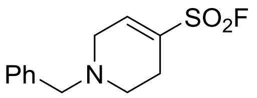

A reaction tube was charged with alkenyl triflate **3r** (96 mg, 0.30 mmol, 1.0 equiv.), DABSO (43 mg, 0.18 mmol, 0.60 equiv.) and $PdCl_2(AmPhos)_2$ (10.6 mg, 0.015 mmol, 0.050 equiv.), sealed with a rubber septum, evacuated and filled with N_2 for three times. Anhydrous degassed isopropanol (1.2 mL) and anhydrous triethylamine (0.13 mL, 0.90 mmol, 3.0 equiv.) were added subsequently. The septum was quickly replaced with a Teflon microwave cap, and the reaction was heated to 95 °C under microwave irradiation for 3 h. After cooling to ambient temperature, the reaction mixture was concentrated *in vacuo*, followed by the addition of ethyl acetate (1.2 mL), DIPEA (0.16 mL, 0.90 mmol, 3.0 equiv.) and then NFSI (142 mg, 0.45 mmol, 1.5 equiv.). The reaction mixture was stirred at room temperature for 1 h, and then diluted with EtOAc, filtered through a plug of silica and concentrated *in vacuo* to yield the crude product (53% NMR yield) which was then purified by flash column chromatography (10% EtOAc in petrol) to afford the *alkenylsulfonyl fluoride* **5r** as a yellow oil (35.5 mg, 46%);

R_f 0.18 (10% EtOAc in hexane) [$KMnO_4$/UV];

^{1}H NMR (400 MHz, $CDCl_3$) δ 7.38–7.27 (m, 5H, 5 × Ar*H*), 7.08–7.03 (m, 1H, SC=C*H*), 3.66 (s, 2H, PhC*H*$_2$N), 3.27 (app. quint, J = 3.0 Hz, 2H, NC*H*$_2$CH), 2.74 (t, J = 5.5 Hz, 2H, NC*H*$_2$CH$_2$), 2.62–2.56 (m, 2H, CH=CC*H*$_2$);

^{13}C {^{1}H} NMR (101 MHz, $CDCl_3$) δ 141.7, 137.2, 132.3 (d, $^2J_{CF}$ = 24.7 Hz), 129.1, 128.7, 127.7, 61.8, 52.2, 48.4, 24.8;

^{19}F {^{1}H} NMR (377 MHz, $CDCl_3$) δ 51.1;

IR ν_{max} (neat)/cm^{-1} 2919, 1404, 1207, 1159, 1029, 781, 736, 701, 652;

HRMS (ESI$^+$) found *m/z* 256.0802 [M+H]$^+$, $C_{12}H_{15}NO_2FS^+$ requires *m/z* 256.0802.

Note: The sulfination step can be performed at 80 °C for 3 h according to General Procedure B, with an NMR yield of 53%. However, the purified product was found to be contaminated with trace amount of unreacted starting material **3r**. Heating to 95 °C with microwave irradiation, on the other hand, ensured full consumption of **3r** without significantly increasing the reaction time, where prolonged reaction time would be detrimental to the yield of the alkenylsulfinate and hence the alkenylsulfonyl fluoride.

1-Tosyl-1,2,3,6-tetrahydropyridine-4-sulfonyl fluoride (5s)

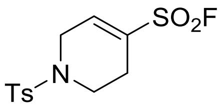

Prepared according to General Procedure B using alkenyl triflate **3s** (116 mg, 0.30 mmol), with the first step heated for 2 h. The crude product was purified by flash column chromatography (10–40% Et_2O in petrol) to afford the *alkenylsulfonyl fluoride* **5s** as a white solid (86.2 mg, 90%);

mp 176–178 °C (Et_2O);

$\mathbf{R}_f$ 0.18 (40% Et_2O in hexane)

^{1}H NMR (400 MHz, $CDCl_3$) δ 7.68 (d, J = 8.1 Hz, 2H, 2 × Ar*H*), 7.36 (d, J = 8.1 Hz, 2H, 2 × Ar*H*), 7.05–7.00 (m, 1H, SC=C*H*), 3.93 (app. quint, J = 2.9 Hz, 2H, CHCH_2), 3.35 (t, J = 5.6 Hz, 2H, NCH_2CH$_2$), 2.68–2.61 (m, 2H, NCH$_2$CH_2), 2.44 (s, 3H, CH_3);

^{13}C {^{1}H} NMR (101 MHz, $CDCl_3$) δ 144.7, 138.5 (d, $^3J_{CF}$ = 2.5 Hz), 132.8, 132.6 (d, $^2J_{CF}$ = 26.2 Hz), 130.2, 127.7, 44.9, 41.9, 24.2, 21.7;

^{19}F {^{1}H} NMR (377 MHz, $CDCl_3$) δ 51.7;

IR ν_{max} (neat)/cm^{-1} 3657, 2980, 2889, 1381, 1250, 1206, 1162, 1073;

LRMS (ESI$^+$) *m/z* 320.0 ([M+H]$^+$), 342.0 ([M+Na]$^+$);

HRMS (ESI$^+$) found *m/z* 320.0421 [M+H]$^+$, $C_{12}H_{15}FNO_4S_2^+$ requires *m/z* 320.0421.

General Procedure C: Synthesis of alkenylsulfonyl fluorides from tetralone-derived alkenyl triflates, DABSO and NFSI as exemplified by the preparation of 3,4-dihydronaphthalene-2-sulfonyl fluoride (5u)

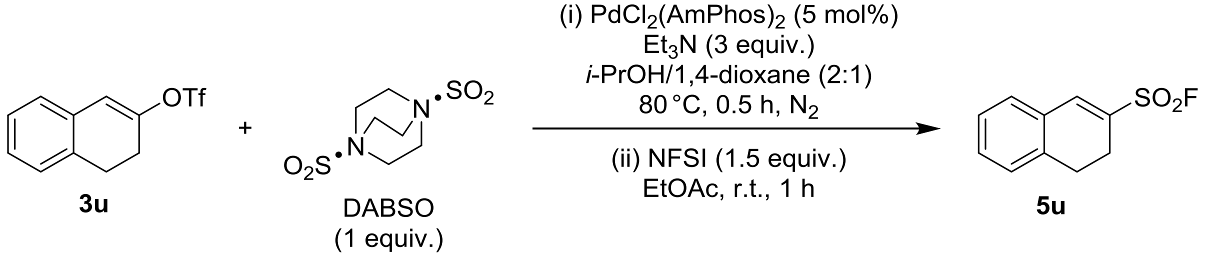

A reaction tube was charged with alkenyl triflate **3u** (83 mg, 0.30 mmol, 1.0 equiv.), DABSO (72 mg, 0.30 mmol, 1.0 equiv.) and $PdCl_2(AmPhos)_2$ (10.6 mg, 0.015 mmol, 0.050 equiv.), sealed with a rubber septum, and evacuated and back-filled with N_2 for three times. Anhydrous degassed isopropanol (0.8 mL), 1,4-dioxane (0.4 mL) and triethylamine (0.13 mL, 0.90 mmol, 3.0 equiv.) were added subsequently, and the reaction mixture was stirred under positive pressure of N_2 in a preheated aluminum heating block at 80 °C for 0.5 h. After cooling to ambient temperature, the reaction mixture was concentrated *in vacuo*, followed by addition of EtOAc (1.2 mL), then NFSI (142 mg, 0.45 mmol, 1.5 equiv.). The reaction mixture was stirred at room temperature for 1 h until completion. The reaction mixture was then diluted with EtOAc, filtered through a plug of silica and concentrated *in vacuo* to yield the crude product, which was then purified by flash column chromatography (10–20% CH_2Cl_2 in petrol), affording the *alkenylsulfonyl fluoride* **5u** as a colourless oil (35.0 mg, 55%);

$\mathbf{R}_f$ 0.30 (20% CH_2Cl_2 in hexane) [$KMnO_4$/UV];

^{1}**H NMR** (400 MHz, $CDCl_3$) δ 7.63 (s, 1H, SC=C*H*), 7.38 (dt, J = 7.4, 4.3 Hz, 1H, Ar*H*), 7.29 (d, J = 4.4 Hz, 2H, 2 × Ar*H*), 7.23 (dt, J = 7.4, 1.0 Hz, 1H, Ar*H*), 3.05 (t, J = 8.3 Hz, 2H, $SCCH_2CH_2$), 2.79 (t, J = 8.3 Hz, 2H, $SCCH_2CH_2$);

^{13}**C {^{1}H} NMR** (101 MHz, $CDCl_3$) δ 140.3 (d, $^3J_{CF}$ = 3.2 Hz), 136.2, 131.9, 130.4 (d, $^2J_{CF}$ = 24.7 Hz), 129.9, 129.8, 128.3, 127.6, 77.5, 77.2, 76.8, 27.3, 22.1;

^{19}**F {^{1}H} NMR** (377 MHz, $CDCl_3$) δ 54.2

IR ν_{max} (neat)/cm^{-1} 3065, 3023, 2953, 2917, 2849, 1629, 1397, 1193, 1044;

HRMS (EI$^+$) found m/z 212.0304 [M]$^+$, $C_{10}H_9FO_2S^+$ requires m/z 212.0302.

Note: Alternatively, when General Procedure B was followed with the first step (sulfination) heated for 0.5 h, **5u** was afforded in 43% yield (27.5 mg).

3,4-Dihydronaphthalene-1-sulfonyl fluoride (5v)

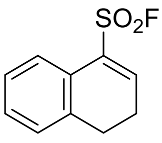

Prepared according to General Procedure C using alkenyl triflate **3v** (83 mg, 0.30 mmol), with the first step heated for 1 h. The crude product was purified by flash column chromatography (5–20% CH_2Cl_2 in petrol) to afford the *alkenylsulfonyl fluoride* **5v** as a colourless oil (40.1 mg, 63%);

R*f* 0.30 (20% CH_2Cl_2 in hexane) [$KMnO_4$/UV];

^{1}H NMR (400 MHz, $CDCl_3$) δ 7.72 (dt, *J* = 6.1, 3.0 Hz, 1H, Ar*H*), 7.45 (t, *J* = 4.9 Hz, 1H, SC=C*H*), 7.31 (dt, *J* = 7.9, 3.9 Hz, 2H, 2 × Ar*H*), 7.25–7.20 (m, 1H, Ar*H*), 2.89 (t, *J* = 8.1 Hz, 2H, SC=CHCH_2CH_2), 2.66–2.58 (m, 2H, SC=CHCH_2);

^{13}C {^{1}H} NMR (101 MHz, $CDCl_3$) δ 144.6 (d, $^3J_{CF}$ = 2.4 Hz), 135.7, 134.4 (d, $^2J_{CF}$ = 22.7 Hz), 129.7, 128.4, 127.5, 125.8, 124.9, 26.7, 23.8.

^{19}F {^{1}H} NMR (377 MHz, $CDCl_3$) δ 57.2;

IR ν_{max} (neat)/cm^{-1} 3072, 3028, 2951, 2894, 2840, 1400, 1221, 1201;

HRMS (EI^+) found *m/z* 212.0305 $[M]^+$, $C_{10}H_9FO_2S^+$ requires *m/z* 212.0302.

6-Methoxy-3,4-dihydronaphthalene-1-sulfonyl fluoride (5w)

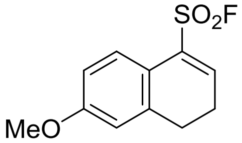

Prepared according to General Procedure C using alkenyl triflate **3w** (92 mg, 0.30 mmol), with the first step heated for 1.5 h. The crude product was purified by flash column chromatography (0–3% EtOAc in petrol) to afford the *alkenylsulfonyl fluoride* **5w** as a yellow oil (41.3 mg, 57%) which solidified at -20 °C;

mp 41–42 °C (hexane);

R*f* 0.25 (10% EtOAc in hexane) [$KMnO_4$/UV];

^{1}H NMR (400 MHz, $CDCl_3$) δ 7.65 (dd, *J* = 8.7, 2.5 Hz, 1H, Ar*H*), 7.29 (t, *J* = 4.9 Hz, 1H, SC=C*H*), 6.81 (dd, *J* = 8.7, 2.7 Hz, 1H, Ar*H*), 6.77 (d, *J* = 2.7 Hz, 1H, Ar*H*), 3.83 (s, 3H, OCH_3), 2.85 (t, *J* = 8.1 Hz, 2H, SC=CHCH_2CH_2), 2.62–2.54 (m, 2H, SC=CHCH_2);

^{13}C {^{1}H} NMR (101 MHz, $CDCl_3$) δ 160.4, 141.6 (d, $^3J_{CF}$ = 2.6 Hz), 137.8, 134.0 (d, $^2J_{CF}$ = 22.7 Hz), 126.4, 118.6, 114.6, 111.9, 55.5, 27.2, 23.7;

^{19}F {^{1}H} NMR (377 MHz, $CDCl_3$) δ 56.9;

IR ν_{max} (neat)/cm^{-1} 3070, 2946, 2840, 1607, 1566, 1500, 1399, 1256, 1205;

HRMS ($APCI^-$) found *m/z* 241.0337 $[M-H]^-$, $C_{11}H_{10}FO_3S^-$ requires *m/z* 241.0340.

5-(Fluorosulfonyl)-7,8-dihydronaphthalen-2-yl trifluoromethanesulfonate (5x)

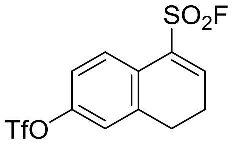

Prepared according to General Procedure C using alkenyl triflate **3x** (108 mg, 0.25 mmol), with the first step heated for 0.5 h. The crude product was purified by flash column chromatography (40:1:1 to 20:1:1 petrol/CH_2Cl_2/EtOAc) to afford the *alkenylsulfonyl fluoride* **5x** as a yellow oil (53.0 mg, 59%) which solidified at -20 °C;

mp 32–34 °C (hexane);

R*f* 0.24 (18:1:1 hexane/CH_2Cl_2/EtOAc) [$KMnO_4$/UV];

^{1}H NMR (400 MHz, $CDCl_3$) δ 7.82 (dd, *J* = 8.8, 2.1 Hz, 1H, Ar*H*), 7.54 (t, *J* = 4.8 Hz, 1H, SC=C*H*), 7.22 (dd, *J* = 8.7, 2.7 Hz, 1H, Ar*H*), 7.18 (d, *J* = 2.6 Hz, 1H, Ar*H*), 2.95 (t, *J* = 8.2 Hz, 2H, SC=CHCH$_2$CH_2), 2.68 (tdd, *J* = 7.9, 4.8, 2.5 Hz, 2H, SC=CHCH_2);

^{13}C {^{1}H} NMR (101 MHz, $CDCl_3$) δ 149.6, 146.0 (d, $^3J_{CF}$ = 2.3 Hz), 138.7, 133.4 (d, $^2J_{CF}$ = 24.6 Hz), 126.9, 126.1, 121.5, 120.3, 118.8 (app. d, $^1J_{CF}$ = 320.8 Hz), 26.7, 23.4;

^{19}F NMR (377 MHz, $CDCl_3$) δ 57.4 (s, 1F, SO$_2$*F*), -72.7 (s, 3F, SO$_2$CF_3);

IR ν_{max} (neat)/cm^{-1} 3079, 2956, 2922, 1602, 1579, 1461, 1406, 1250, 1203, 1138, 1114;

HRMS (ESI/CI) not found.

Prop-2-yn-1-yl 4-(fluorosulfonyl)-3,6-dihydropyridine-1(2*H*)-carboxylate

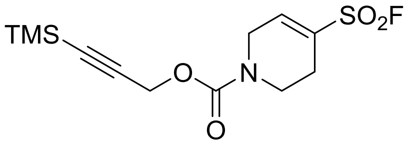

Prepared according to General Procedure B using the corresponding alkenyl triflate (116 mg, 0.30 mmol), with the first step heated for 6 h, affording the *alkenylsulfonyl fluoride* in 42% yield as determined by ^{19}F-NMR spectroscopy against $PhCF_3$ as internal standard. Alternatively, the first step was heated to 95 °C under microwave irradiation for 3 h, and crude product was purified by flash column chromatography (10:3:1 to 6:3:1 petrol/CH_2Cl_2/Et_2O) to afford the *alkenylsulfonyl fluoride* as a white solid (28.3 mg, 30%);

mp 70–71 °C (CH_2Cl_2);

R*f* 0.38 (6:3:1 hexane/CH_2Cl_2/Et_2O) [$KMnO_4$/UV];

^{1}H NMR (400 MHz, $CDCl_3$) δ 7.14 – 7.05 (m, 1H, SC=C*H*), 4.75 (s, 2H, OCH_2), 4.38 – 4.25 (m, 2H, CHCH_2), 3.73 (t, *J* = 5.6 Hz, 2H, NCH_2CH$_2$), 2.69 – 2.55 (m, 2H, NCH$_2$CH_2), 0.18 (s, 9H, Si(CH_3)$_3$);

^{13}C {^{1}H} NMR (101 MHz, $CDCl_3$) δ 154.2, {139.9, 139.4}, 99.2, 92.5, 54.5, 43.7, {40.0, 39.7}, 30.5, {24.2, 24.0}, -0.2 (recorded as a pair of rotamers);

^{19}F {^{1}H} NMR (377 MHz, $CDCl_3$) δ 51.40, 51.38 (recorded as a pair of rotamers);

IR ν_{max} (neat)/cm^{-1} 2960, 2187, 1713, 1435, 1406, 1234, 1204, 1111;

HRMS (ESI) not found.

3.5 Derivatisation of Alkenylsulfonyl Fluorides

3.5.1 Nucleophilic addition of p-methoxyphenol for the preparation of 4-methoxyphenyl 1,2,3,6-tetrahydro-[1,1'-biphenyl]-4-sulfonate (6)

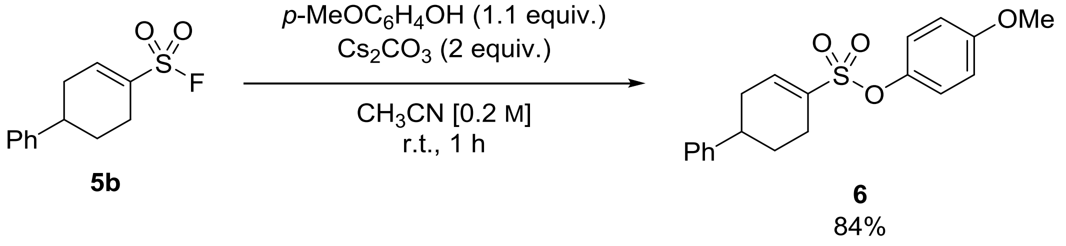

A sample vial was charged with alkenylsulfonyl fluoride **5b** (24.0 mg, 0.10 mmol, 1.0 equiv.), *p*-methoxyphenol (13.6 mg, 0.11 mmol, 1.1 equiv.), Cs_2CO_3 (65.2 mg, 0.20 mmol, 2.0 equiv.) and CH_3CN (0.5 mL). The reaction was stirred at ambient temperature for 1 h, which was then diluted with hexane and directly purified by flash column chromatography (10–20% EtOAc in petrol), affording the *alkenyl sulfonate ester* **6** as a white solid (28.8 mg, 84%);

mp 58–60 °C (EtOAc);

$\mathbf{R}_f$ 0.46 (25% EtOAc in hexane) [$KMnO_4$/UV];

^{1}H NMR (400 MHz, $CDCl_3$) δ 7.38–7.29 (m, 2H, 2 × Ar*H*), 7.27–7.22 (m, 1H, Ar*H*), 7.19 (d, *J* = 7.7 Hz, 2H, 2 × Ar*H*), 7.18–7.09 (m, 2H, 2 × Ar*H*), 6.90–6.87 (m, 3H, 2 × Ar*H* + SC=C*H*), 3.81 (s, 3H, OCH_3), 2.90–2.80 (m, 1H, PhC*H*), 2.74–2.52 (m, 3H, SC=CHCH_2 + SCCH$_2$CH_aH$_b$), 2.40–2.28 (m, 1H, SCCH$_2$CH$_a$$H_b$), 2.19–2.11 (m, 1H, SCC$H_aH_b$), 1.87 (dddd, *J* = 13.2, 11.5, 10.3, 5.6 Hz, 1H, SCCH$_a$$H_b$);

^{13}C {^{1}H} NMR (101 MHz, $CDCl_3$) δ 158.3, 144.6, 143.1, 141.7, 134.9, 128.9, 126.9, 126.8, 123.3, 114.8, 55.8, 38.5, 33.3, 29.1, 24.5;

IR ν_{max} (neat)/cm^{-1} 3028, 2931, 2839, 1502, 1365, 1251, 1191, 1167, 1149;

LRMS (ESI$^+$) *m/z* 367.0 ([M+Na]$^+$);

HRMS (ESI$^+$) found *m/z* 367.0975 [M+H]$^+$, $C_{19}H_{20}O_4SNa^+$ requires *m/z* 367.0975.

3.5.2 Nucleophilic addition of pyrrolidine for the preparation of 1-((1,2,3,6-tetrahydro-[1,1'-biphenyl]-4-yl)sulfonyl)pyrrolidine (7)

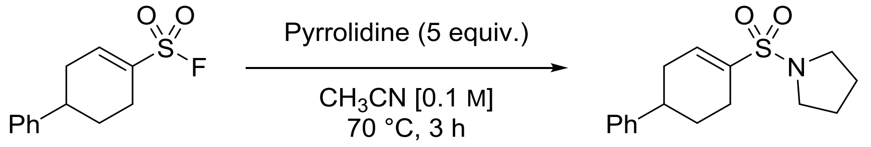

5b **7** 51%

A reaction tube was charged with alkenylsulfonyl fluoride **5b** (24.0 mg, 0.10 mmol, 1.0 equiv.), pyrrolidine (42 µL, 0.50 mmol, 5.0 equiv.) and CH_3CN (1.0 mL). The reaction was heated to 70 °C and stirred for 3 h, which was then cooled to ambient temperature, diluted with hexane and directly purified by flash column chromatography (10–25% EtOAc in petrol), affording the *alkenyl sulfonamide* **7** as a white solid (14.8 mg, 51%);

mp 79–81 °C (EtOAc);

R*f* 0.30 (25% EtOAc in hexane) [$KMnO_4$/UV];

^{1}H NMR (400 MHz, $CDCl_3$) δ 7.35–7.30 (m, 2H, 2 × Ar*H*), 7.26–7.19 (m, 3H, 3 × Ar*H*), 6.88–6.82 (m, 1H, SC=C*H*), 3.38–3.27 (m, 4H, 2 × NCH_2), 2.89–2.79 (m, 1H, PhC*H*), 2.64–2.48 (m, 2H, SC=CHC$H_a$$H_b$ + SCC$H_c$$H_d$), 2.47–2.32 (m, 2H, SC=CHC$H_a$$H_b$ + SCC$H_c$$H_d$), 2.13–2.03 (m, 1H, SC$CH_2CH_a$$H_b$), 1.96–1.88 (m, 4H, N$CH_2CH_2$), 1.88–1.79 (m, 1H, SC$CH_2CH_a$$H_b$);

^{13}C {^{1}H} NMR (101 MHz, $CDCl_3$) δ 145.1, 137.3, 136.9, 128.8, 126.9, 126.7, 47.8, 38.8, 33.1, 29.4, 26.0, 24.6;

IR ν_{max} (neat)/cm^{-1} 2938, 2880, 1646, 1602, 1494, 1452, 1326, 1147;

LRMS (ESI$^+$) *m*/*z* 292.1 ([M+H]$^+$);

HRMS (ESI$^+$) found *m*/*z* 292.1365 [M+H]$^+$, $C_{16}H_{22}NO_2S^+$ requires *m*/*z* 292.1366.

3.5.3 General Procedure D: Synthesis of alkenyl sulfonamides using alkenylsulfonyl fluoride, anilines and $Ca(NTf_2)_2$ as exemplified by the preparation of *tert*-butyl 4-(*N*-(*p*-tolyl)sulfamoyl)-3,6-dihydropyridine-1(2*H*)-carboxylate (8a)

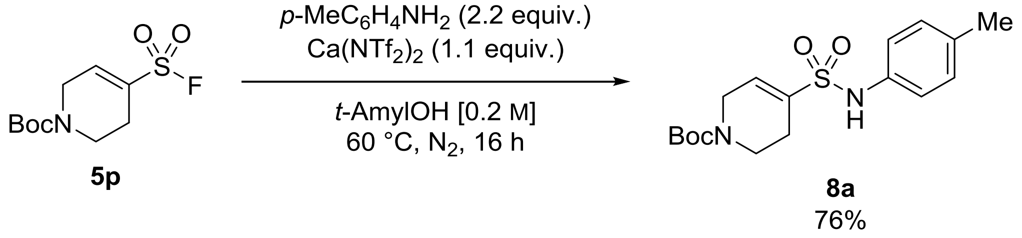

A reaction tube was charged with alkenylsulfonyl fluoride **5p** (26.5 mg, 0.10 mmol, 1.0 equiv.), *p*-toluidine (23.5 mg, 0.22 mmol, 2.2 equiv.), $Ca(NTf_2)_2$ (66.0 mg, 0.11 mmol, 1.1 equiv.) and *t*-amyl alcohol (0.5 mL), evacuated and back-filled with N_2 for three times. The reaction was heated at 60 °C for 16 h under N_2, which was then cooled to ambient temperature, diluted with EtOAc, washed subsequently with 1 M aq. HCl and brine, dried over anhydrous $MgSO_4$, filtered and concentrated *in vacuo*. The crude mixture was then purified by flash column chromatography (20–40% EtOAc in petrol), affording the *alkenyl sulfonamide* **8a** as a light brown solid (26.6 mg, 76%);

mp 195–196 °C (EtOAc);

$\mathbf{R}_f$ 0.60 (50% EtOAc in hexane) [$KMnO_4$/UV];

^{1}**H NMR** (400 MHz, $CDCl_3$) δ 7.10 (d, *J* = 7.9 Hz, 2H, 2 × Ar*H*), 7.02 (d, *J* = 8.4 Hz, 2H, 2 × Ar*H*), 6.71 (br. s, 1H, N*H*), 6.54 (app. s, 1H, SC=C*H*), 4.02 (app. s, 2H, SC=CHC*H*$_2$), 3.49 (t, *J* = 5.6 Hz, 2H, SCCH$_2$C*H*$_2$), 2.39 (app. br. s, 2H, SCC*H*$_2$), 2.31 (s, 3H, ArC*H*$_3$), 1.44 (s, 9H, C(C*H*$_3$)$_3$) (recorded as a pair of rotamers);

^{13}**C {^{1}H} NMR** (101 MHz, $CDCl_3$) δ 154.5, 136.3, 135.6, {135.3, 134.8}, 133.7, 130.2, 121.6, 80.7, {43.7, 43.1}, {40.4, 39.1}, 28.5, 24.2, 21.0 (recorded as a pair of rotamers);

IR ν_{max} (neat)/cm^{-1} 3241, 2976, 2926, 1697, 1674, 1511, 1334, 1282, 1241, 1157;

HRMS (ESI$^-$) found *m/z* 351.1385 [M–H]$^-$, $C_{17}H_{23}N_2O_4S^-$ requires *m/z* 351.1384.

***N*-phenyl-1-tosyl-1,2,3,6-tetrahydropyridine-4-sulfonamide (8b)**

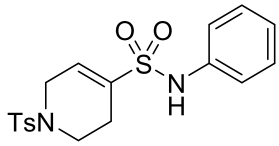

8b

Prepared according to General Procedure D using alkenylsulfonyl fluoride **5p** (26.5 mg, 0.10 mmol, 1.0 equiv.), aniline (20 µL, 0.22 mmol, 2.2 equiv.), $Ca(NTf_2)_2$ (120 mg, 0.20 mmol, 2.0 equiv.) and *t*-amyl alcohol (0.5 mL). The crude product was purified by flash column chromatography (20–40% EtOAc in petrol) to afford the *alkenyl sulfonamide* **8b** as a brown oil (21.4 mg, 55%);

R*f* 0.21 (30% EtOAc in hexane) [$KMnO_4$/UV];

^{1}H NMR (400 MHz, $CDCl_3$) δ 7.63 (d, *J* = 8.3 Hz, 2H, 2 × Ar*H*), 7.30 (t, *J* = 7.6 Hz, 4H, 4 × Ar*H*), 7.16 (tt, *J* = 7.4, 1.4 Hz, 1H, Ar*H*), 7.09 (dd, *J* = 8.5, 1.2 Hz, 2H, 2 × Ar*H*), 6.69 (tt, *J* = 3.2, 1.5 Hz, 1H, SC=C*H*), 6.45 (br. s, 1H, N*H*), 3.75 (dt, *J* = 3.2, 2.7 Hz, 2H, SC=CHCH_2), 3.21 (t, *J* = 5.6 Hz, 2H, $SCCH_2CH_2$), 2.50–2.44 (m, 2H, $SCCH_2$), 2.43 (s, 3H, $ArCH_3$);

^{13}C {^{1}H} NMR (101 MHz, $CDCl_3$) δ 144.4, 136.7, 136.2, 133.2, 133.1, 130.1, 129.7, 127.7, 125.7, 121.0, 44.7, 42.2, 24.4, 21.7;

IR ν_{max} (neat)/cm^{-1} 3262, 2923, 2854, 1598, 1495, 1338, 1156;

LRMS (ESI$^+$) *m/z* 415.0 ([M+Na]$^+$);

HRMS (ESI$^+$) found *m/z* 393.0936 [M+H]$^+$, $C_{18}H_{20}N_2O_4S_2^+$ requires *m/z* 393.0937.

3.5.4 General Procedure E: Conjugate addition of thiols or thiophenols to alkenylsulfonyl fluorides as exemplified by the preparation of *tert*-butyl 3-((4-fluorophenyl)thio)-4-(fluorosulfonyl)piperidine-1-carboxylate (9a)

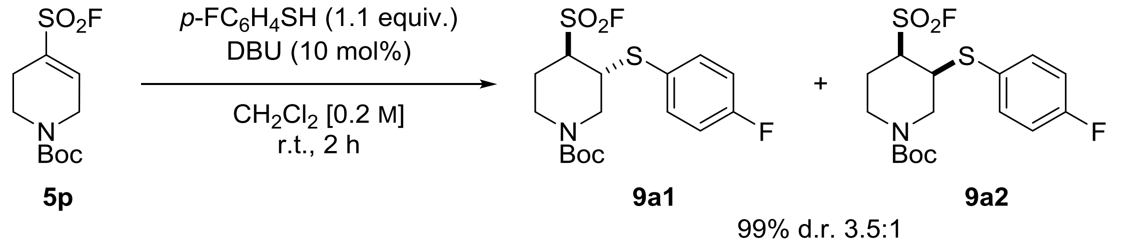

Alkenylsulfonyl fluoride **5p** (26.5 mg, 0.10 mmol, 1.0 equiv.) and 4-fluorothiophenol (12 µL, 0.11 mmol, 1.1 equiv.) were dissolved in CH_2Cl_2 in a sample vial and DBU (1.5 µL, 0.010 mmol, 0.10 equiv.) was added as a solution in CH_2Cl_2 (0.1 mL). The reaction was stirred at ambient temperature for 2 h until completion, which was then directly purified by flash column chromatography (10–30% EtOAc in petrol), affording the *trans-sulfonyl fluoride* **9a1** as a white solid (8.7 mg, 22%) and *cis-sulfonyl fluoride* **9a2** as a white solid (30.4 mg, 77%);

tert-Butyl *trans*-3-((4-fluorophenyl)thio)-4-(fluorosulfonyl)piperidine-1-carboxylate **9a1**:

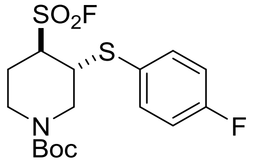

mp 84–86 °C (EtOAc);

R*f* 0.58 (30% EtOAc in hexane) [$KMnO_4$/UV];

^{1}H NMR (500 MHz, DMSO-d_6, 363 K) δ 7.59 (dd, J = 8.8, 5.3 Hz, 2H, 2 × ArCH), 7.24 (t, J = 8.8 Hz, 2H, 2 × ArCH), 4.33 (td, J = 9.2, 4.7 Hz, 1H, FO_2SCH), 3.95 (dd, J = 13.9, 4.2 Hz, 1H, NCH_aH_bCH), 3.78 (dt, J = 13.5, 4.8 Hz, 1H, $NCH_cH_dCH_2$), 3.43 (td, J = 8.6, 4.1 Hz, 1H, ArSCH), 3.27–3.13 (m, 2H, NCH_aH_bCH + $NCH_cH_dCH_2$), 2.39–2.30 (m, 1H, $NCH_2CH_eH_f$), 1.91 (dtd, J = 14.1, 9.7, 4.4 Hz, 1H, $NCH_2CH_eH_f$), 1.35 (s, 9H, $C(CH_3)_3$);

^{13}C {^{1}H} NMR (126 MHz, DMSO-d_6, 363 K) δ 162.0 (d, $^1J_{CF}$ = 246.7 Hz), 153.0, 135.0 (d, $^3J_{CF}$ = 8.3 Hz), 126.9, 116.1 (d, $^2J_{CF}$ = 22.2 Hz), 79.2, 62.0 (d, $^2J_{CF}$ = 10.3 Hz), 46.4, 44.1, 40.3, 27.5, 25.2;

^{19}F {^{1}H} NMR (470 MHz, DMSO-d_6, 363 K) δ 54.4 (s, 1F, SO_2F), -113.0 (s, 1F, ArF);

IR ν_{max} (neat)/cm^{-1} 2976, 2928, 1696, 1590, 1491, 1402, 1225, 1198, 1159, 1123;

HRMS (ESI$^+$) found m/z 416.0772 [M+Na]$^+$, $C_{16}H_{21}F_2NO_4S_2Na^+$ requires m/z 416.0772.

tert-Butyl *cis*-3-((4-fluorophenyl)thio)-4-(fluorosulfonyl)piperidine-1-carboxylate **9a2**:

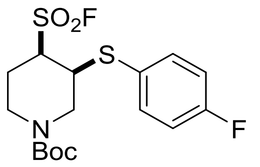

mp 89–91 °C (EtOAc);

$\mathbf{R}_f$ 0.39 (30% EtOAc in hexane) [$KMnO_4$/UV];

^{1}H NMR (500 MHz, DMSO-d_6, 363 K) δ 7.62–7.50 (m, 2H, 2 × ArC*H*), 7.22 (t, *J* = 9.1, 2H, 2 × ArC*H*), 4.75 (dt, *J* = 12.2, 3.2 Hz, 1H, FO_2SCH), 4.21–4.11 (m, 2H, NCH_aH_bCH + $NCH_cH_dCH_2$), 3.83–3.78 (m, 1H, ArSC*H*), 3.22 (d, *J* = 14.0 Hz, 1H, NCH_aH_bCH), 2.96 (ddd, *J* = 14.6, 12.3, 3.2 Hz, 1H, $NCH_cH_dCH_2$), 2.16–2.07 (m, 1H, $NCH_2CH_eH_f$), 1.95 (qd, *J* = 12.2, 4.7 Hz, 1H, $NCH_2CH_eH_f$), 1.41 (s, 9H, $C(CH_3)_3$);

^{13}C {^{1}H} NMR (126 MHz, DMSO-d_6, 363 K) δ 161.7 (d, $^1J_{CF}$ = 246.7 Hz), 153.6, 134.1, 128.3 (d, $^3J_{CF}$ = 3.0 Hz), 115.8 (d, $^2J_{CF}$ = 22.0 Hz), 78.9, 62.6 (d, $^2J_{CF}$ = 13.1 Hz), 46.5, 45.6, 41.0, 27.6, 22.5;

^{19}F {^{1}H} NMR (470 MHz, DMSO-d_6, 363 K) δ 54.4 (s, 1F, SO_2F), -113.8 (s, 1F, Ar*F*);

IR ν_{max} (neat)/cm^{-1} 2976, 2930, 2866, 1694, 1491, 1406, 1367, 1286, 1239, 1158;

HRMS (ESI$^+$) found *m*/*z* 416.0772 $[M+Na]^+$, $C_{16}H_{21}F_2NO_4S_2Na^+$ requires *m*/*z* 416.0772.

3-((4-Fluorophenyl)thio)-1-tosylpiperidine-4-sulfonyl fluoride (9b)

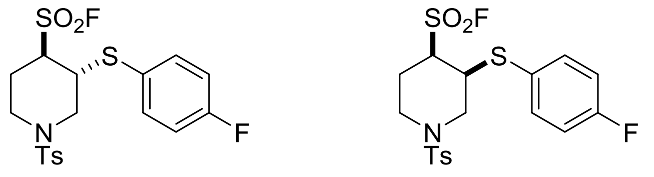

9b1 **9b2**

99% d.r. 2.2:1

Prepared according to General Procedure E using alkenylsulfonyl fluoride **5p** (49.8 mg, 0.156 mmol), 4-fluorothiophenol (18 µL, 0.17 mmol), DBU (2.2 µL, 0.015 mmol) and CH_2Cl_2 (0.75 mL). The crude product was purified by flash column chromatography (10–25% EtOAc in petrol), affording the *trans-sulfonyl fluoride* **9b1** as a white solid (48.0 mg, 69%) and *cis-sulfonyl fluoride* **9b2** as a white solid (21.8 mg, 31%);

trans-3-((4-Fluorophenyl)thio)-1-tosylpiperidine-4-sulfonyl fluoride **9b1**:

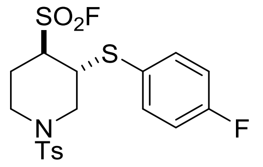

mp 118–120 °C (EtOAc);

R_f 0.45 (30% EtOAc in hexane) [$KMnO_4$/UV];

^{1}H NMR (400 MHz, $CDCl_3$) δ 7.60 (d, J = 8.3 Hz, 2H, 2 × ArH), 7.52 (dd, J = 8.8, 5.2 Hz, 2H, 2 × ArH), 7.33 (d, J = 7.6 Hz, 2H, 2 × ArH), 7.08 (t, J = 8.6 Hz, 2H, 2 × ArH), 3.59–3.52 (m, 2H, NCH_aH_bCH + ArSCH), 3.50–3.43 (m, 1H, FO_2SCH), 3.37–3.29 (m, 1H, $NCH_cH_dCH_2$), 3.18–3.11 (m, 1H, NCH_aH_bCH), 3.07 (ddd, J = 12.0, 7.4, 4.0 Hz, 1H, $NCH_cH_dCH_2$), 2.63–2.53 (m, 1H, $NCH_2CH_eH_f$), 2.44 (s, 3H, CH_3), 2.20 (dtd, J = 14.4, 7.2, 3.7 Hz, 1H, $NCH_2CH_eH_f$);

^{13}C {^{1}H} NMR (101 MHz, $CDCl_3$) δ 163.5 (d, $^1J_{CF}$ = 250.8 Hz), 144.5, 136.4 (d, $^3J_{CF}$ = 8.4 Hz), 133.2, 130.1, 127.6, 126.6 (d, $^4J_{CF}$ = 3.3 Hz), 117.0 (d, $^2J_{CF}$ = 22.1 Hz), 61.1 (d, $^2J_{CF}$ = 11.2 Hz), 48.5, 44.4, 42.9, 24.4, 21.7;

^{19}F NMR (377 MHz, $CDCl_3$) δ 54.5 (s, 1F, SO_2F), -110.9 (tt, J = 8.4, 5.3 Hz, 1F, ArF);

IR ν_{max} (neat)/cm^{-1} 3065, 2926, 2857, 1590, 1491, 1404, 1226, 1199, 1165;

HRMS (ESI$^+$) found m/z 448.0518 [M+H]$^+$, $C_{18}H_{20}F_2NO_4S_3^+$ requires m/z 448.0517.

cis-3-((4-Fluorophenyl)thio)-1-tosylpiperidine-4-sulfonyl fluoride **9b2**:

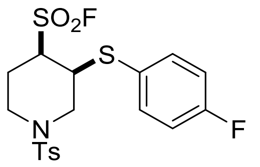

mp 104–105 °C (EtOAc);

R*f* 0.32 (30% EtOAc in hexane) [$KMnO_4$/UV];

^{1}H NMR (500 MHz, $CDCl_3$) δ 7.67 (d, J = 8.4 Hz, 2H, 2 × Ar*H*), 7.60 (dd, J = 8.8, 5.2 Hz, 2H, 2 × Ar*H*), 7.33 (d, J = 8.0 Hz, 2H, 2 × Ar*H*), 7.06 (t, J = 8.6 Hz, 2H, 2 × Ar*H*), 3.89 (dd, J = 12.6, 4.2 Hz, 2H, $NCH_aH_bCH_2$ + NCH_cH_dCH), 3.64 (dtd, J = 11.4, 3.8, 1.2 Hz, 1H, FO_2SCH), 3.58 (qd, J = 3.8, 2.5, 1.7 Hz, 1H, ArSC*H*), 2.71 (dd, J = 12.6, 2.4 Hz, 1H, NCH_cH_dCH), 2.58 (td, J = 11.8, 3.1 Hz, 1H, $NCH_aH_bCH_2$), 2.43–2.34 (m, 1H,$NCH_2CH_eH_f$), 2.20 (ddd, J = 13.6, 3.5, 1.4 Hz, 1H, $NCH_2CH_eH_f$);

^{13}C {^{1}H} NMR (101 MHz, $CDCl_3$) δ 163.6 (d, $^1J_{CF}$ = 250.2 Hz), 144.4, 137.3 (d, $^3J_{CF}$ = 8.6 Hz), 133.4, 130.1, 127.8, 127.7 (d, $^4J_{CF}$ = 3.2 Hz), 116.8 (d, $^2J_{CF}$ = 21.9 Hz), 63.2 (d, $^2J_{CF}$ = 15.0 Hz), 49.9, 47.0, 44.4, 23.7, 21.7;

^{19}F NMR (377 MHz, $CDCl_3$) δ 55.3 (s, 1F, SO_2F), -111.3 (tt, J = 8.4, 5.4 Hz, 1F, Ar*F*);

IR ν_{max} (neat)/cm^{-1} 2924, 2850, 1590, 1491, 1407, 1163;

HRMS (ESI$^+$) found *m/z* 448.0518 $[M+H]^+$, $C_{18}H_{20}F_2NO_4S_3^+$ requires *m/z* 448.0517.

3-((4-Methoxybenzyl)thio)-1-tosylpiperidine-4-sulfonyl fluoride (9c)

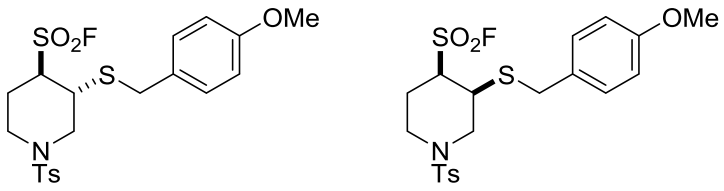

9c1 **9c2**

98% d.r. 2.9:1

Prepared according to General Procedure E using alkenylsulfonyl fluoride **5p** (47.9 mg, 0.150 mmol), 4-methoxybenzyl mercaptan (23 μL, 0.165 mmol), DBU (2.2 μL, 0.015 mmol) and CH_2Cl_2 (0.75 mL), and the reaction was stirred for 1 h. The crude product was purified by flash column chromatography (10–25% EtOAc in petrol), affording the *trans-sulfonyl fluoride* **9c1** as an off-white solid (51.7 mg, 73%) and *cis-sulfonyl fluoride* **9c2** as an off-white solid (17.8 mg, 25%);

trans-3-((4-Methoxybenzyl)thio)-1-tosylpiperidine-4-sulfonyl fluoride **9c1**:

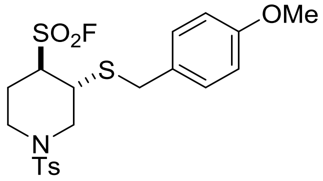

mp 79–81 °C (EtOAc);

bp 131 °C (decomp.);

$\mathbf{R}_f$ 0.41 (30% EtOAc in hexane) [$KMnO_4$/UV];

^{1}H NMR (500 MHz, $CDCl_3$) δ 7.57 (d, J = 8.3 Hz, 2H, 2 × ArH), 7.31 (d, J = 8.0 Hz, 2H, 2 × ArH), 7.26 (d, J = 8.6 Hz, 2H, 2 × ArH), 6.88 (d, J = 8.6 Hz, 2H, 2 × ArH), 3.85 (d, J = 13.7 Hz, 1H, SCH_aH$_b$), 3.82 (s, 3H, OCH_3), 3.81 (d, J = 13.7 Hz, 1H, SCH$_a$$H_b$), 3.44 (dd, J = 12.7, 3.9 Hz, 1H, NCH_cH$_d$CH), 3.34 (td, J = 6.7, 4.8 Hz, 1H, FO$_2$SCH), 3.28 (dq, J = 8.2, 4.0, 3.5 Hz, 1H, NCH_eH$_f$CH$_2$), 3.18 (td, J = 6.8, 3.8 Hz, 1H, NCH$_2$CH), 2.98 (td, J = 12.6, 11.0, 5.2 Hz, 2H, NCH$_e$$H_fCH_2$ + NCH$_c$$H_d$CH), 2.55–2.47 (m, 1H, NCH$_2$CH_gH$_h$), 2.43 (s, 3H, CH_3), 2.13 (dtd, J = 14.6, 7.3, 3.8 Hz, 1H, NCH$_2$CH$_g$$H_h$);

^{13}C {^{1}H} NMR (101 MHz, $CDCl_3$) δ 159.3, 144.3, 133.1, 130.2, 130.1, 128.6, 127.7, 114.5, 62.1 (d, $^2J_{CF}$ = 10.0 Hz), 55.5, 49.3, 43.0, 39.8, 36.4, 24.6, 21.7;

^{19}F {^{1}H} NMR (377 MHz, $CDCl_3$) δ 54.5;

IR ν_{max} (neat)/cm^{-1} 2976, 2928, 2870, 1696, 1491, 1402, 1279, 1225, 1198, 1159;

HRMS (ESI$^+$) found m/z 496.0692 [M+Na]$^+$, $C_{20}H_{24}FNO_5S_3Na^+$ requires m/z 496.0693.

cis-3-((4-Methoxybenzyl)thio)-1-tosylpiperidine-4-sulfonyl fluoride **9c2**:

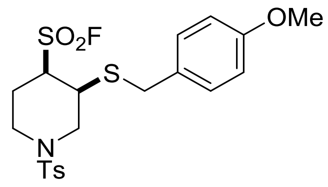

mp 143–145 °C (decomp.) (EtOAc);

R*f* 0.31 (30% EtOAc in hexane) [$KMnO_4$/UV];

^{1}H NMR (400 MHz, $CDCl_3$) δ 7.63 (d, *J* = 8.3 Hz, 2H, 2 × Ar*H*), 7.32 (d, *J* = 8.0 Hz, 2H, 2 × Ar*H*), 7.29 (d, *J* = 8.6 Hz, 2H, 2 × Ar*H*), 6.87 (d, *J* = 8.6 Hz, 2H, 2 × Ar*H*), 3.86 (d, *J* = 13.5 Hz, 1H, SCH_aH_b), 3.82 (s, 3H, OCH_3), 3.80 (d, *J* = 13.4 Hz, 1H, SCH_aH_b), 3.74 (d, *J* = 12.6 Hz, 1H, $NCH_cH_dCH_2$), 3.60 (dd, *J* = 12.3, 4.3 Hz, 1H, NCH_eH_fCH), 3.50 (dt, *J* = 10.8, 3.1 Hz, 1H, FO_2SCH), 3.26 (dq, *J* = 5.0, 3.0, 2.2 Hz, 1H, NCH_2CH), 2.68 (dd, *J* = 12.3, 2.6 Hz, 1H, NCH_eH_fCH), 2.53 (td, *J* = 11.5, 3.0 Hz, 1H, $NCH_cH_dCH_2$), 2.43 (s, 3H, CCH_3), 2.42 (dd, *J* = 13.6, 10.8 Hz, 1H, $NCH_2CH_gH_h$), 2.15 (dd, *J* = 13.6, 3.8 Hz, 1H, $NCH_2CH_gH_h$);

^{13}C {^{1}H} NMR (101 MHz, $CDCl_3$) δ 159.1, 144.3, 133.3, 130.5, 130.0, 128.7, 127.8, 114.3, 63.2 ($^2J_{CF}$ = 12.9 Hz), 55.5, 51.5, 44.2, 39.6, 36.3, 24.0, 21.7;

^{19}F {^{1}H} NMR (377 MHz, $CDCl_3$) δ 54.6;

IR ν_{max} (neat)/cm^{-1} 2976, 2930, 2866, 1694, 1491, 1406, 1286, 1239, 1158;

HRMS (ESI$^+$) found *m*/*z* 496.0692 [M+Na]$^+$, $C_{20}H_{24}FNO_5S_3Na^+$ requires *m*/*z* 496.0693.

3.5.5 Pd-catalysed hydrogenation of alkenylsulfonyl fluoride for the preparation of 1,4-dioxaspiro[4.5]decane-8-sulfonyl fluoride (10)

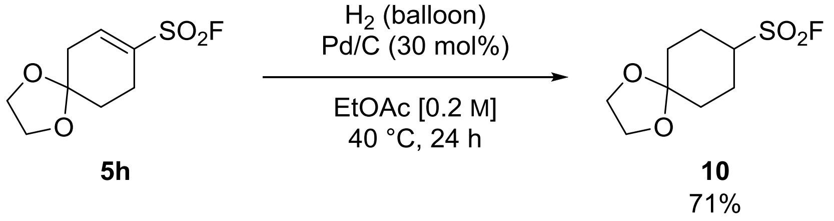

A reaction tube was charged with alkenylsulfonyl fluoride **5h** (67 mg, 0.30 mmol, 1.0 equiv.) and 10 wt% Pd on carbon (95 mg, 0.090 mmol, 0.30 equiv.), which was evacuated and back-filled with H_2 for three times. EtOAc (1.5 mL) was bubbled with H_2 for 10 min and then added to the mixture. The reaction was heated at 40 °C for 24 h with three balloons of H_2 attached, which was then cooled to ambient temperature, diluted with EtOAc and filtered over Celite®. The filtrate was concentrated *in vacuo* and purified by flash column chromatography (20:10:1 CH_2Cl_2/petrol/EtOAc mixture), affording the *sulfonyl fluoride* **10** as a white solid (47.8 mg, 71%);

mp 64–66 °C (EtOAc);

R*f* 0.53 (20:10:2.5 CH_2Cl_2/hexane/EtOAc) [Anisaldehyde–purple (hot) / brown (cooled)];

^{1}H NMR (400 MHz, $CDCl_3$) δ 3.96 (t, J = 2.1 Hz, 4H, 2 × OCH_2), 3.33 (ttd, J = 11.9, 3.8, 2.1 Hz, 1H, SCH), 2.36–2.24 (m, 2H, 2 × SCHCH_aH$_b$), 2.07 (tdd, J = 13.3, 11.9, 4.0 Hz, 2H, 2 × SCHCH$_a$$H_b$), 1.93 (dddd, J = 14.5, 5.4, 2.6, 1.4 Hz, 2H, 2 × CCH_aH$_b$), 1.62 (td, J = 13.5, 4.4 Hz, 2H, 2 × CCH$_a$$H_b$);

^{13}C {^{1}H} NMR (101 MHz, $CDCl_3$) δ 106.7, 64.8, 64.7, 59.4 (d, $^2J_{CF}$ = 13.6 Hz), 33.3, 24.3;

^{19}F {^{1}H} NMR (377 MHz, $CDCl_3$) δ 42.7;

IR ν_{max} (neat)/cm^{-1} 2963, 2930, 2893, 1448, 1392, 1198, 1185, 1106;

HRMS (ESI/APCI) not found.

3.5.6 One-pot dealkylation/propagyloxycarbonylation with propargyl chloroformate for the preparation of prop-2-yn-1-yl 4-(fluorosulfonyl)-3,6-dihydropyridine-1(2*H*)-carboxylate (11)

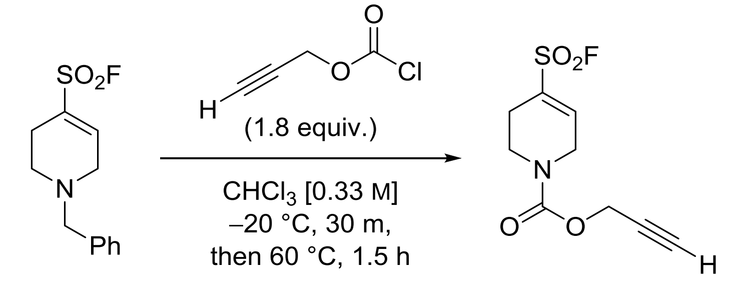

5r **11** 98%

A reaction tube was charged with freshly synthesized alkenylsulfonyl fluoride **5r** (40.0 mg, 0.16 mmol, 1.0 equiv.), evacuated and filled with N_2 for three times, followed by the addition of anhydrous $CHCl_3$ (0.45 mL). The solution was cooled to -20 °C and propargyl chloroformate (18.5 μL, 0.19 mmol, 1.2 equiv.) was added. The reaction was stirred at -20 °C for 30 min, then heated to 60 °C for 30 min. Another portion of propargyl chloroformate (9.5 μL, 0.097 mmol, 0.60 equiv.) was added and the reaction was stirred at 60 °C for 1 h, which was then cooled to ambient temperature and directly purified by flash column chromatography (15–25% EtOAc in petrol), affording the *sulfonyl fluoride* **11** as a colourless oil (37.2 mg, 98%);

R*f* 0.18 (20% EtOAc in hexane) [$KMnO_4$/UV];

^{1}H NMR (400 MHz, $CDCl_3$) δ 7.11 (br. s, 0.5H, SC=CH^{rot1}), 7.07 (br. s, 0.5H, SC=CH^{rot2}), 4.75 (d, J = 2.4 Hz, 2H, OCH_2), 4.31 (quint, J = 2.9 Hz, 2H, CHCH_2), 3.73 (t, J = 5.7 Hz, 2H, $SCCH_2$CH_2), 2.62 (br. s, 2H, SCCH_2), 2.50 (t, J = 2.4 Hz, 1H, OCH_2CC*H*) (recorded as a pair of rotamers);

^{13}C {^{1}H} NMR (101 MHz, $CDCl_3$) δ 154.1, {139.9, 139.2}, 132.7, 77.9, 75.3, 53.7, 43.7, {40.0, 39.7}, {24.2, 24.0}(recorded as a pair of rotamers);

^{19}F {^{1}H} NMR (377 MHz, $CDCl_3$) δ 51.44, 51.40 (recorded as a pair of rotamers);

IR ν_{max} (neat)/cm^{-1} 3293, 2953, 2129, 1710, 1404, 1237, 1203;

HRMS (ESI/APCI) not found.

3.5.7 Copper(I)-catalysed azide-alkyne cycloaddition (CuAAC) with alkenylsulfonyl fluoride 11 and benzyl azide for the preparation of (1-benzyl-1*H*-1,2,3-triazol-4-yl)methyl 4-(fluorosulfonyl)-3,6-dihydro-pyridine-1(2*H*)-carboxylate (12)

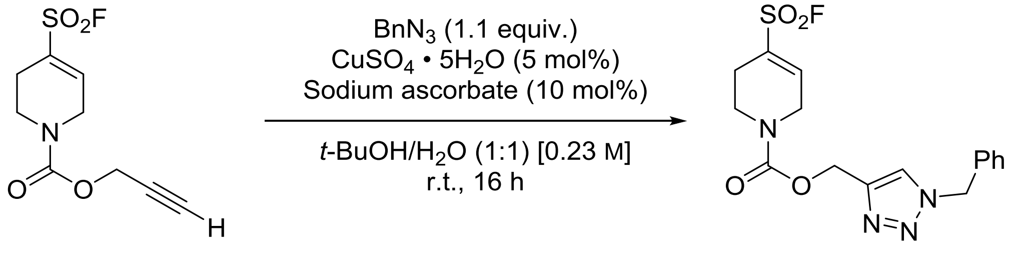

A sample vial was charged with alkenylsulfonyl fluoride **11** (34.5 mg, 0.138 mmol, 1.0 equiv.), $CuSO_4$•$5H_2O$ (1.7 mg, 7.0 μmol, 0.050 equiv.), sodium ascorbate (2.8 mg, 14 μmol, 0.10 equiv.), *t*-butanol (0.3 mL) and deionised water (0.3 mL), followed by the addition of benzyl azide (19 μL, 0.15 mmol, 1.1 equiv.), The reaction was stirred vigorously at ambient temperature for 16 h, which was then diluted with ethyl acetate and water. The two phases were separated and the aqueous phase was extracted with EtOAc for three times. The combined organic extract was washed with brine, dried with anhydrous $MgSO_4$, filtered and concentrated *in vacuo*. The crude mixture was then purified by flash column chromatography (50–70% EtOAc in petrol), affording the *sulfonyl fluoride* **12** as a yellow oil (52.7 mg, 99%);

R_f 0.46 (75% EtOAc in hexane) [$KMnO_4$/UV];

^{1}H NMR (400 MHz, $CDCl_3$) δ 7.55 (s, 1H, NC=C*H*), 7.40–7.36 (m, 3H, 3 × Ar*H*), 7.30–7.27 (m, 2H, 2 × Ar*H*), 7.08 (br. s, 0.5H, SC=CH^{rot1}), 7.02 (br. s, 0.5H, SC=CH^{rot2}), 5.52 (s, 2H, PhCH_2), 5.24 (s, 2H, OCH_2), 4.27–4.23 (m, 2H, SC=CHCH_2), 3.68 (t, *J* = 5.6 Hz, 2H, NC$H_2$$CH_2$), 2.56 (br. s, 2H, N$CH_2CH_2$) (recorded as a pair of rotamers);

^{13}C {^{1}H} NMR (101 MHz, $CDCl_3$) δ 154.8, 143.4, {139.8, 139.4}, 134.5, {132.9, 132.7}, 129.3, 129.0, 128.3, 124.0, 59.0, 54.4, 43.6, {40.0, 39.5}, {24.2, 23.9}(recorded as a pair of rotamers);

^{19}F {^{1}H} NMR (377 MHz, $CDCl_3$) δ 51.4;

IR ν_{max} (neat)/cm^{-1} 3142, 3066, 2953, 1704, 1432, 1402, 1280, 1236, 1202, 1113;

LRMS (ESI$^+$) *m/z* 403.0 ([M+Na]$^+$);

HRMS (ESI$^+$) found *m/z* 381.1028 [M+H]$^+$, $C_{16}H_{18}FN_4O_4S^+$ requires *m/z* 381.1027.

3.5.8 Boc deprotection using HCl in 1,4-dioxane for the preparation of 1,2,3,6-tetrahydropyridine-4-sulfonyl fluoride hydrochloride (13)

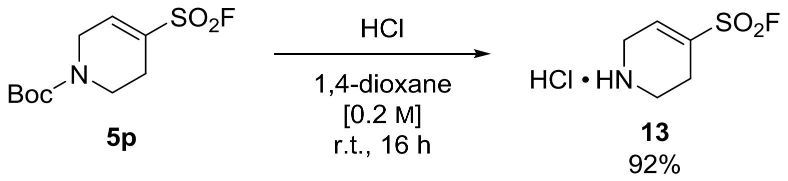

A reaction tube was charged with alkenylsulfonyl fluoride **5p** (53.0 mg, 0.20 mmol, 1.0 equiv.), evacuated and filled with N_2 for three times, followed by the addition of anhydrous 1,4-dioxane (0.5 mL) and hydrogen chloride solution (4 N in 1,4-dioxane) (0.5 mL). The reaction was stirred at ambient temperature for 16 h, and then filtered under vacuum. The residue was washed with Et_2O, affording the *alkenylsulfonyl fluoride* **13** as a white solid (37.2 mg, 92%);

mp 175 °C (decomp.) (EtOAc);

^{1}H NMR (400 MHz, CD_3OD) δ 7.25 (dt, J = 2.9, 1.5 Hz, 1H, SC=C*H*), 4.07 (dt, J = 5.8, 2.6 Hz, 2H, CHCH_2), 3.52 (t, J = 6.1 Hz, 2H, CCH_2CH_2), 2.85 (tt, J = 6.1, 2.3 Hz, 2H, SCCH_2);

^{13}C {^{1}H} NMR (101 MHz, CD_3OD) δ 138.0, 133.2 (d, $^2J_{CF}$ = 27.7 Hz), 43.1, 41.0, 21.8;

^{19}F {^{1}H} NMR (377 MHz, CD_3OD) δ 50.1;

IR ν_{max} (neat)/cm^{-1} 2963, 2730, 1658, 1542, 1450, 1392, 1209;

LRMS (ESI$^+$) *m*/*z* 166.0 ([M+H]$^+$);

HRMS (ESI$^+$) found *m*/*z* 166.0333 [M+H]$^+$, $C_5H_9FNO_2S^+$ requires *m*/*z* 166.0333.

3.5.9 General Procedure E: Boc deprotection using TFA for the preparation of 1,2,3,6-tetrahydropyridine-4-sulfonyl fluoride trifluoroacetate (14)

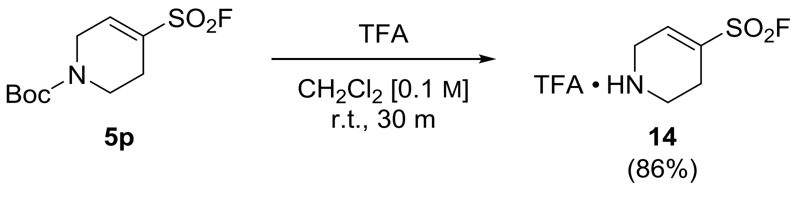

To a solution of alkenylsulfonyl fluoride **5p** (650 mg, 0.25 mmol, 1.0 equiv.) in CH_2Cl_2 (1.6 mL) was added trifluoroacetic acid (0.4 mL), which was stirred at ambient temperature for 30 min. Toluene (0.5 mL) was then added and the mixture was concentrated *in vacuo*. Another portion of toluene (0.5 mL) was added and upon removal of volatiles *in vacuo*, the *alkenylsulfonyl fluoride* **14** was obtained as an off-white solid (56.5 mg, 86%), which was used for subsequent functionalisation without further purification.

3.5.10 Reductive amination using alkenylsulfonyl fluoride 13, nicotinaldehyde and triacetoxyborohydride for the preparation of 1-(pyridin-3-ylmethyl)-1,2,3,6-tetrahydropyridine-4-sulfonyl fluoride (15)

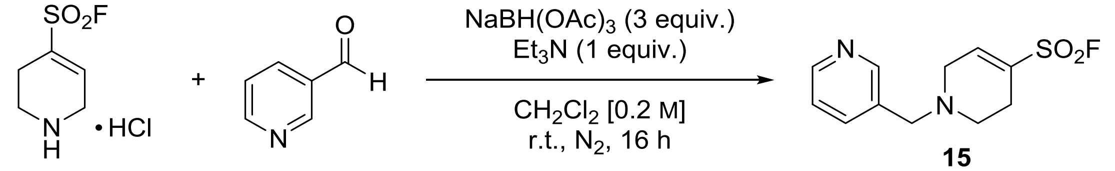

13 (3 equiv.) **15** 78%

A sample vial was charged with alkenylsulfonyl fluoride **13** (20.2 mg, 0.10 mmol, 1.0 equiv.), nicotinaldehyde (32.1 mg, 0.30 mmol, 3.0 equiv.) and $NaBH(OAc)_3$ (63.6 mg, 0.30 mmol, 3.0 equiv.), evacuated and filled with N_2 for three times. A solution of Et_3N (14 µL, 0.10 mmol, 1.0 equiv.) in CH_2Cl_2 (0.5 mL) was then added. The reaction was stirred at ambient temperature for 16 h, which was then diluted with EtOAc and washed with sat. aq. $NaHCO_3$. The organic extract was dried with anhydrous $MgSO_4$, filtered, concentrated *in vacuo* and purified by flash column chromatography (50–100% EtOAc in petrol), affording the *alkenylsulfonyl fluoride* **15** as a red oil (20.0 mg, 78%);

$\mathbf{R}_f$ 0.17 (EtOAc) [$KMnO_4$/UV];

^{1}H NMR (400 MHz, $CDCl_3$) δ 8.55 (dd, J = 3.9, 2.6 Hz, 2H, 2 × Ar*H*), 7.69 (dt, J = 7.8, 2.0 Hz, 1H, Ar*H*), 7.29 (ddd, J = 7.8, 4.8, 0.9 Hz, 1H, Ar*H*), 7.05 (tt, J = 3.3, 1.3 Hz, 1H, SC=C*H*), 3.67 (s, 2H, ArCH_2), 3.28 (quint, J = 3.0 Hz, 2H, CHCH_2), 2.74 (t, J = 5.5 Hz, 2H, CH_2CH_2), 2.63–2.56 (m, 2H, CH_2CH_2);

^{13}C {^{1}H} NMR (101 MHz, $CDCl_3$) δ 150.2, 149.3, 141.2 (d, $^3J_{CF}$ = 2.6 Hz), 136.7, 132.9, 132.4 (d, $^2J_{CF}$ = 24.9 Hz), 123.7, 59.0, 52.1, 48.3, 24.8;

^{19}F {^{1}H} NMR (377 MHz, $CDCl_3$) δ 51.2;

IR ν_{max} (neat)/cm^{-1} 3055, 2922, 2850, 1652, 1577, 1400, 1205;

LRMS (ESI$^+$) *m/z* 257.0 ([M+H]$^+$);

HRMS (ESI$^+$) found *m/z* 257.0755 [M+H]$^+$, $C_{11}H_{14}FN_2O_2S^+$ requires *m/z* 257.0755.

3.5.11 Biotinylation of alkenylsulfonyl fluoride 13 for the preparation of 1-(5-((3a*S*,4*S*,6a*R*)-2-oxohexahydro-1*H*-thieno[3,4-*d*]imidazol-4-yl)pentanoyl)-1,2,3,6-tetrahydropyridine-4-sulfonyl fluoride (16)

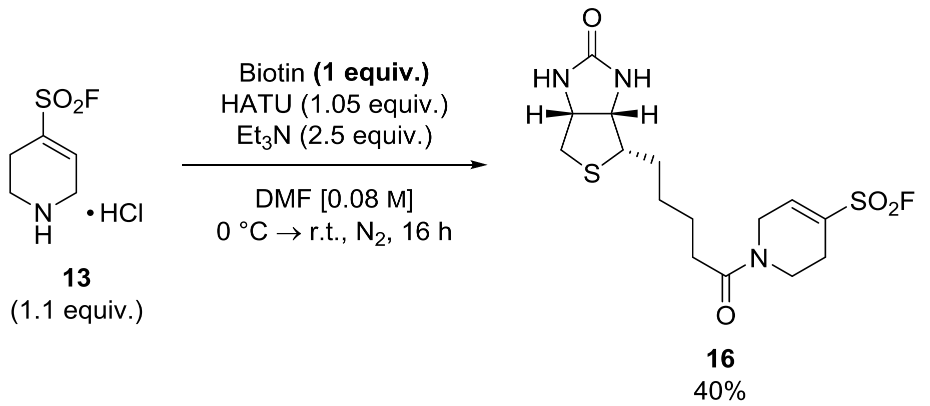

A reaction tube was charged with biotin (32.9 mg, 0.135 mmol, 1.0 equiv.) and HATU (53.2 mg, 0.14 mmol, 1.05 equiv.), evacuated and filled with N_2 for three times. Anhydrous DMF (0.5 mL) and a solution of Et_3N (19 µL, 0.135 mmol, 1.0 equiv.) in DMF (0.5 mL) was then added and was stirred at room temperature for 15 min. The reaction was then cooled to 0 °C, alkenylsulfonyl fluoride **13** (30.3 mg, 0.15 mmol, 1.1 equiv.) was added, followed by a slow addition of a solution of Et_3N (28 µL, 0.20 mmol, 1.5 equiv.) in DMF (0.75 mL) over 30 min. The reaction was warmed up to room temperature gradually and stirred for 16 h under an N_2 atmosphere. The mixture was diluted with EtOAc, washed with 1 M aq. HCl, water and brine, dried with anhydrous $MgSO_4$, filtered and concentrated *in vacuo*. The crude mixture was then purified by flash column chromatography (0–10% MeOH in CH_2Cl_2), affording the *alkenylsulfonyl fluoride* **16** as a white solid (21.2 mg, 40%);

mp 100–102 °C (EtOAc);

$\mathbf{R}_f$ 0.41 (10% MeOH in CH_2Cl_2) [$KMnO_4$/UV];

^{1}H NMR (400 MHz, DMSO-d_6) δ 7.30 (t, J = 3.1 Hz, 0.6H, SC=CH^{rot1}), 7.27 (t, J = 3.0 Hz, 0.4H, SC=CH^{rot2}), 6.42 (br. s, 1H, NH), 6.35 (br. s, 1H, NH), 4.39–4.34 (m, 0.8H, C=CHCH_2^{rot2}), 4.34–4.27 (m, 2.2H, C=CHCH_2^{rot1} + SCH$_2$CH), 4.14 (dd, J = 7.5, 4.5 Hz, 1H, SCHCH), 3.69 (t, J = 5.3 Hz, 0.8H, SCCH$_2$CH_2^{rot2}), 3.66 (t, J = 5.3 Hz, 1.2H, SCCH$_2$CH_2^{rot1}), 3.14–3.07 (m, 1H, SCH), 2.82 (dd, J = 12.5, 5.1 Hz, 1H, SCH_aH$_b$), 2.61–2.55 (m, 2.2H, SCH$_A$$H_B$ + SCCH_2^{rot1}), 2.50–2.45 (m, 0.8H, SCCH_2^{rot2}), 2.39 (t, J = 7.6 Hz, 1.2H, C(=O)CH_2^{rot1}), 2.34 (d, J = 7.8 Hz, 0.8H, C(=O)CH_2^{rot2}), 1.68–1.57 (m, 1H, SCHCH_cH$_d$), 1.56–1.43 (m, 3H, SCHCH$_c$$H_d$ + C(=O)CH$_2$CH_2), 1.40–1.30 (m, 2H, SCHCH$_2$CH_2) (recorded as a pair of rotamers);

^{13}C {^{1}H} NMR (101 MHz, DMSO-d_6) δ {171.3, 171.1}, 162.7, {143.4, 142.8}, 130.4, 61.0, 59.2, 55.5, {44.6, 41.8}, {40.8, 36.7}, 39.9, {32.5, 32.1}, 28.3, 28.1, {24.6, 24.4}, {24.1, 23.3} (recorded as a pair of rotamers);

^{19}F {^{1}H} NMR (377 MHz, DMSO-d_6) δ 52.3, 52.2 (recorded as a pair of rotamers);

IR ν_{max} (neat)/cm^{-1} 3258, 2925, 2856, 1699, 1636, 1432, 1401, 1202;

HRMS (ESI$^+$) found m/z 392.1106 [M+H]$^+$, $C_{15}H_{23}FN_3O_4S_2^+$ requires m/z 392.1109.

3.5.12 Nucleophilic addition of alkenylsulfonyl fluoride 14 to phenyl isocyanate for the preparation of 1-(phenylcarbamoyl)-1,2,3,6-tetrahydro-pyridine-4-sulfonyl fluoride (17)

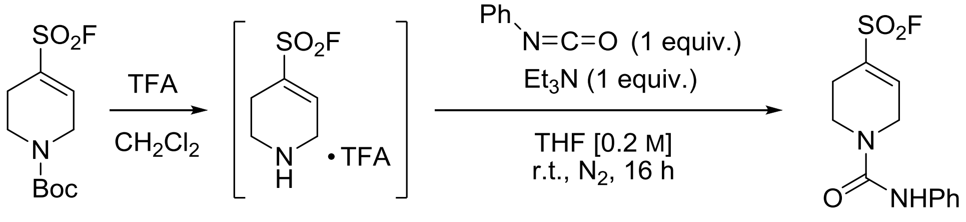

5p **14** **17** 74%

Following General Procedure E, alkenylsulfonyl fluoride **5p** (26.5 mg, 0.10 mmol, 1.0 equiv.) was deprotected and was added with a solution of phenyl isocyanate (11 μL, 0.10 mmol, 1.0 equiv.) and Et_3N (14 μL, 0.10 mmol, 1.0 equiv.) in anhydrous THF (0.5 mL). The reaction was stirred at ambient temperature for 16 h, which was then diluted with EtOAc, filtered over Celite® and concentrated *in vacuo*. The crude product was purified by flash column chromatography (30–45% EtOAc in petrol), affording the *alkenylsulfonyl fluoride* **17** as a white solid (20.9 mg, 74%);

mp 164 °C (decomp.) ($CHCl_3$);

R*f* 0.43 (50% EtOAc in hexane) [$KMnO_4$/UV];

^{1}H NMR (400 MHz, $CDCl_3$) δ 7.35–7.28 (m, 4H, 4 × Ar*H*), 7.14–7.07 (m, 2H, Ar*H* + SC=C*H*), 6.37 (br. s, 1H, N*H*), 4.32 (quint, *J* = 2.9 Hz, 2H, C=CHCH_2), 3.72 (t, *J* = 5.6 Hz, 2H, $SCCH_2CH_2$), 2.68 (tq, *J* = 5.0, 2.2 Hz, 2H, $SCCH_2$);

^{13}C {^{1}H} NMR (101 MHz, $CDCl_3$) δ 154.6, 139.5 (d, $^3J_{CF}$ = 2.5 Hz), 138.3, 133.2, 129.2, 124.1, 120.6, 44.0, 40.3, 24.3;

^{19}F {^{1}H} NMR (377 MHz, $CDCl_3$) δ 51.6;

IR ν_{max} (neat)/cm^{-1} 3303, 2923, 2855, 1633, 1594, 1531, 1447, 1397, 1199;

HRMS (ESI^+) found *m/z* 285.0704 $[M+H]^+$, $C_{12}H_{14}FN_2O_3S^+$ requires *m/z* 285.0704.

3.5.13 Nucleophilic addition of alkenylsulfonyl fluoride 14 to phenyl isothiocyanate for the preparation of 1-(phenylcarbamothioyl)-1,2,3,6-tetrahydropyridine-4-sulfonyl fluoride (18)

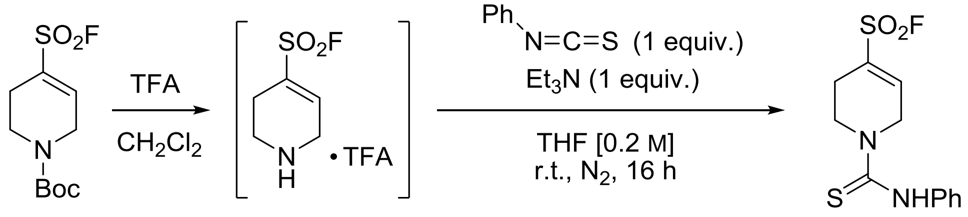

5p **14** **18** 84%

Following General Procedure E, alkenylsulfonyl fluoride **5p** (26.5 mg, 0.10 mmol, 1.0 equiv.) was deprotected and was added with a solution of phenyl isothiocyanate (12 µL, 0.10 mmol, 1.0 equiv.) and Et_3N (14 µL, 0.10 mmol, 1.0 equiv.) in anhydrous THF (0.5 mL). The reaction was stirred at ambient temperature for 16 h, which was then diluted with EtOAc, washed with water and brine, dried with anhydrous $MgSO_4$, filtered and concentrated *in vacuo*. The crude product was purified by flash column chromatography (30–50% EtOAc in petrol), affording the *alkenylsulfonyl fluoride* **18** as a white solid (25.1 mg, 84%);

mp 110 °C (decomp.) (CH_2Cl_2);

$\mathbf{R}_f$ 0.58 (50% EtOAc in hexane) [$KMnO_4$/UV];

^{1}**H NMR** (400 MHz, $CDCl_3$) δ 7.41–7.35 (m, 3H, N*H* + 2 × Ar*H*), 7.22 (tt, J = 7.4, 1.7 Hz, 1H, Ar*H*), 7.13 (d, J = 7.6 Hz, 2H, 2 × Ar*H*), 7.03–7.00 (m, 1H, SC=C*H*), 4.49 (quint, J = 2.9 Hz, 2H, C=CHCH_2), 4.02 (t, J = 5.6 Hz, 2H, $SCCH_2CH_2$), 2.73–2.68 (m, 2H, SCCH_2);

^{13}**C {^{1}H} NMR** (101 MHz, $CDCl_3$) δ 184.8, 139.6, 138.8, 132.9 (d, $^2J_{CF}$ = 26.4 Hz), 129.7, 126.1, 123.2, 49.1, 45.6, 23.8;

^{19}**F {^{1}H} NMR** (377 MHz, $CDCl_3$) δ 52.0;

IR ν_{max} (neat)/cm^{-1} 3303, 2923, 2855, 1633, 1594, 1531, 1447, 1397, 1199;

LRMS (ESI$^+$) *m/z* 301.1 ([M+H]$^+$);

HRMS (ESI$^+$) found *m/z* 301.0476 [M+H]$^+$, $C_{12}H_{14}FN_2O_2S_2^+$ requires *m/z* 301.0475.

3.5.14 Nucleophilic addition of alkenylsulfonyl fluoride 14 to fluorescein isothiocyanate (FITC) isomer I for the preparation of 5-(4-(fluorosulfonyl)-1,2,3,6-tetrahydropyridine-1-carbothioamido)-2-(6-hydroxy-3-oxo-3*H*-xanthen-9-yl)benzoic acid (19)

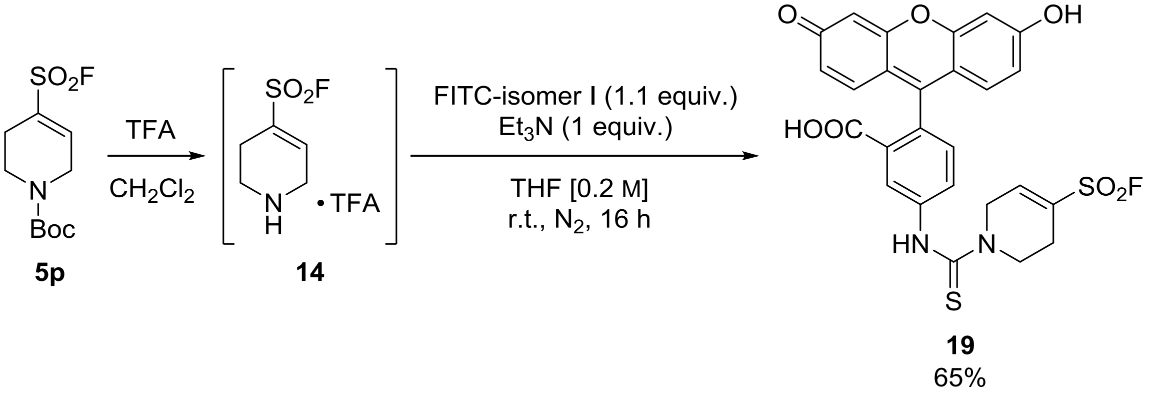

Following General Procedure E, alkenylsulfonyl fluoride **5p** (26.5 mg, 0.10 mmol, 1.0 equiv.) was deprotected and was added with FITC isomer 1 (42.8 mg, 0.11 mmol, 1.1 equiv.) and a solution of Et_3N (14 µL, 0.10 mmol, 1.0 equiv.) in anhydrous THF (0.5 mL). The reaction was stirred at ambient temperature for 16 h, which was then diluted with EtOAc and washed with water. The aqueous phase was extracted with EtOAc for three times, and the combined organic extract was washed with brine and dried with anhydrous $MgSO_4$, filtered and concentrated *in vacuo*. The crude product was purified by flash column chromatography (50–100% EtOAc in petrol), affording the *alkenylsulfonyl fluoride* **19** as an orange solid (36.0 mg, 65%);

mp 167–170 °C (decomp.) (EtOAc);

R_f 0.38 (75% EtOAc in hexane) [$KMnO_4$/UV];

^{1}H NMR (400 MHz, DMSO-d_6) δ 10.13 (br. s, 2H, 2 × O*H*), 9.88 (s, 1H, N*H*), 7.96 (d, *J* = 1.9 Hz, 1H, Ar*H*), 7.78 (dd, *J* = 8.3, 2.0 Hz, 1H, Ar*H*), 7.40–7.36 (m, 1H, SC=C*H*), 7.19 (d, *J* = 8.3 Hz, 1H, Ar*H*), 6.71–6.65 (m, 2H, 2 × Ar*H*), 6.61–6.55 (m, 4H, 4 × Ar*H*), 4.81 (t, *J* = 3.0 Hz, 2H, CHCH_2), 4.17 (t, *J* = 5.5 Hz, 2H, $SCCH_2CH_2$), 2.75–2.69 (m, 2H, SCCH_2);

^{13}C {^{1}H} NMR (101 MHz, DMSO-d_6) δ 182.2, 168.9, 159.9, 152.3, 148.5, 143.3, 142.9, 132.5, 130.6, 129.4, 126.6, 124.0, 119.6, 113.1, 110.1, 102.7, 83.5, 49.0, 44.6, 24.1;

^{19}F {^{1}H} NMR (377 MHz, DMSO-d_6) δ 52.7;

IR ν_{max} (neat)/cm^{-1} 3067, 2924, 2592, 1726, 1589, 1452, 1397, 1203, 1175, 1111;

LRMS (ESI$^+$) *m*/*z* 555.0 ([M+H]$^+$);

HRMS (ESI$^+$) found *m*/*z* 555.0691 [M+H]$^+$, $C_{26}H_{20}FN_2O_7S_2^+$ requires *m*/*z* 555.0690.

3.5.15 Amide coupling between alkenylsulfonyl fluoride 14 and amino acid using T3P for the preparation of *tert*-butyl (*S*)-(1-(4-(fluorosulfonyl)-3,6-dihydropyridin-1(2*H*)-yl)-1-oxo-3-phenylpropan-2-yl)carbamate (20)

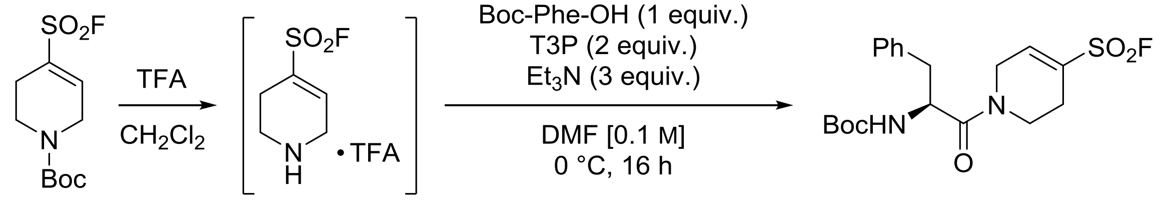

5p **14** **20**
75%

Following General Procedure E, alkenylsulfonyl fluoride **5p** (39.8 mg, 0.15 mmol, 1.0 equiv.) was deprotected and was added subsequently with anhydrous DMF (1.5 mL), Et_3N (63 μL, 0.45 mmol, 3.0 equiv.) and Boc-Phe-OH (39.8 mg, 0.15 mmol, 1.0 equiv.). The mixture was then cooled to 0 °C and T3P (50 wt% in EtOAc) (0.18 mL, 0.30 mmol, 2.0 equiv.) was slowly added. The reaction was stirred at 0 °C for 16 h, which was then diluted with EtOAc and washed with water and brine, dried with anhydrous $MgSO_4$, filtered and concentrated *in vacuo*. The crude product was purified by flash column chromatography (30–60% Et_2O in petrol), affording the *alkenylsulfonyl fluoride* **20** as a white solid (46.2 mg, 75%, >99% ee);

mp 59–61 °C ($CHCl_3$);

R*f* 0.33 (67% Et_2O in hexane) [$KMnO_4$/UV];

$[\alpha]_D^{25}$ +4.2° (c = 1.0, $CHCl_3$)

^{1}H NMR (400 MHz, $CDCl_3$) δ 7.32–7.21 (m, 3H, 3 × Ar*H*), 7.21–7.14 (m, 2H, 2 × Ar*H*), 6.97 (br. s, 0.6H, SC=CH^{rot1}), 6.55 (s, 0.4H, SC=CH^{rot2}), 5.42 (d, *J* = 8.6 Hz, 0.4H, NH^{rot2}), 5.35 (d, *J* = 9.0 Hz, 0.6H, NH^{rot2}), 4.84 (td, *J* = 9.2, 5.4 Hz, 0.6H, C(=O)CH^{rot1}), 4.75 (td, *J* = 9.7, 5.3 Hz, 0.4H, C(=O)CH^{rot2}), 4.38–4.18 (m, 1.2H, C=CHCH_2^{rot1}), 4.07–3.96 (m, 0.8H, C=CHCH_aH$_b^{rot2}$ + NCH_cH$_d$CH$_2^{rot2}$), 3.55–3.45 (m, 0.4H, C=CHCH$_a$$H_b^{rot2}$), 3.47–3.32 (m, 1H, NCH$_c$$H_dCH_2^{rot2}$ + NCH_cH$_d$CH$_2^{rot1}$), 3.27 (dt, *J* = 13.9, 5.4 Hz, 0.6H, NCH$_c$$H_dCH_2^{rot1}$), 3.10 (dd, *J* = 12.9, 5.1 Hz, 0.4H, PhCH_eH$_f^{rot2}$), 3.04 (dd, *J* = 13.0, 5.6 Hz, 0.6H, PhCH_eH$_f^{rot1}$), 2.96 (dd, *J* = 12.9, 9.7 Hz, 0.6H, PhCH$_e$$H_f^{rot1}$), 2.89 (dd, *J* = 12.9, 10.3 Hz, 0.4H, PhCH$_e$$H_f^{rot2}$), 2.51–2.38 (m, 0.8H, SCCH_2^{rot2}), 2.38–2.29 (m, 0.6H, SCCH_gH$_h^{rot1}$), 1.67 (dd, *J* = 15.1, 7.1 Hz, 0.6H, SCCH$_g$$H_h^{rot1}$), 1.44 (s, 3.6H, C(C$H_3$)$_3^{rot2}$), 1.43 (s, 5.4H, C(C$H_3$)$_3^{rot1}$) (recorded as a pair of rotamers);

^{13}C {^{1}H} NMR (101 MHz, $CDCl_3$) δ {171.4, 171.0}, 155.2, 139.3, 138.3, {136.3, 136.0}, 129.4, {129.0, 128.9}, {127.7, 127.6}, 80.3, {52.0, 51.5}, {44.5, 42.1}, {41.6, 37.9}, {40.9, 40.6}, 28.5, {24.2, 23.6}(recorded as a pair of rotamers);

^{19}F {^{1}H} NMR (377 MHz, $CDCl_3$) δ 51.9, 51.1 (recorded as a pair of rotamers);

IR ν_{max} (neat)/cm^{-1} 3420, 3313, 3029, 2929, 1705, 1641, 1497, 1454, 1405, 1367, 1204, 1168;

LRMS (ESI$^+$) *m*/*z* 413.2 ([M+H]$^+$);

HRMS (ESI$^+$) found *m*/*z* 435.1359 [M+Na]$^+$, $C_{19}H_{25}FN_2O_5SNa^+$ requires *m*/*z* 435.1360;

The **ee** was determined by HPLC using a Chiralpak IC column (90:10 hexane/*i*-PrOH); flow rate 1 mL/min; τ_{major} = 19.72 min, τ_{minor} = 37.49 min (>99:1 er, >99% ee).

4 NMR Spectra of Alkenyl Triflates

1-Cyclohept-1-enyl trifluoromethanesulfonate (3a)

^{1}H-NMR Spectrum:

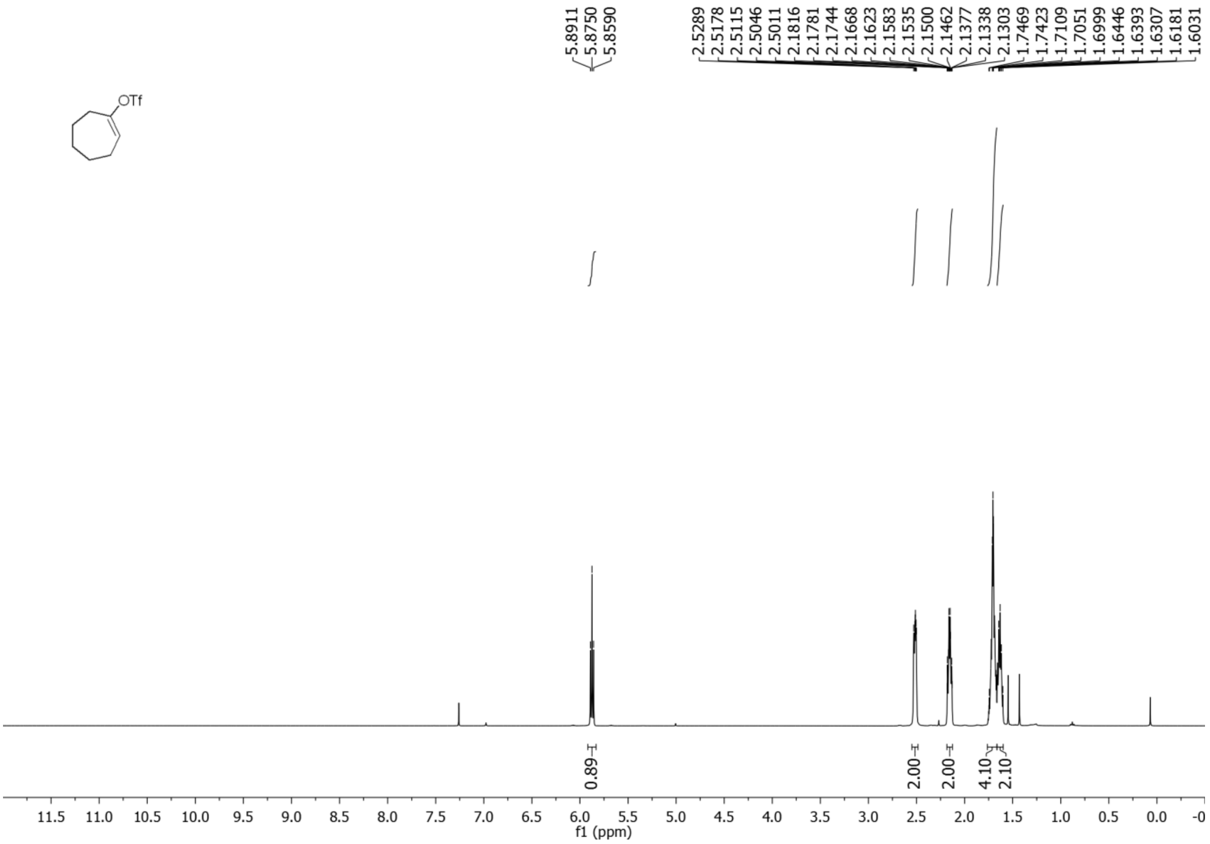

^{13}C-NMR Spectrum:

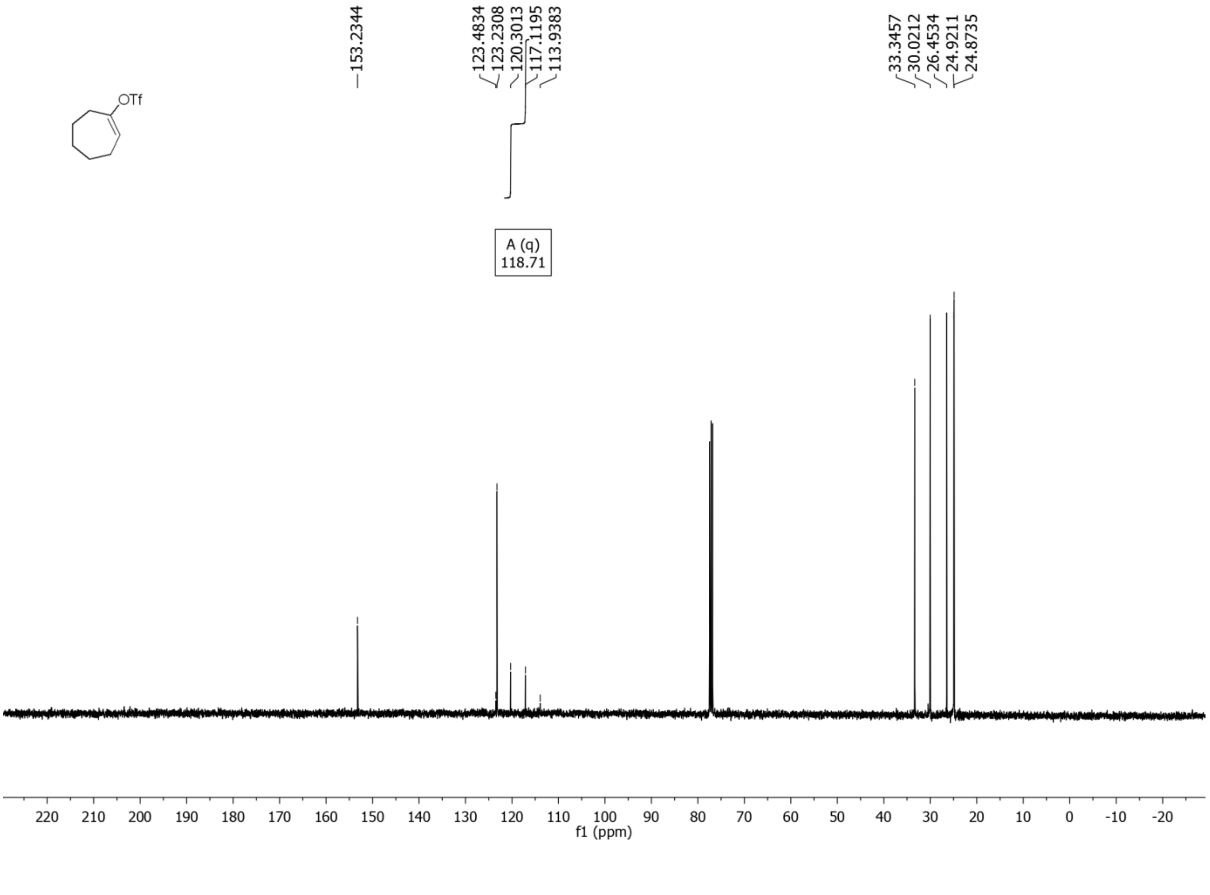

^{19}F-NMR Spectrum:

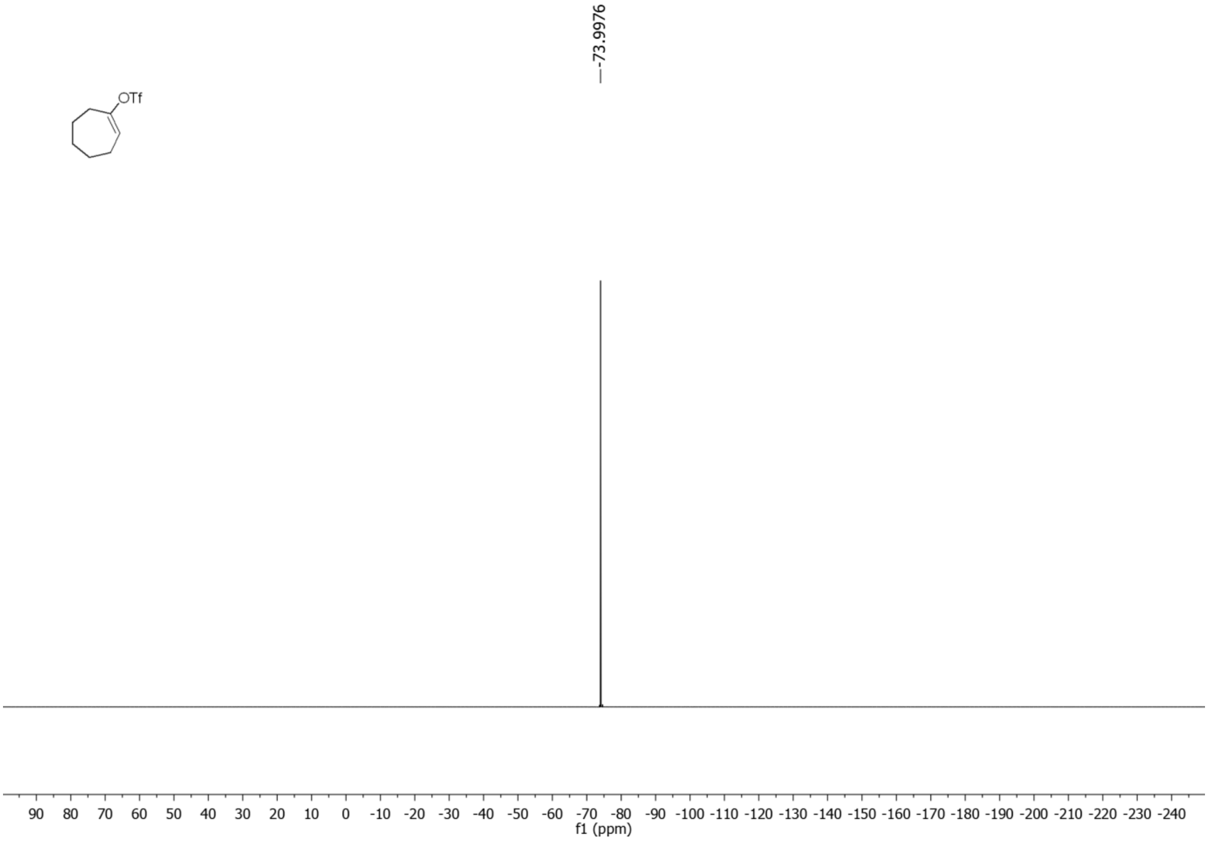

4-Phenyl-1-cyclohexen-1-yl trifluoromethanesulfonate (3b)

^{1}H-NMR Spectrum:

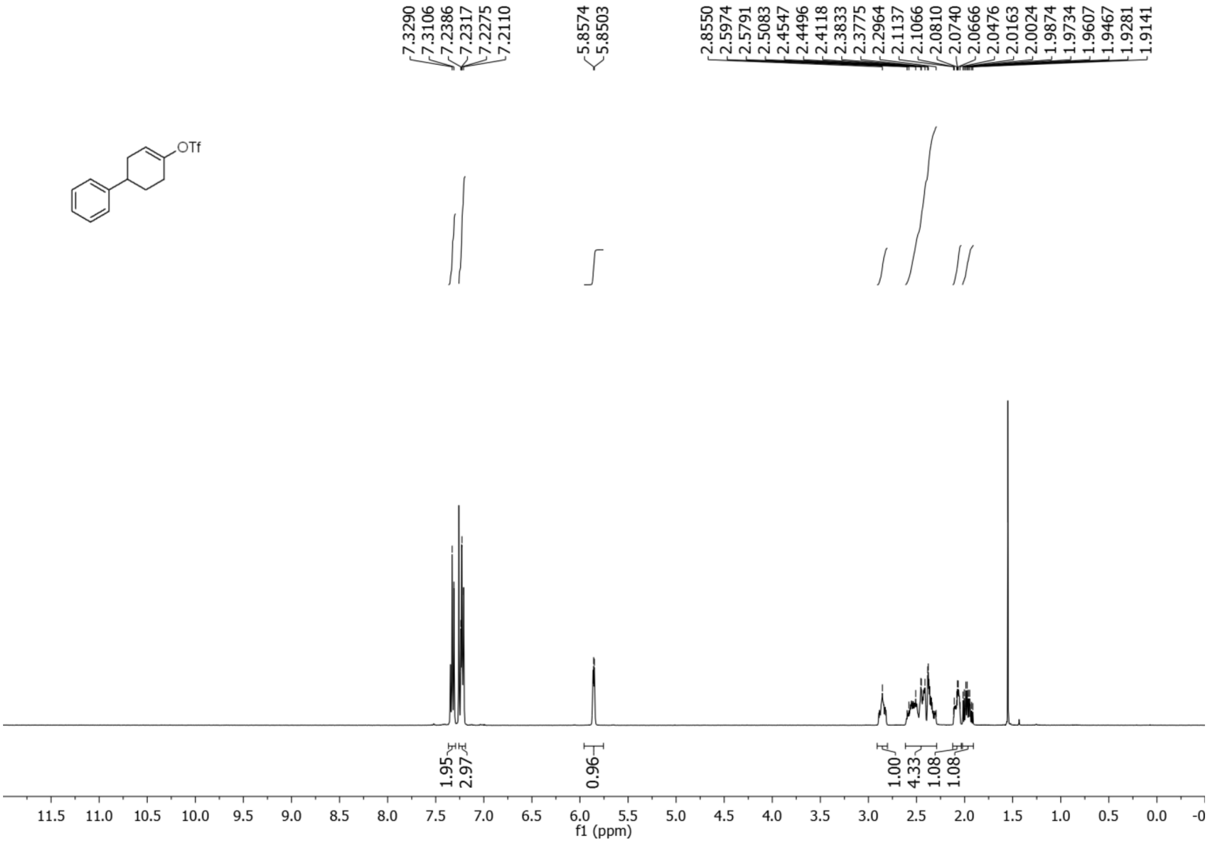

^{13}C-NMR Spectrum:

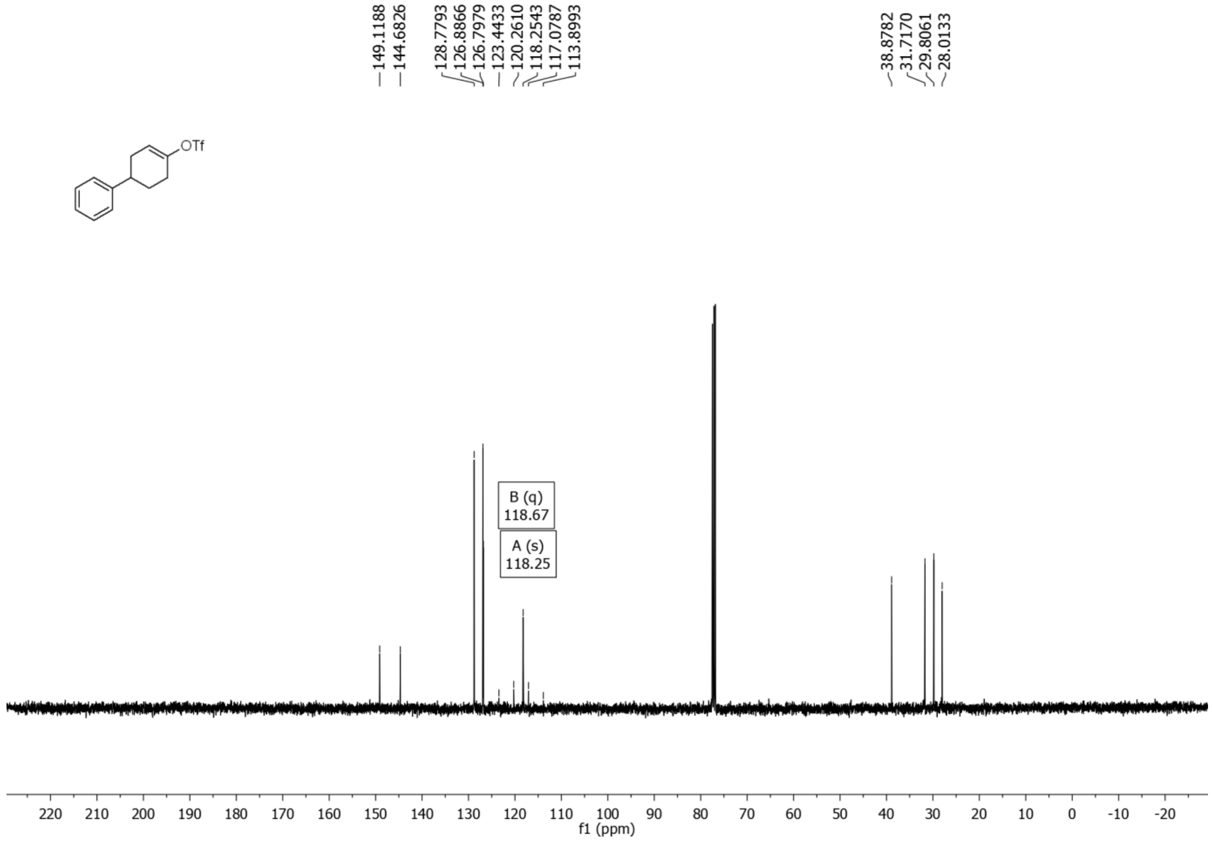

^{19}F-NMR Spectrum:

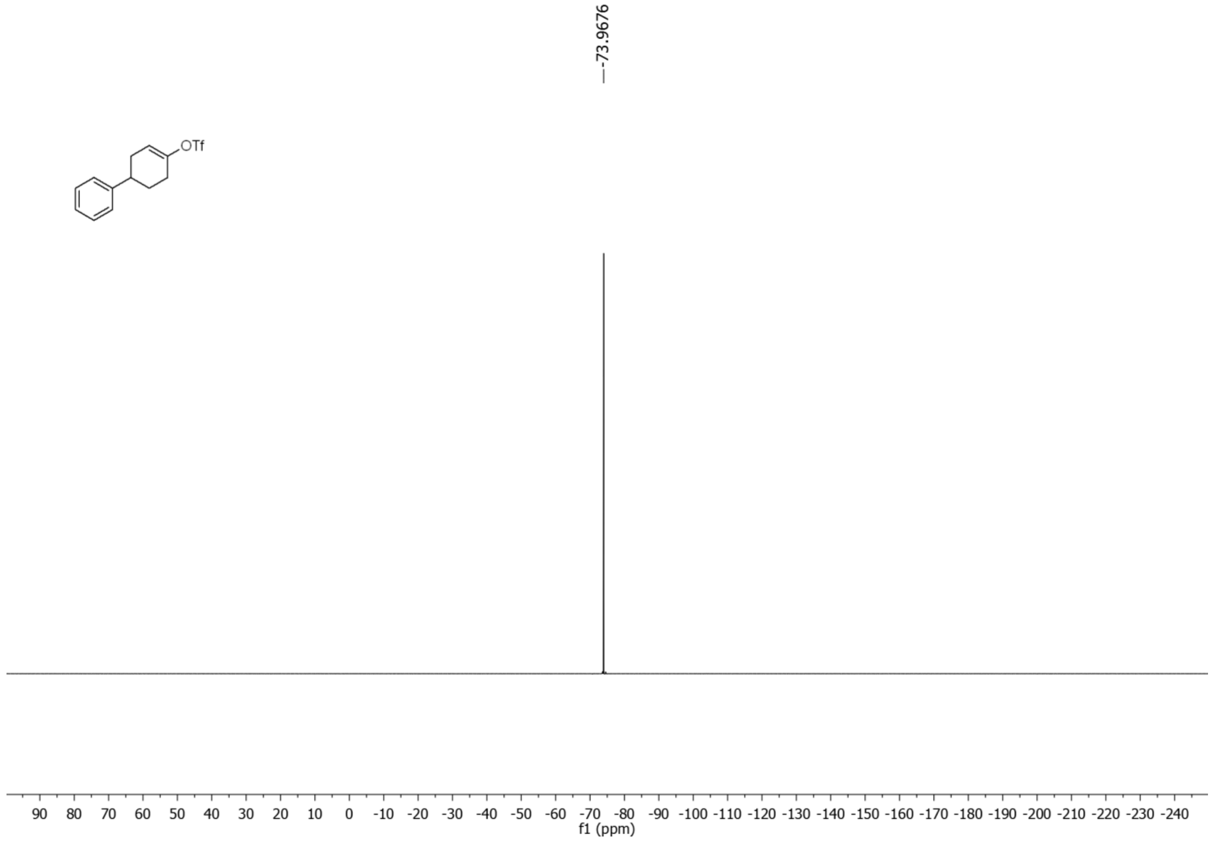

4-(*tert*-Butyl)cyclohex-1-en-1-yl trifluoromethanesulfonate (3c)

^{1}H-NMR Spectrum:

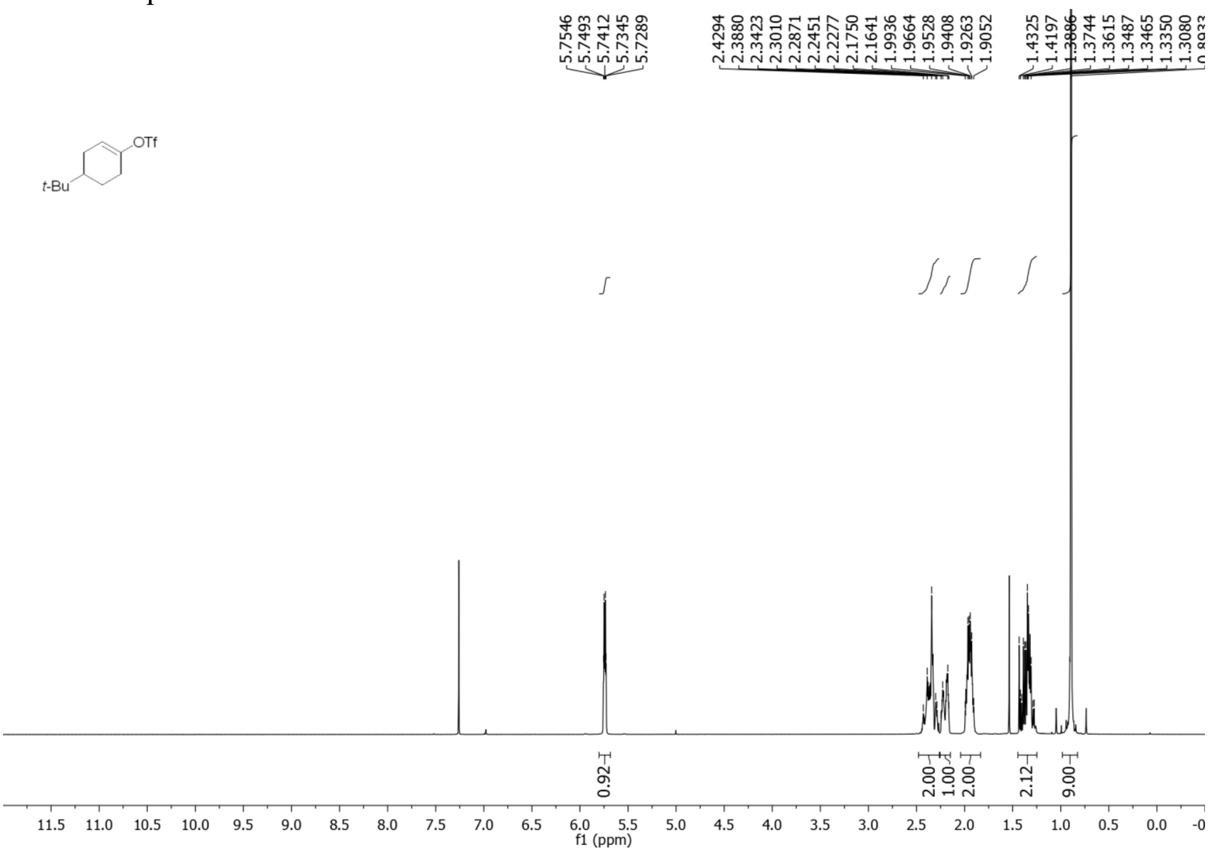

^{13}C-NMR Spectrum:

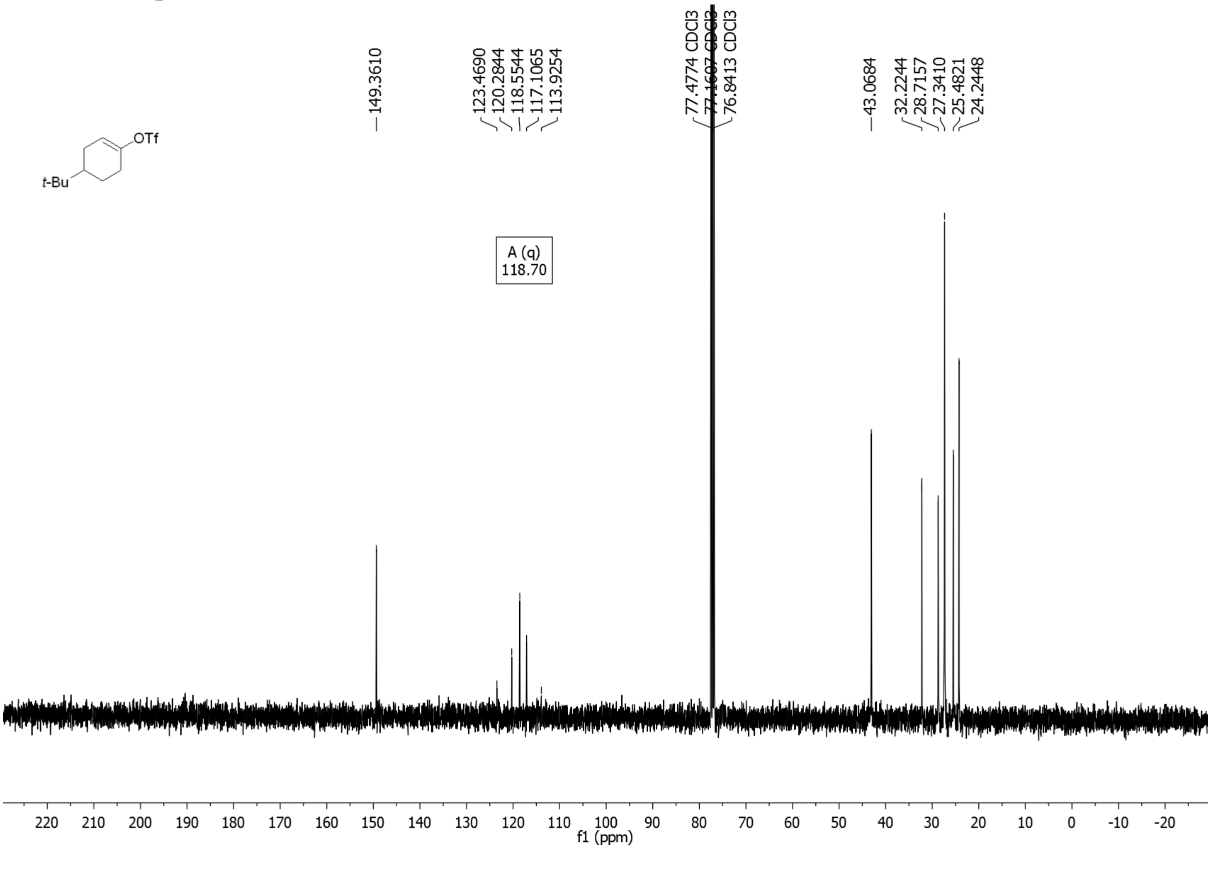

^{19}F-NMR Spectrum:

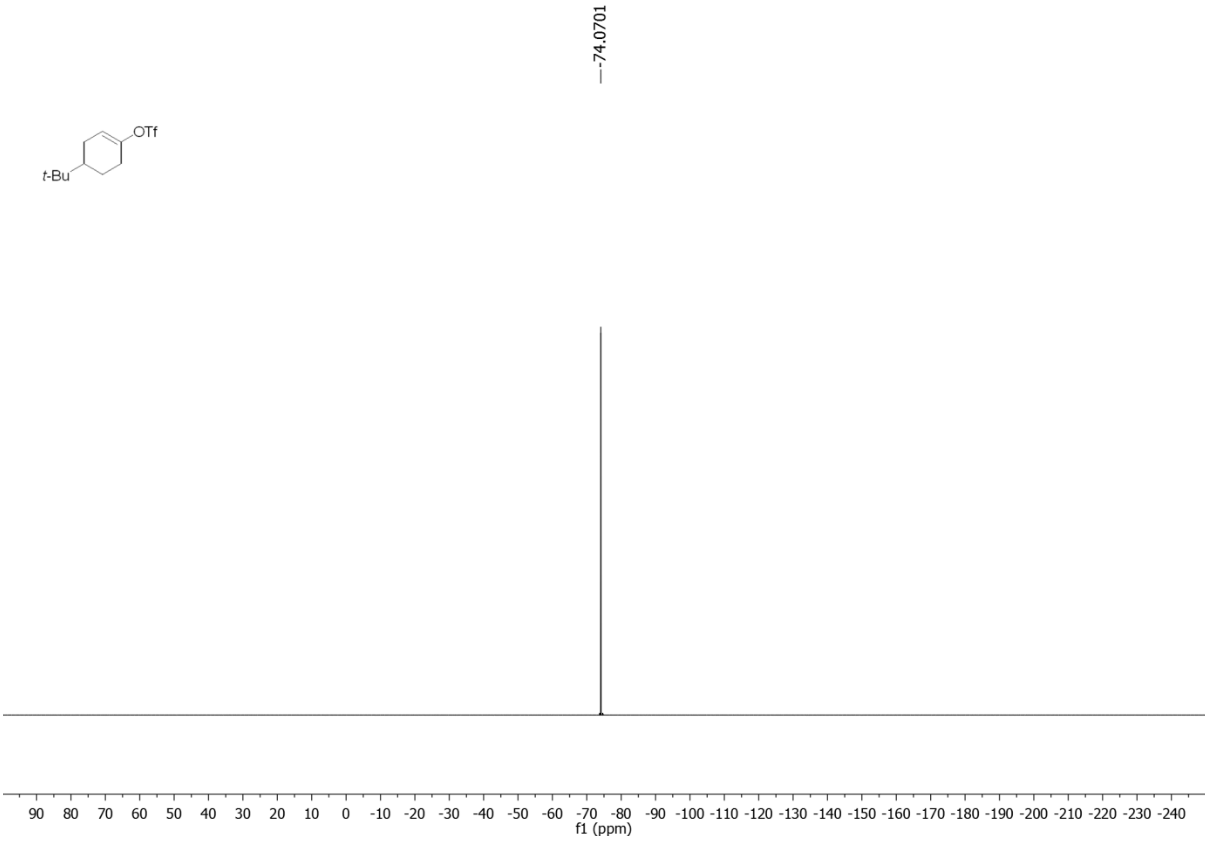

4,4-Difluorocyclohex-1-en-1-yl trifluoromethanesulfonate (3d)

^{1}H-NMR Spectrum:

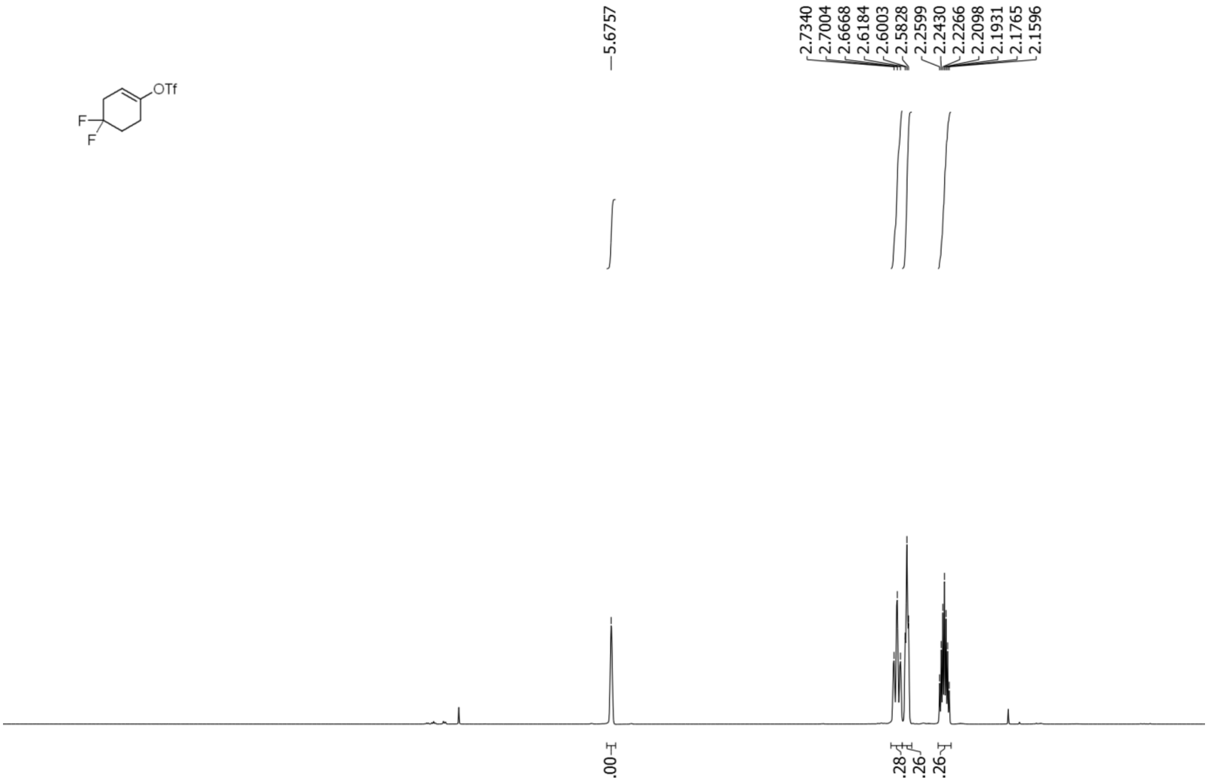

^{13}C-NMR Spectrum:

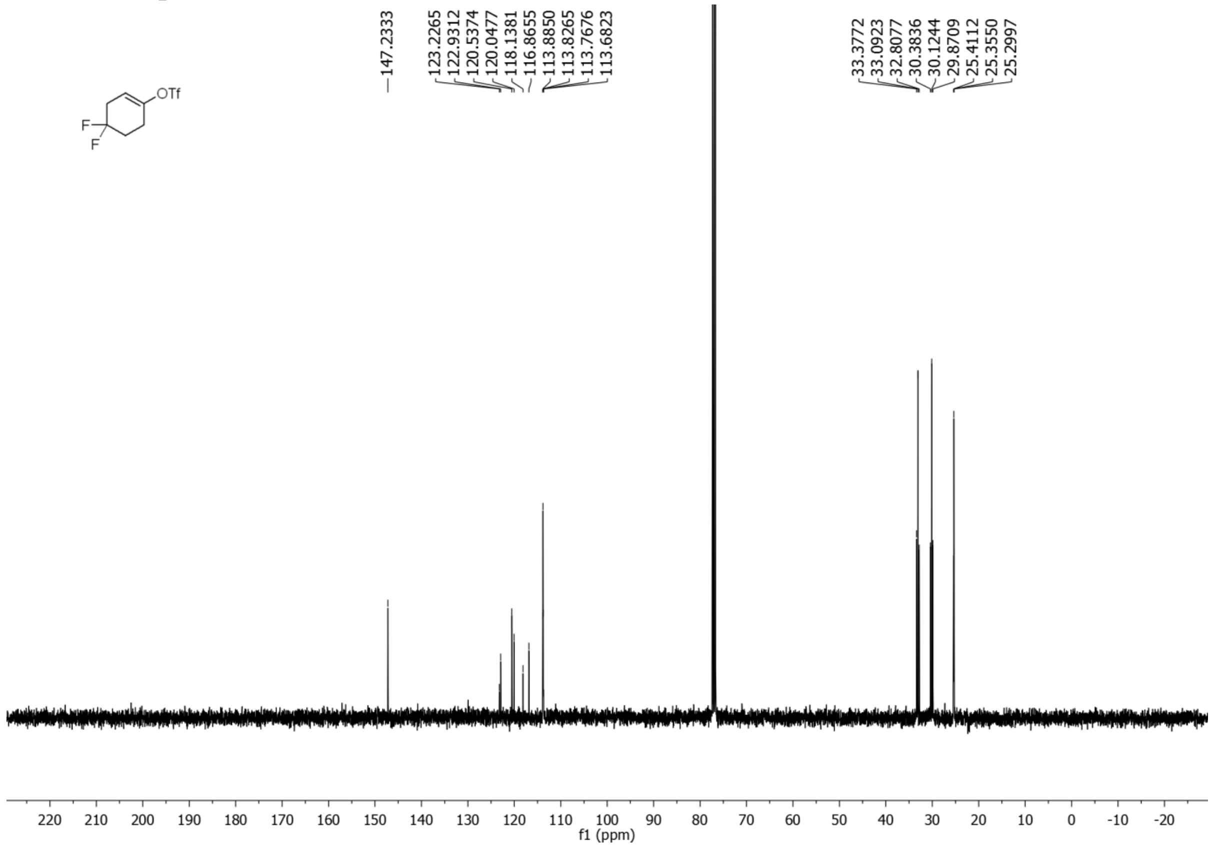

^{19}F-NMR Spectrum:

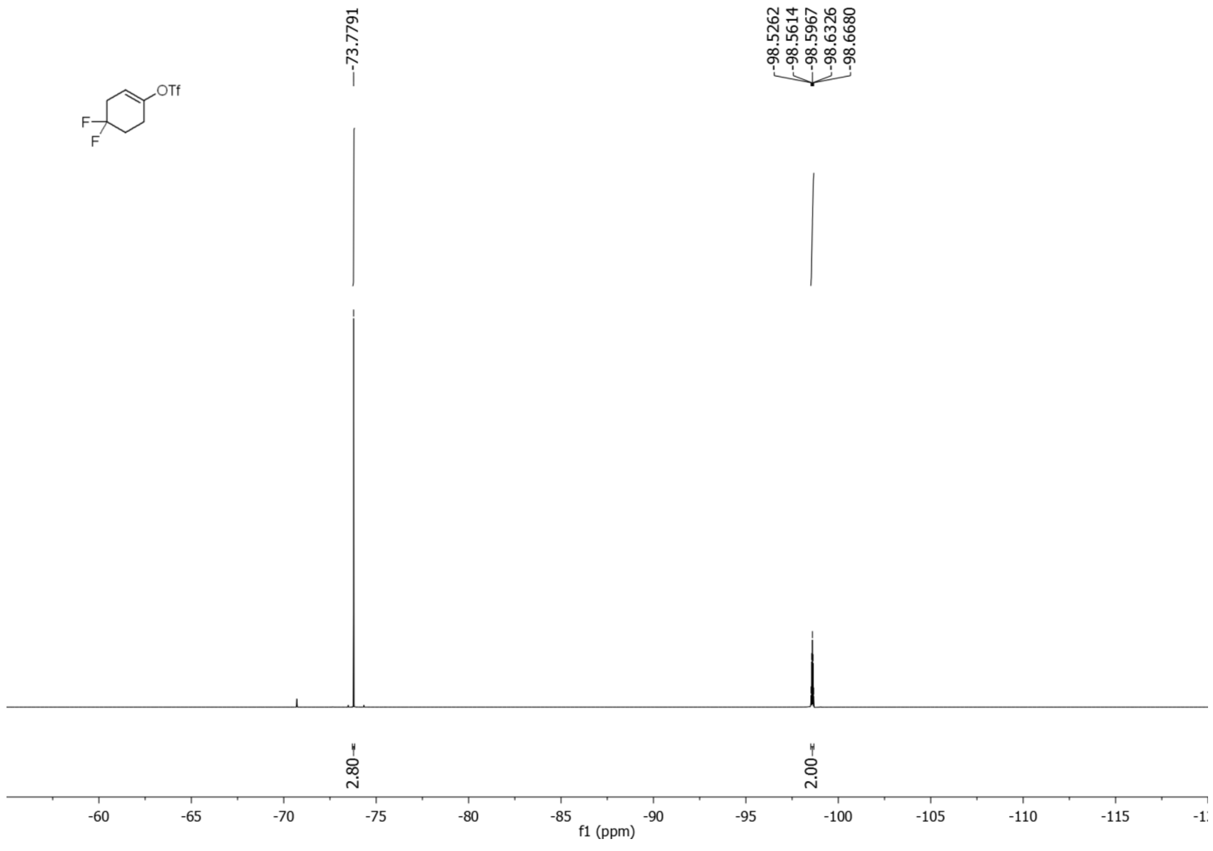

Ethyl 4-(((trifluoromethyl)sulfonyl)oxy)cyclohex-3-ene-1-carboxylate (3e)

^{1}H-NMR Spectrum:

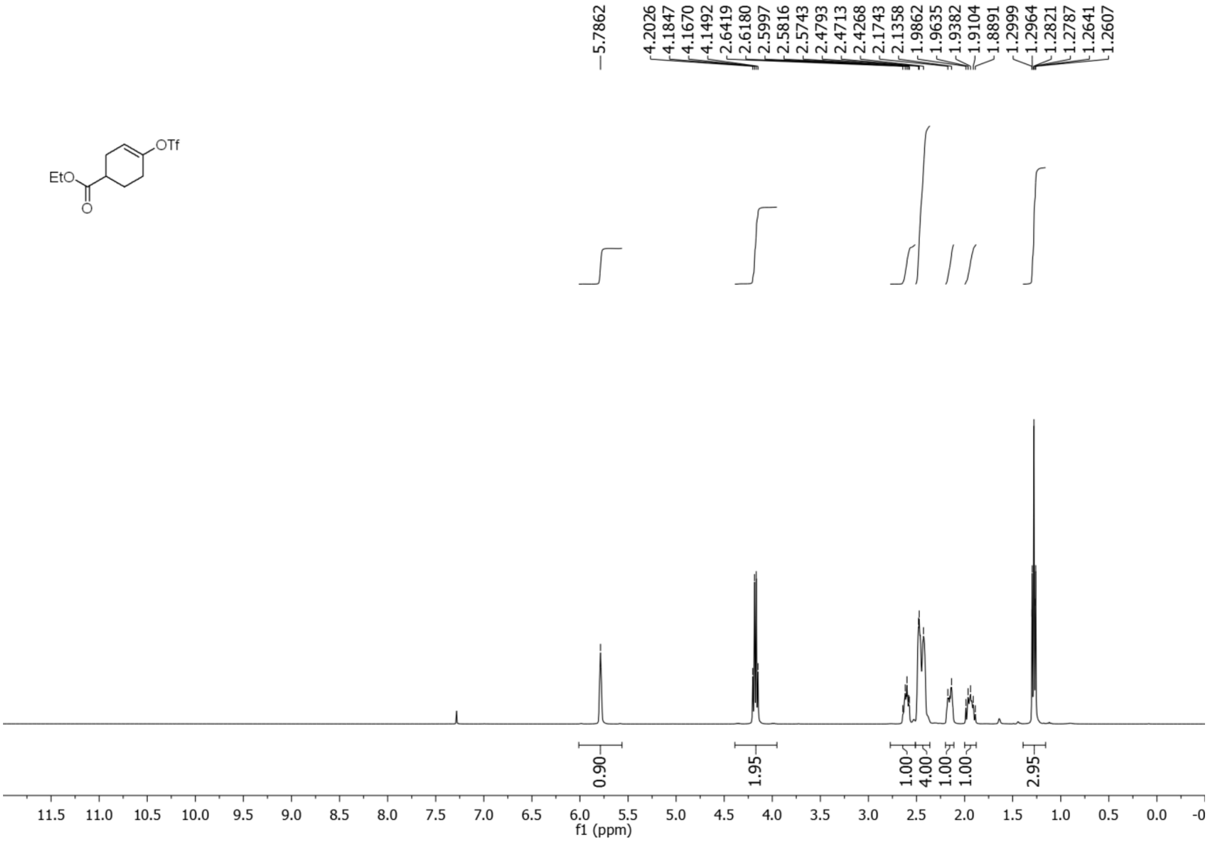

^{13}C-NMR Spectrum:

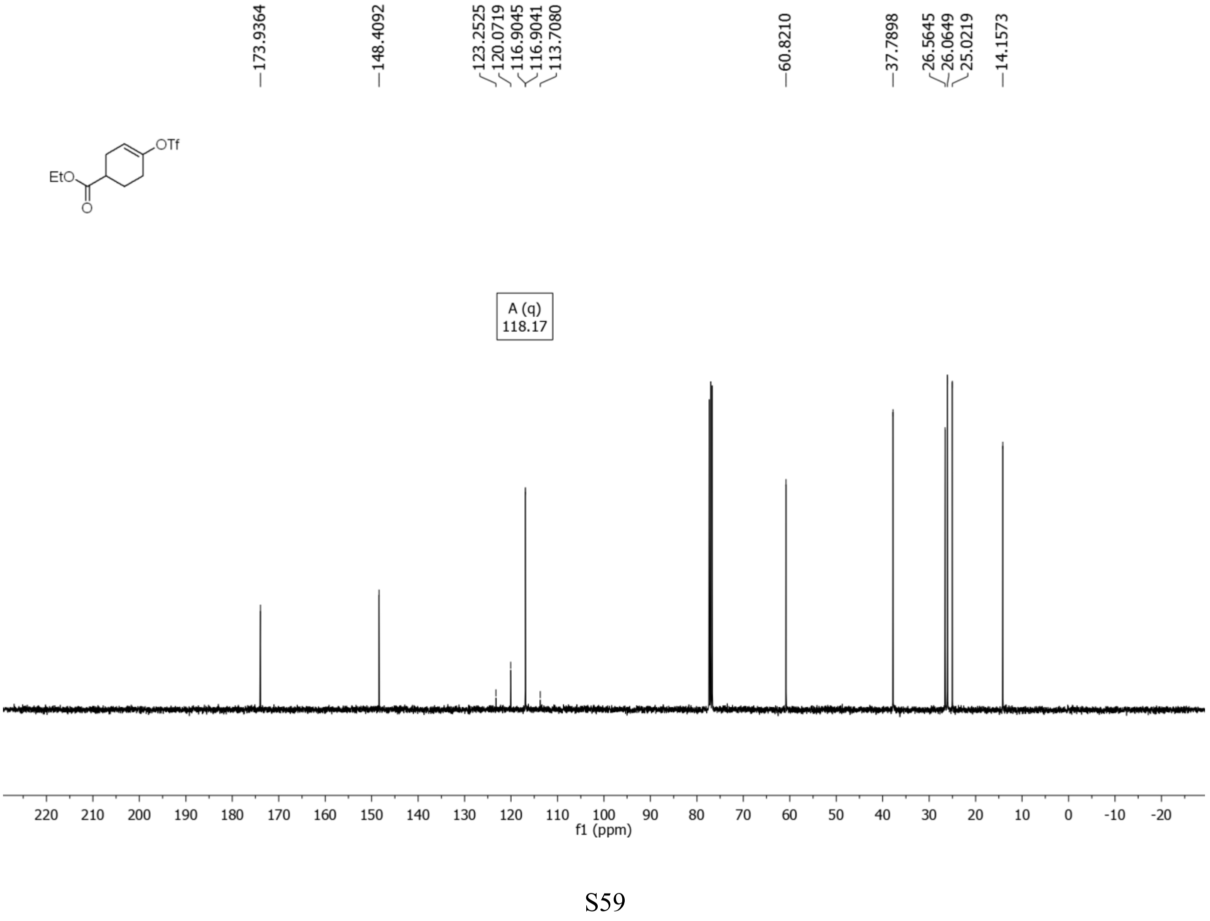

^{19}F-NMR Spectrum:

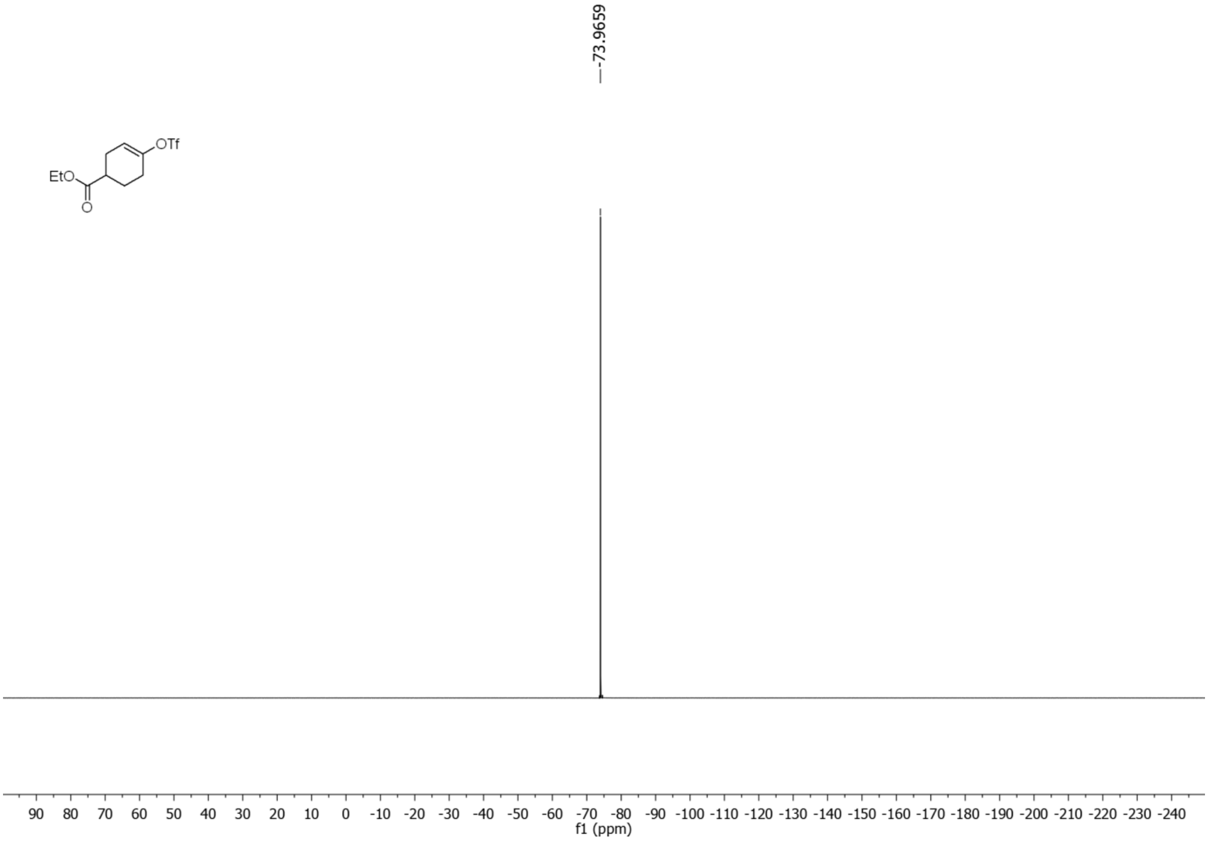

4-Methoxycyclohex-1-en-1-yl trifluoromethanesulfonate (3f)

^{1}H-NMR Spectrum:

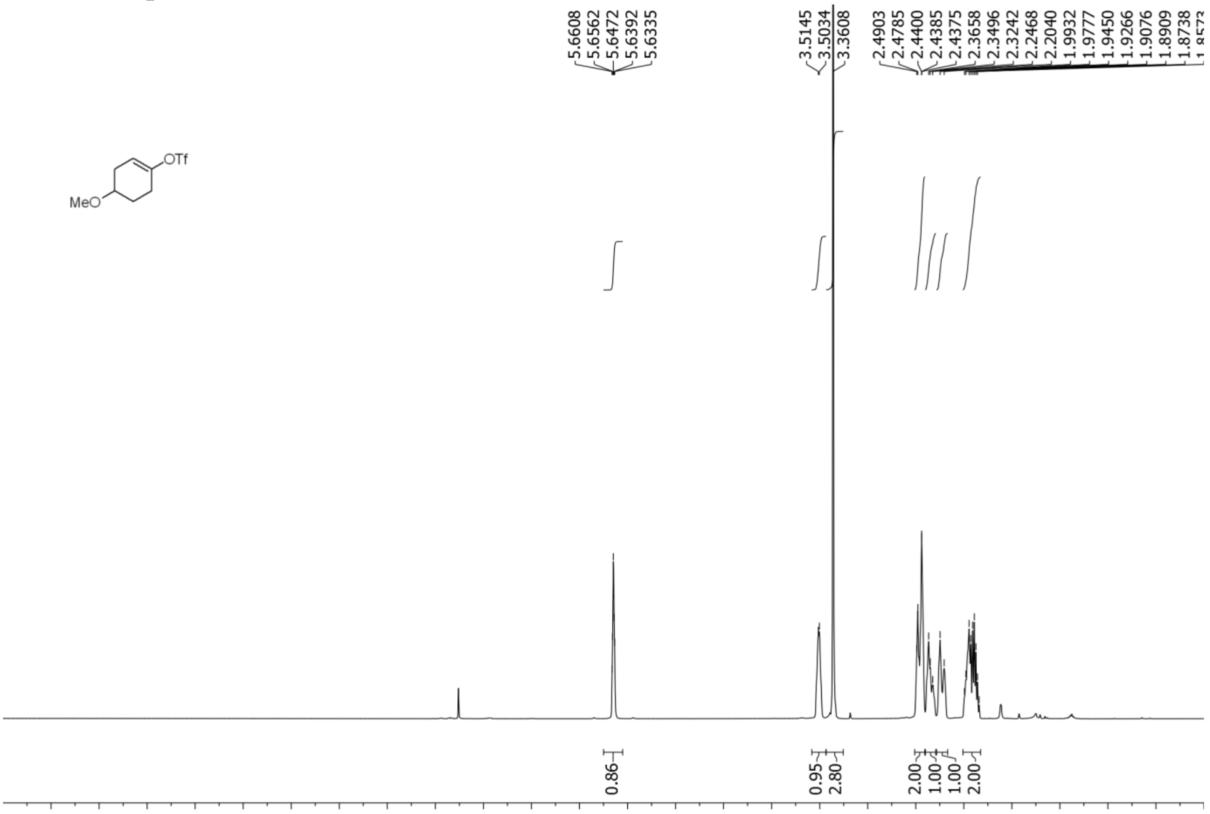

^{13}C-NMR Spectrum:

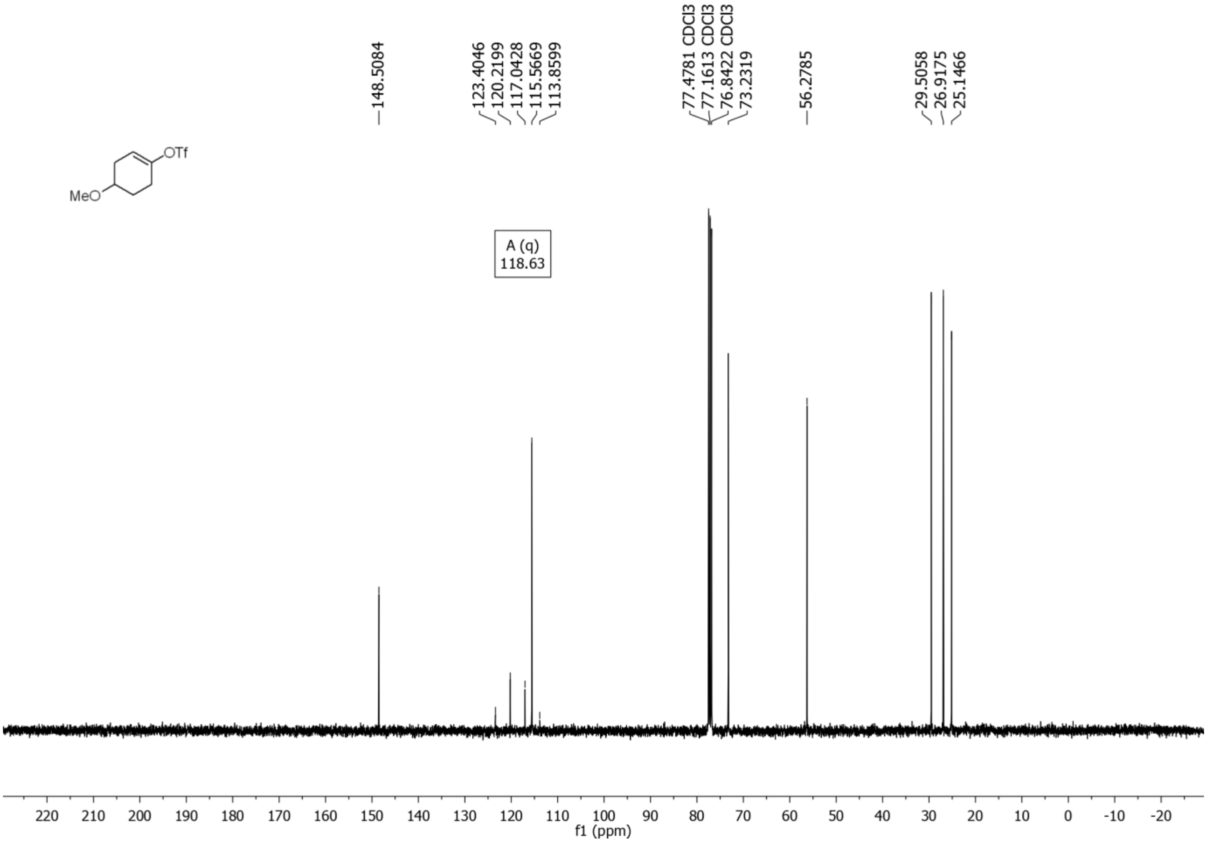

^{19}F-NMR Spectrum:

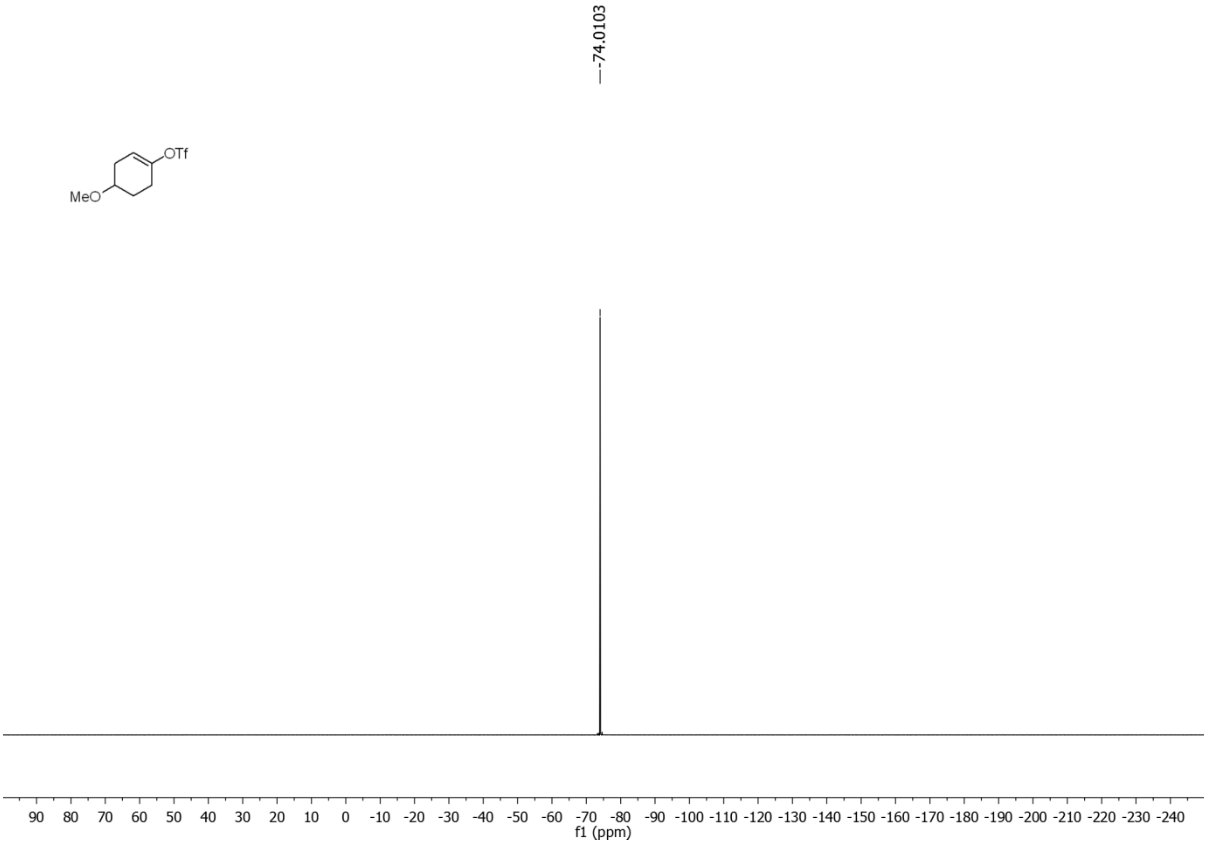

4-((*tert*-Butoxycarbonyl)amino)cyclohex-1-en-1-yl trifluoromethanesulfonate (3g)

^{1}H-NMR Spectrum:

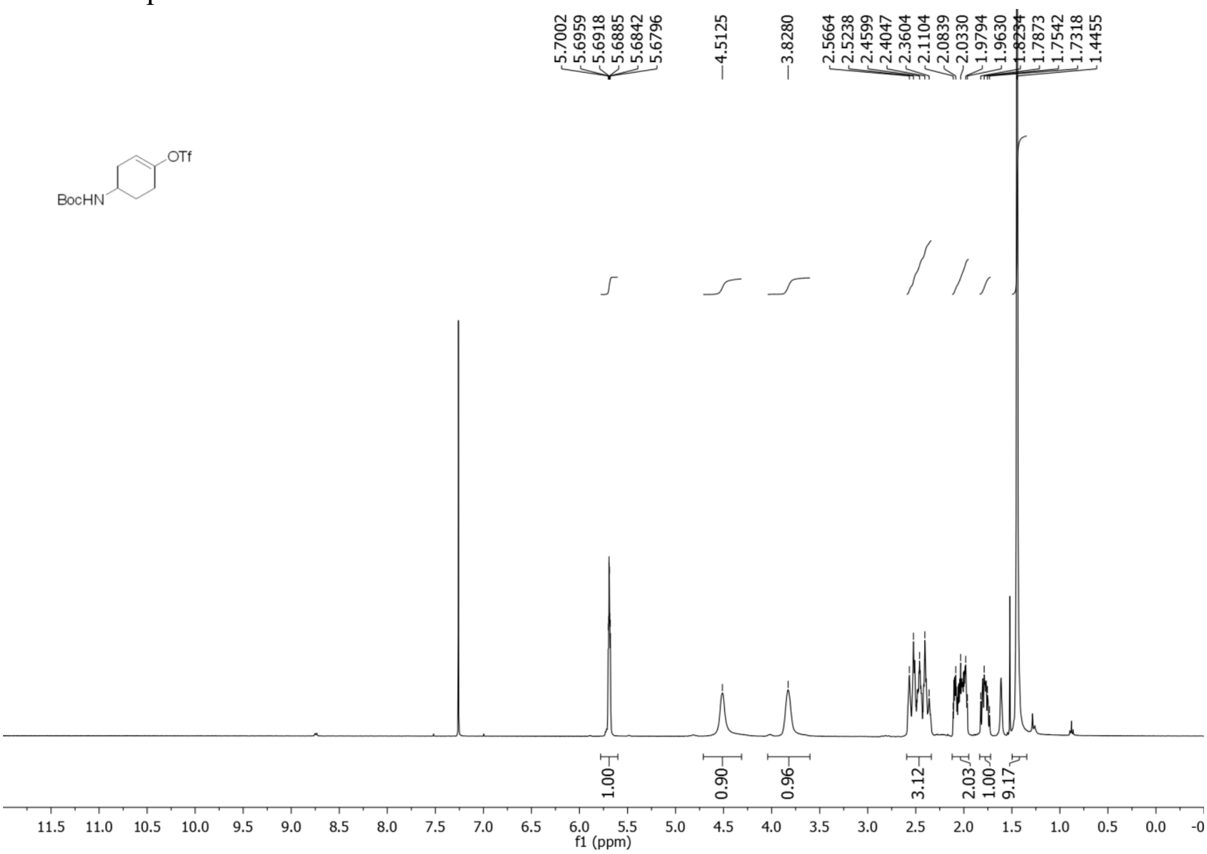

^{13}C-NMR Spectrum:

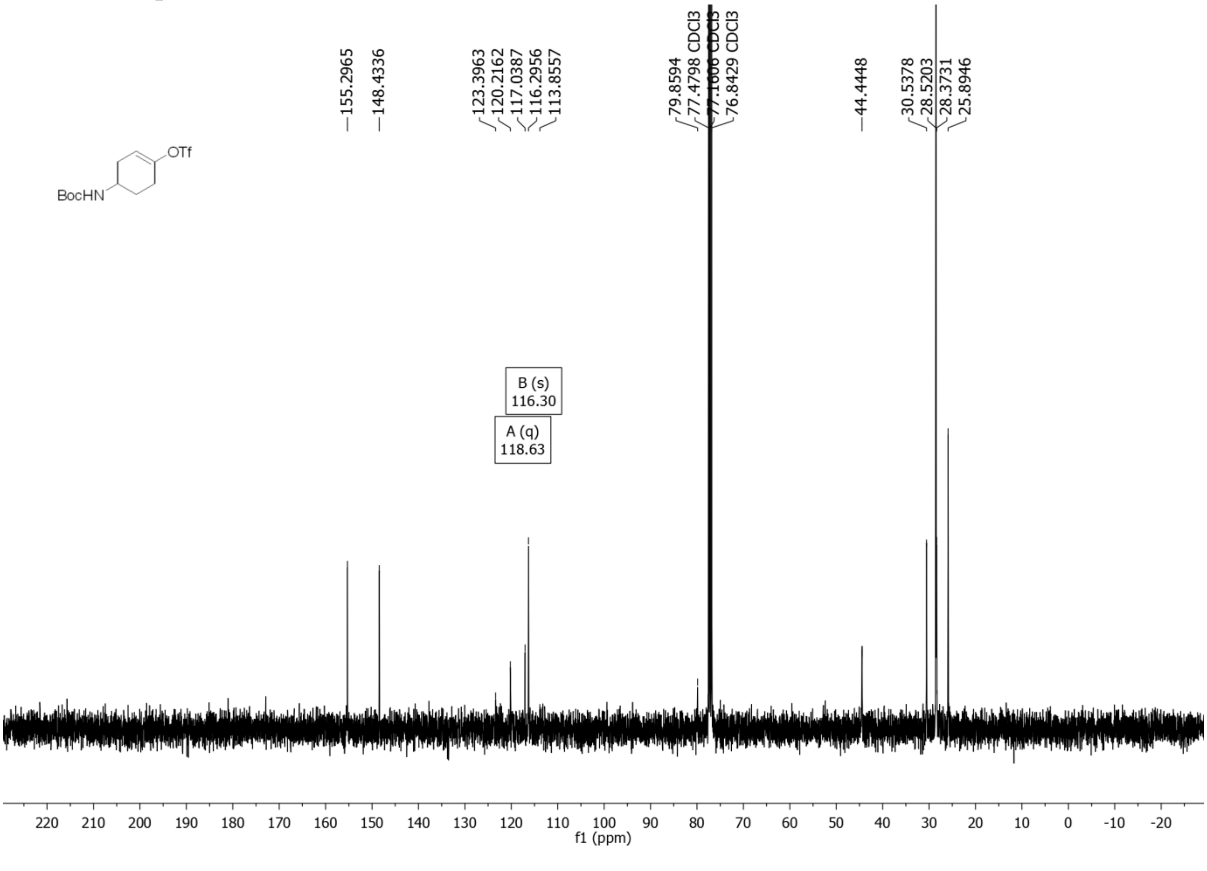

^{19}F-NMR Spectrum:

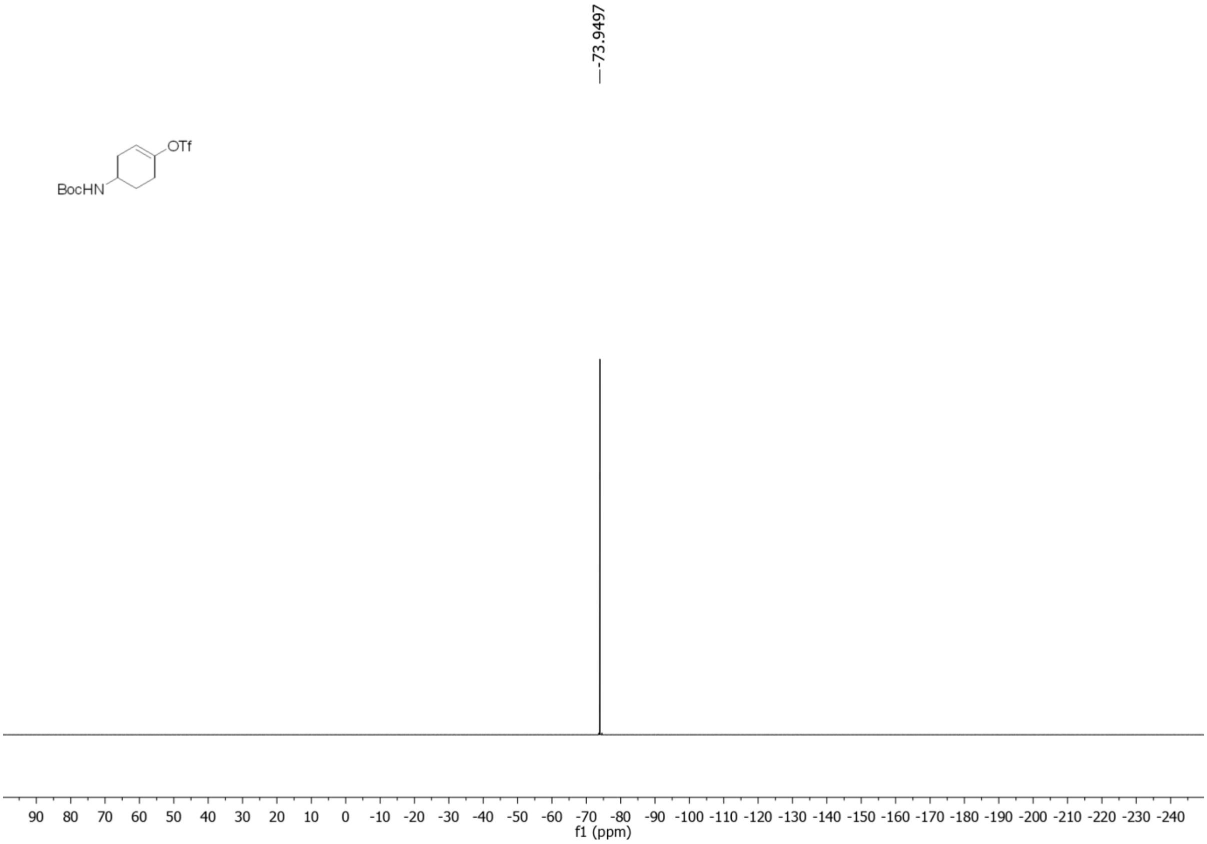

1,4-Dioxaspiro[4.5]dec-7-en-8-yl trifluoromethanesulfonate (3h)

^{1}H-NMR Spectrum:

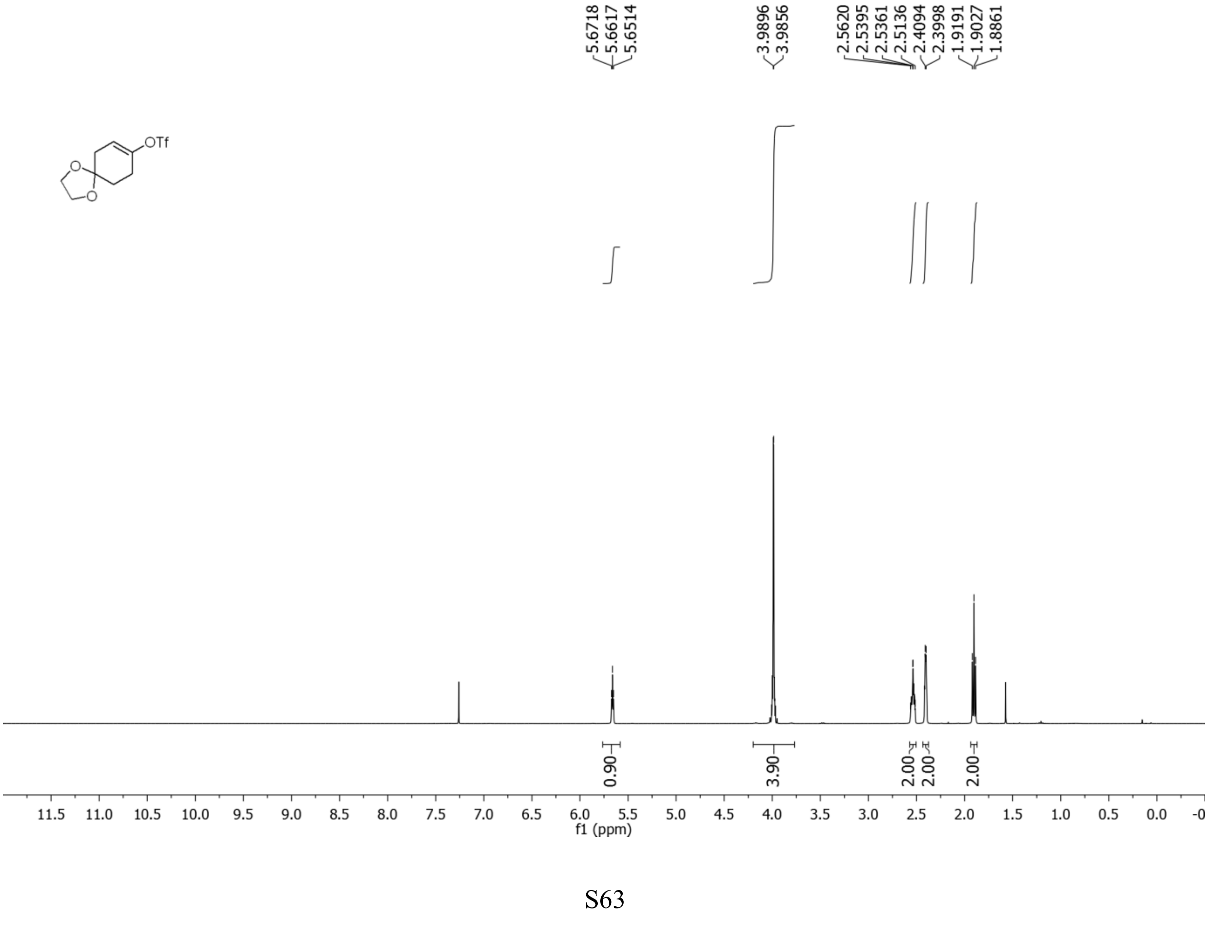

^{13}C-NMR Spectrum:

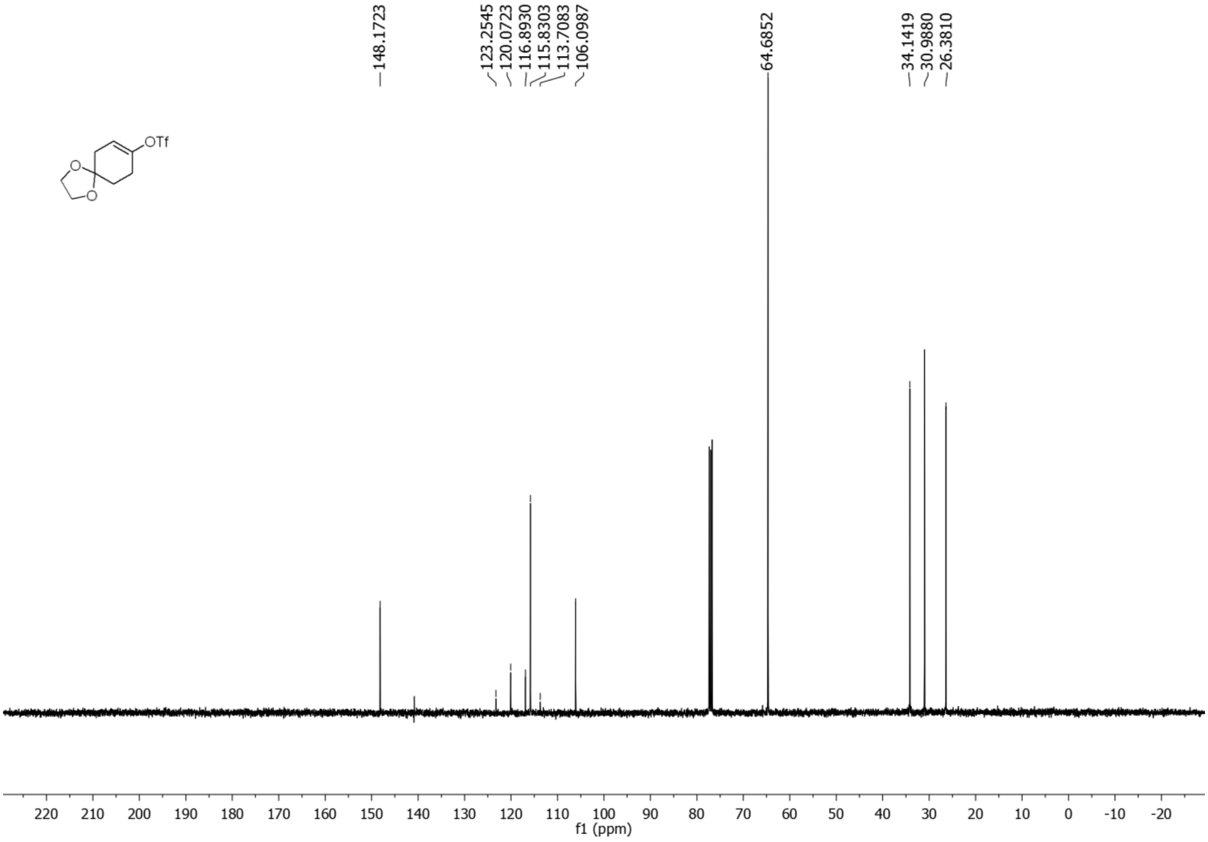

^{19}F-NMR Spectrum:

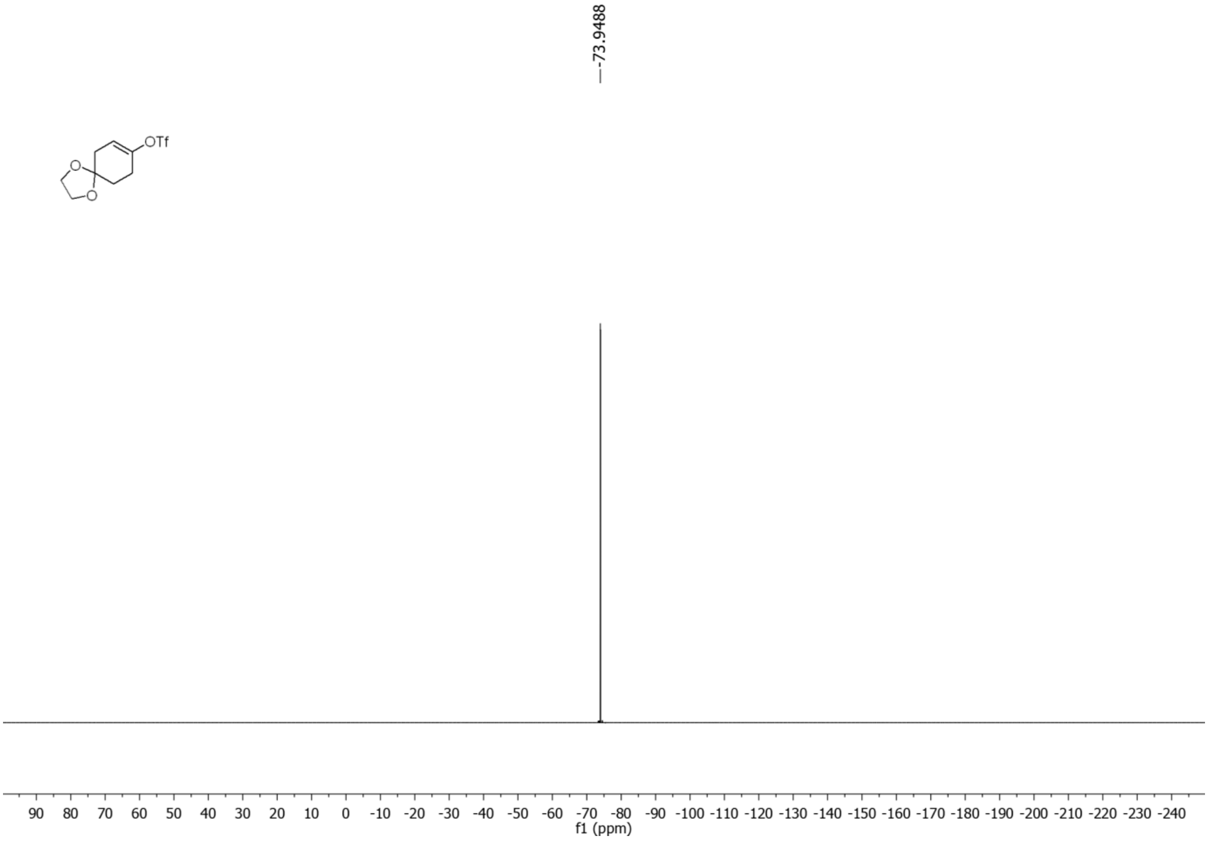

3-(1-Methyl-1*H*-indol-3-yl)cyclohex-1-en-1-yl trifluoromethanesulfonate (3i)

^{1}H-NMR Spectrum:

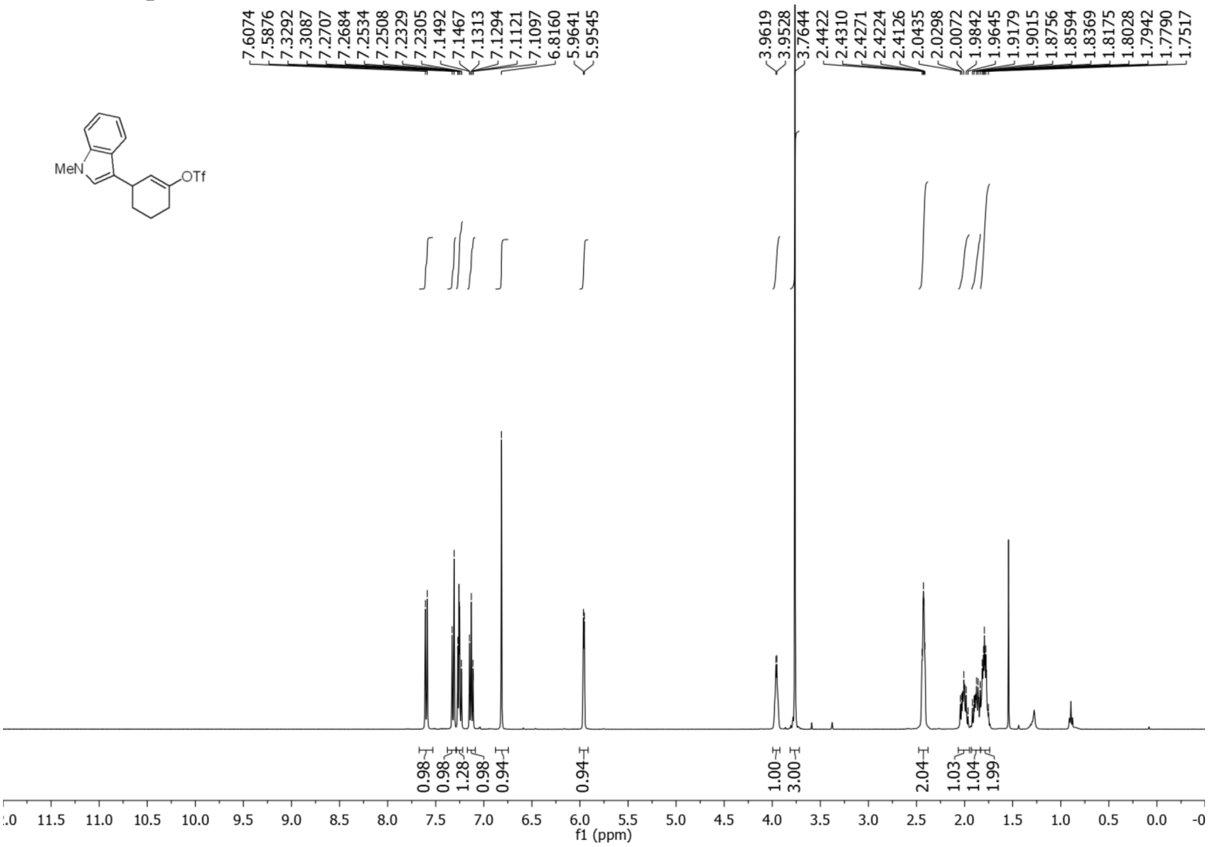

^{13}C-NMR Spectrum:

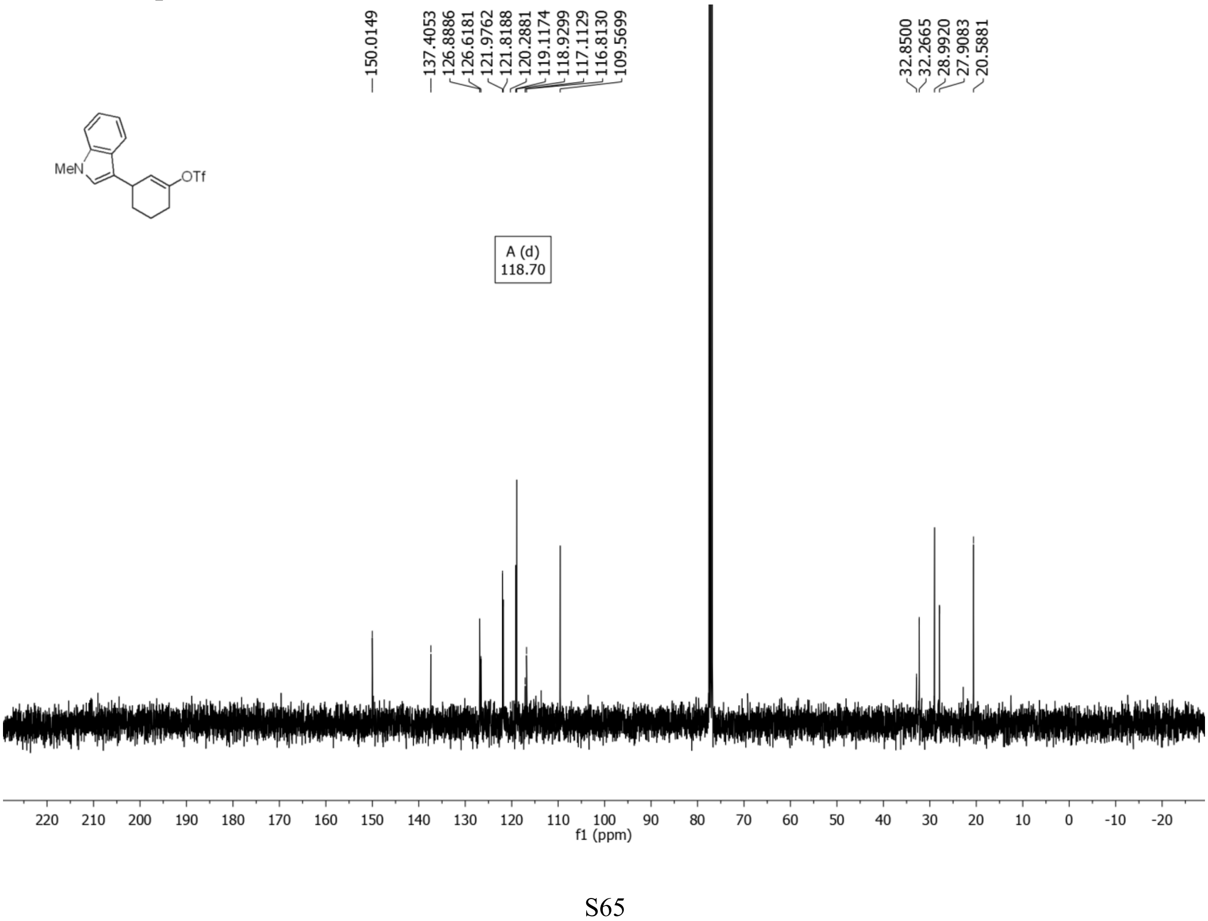

^{19}F-NMR Spectrum:

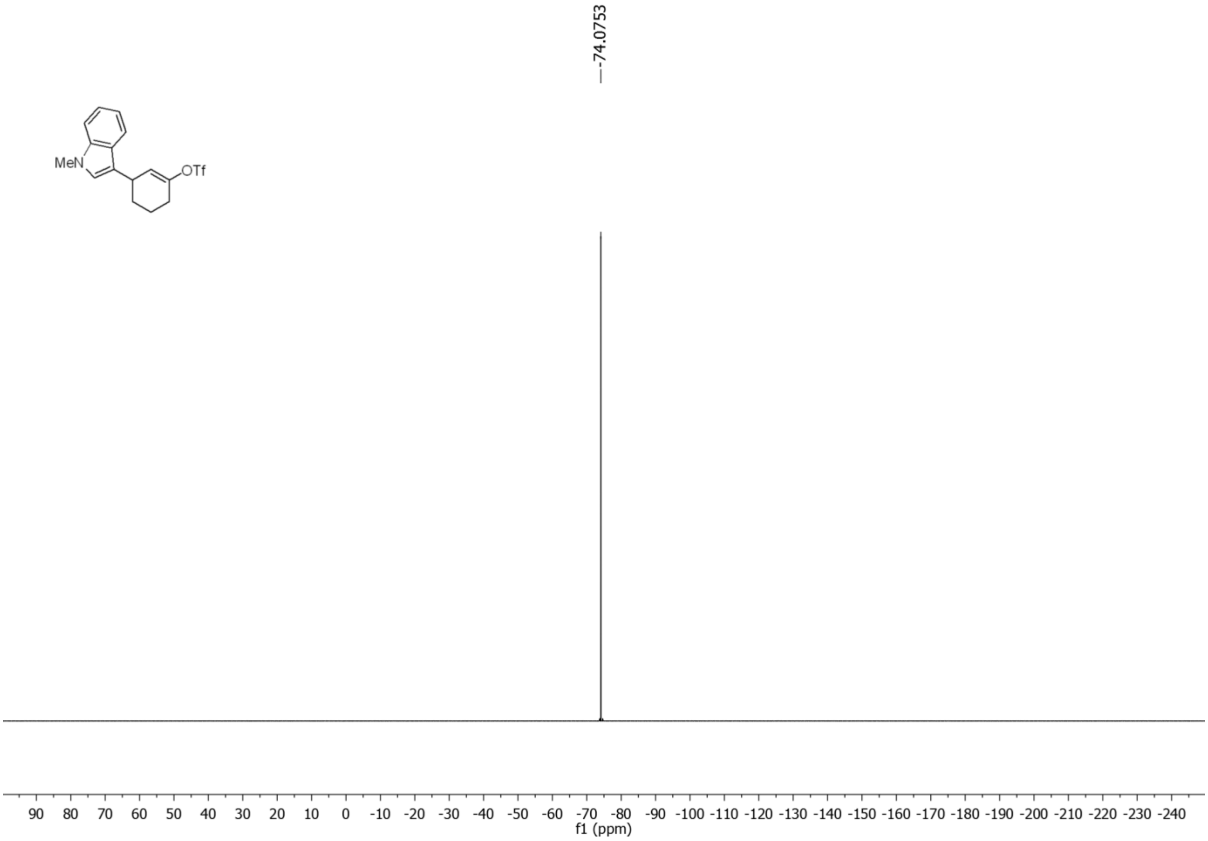

3-(5-Methylfuran-2-yl)cyclohex-1-en-1-yl trifluoromethanesulfonate (3k)

^{1}H-NMR Spectrum:

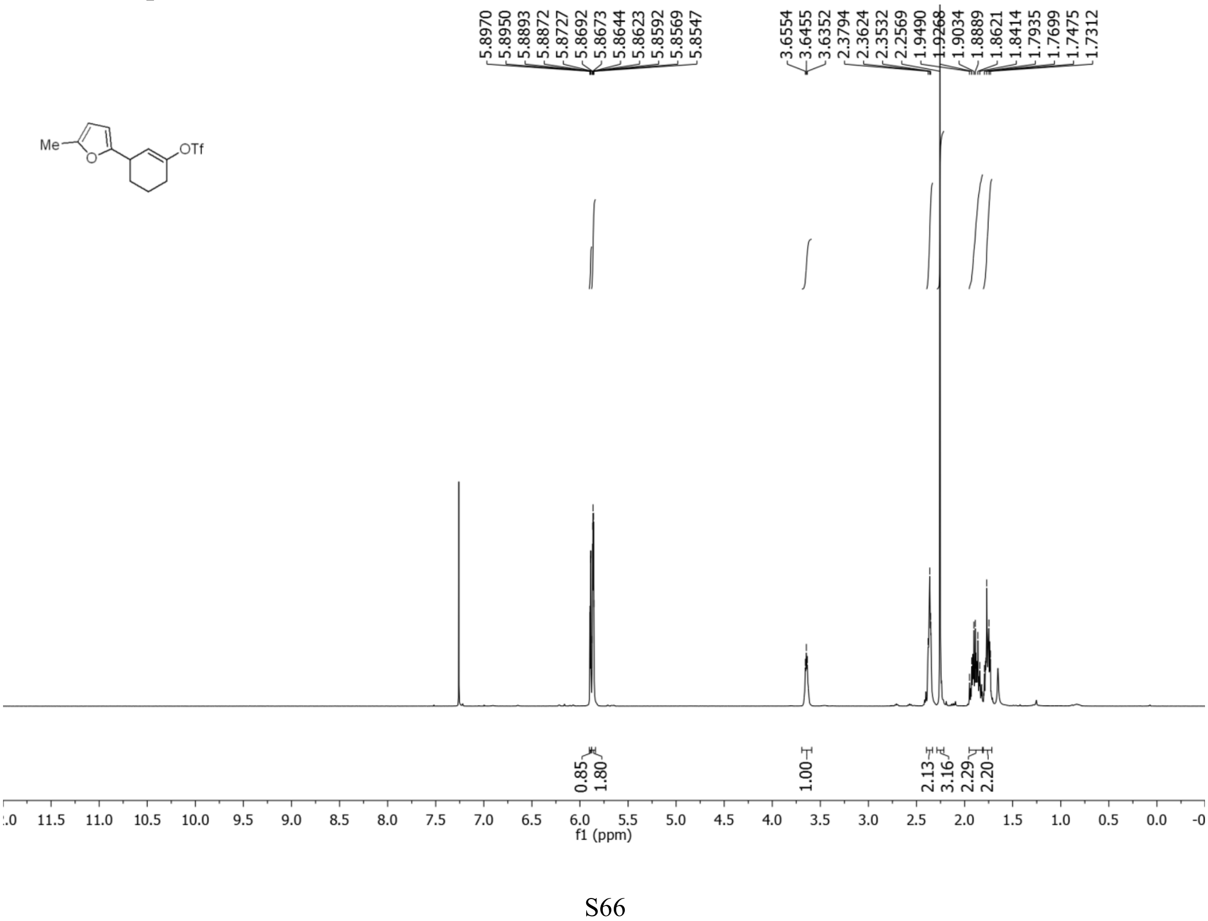

^{13}C-NMR Spectrum:

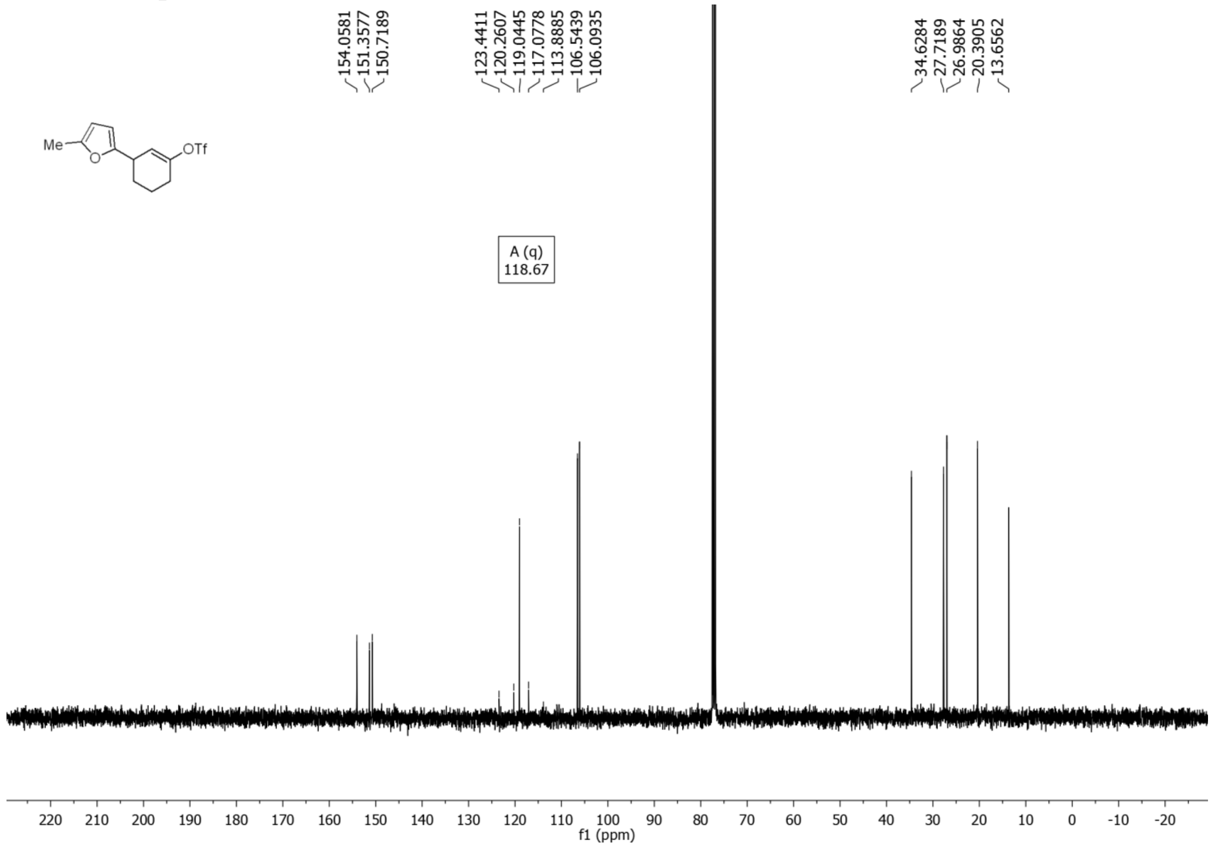

^{19}F-NMR Spectrum:

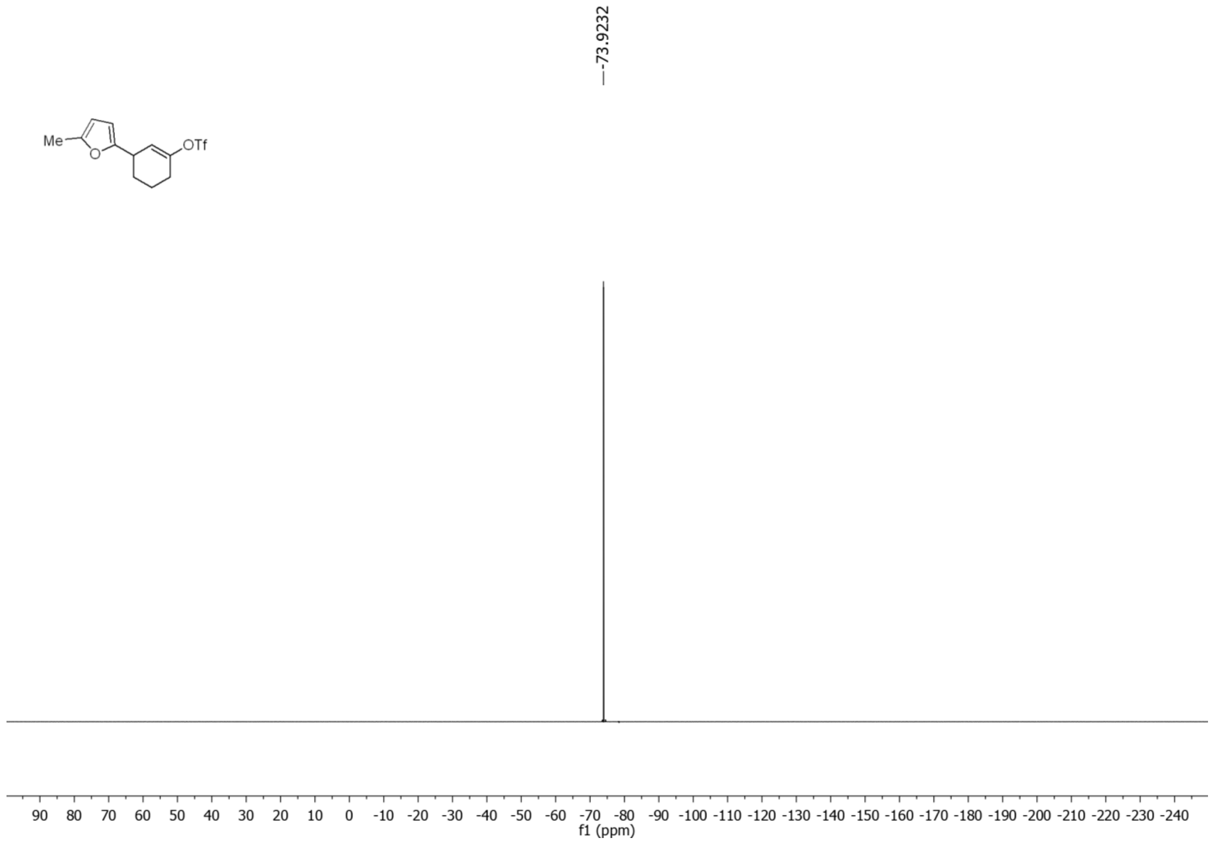

6-Methylcyclohex-1-en-1-yl trifluoromethanesulfonate (3l)

^{1}H-NMR Spectrum:

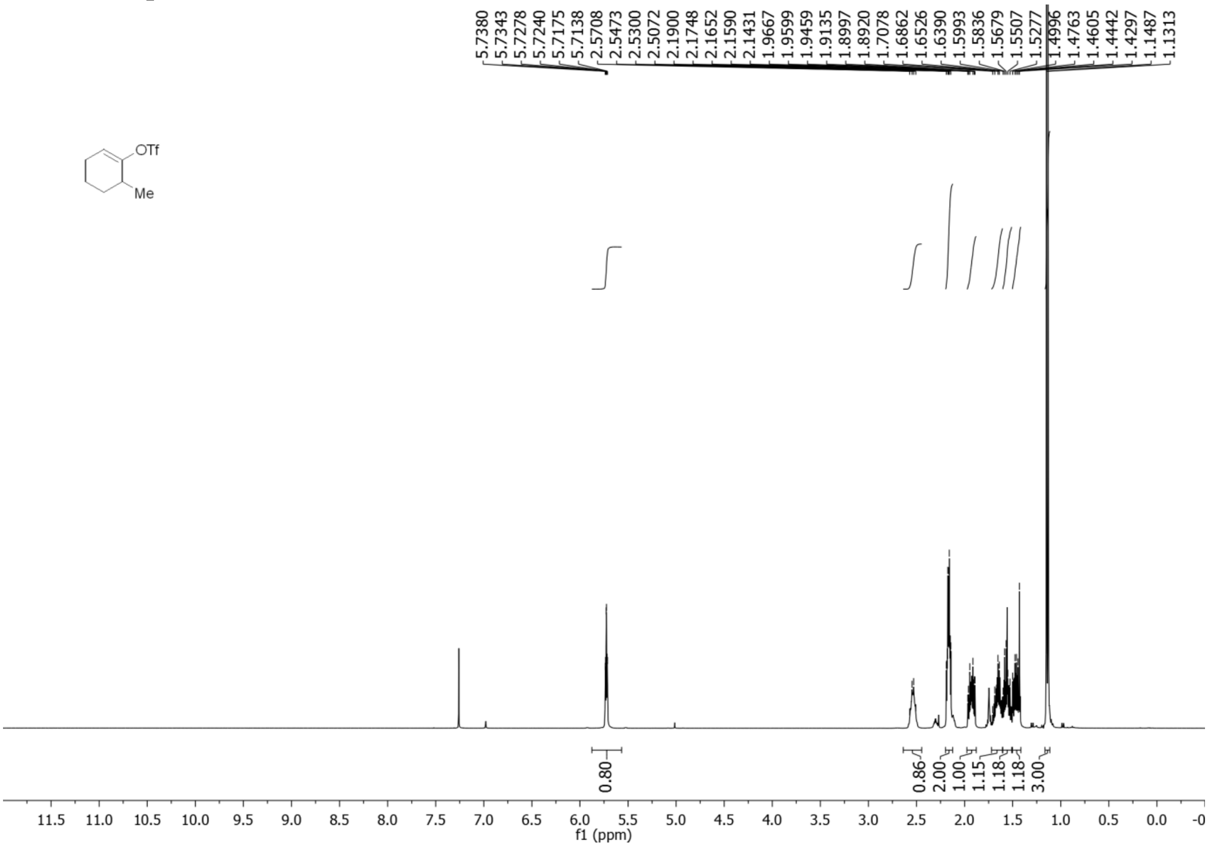

^{13}C-NMR Spectrum:

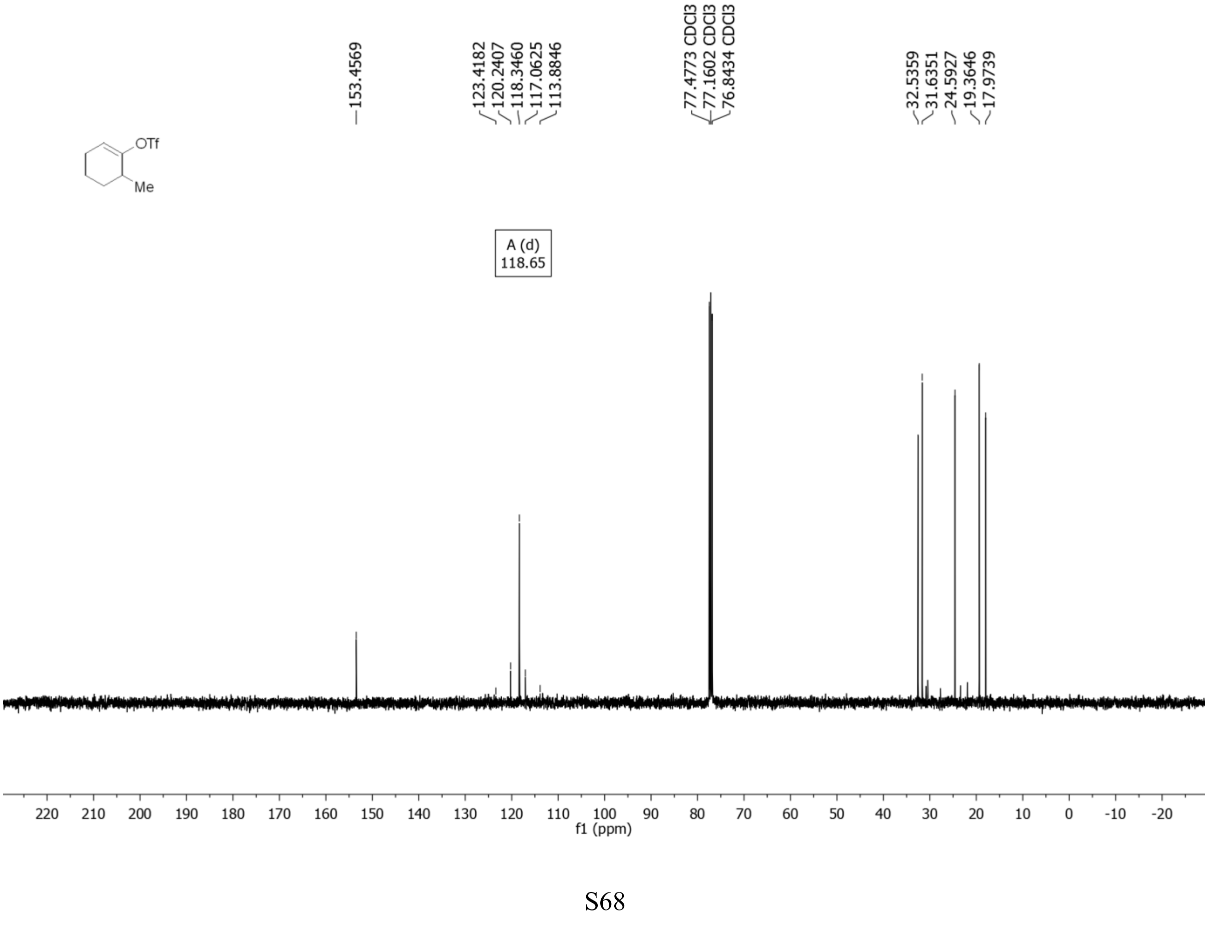

^{19}F-NMR Spectrum:

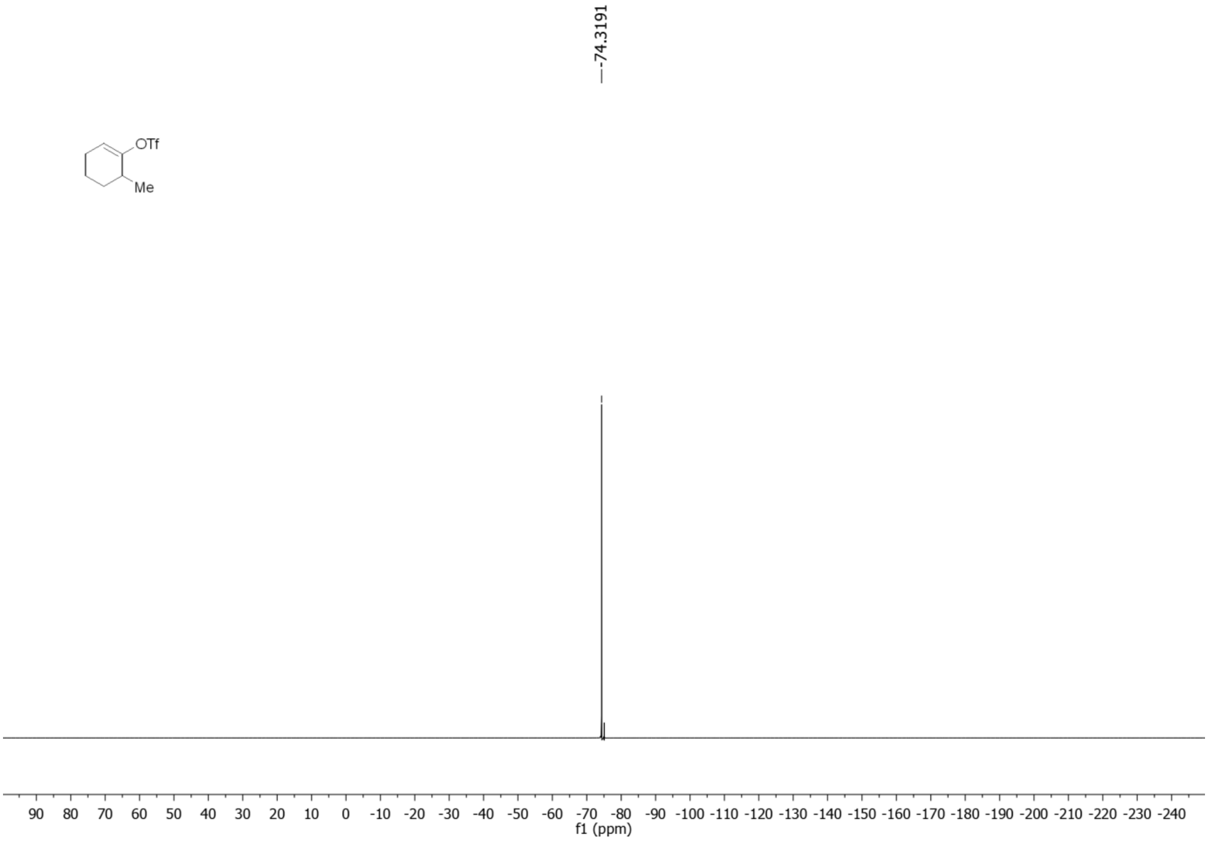

1,1-Dioxido-3,6-dihydro-2*H*-thiopyran-4-yl trifluoromethanesulfonate (3m)

^{1}H-NMR Spectrum:

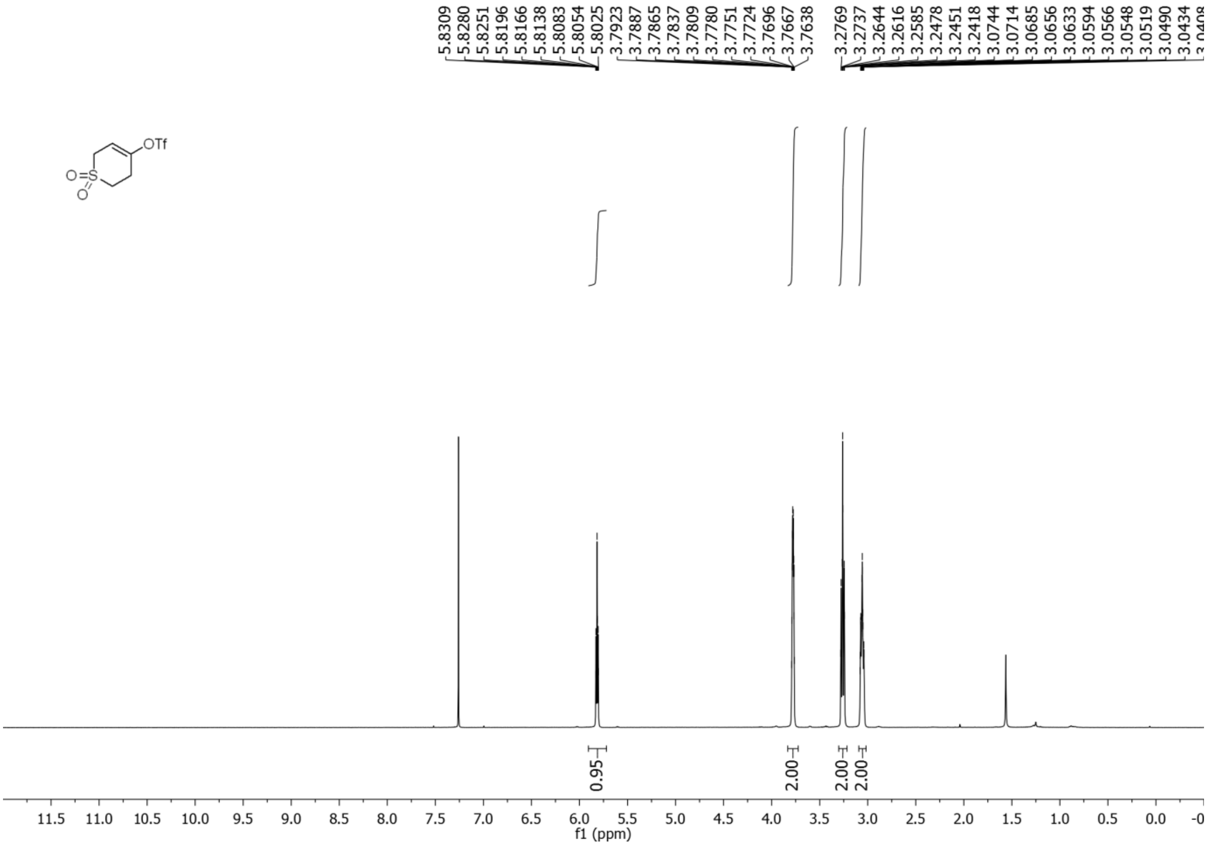

^{13}C-NMR Spectrum:

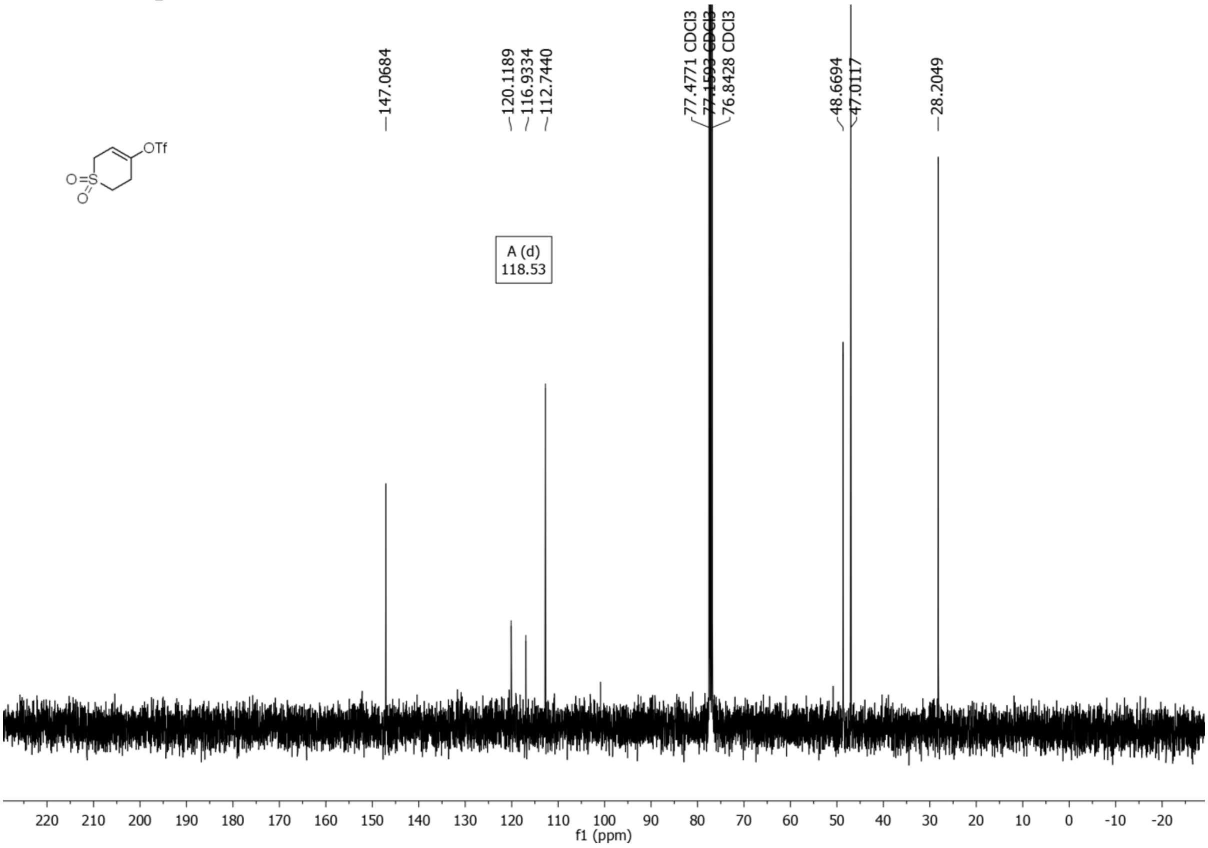

^{19}F-NMR Spectrum:

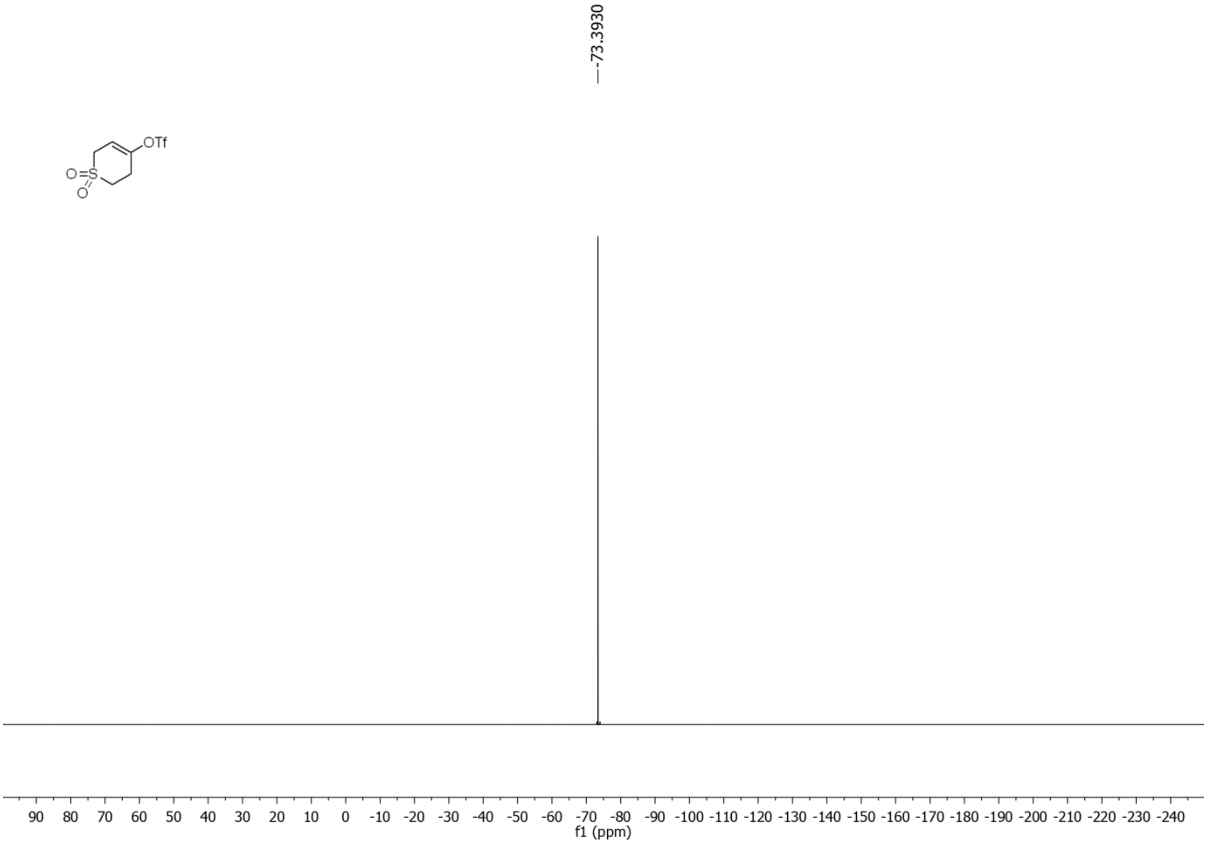

3,6-Dihydro-2*H*-pyran-4-yl trifluoromethanesulfonate (3n)

^{1}H-NMR Spectrum:

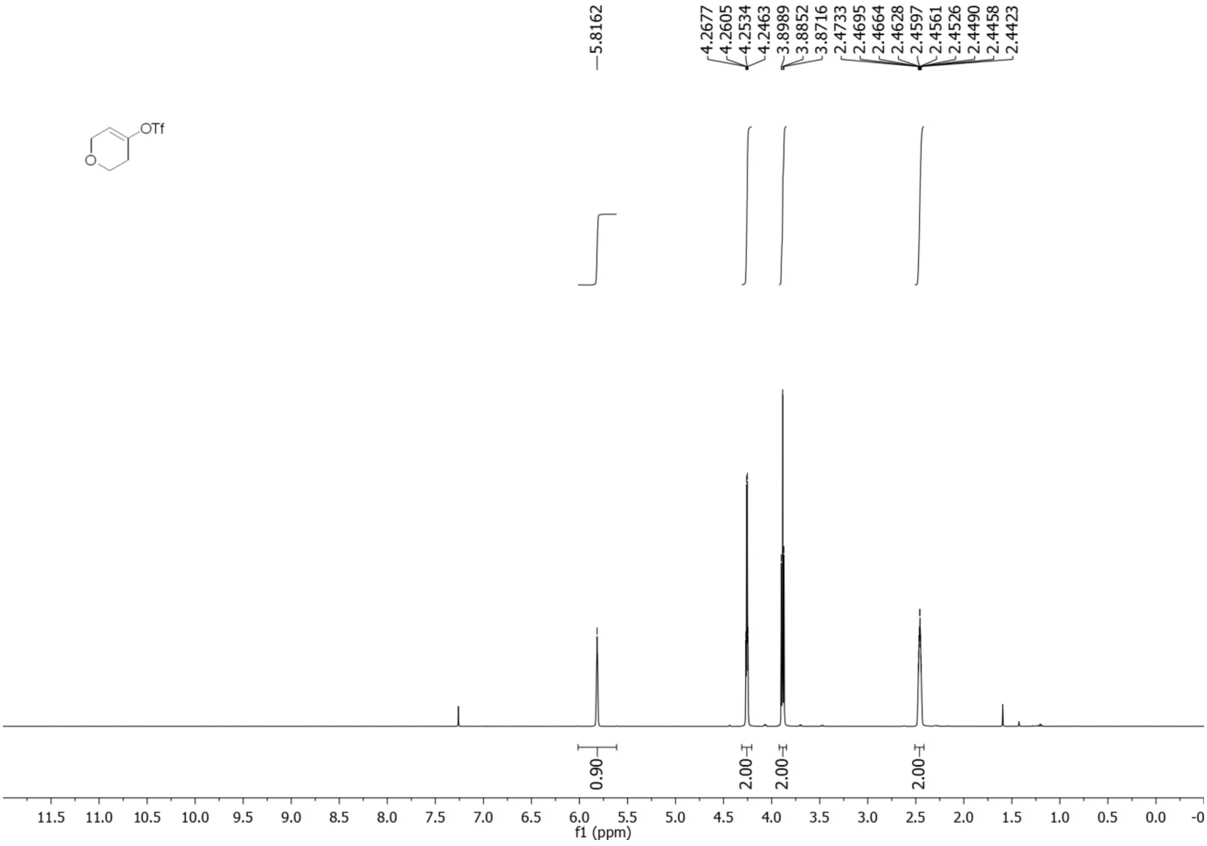

^{13}C-NMR Spectrum:

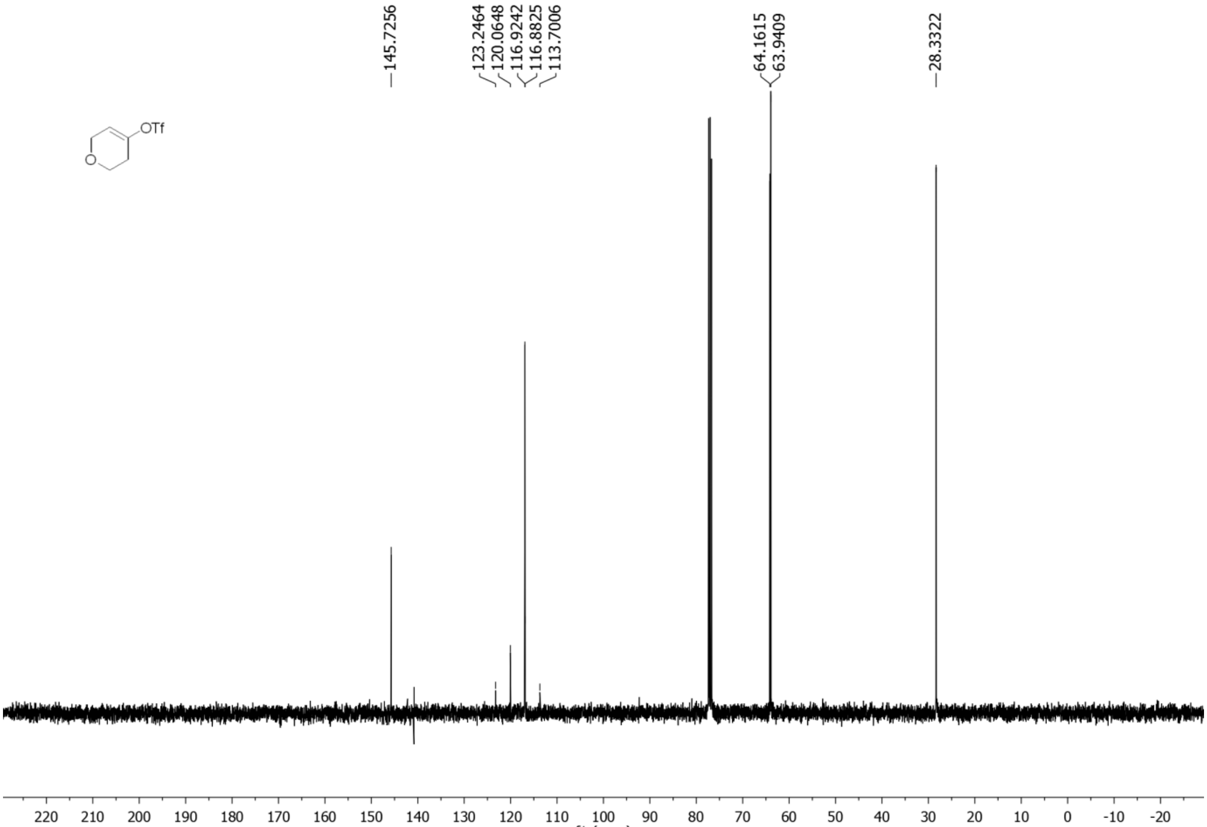

^{19}F-NMR Spectrum:

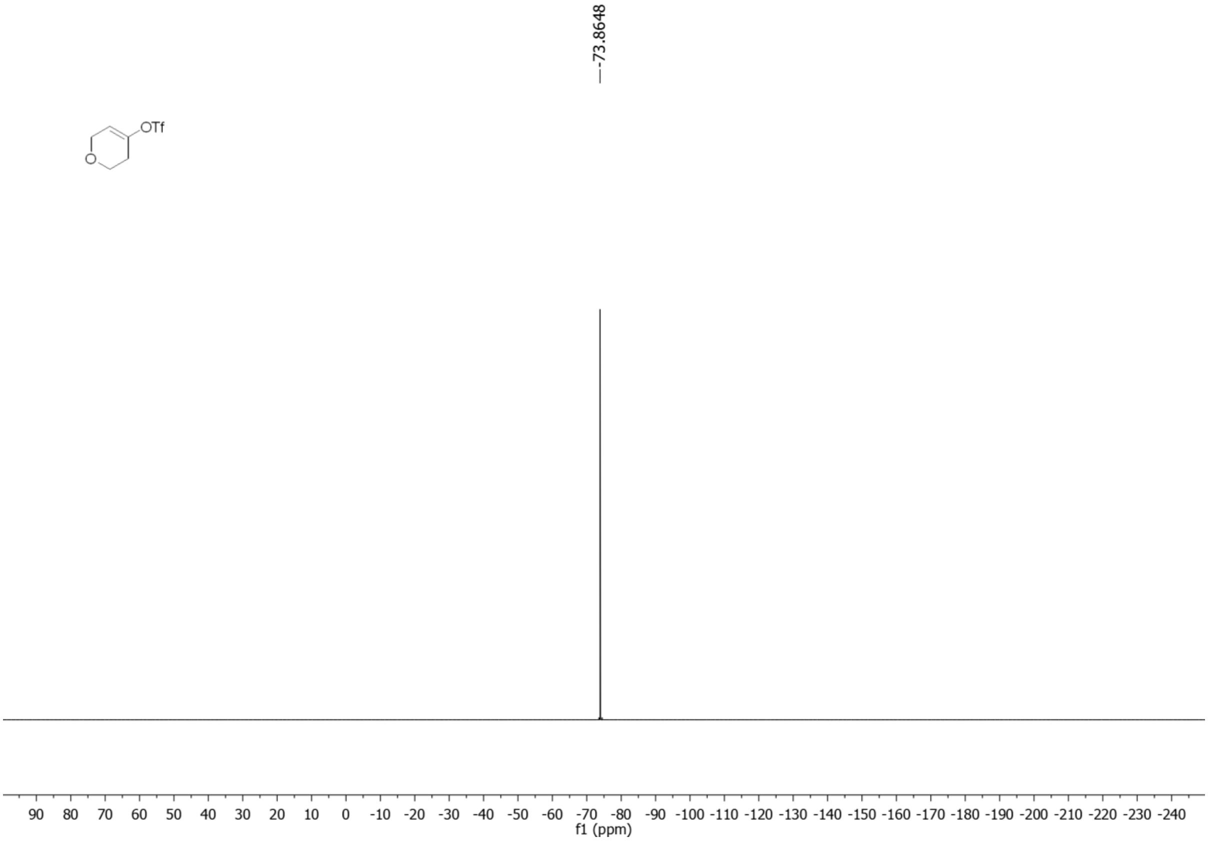

***tert*-Butyl 5-(((trifluoromethyl)sulfonyl)oxy)-3,6-dihydropyridine-1(2*H*)-carboxylate (3o)**

^{1}H-NMR Spectrum:

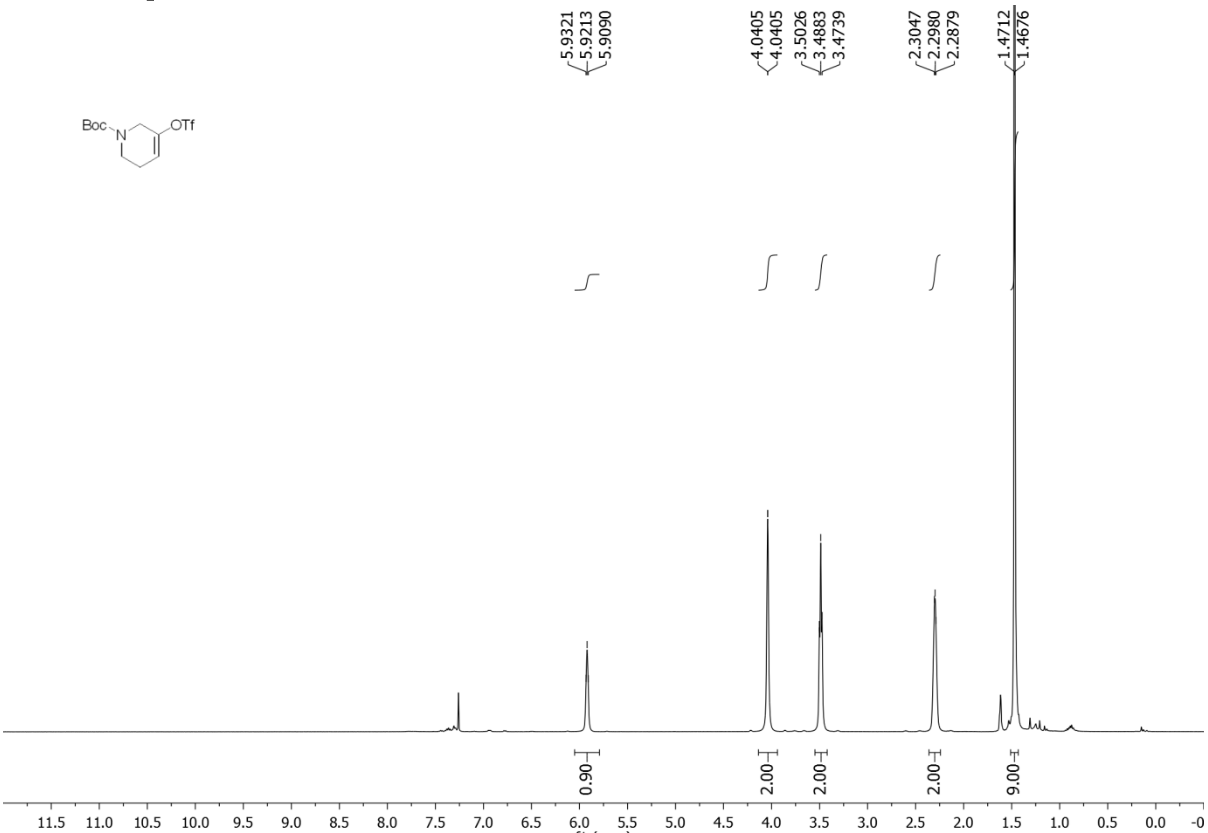

^{13}C-NMR Spectrum:

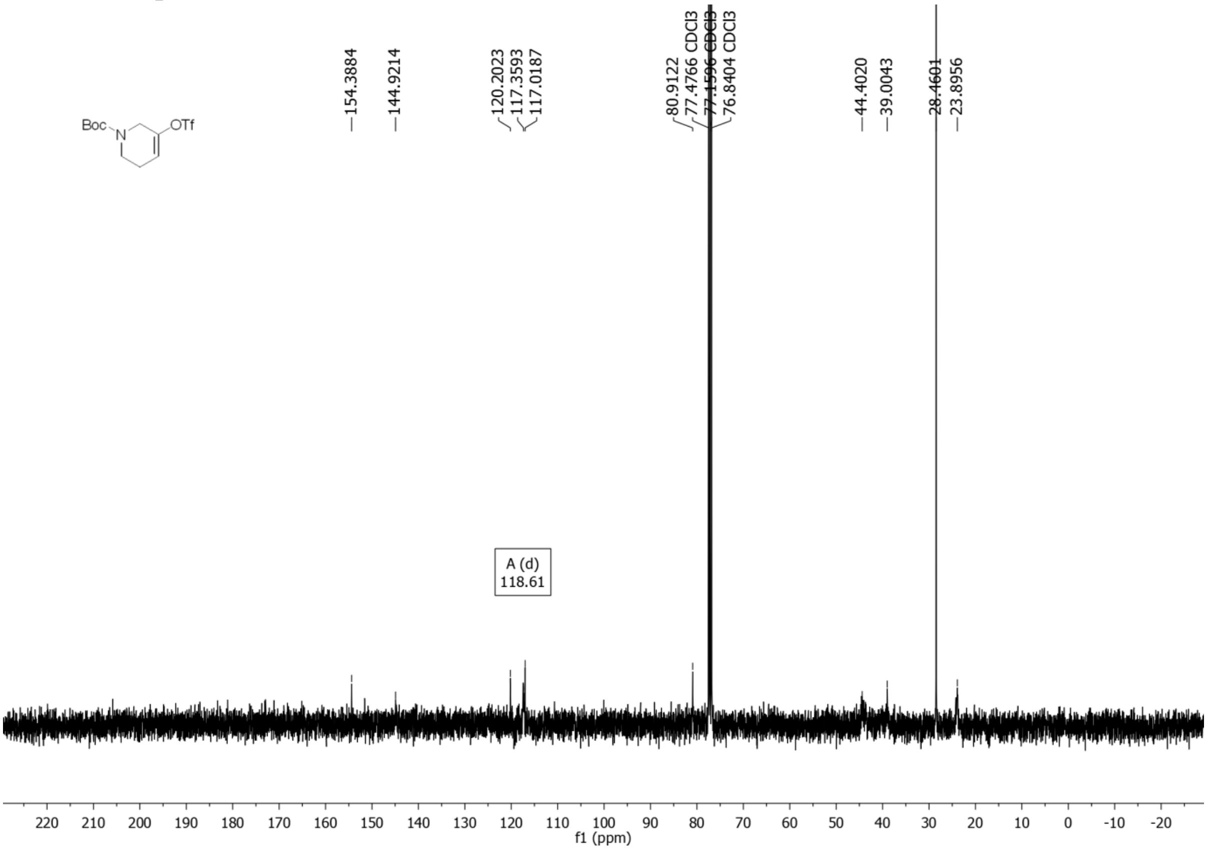

^{19}F-NMR Spectrum:

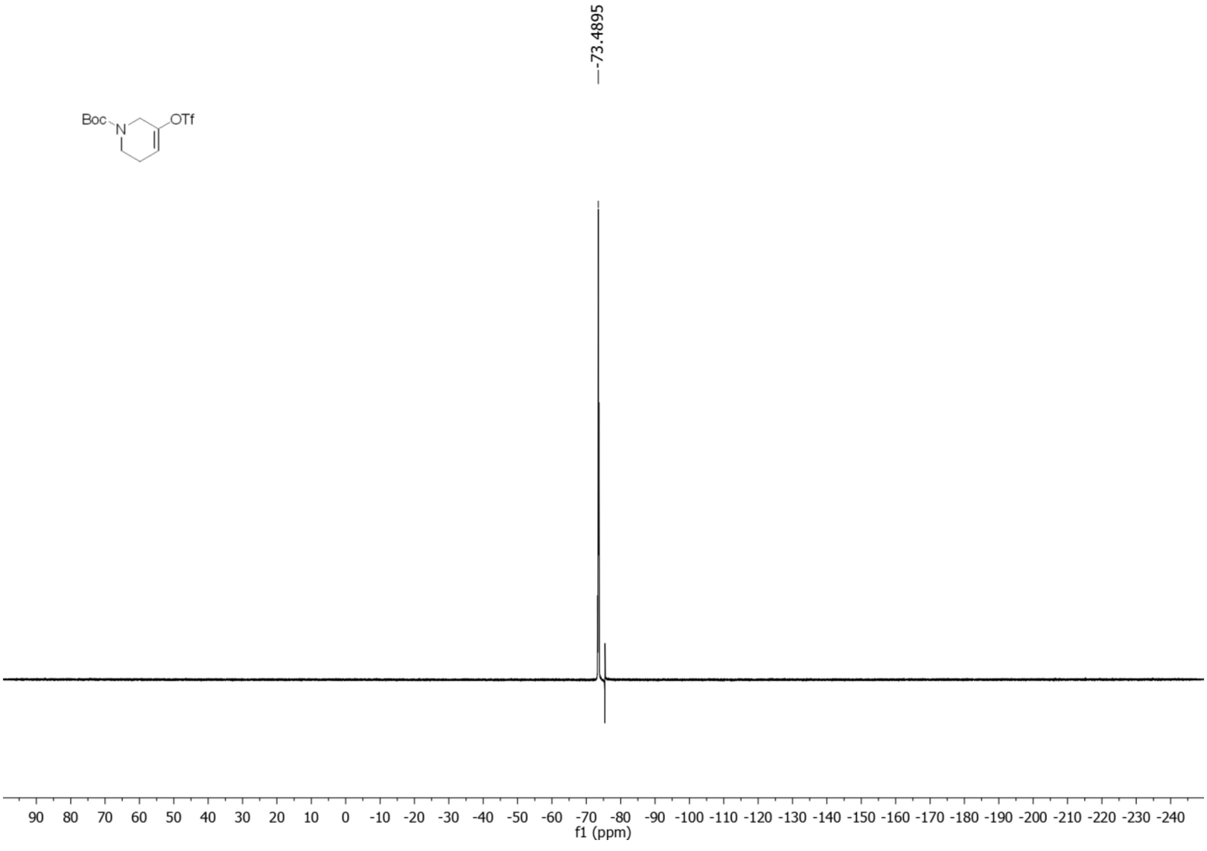

***tert*-Butyl 5-(((trifluoromethyl)sulfonyl)oxy)-3,4-dihydropyridine-1(*2H*)-carboxylate (3o')**

^{1}H-NMR Spectrum:

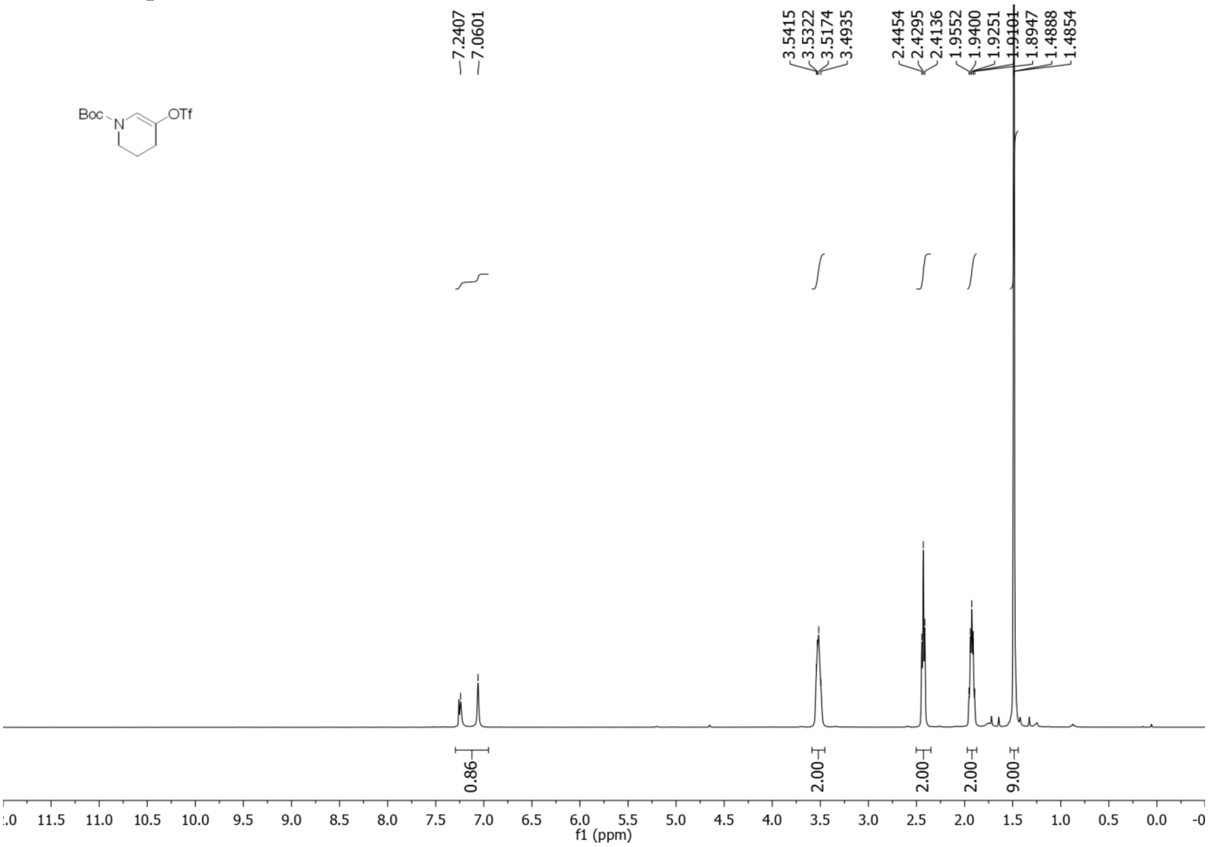

^{13}C-NMR Spectrum:

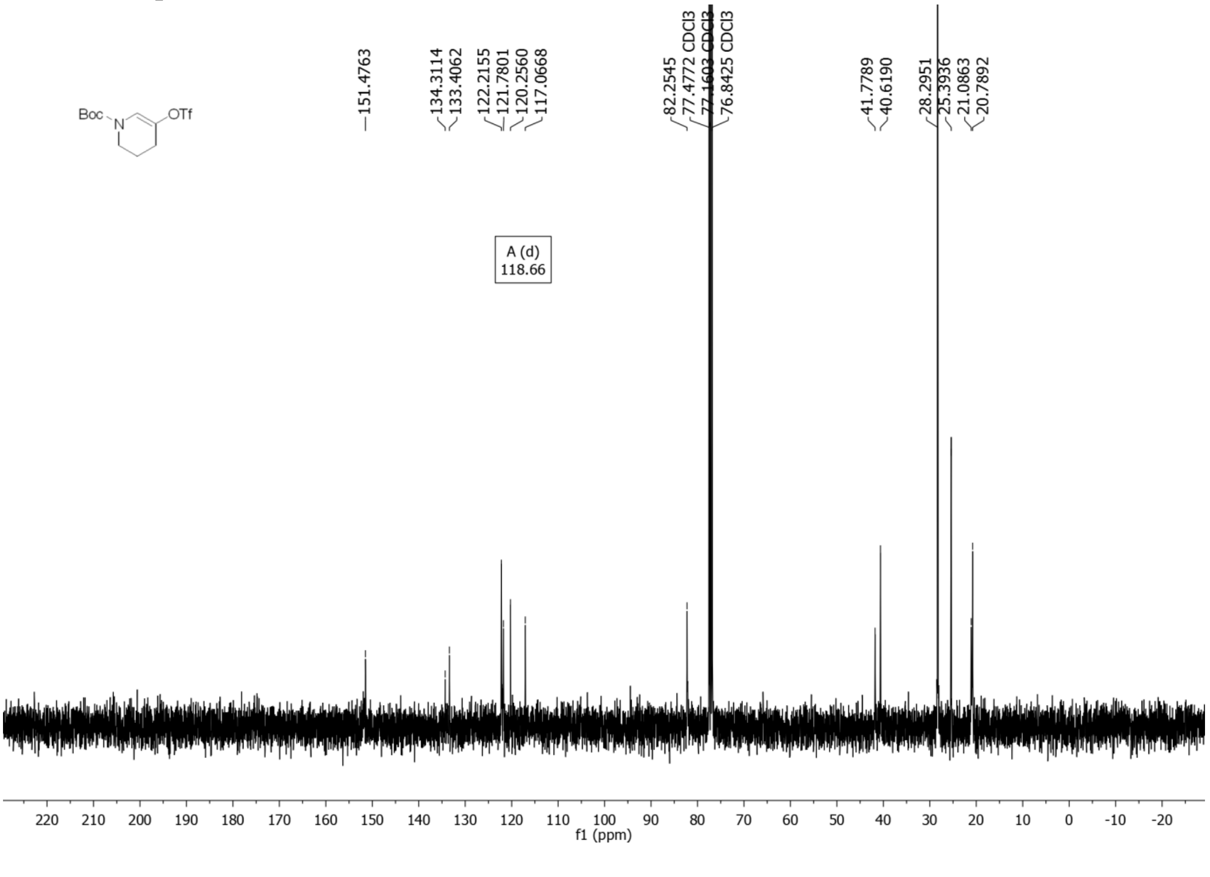

^{19}F-NMR Spectrum:

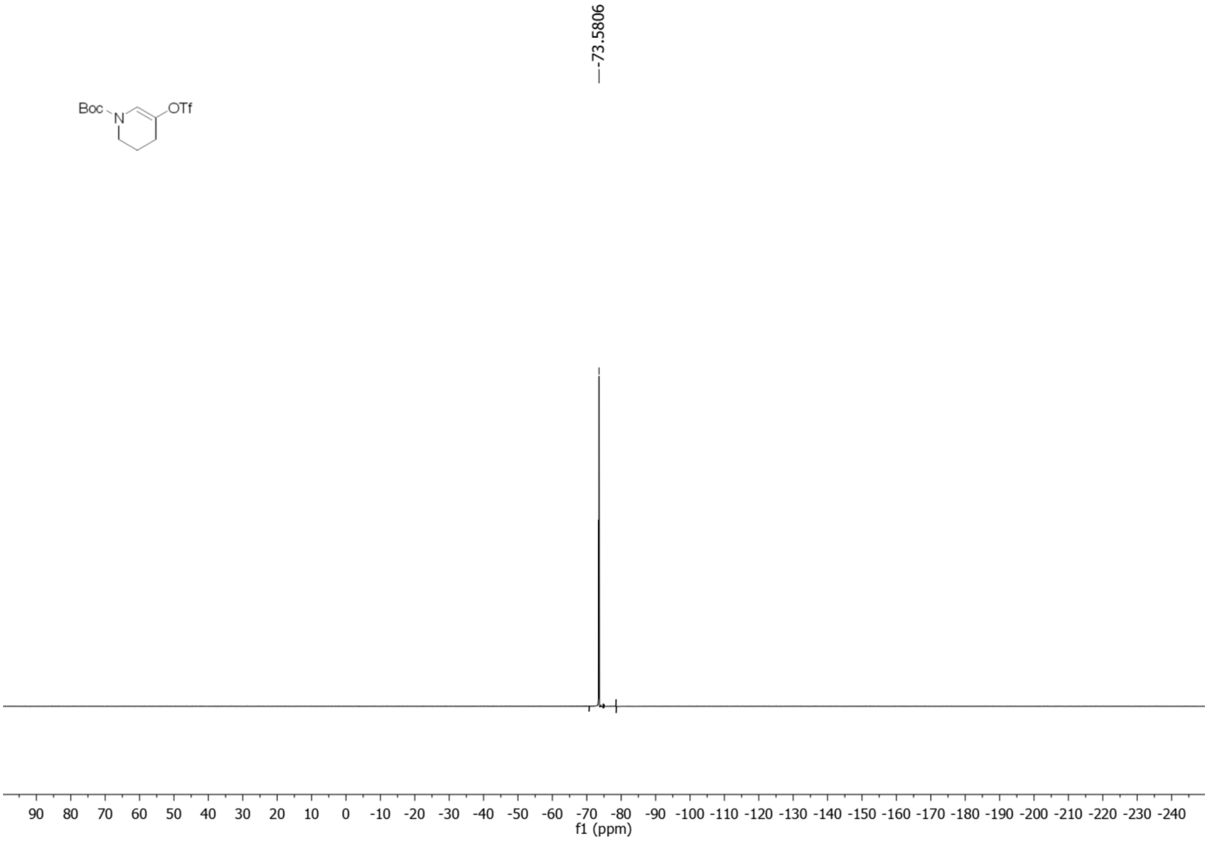

***tert*-Butyl 4-(((trifluoromethyl)sulfonyl)oxy)-3,6-dihydropyridine-1(2*H*)-carboxylate (3p)**

^{1}H-NMR Spectrum:

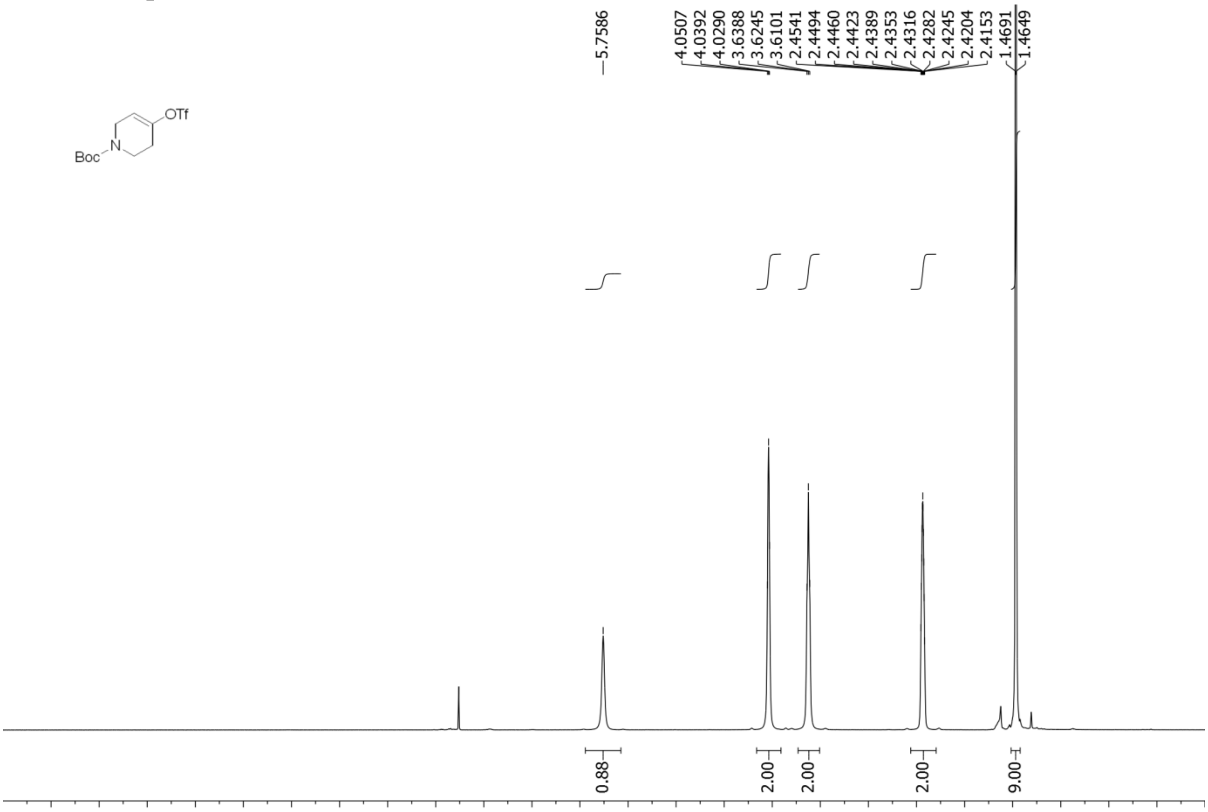

^{13}C-NMR Spectrum:

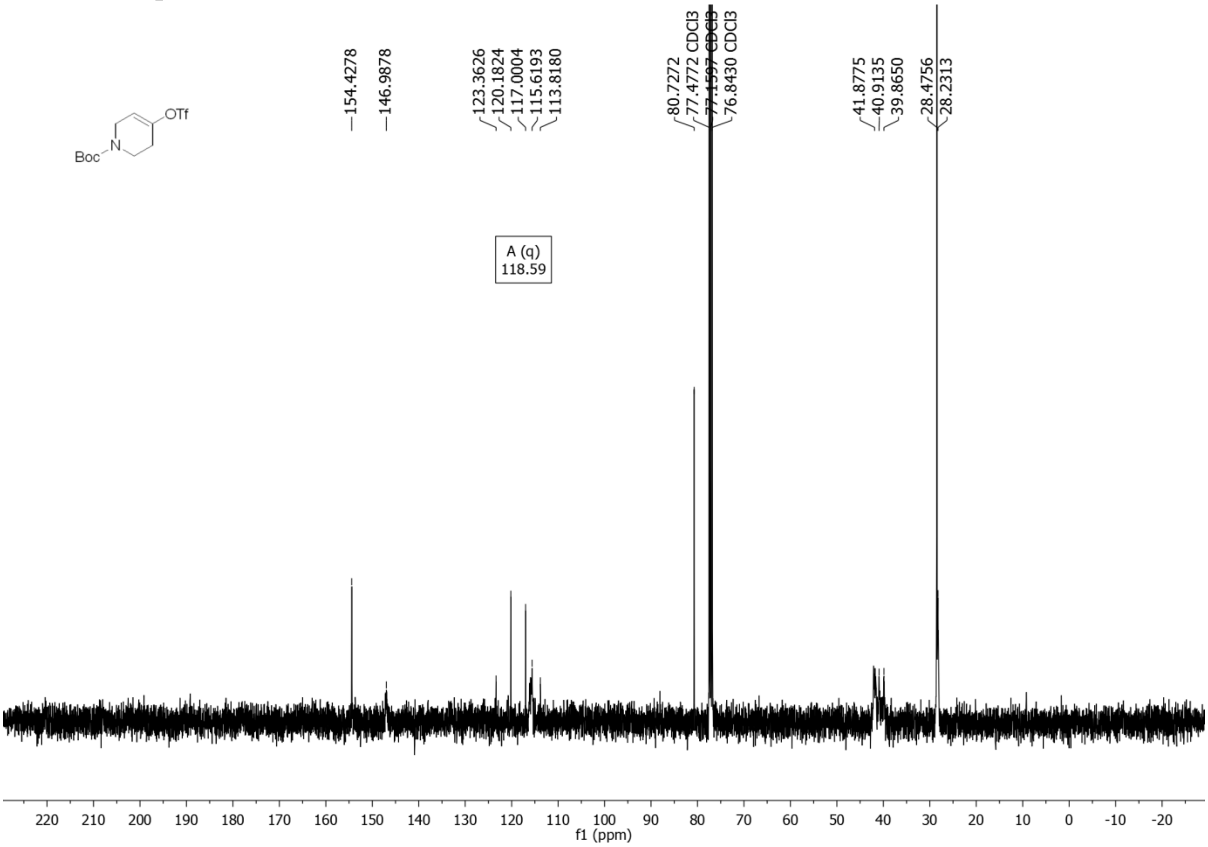

^{19}F-NMR Spectrum:

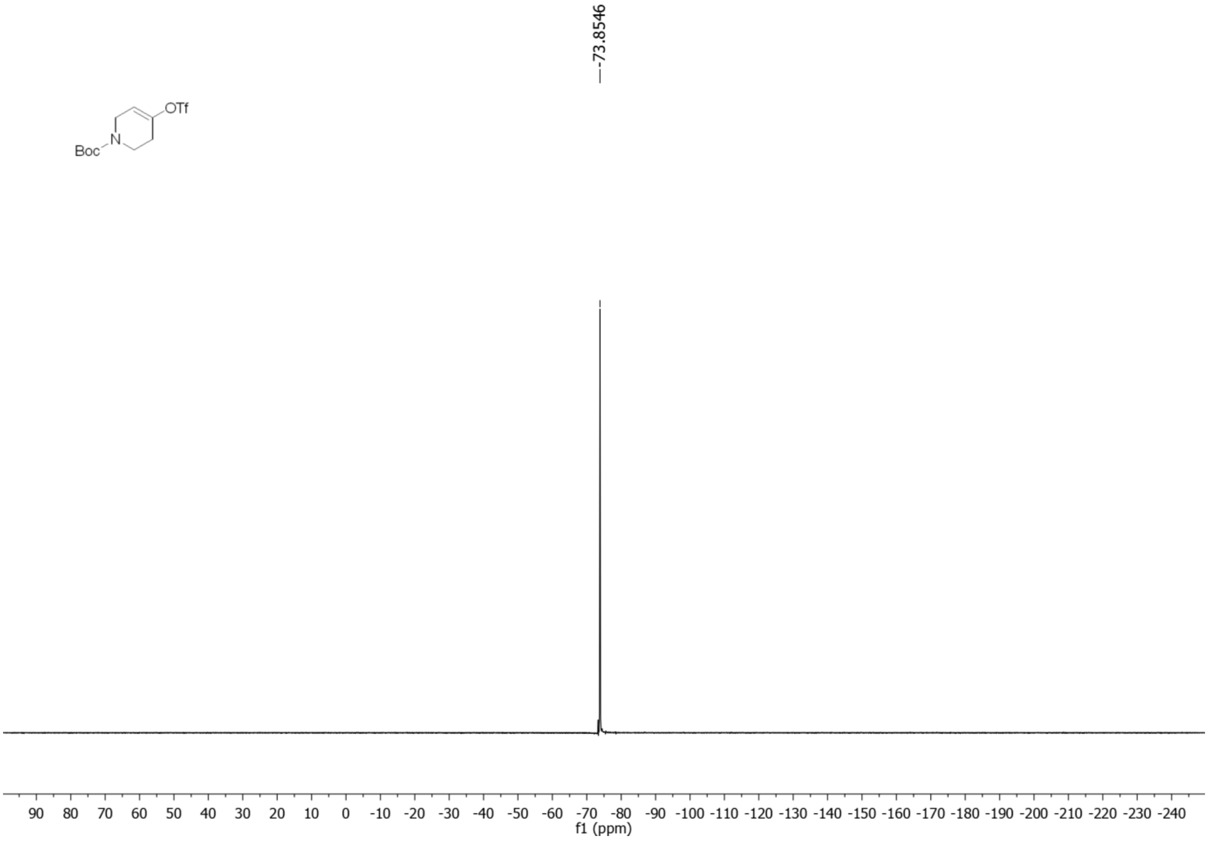

1-Benzyloxycarbonyl-1,2,3,6-tetrahydropyridin-4-yl trifluoromethanesulfonate (3q)

^{1}H-NMR Spectrum:

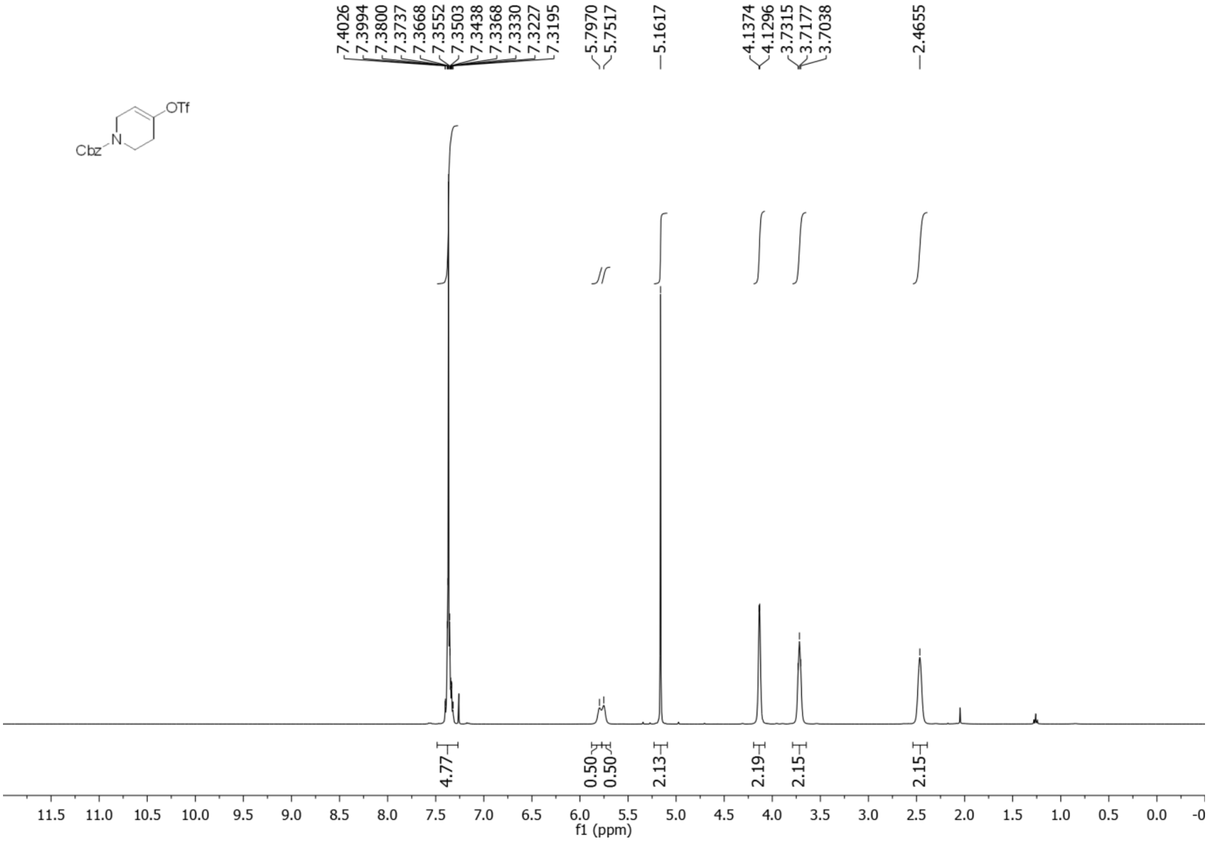

^{13}C-NMR Spectrum:

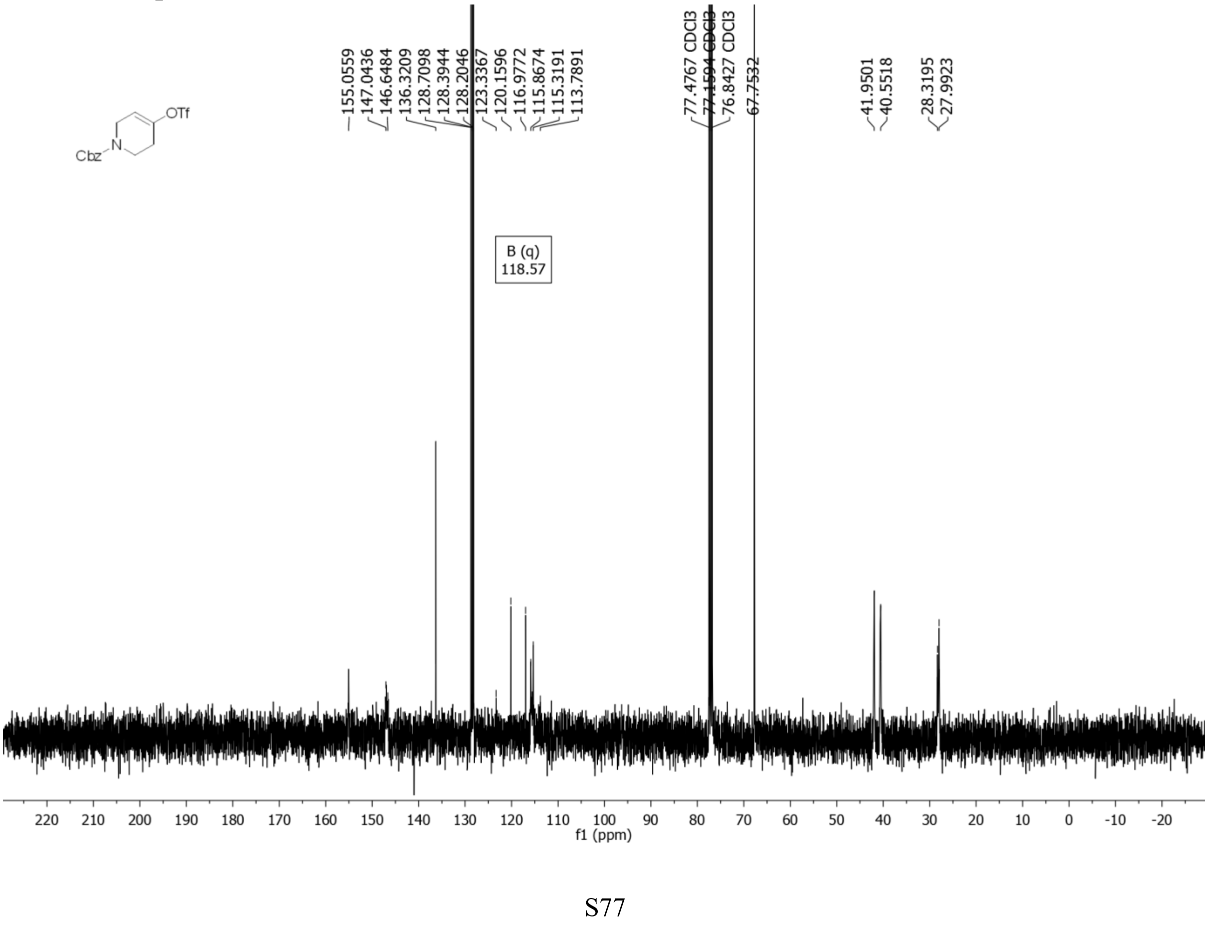

^{19}F-NMR Spectrum:

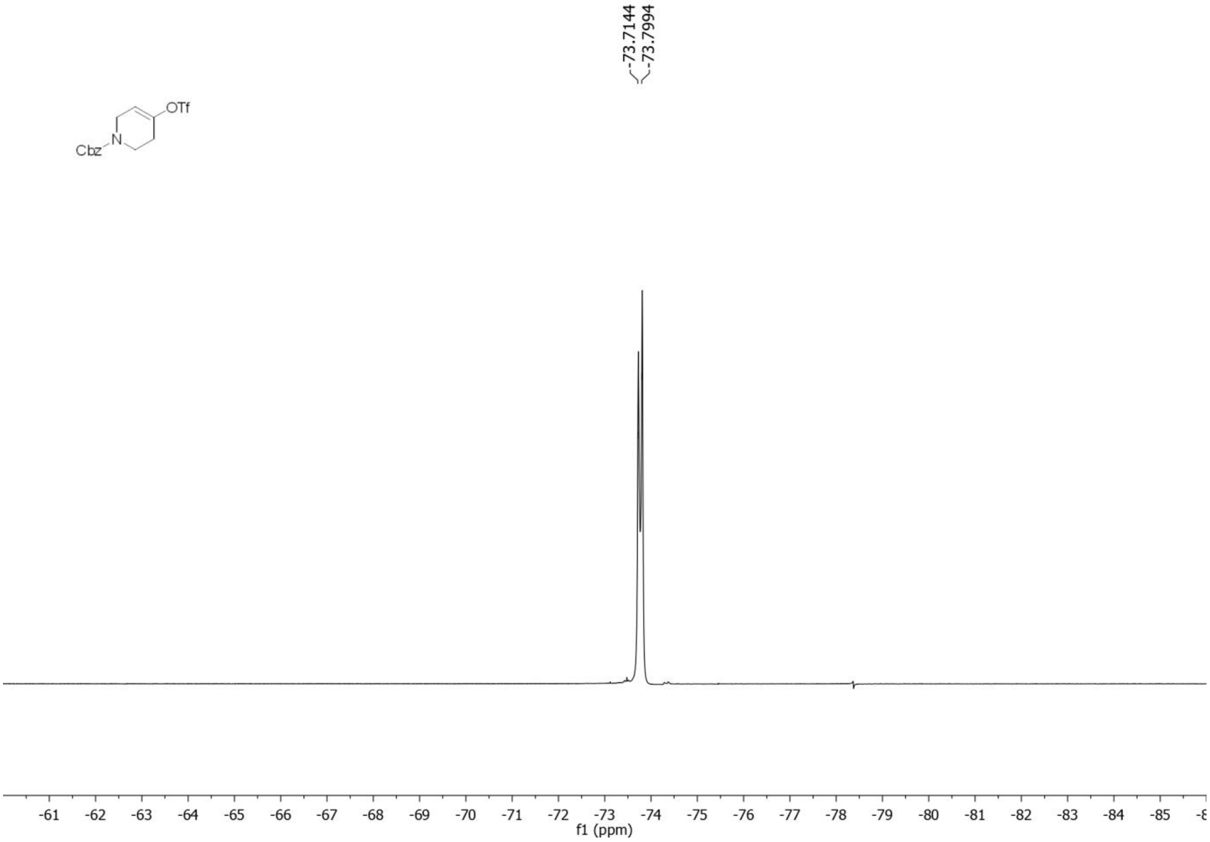

1-Benzyl-1,2,3,6-tetrahydropyridin-4-yl trifluoromethanesulfonate (3r)

^{1}H-NMR Spectrum:

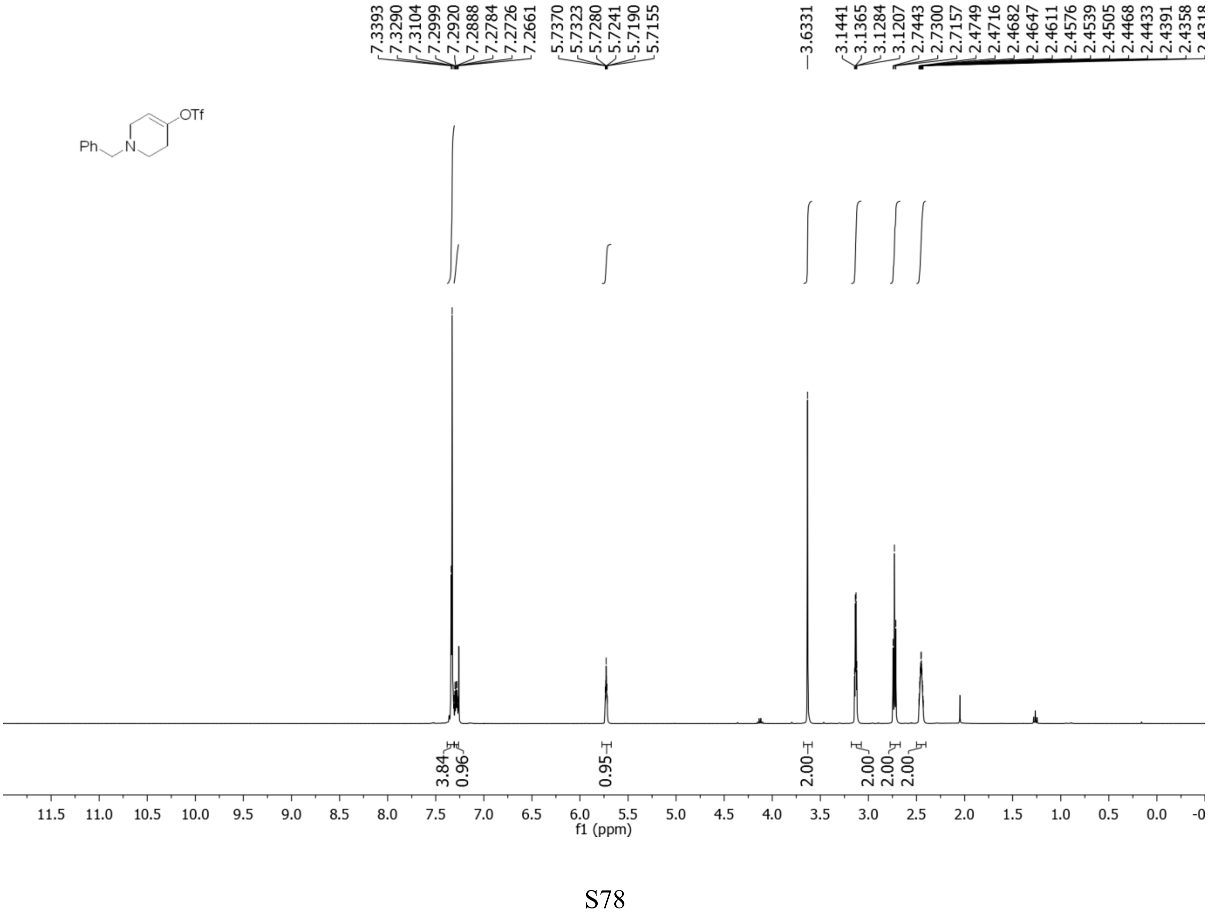

^{13}C-NMR Spectrum:

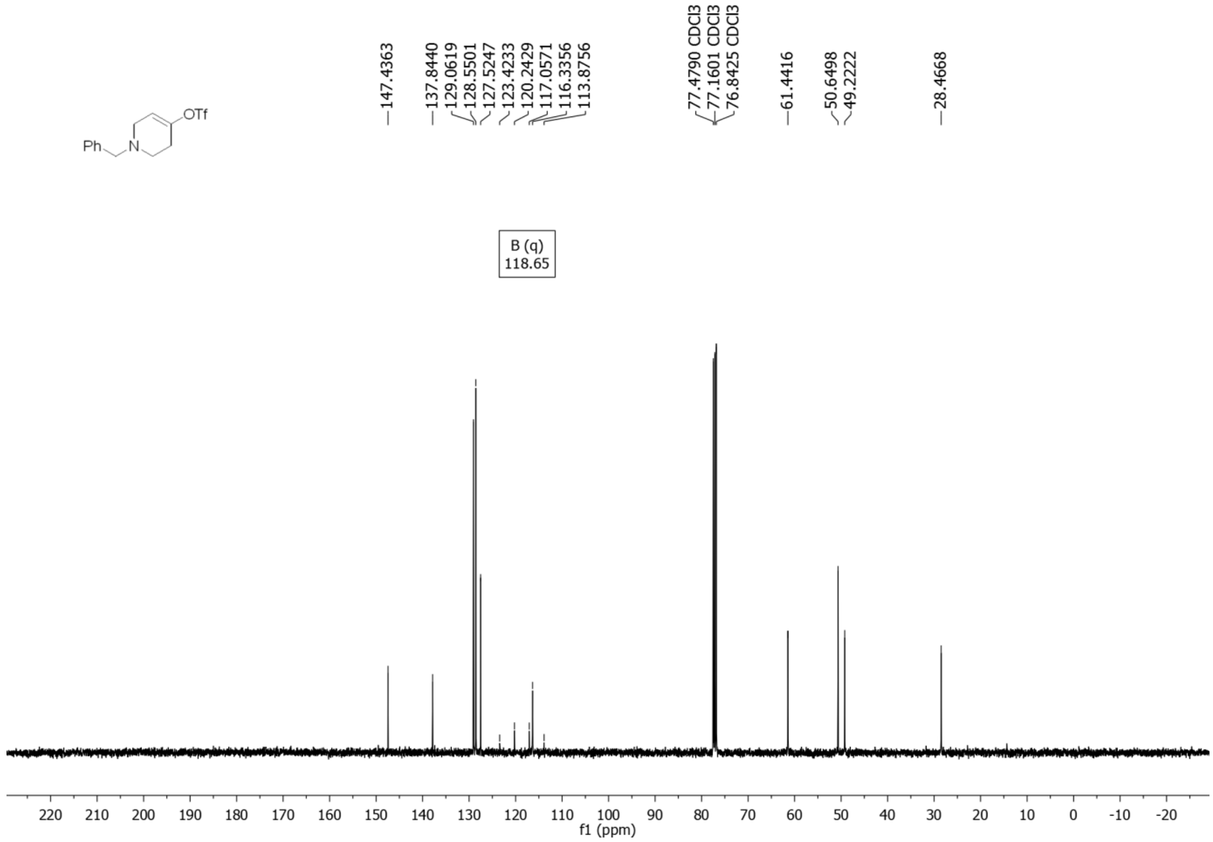

^{19}F-NMR Spectrum:

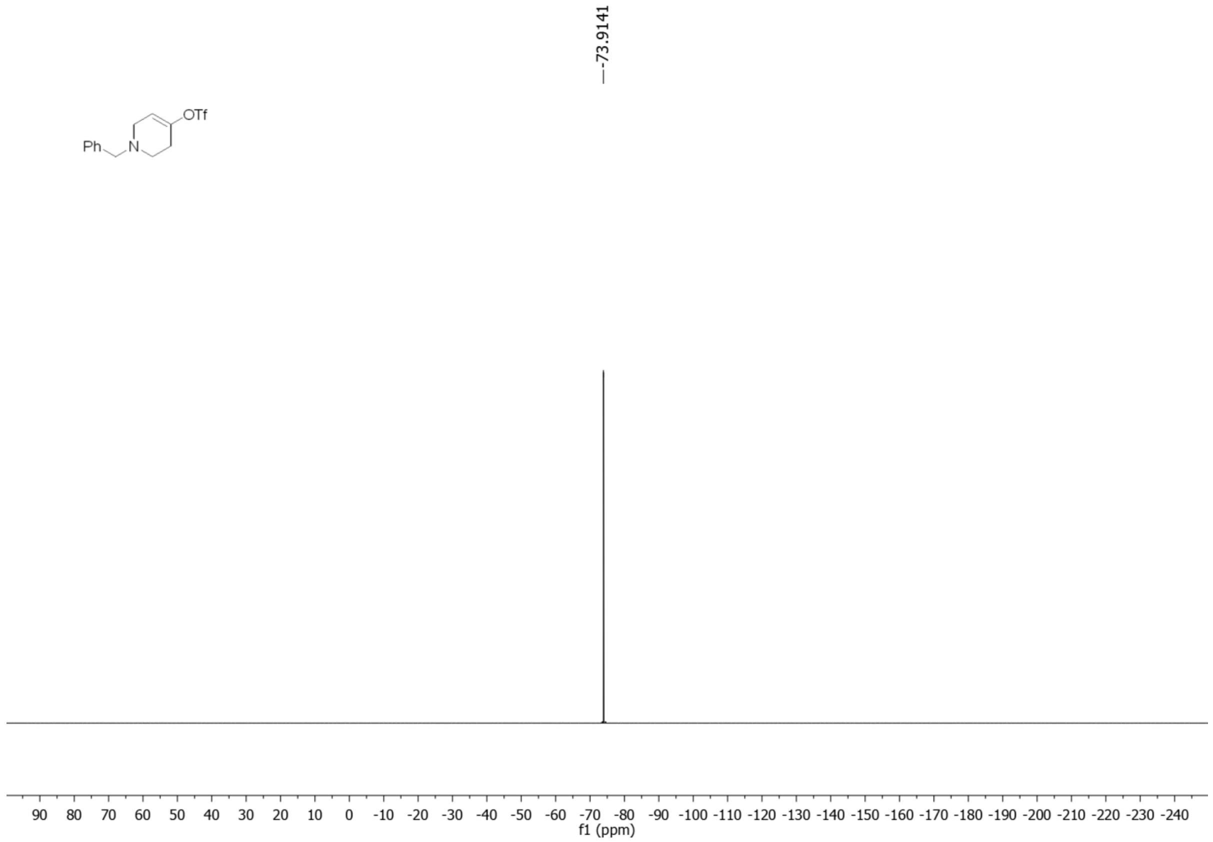

1-Tosyl-1,2,3,6-tetrahydropyridin-4-yl trifluoromethanesulfonate (3s)

^{1}H-NMR Spectrum:

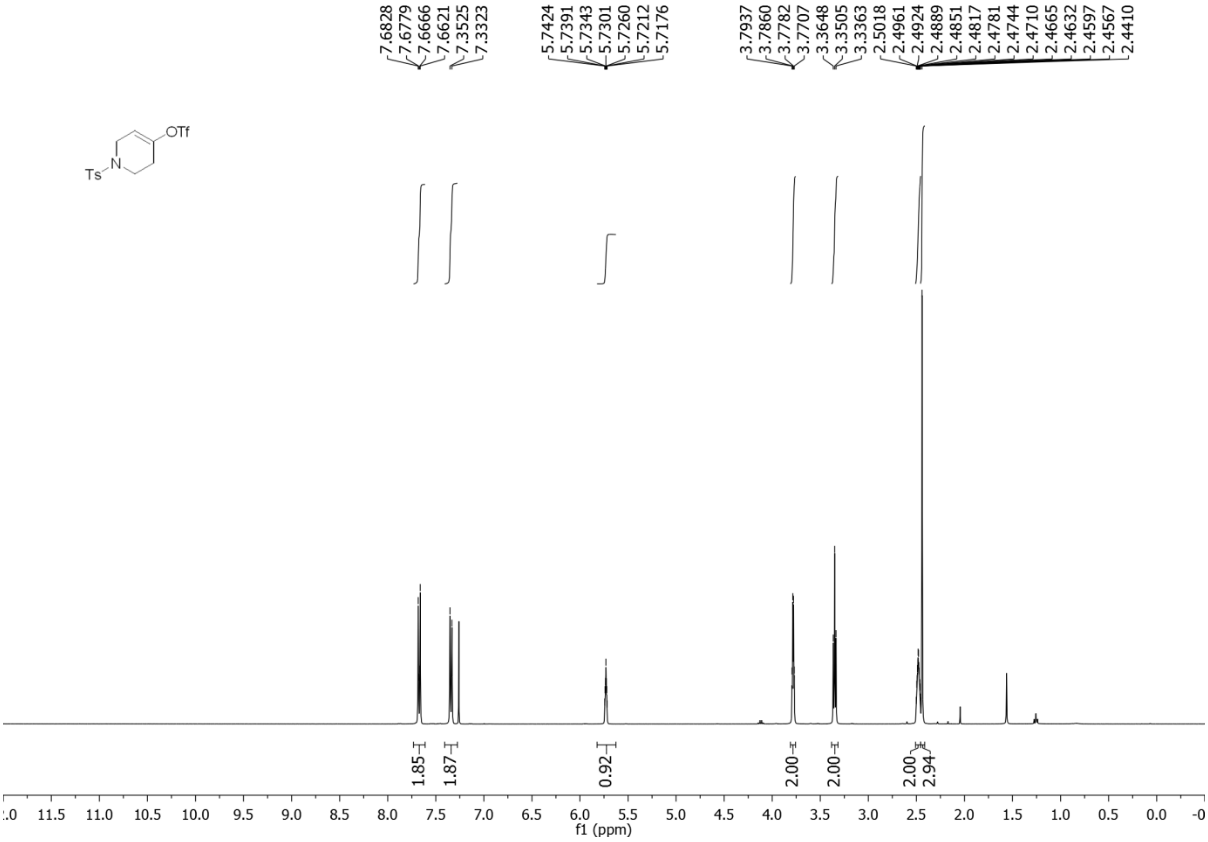

^{13}C-NMR Spectrum:

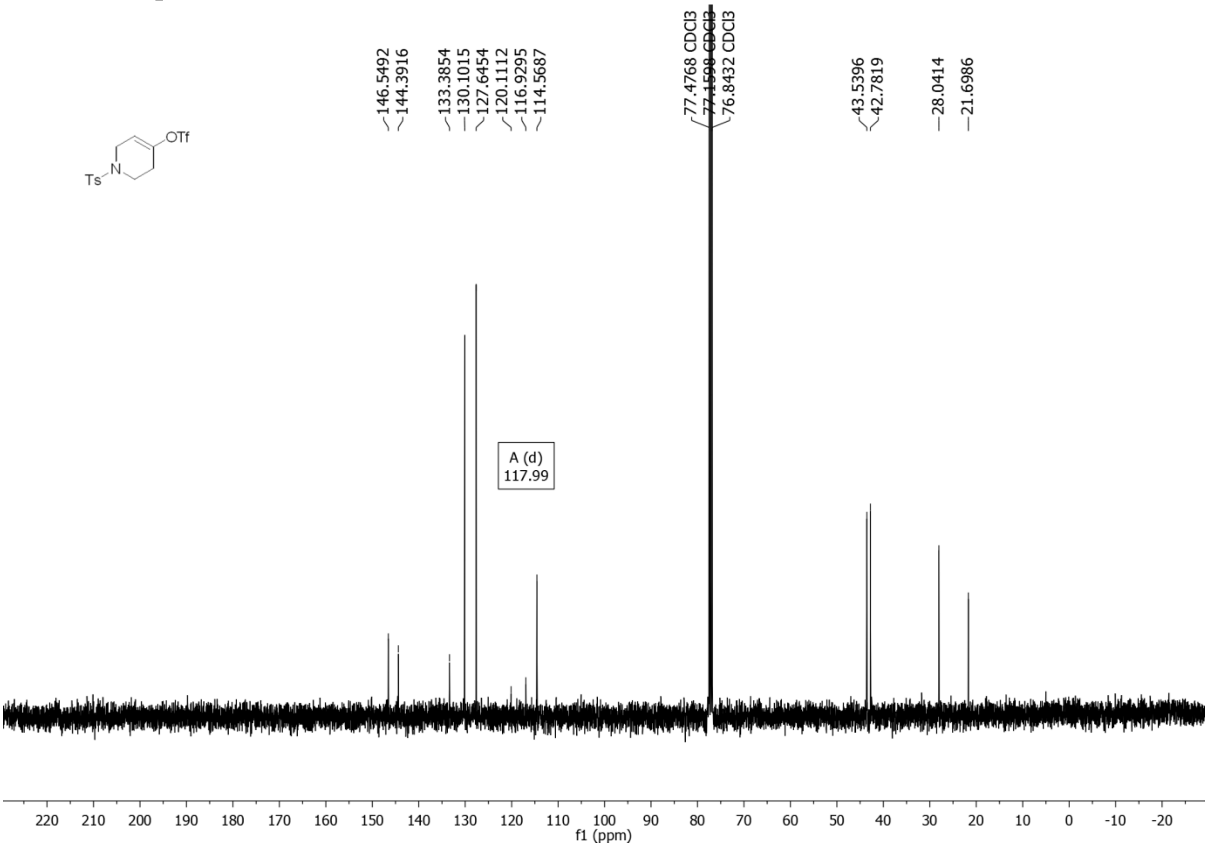

^{19}F-NMR Spectrum:

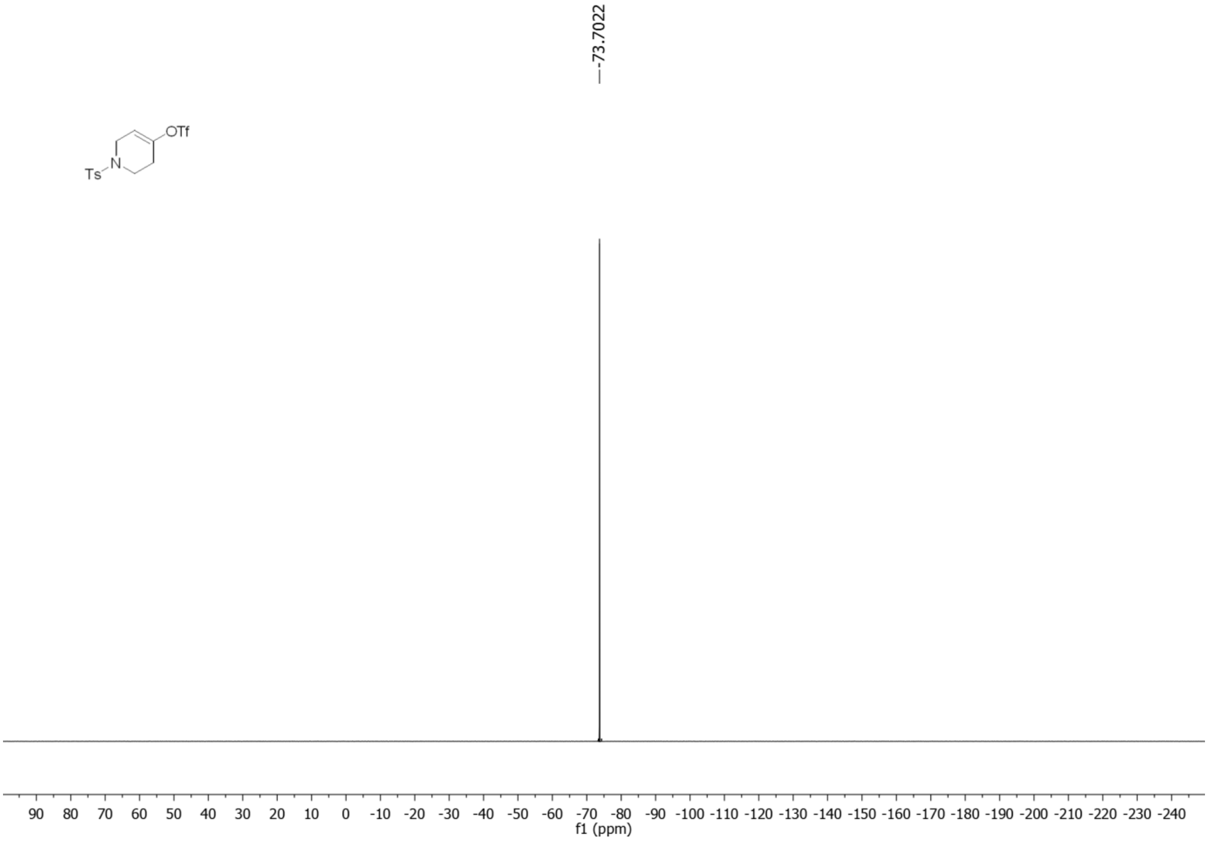

3,4-Dihydronaphthalen-2-yl trifluoromethanesulfonate (3u)

^{1}H-NMR Spectrum:

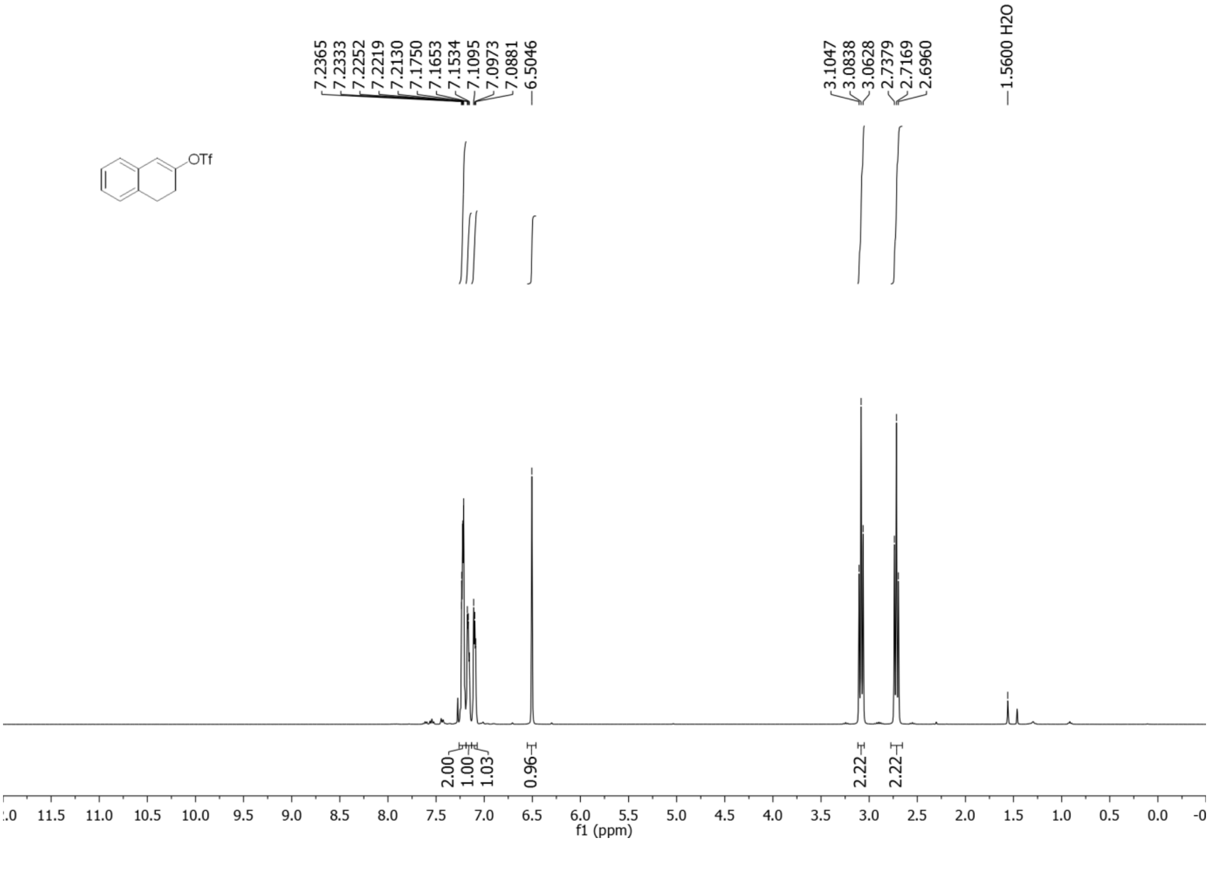

^{13}C-NMR Spectrum:

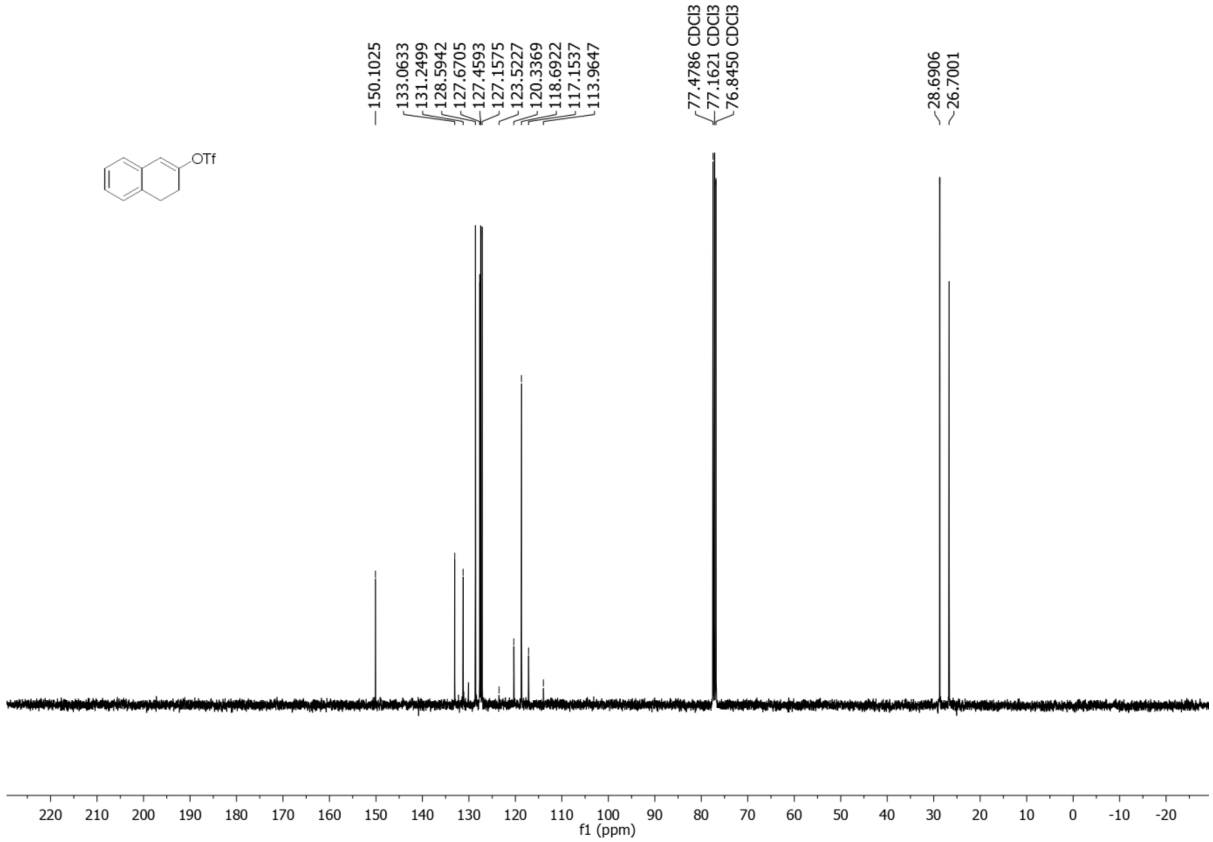

^{19}F-NMR Spectrum:

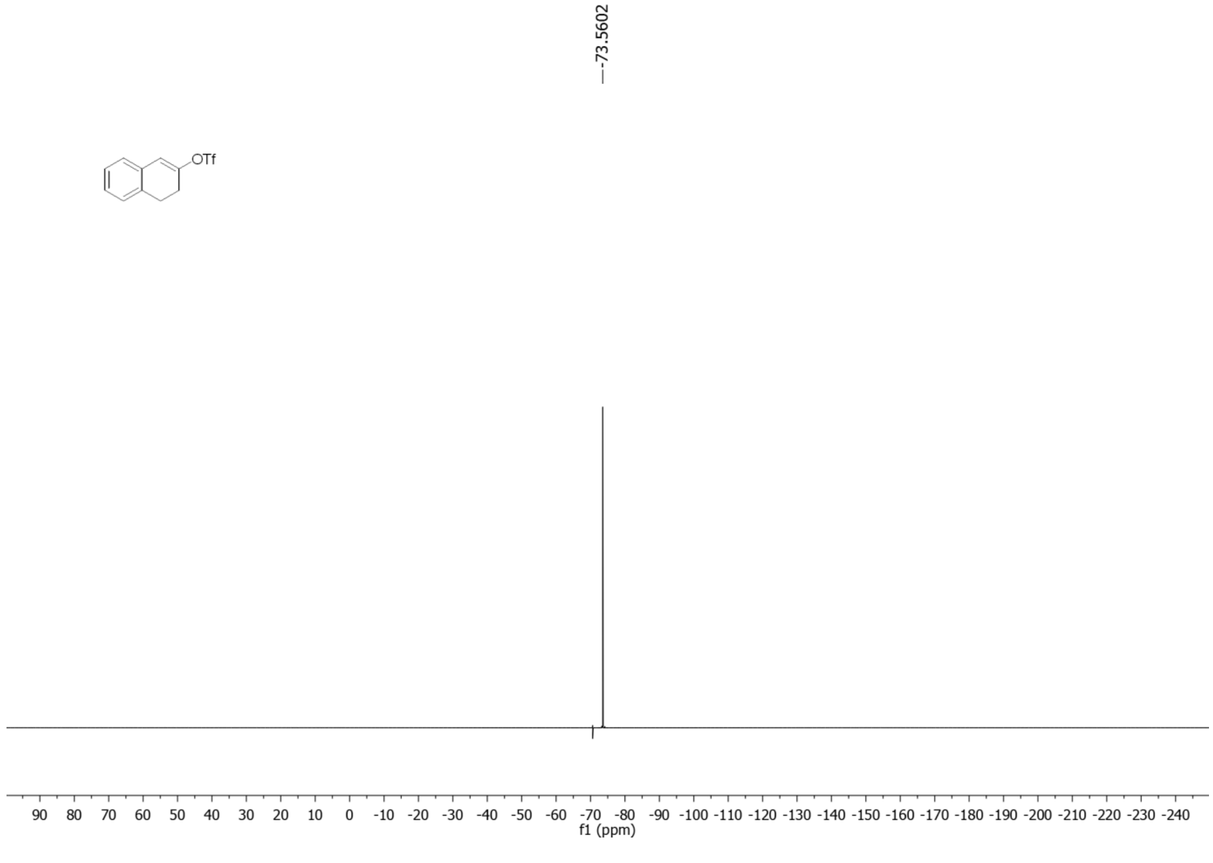

3,4-Dihydronaphthalen-1-yl trifluoromethanesulfonate (3v)

^{1}H-NMR Spectrum:

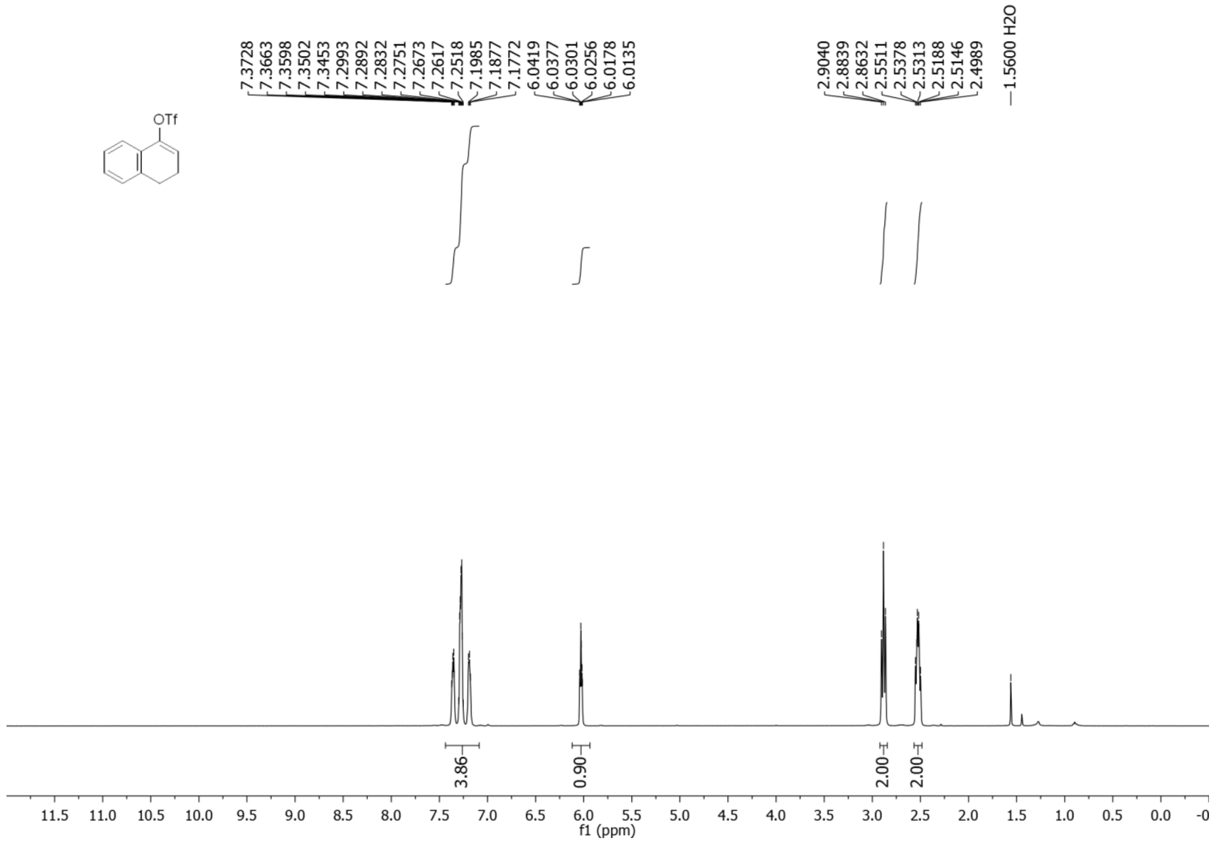

^{13}C-NMR Spectrum:

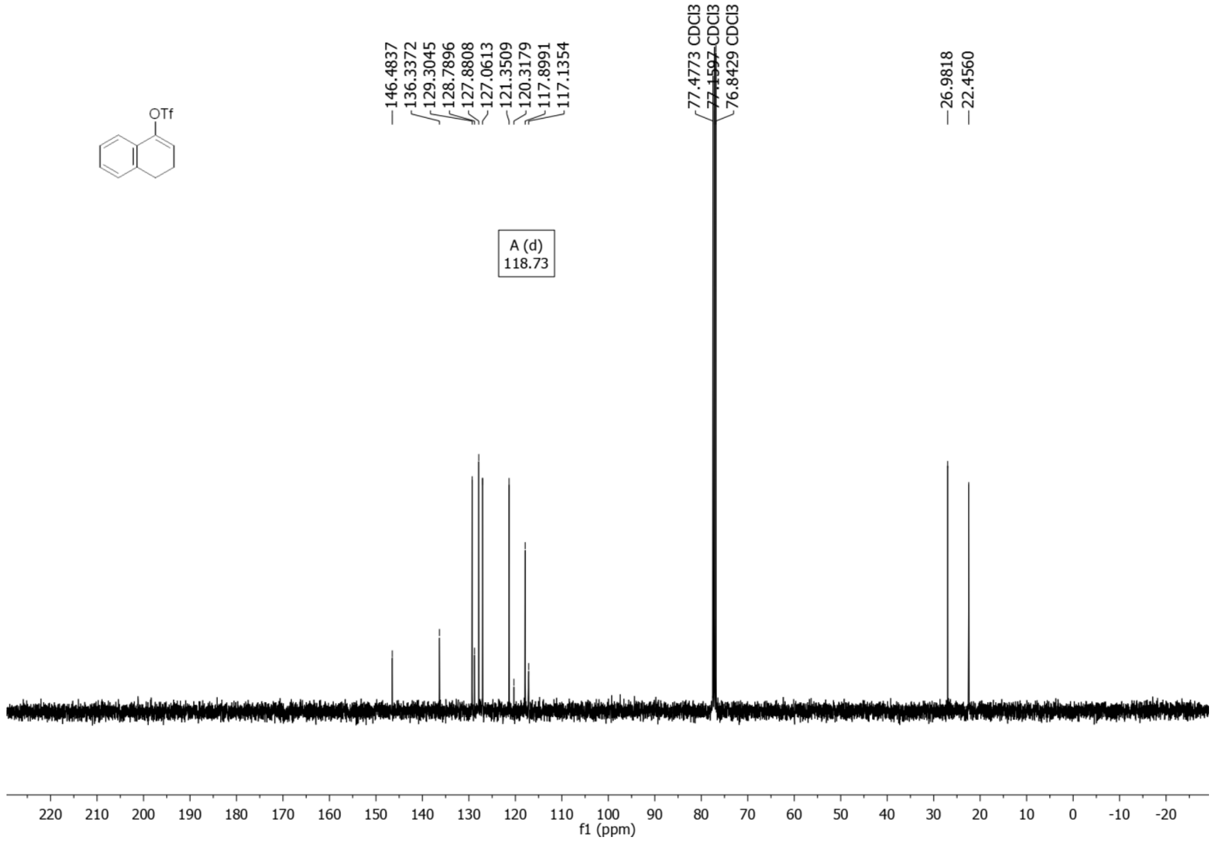

^{19}F-NMR Spectrum:

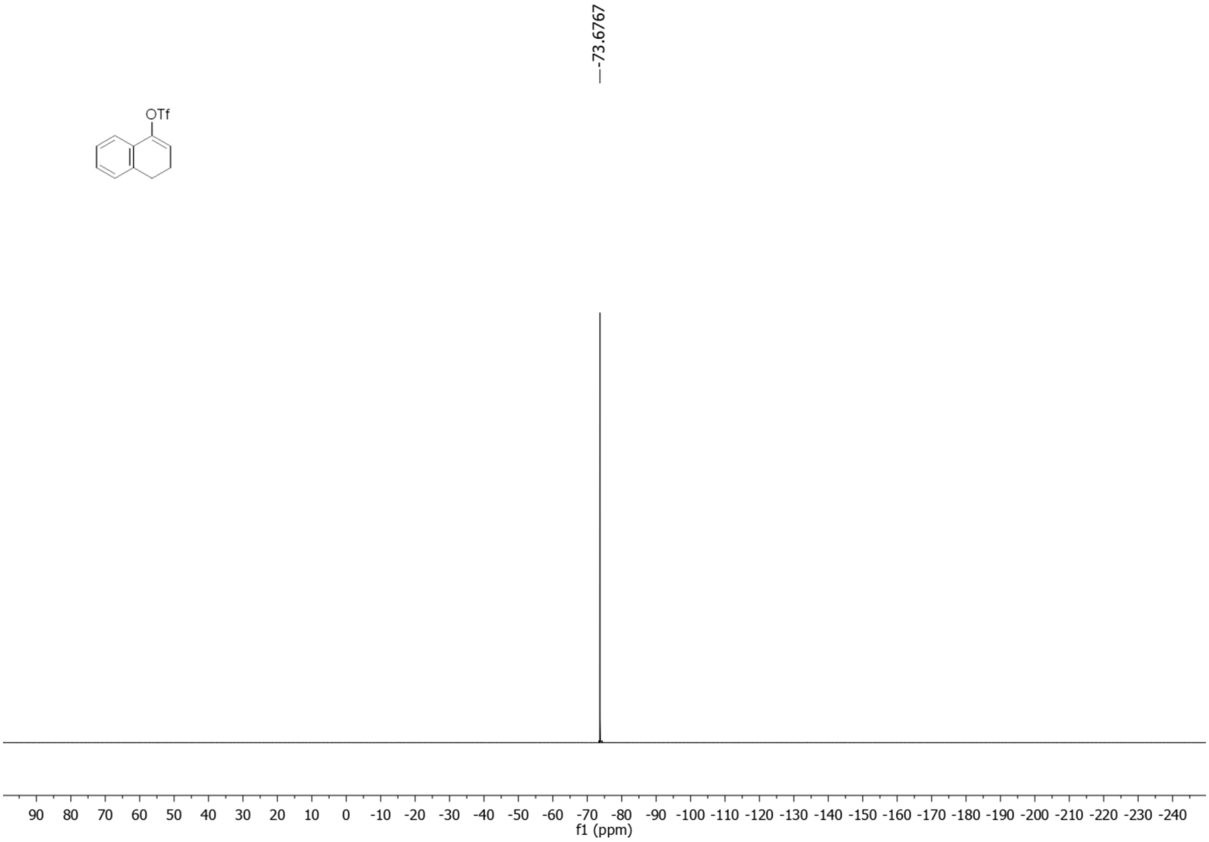

6-Methoxy-3,4-dihydronaphthalen-1-yl trifluoromethanesulfonate (3w)

^{1}H-NMR Spectrum:

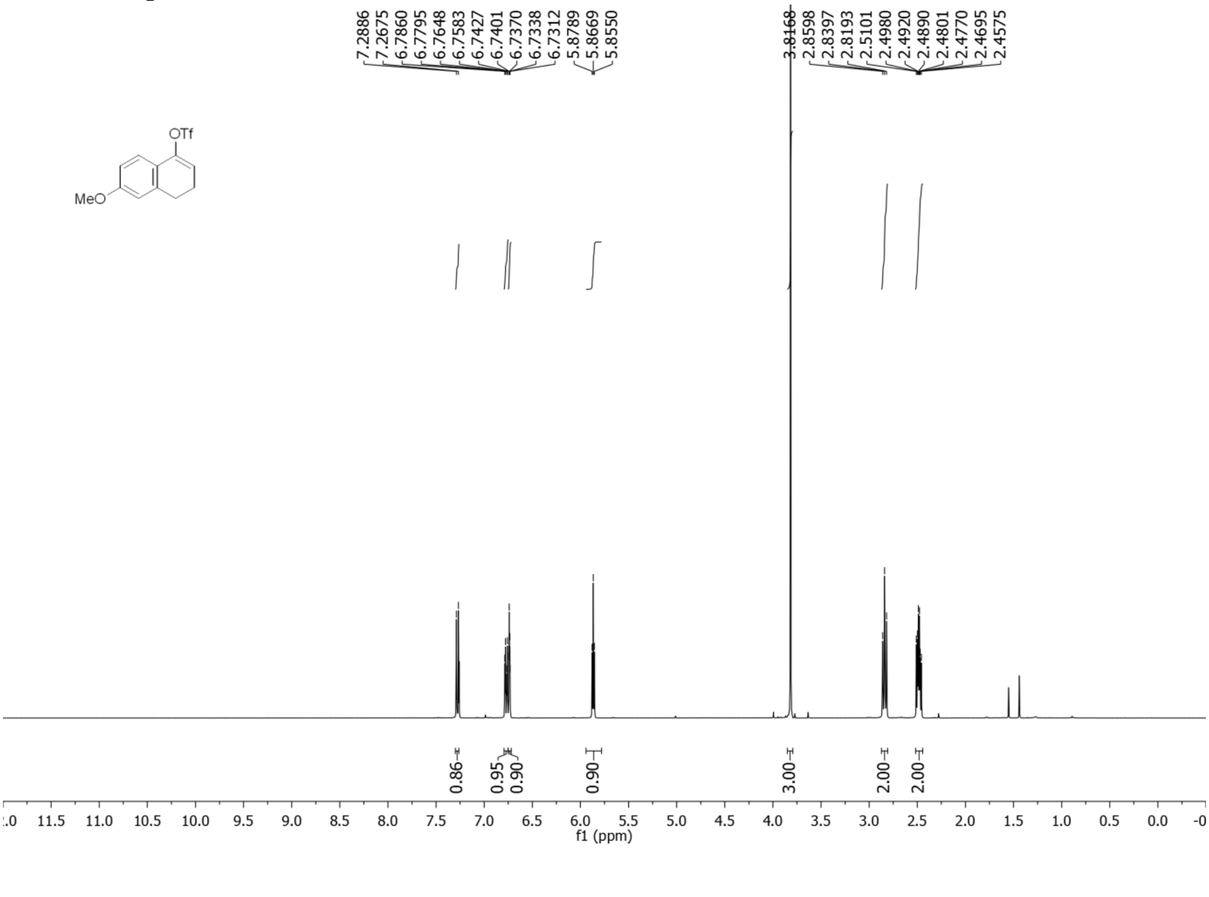

^{13}C-NMR Spectrum:

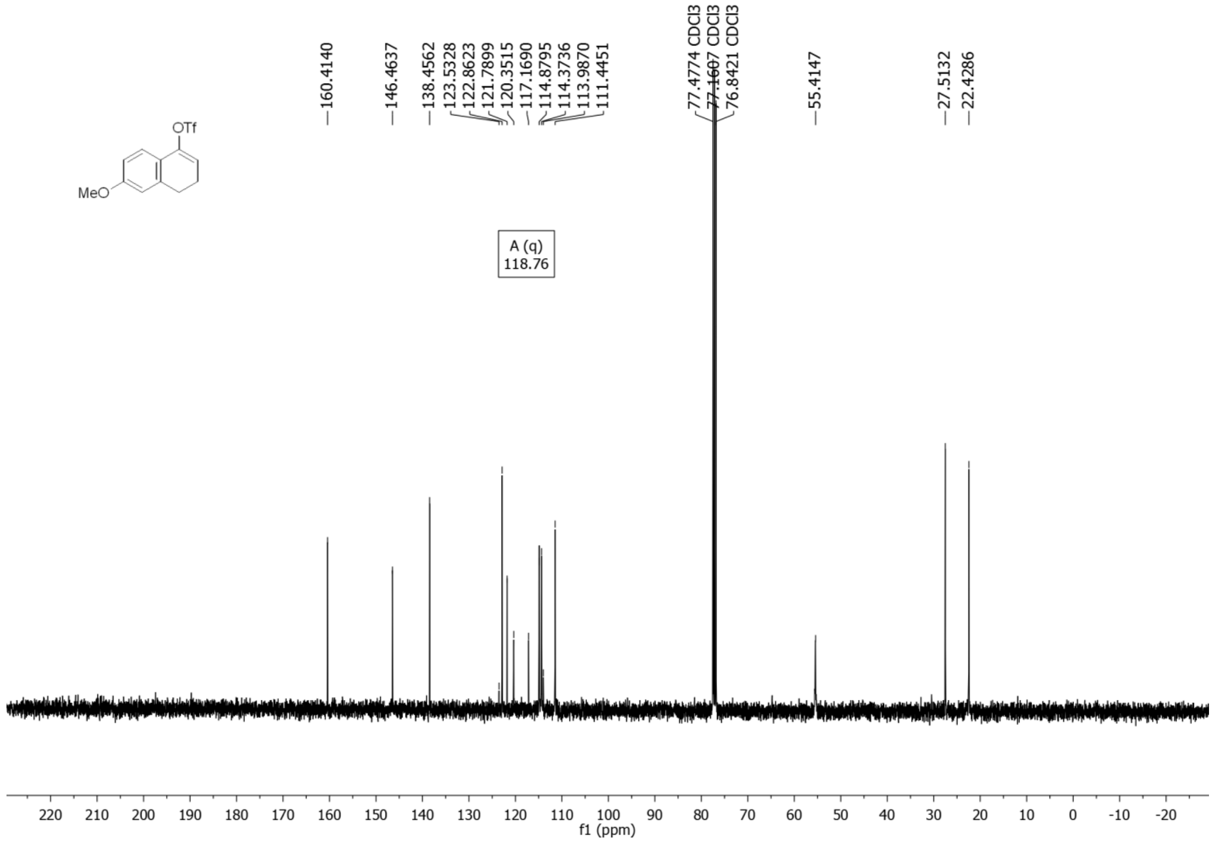

^{19}F-NMR Spectrum:

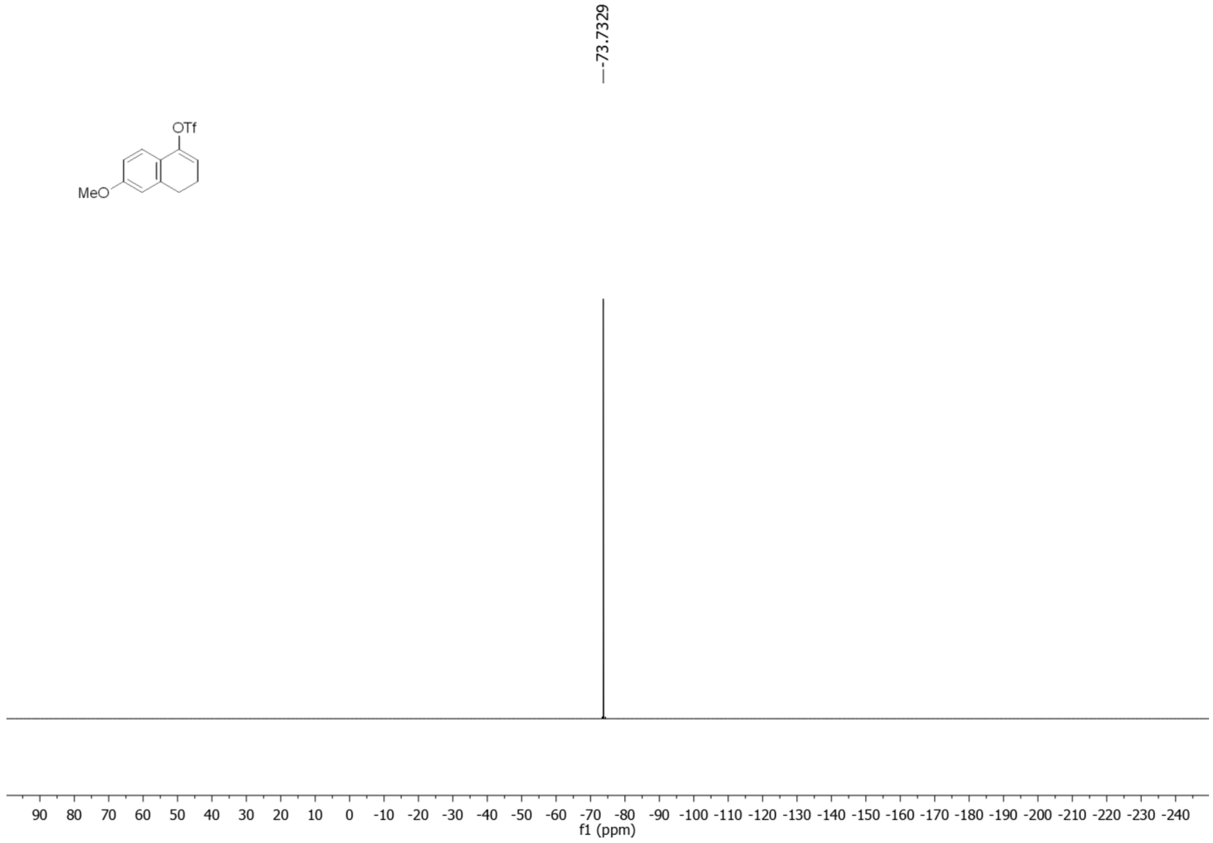

3,4-Dihydronaphthalene-1,6-diyl bis(trifluoromethanesulfonate) (3x)

^{1}H-NMR Spectrum:

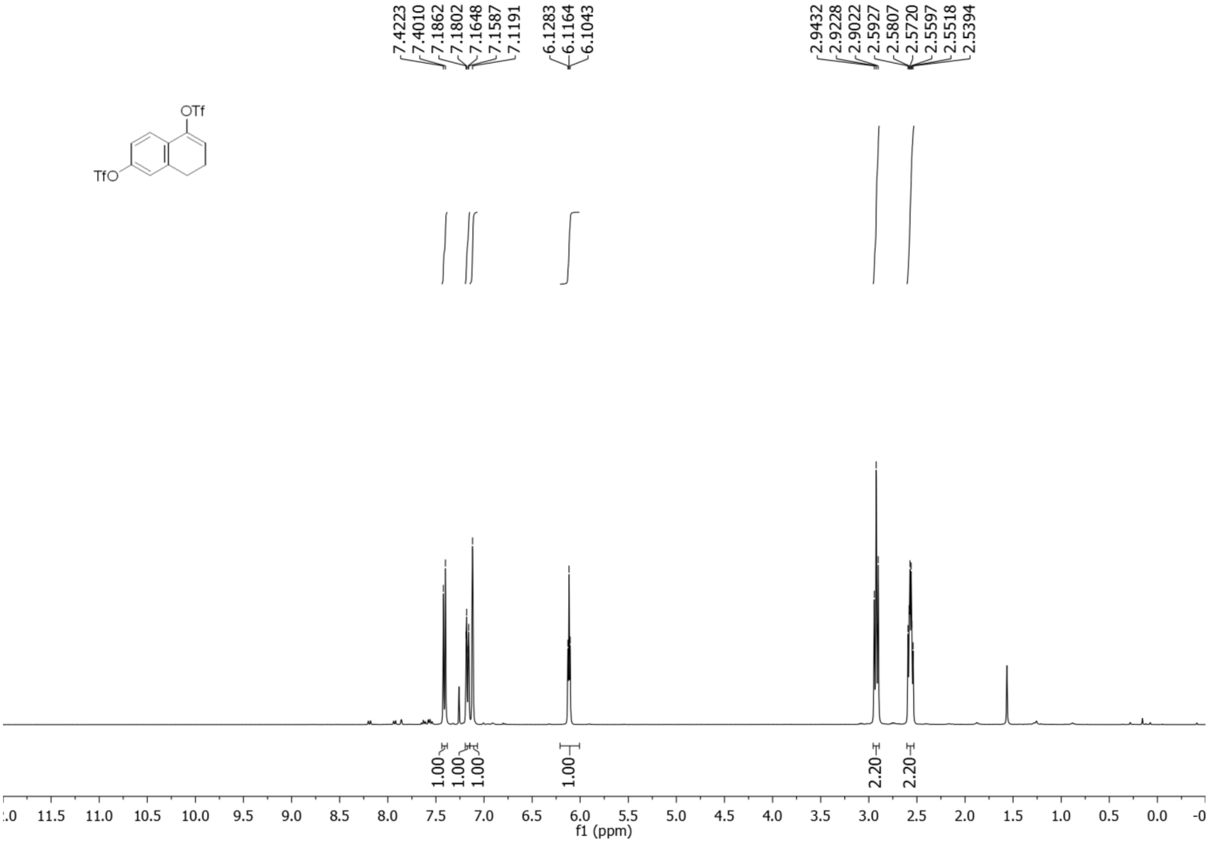

^{13}C-NMR Spectrum:

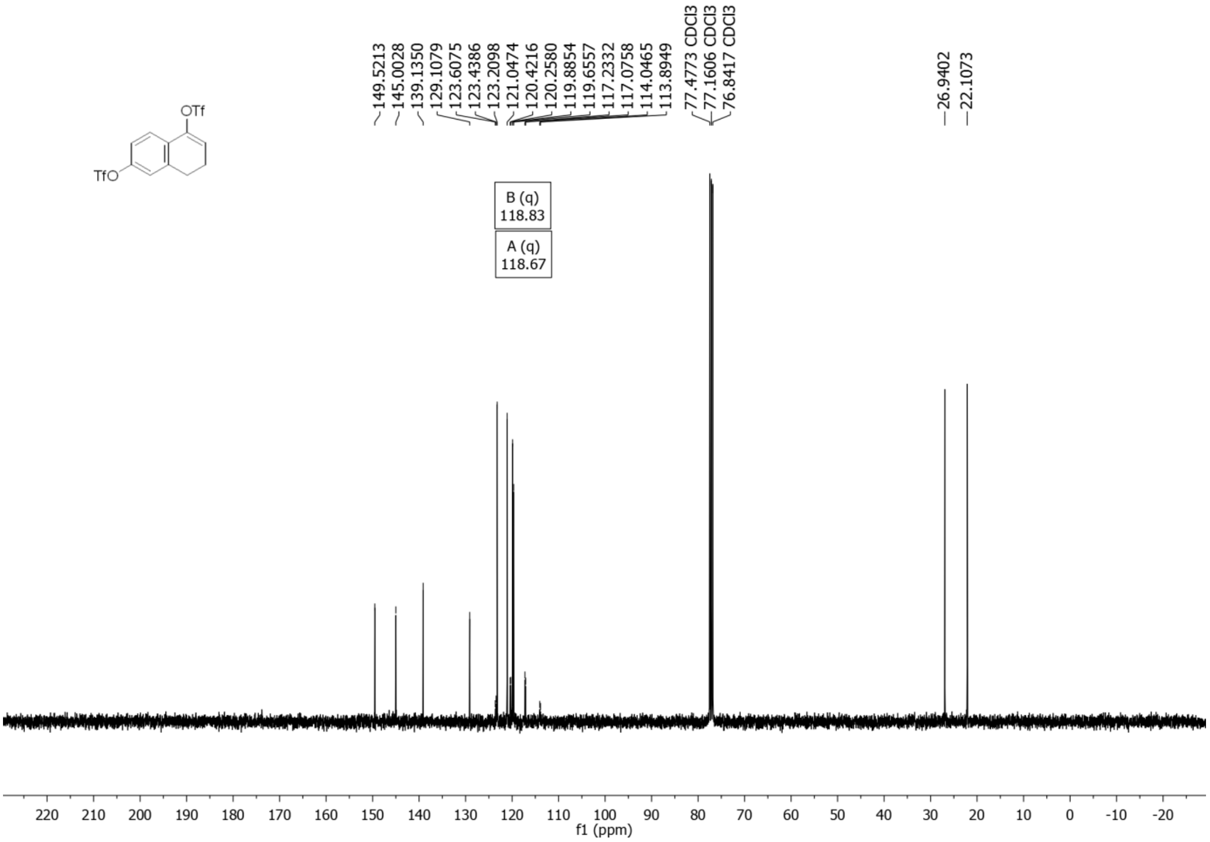

^{19}F-NMR Spectrum:

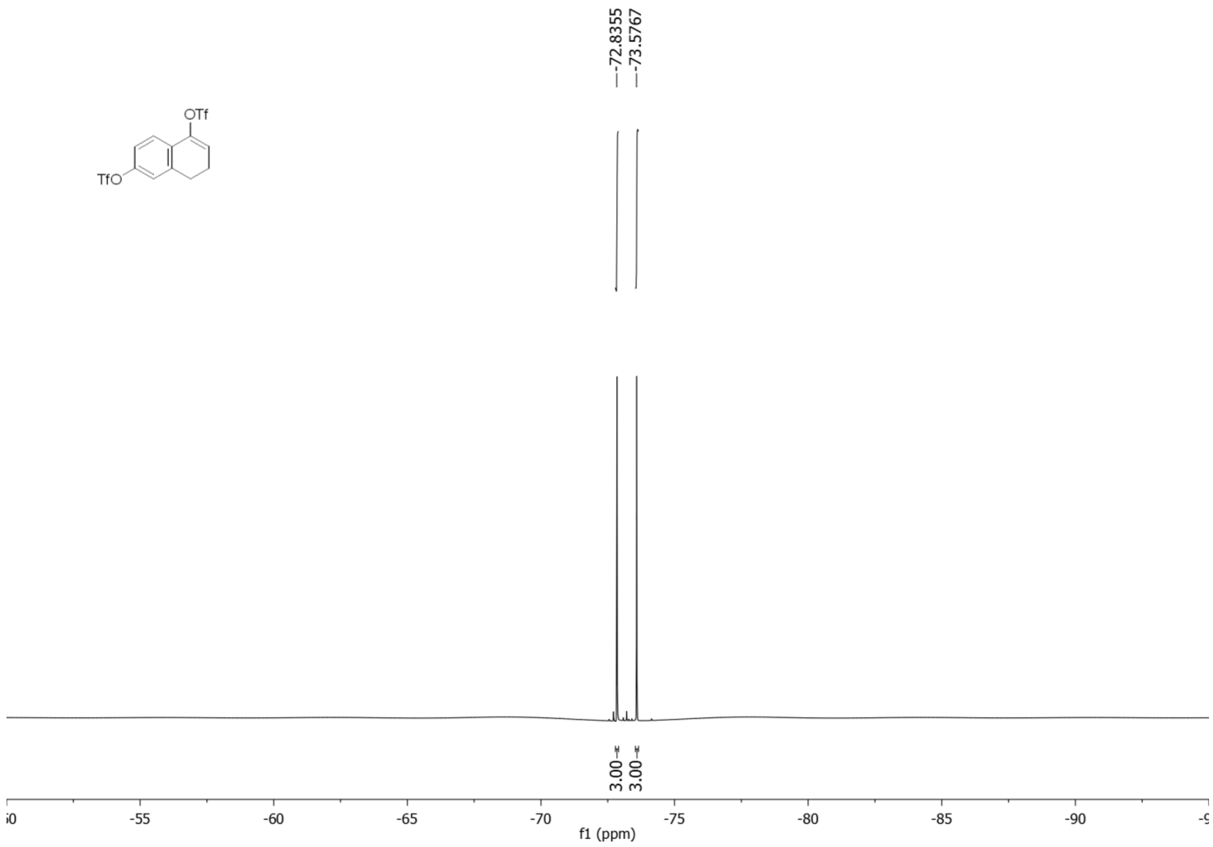

5 NMR Spectra of Alkenylsulfonyl Fluorides Synthesized by Palladium Catalysis

Cyclohept-1-ene-1-sulfonyl fluoride (5a)

^{1}H-NMR Spectrum:

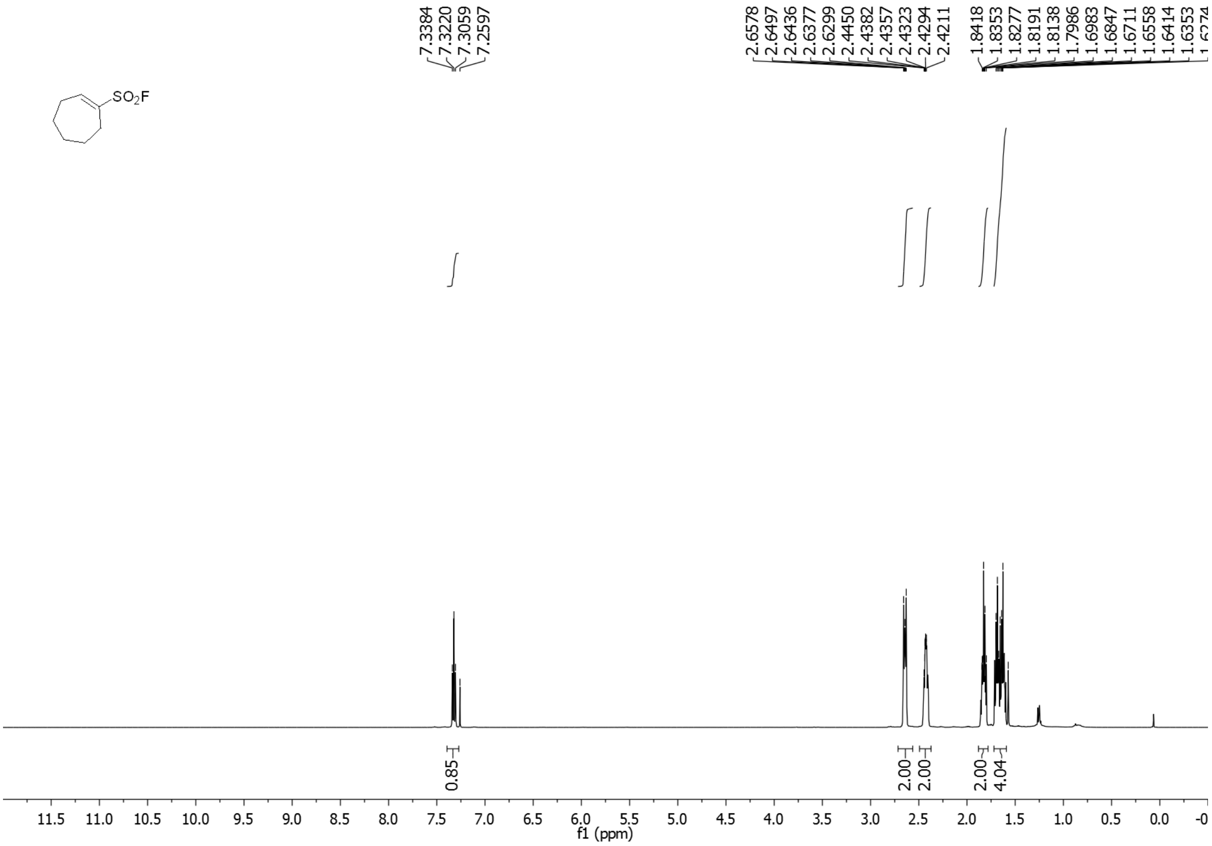

^{13}C-NMR Spectrum:

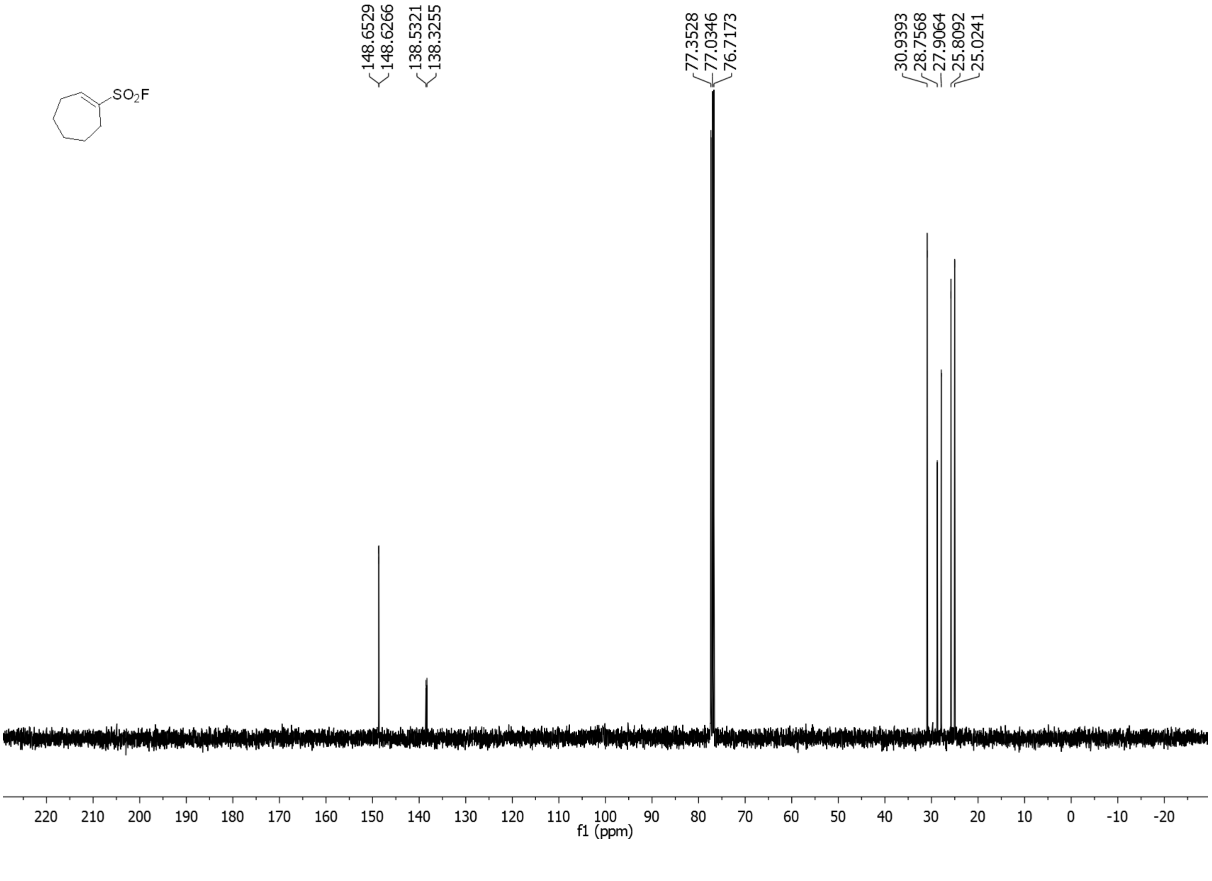

^{19}F-NMR Spectrum:

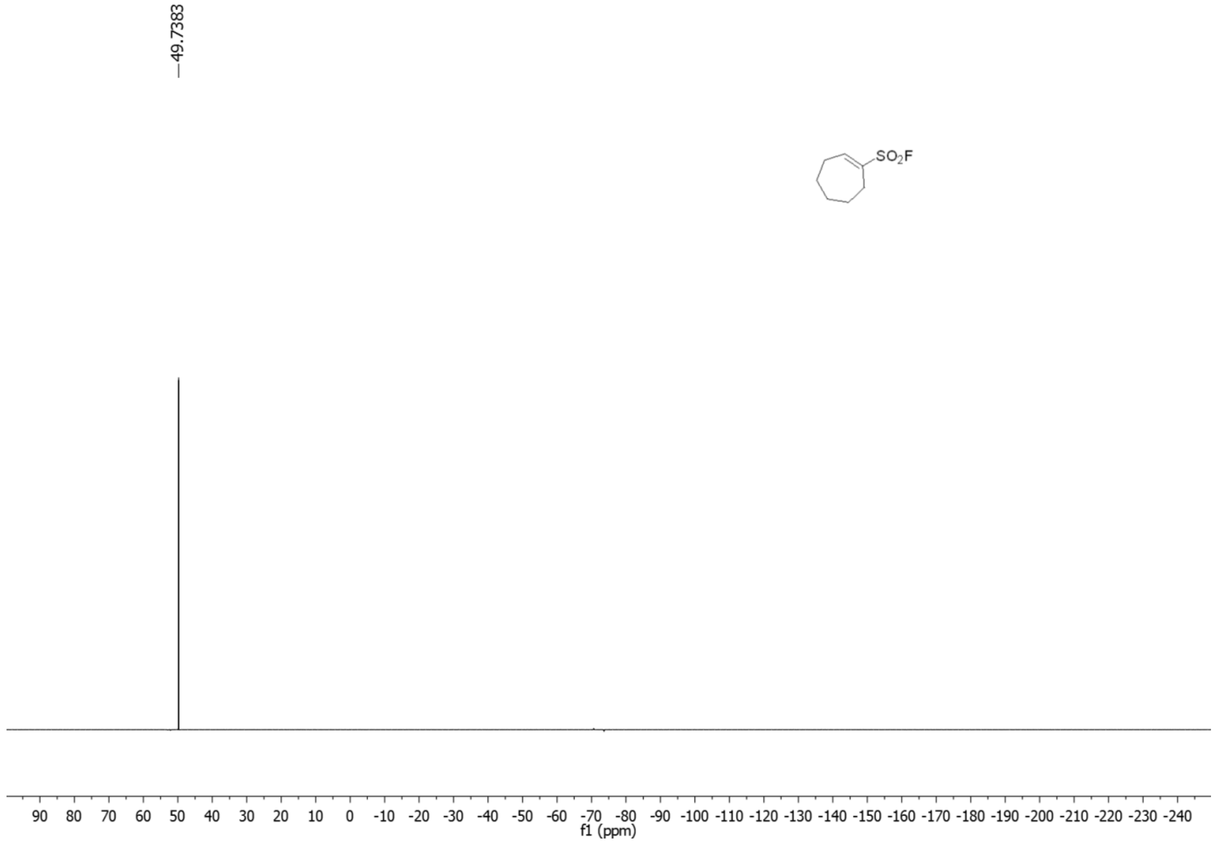

1,2,3,6-Tetrahydro-[1,1'-biphenyl]-4-sulfonyl fluoride (5b)

^{1}H-NMR Spectrum:

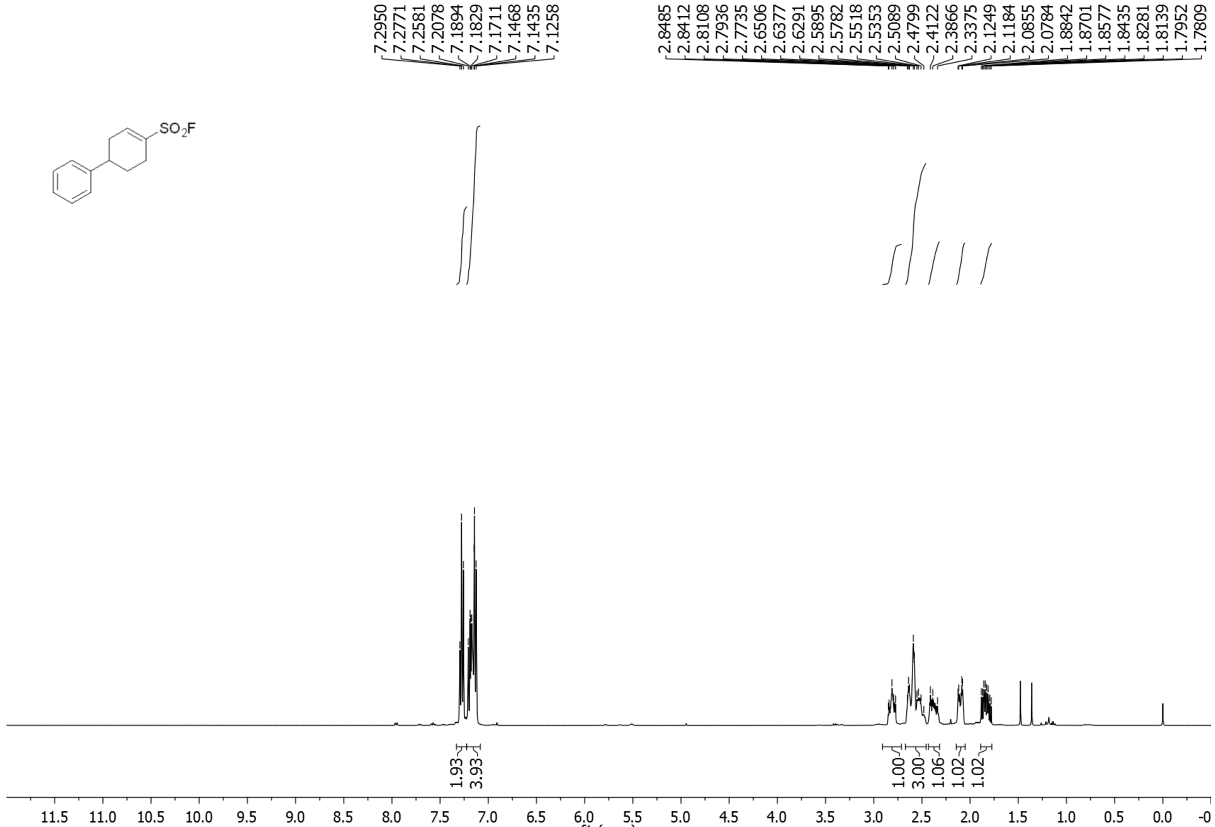

^{13}C-NMR Spectrum:

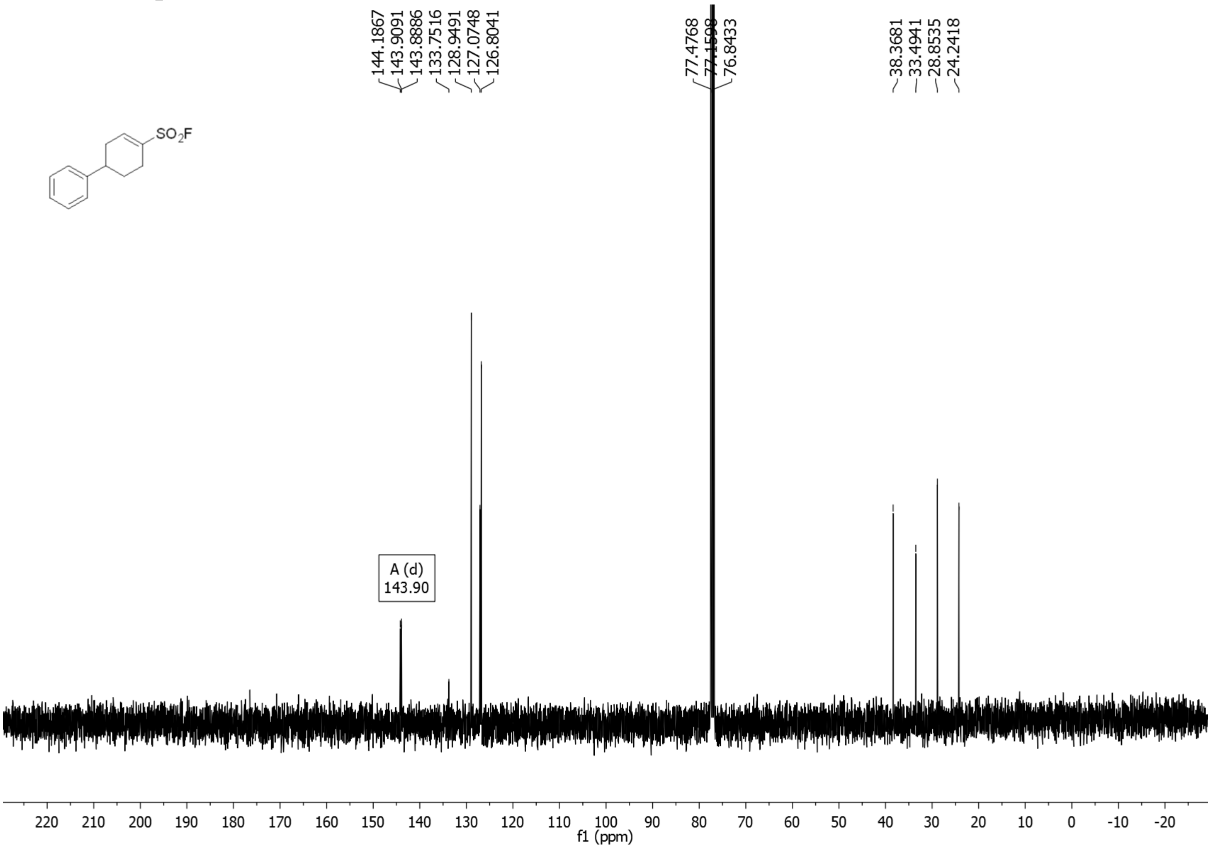

^{19}F-NMR Spectrum:

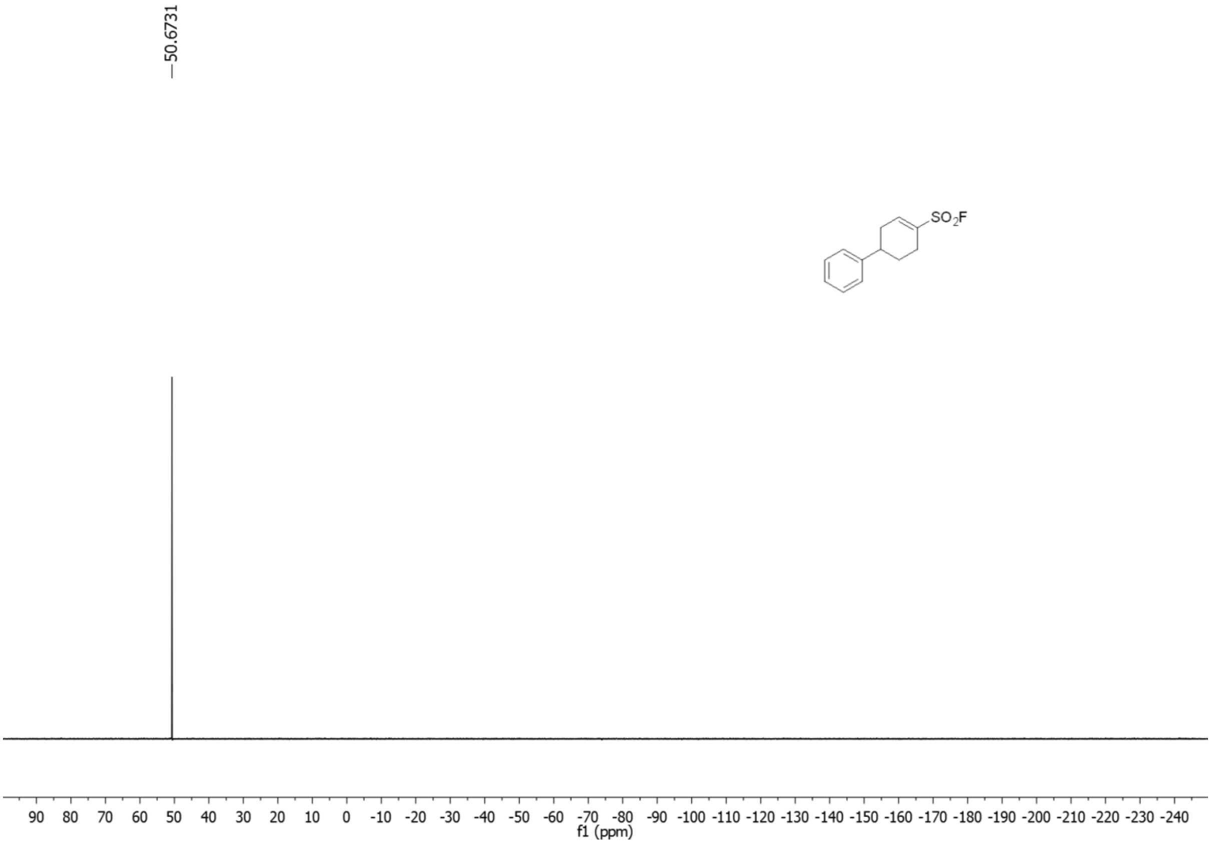

4-(*tert*-Butyl)cyclohex-1-ene-1-sulfonyl fluoride (5c)

^{1}H-NMR Spectrum:

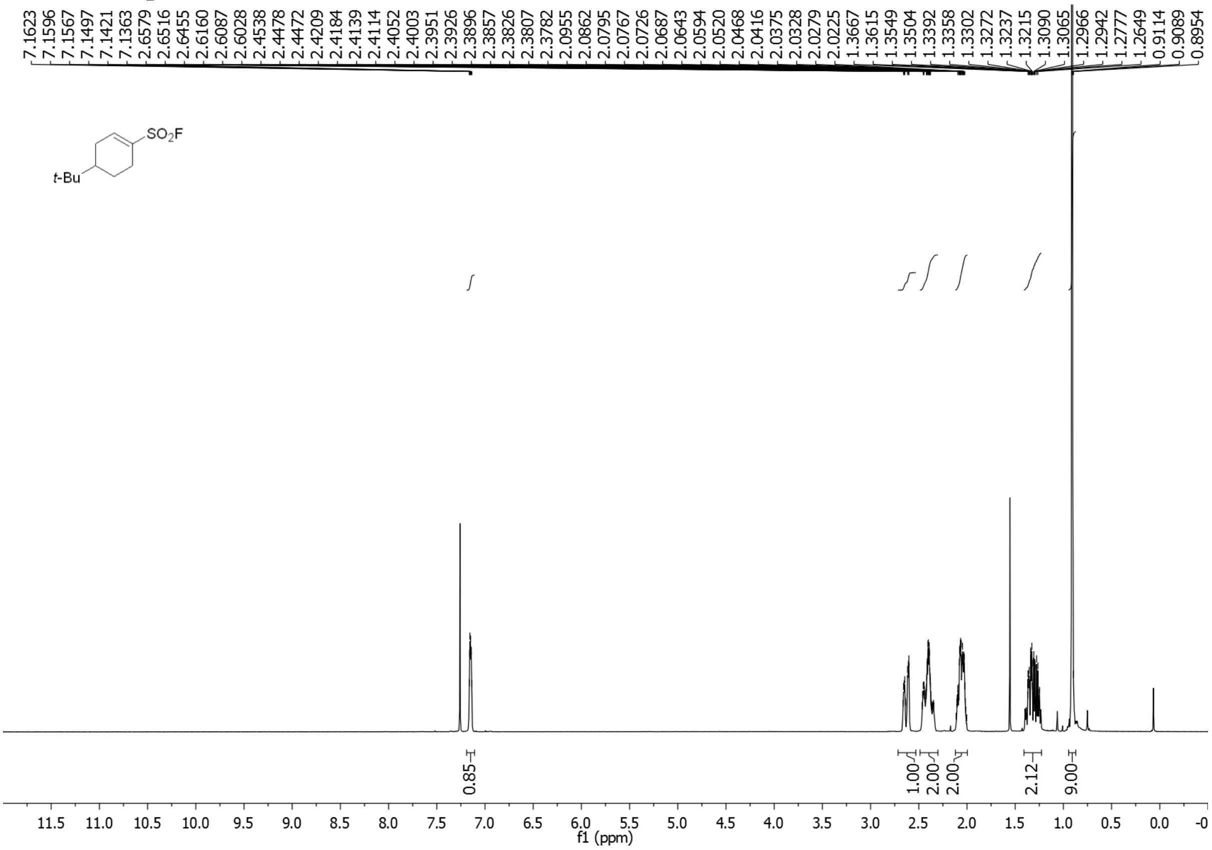

^{13}C-NMR Spectrum:

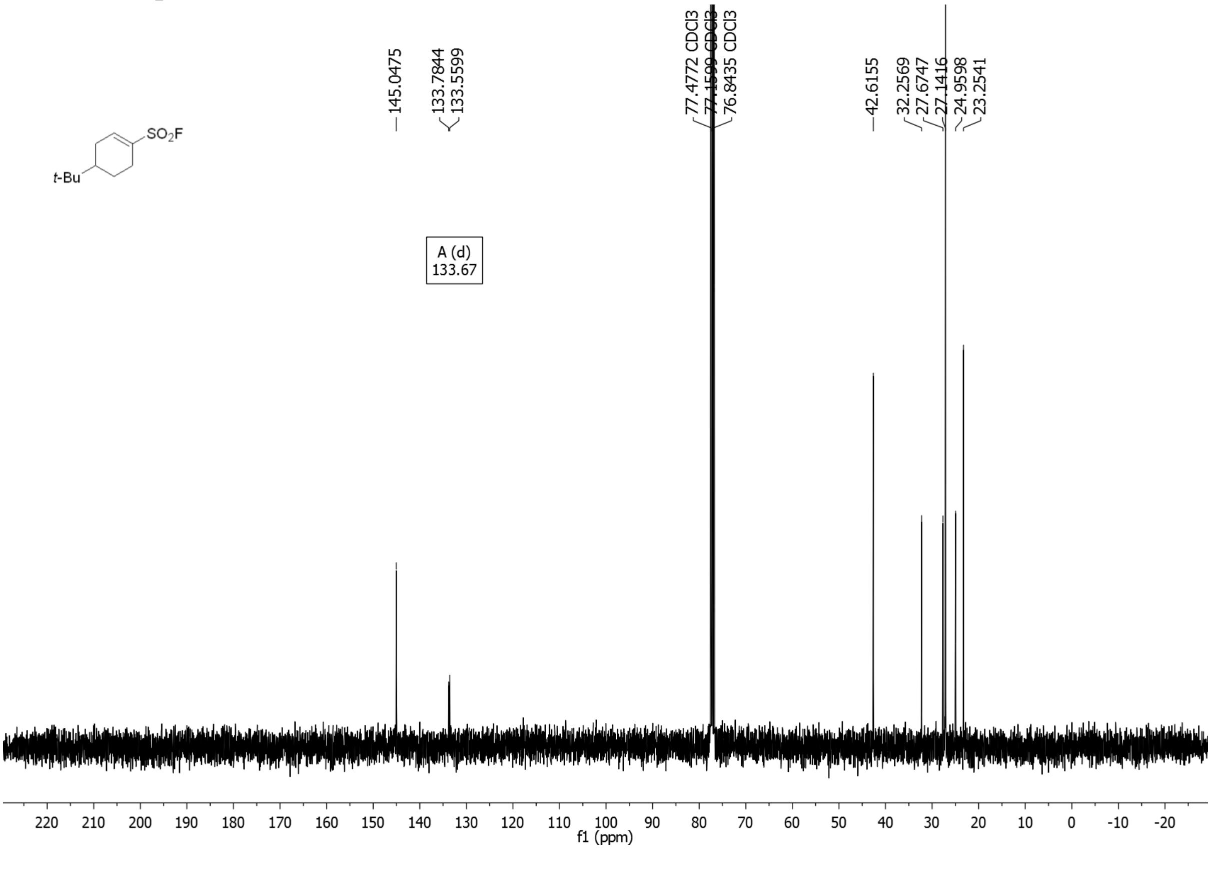

^{19}F-NMR Spectrum:

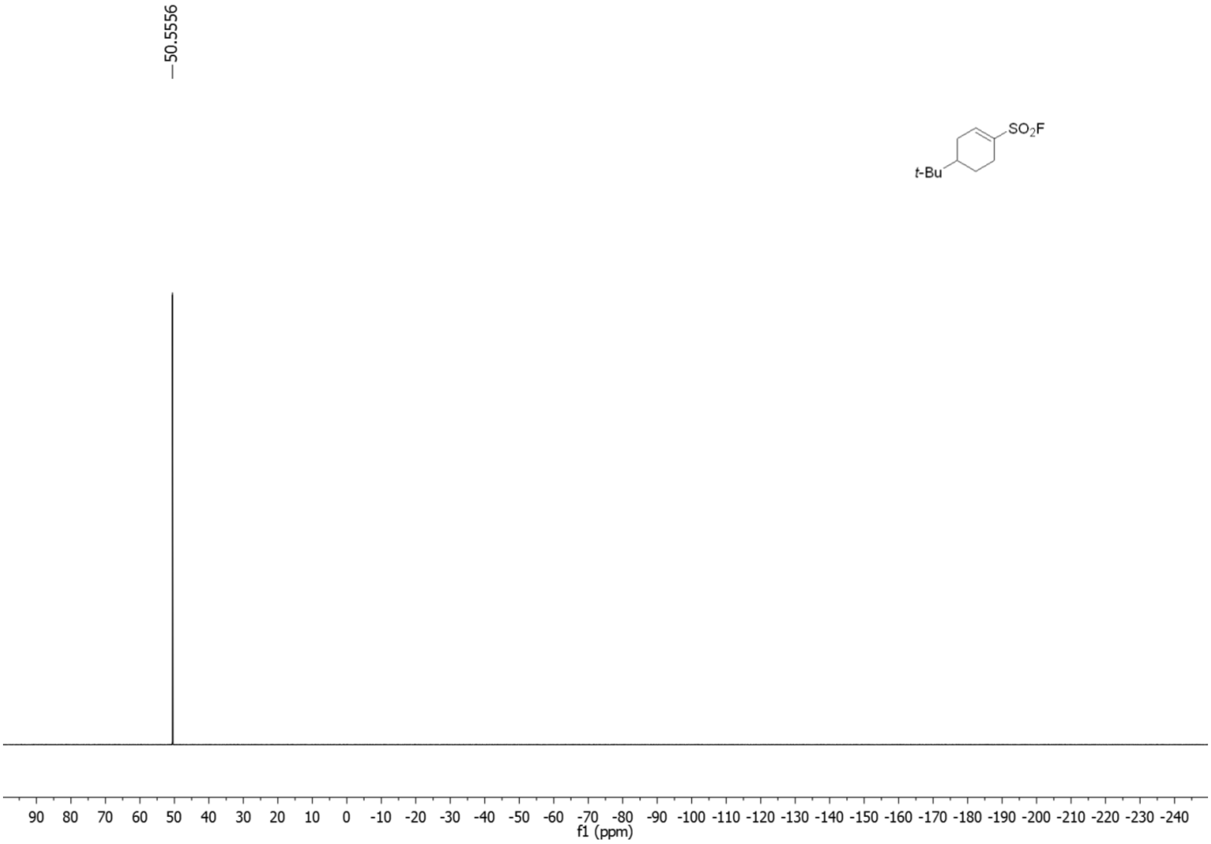

4,4-Difluorocyclohex-1-ene-1-sulfonyl fluoride (5d)

^{1}H-NMR Spectrum:

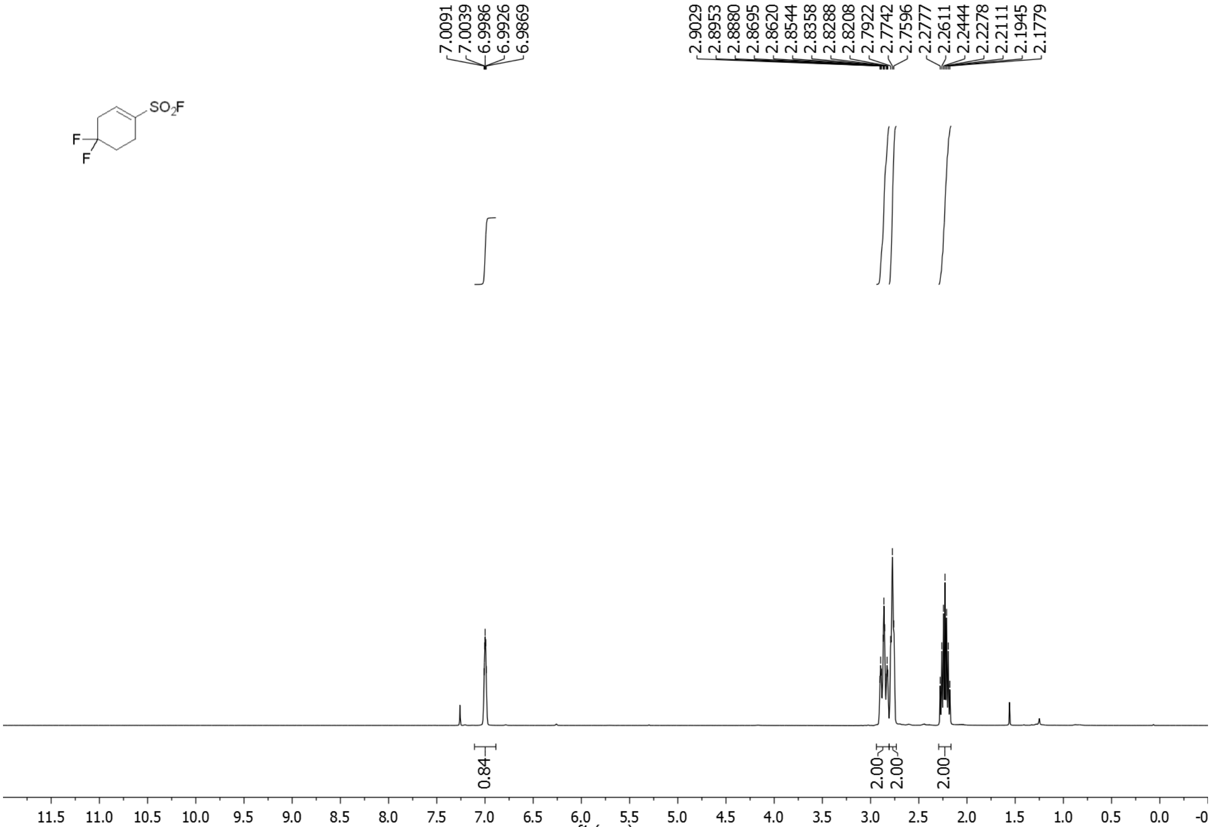

^{13}C-NMR Spectrum:

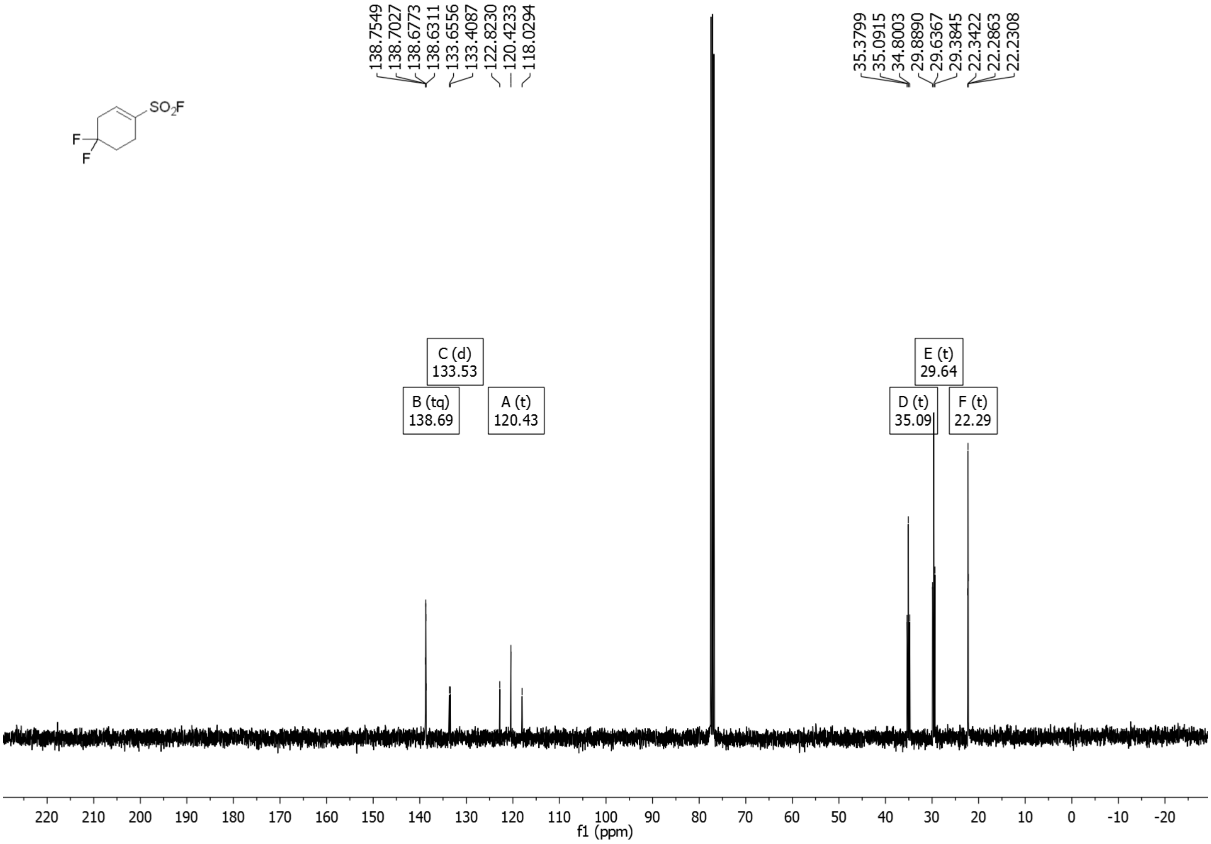

^{19}F-NMR Spectrum:

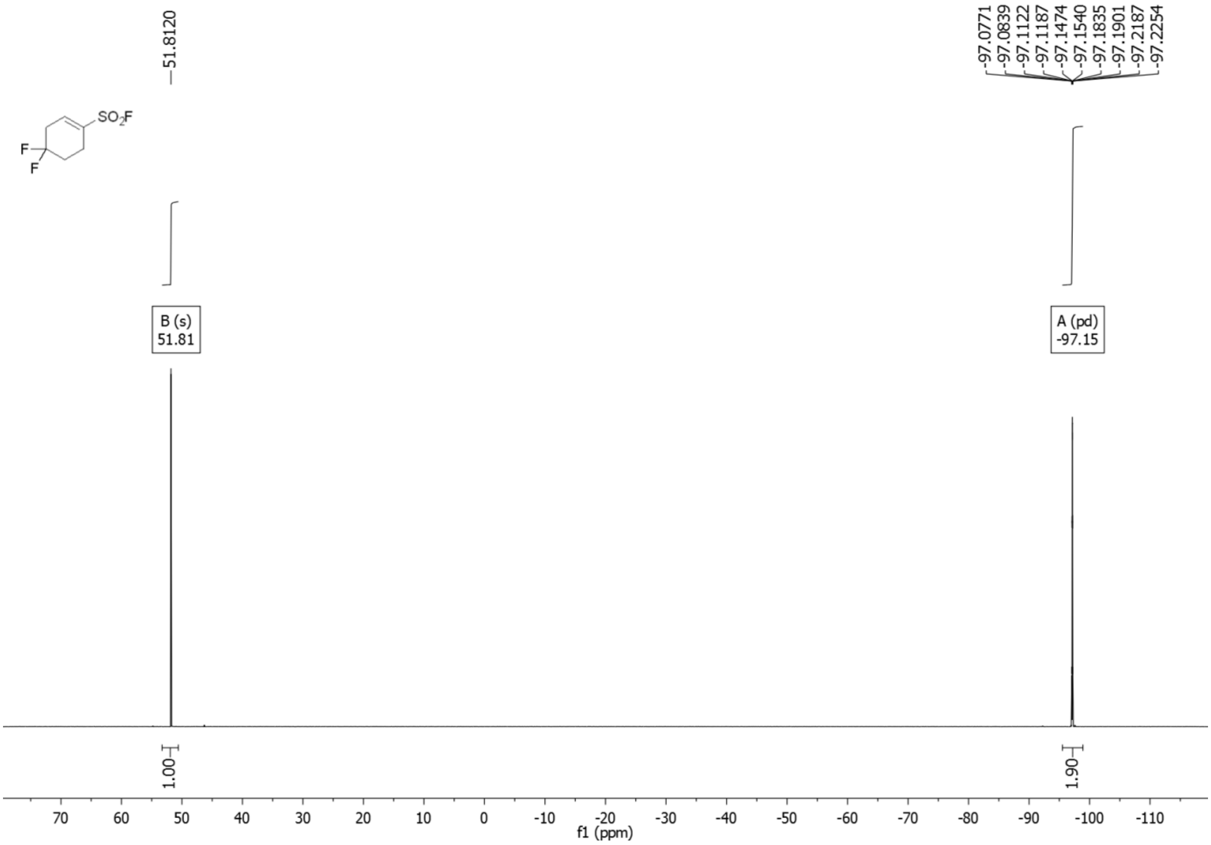

Ethyl 4-(fluorosulfonyl)cyclohex-3-ene-1-carboxylate (5e)

^{1}H-NMR Spectrum:

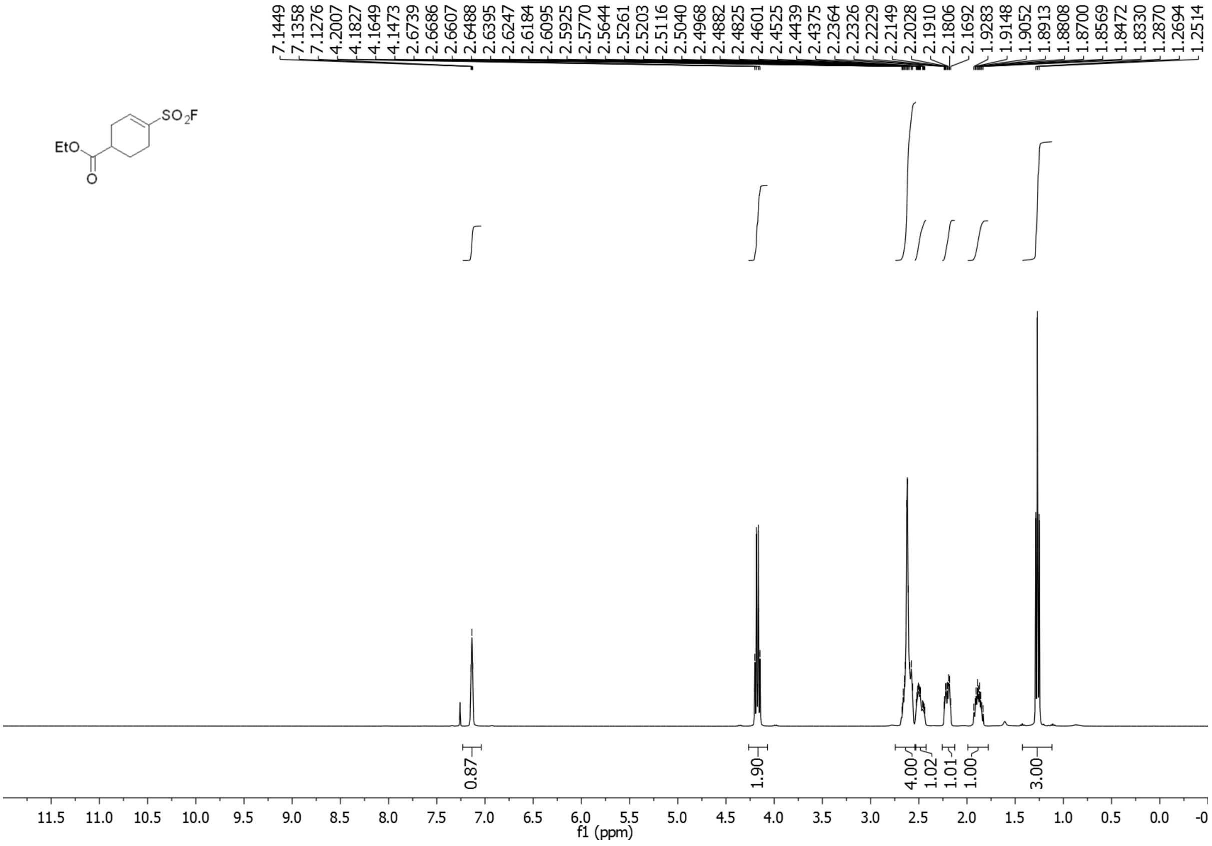

^{13}C-NMR Spectrum:

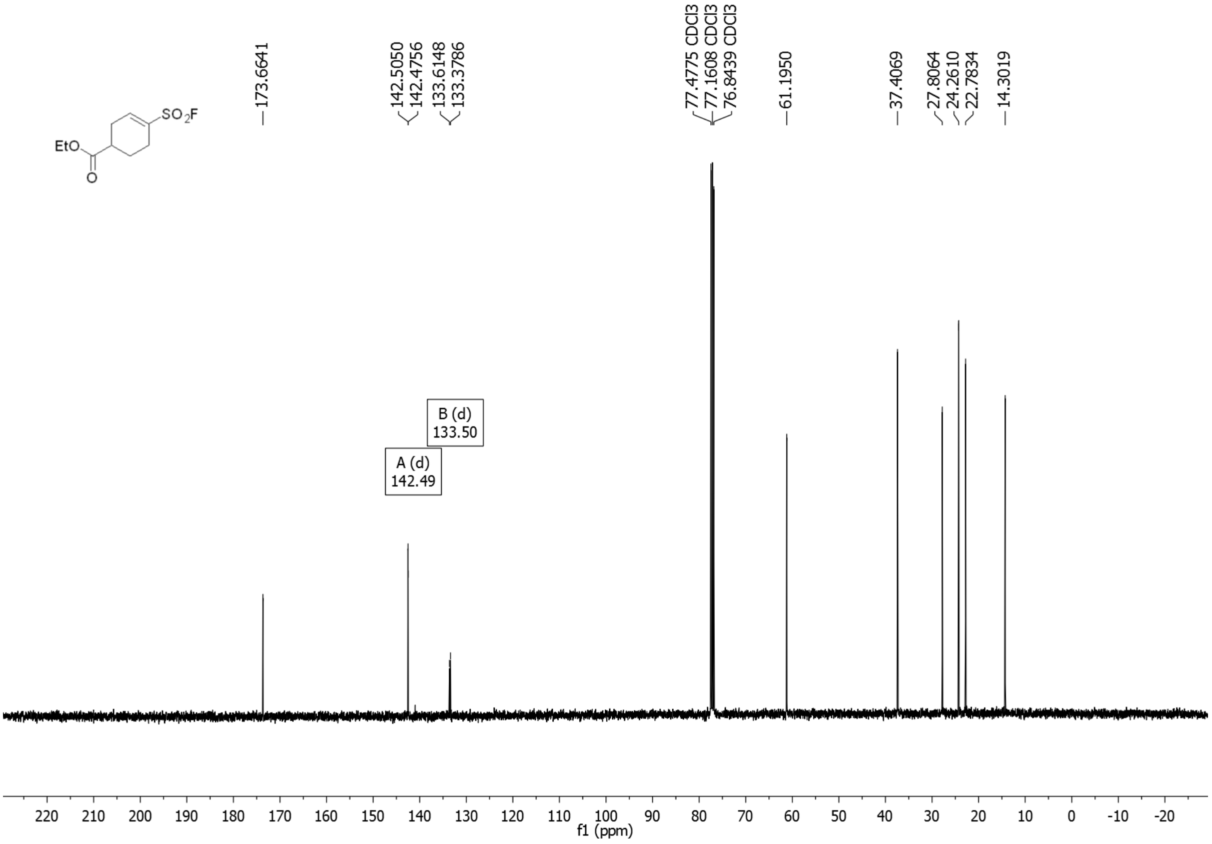

^{19}F-NMR Spectrum:

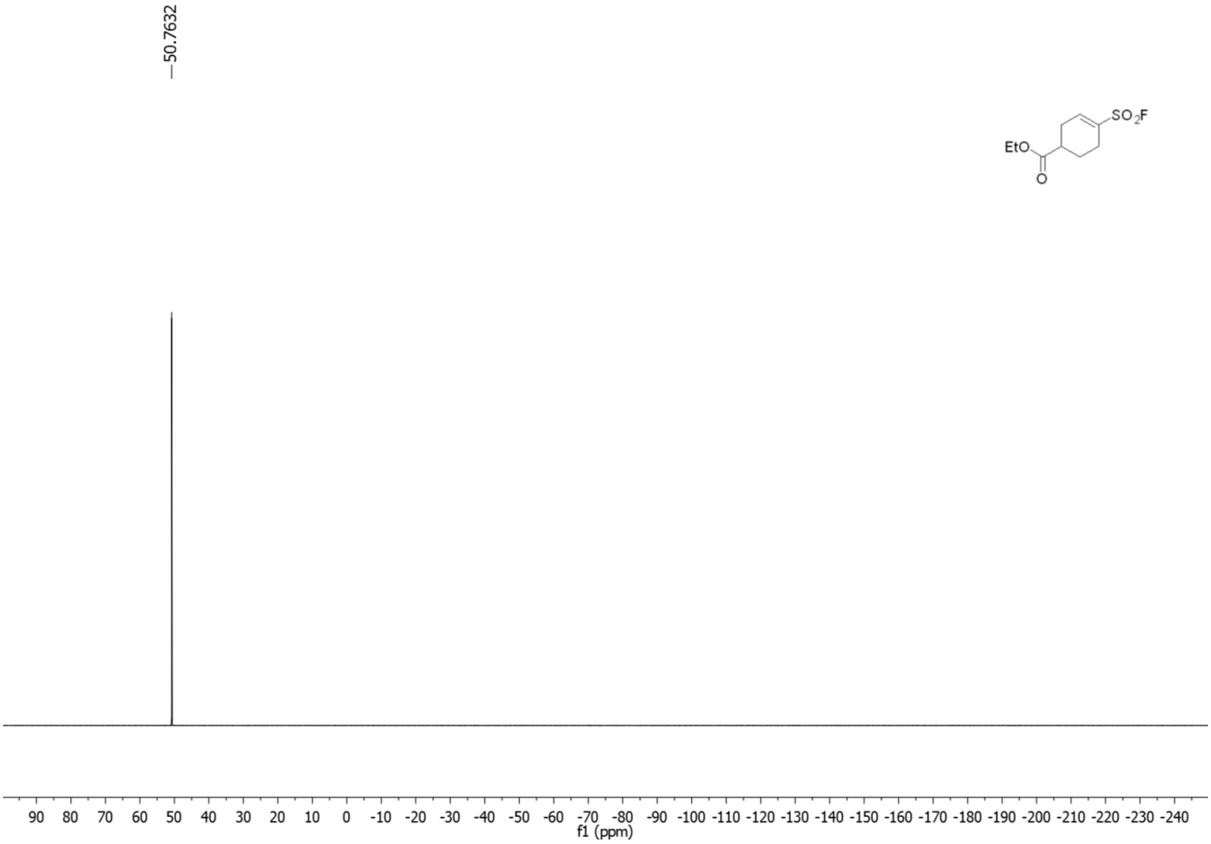

4-Methoxycyclohex-1-ene-1-sulfonyl fluoride (5f)

^{1}H-NMR Spectrum:

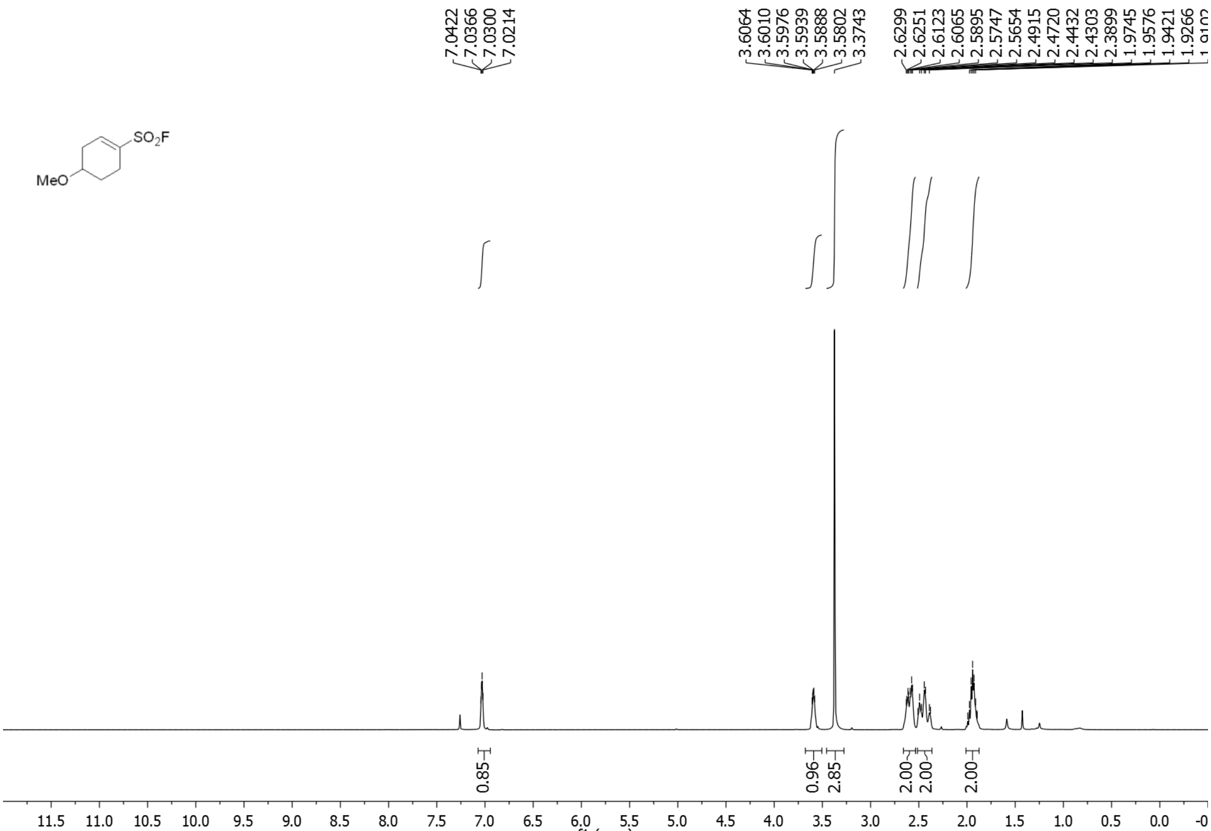

^{13}C-NMR Spectrum:

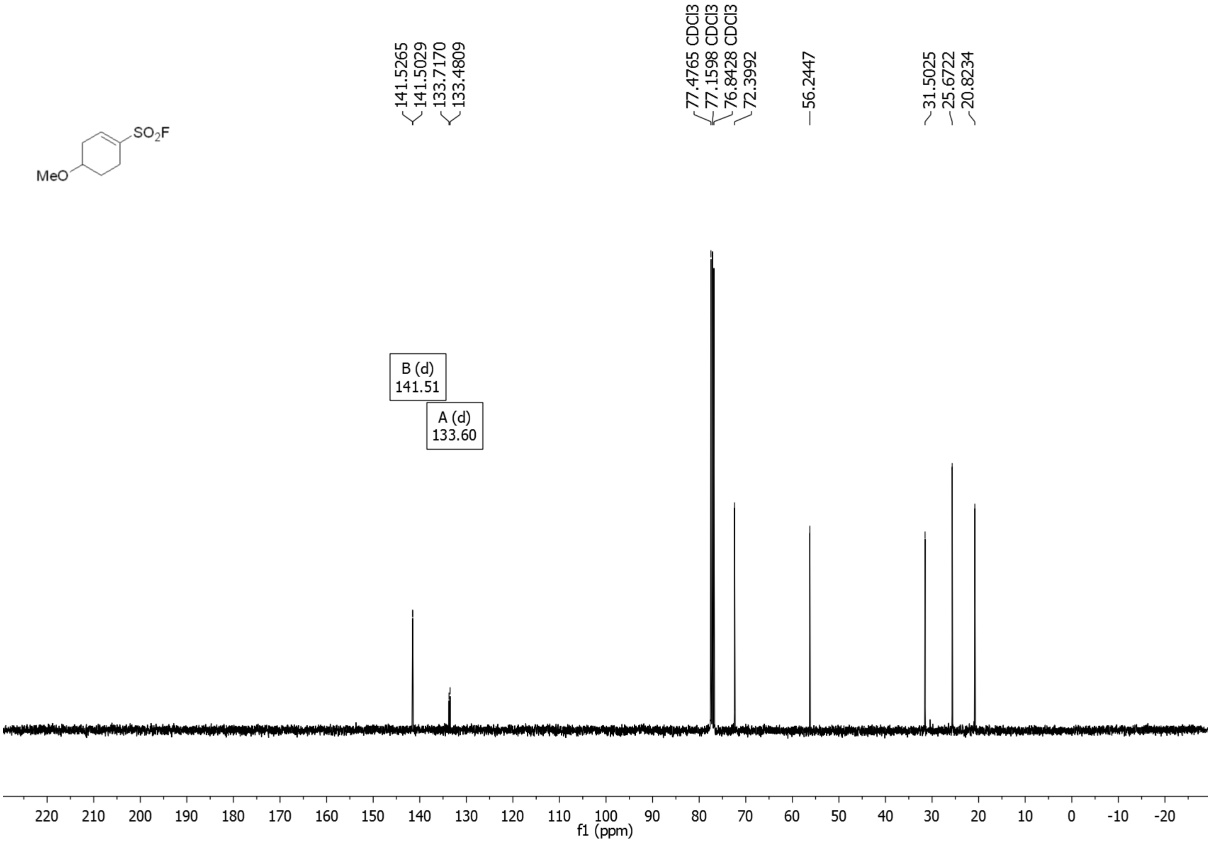

^{19}F-NMR Spectrum:

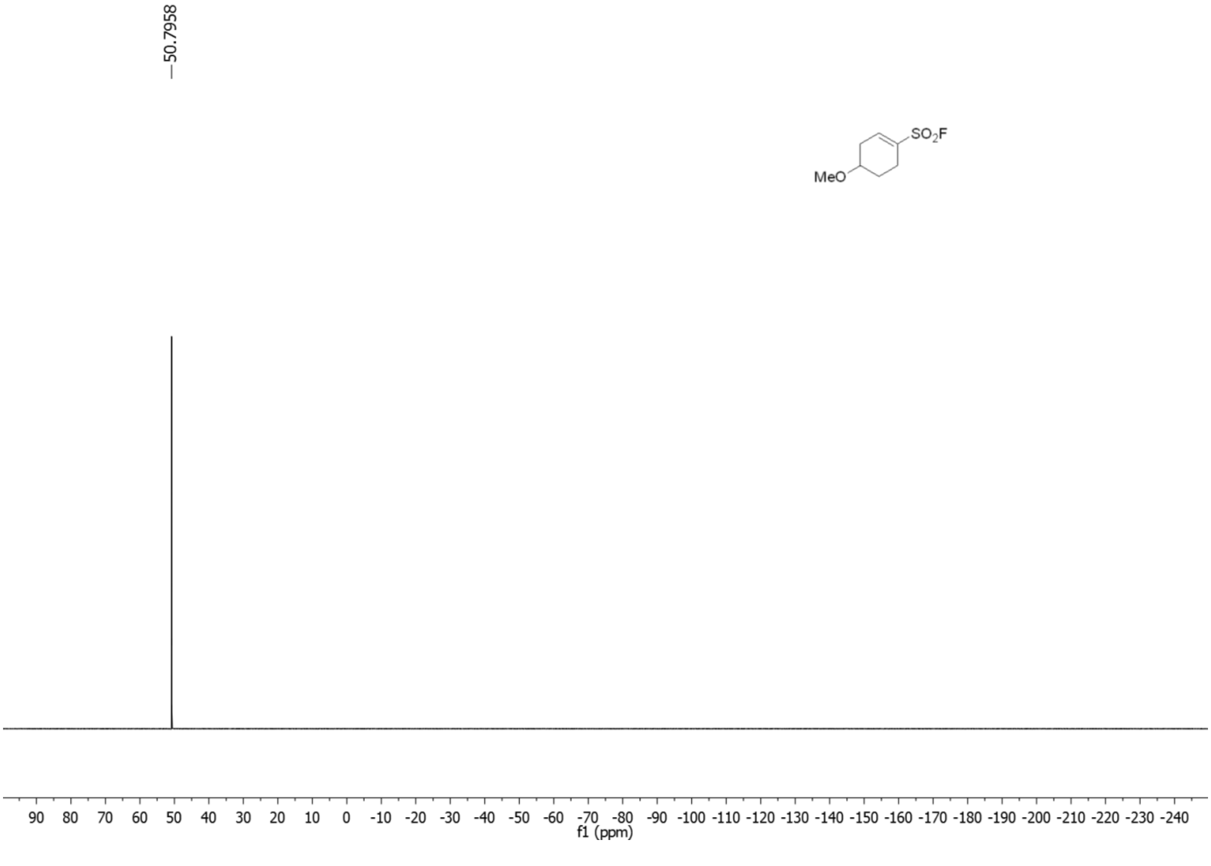

***tert*-Butyl (4-(fluorosulfonyl)cyclohex-3-en-1-yl)carbamate (5g)**

^{1}H-NMR Spectrum:

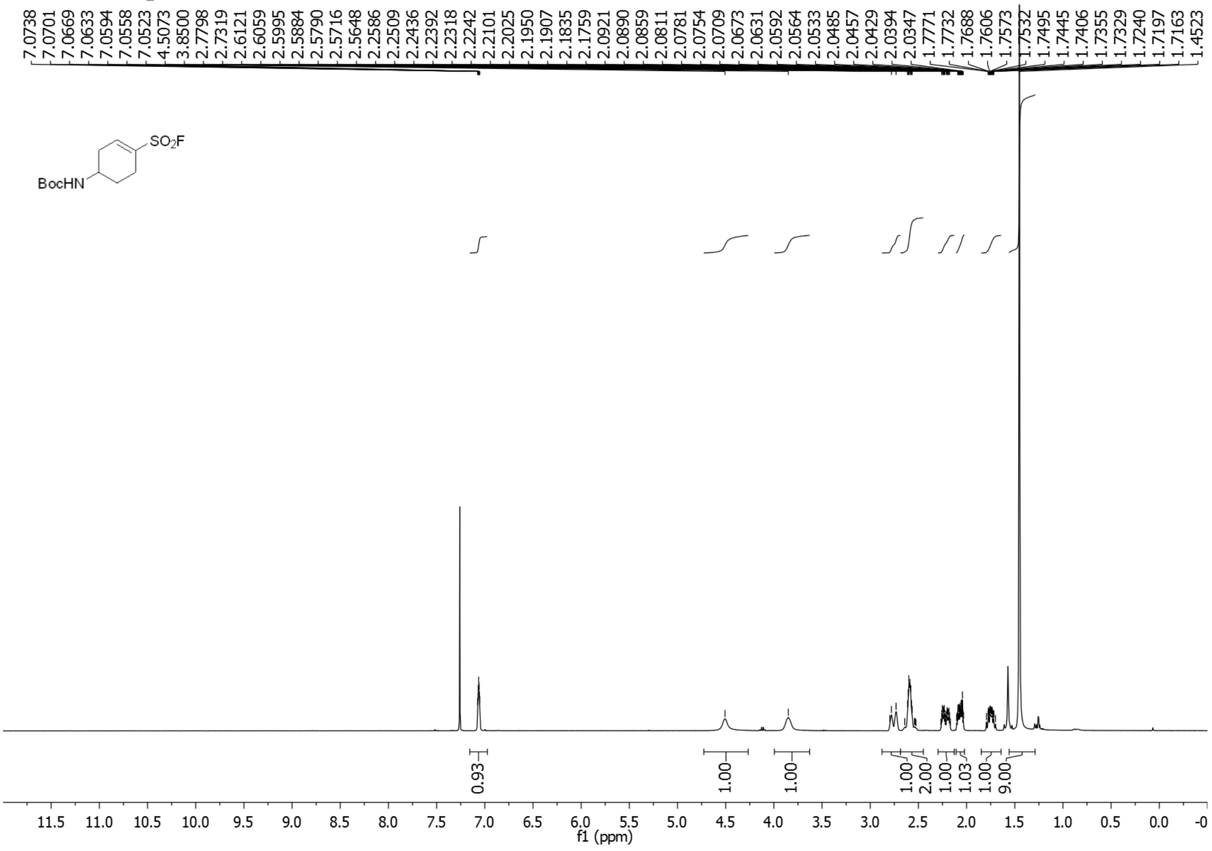

^{13}C-NMR Spectrum:

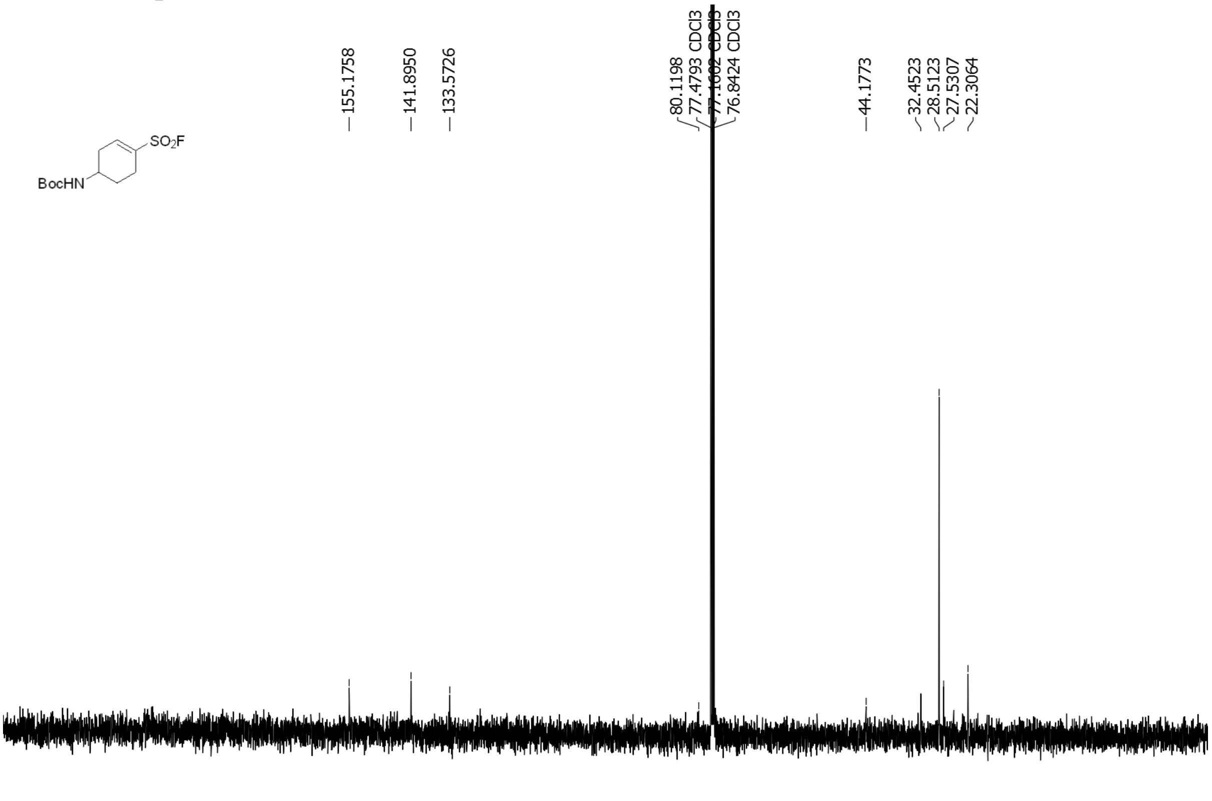

^{19}F-NMR Spectrum:

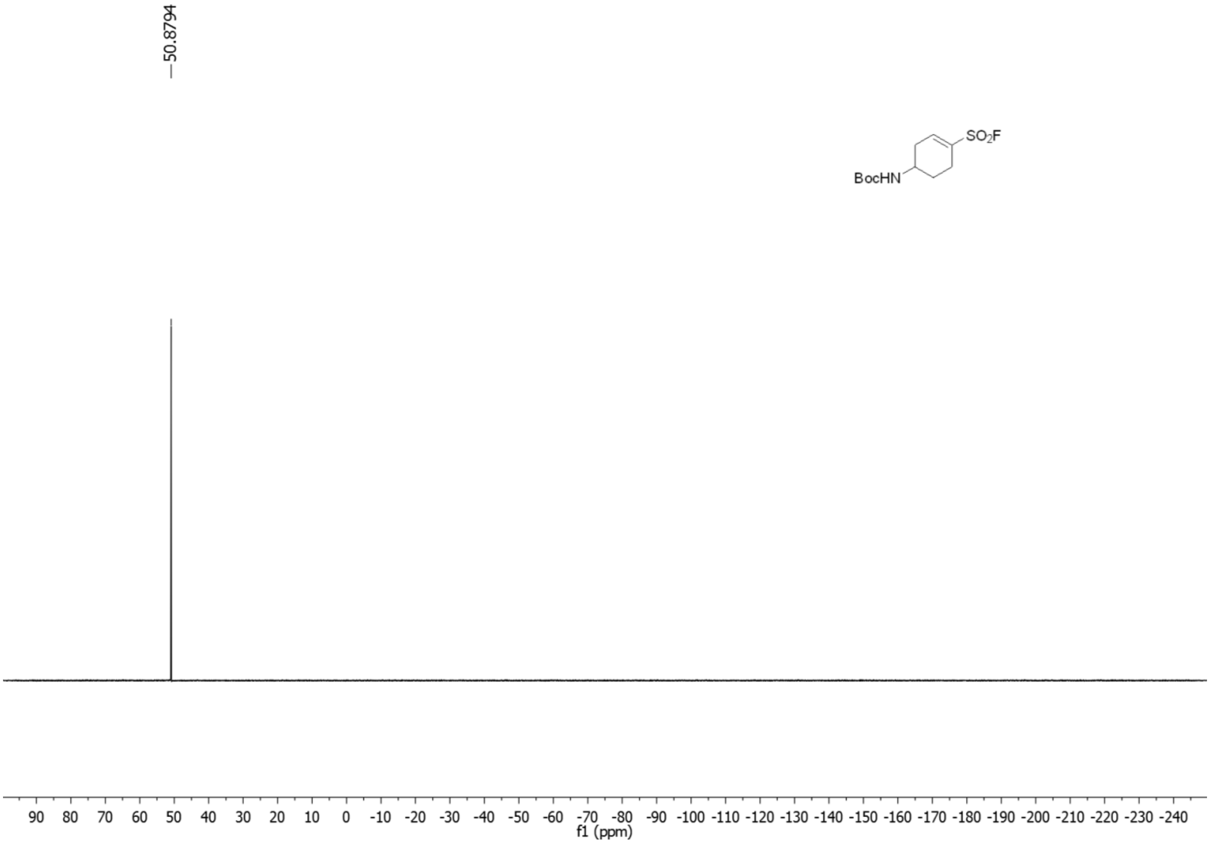

1,4-Dioxaspiro[4.5]dec-7-ene-8-sulfonyl fluoride (5h)

^{1}H-NMR Spectrum:

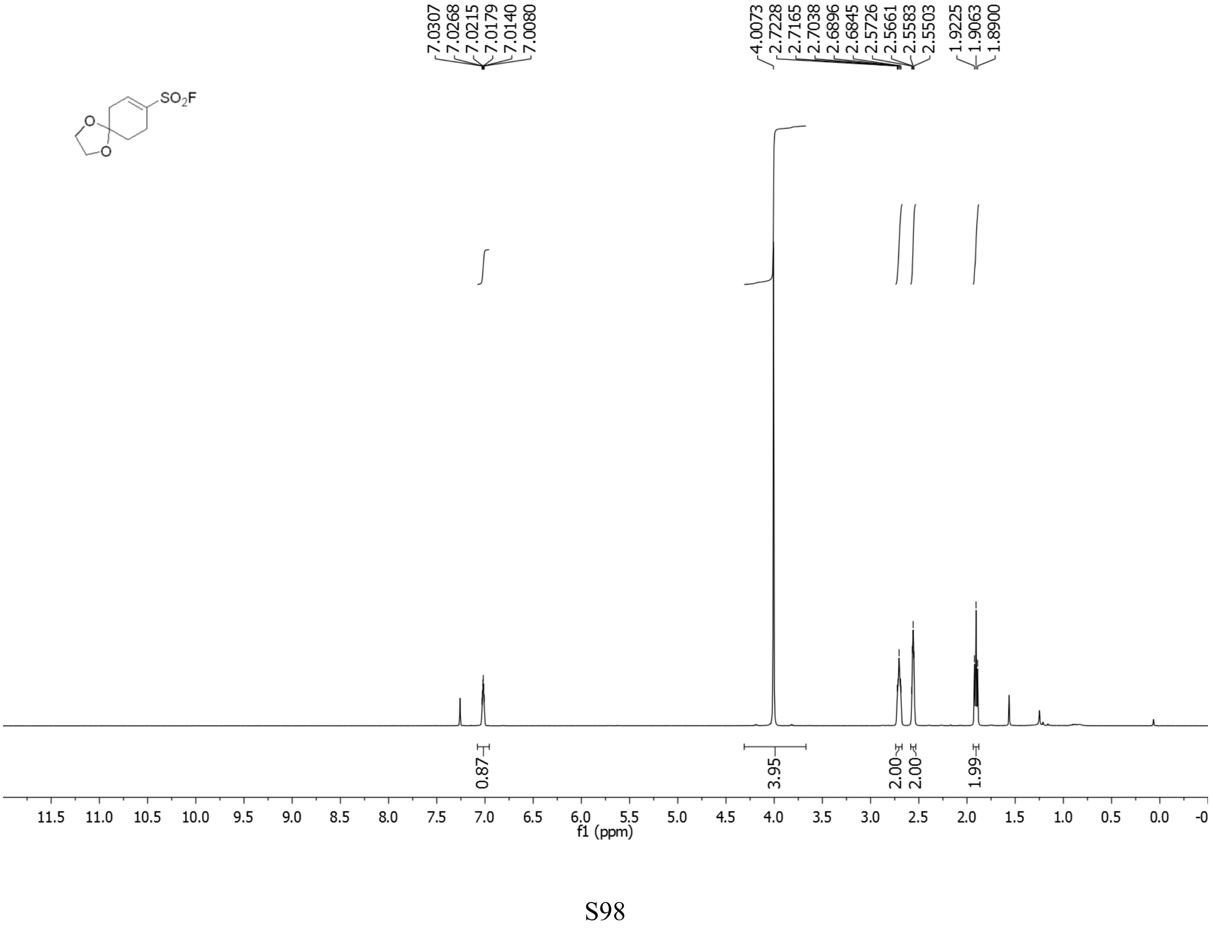

^{13}C-NMR Spectrum:

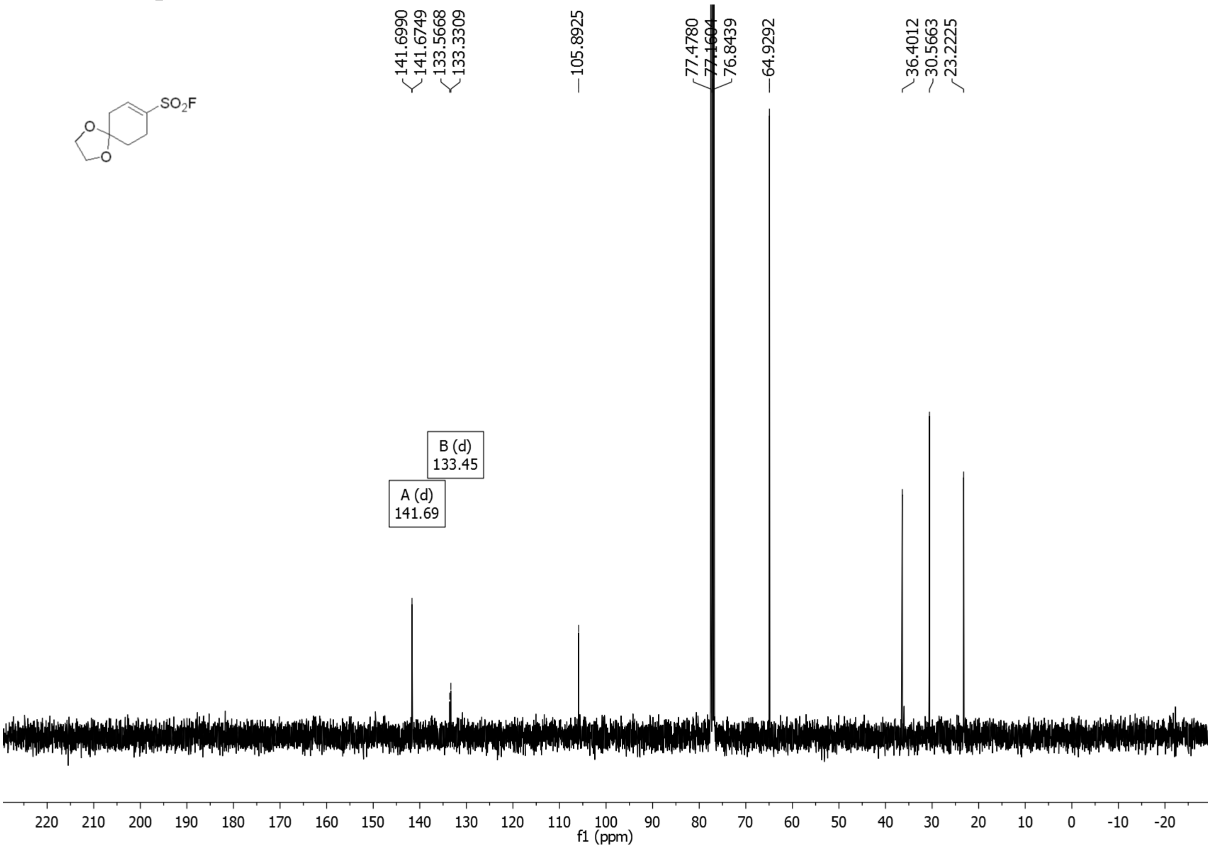

^{19}F-NMR Spectrum:

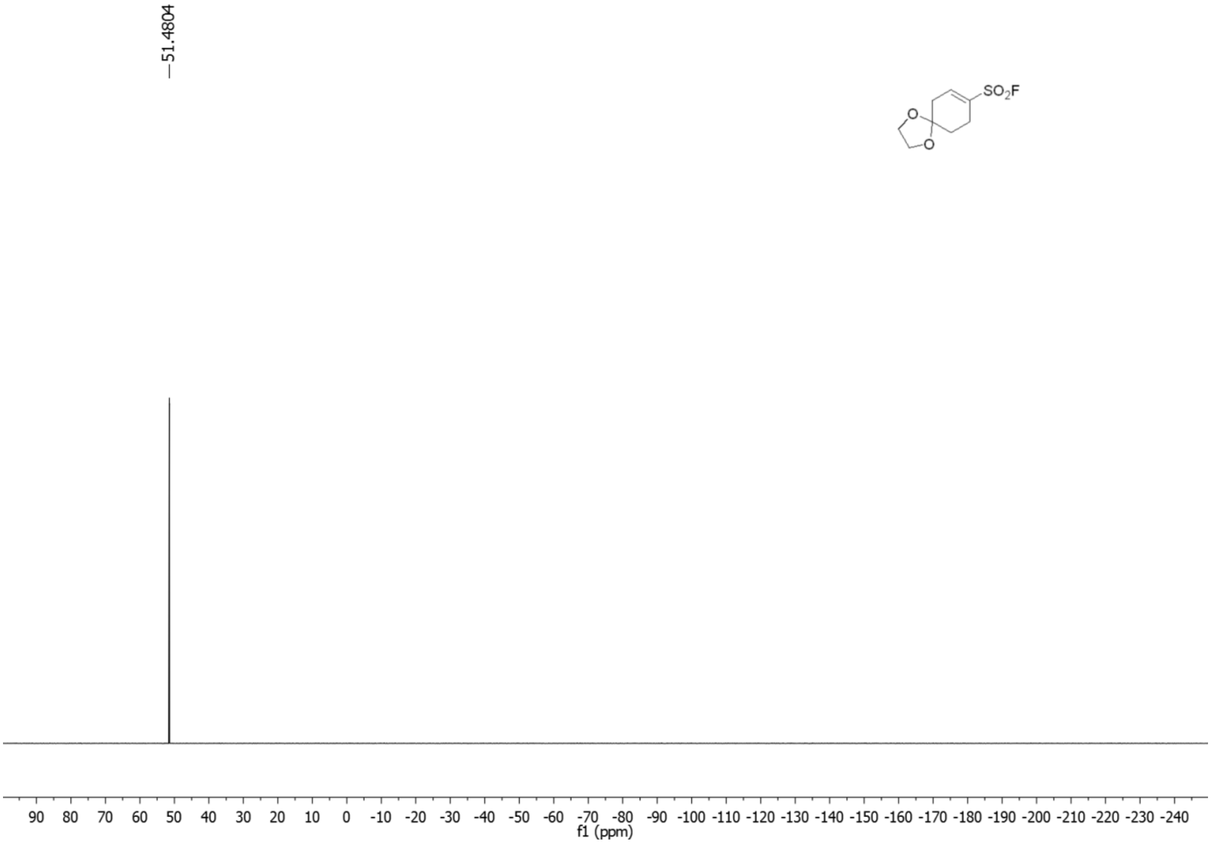

3-(1-Methyl-1*H*-indol-3-yl)cyclohex-1-ene-1-sulfonyl fluoride (5i)

^{1}H-NMR Spectrum:

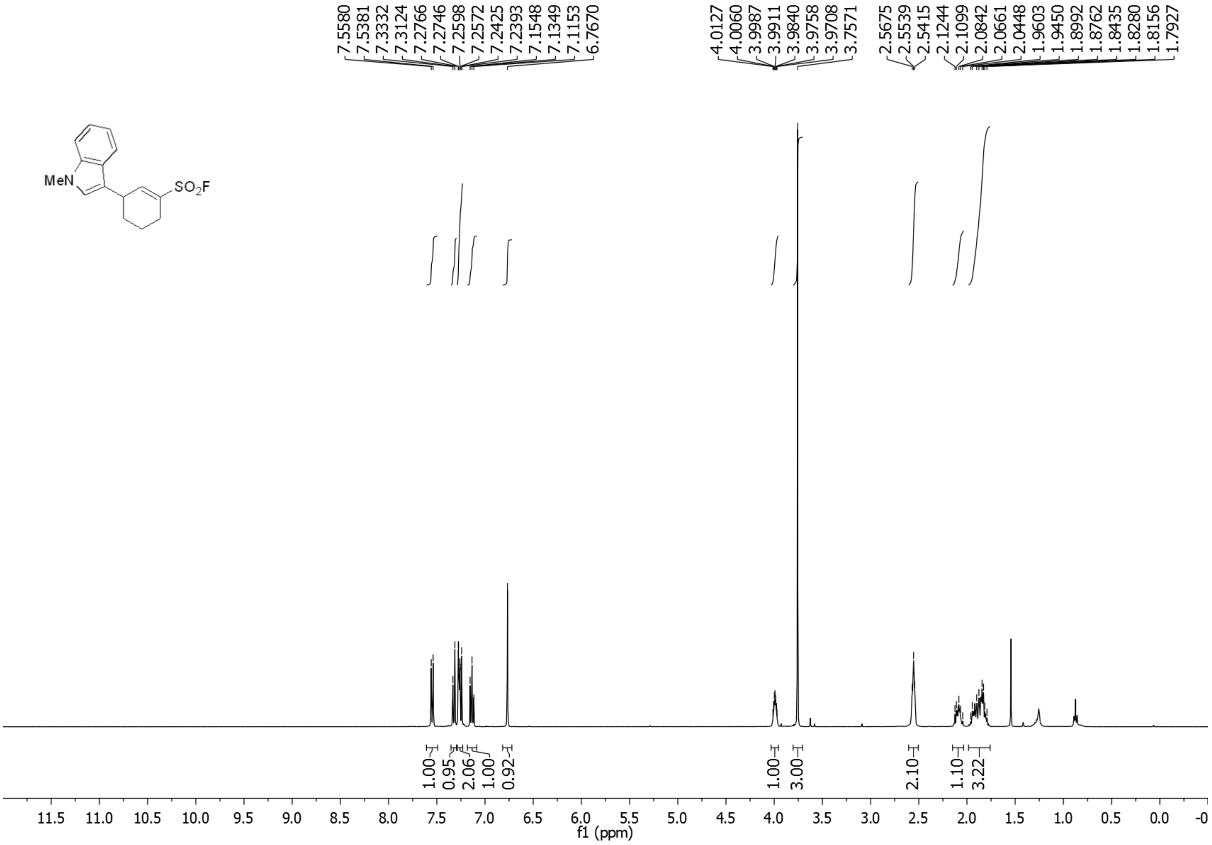

^{13}C-NMR Spectrum:

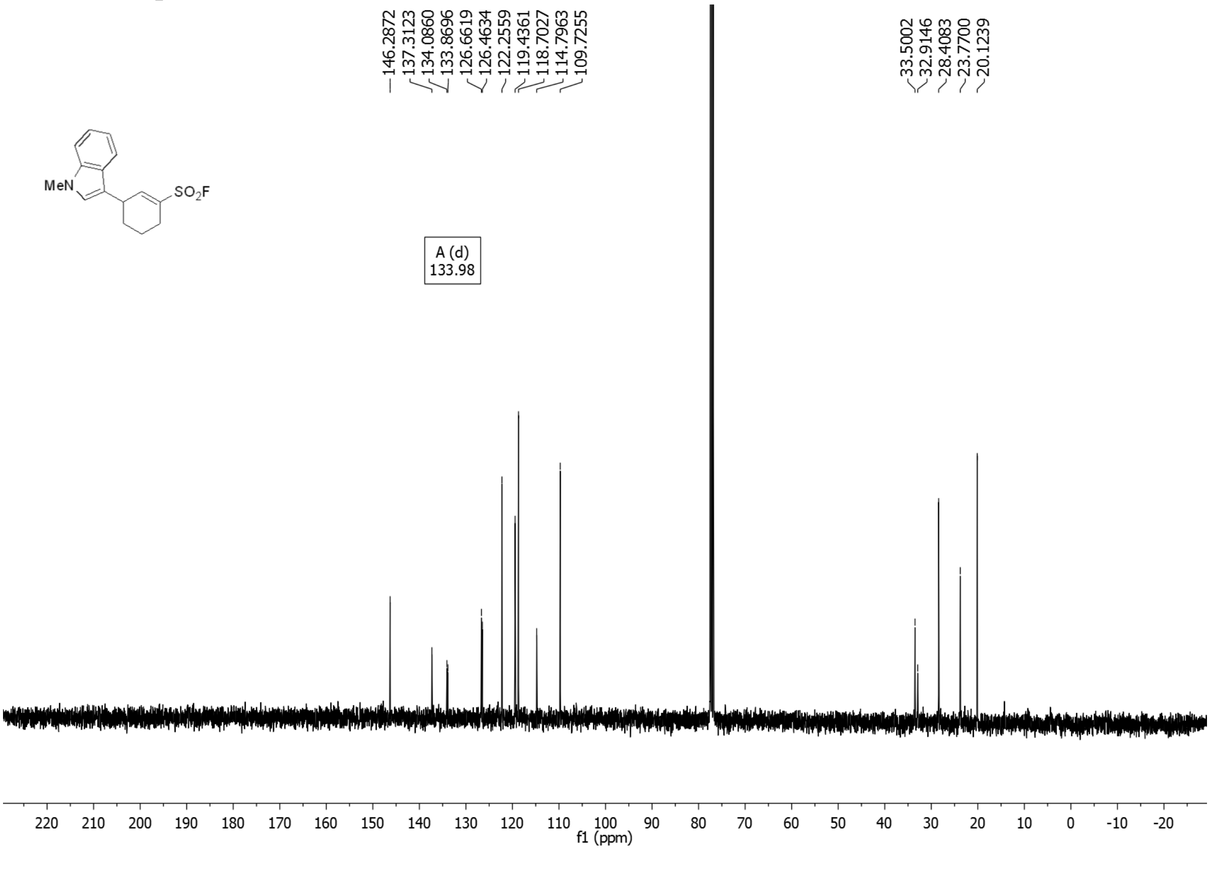

^{19}F-NMR Spectrum:

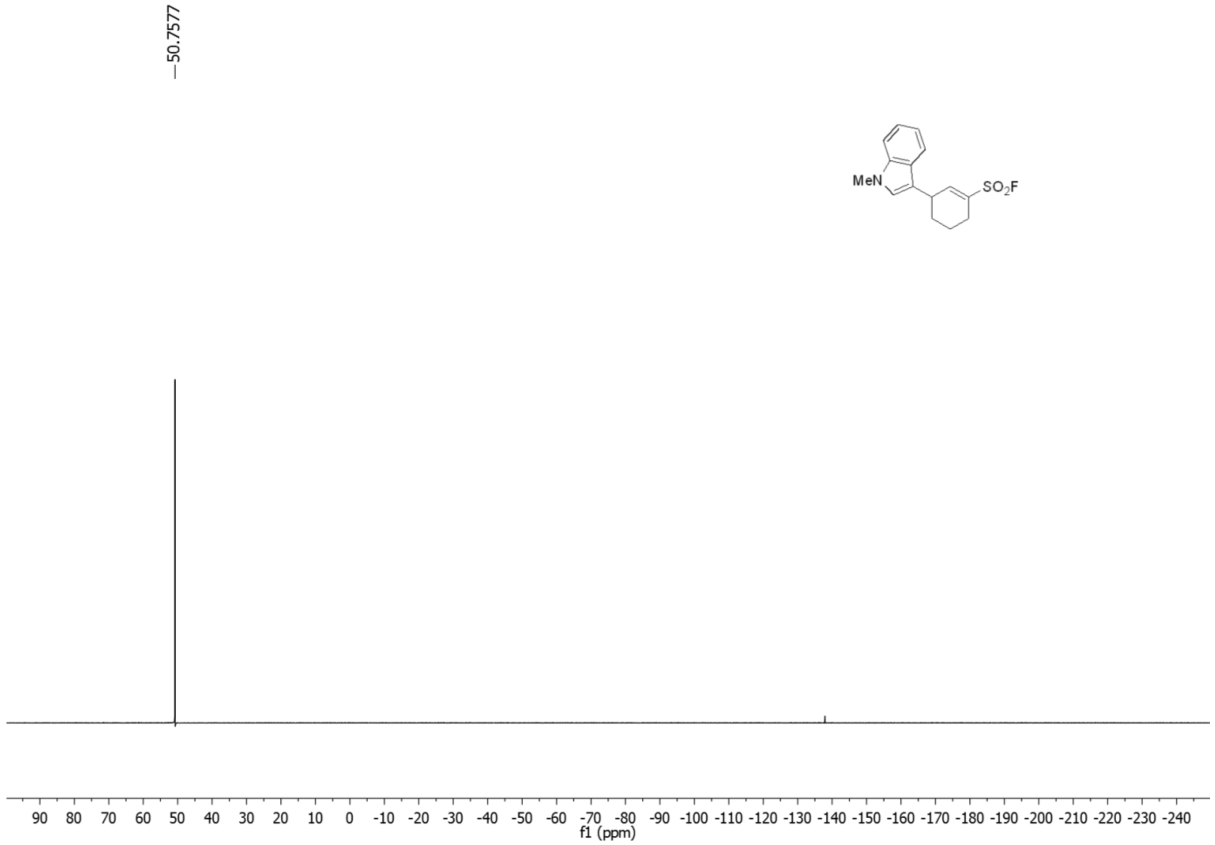

3-(5-Methylfuran-2-yl)cyclohex-1-ene-1-sulfonyl fluoride (5k)

^{1}H-NMR Spectrum:

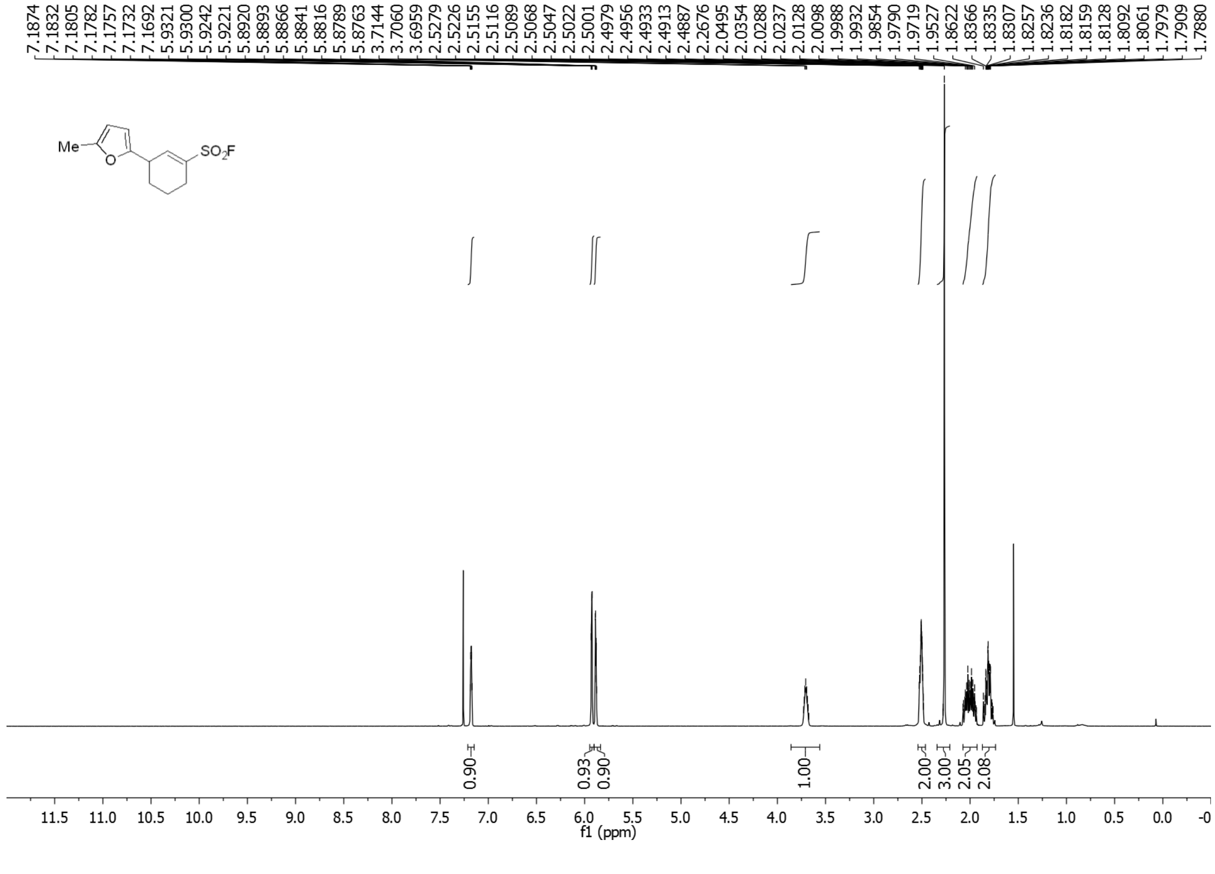

^{13}C-NMR Spectrum:

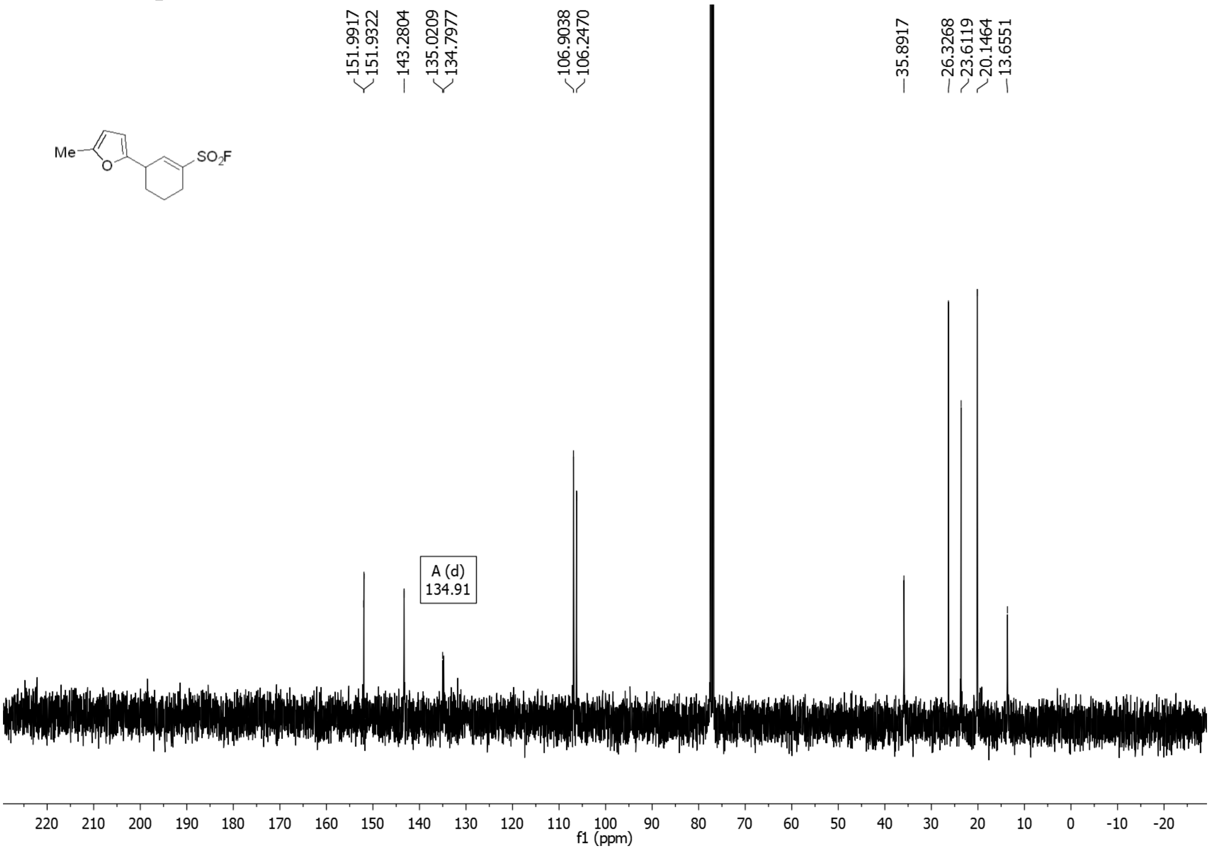

^{19}F-NMR Spectrum:

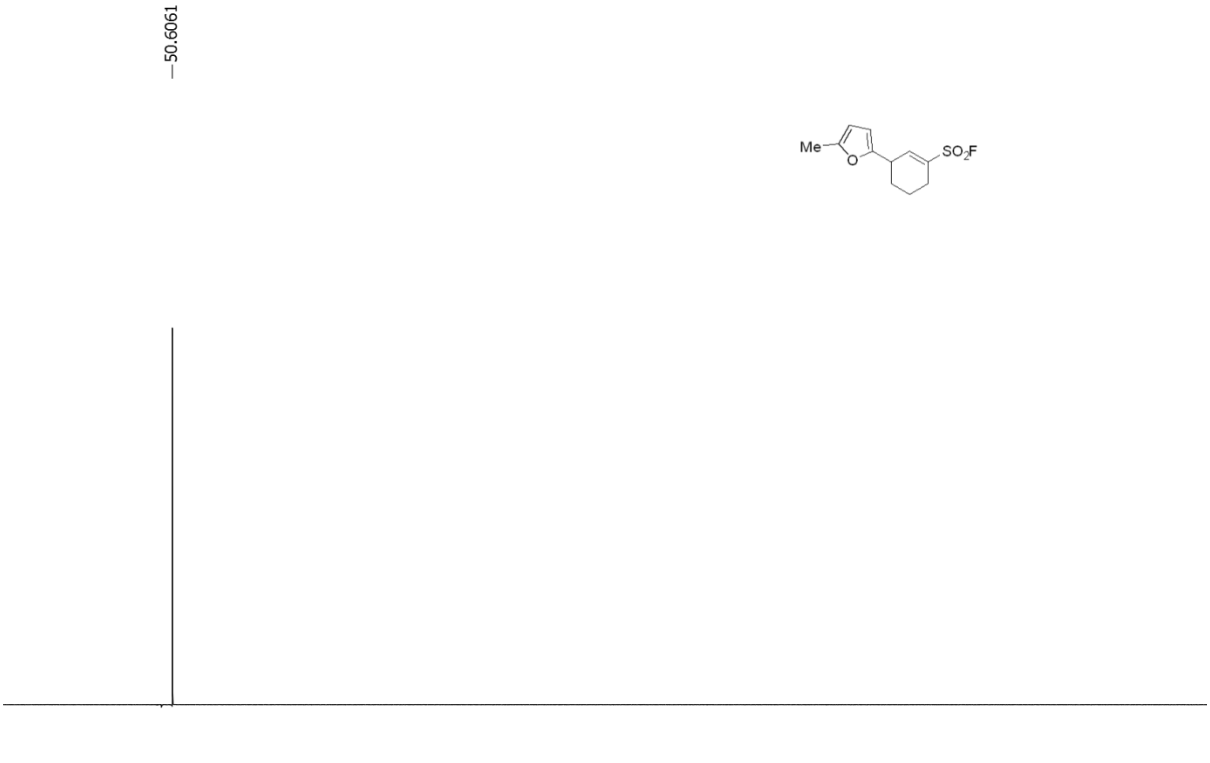

6-Methylcyclohex-1-ene-1-sulfonyl fluoride (5l)

^{1}H-NMR Spectrum:

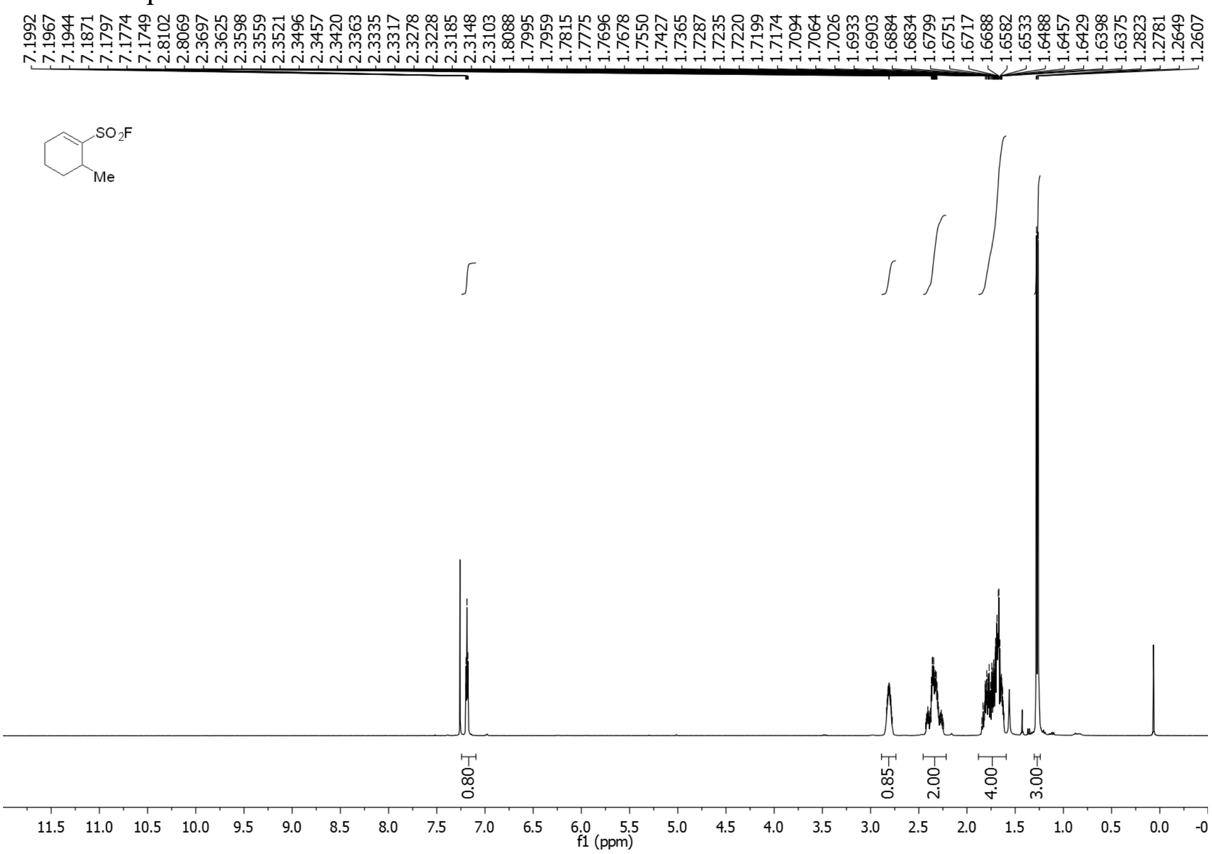

^{13}C-NMR Spectrum:

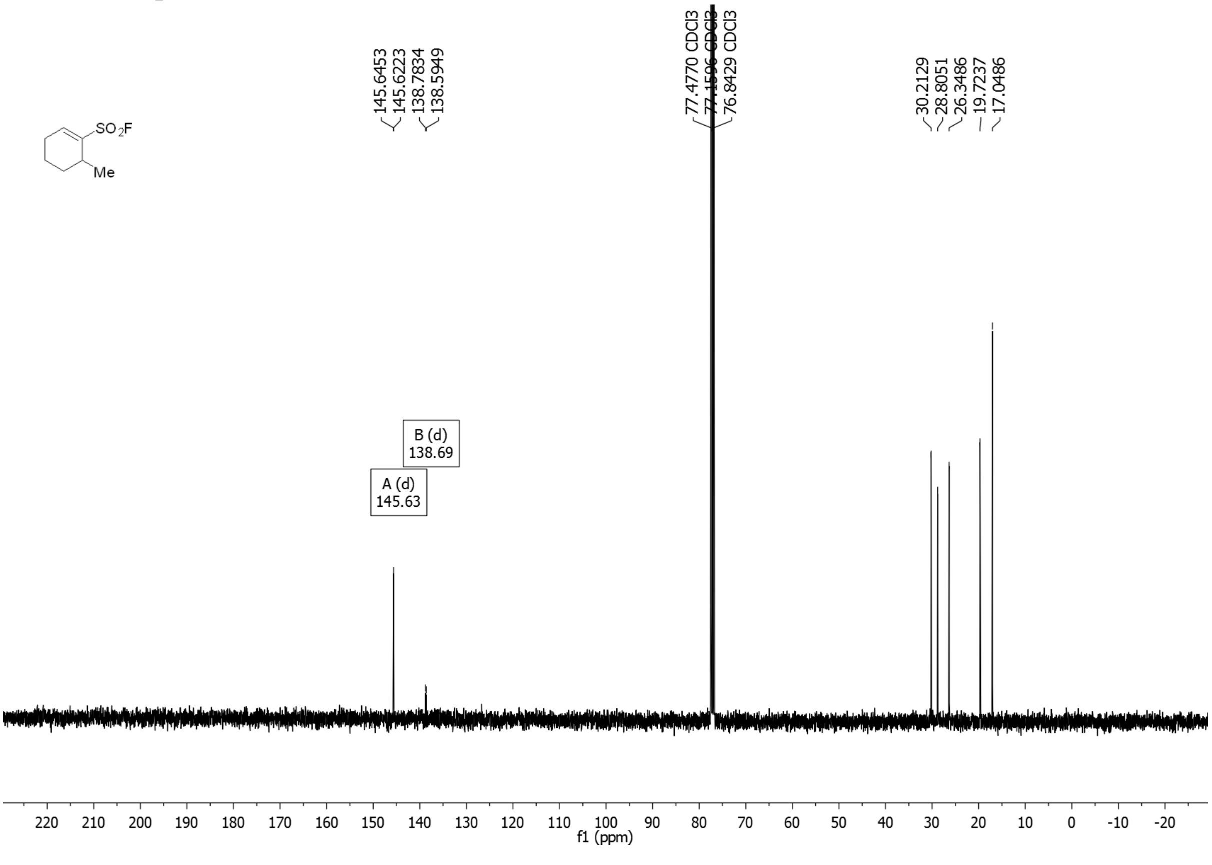

^{19}F-NMR Spectrum:

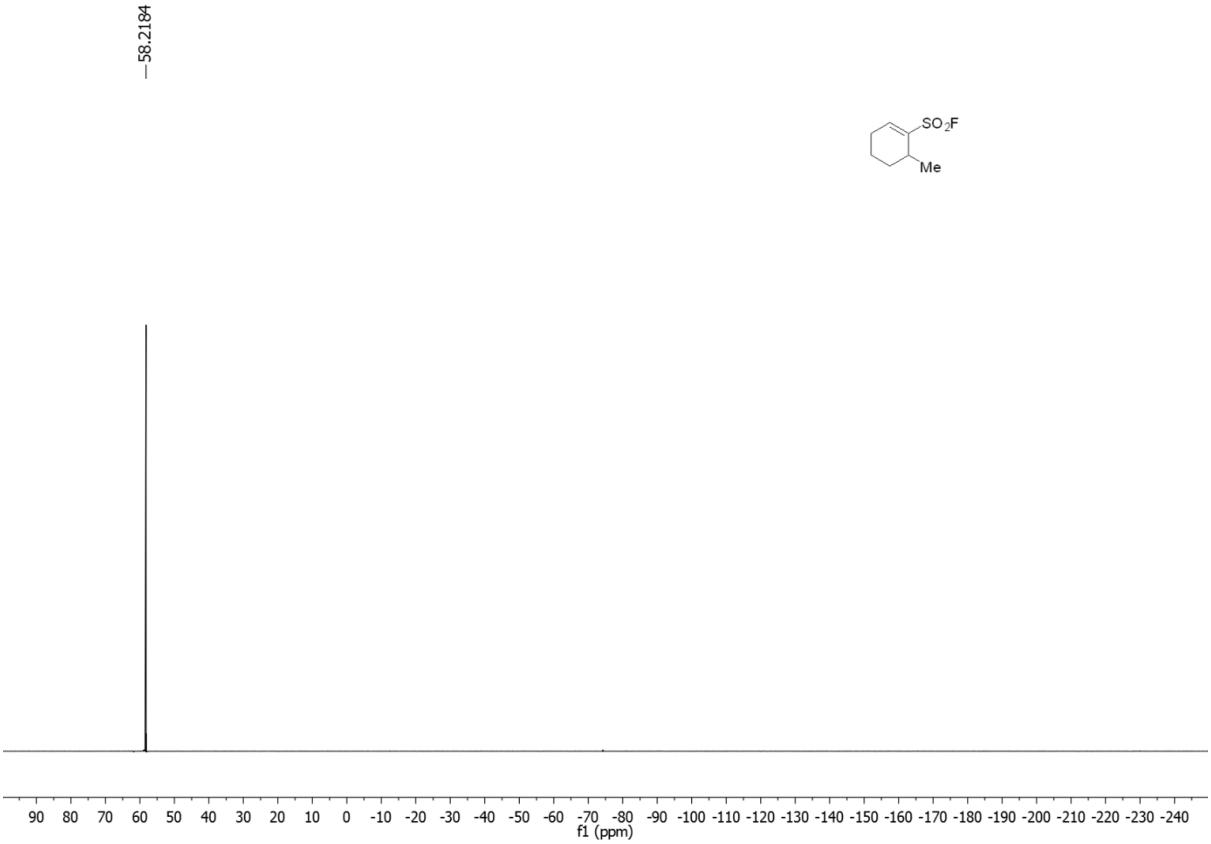

3,6-Dihydro-2*H*-thiopyran-4-sulfonyl fluoride 1,1-dioxide (5m)

^{1}H-NMR Spectrum:

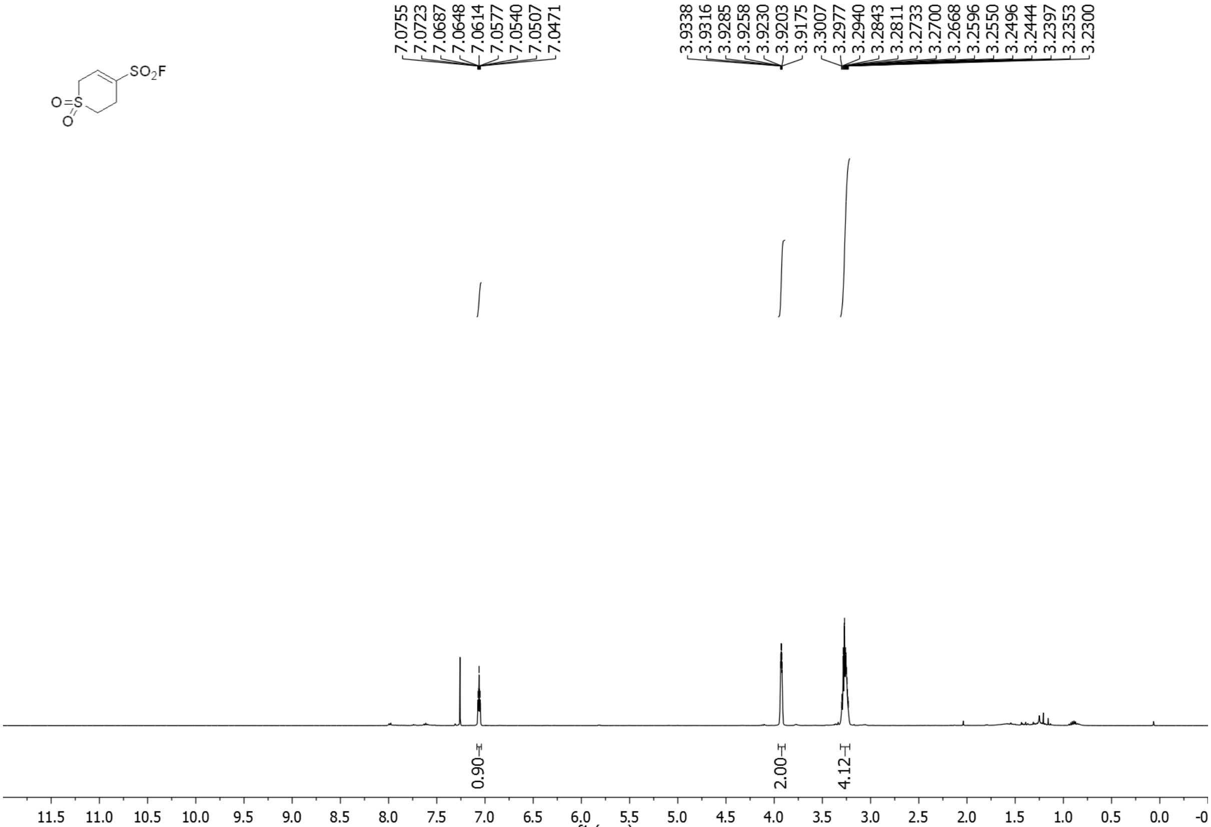

^{13}C-NMR Spectrum:

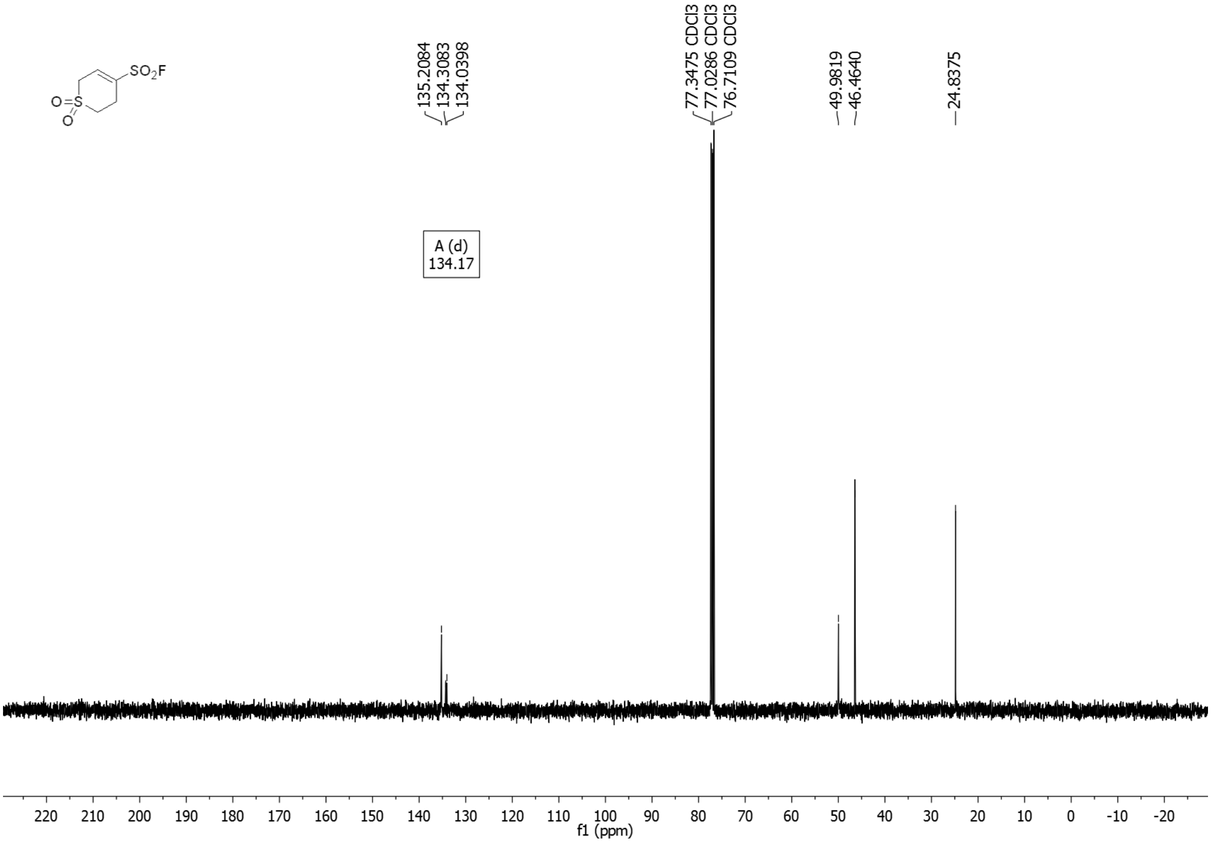

^{19}F-NMR Spectrum:

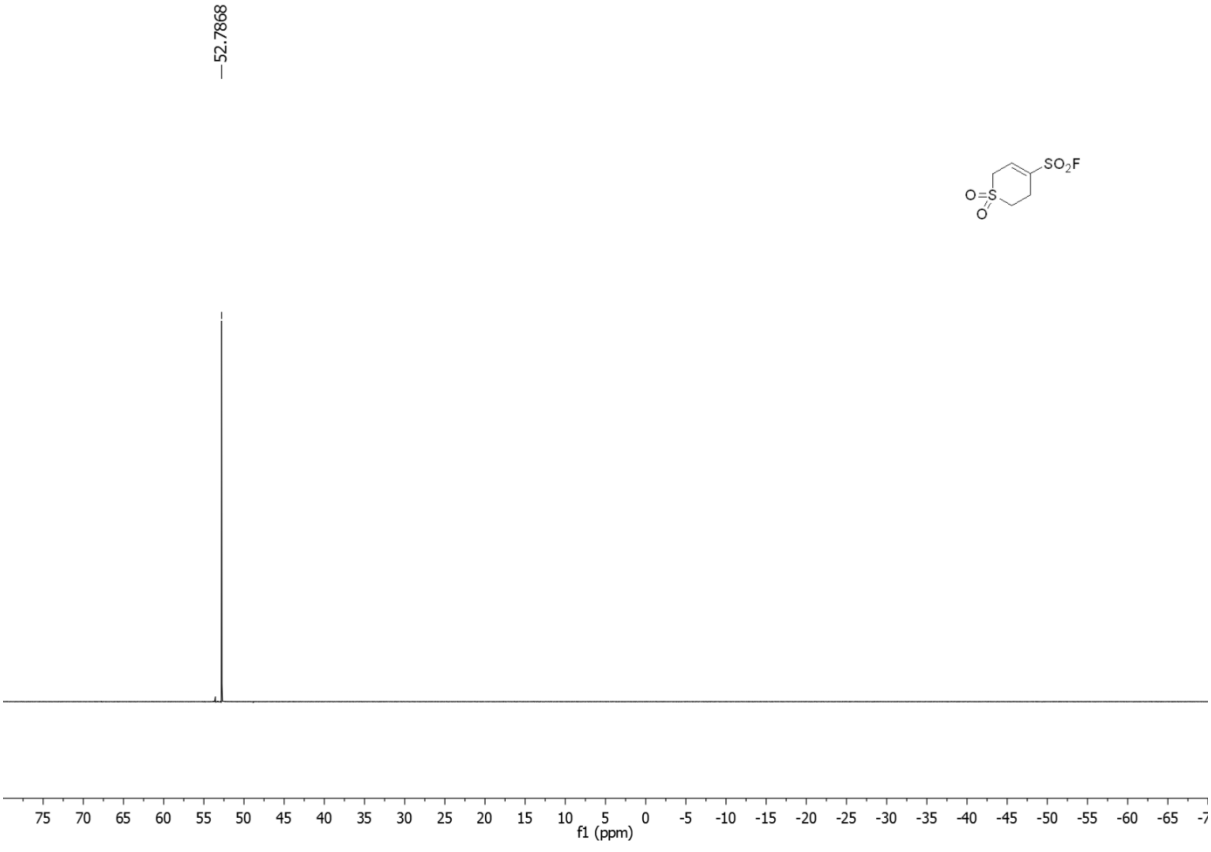

3,6-Dihydro-2*H*-pyran-4-sulfonyl fluoride (5n)

^{1}H-NMR Spectrum:

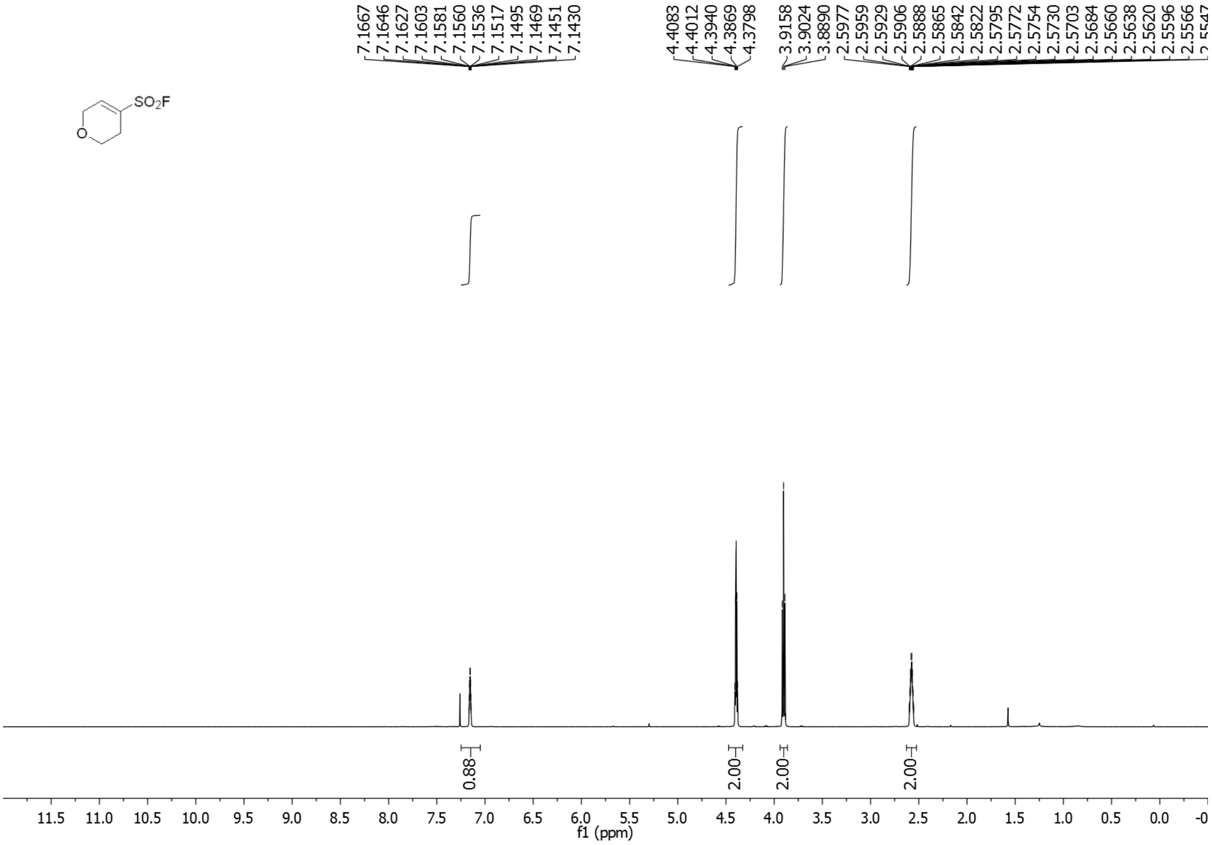

^{13}C-NMR Spectrum:

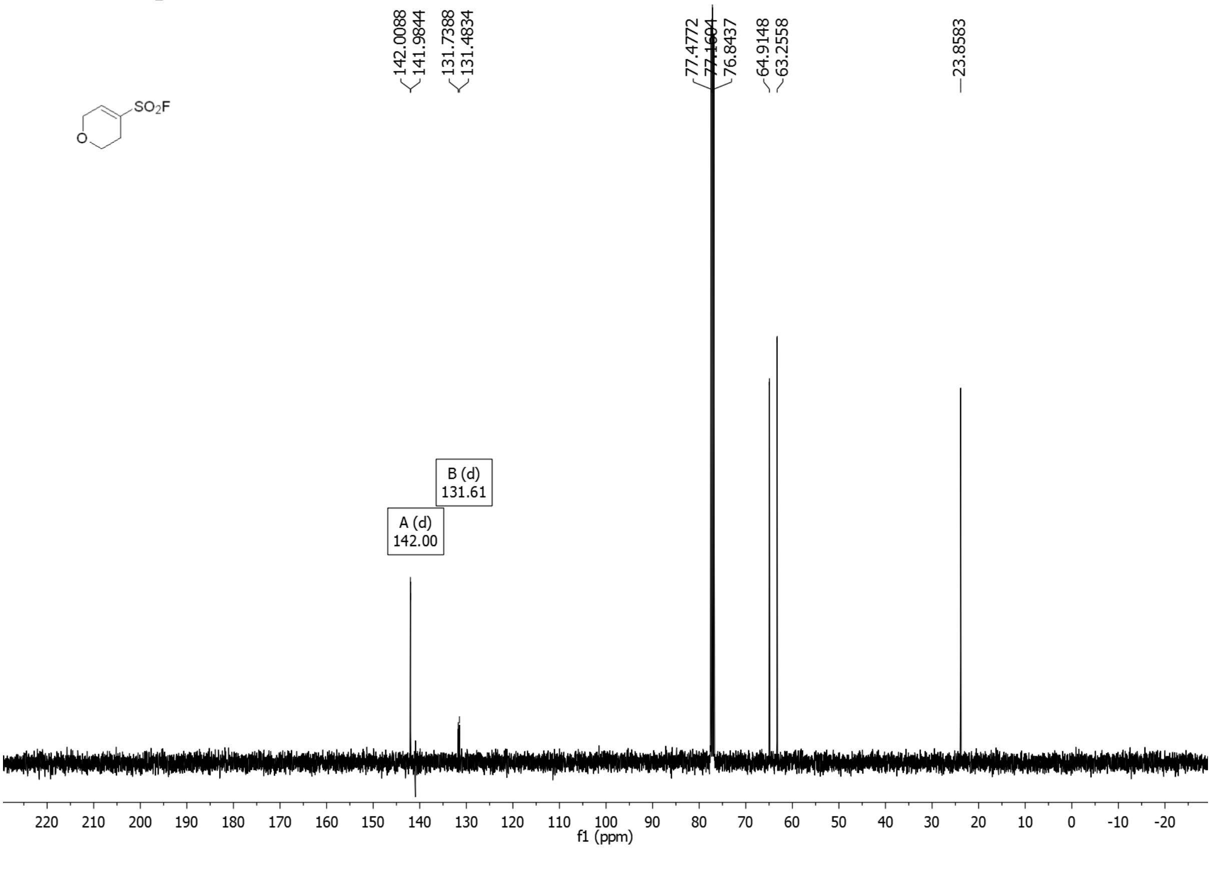

^{19}F-NMR Spectrum:

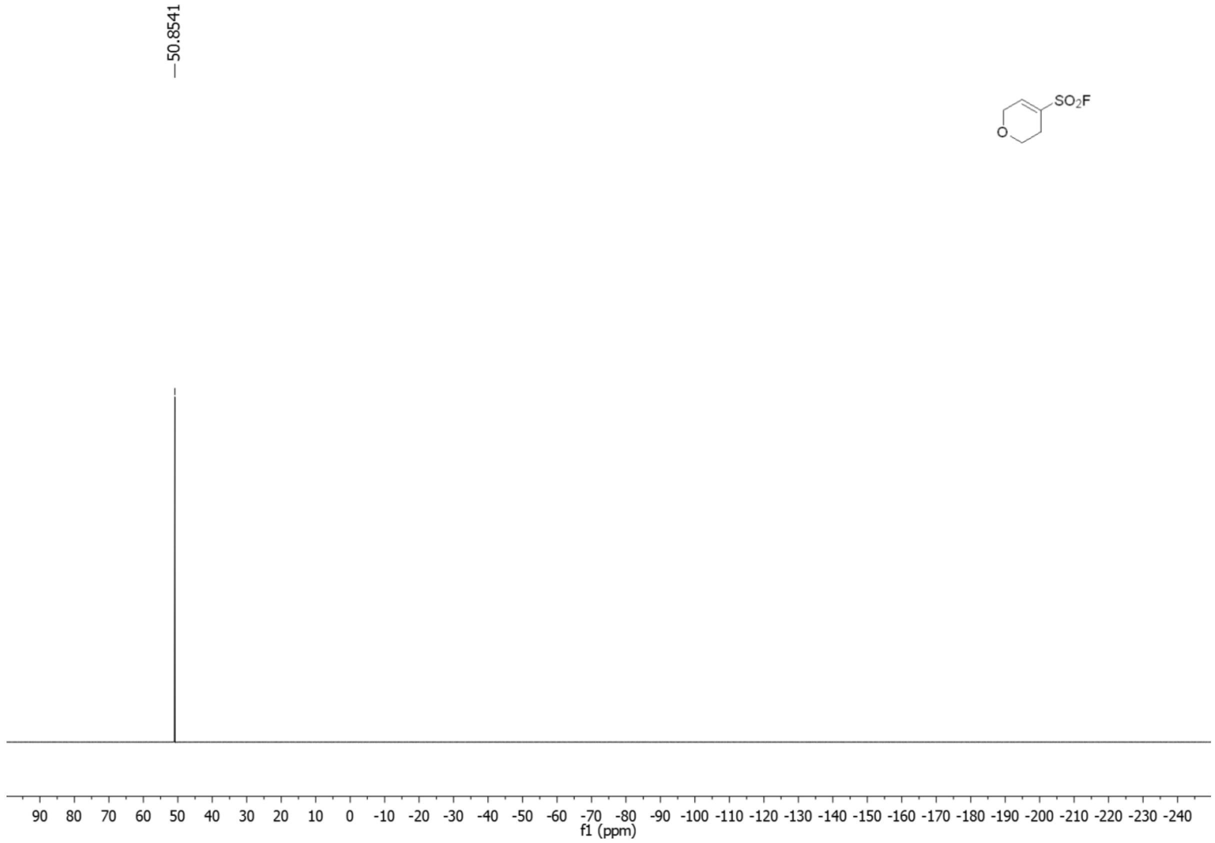

***tert*-Butyl 5-(fluorosulfonyl)-3,6-dihydropyridine-1(2*H*)-carboxylate (5o)**

^{1}H-NMR Spectrum:

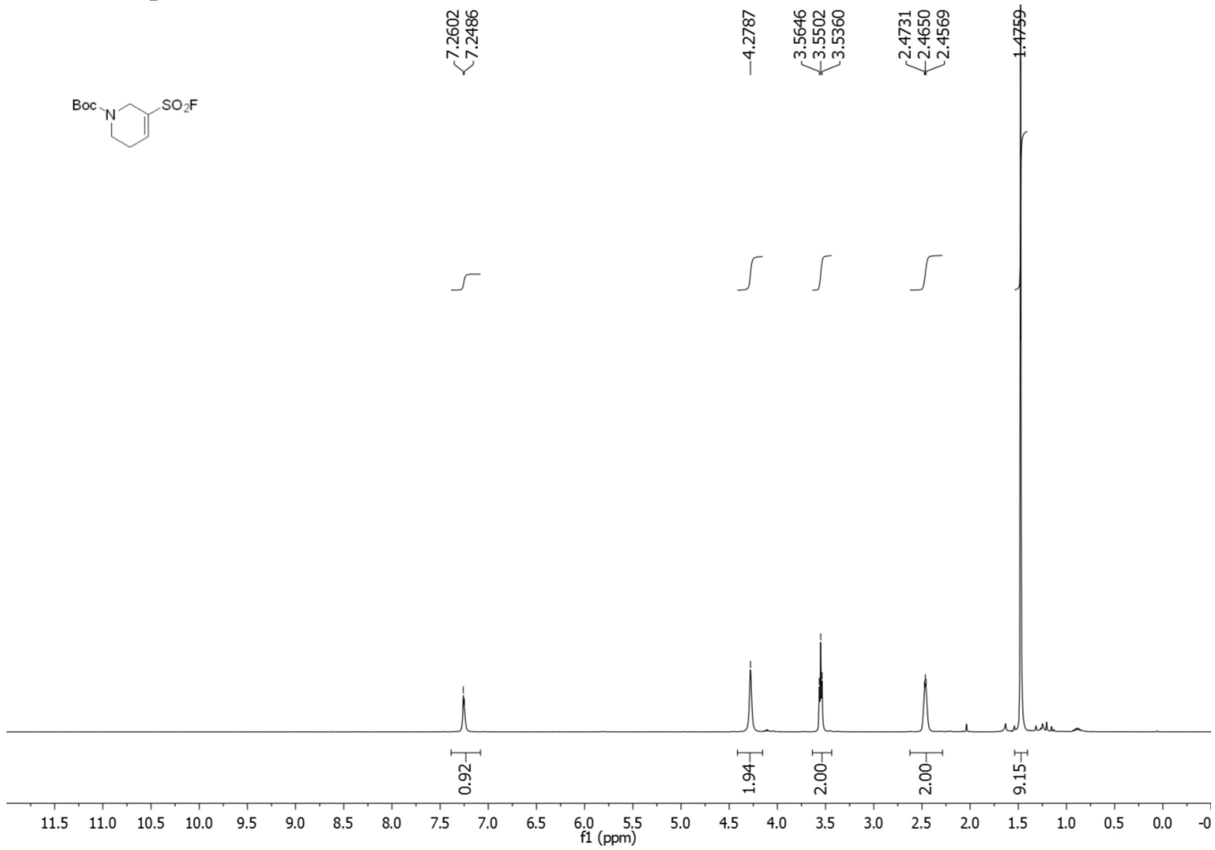

^{13}C-NMR Spectrum:

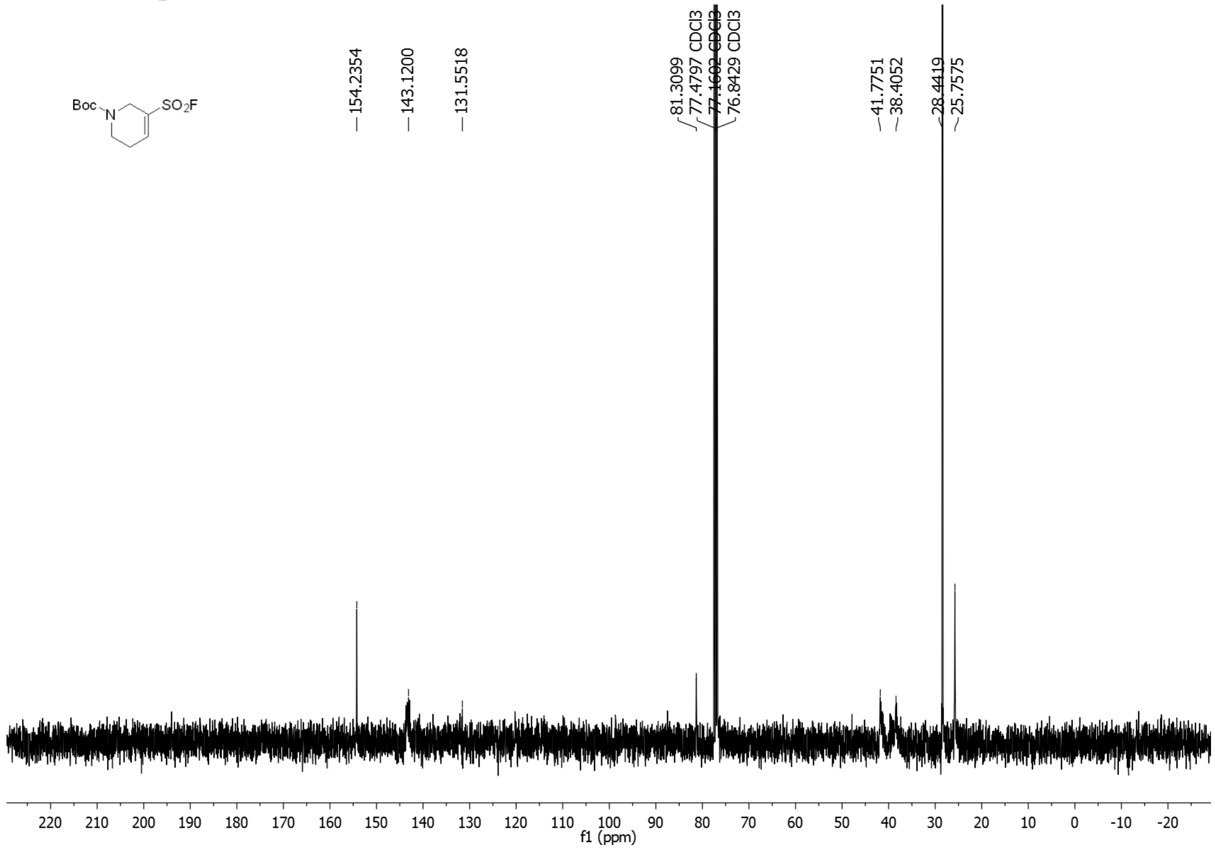

^{19}F-NMR Spectrum:

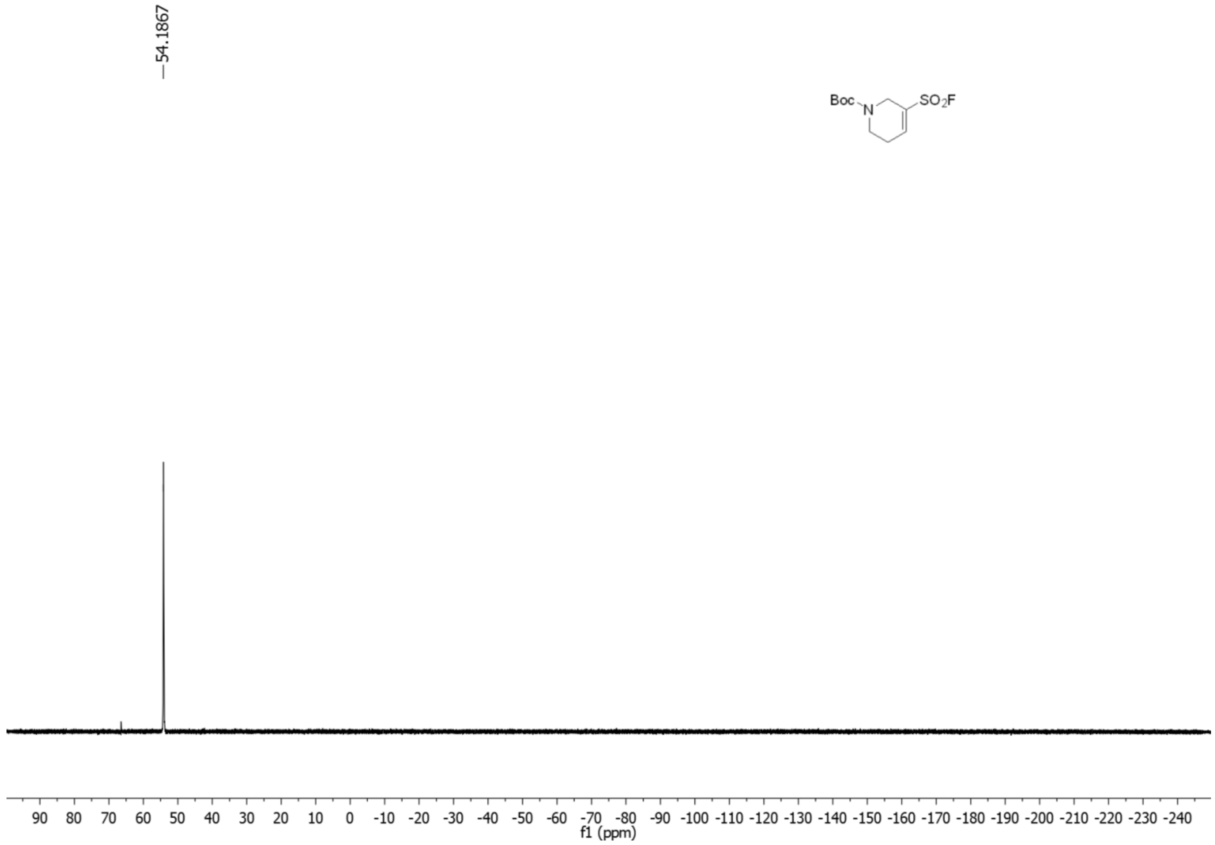

tert-Butyl 4-(fluorosulfonyl)-3,6-dihydropyridine-1(2*H*)-carboxylate (5p)

^{1}H-NMR Spectrum:

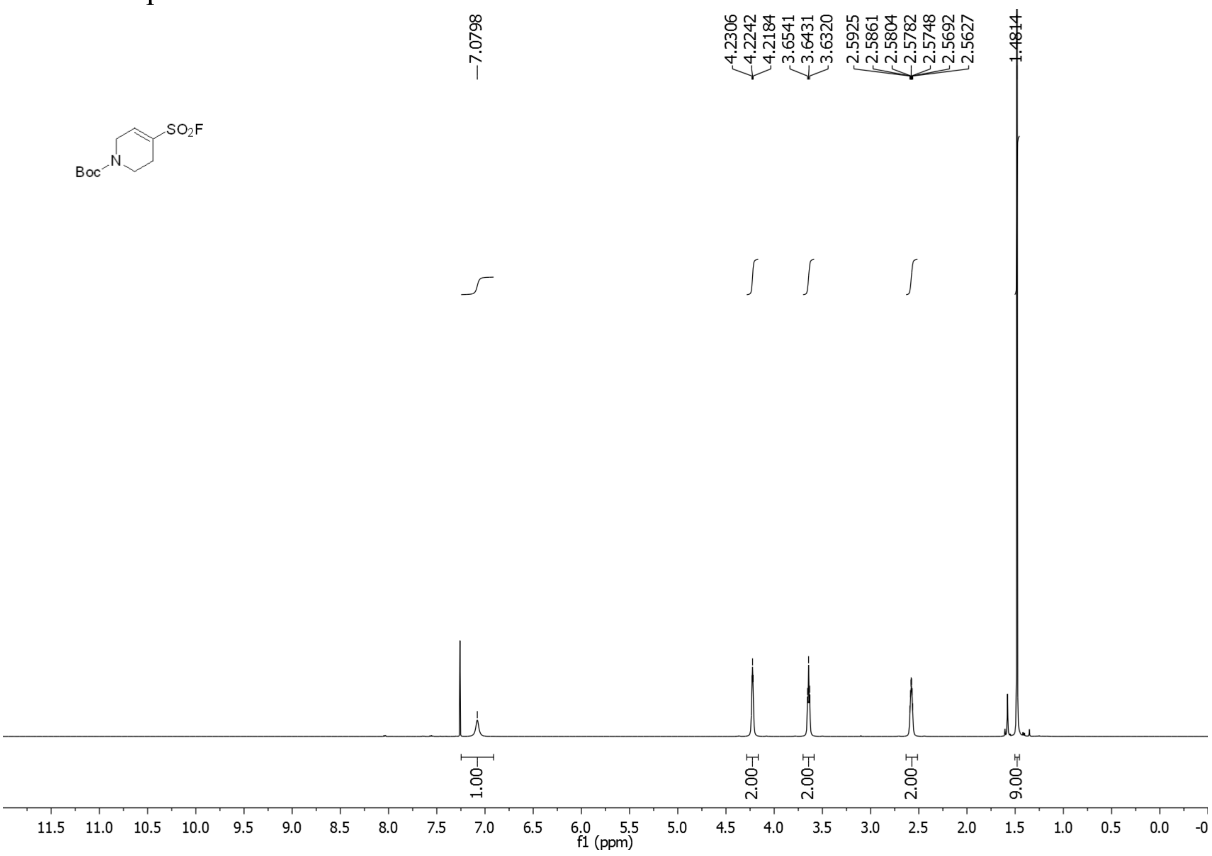

^{13}C-NMR Spectrum:

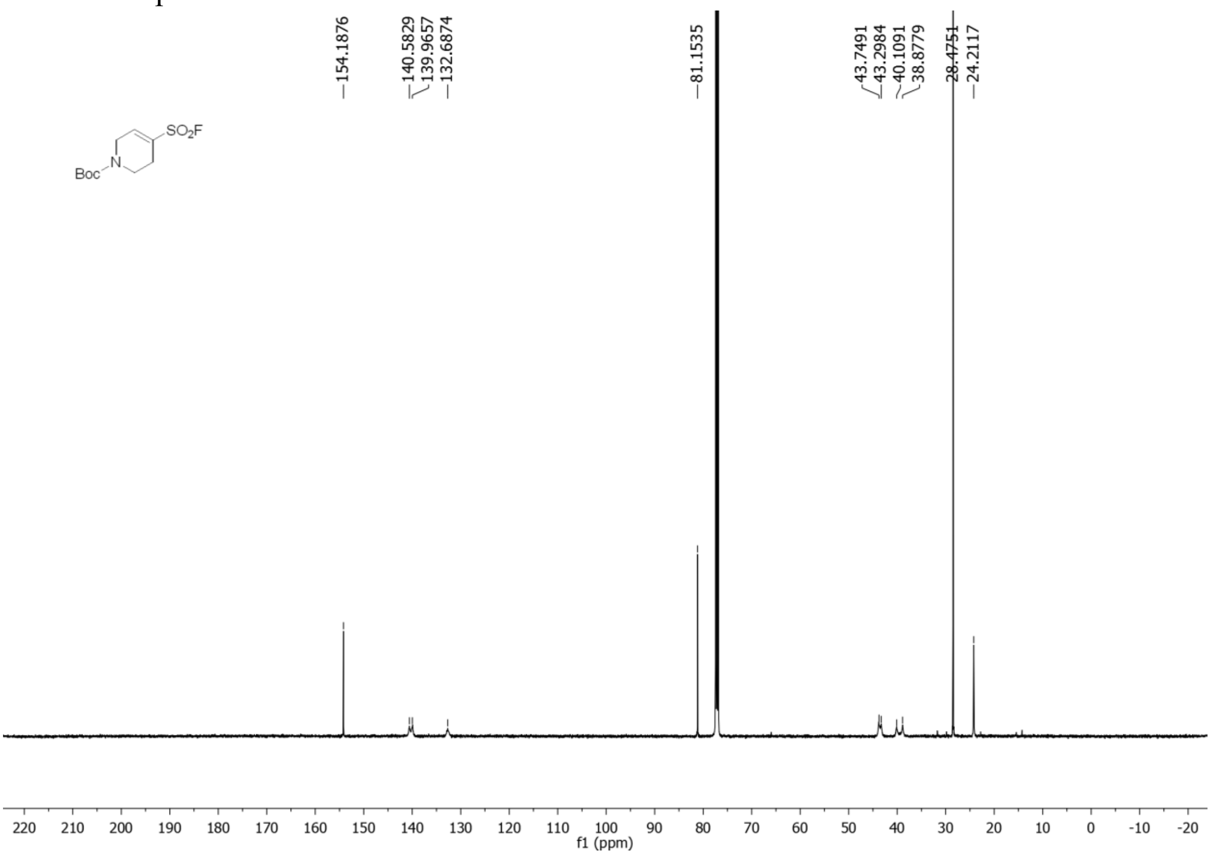

^{19}F-NMR Spectrum:

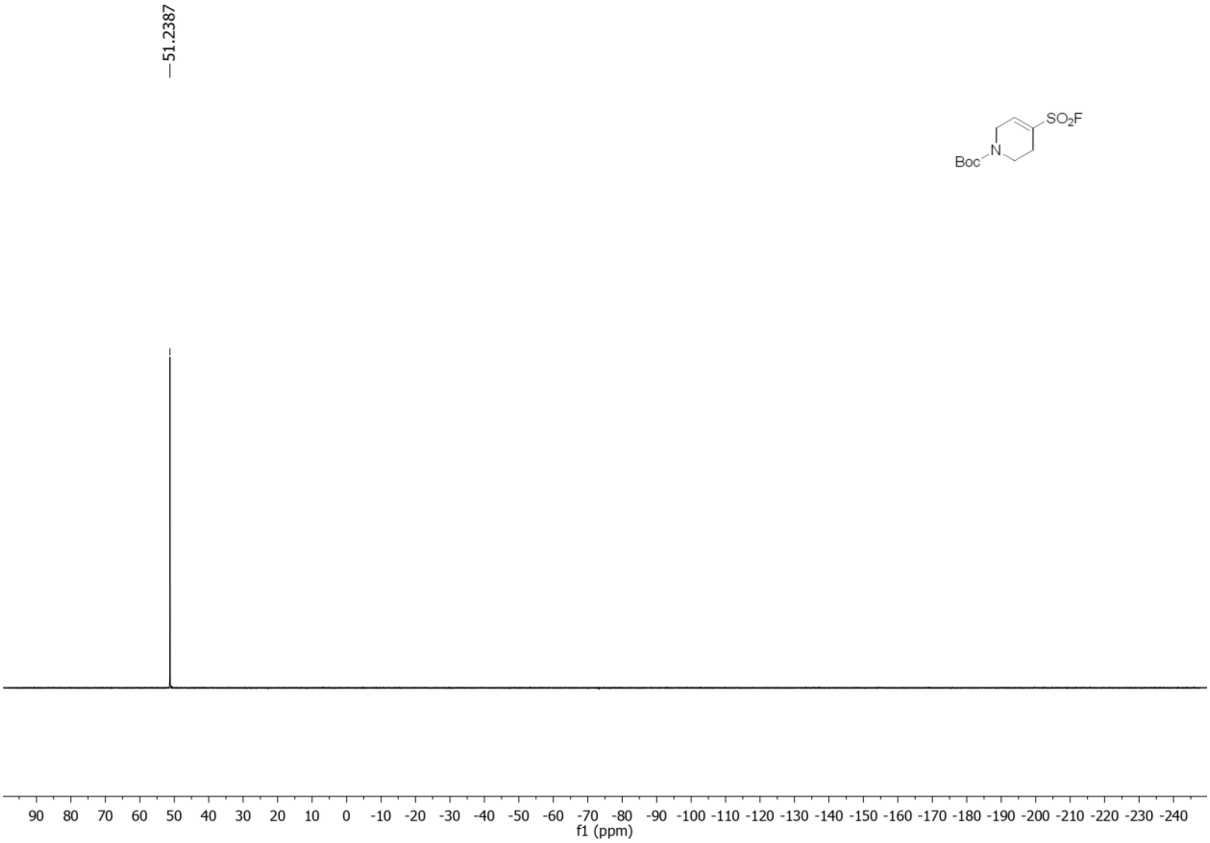

Benzyl 4-(fluorosulfonyl)-3,6-dihydropyridine-1(2*H*)-carboxylate (5q)

^{1}H-NMR Spectrum:

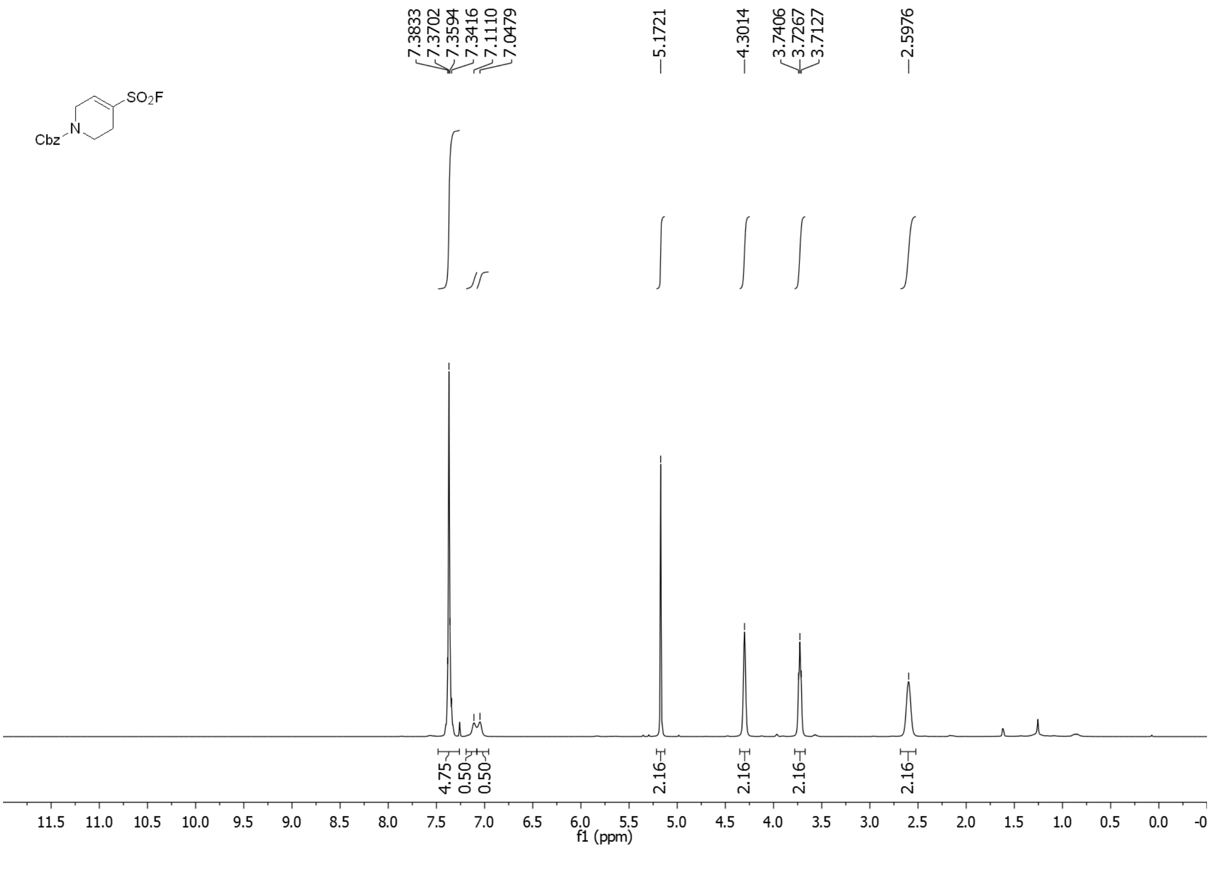

^{13}C-NMR Spectrum:

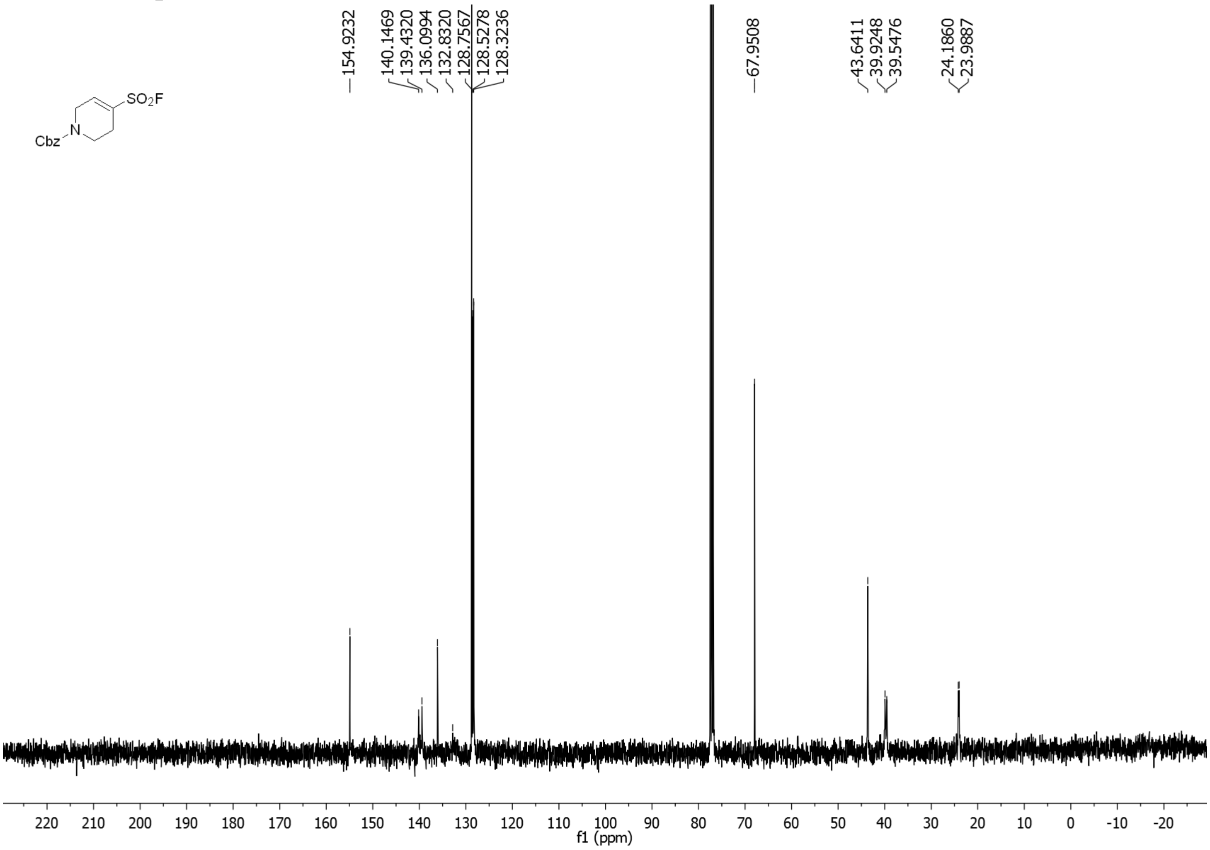

^{19}F-NMR Spectrum:

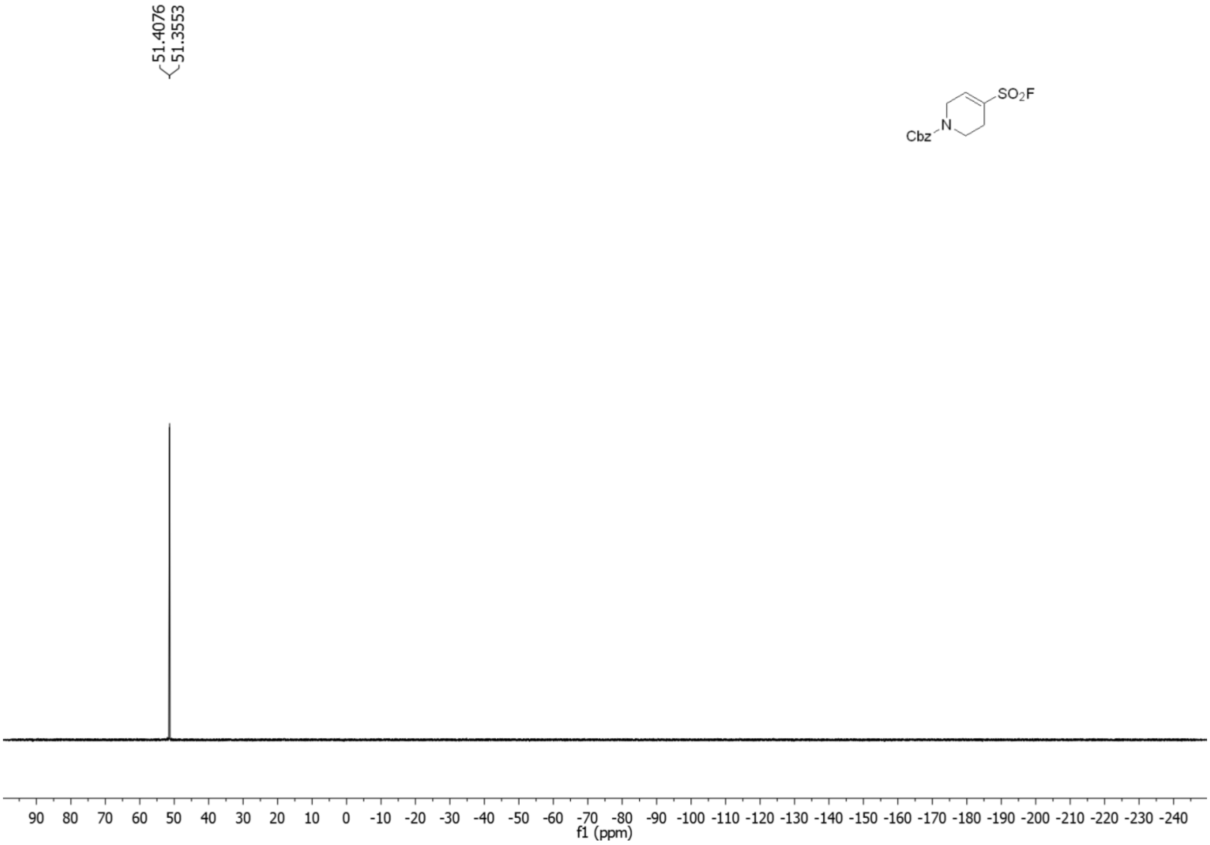

1-Benzyl-1,2,3,6-tetrahydropyridine-4-sulfonyl fluoride (5r)

^{1}H-NMR Spectrum:

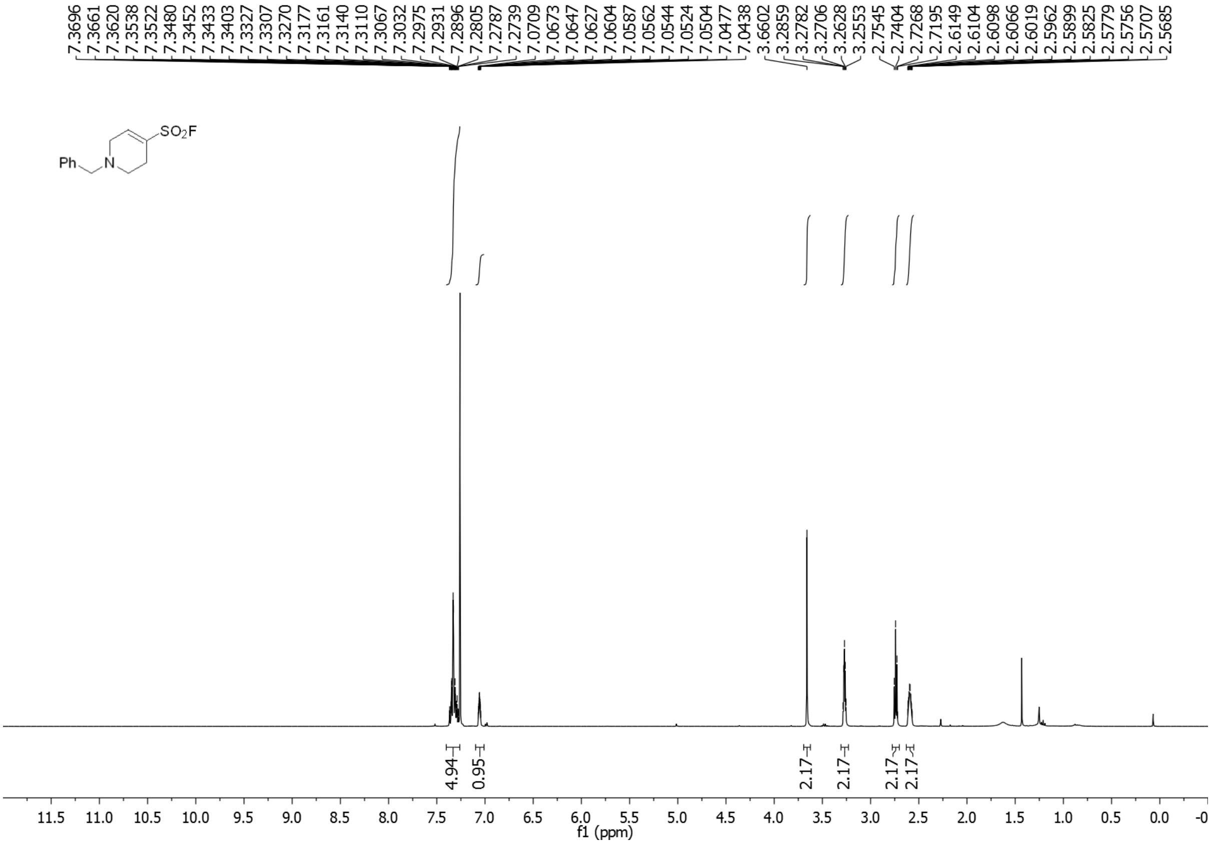

^{13}C-NMR Spectrum:

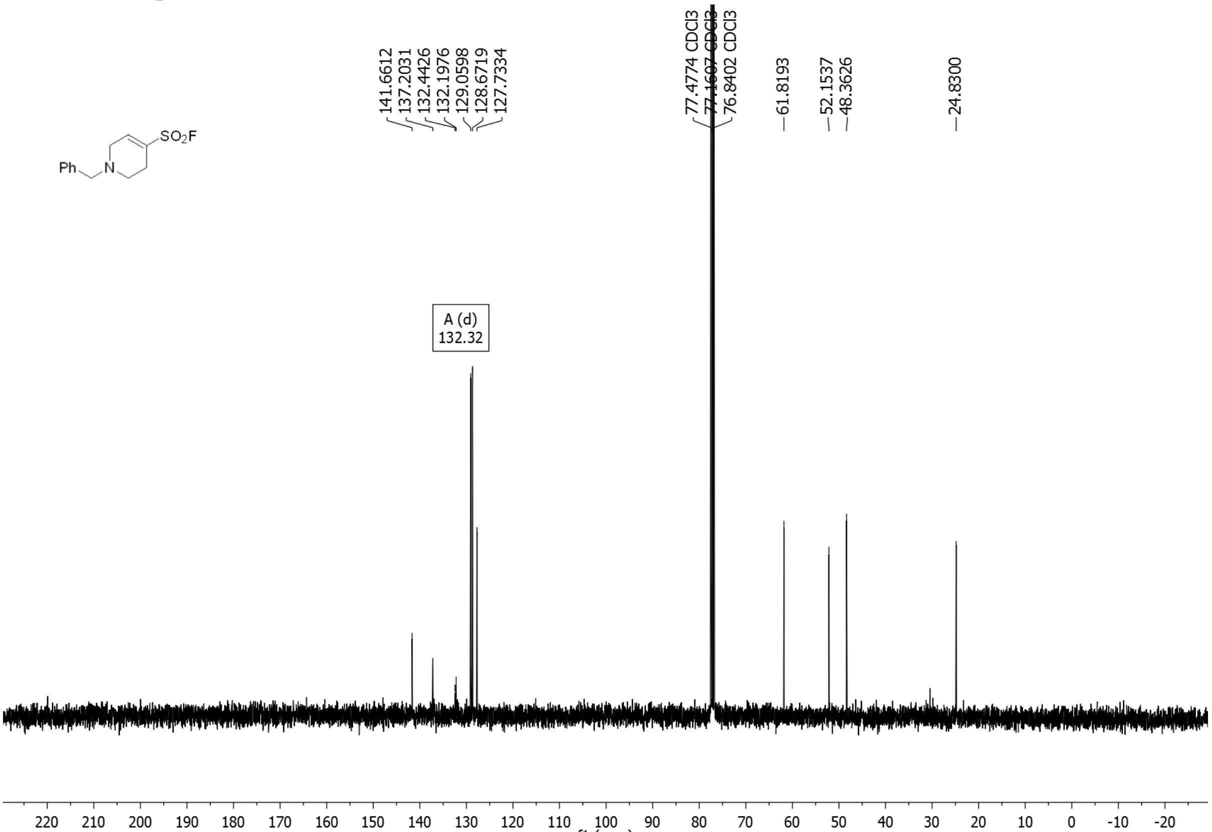

^{19}F-NMR Spectrum:

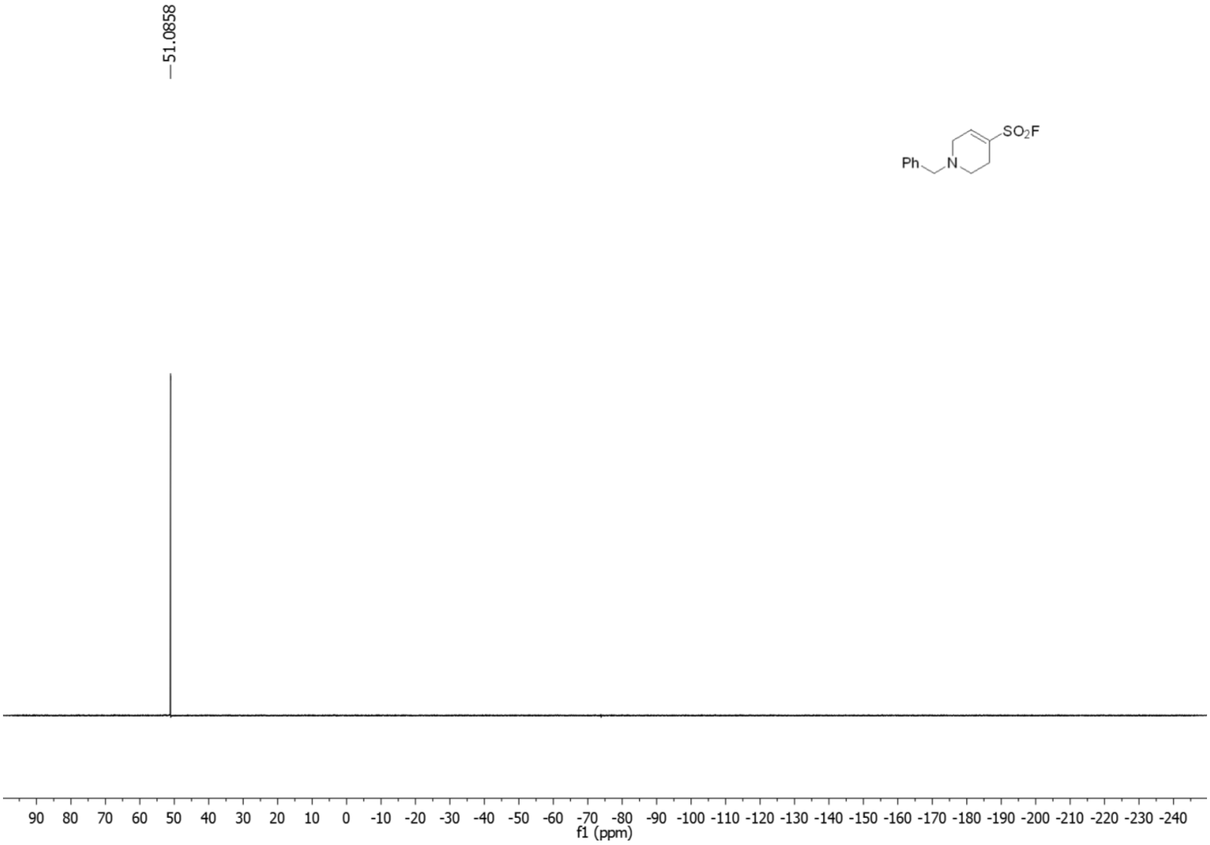

1-Tosyl-1,2,3,6-tetrahydropyridine-4-sulfonyl fluoride (5s)

^{1}H-NMR Spectrum:

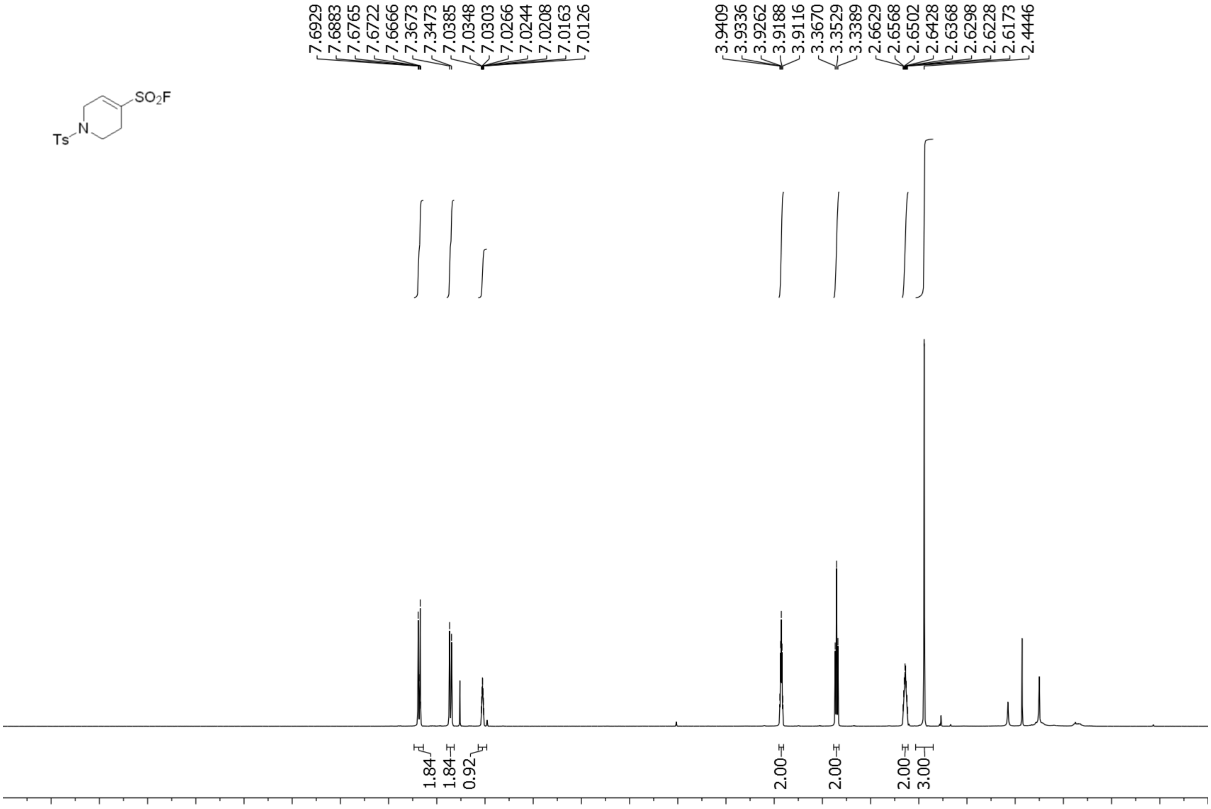

^{13}C-NMR Spectrum:

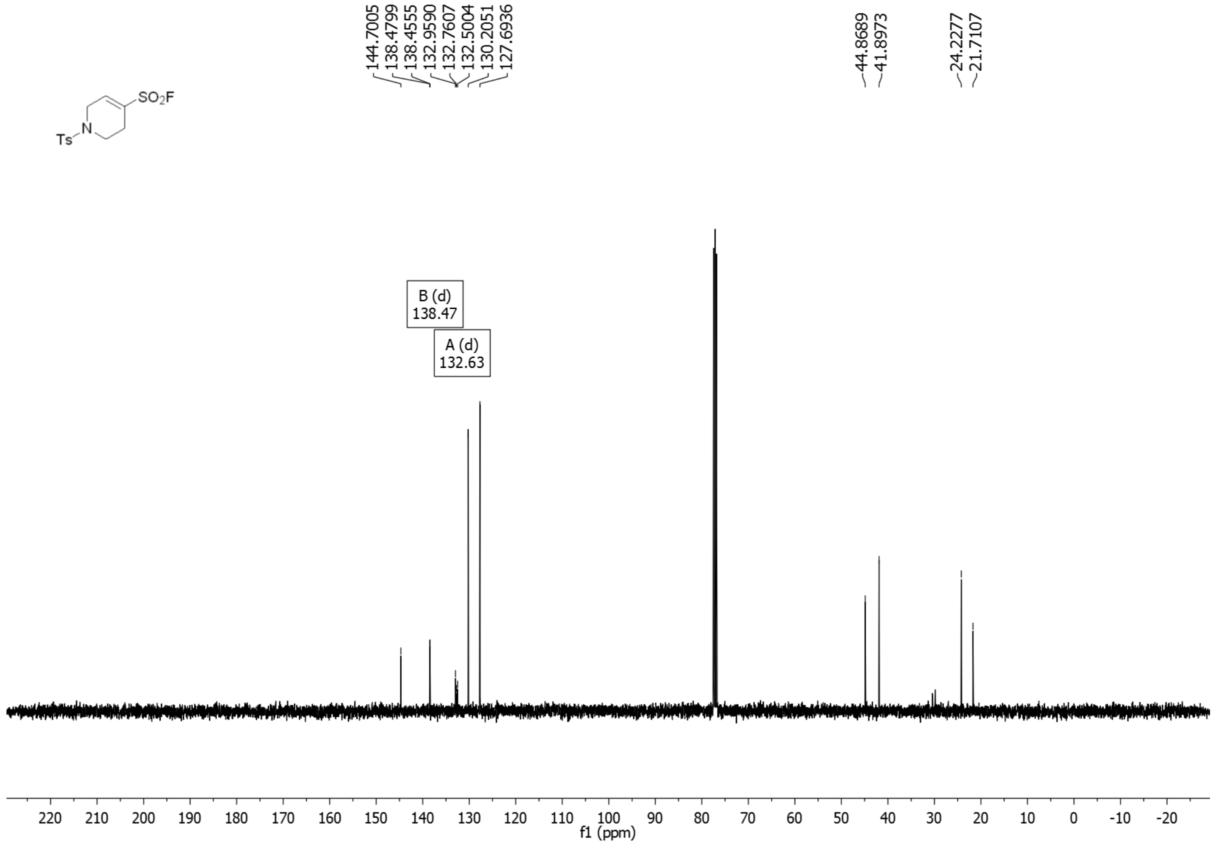

^{19}F-NMR Spectrum:

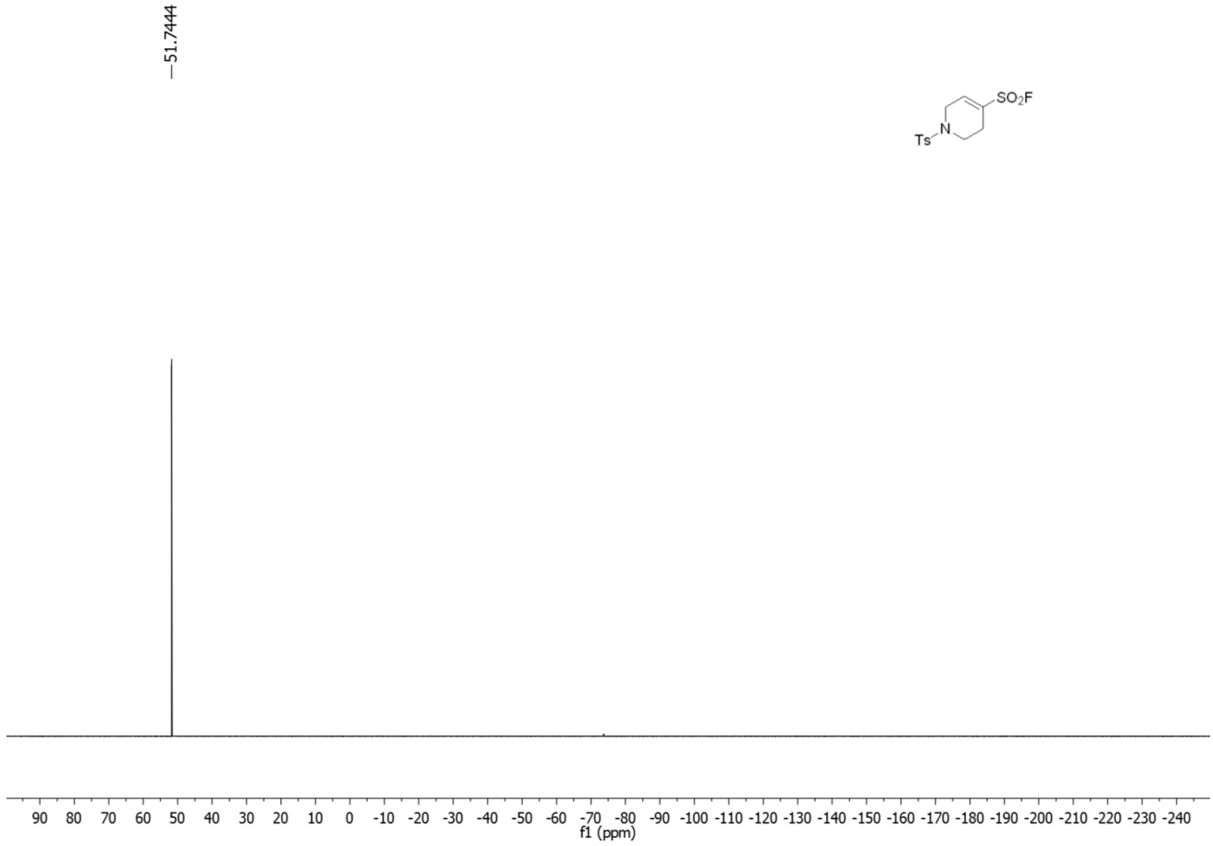

3,4-Dihydronaphthalene-2-sulfonyl fluoride (5u)

^{1}H-NMR Spectrum:

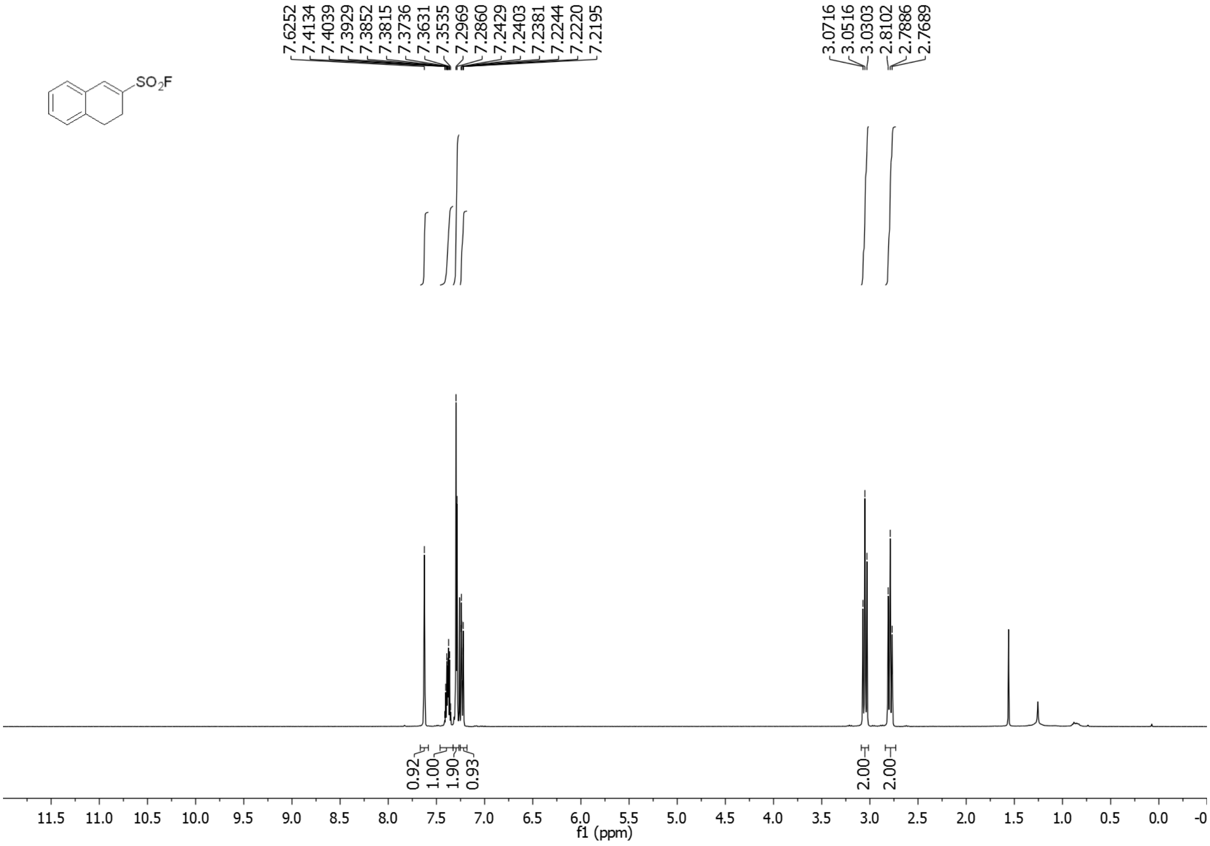

^{13}C-NMR Spectrum:

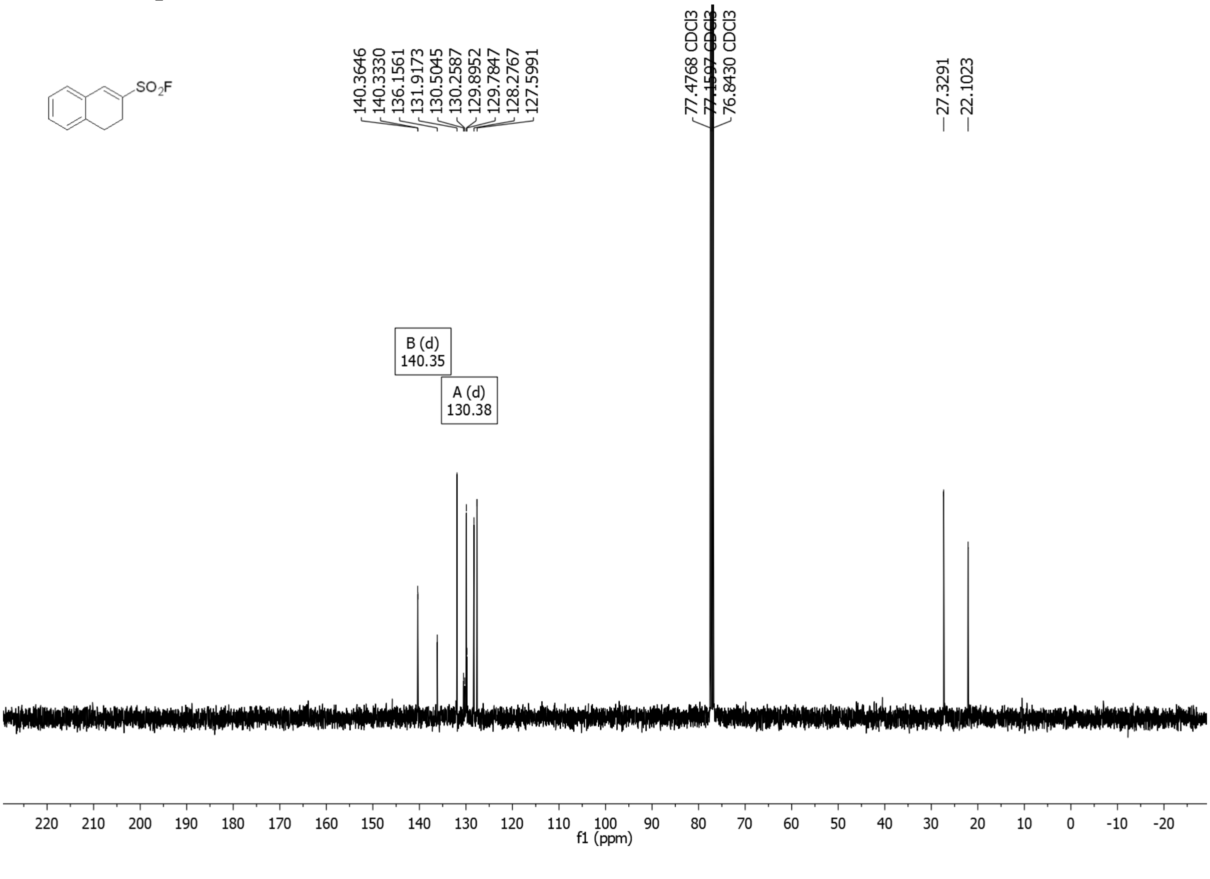

^{19}F-NMR Spectrum:

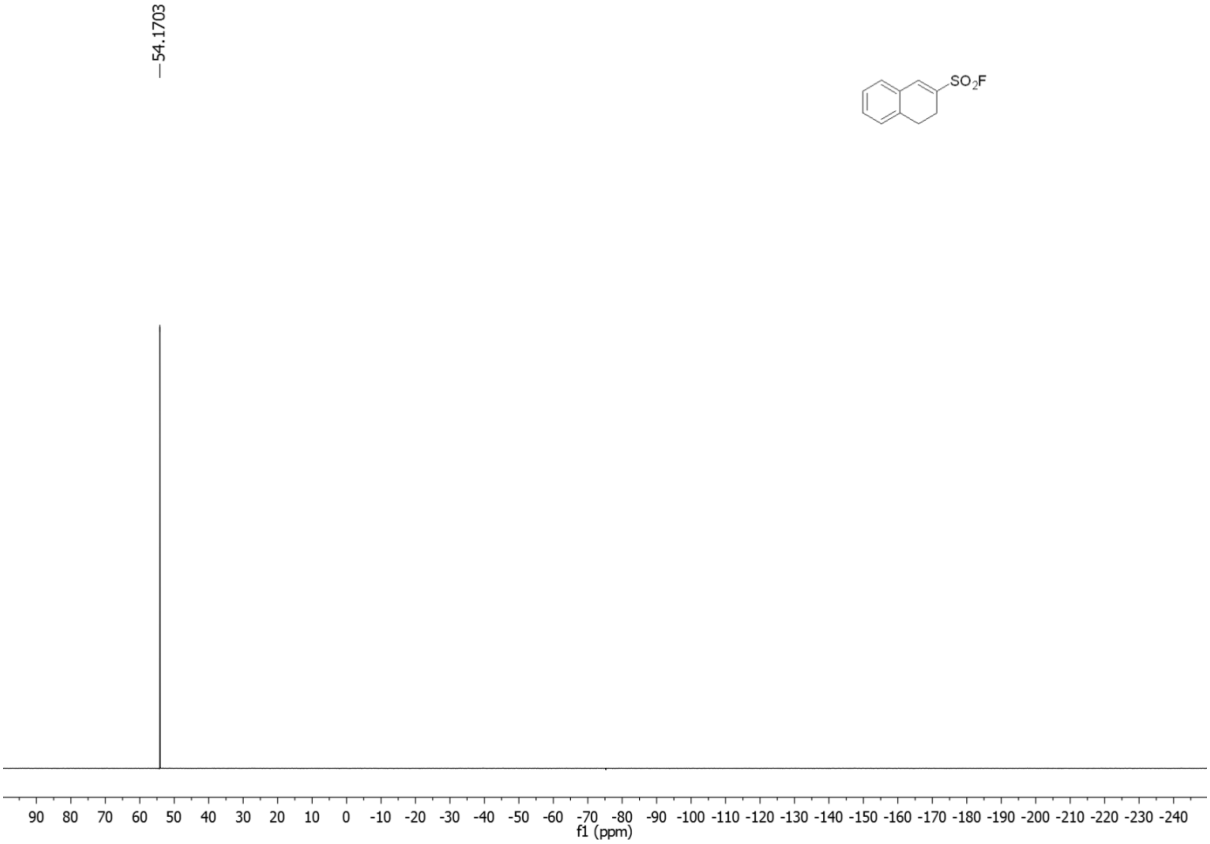

3,4-Dihydronaphthalene-1-sulfonyl fluoride (5v)

^{1}H-NMR Spectrum:

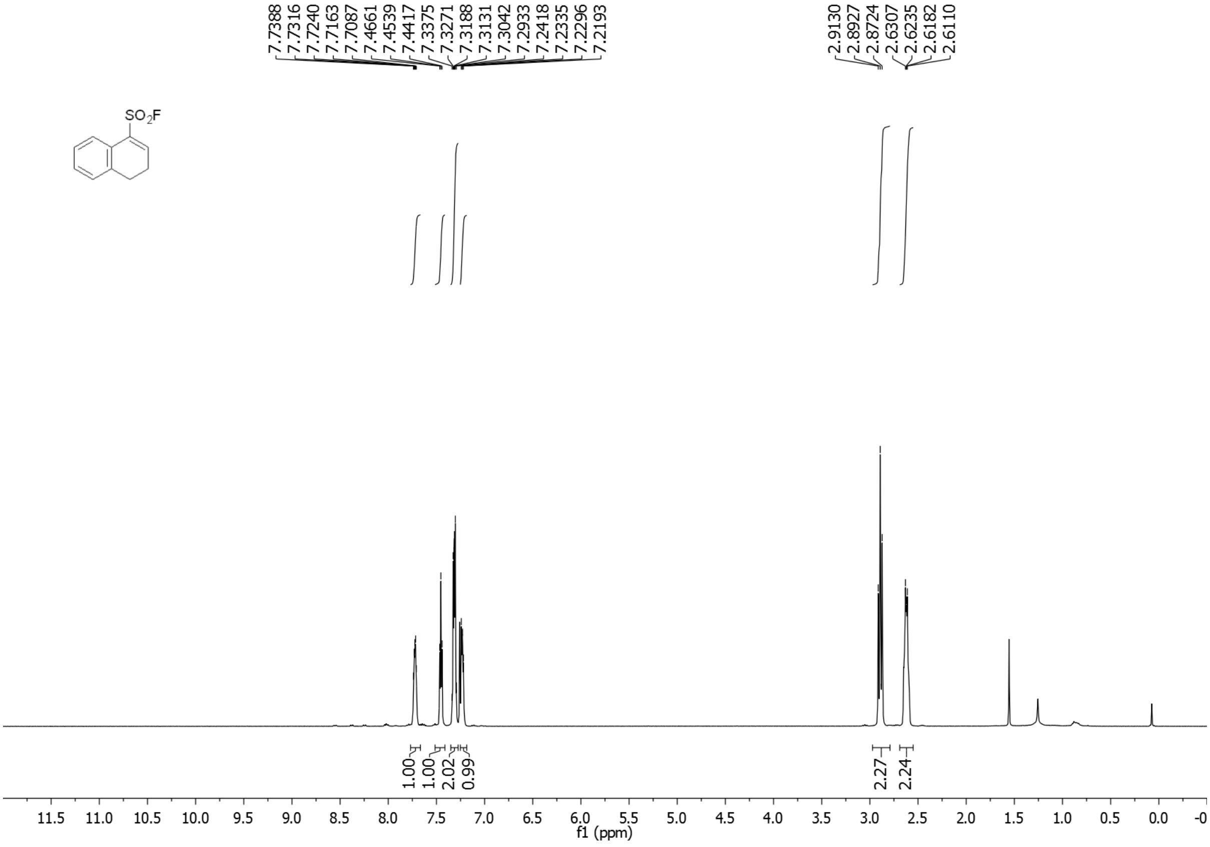

^{13}C-NMR Spectrum:

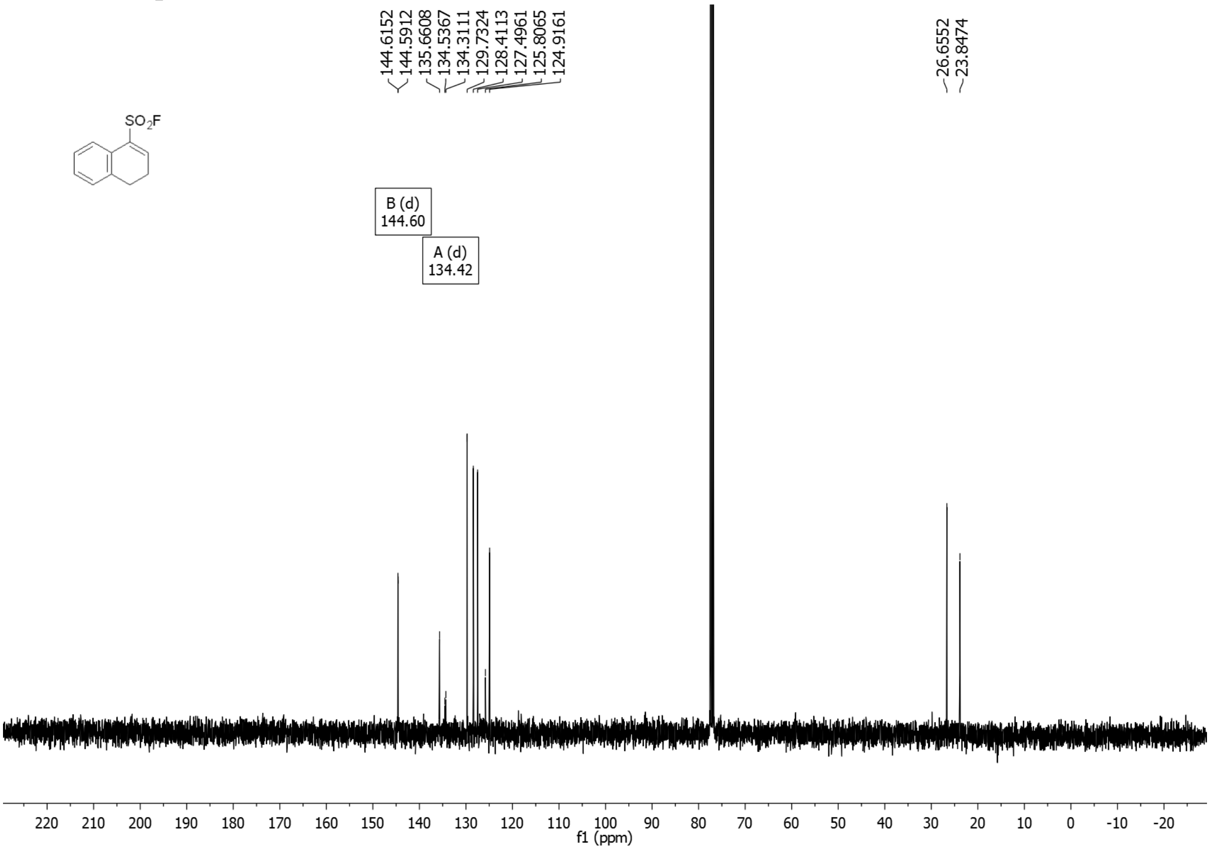

^{19}F-NMR Spectrum:

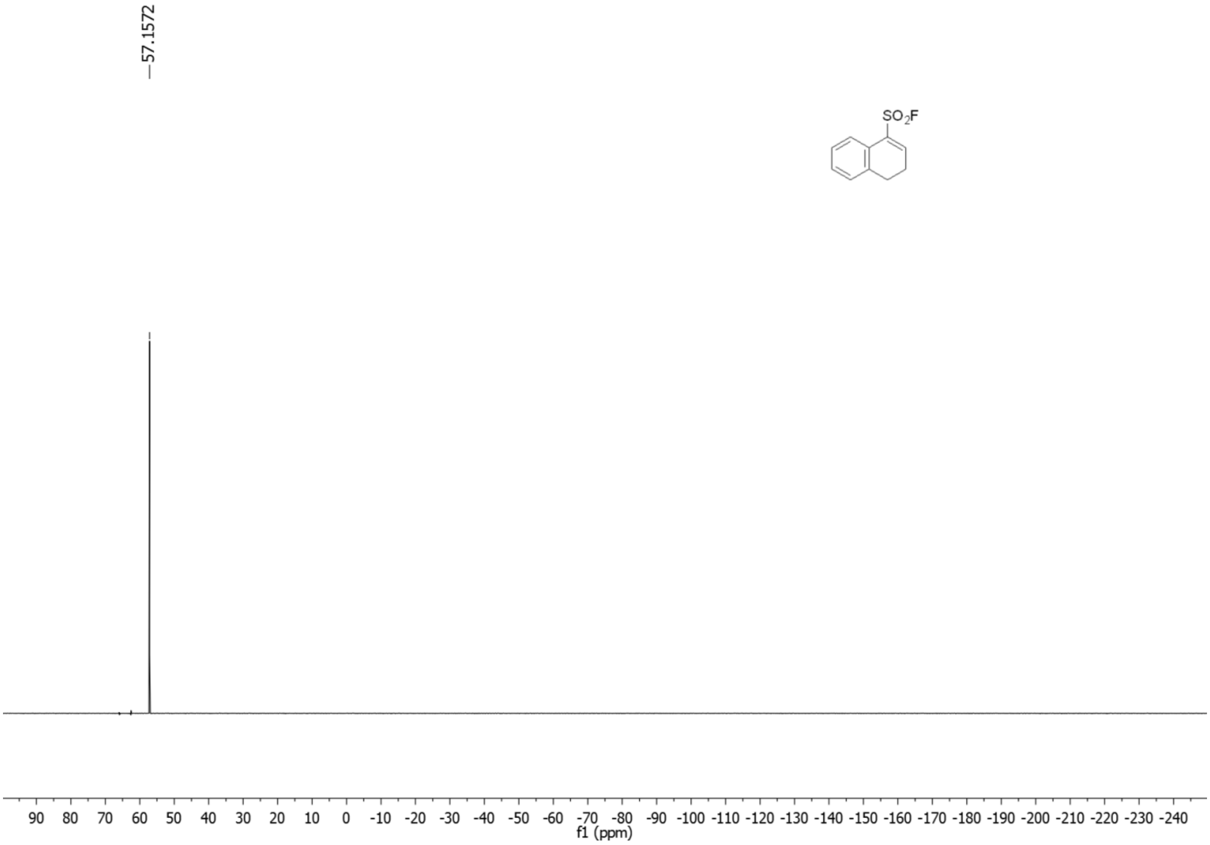

6-Methoxy-3,4-dihydronaphthalene-1-sulfonyl fluoride (5w)

^{1}H-NMR Spectrum:

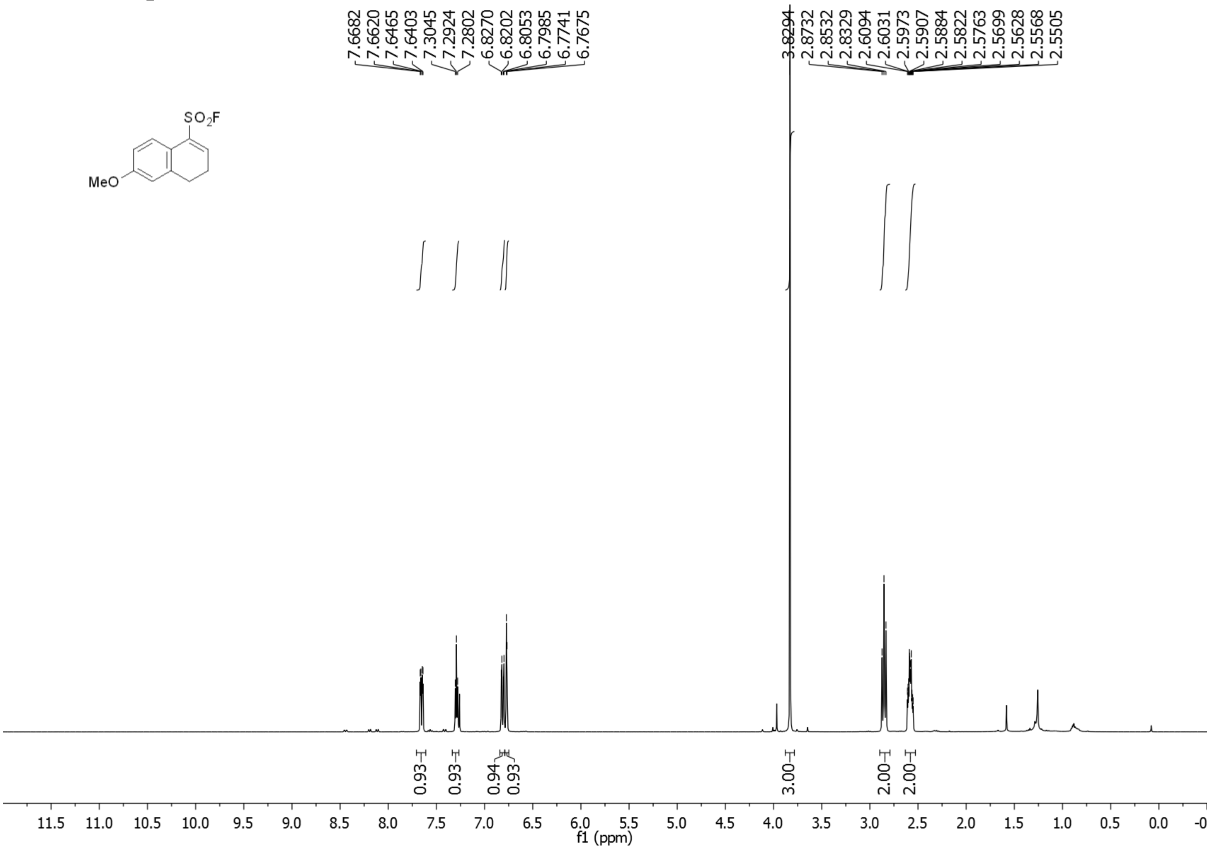

^{13}C-NMR Spectrum:

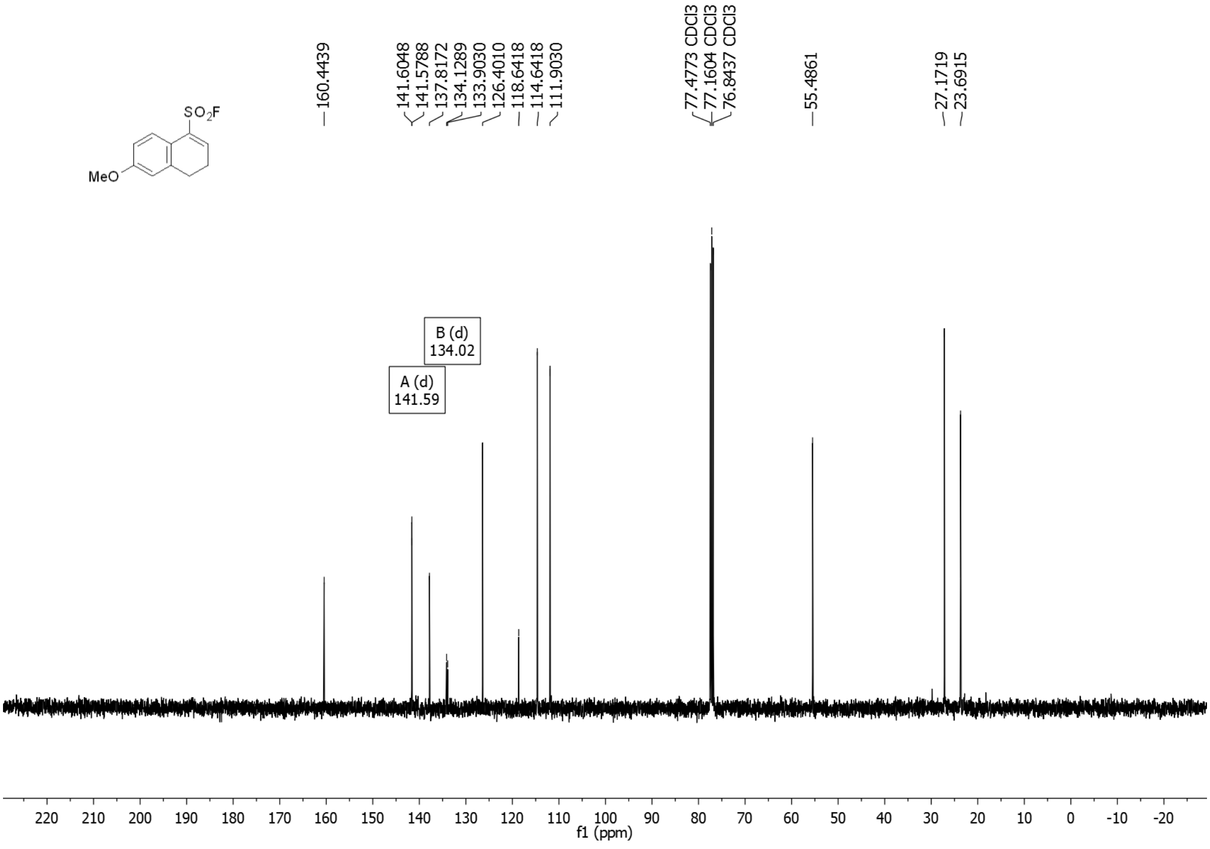

^{19}F-NMR Spectrum:

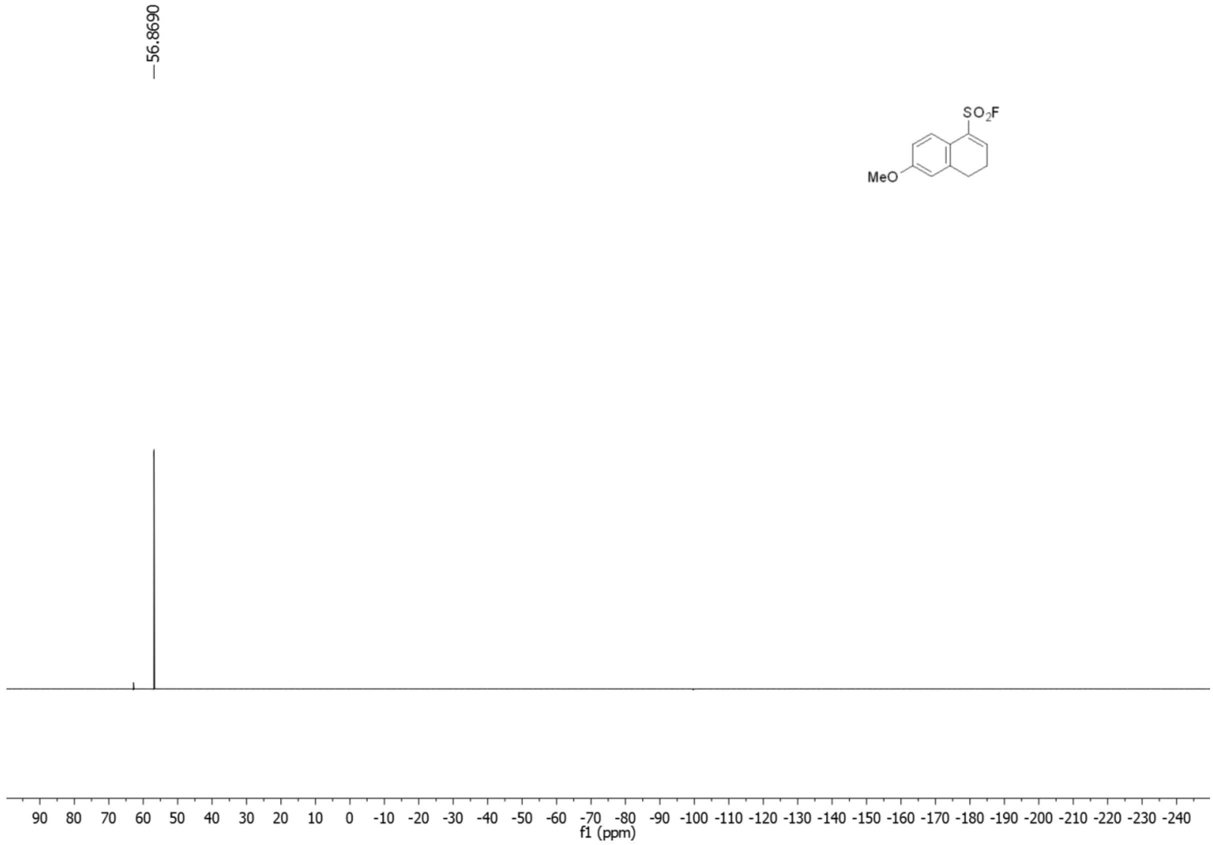

5-(Fluorosulfonyl)-7,8-dihydronaphthalen-2-yl trifluoromethanesulfonate (5x)

^{1}H-NMR Spectrum:

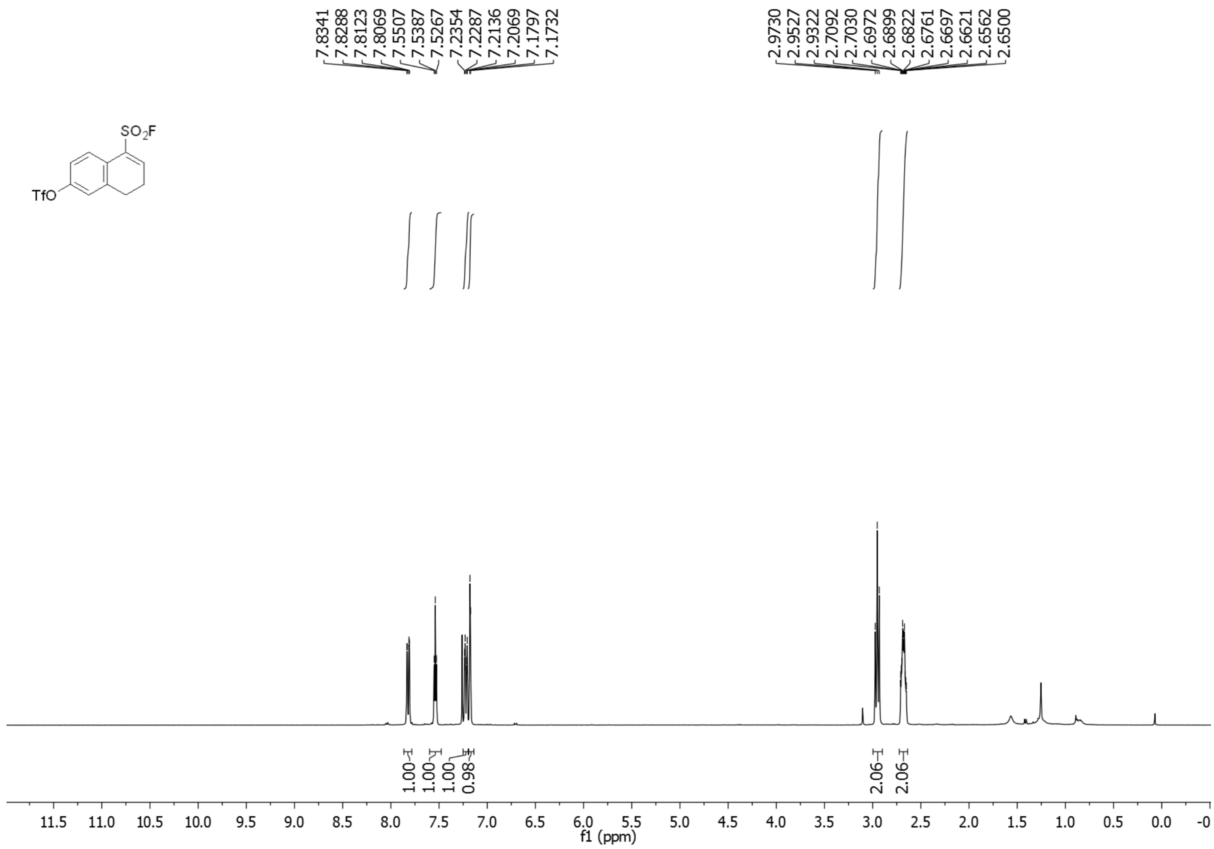

^{13}C-NMR Spectrum:

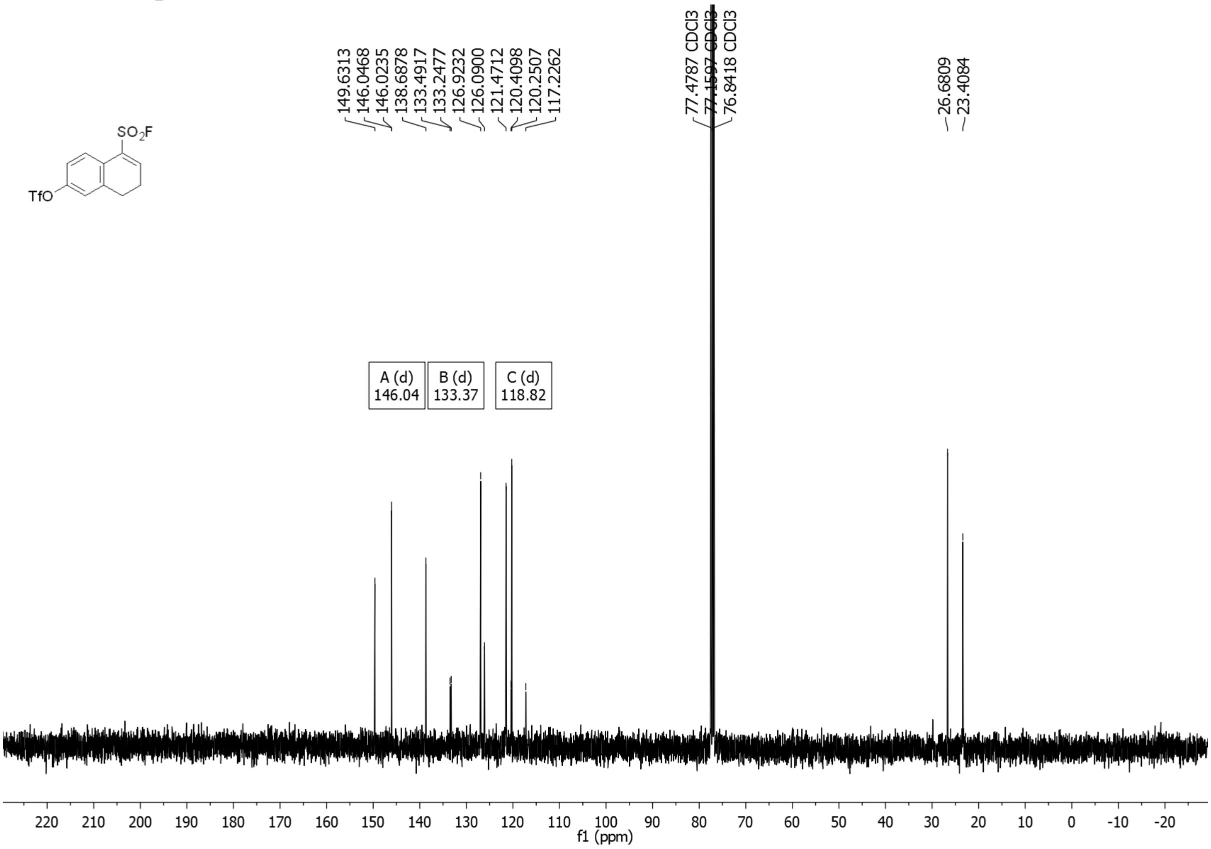

^{19}F-NMR Spectrum:

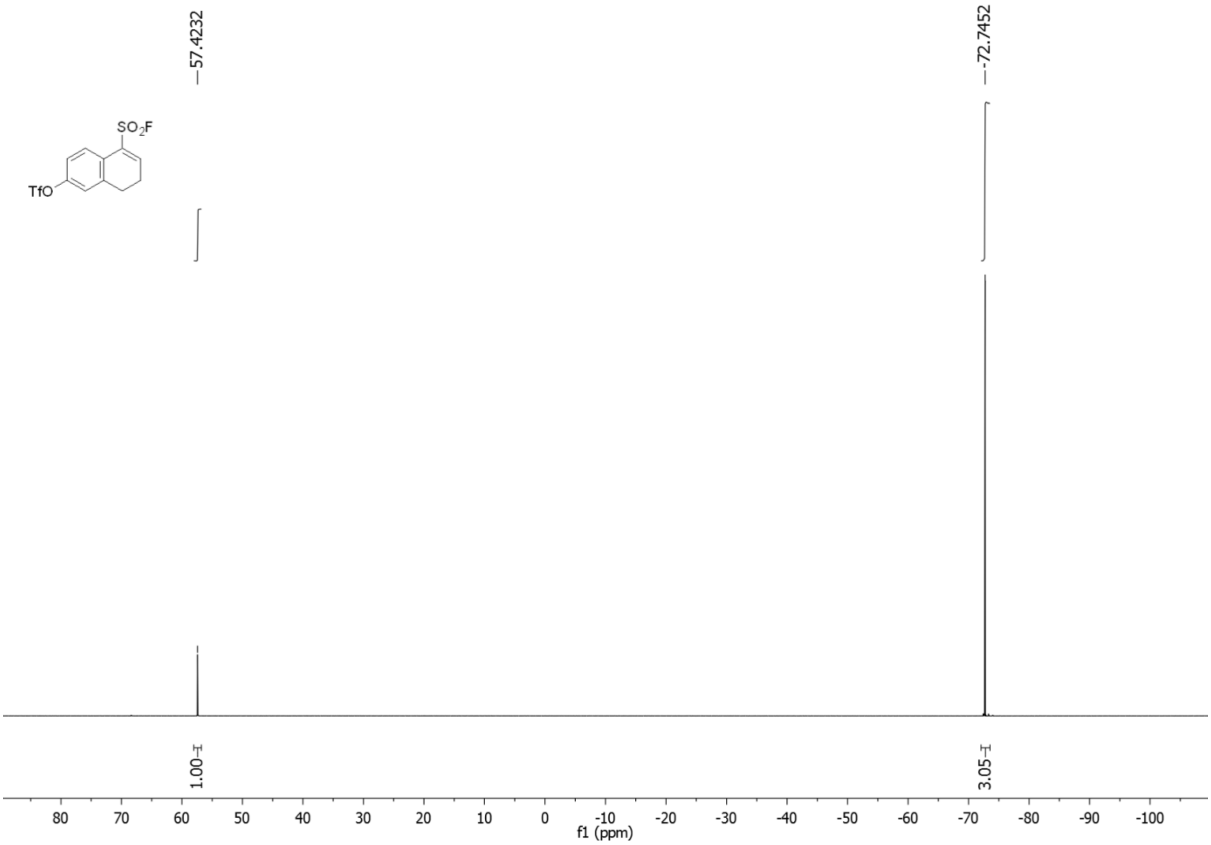

NMR Spectra of Derivatives of Alkenylsulfonyl Fluorides

4-Methoxyphenyl 1,2,3,6-tetrahydro-[1,1'-biphenyl]-4-sulfonate (6)

^{1}H-NMR Spectrum:

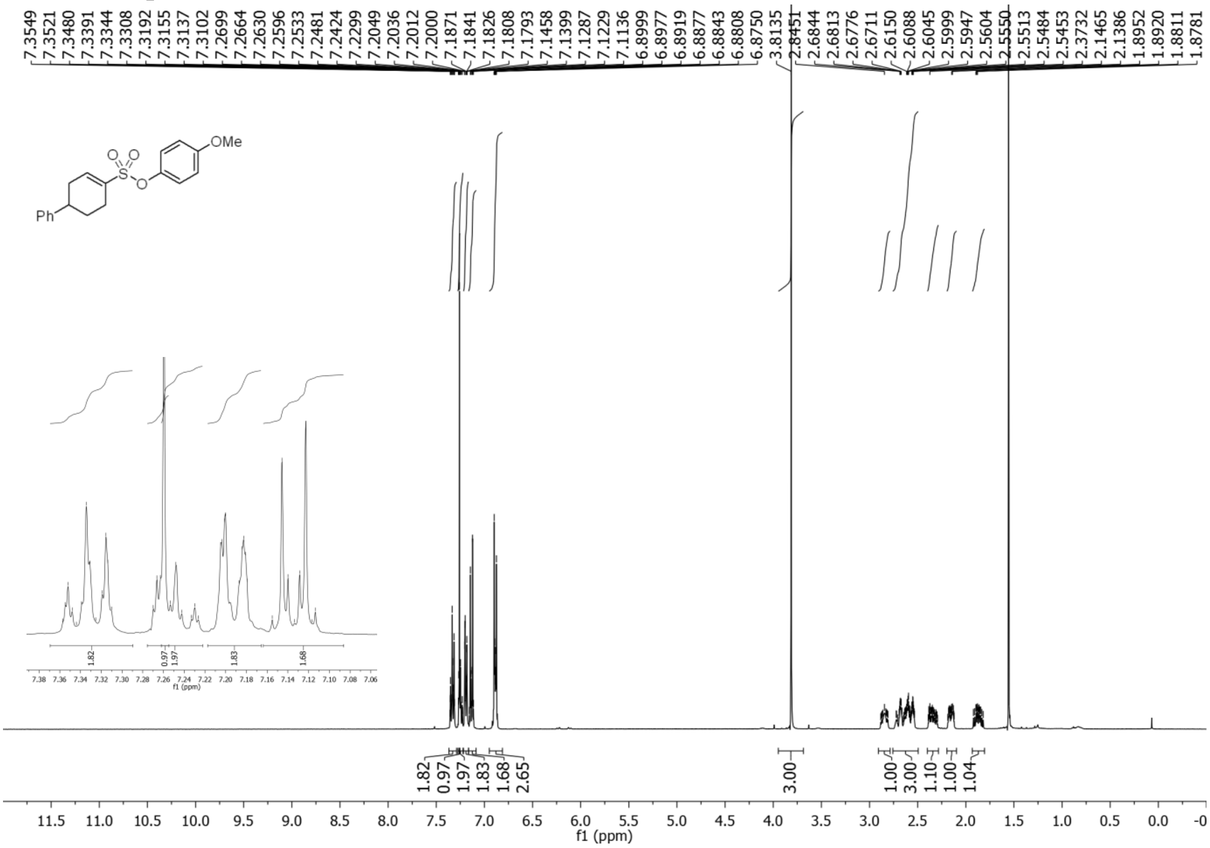

^{13}C-NMR Spectrum:

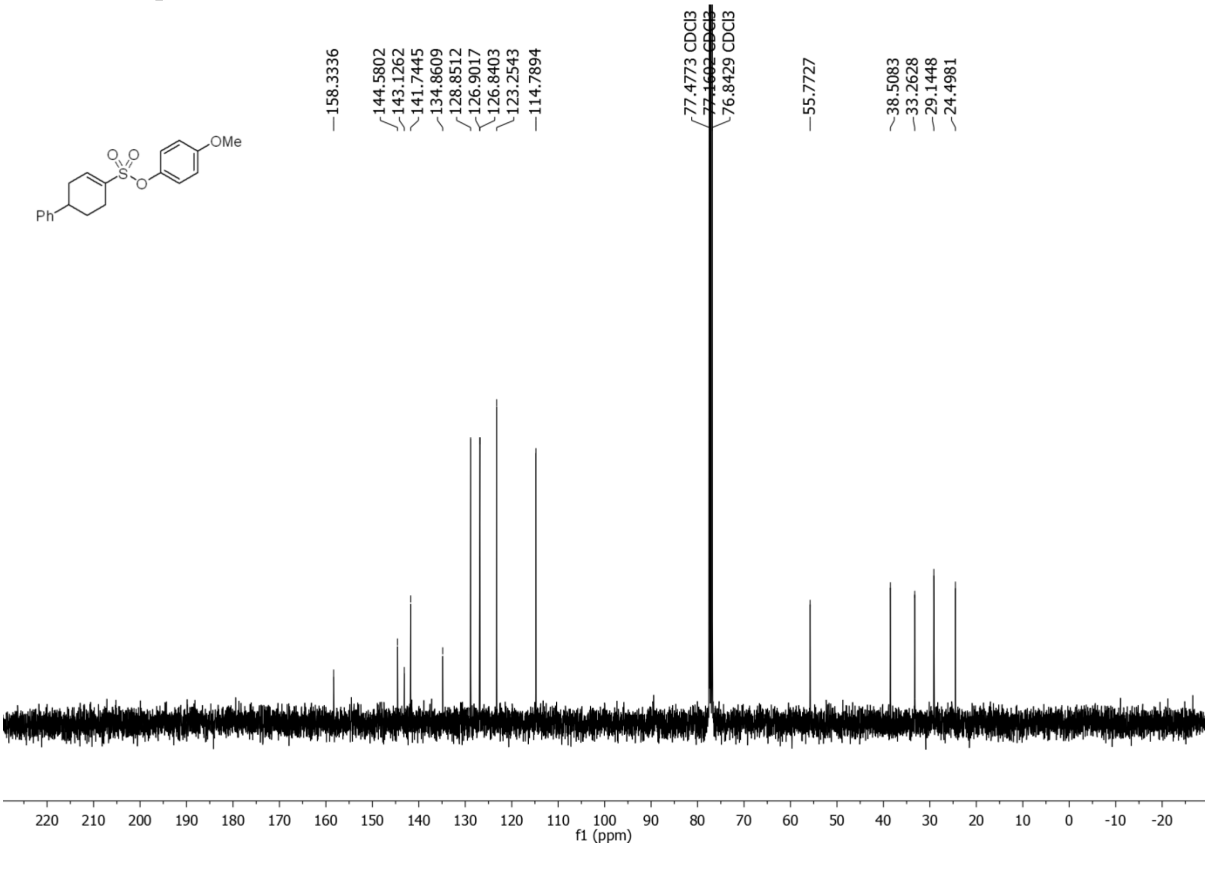

1-((1,2,3,6-Tetrahydro-[1,1'-biphenyl]-4-yl)sulfonyl)pyrrolidine (7)

^{1}H-NMR Spectrum:

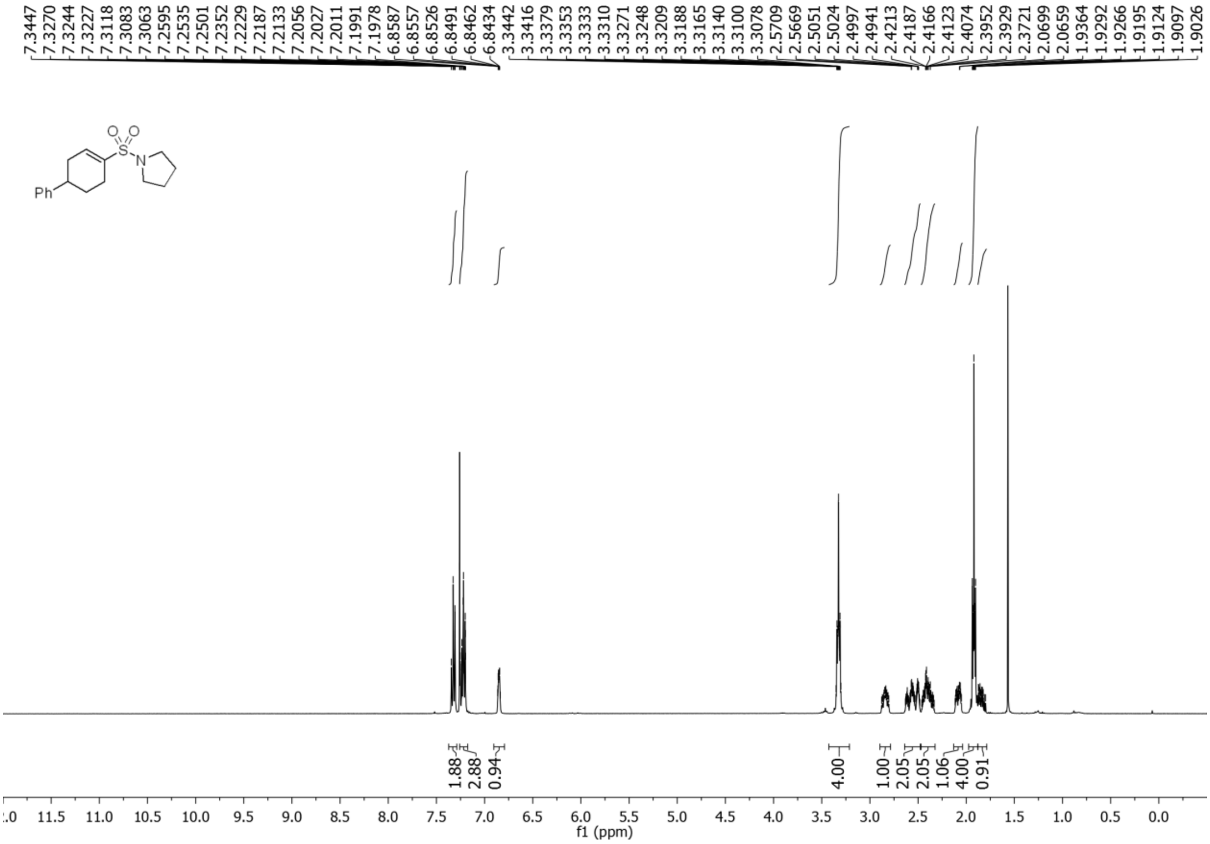

^{13}C-NMR Spectrum:

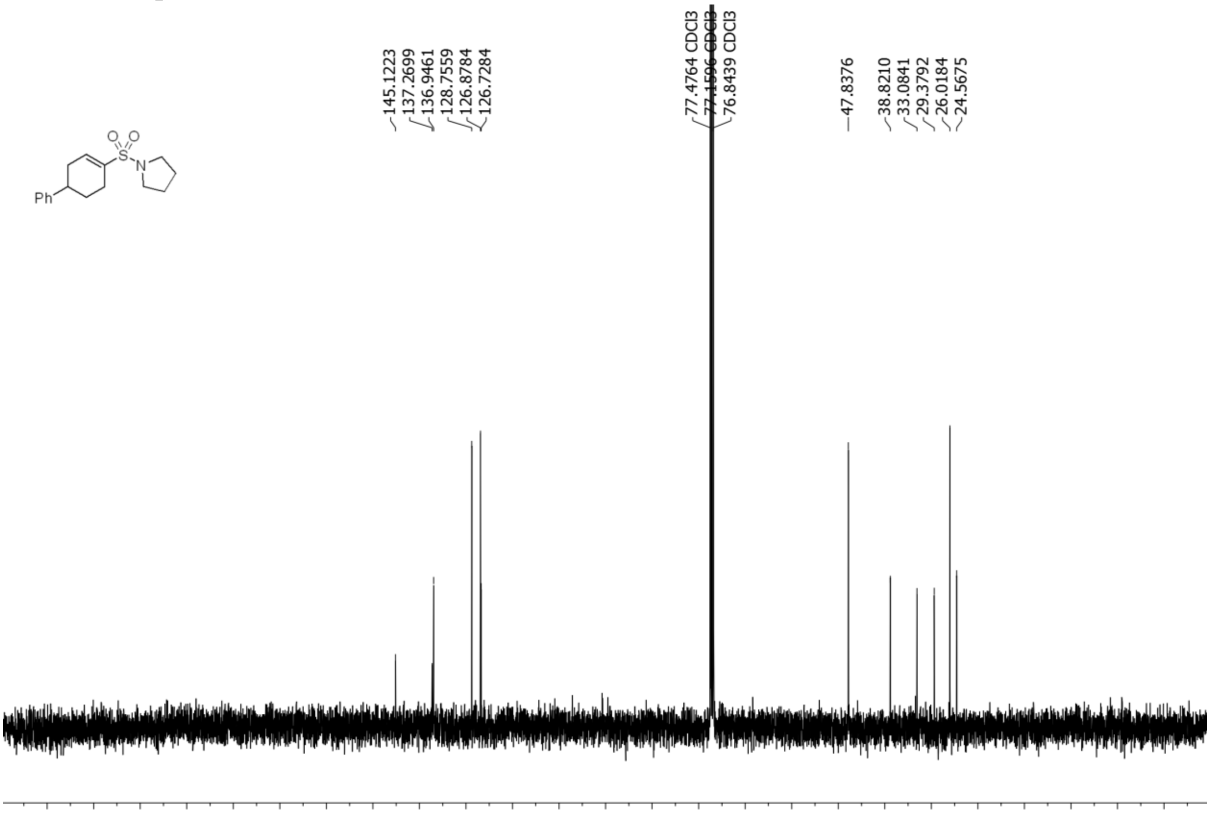

tert-Butyl 4-(_N_-(_p_-tolyl)sulfamoyl)-3,6-dihydropyridine-1(2_H_)-carboxylate (8a)

^{1}H-NMR Spectrum:

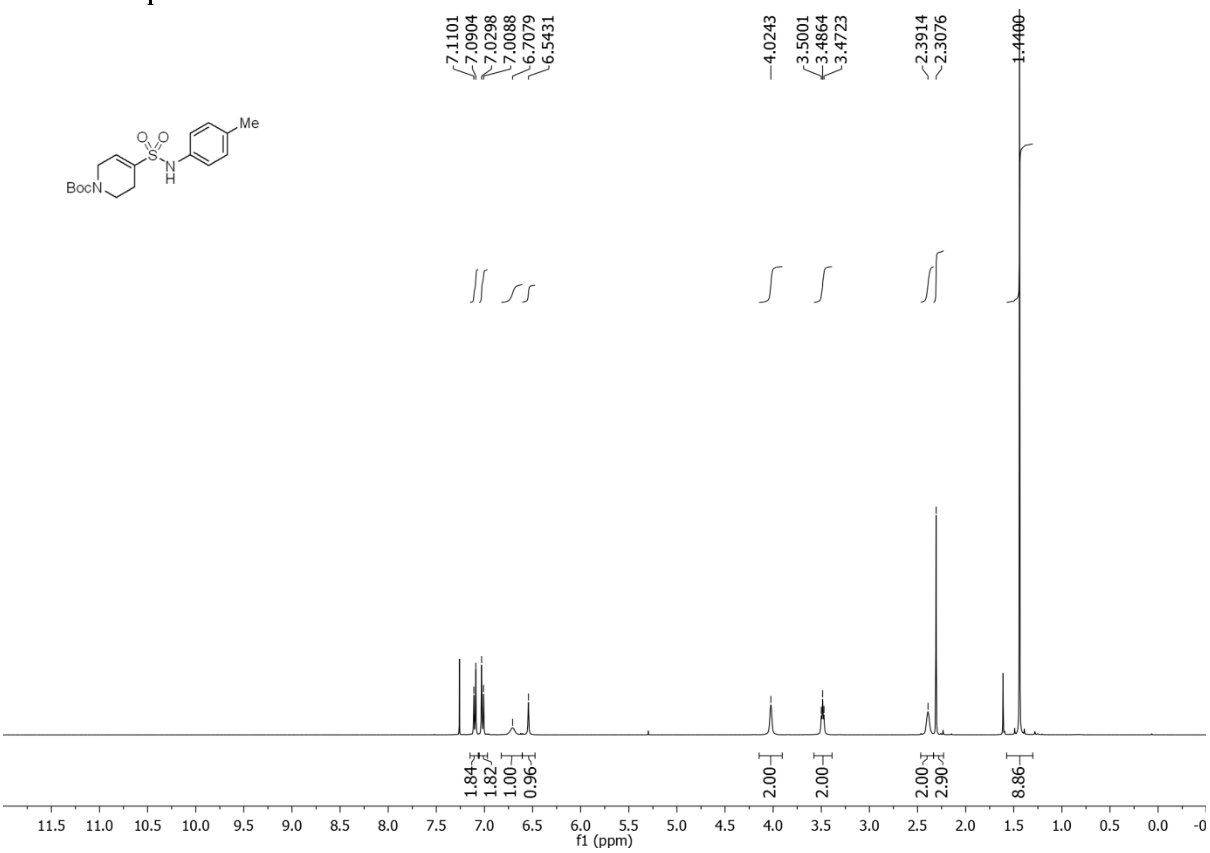

^{13}C-NMR Spectrum:

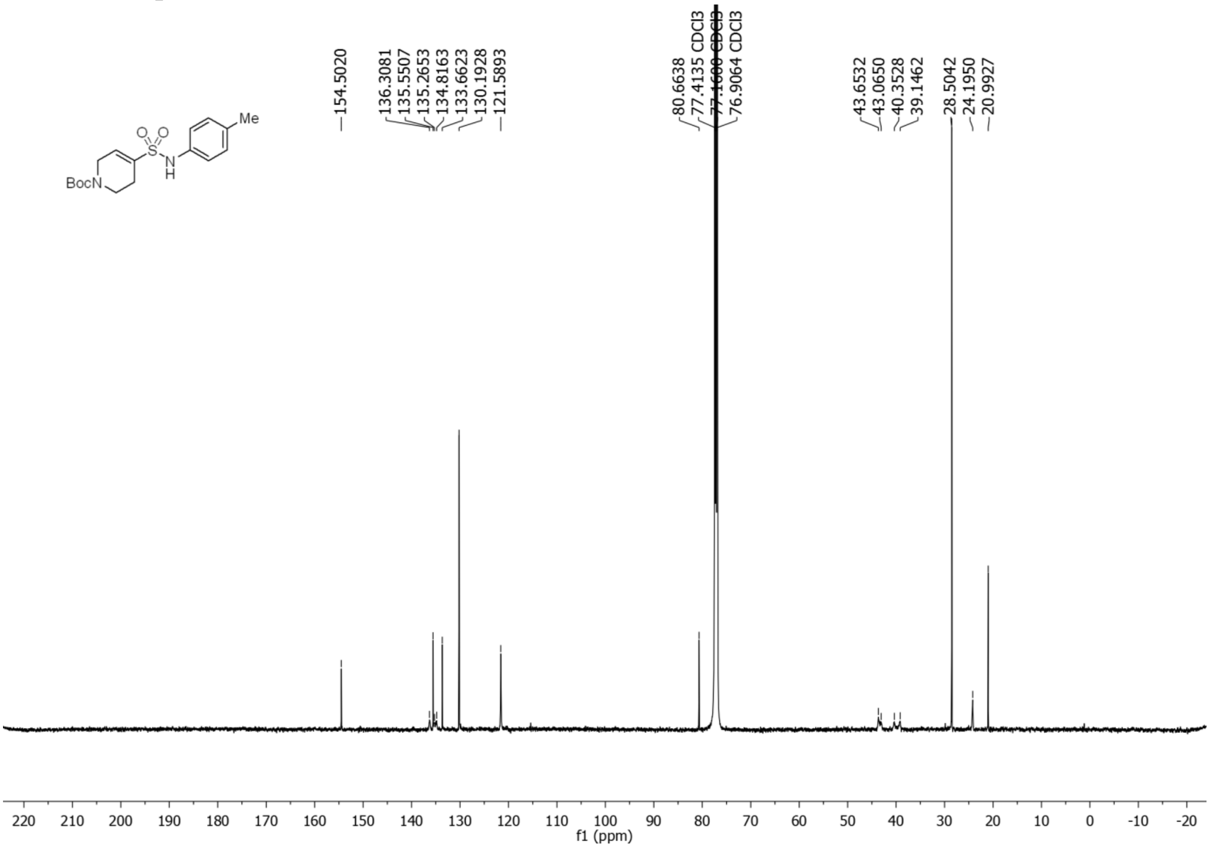

***N*-Phenyl-1-tosyl-1,2,3,6-tetrahydropyridine-4-sulfonamide (8b)**

^{1}H-NMR Spectrum:

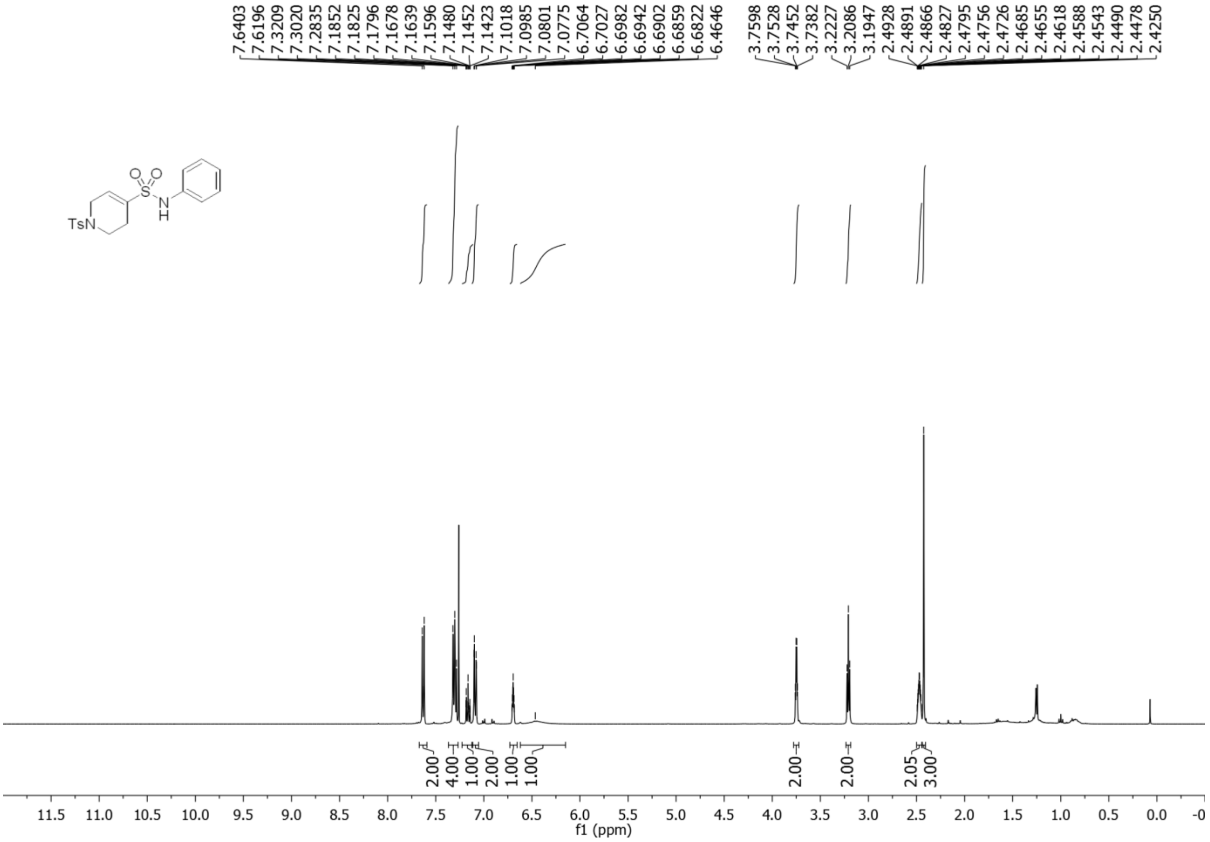

^{13}C-NMR Spectrum:

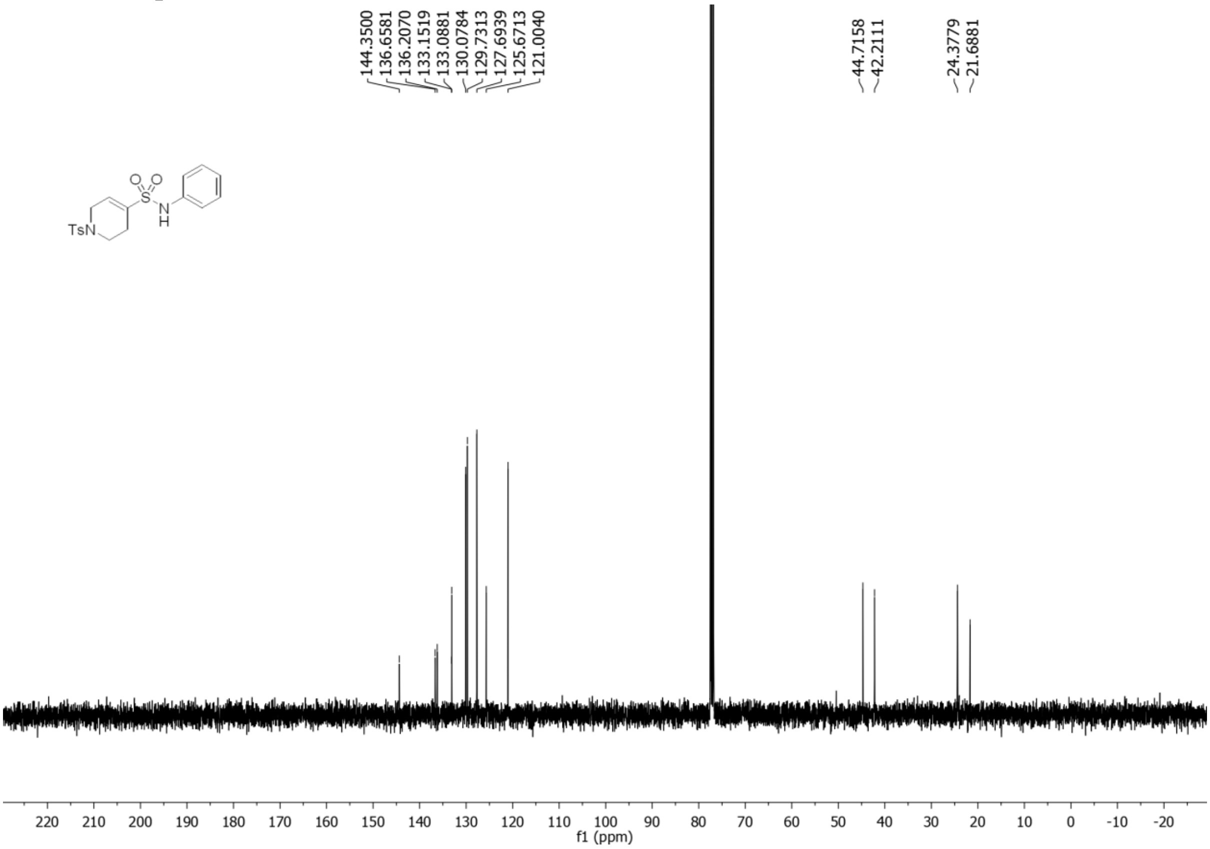

***cis-tert*-Butyl 3-((4-fluorophenyl)thio)-4-(fluorosulfonyl)piperidine-1-carboxylate (9a1)**

^{1}H- NMR Spectrum (363 K):

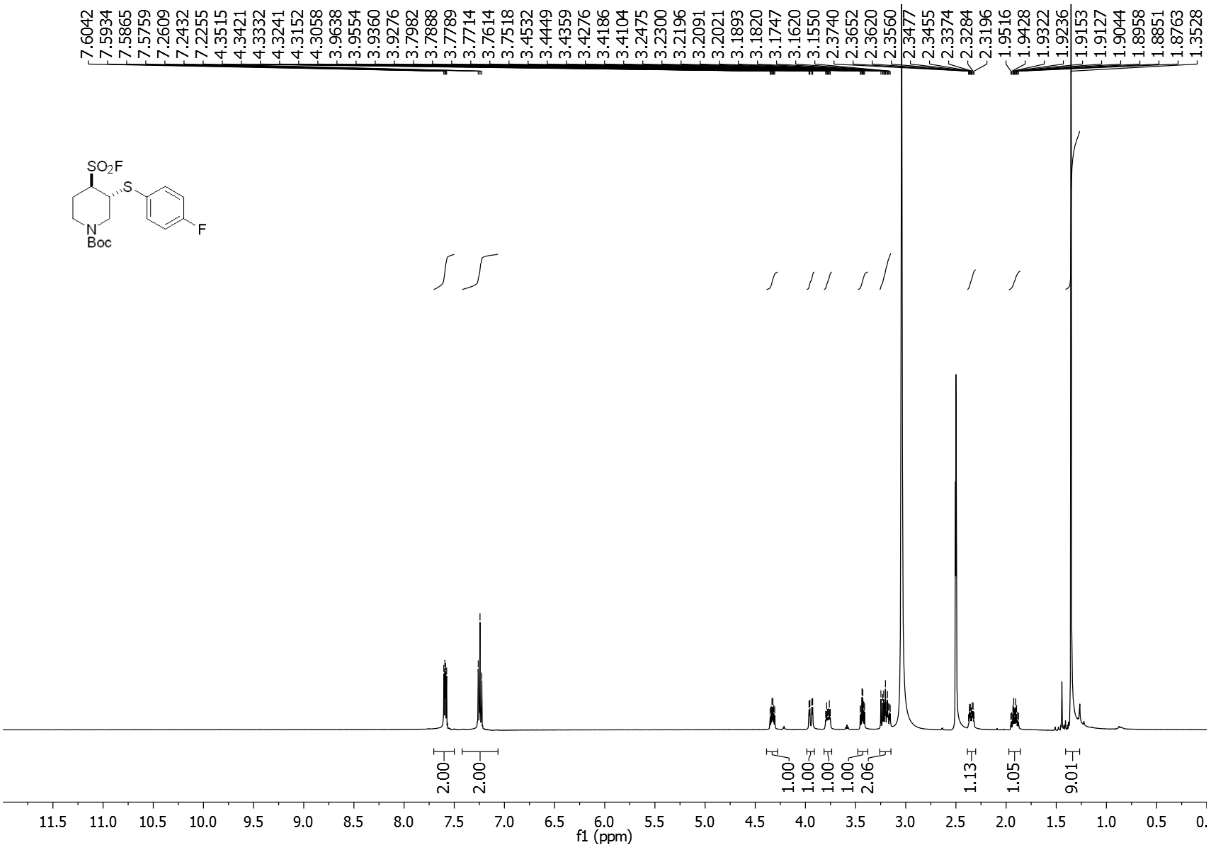

^{13}C- NMR Spectrum (363 K):

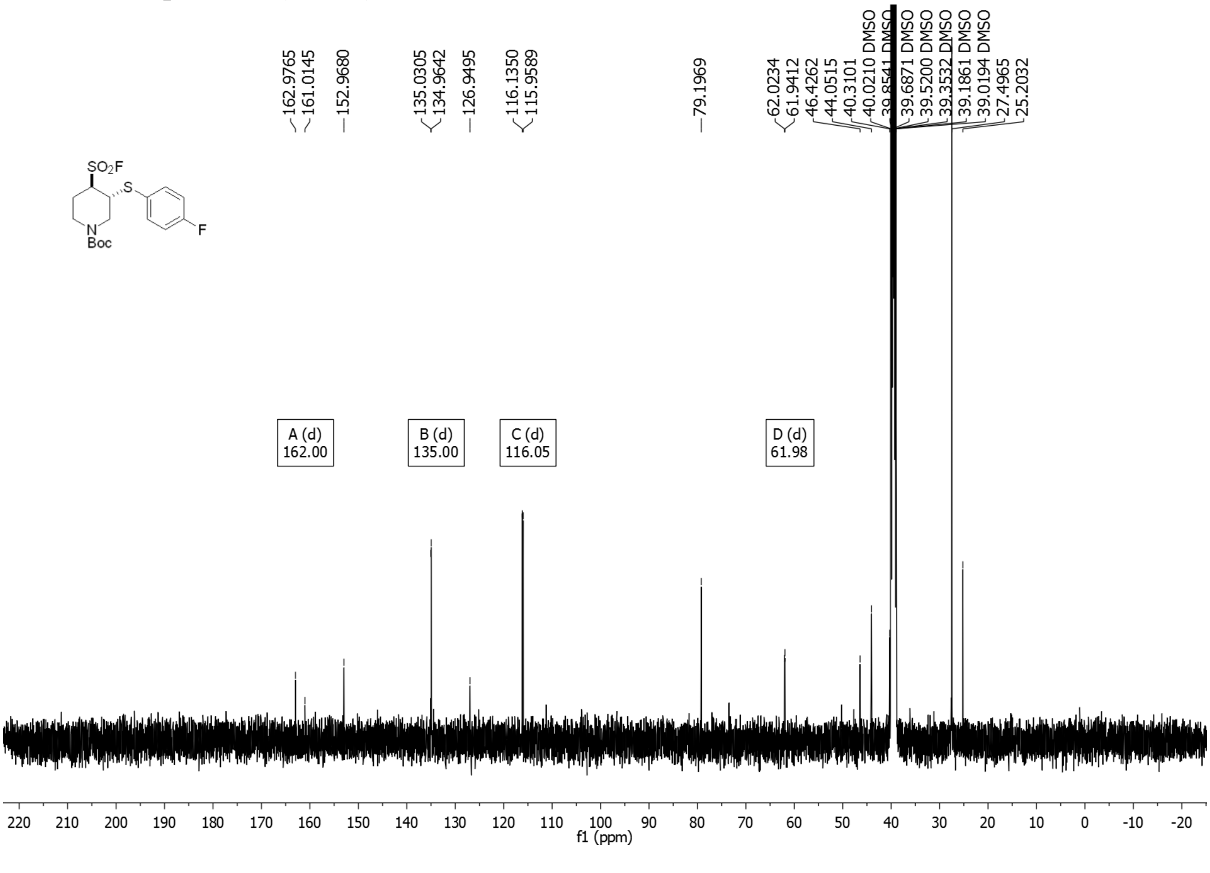

^{19}F-NMR Spectrum (363 K):

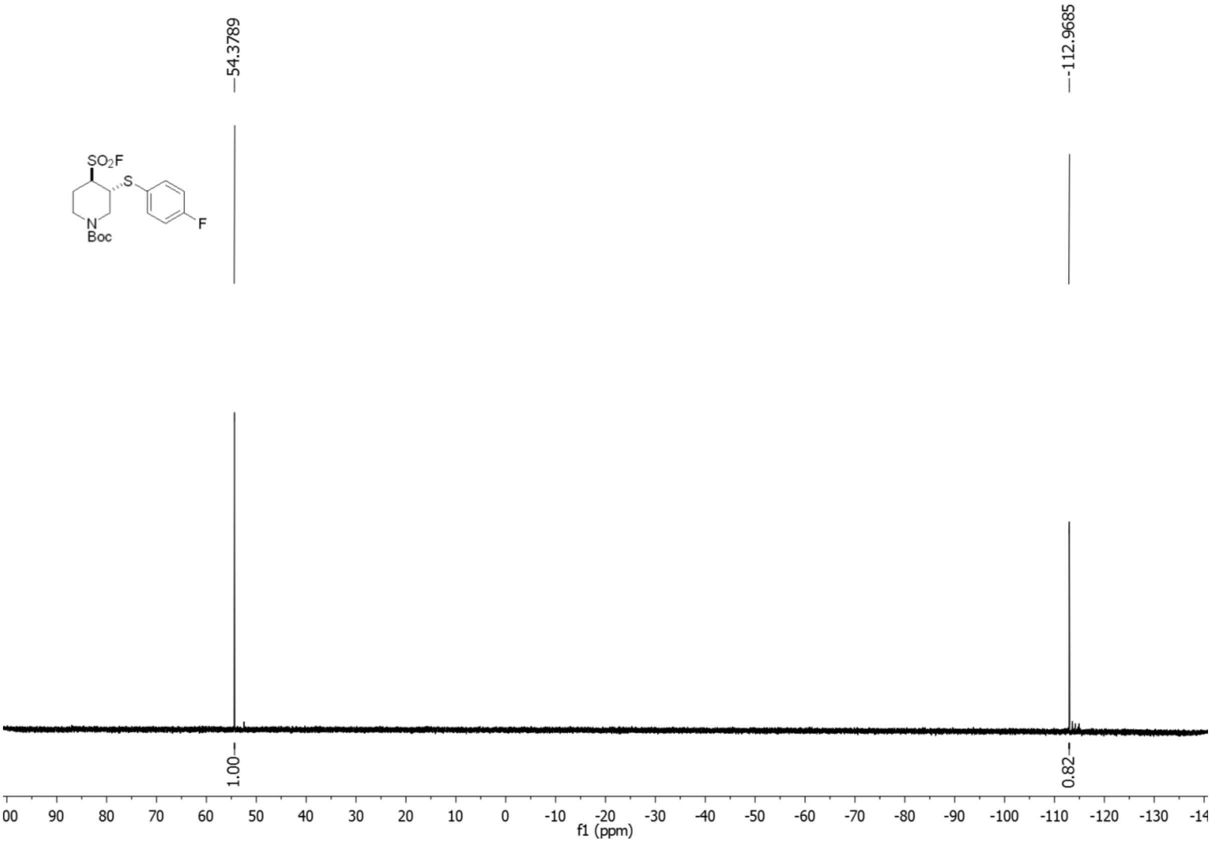

^{1}H-^{1}H NOESY NMR Spectrum (363 K):

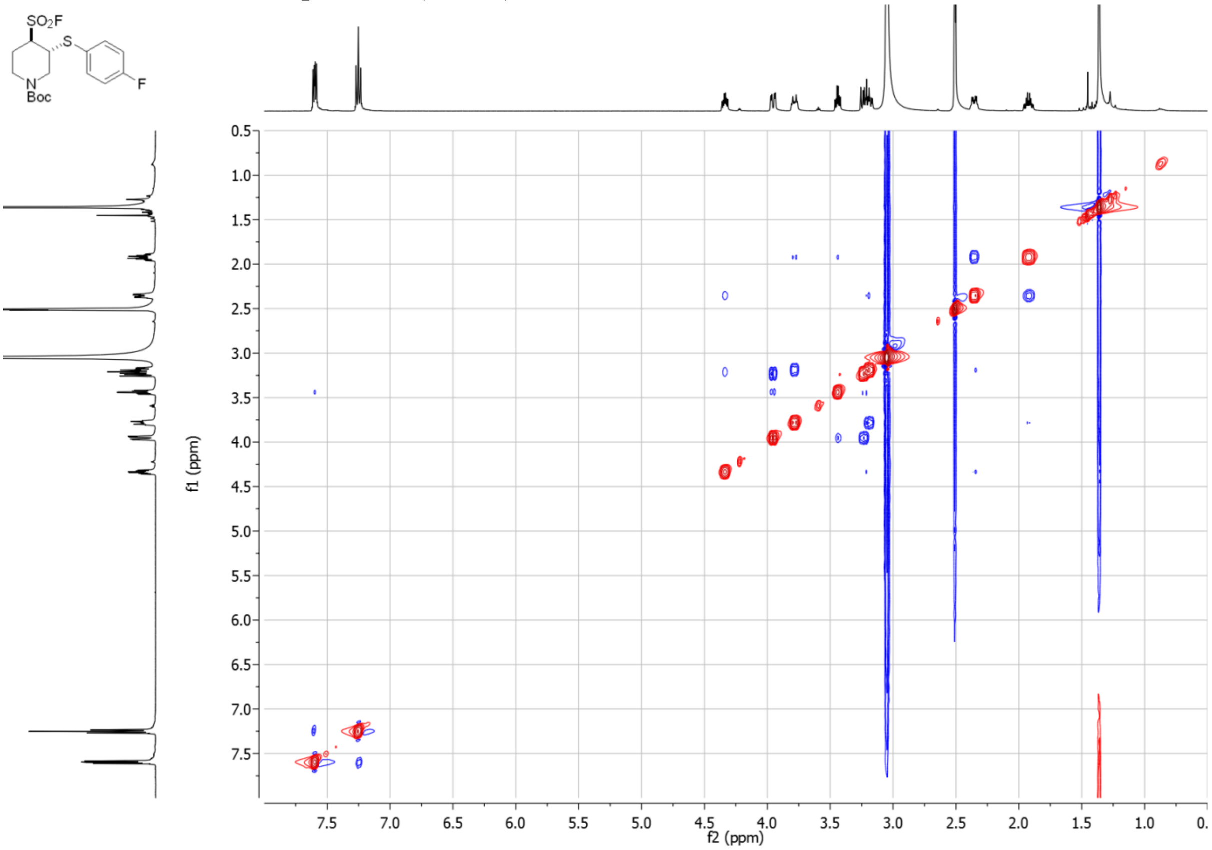

***trans-tert*-Butyl 3-((4-fluorophenyl)thio)-4-(fluorosulfonyl)piperidine-1-carboxylate (9a2)**

^{1}H- NMR Spectrum (363K):

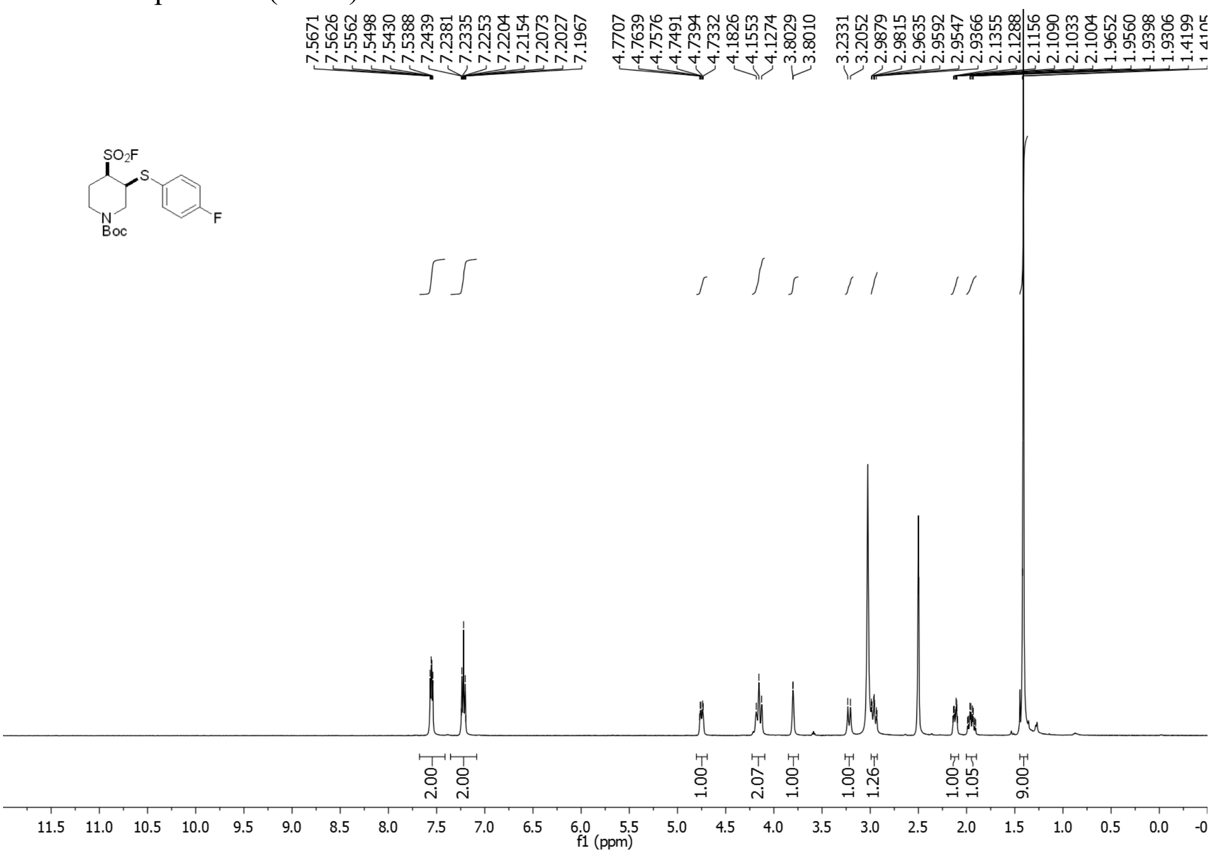

^{13}C- NMR Spectrum (363K):

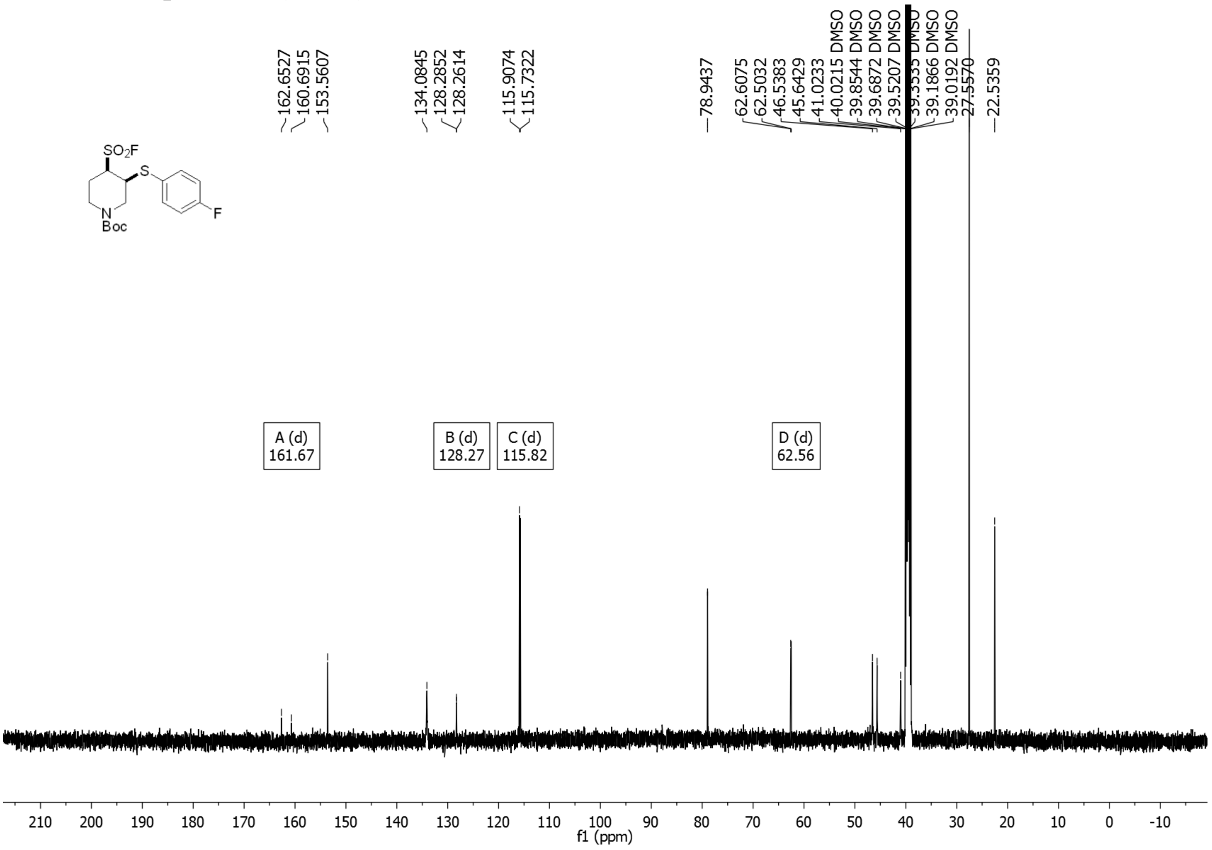

^{19}F-NMR Spectrum (363K):

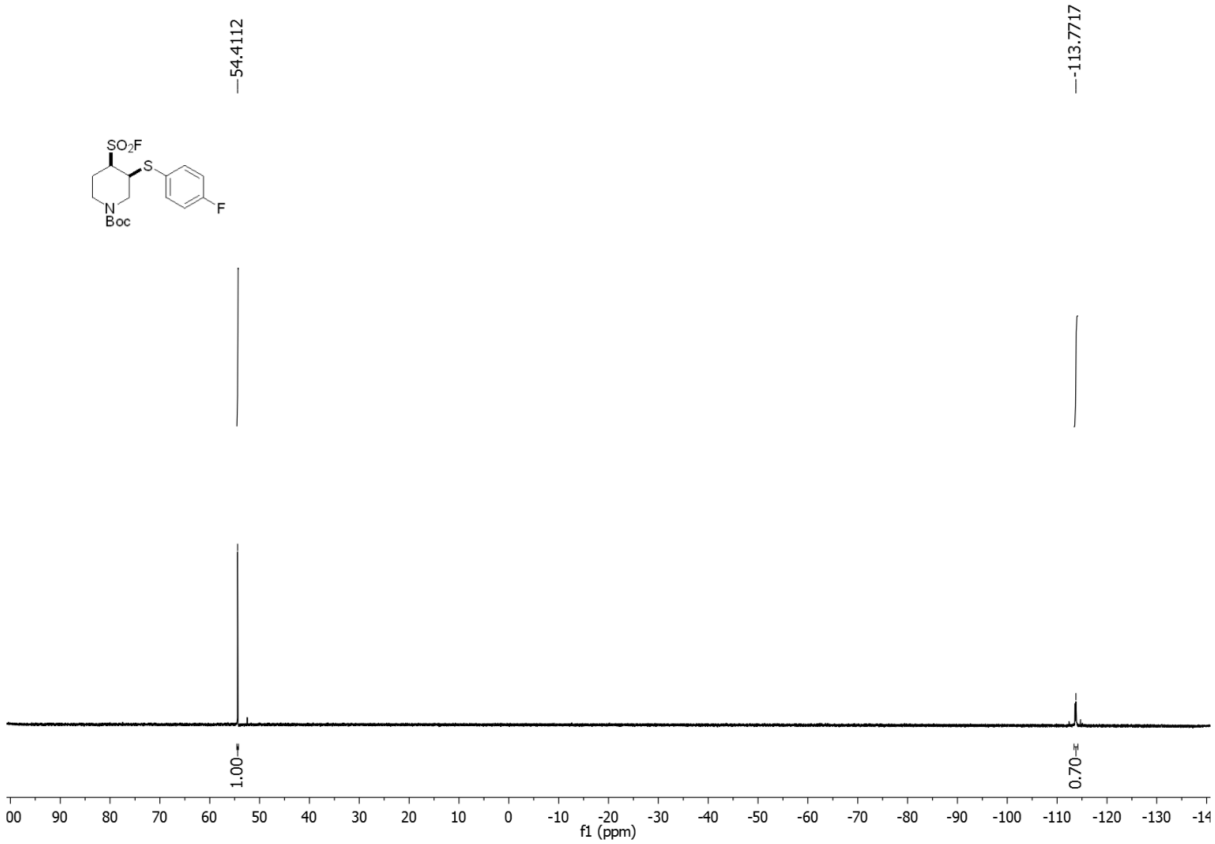

^{1}H-^{1}H NOESY NMR Spectrum (363 K):

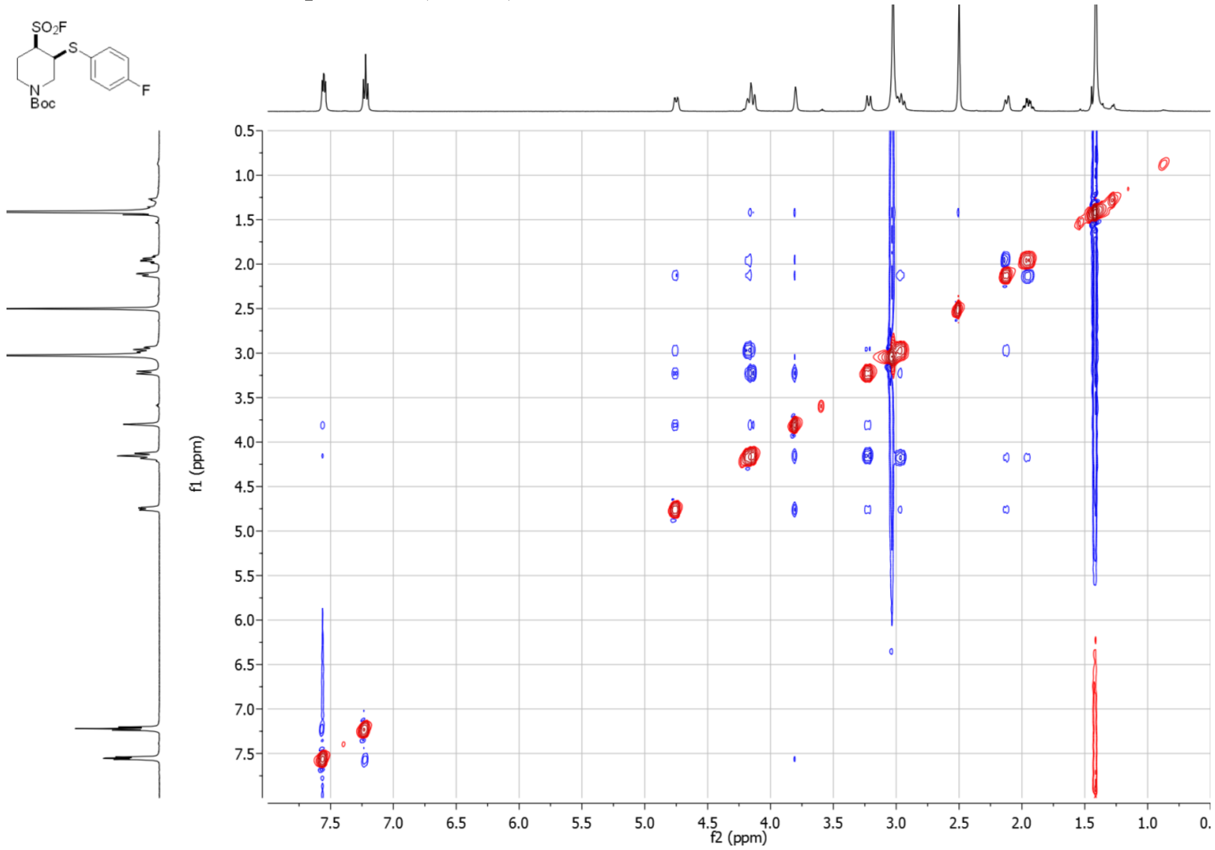

trans-3-((4-Fluorophenyl)thio)-1-tosylpiperidine-4-sulfonyl fluoride (9b1)

^{1}H-NMR Spectrum:

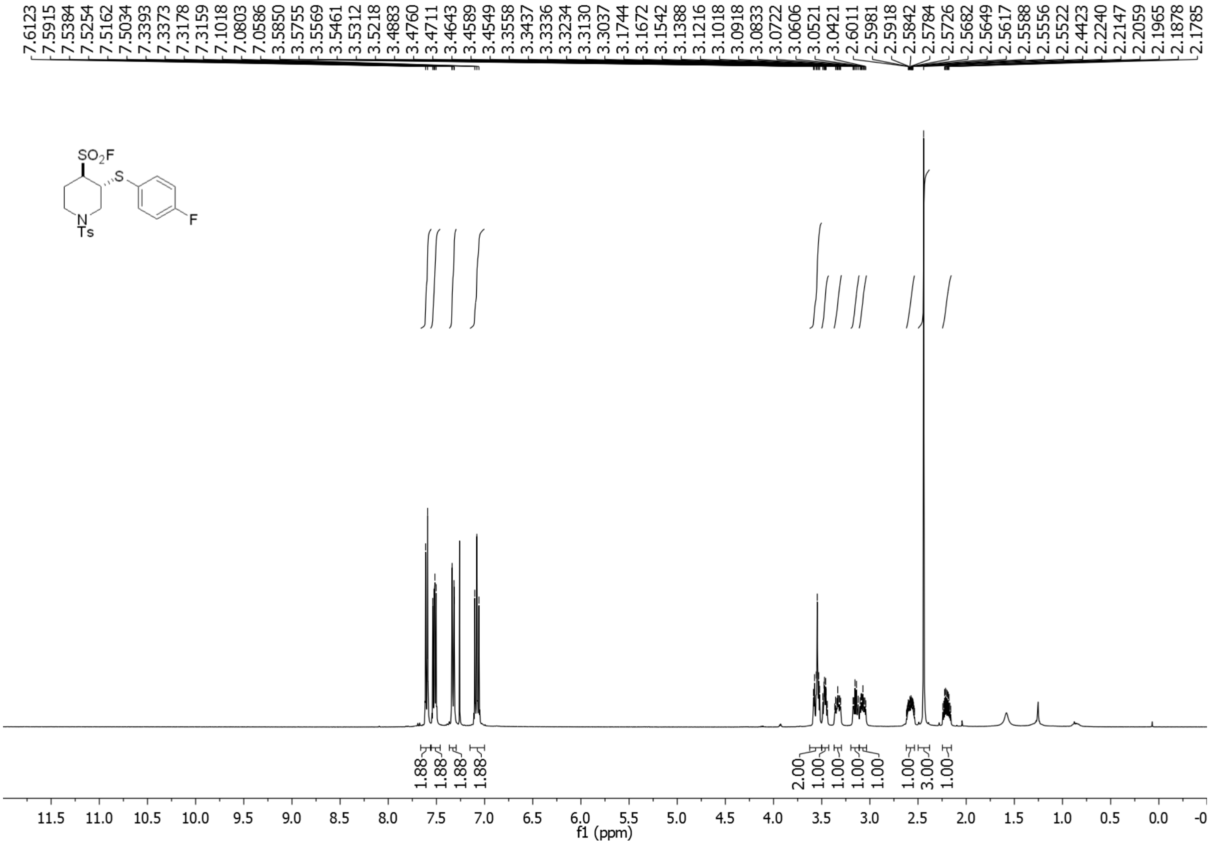

^{13}C-NMR Spectrum:

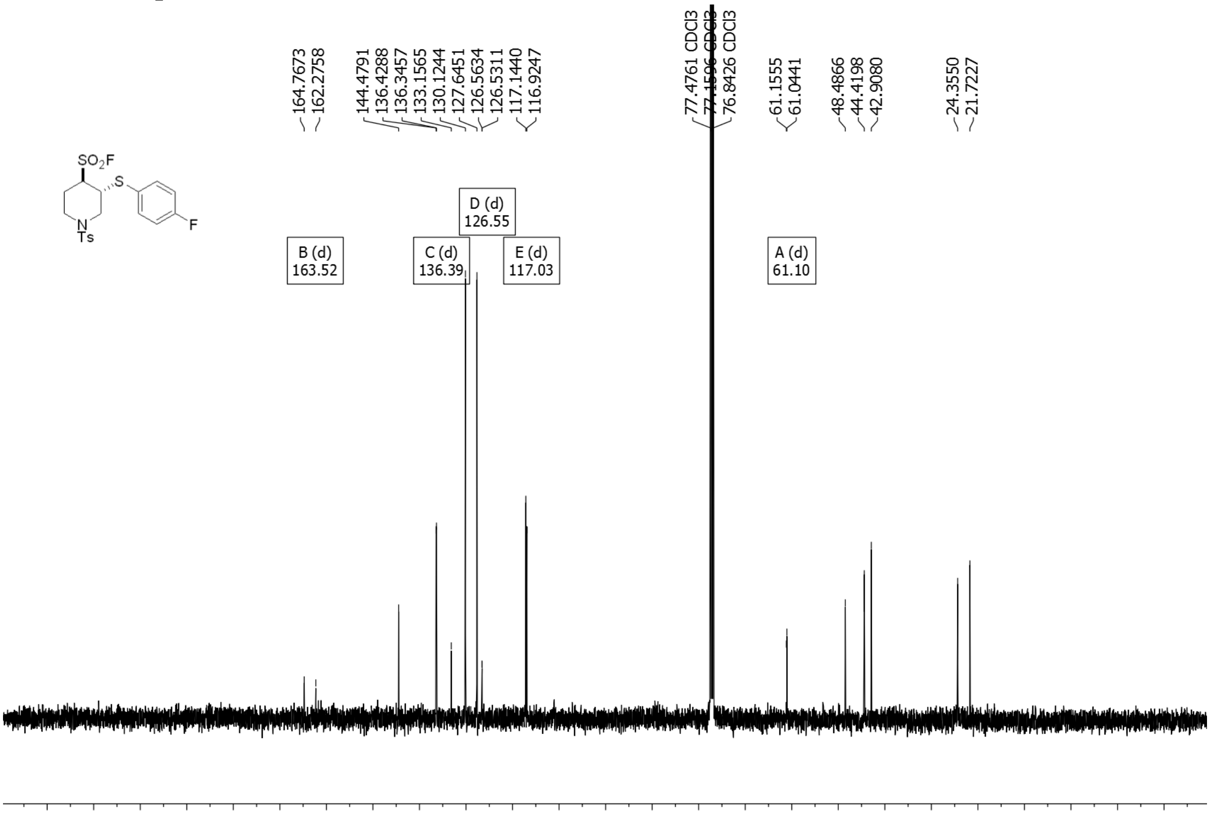

^{19}F-NMR Spectrum:

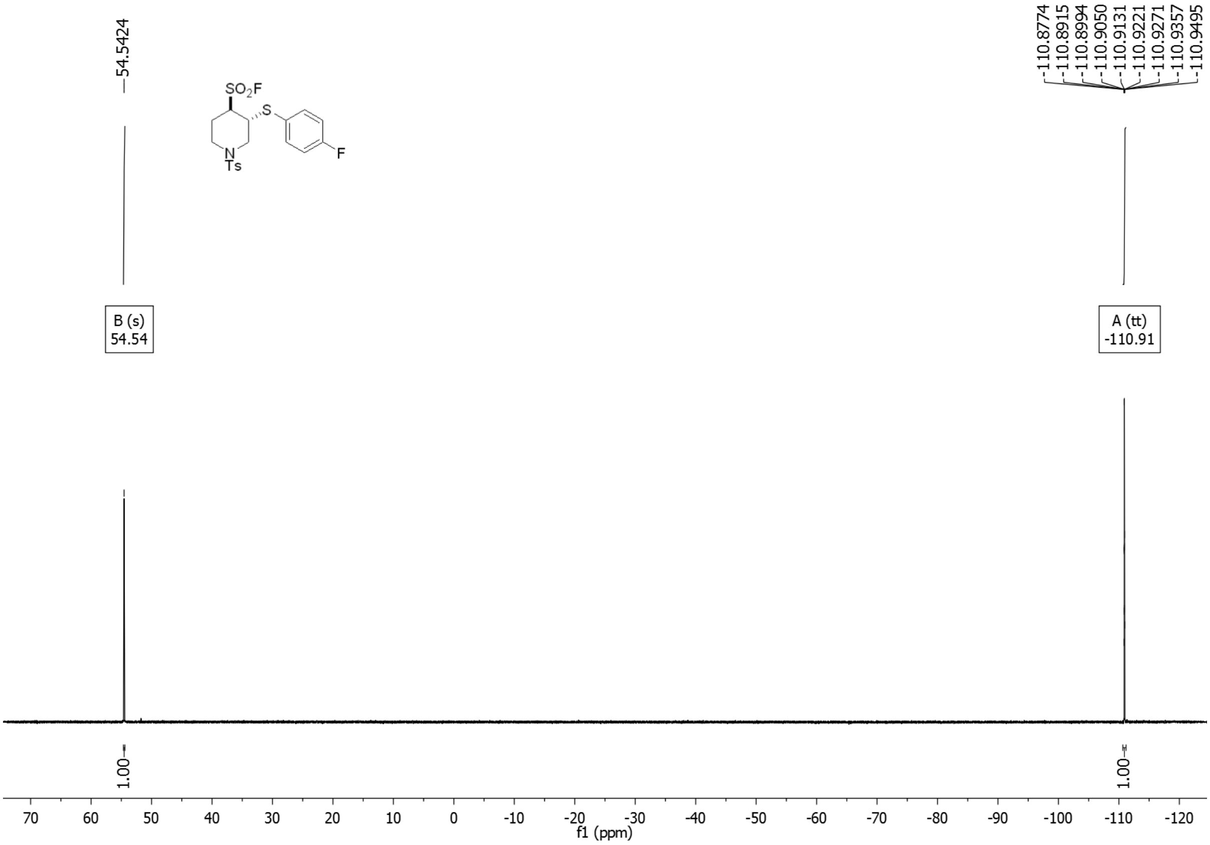

^{1}H-^{1}H COSY NMR Spectrum:

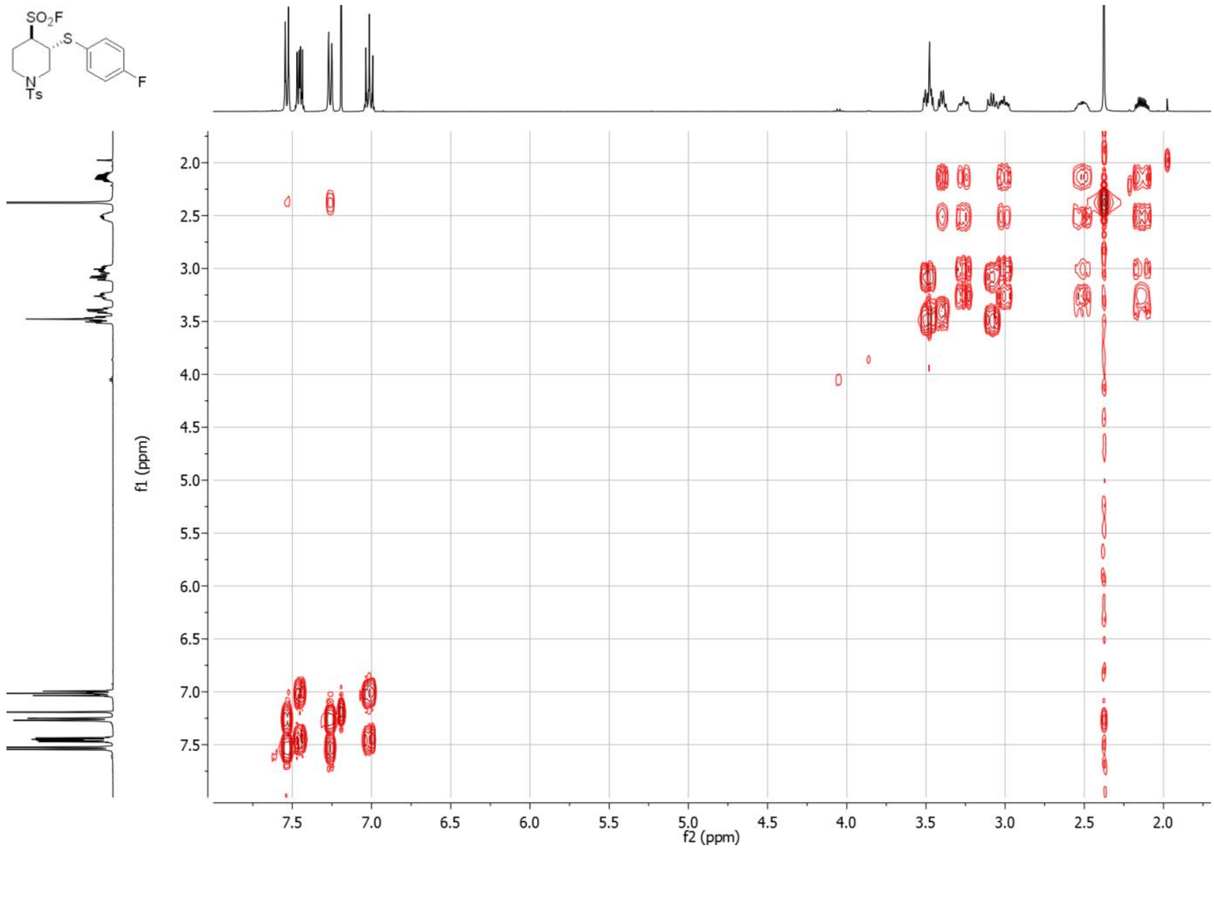

^{1}H-^{1}H NOESY NMR Spectrum:

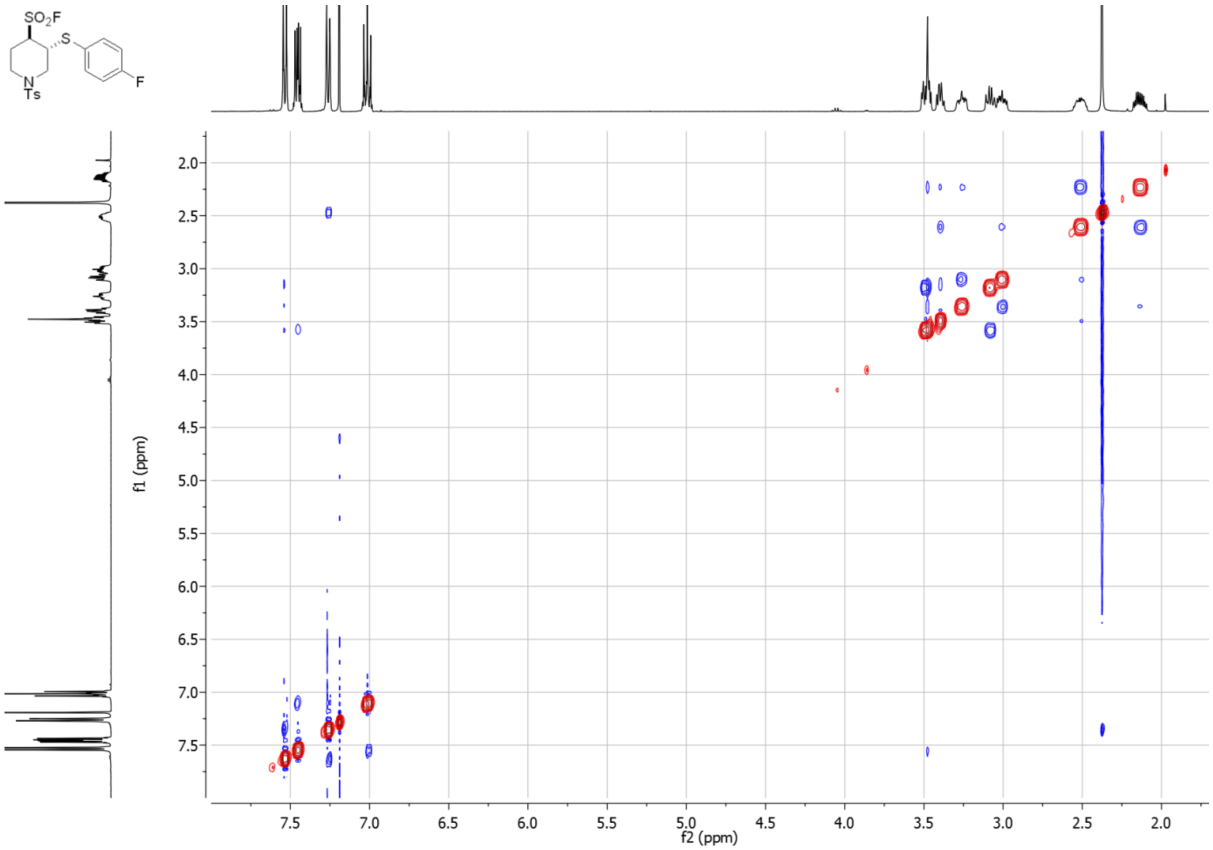

^{1}H-^{13}C HSQC NMR Spectrum:

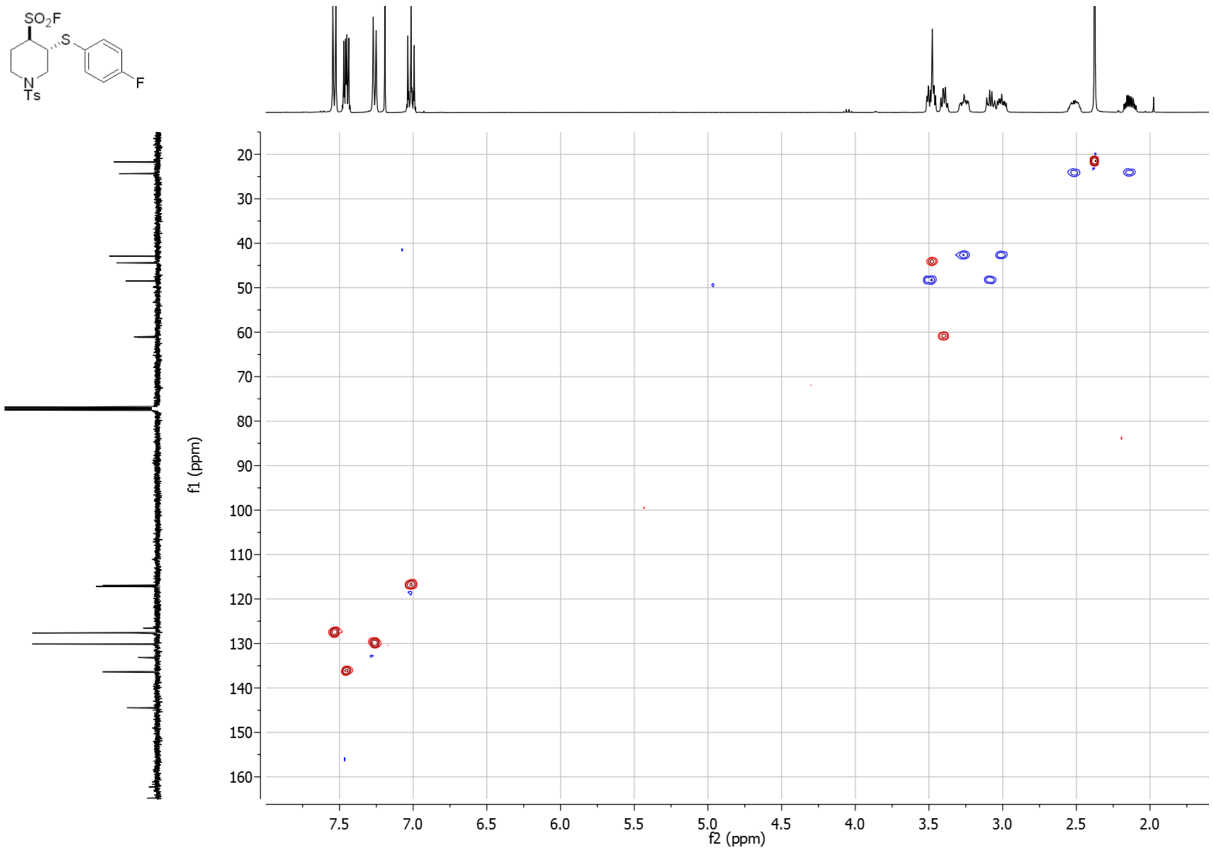

***cis*-3-((4-Fluorophenyl)thio)-1-tosylpiperidine-4-sulfonyl fluoride (9b2)**

^{1}H-NMR Spectrum:

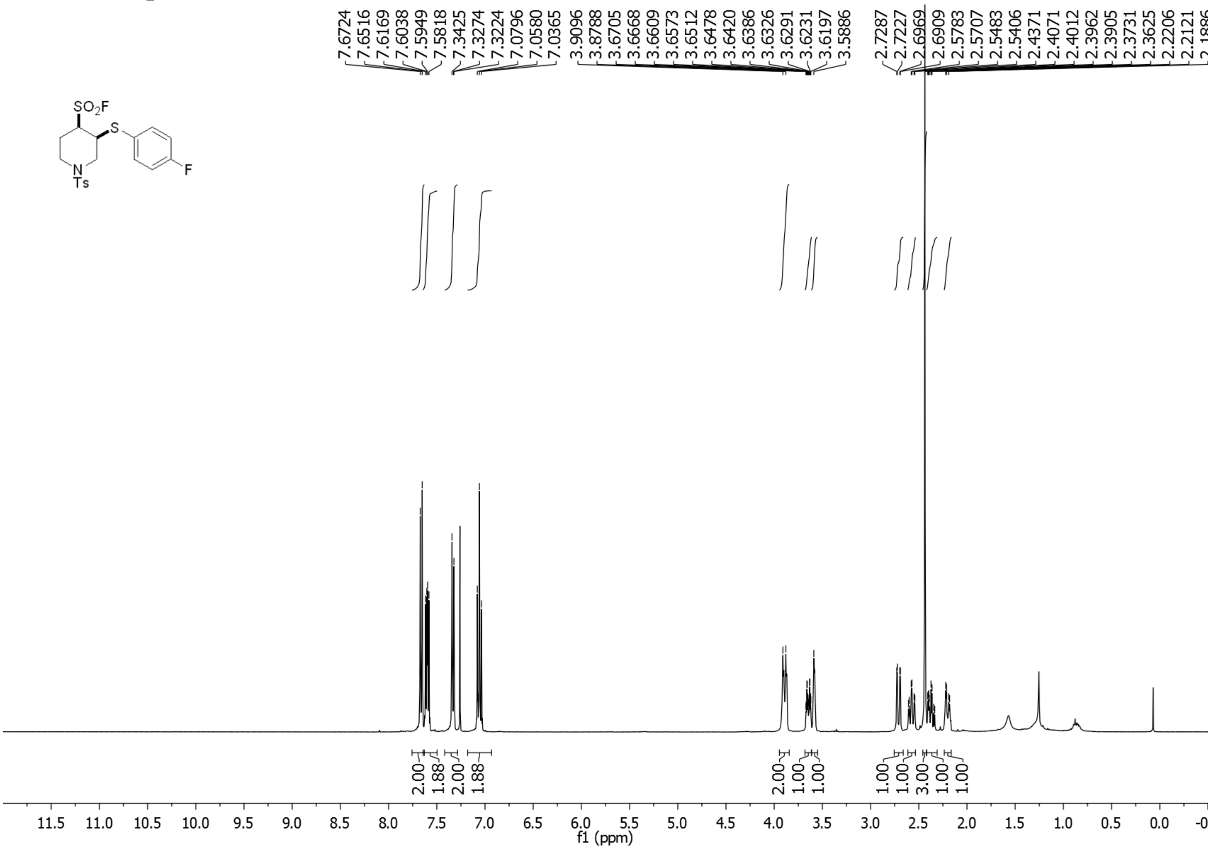

^{13}C-NMR Spectrum:

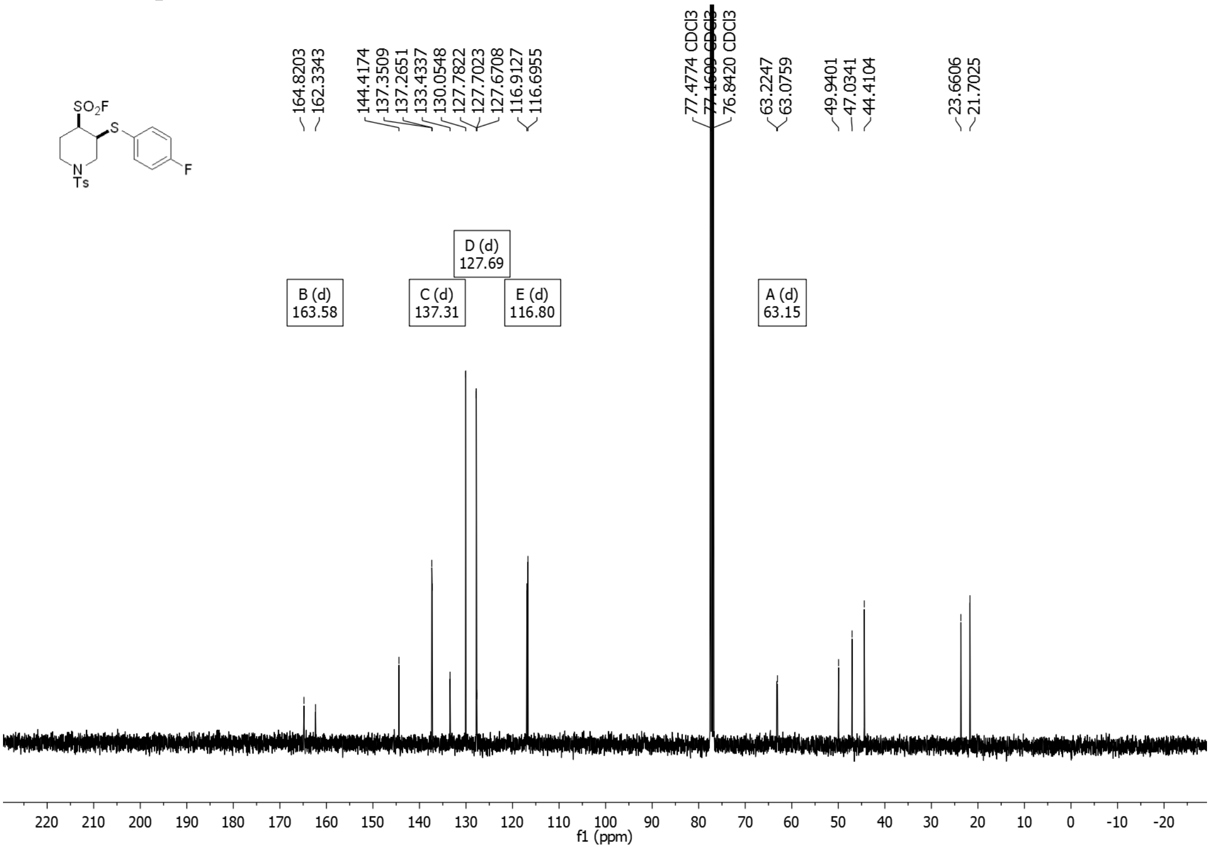

^{19}F-NMR Spectrum:

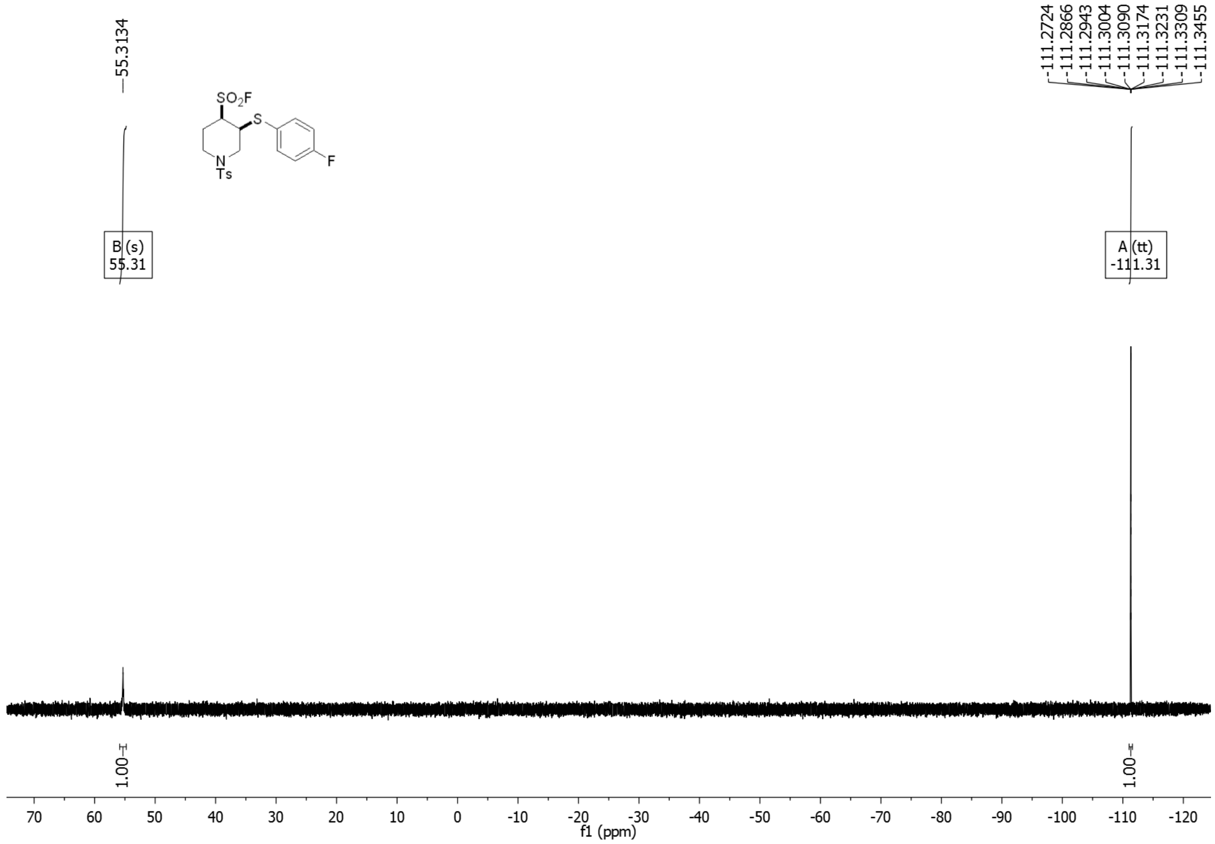

^{1}H-^{1}H COSY NMR Spectrum:

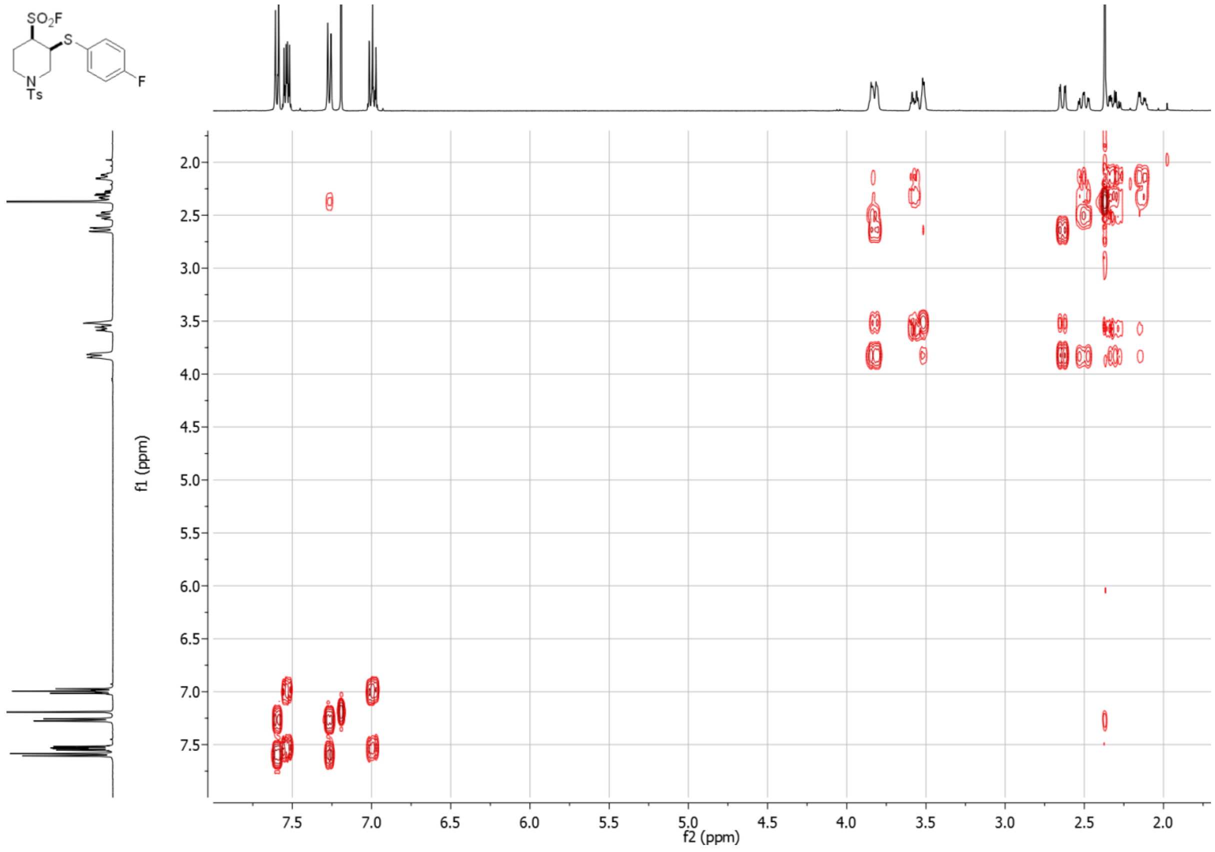

^{1}H-^{1}H NOESY NMR Spectrum:

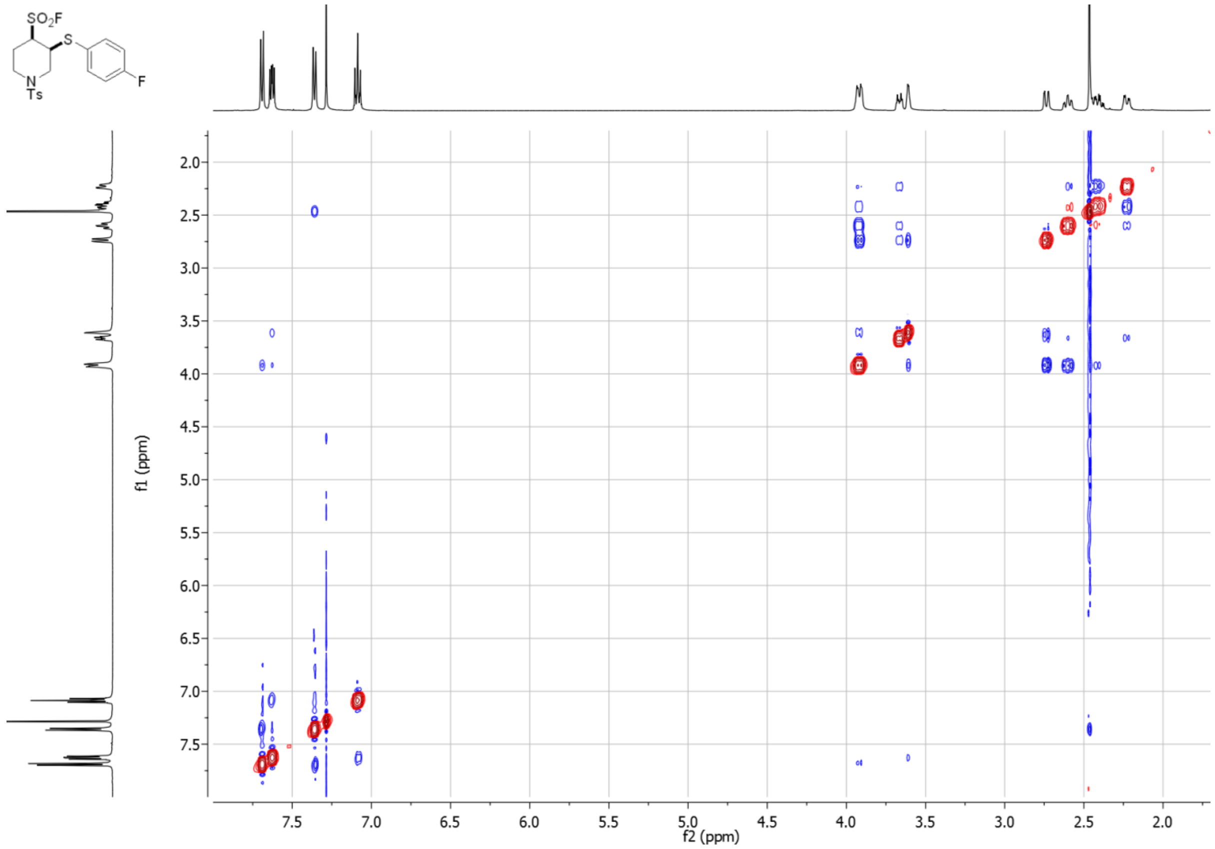

^{1}H-^{13}C HSQC NMR Spectrum:

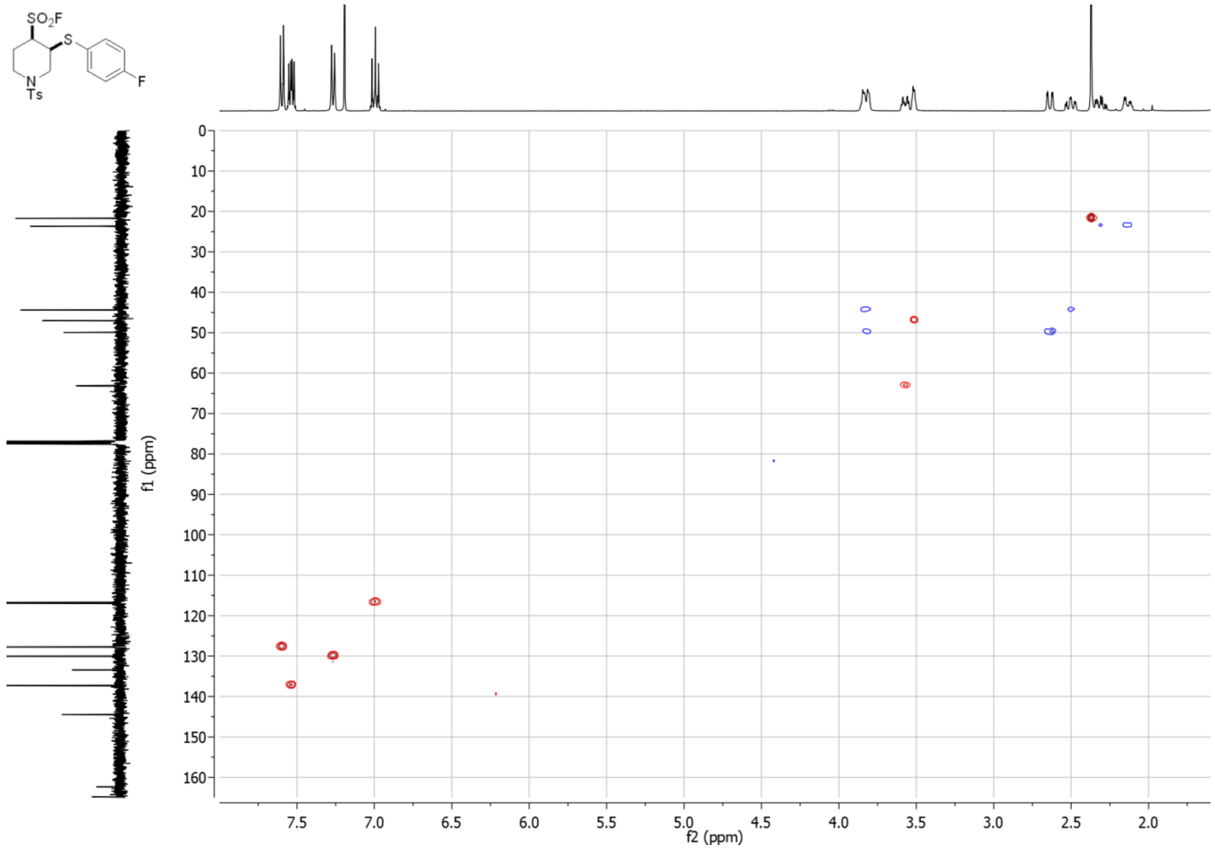

***trans*-3-((4-Methoxybenzyl)thio)-1-tosylpiperidine-4-sulfonyl fluoride (9c1)**

^{1}H-NMR Spectrum:

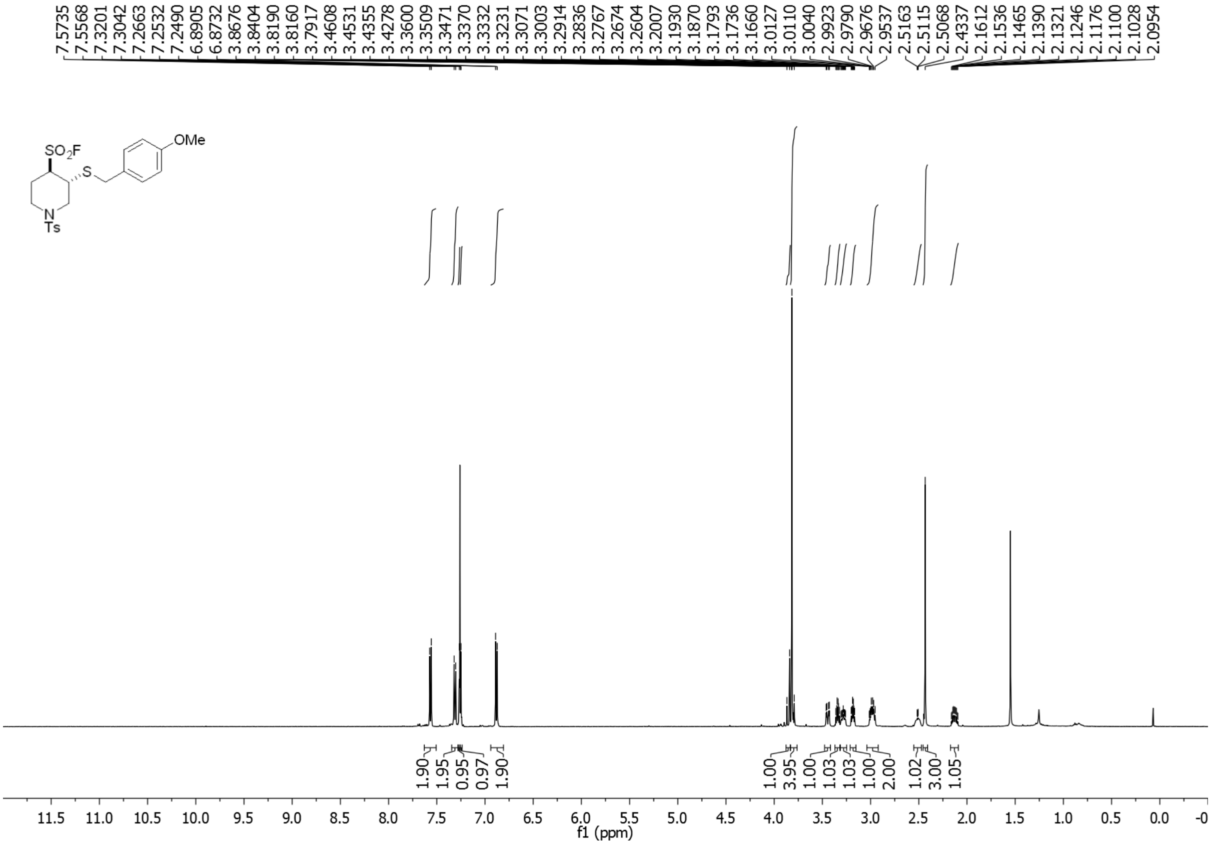

^{13}C-NMR Spectrum:

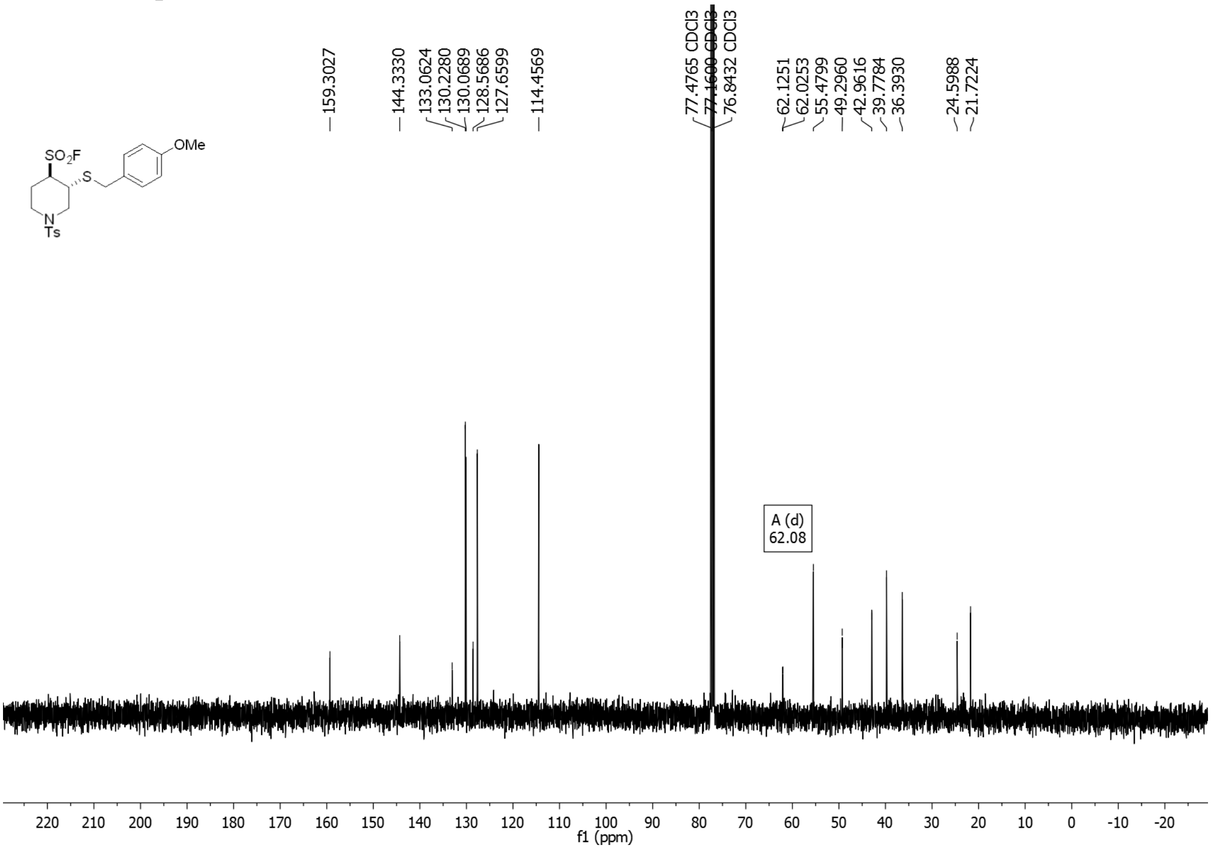

^{19}F-NMR Spectrum:

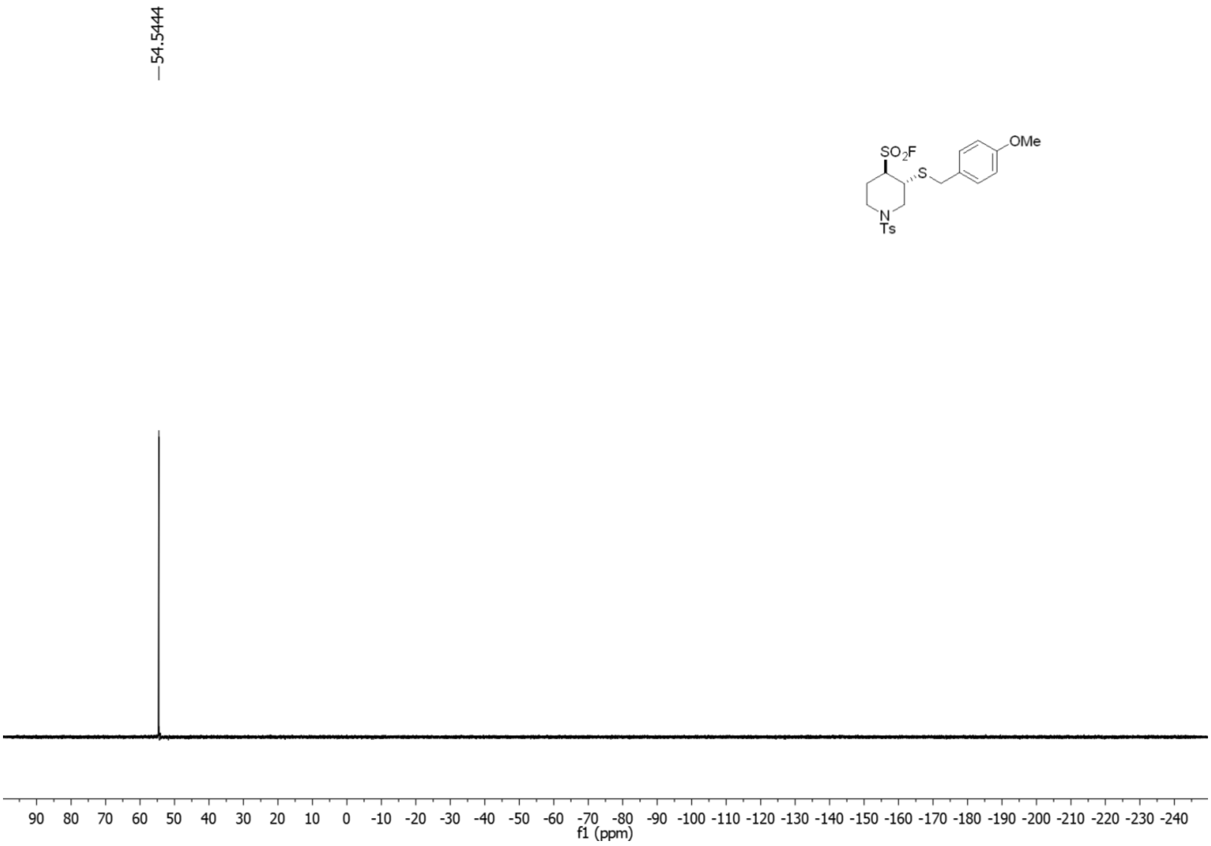

^{1}H-^{1}H COSY NMR Spectrum:

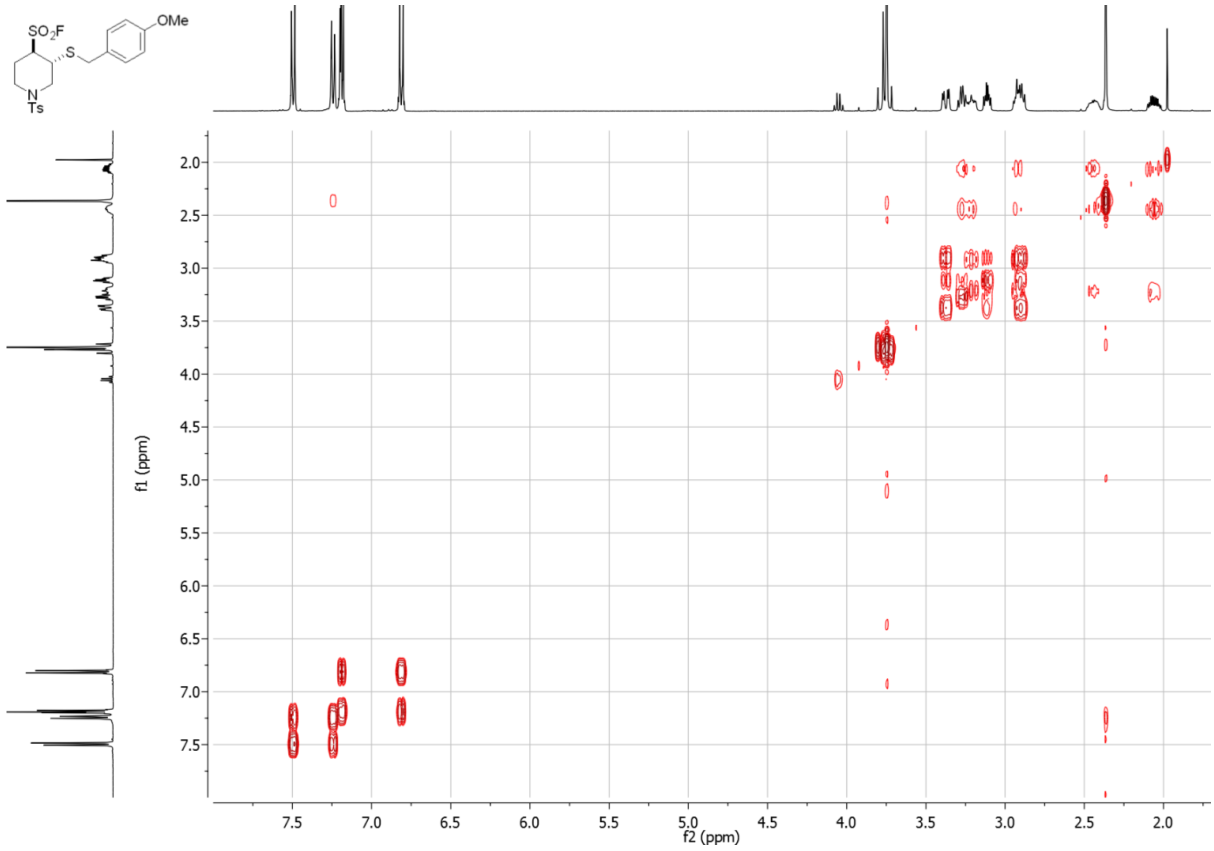

$^1H-^1H$ NOESY NMR Spectrum:

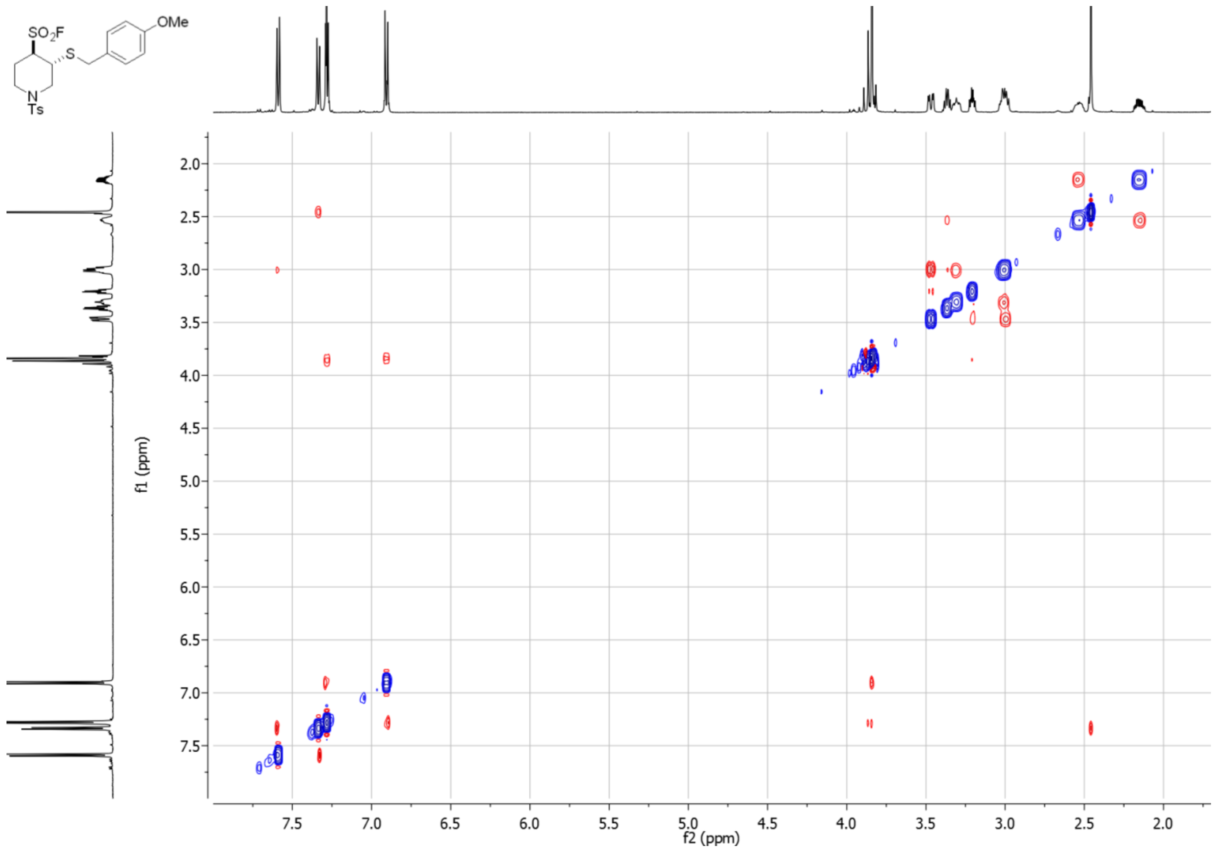

$^1H-^{13}C$ HSQC NMR Spectrum:

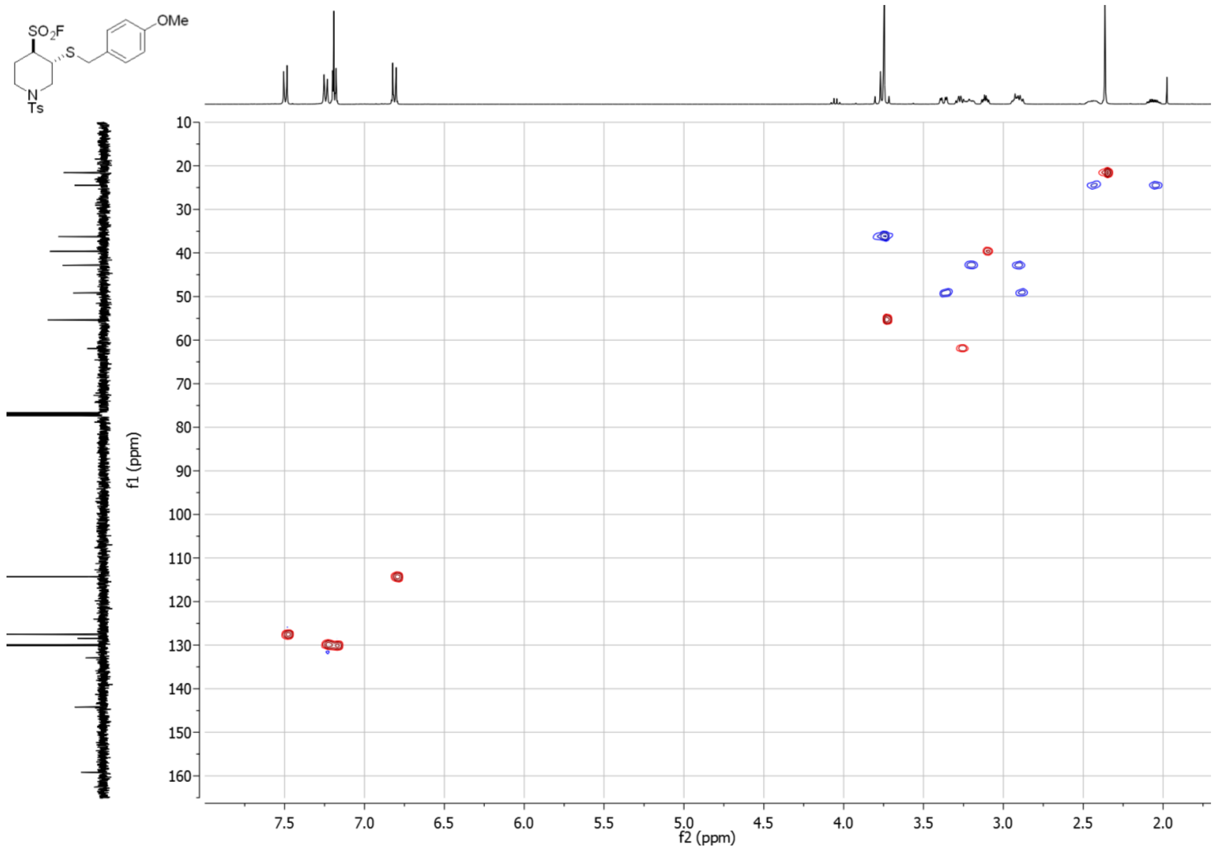

***cis*-3-((4-Methoxybenzyl)thio)-1-tosylpiperidine-4-sulfonyl fluoride (9c2)**

^{1}H-NMR Spectrum:

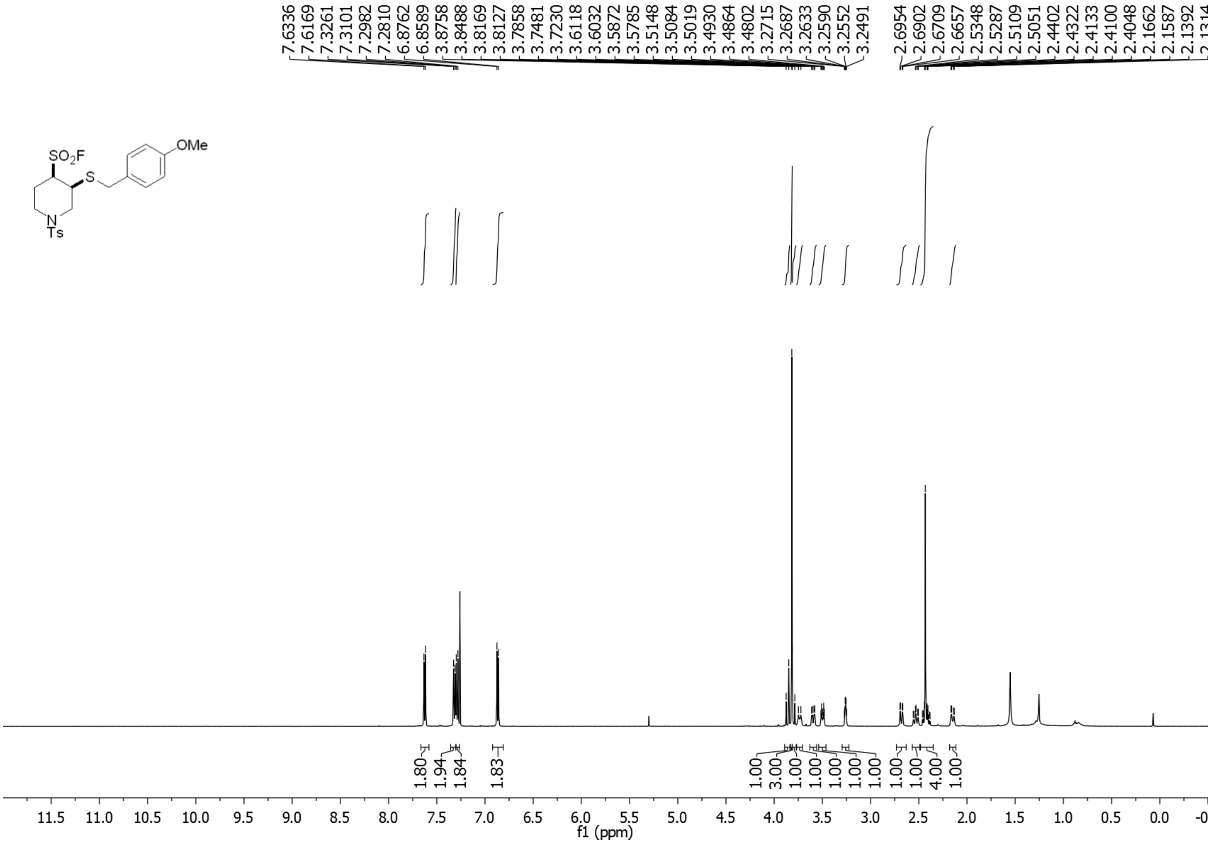

^{13}C-NMR Spectrum:

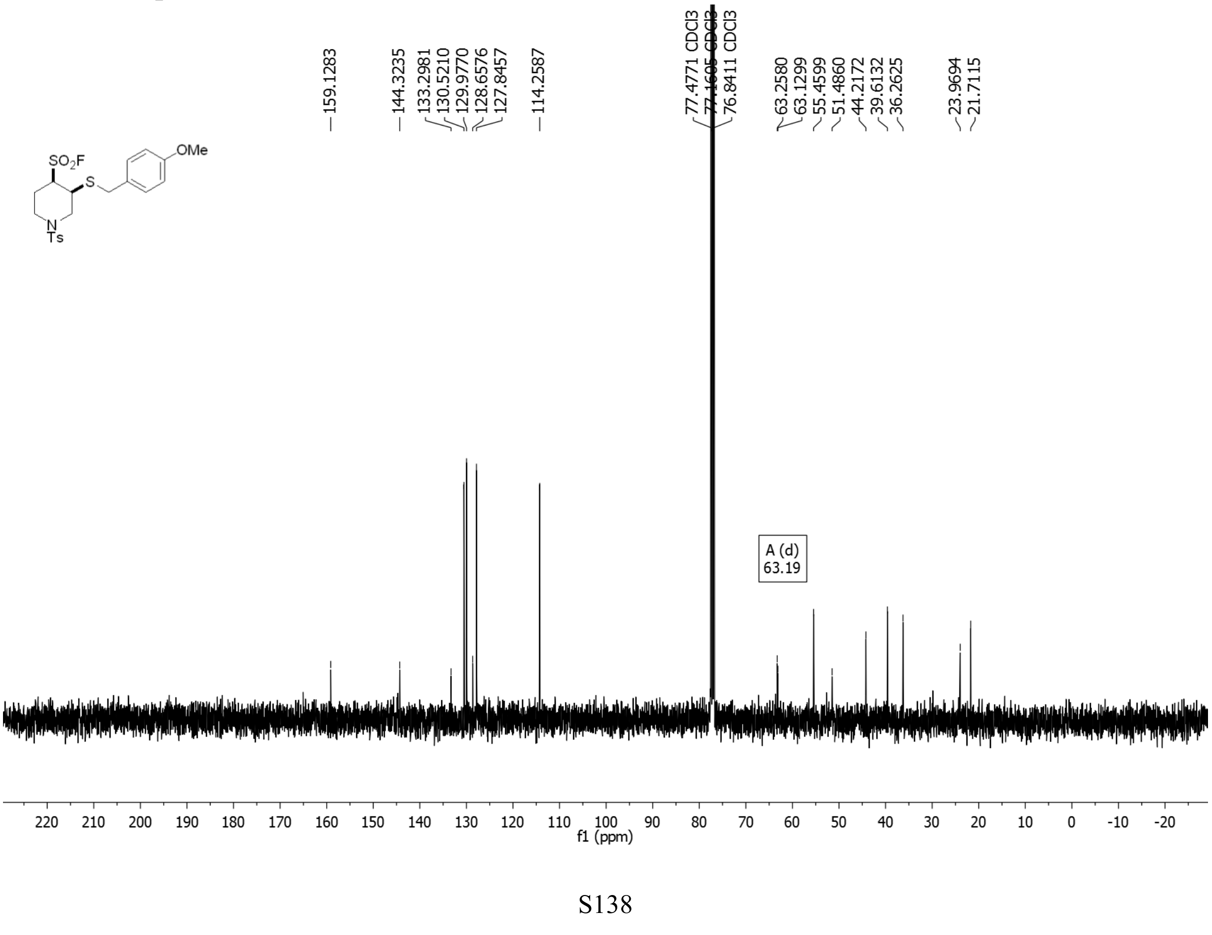

^{19}F-NMR Spectrum:

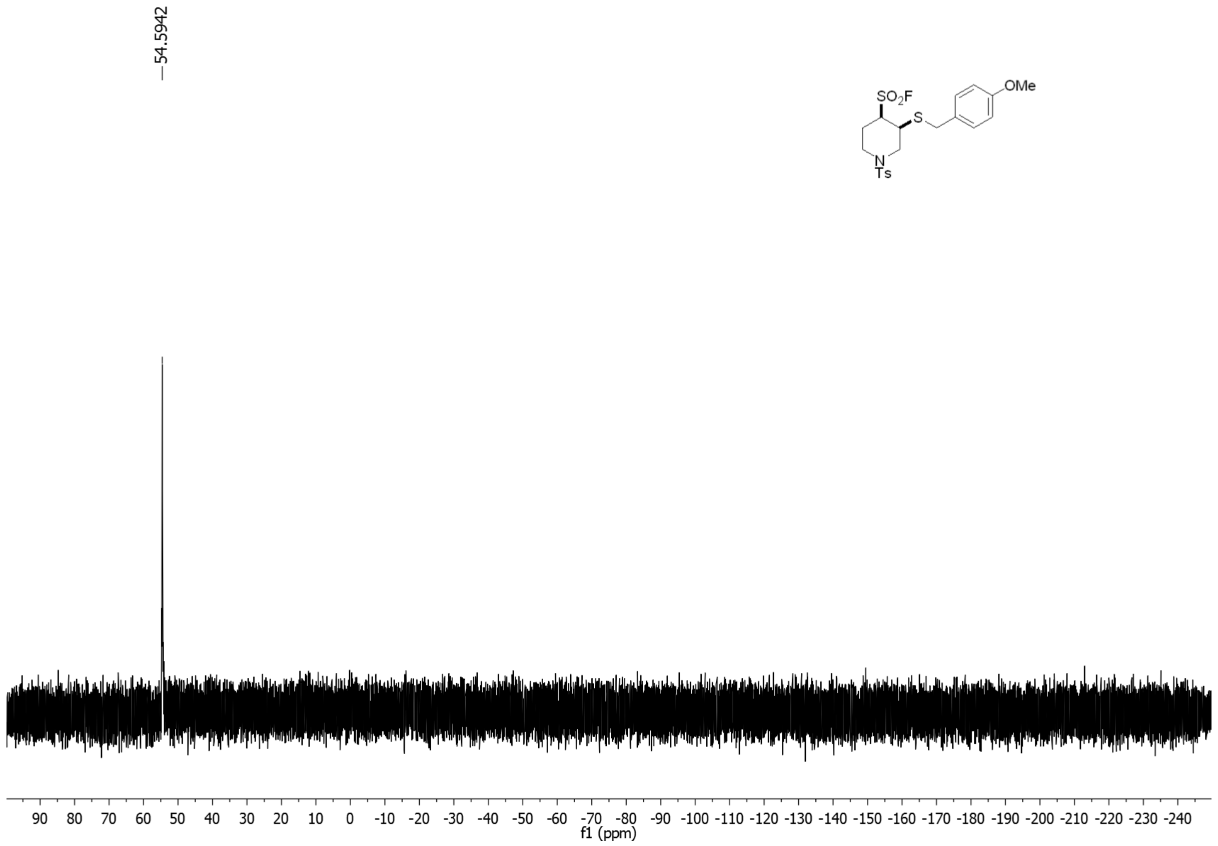

^{1}H-^{1}H COSY NMR Spectrum:

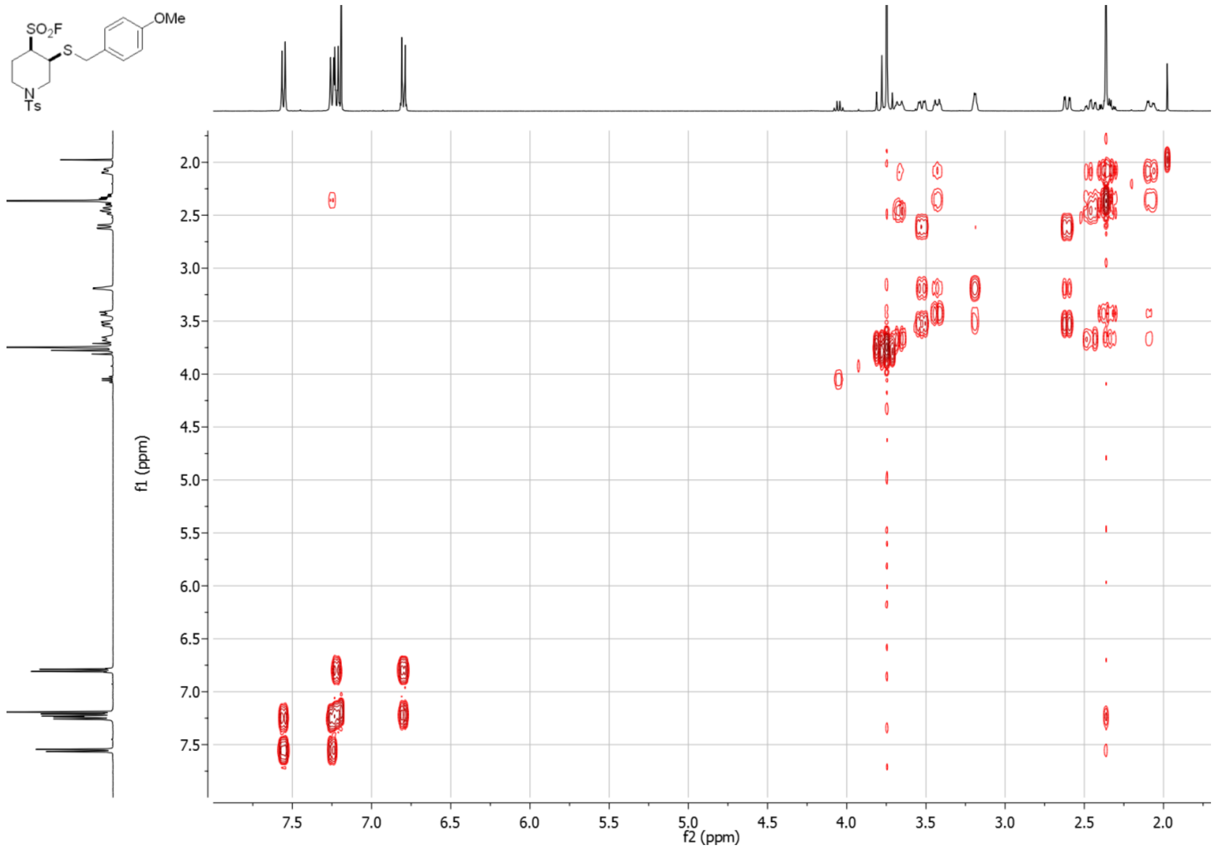

^1H-^1H NOESY NMR Spectrum:

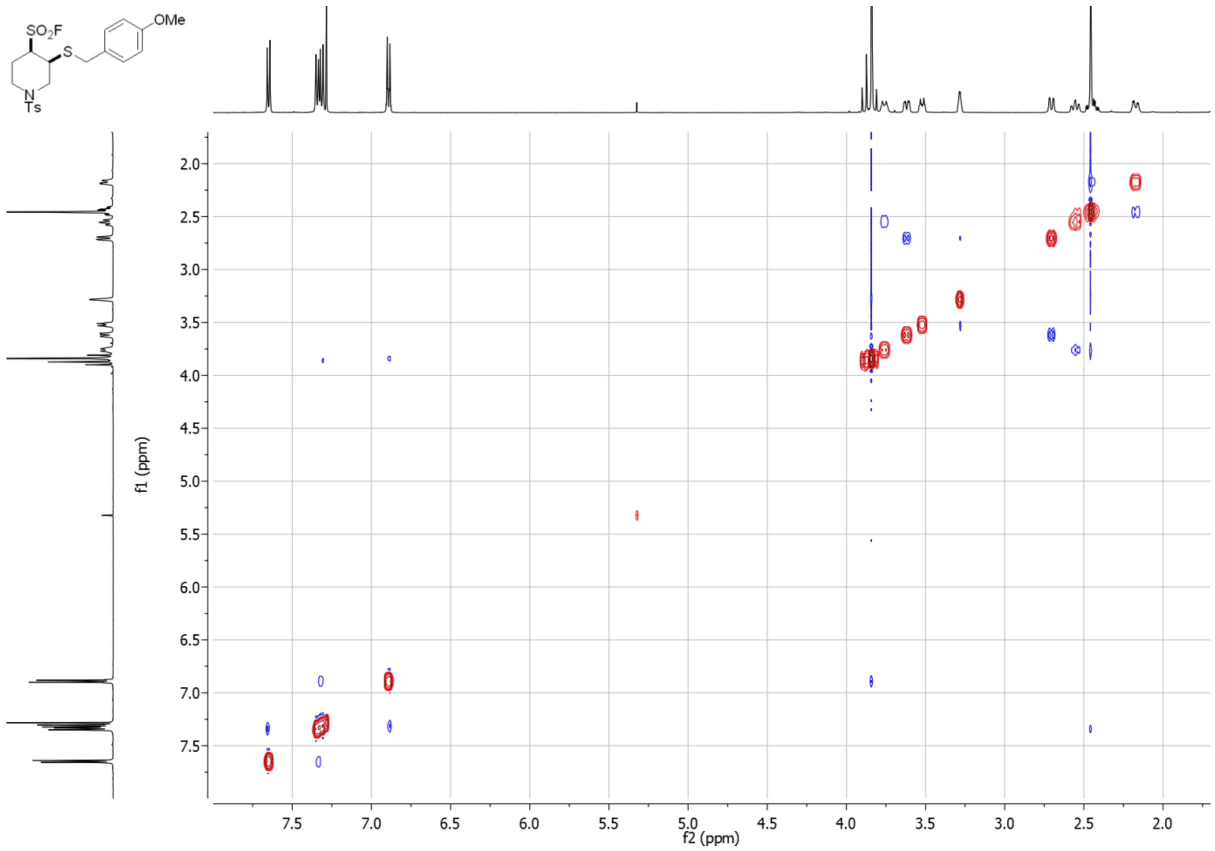

^1H-^{13}C HSQC NMR Spectrum:

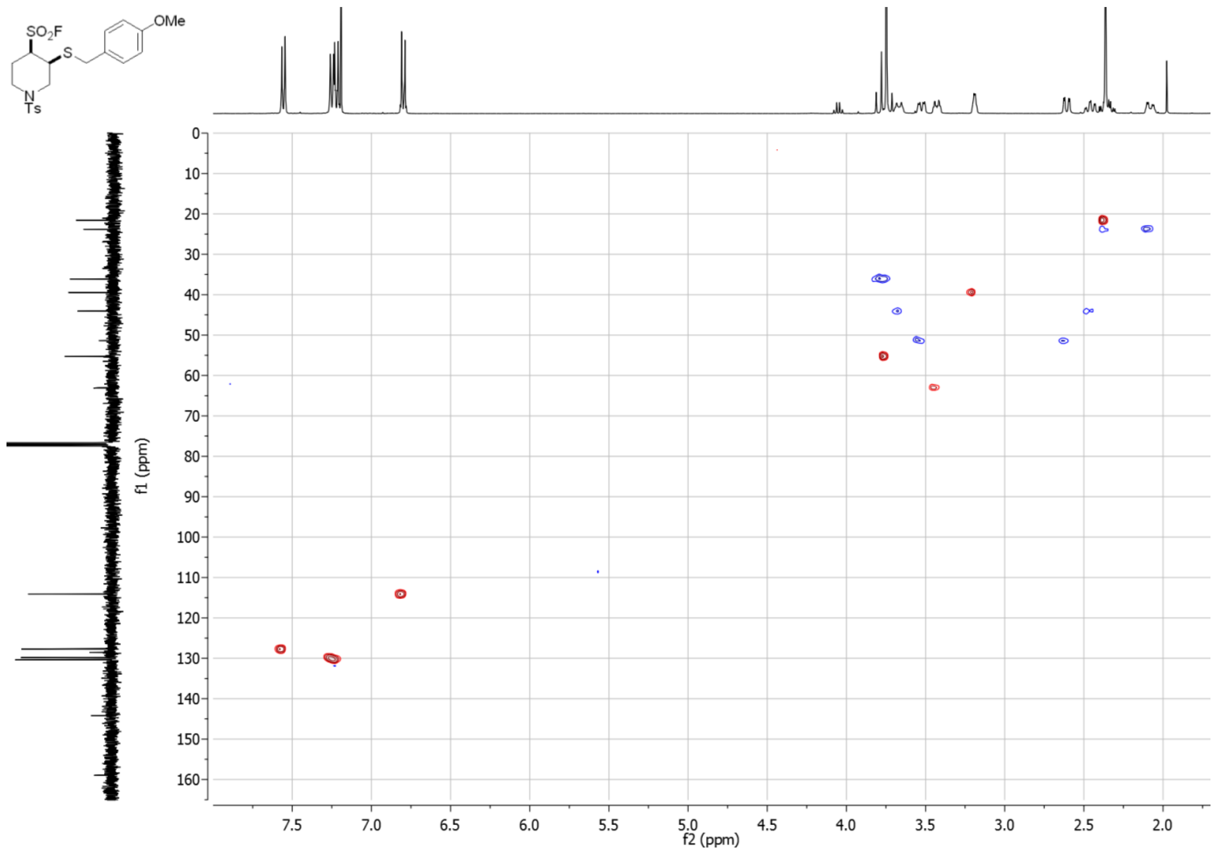

1,4-Dioxaspiro[4.5]decane-8-sulfonyl fluoride (10)

^{1}H-NMR Spectrum:

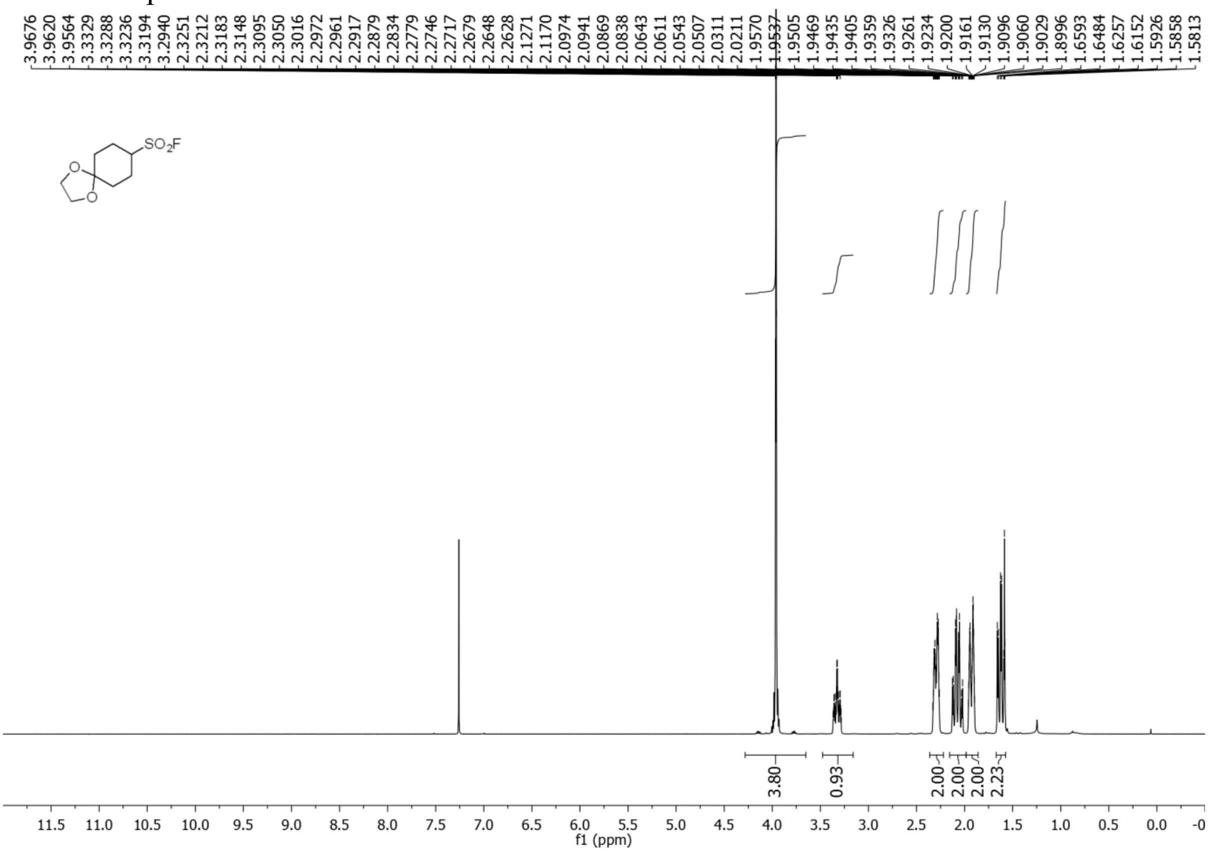

^{13}C-NMR Spectrum:

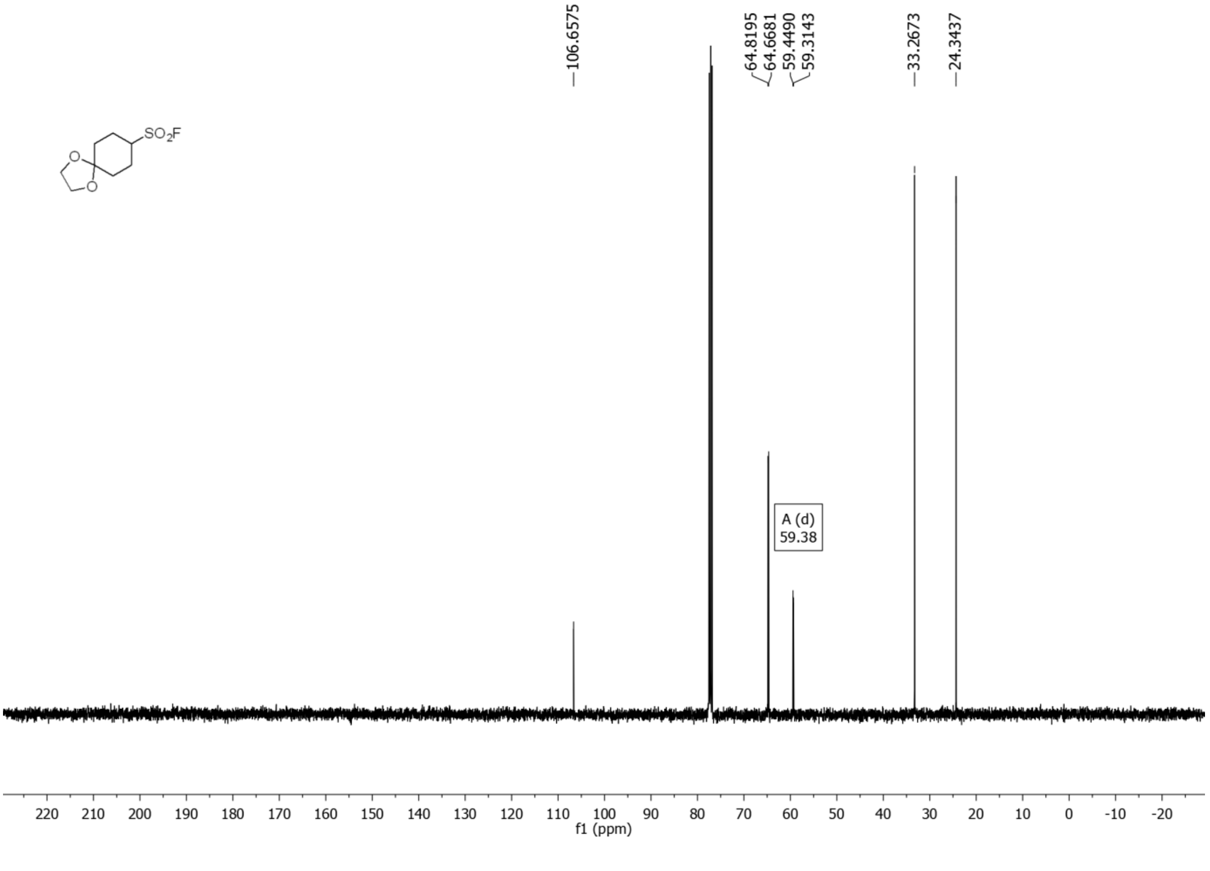

^{19}F-NMR Spectrum:

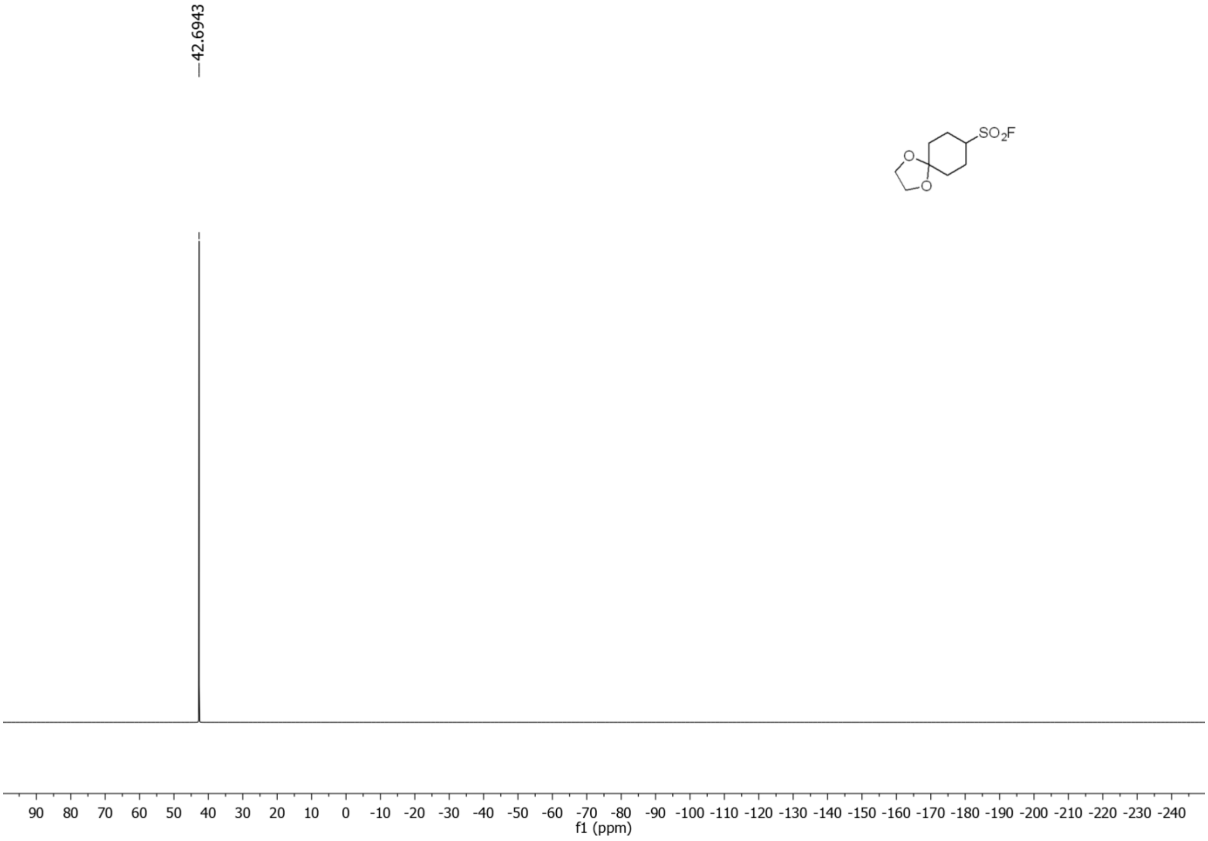

Prop-2-yn-1-yl 4-(fluorosulfonyl)-3,6-dihydropyridine-1(2*H*)-carboxylate (11)

^{1}H-NMR Spectrum:

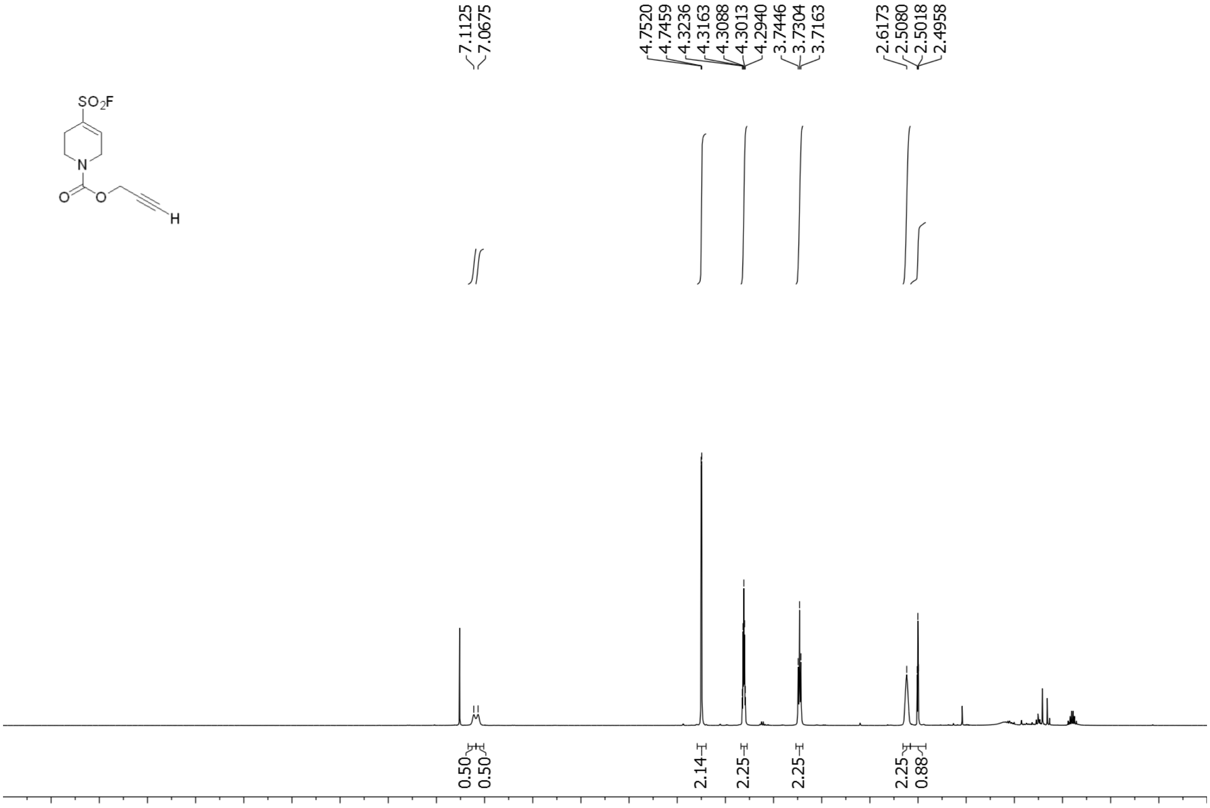

^{13}C-NMR Spectrum:

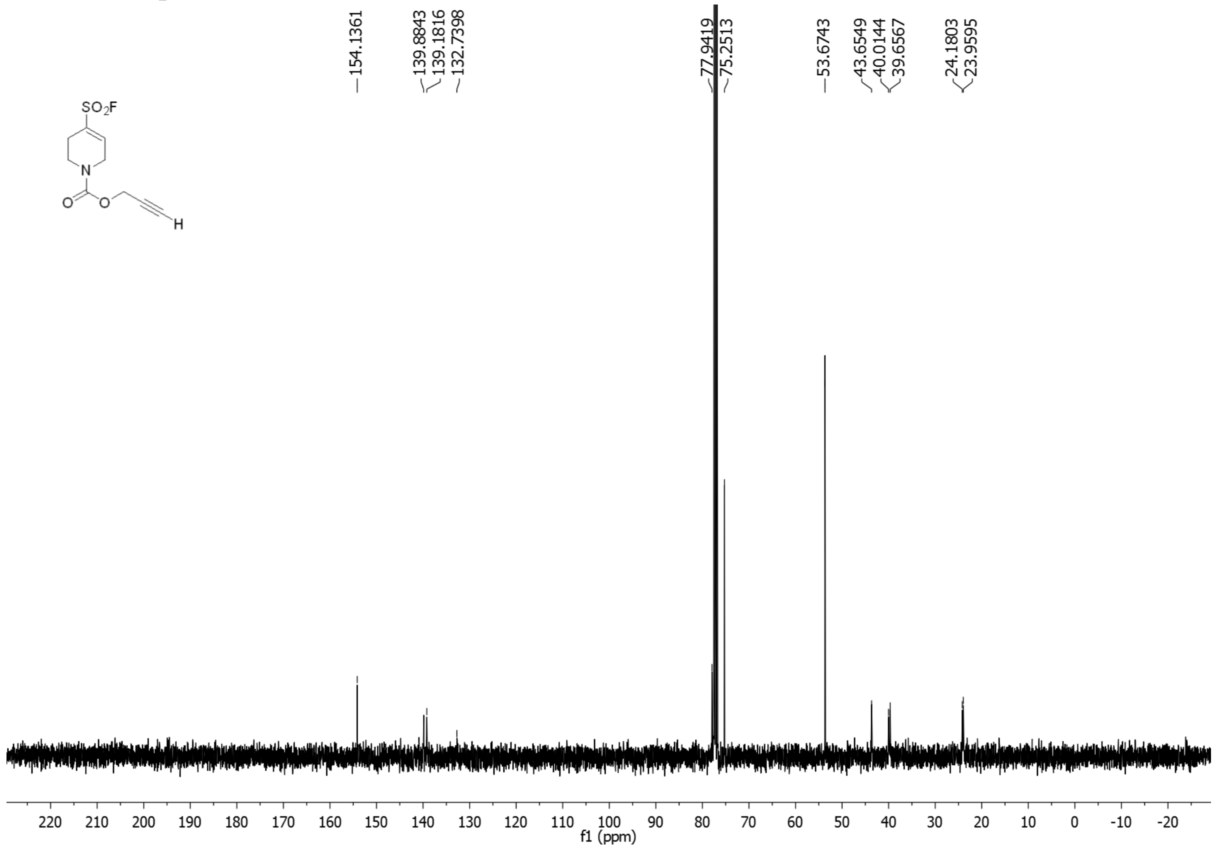

^{19}F-NMR Spectrum:

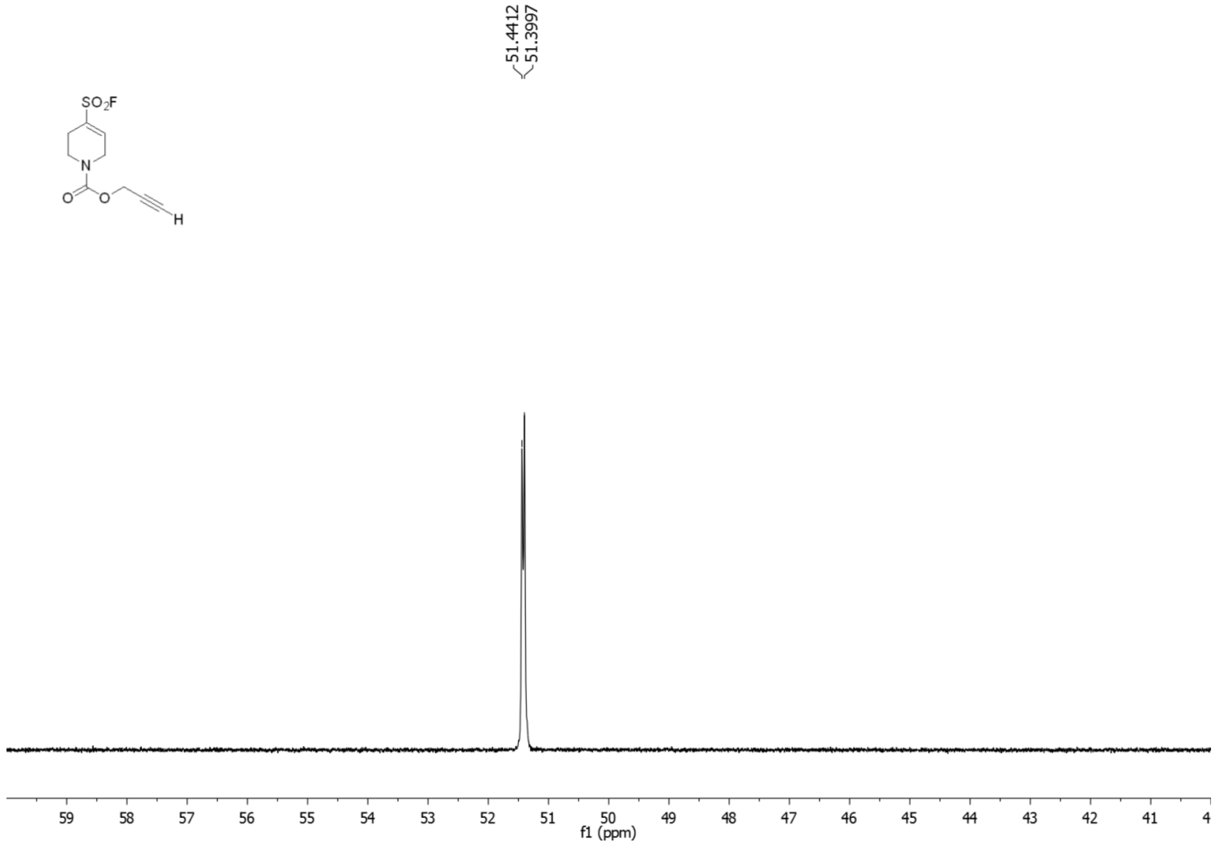

(1-Benzyl-1*H*-1,2,3-triazol-4-yl)methyl 4-(fluorosulfonyl)-3,6-dihydro-pyridine-1(2*H*)-carboxylate (12)

^{1}H-NMR Spectrum:

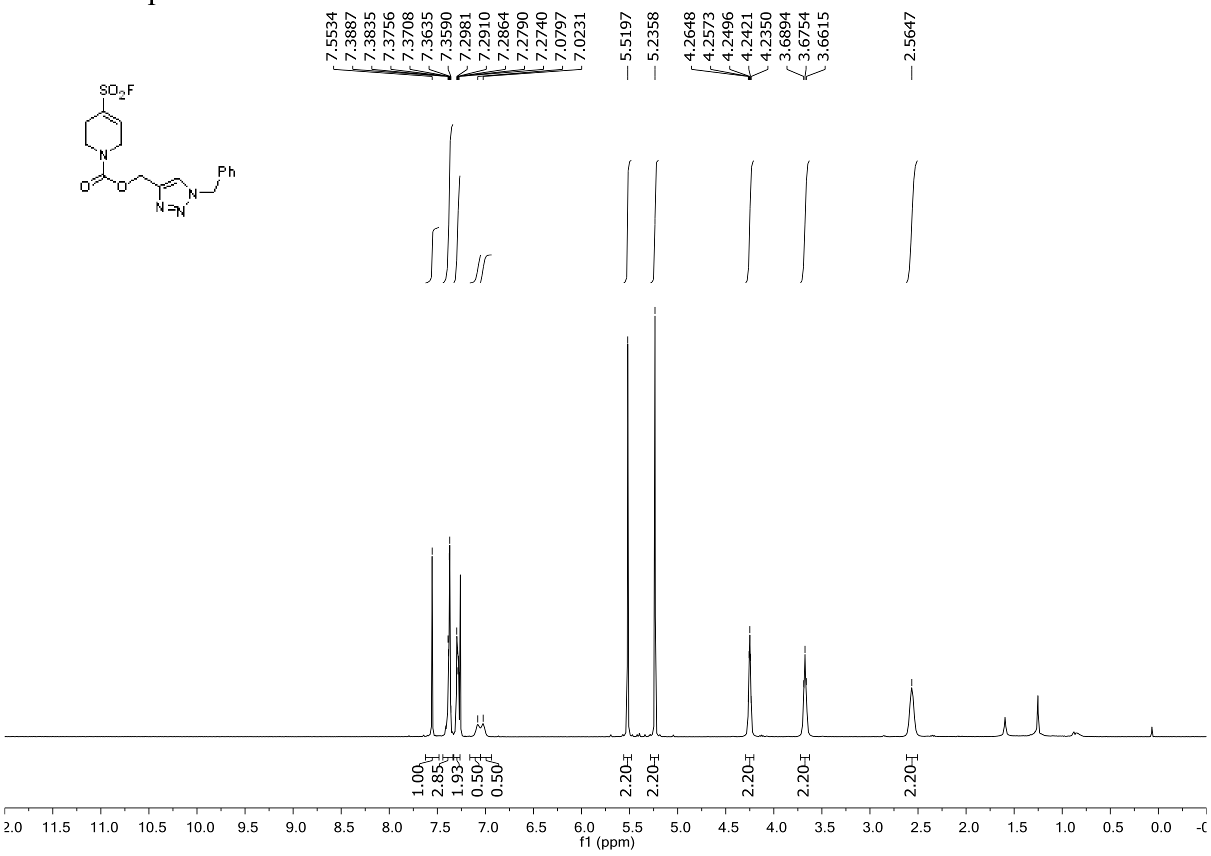

^{13}C-NMR Spectrum:

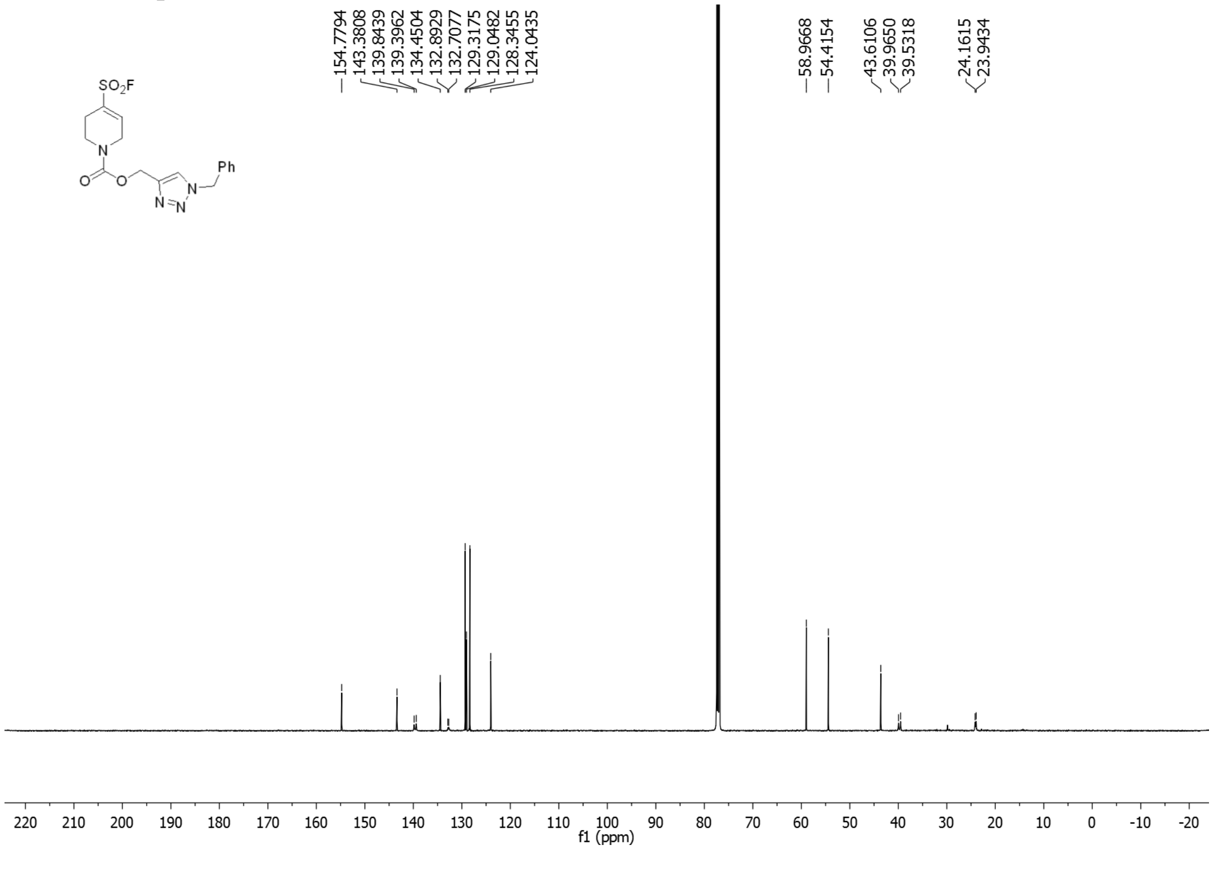

^{19}F-NMR Spectrum:

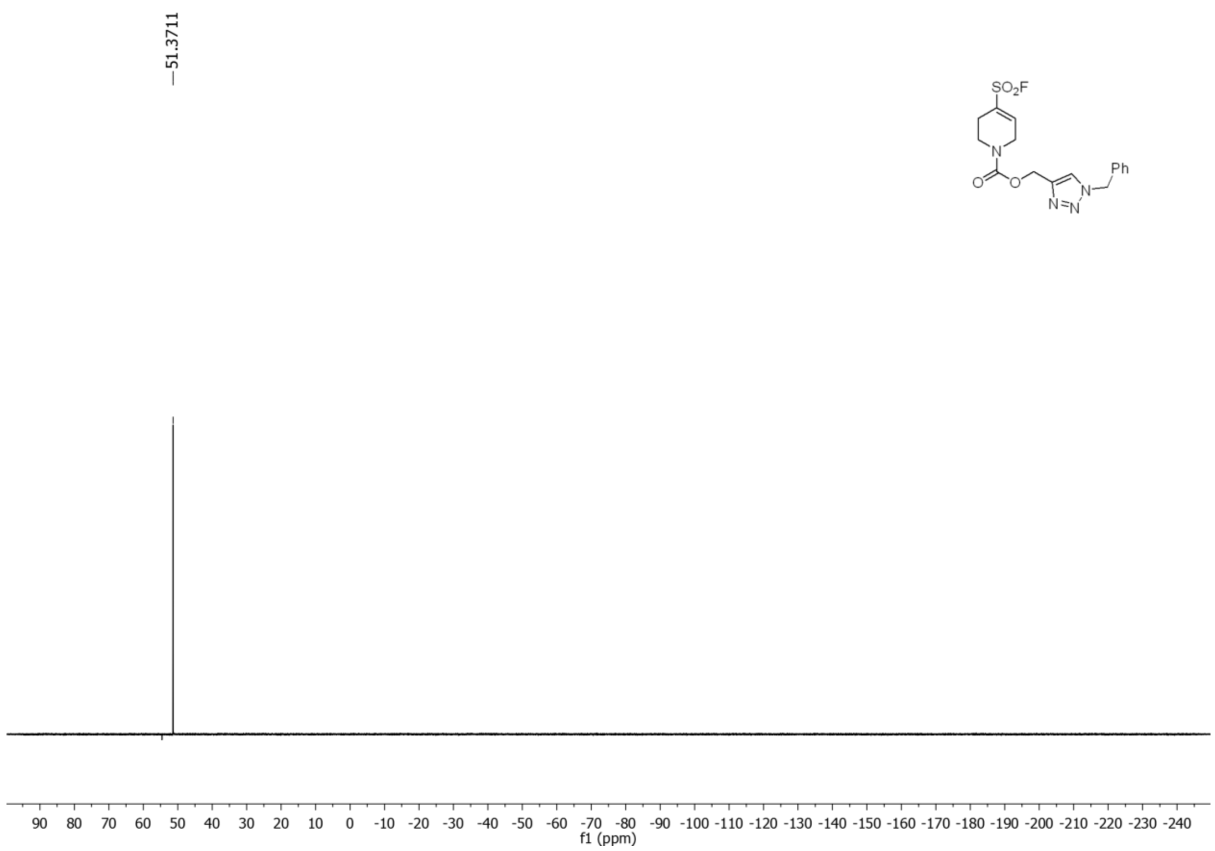

1,2,3,6-Tetrahydropyridine-4-sulfonyl fluoride hydrochloride (13)

^{1}H-NMR Spectrum:

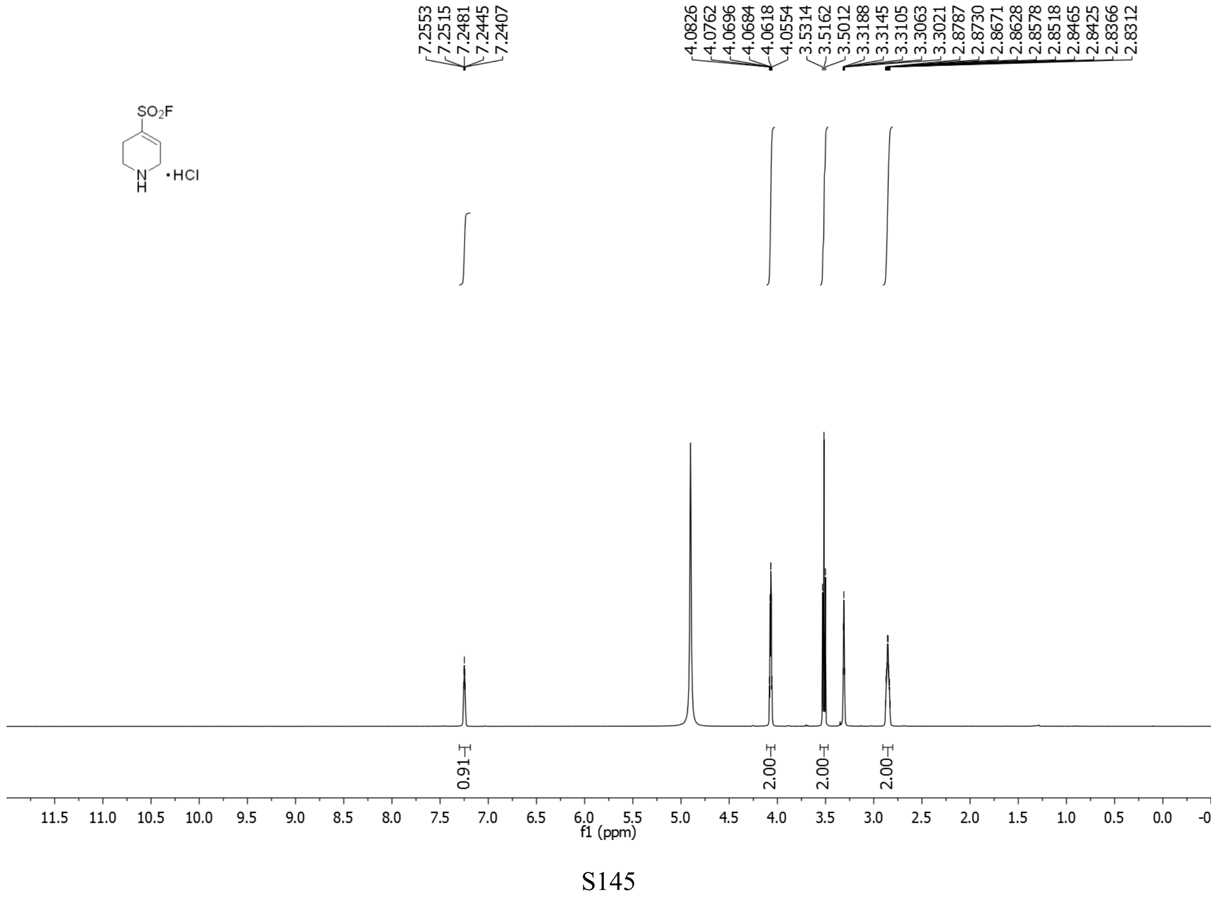

^{13}C-NMR Spectrum:

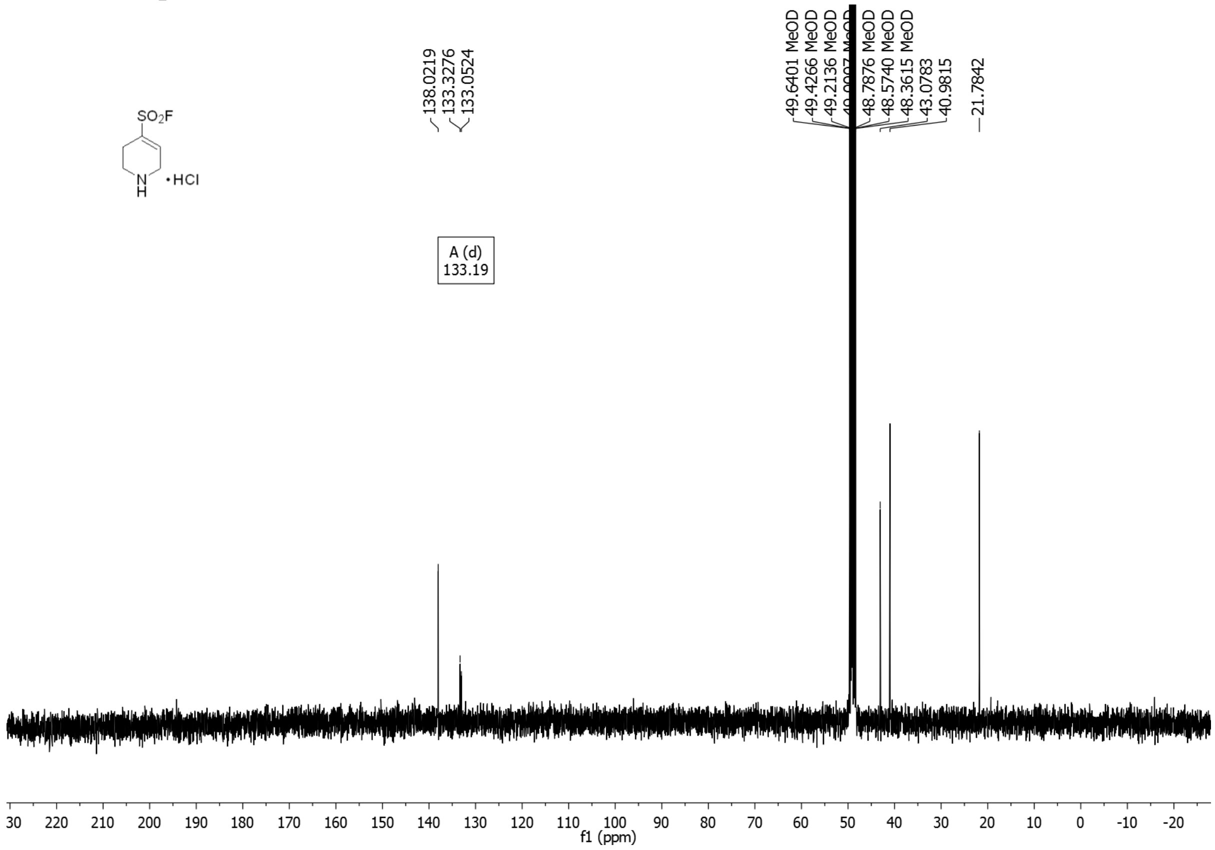

^{19}F-NMR Spectrum:

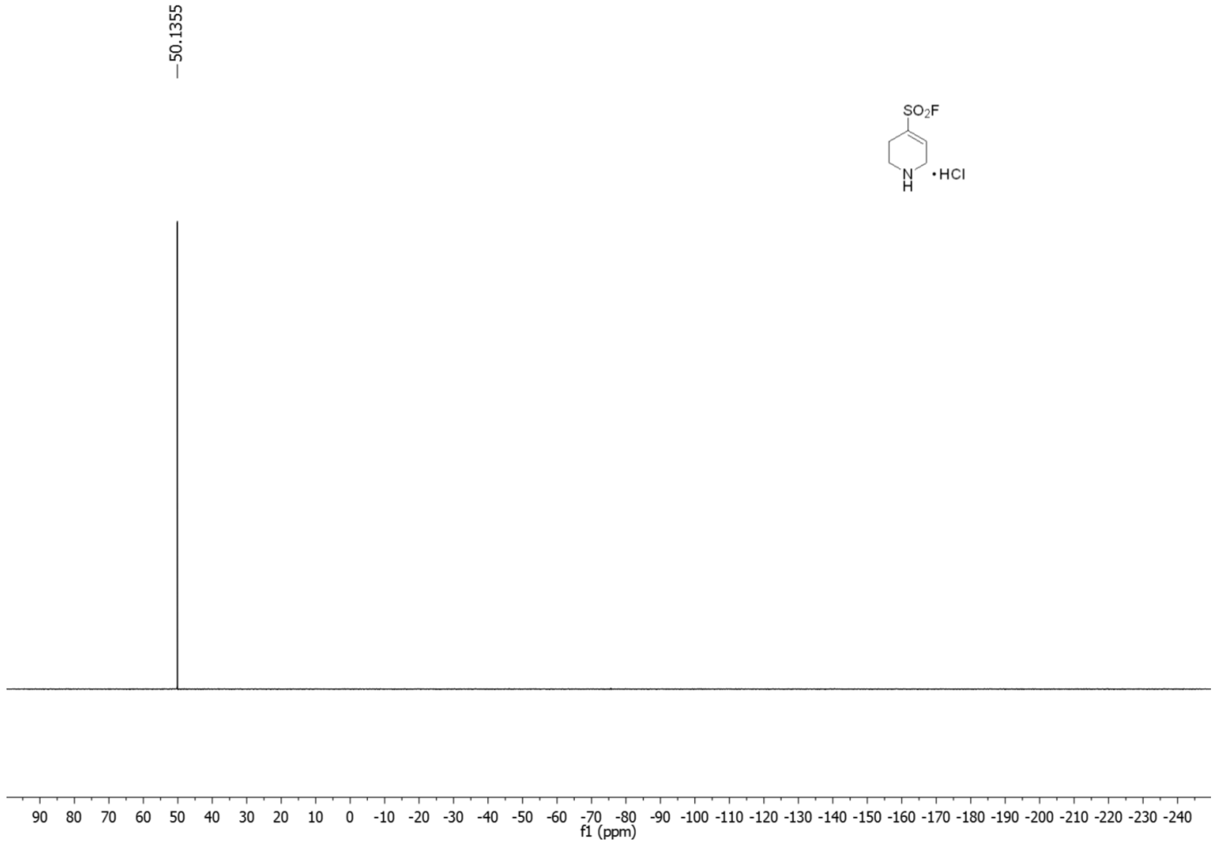

1-(Pyridin-3-ylmethyl)-1,2,3,6-tetrahydropyridine-4-sulfonyl fluoride (15)

^{1}H-NMR Spectrum:

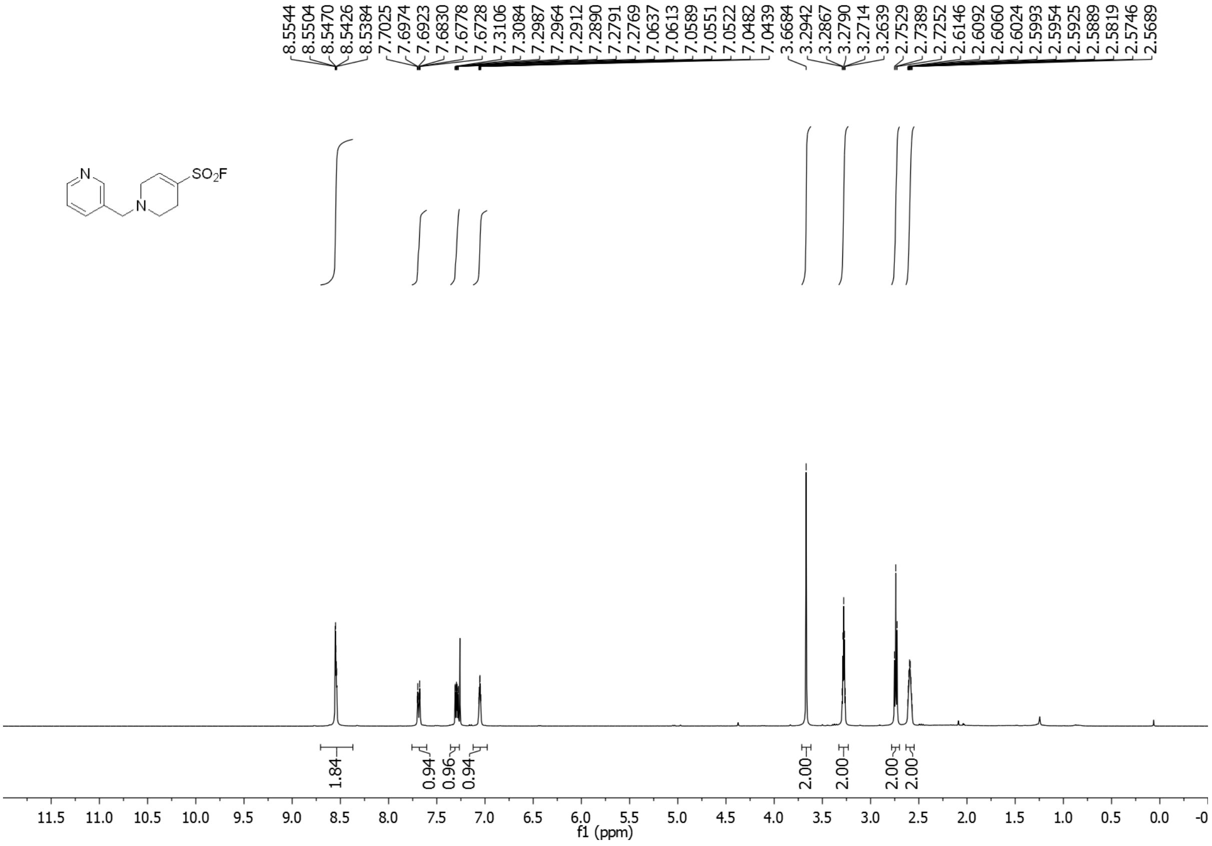

^{13}C-NMR Spectrum:

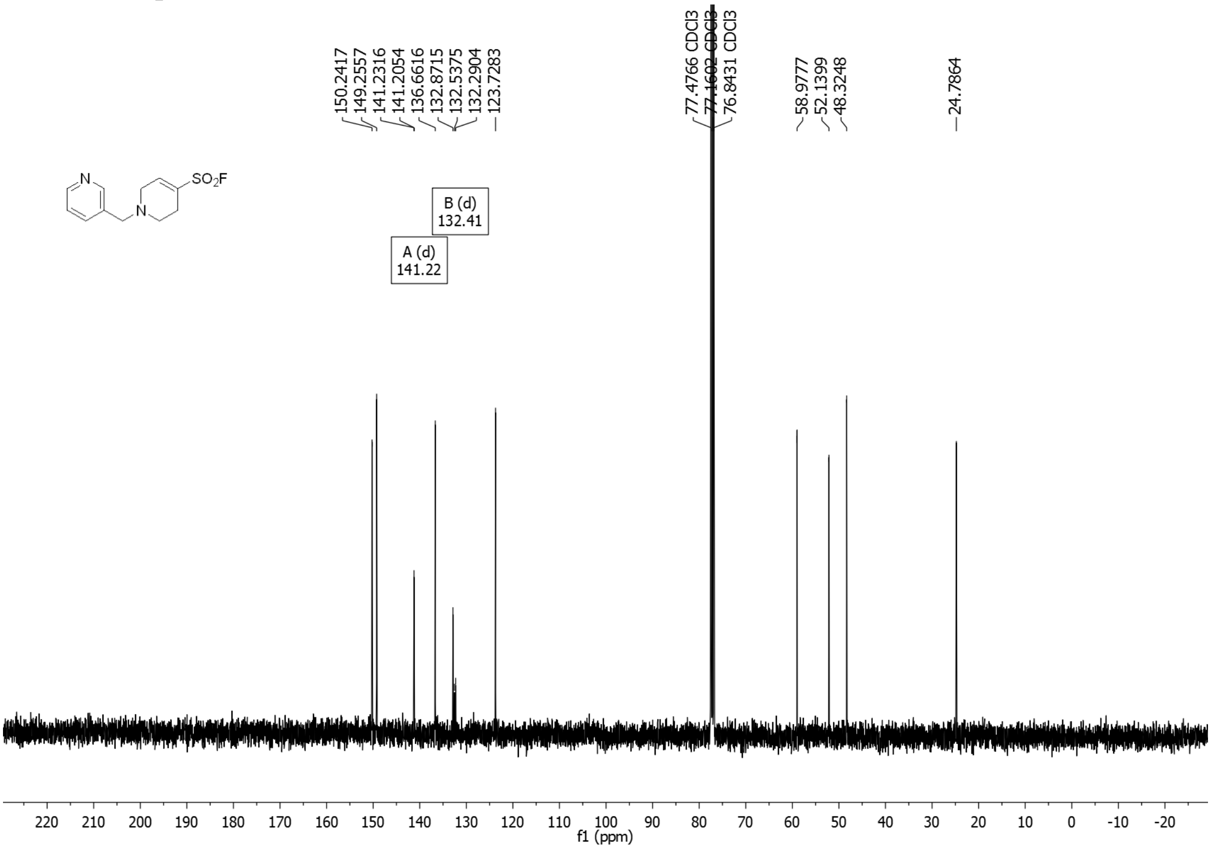

^{19}F-NMR Spectrum:

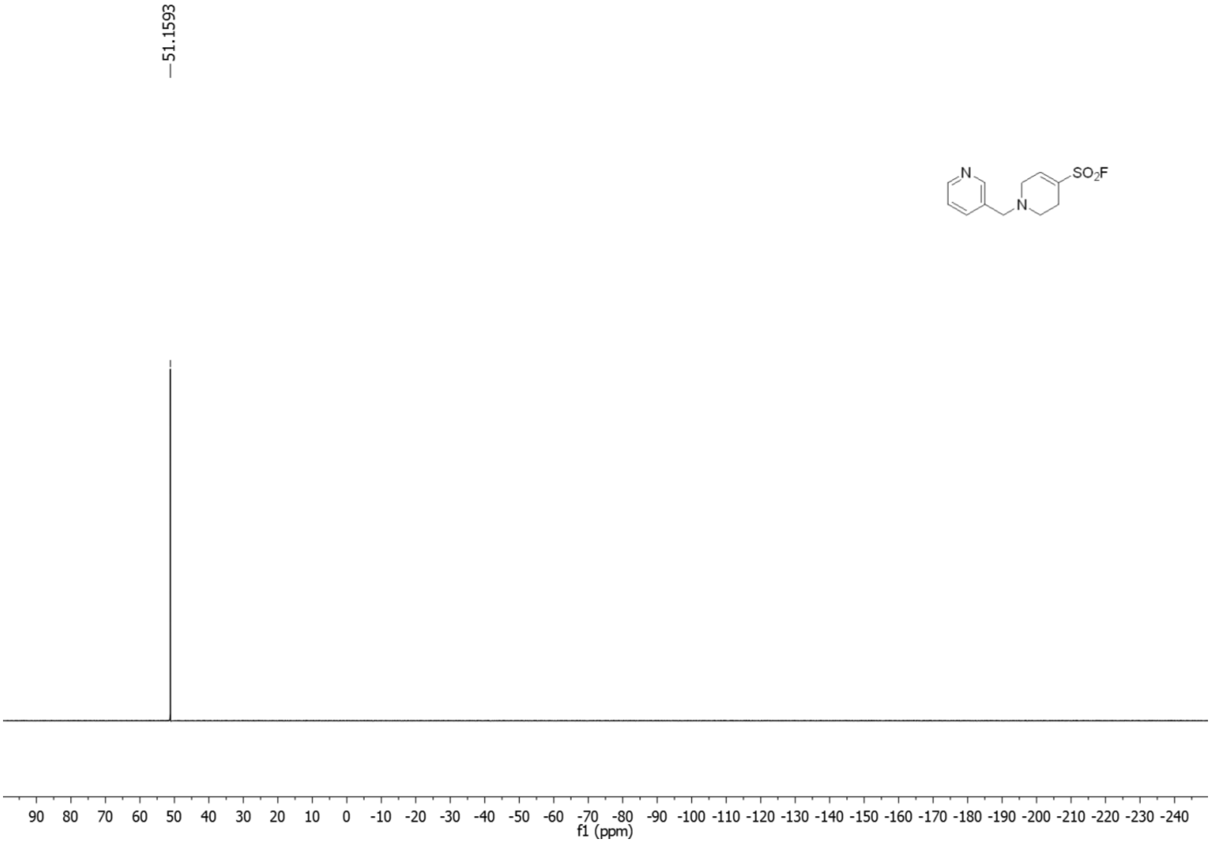

1-(5-((3a*S*,4*S*,6a*R*)-2-Oxohexahydro-1*H*-thieno[3,4-*d*]imidazol-4-yl)pentanoyl)-1,2,3,6-tetrahydropyridine-4-sulfonyl fluoride (16)

^{1}H-NMR Spectrum:

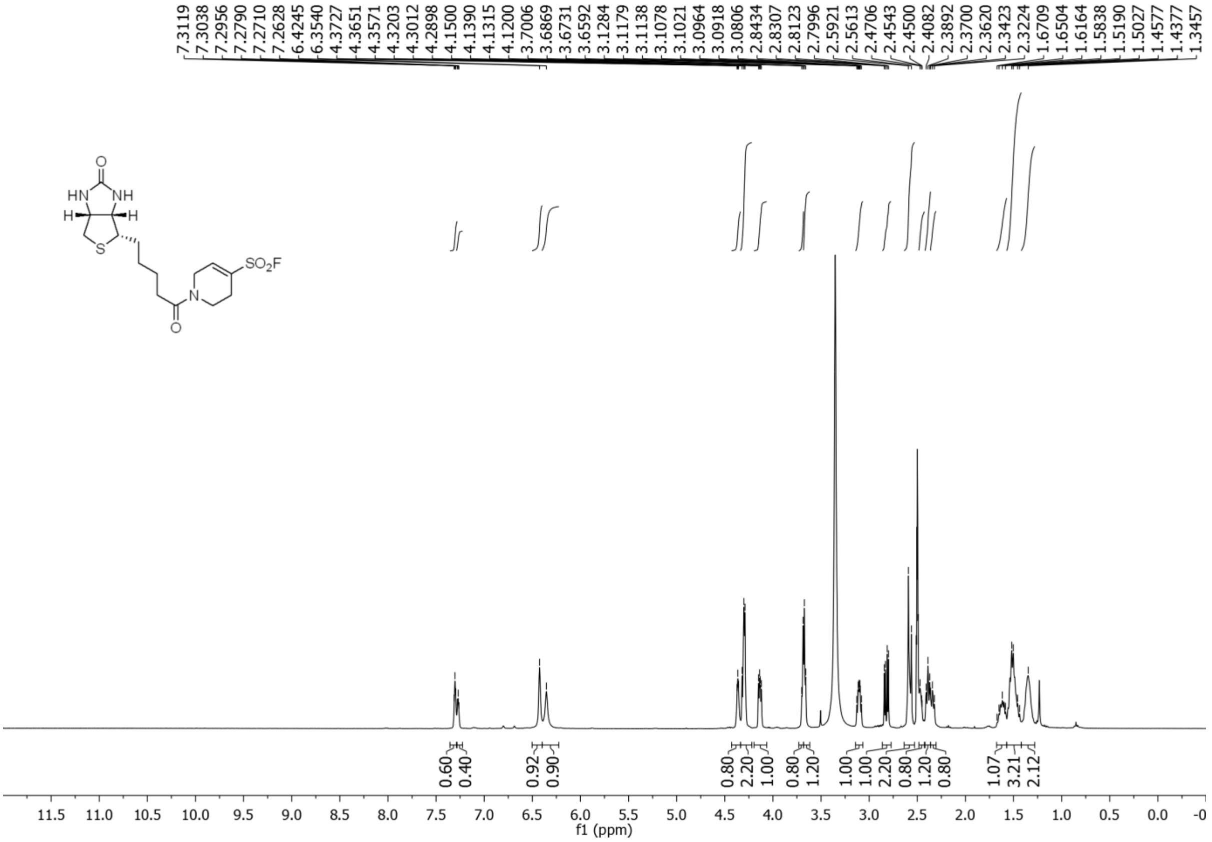

^{13}C-NMR Spectrum:

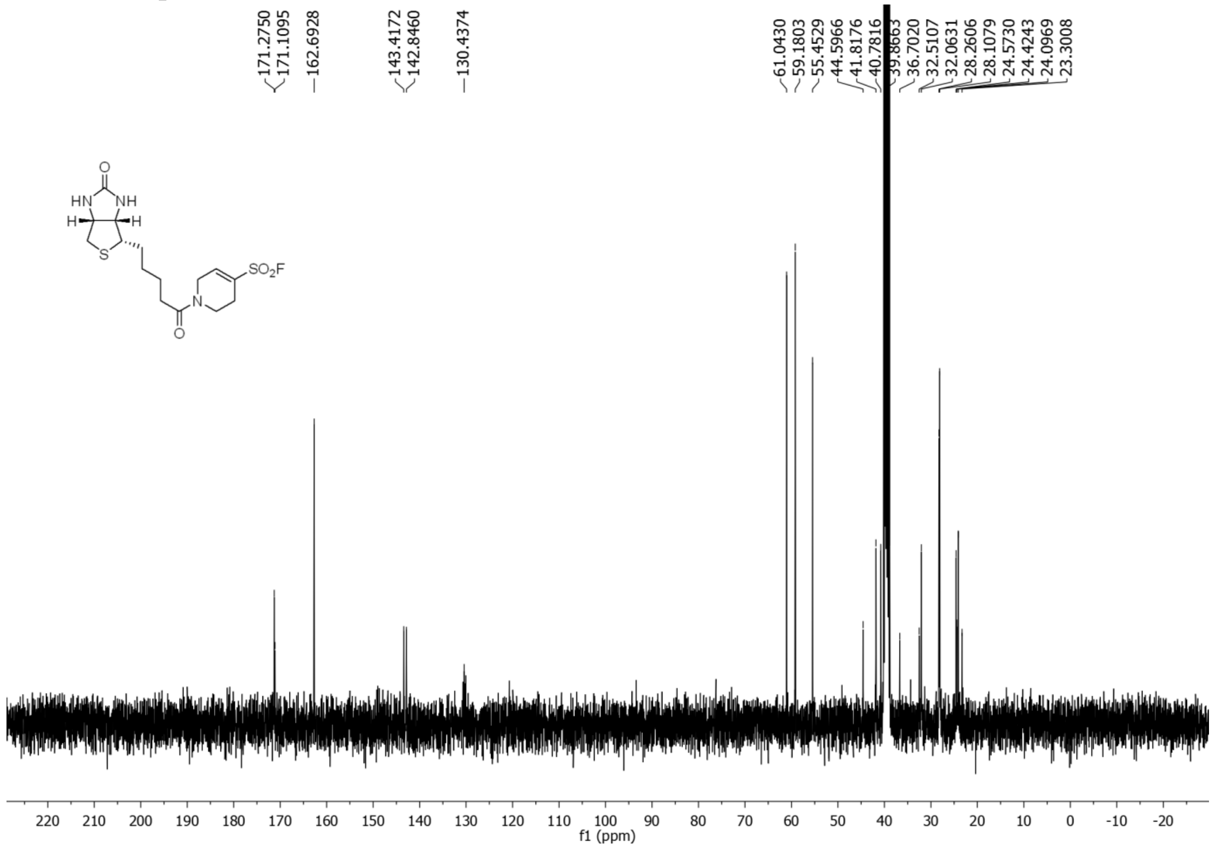

^{19}F-NMR Spectrum:

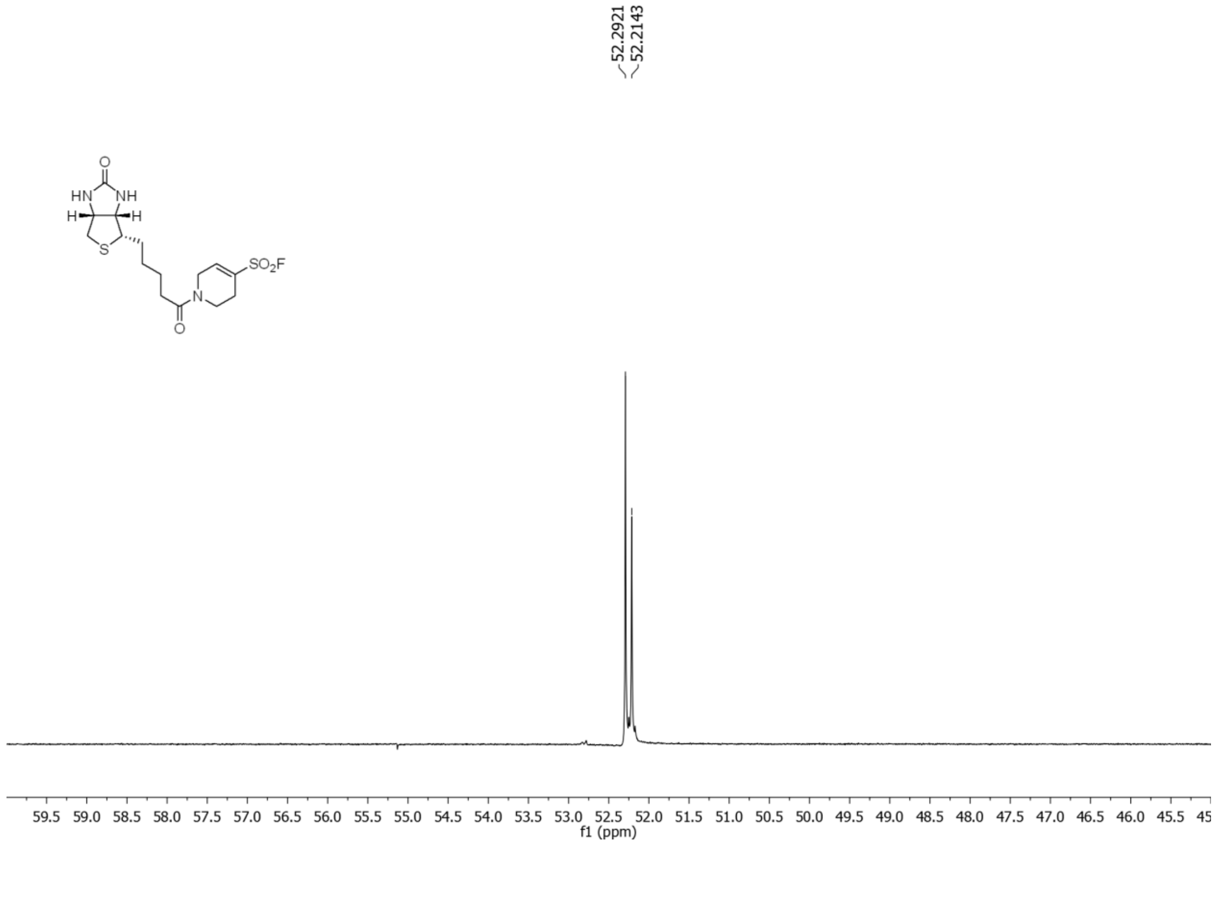

1-(Phenylcarbamoyl)-1,2,3,6-tetrahydro-pyridine-4-sulfonyl fluoride (17)

^{1}H-NMR Spectrum:

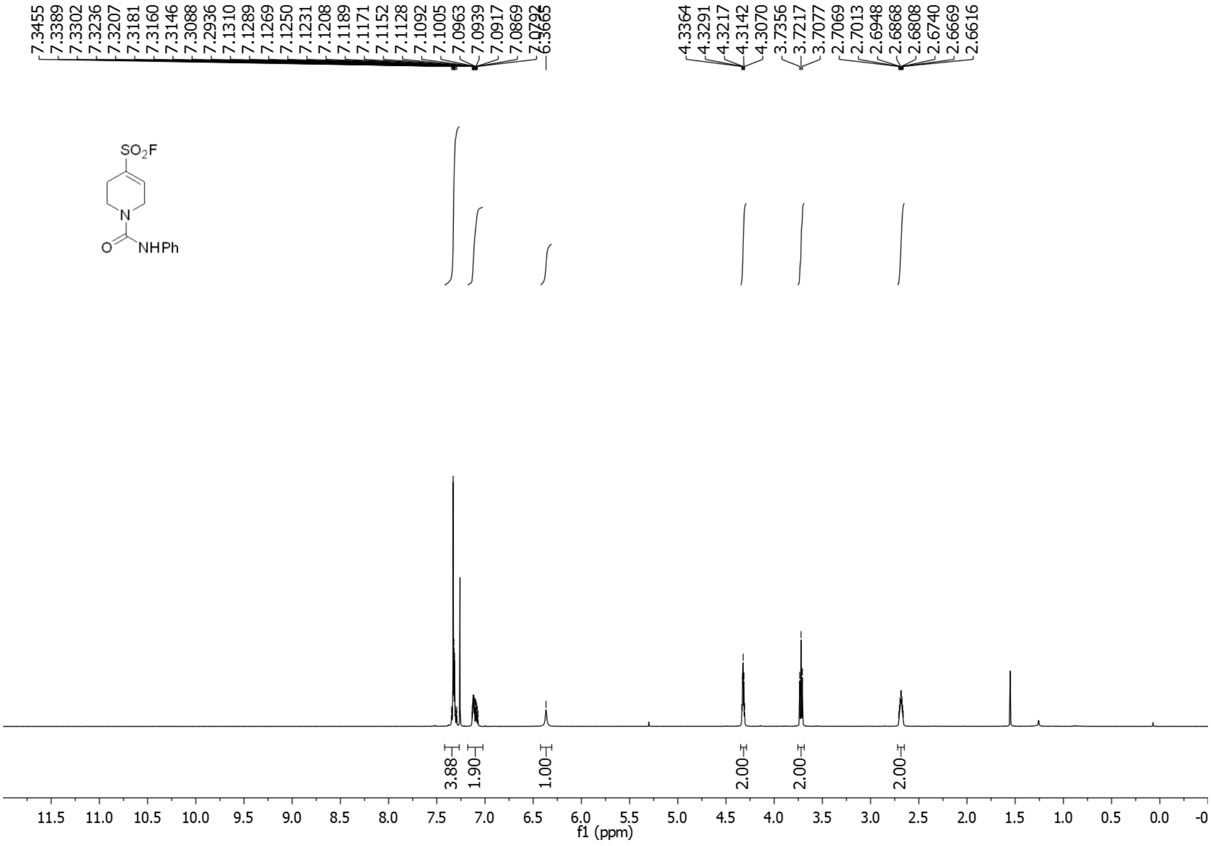

^{13}C-NMR Spectrum:

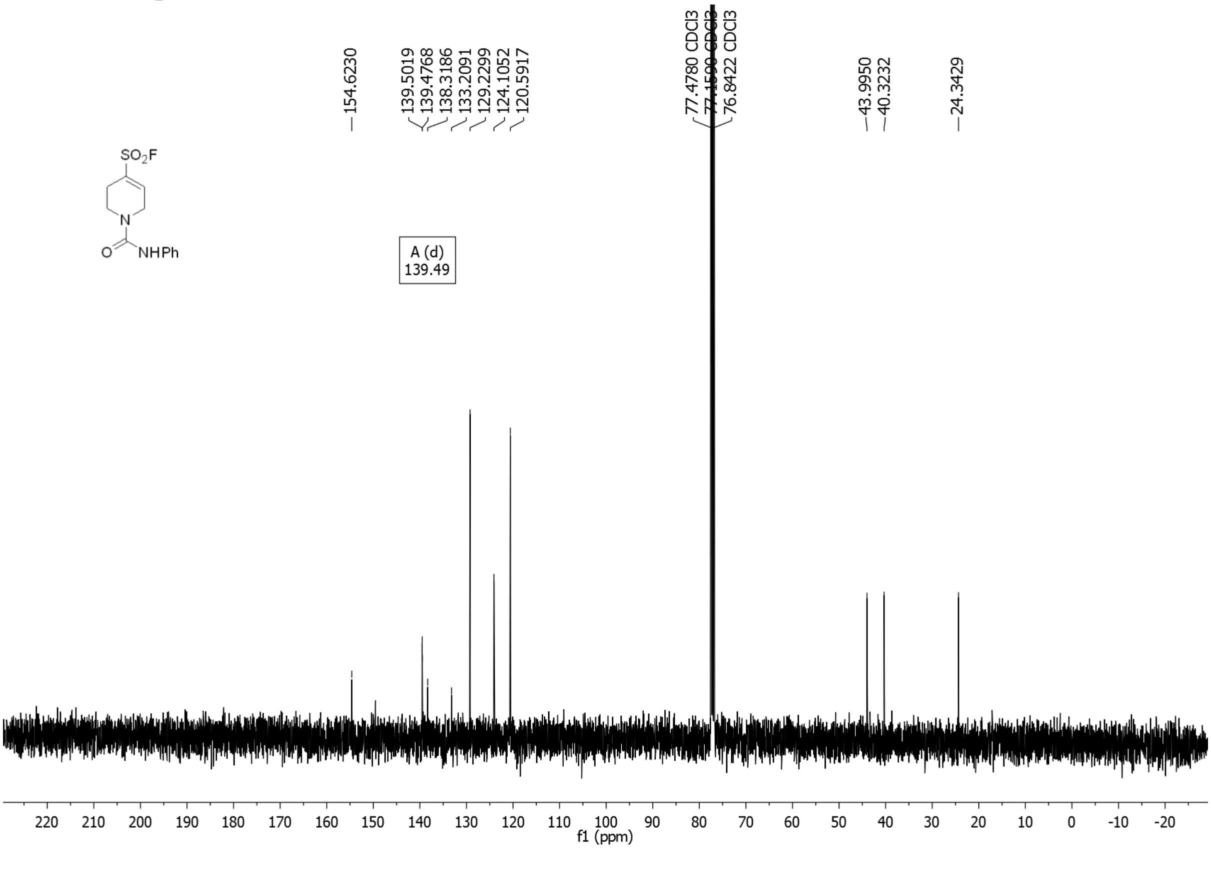

^{19}F-NMR Spectrum:

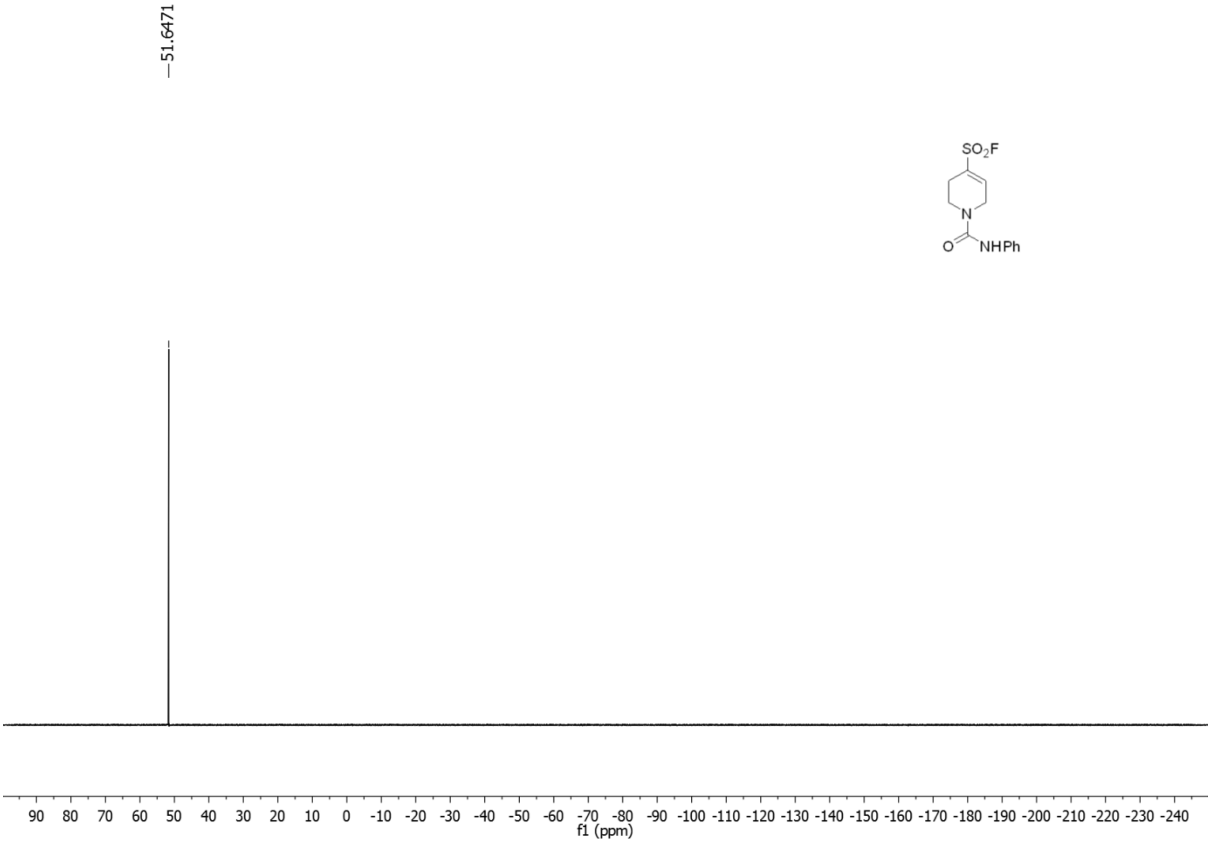

1-(Phenylcarbamothioyl)-1,2,3,6-tetrahydropyridine-4-sulfonyl fluoride (18)

^{1}H-NMR Spectrum:

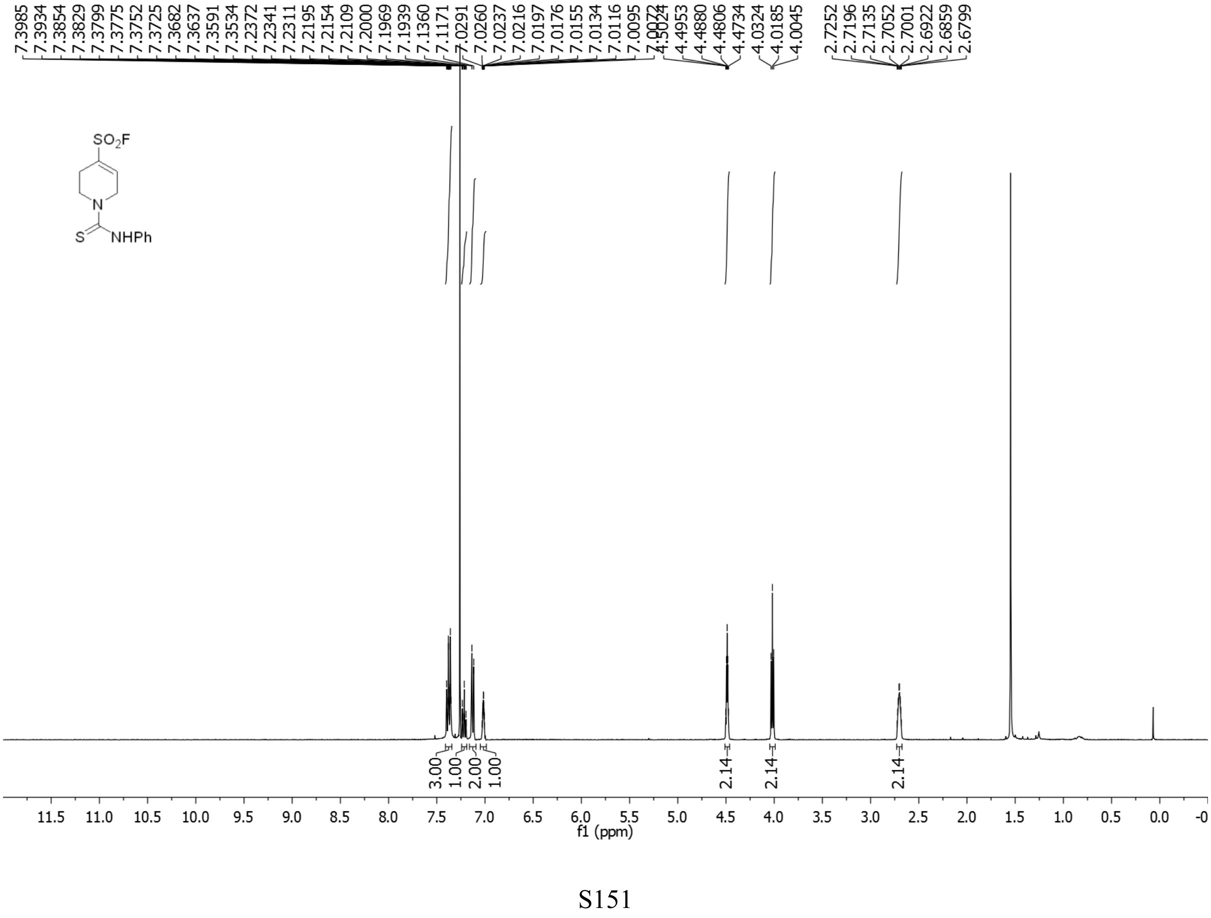

^{13}C-NMR Spectrum:

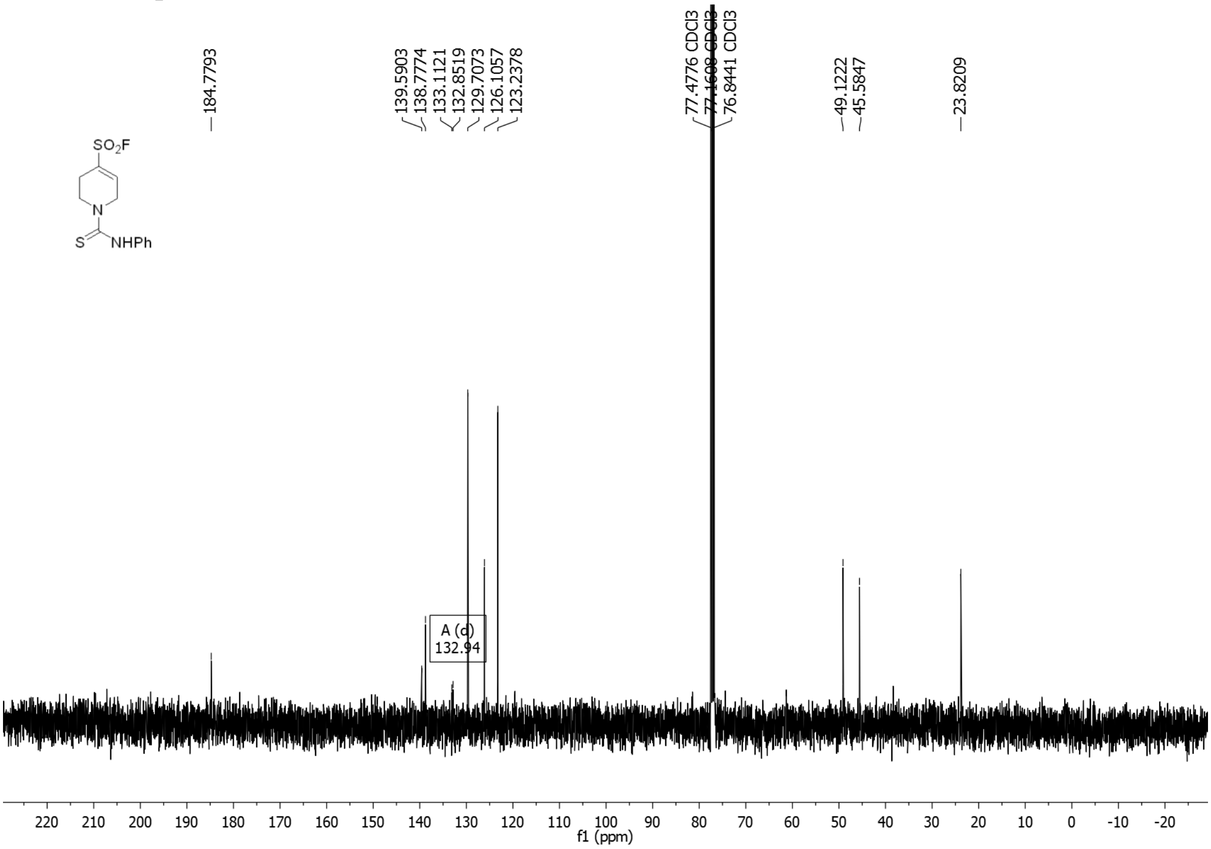

^{19}F-NMR Spectrum:

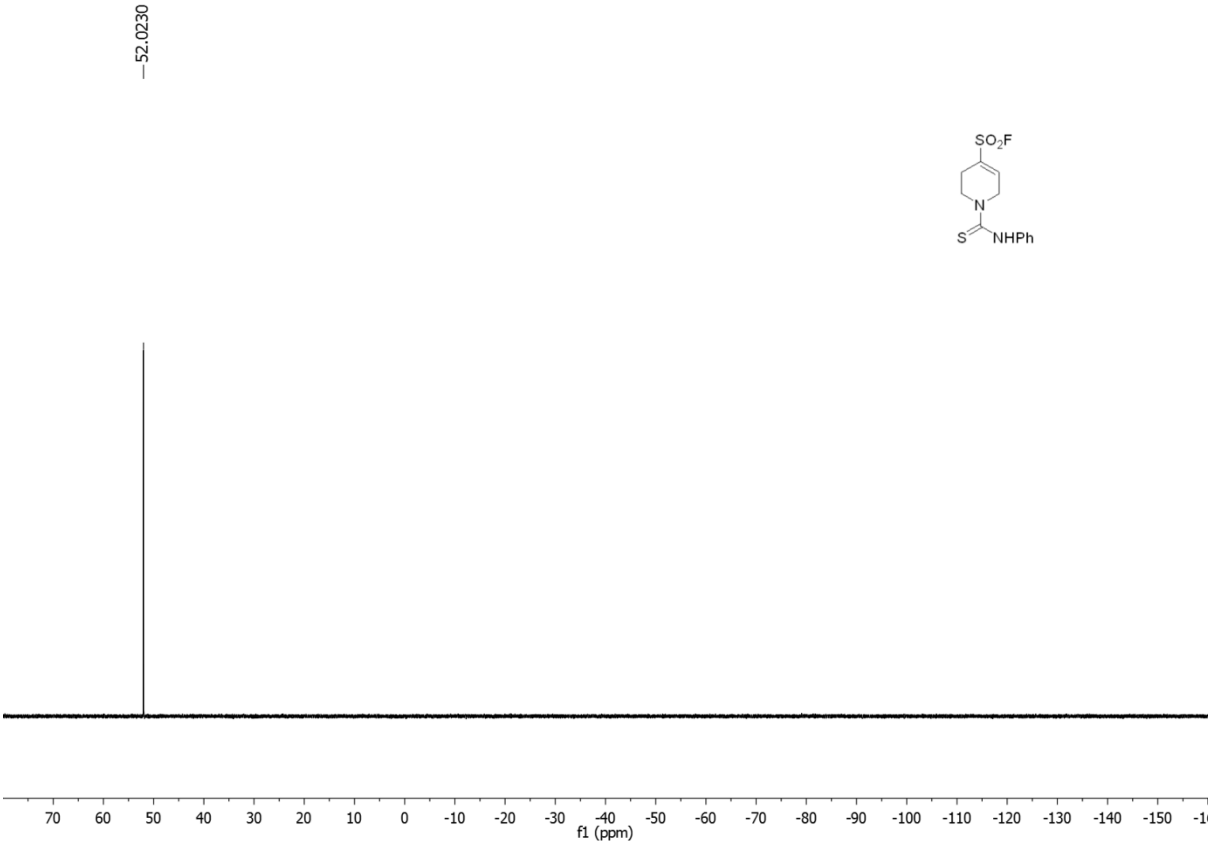

5-(4-(Fluorosulfonyl)-1,2,3,6-tetrahydropyridine-1-carbothioamido)-2-(6-hydroxy-3-oxo-3*H*-xanthen-9-yl)benzoic acid (19)

^{1}H-NMR Spectrum:

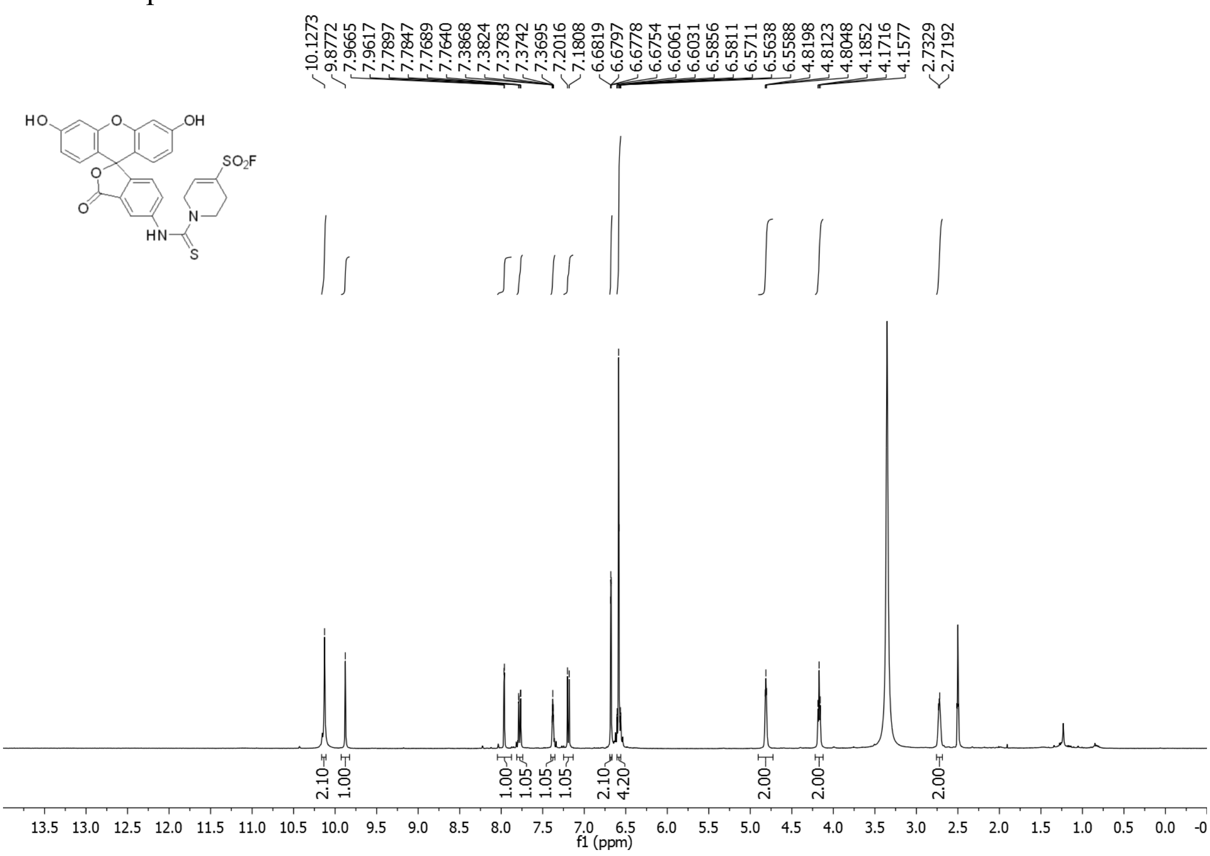

^{13}C-NMR Spectrum:

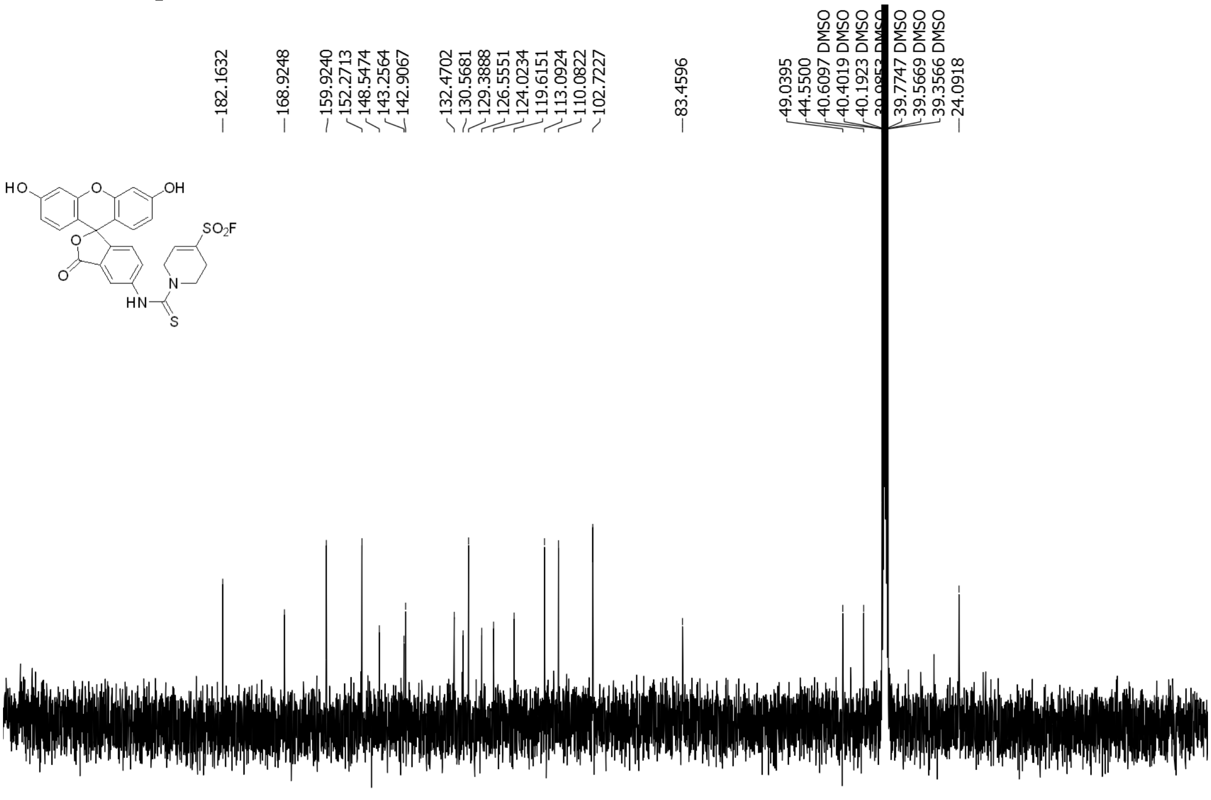

^{19}F-NMR Spectrum:

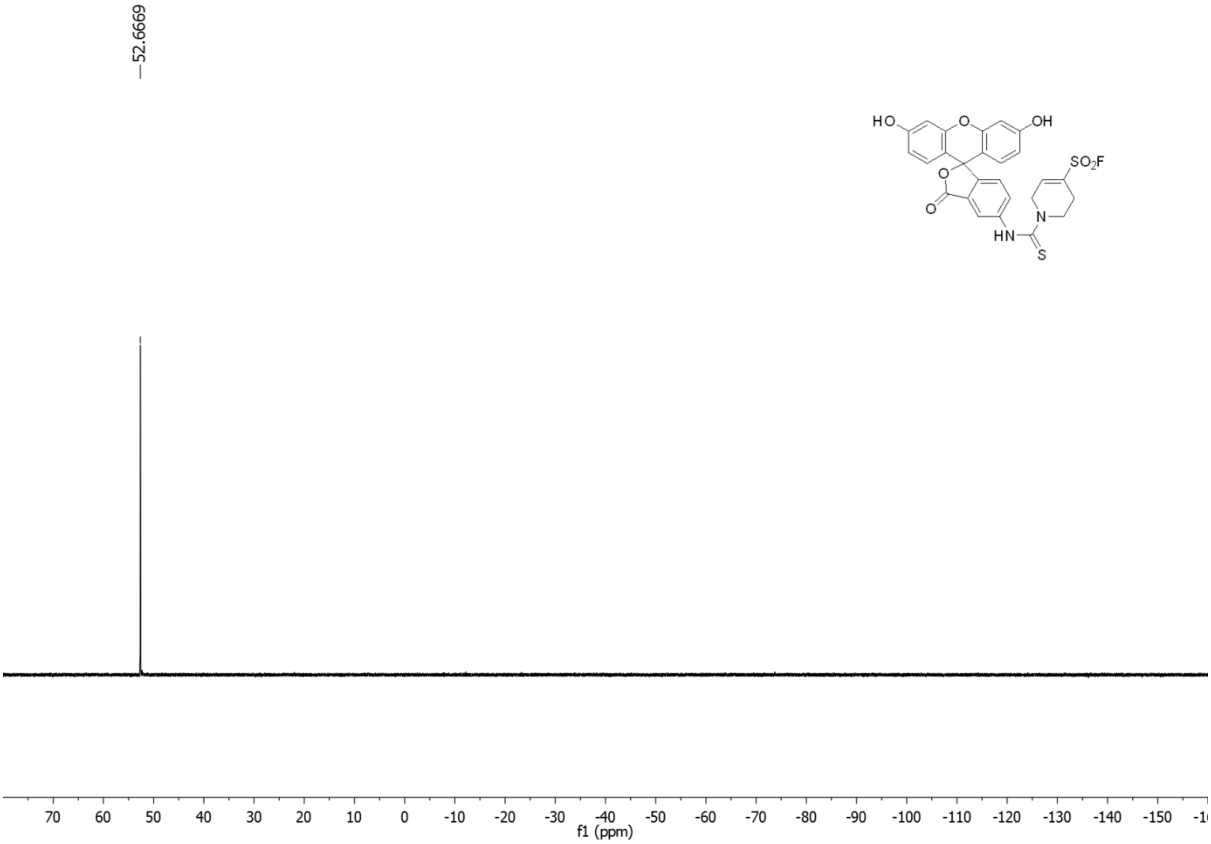

tert-Butyl (*S*)-(1-(4-(fluorosulfonyl)-3,6-dihydropyridin-1(2*H*)-yl)-1-oxo-3-phenylpropan-2-yl)carbamate (20)

^{1}H-NMR Spectrum:

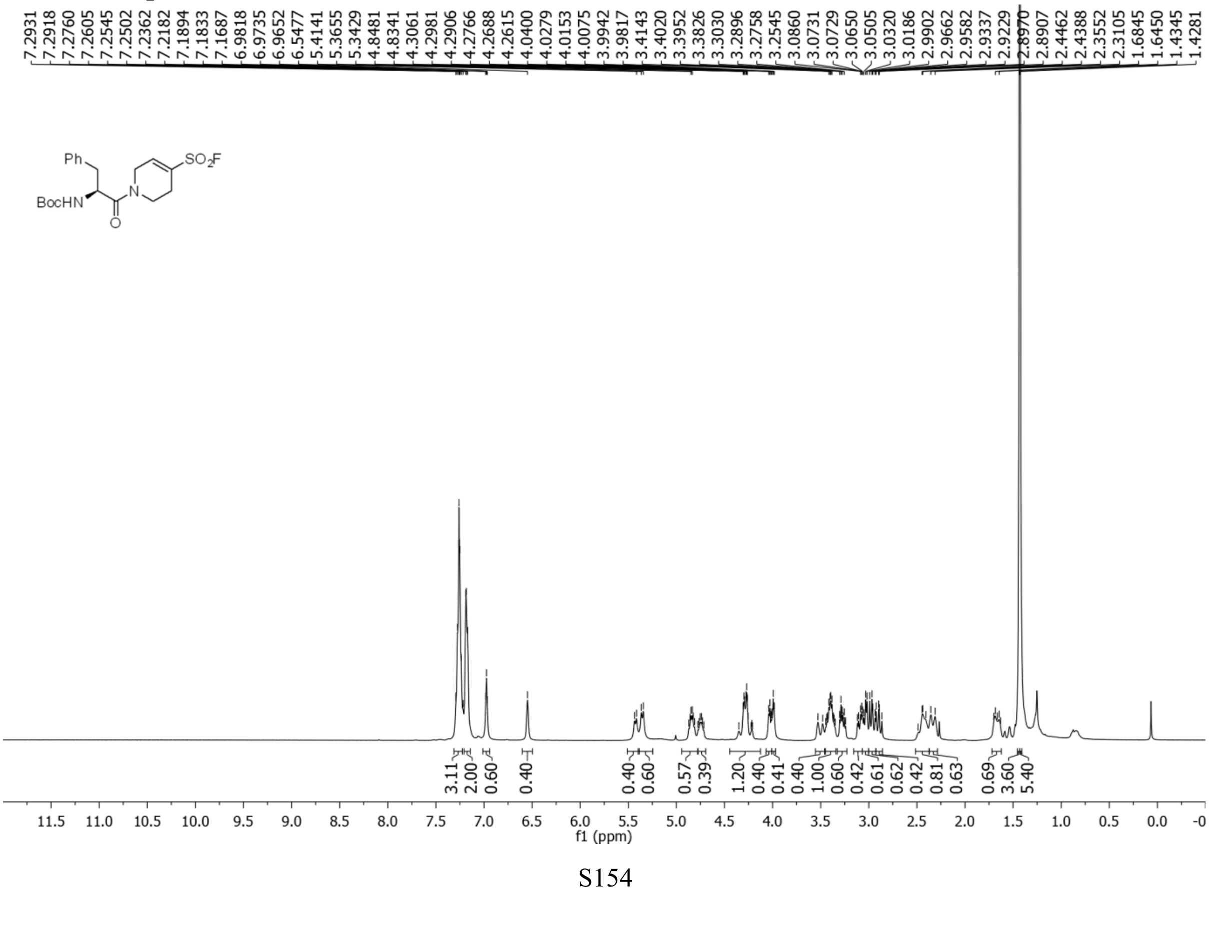

^{13}C-NMR Spectrum:

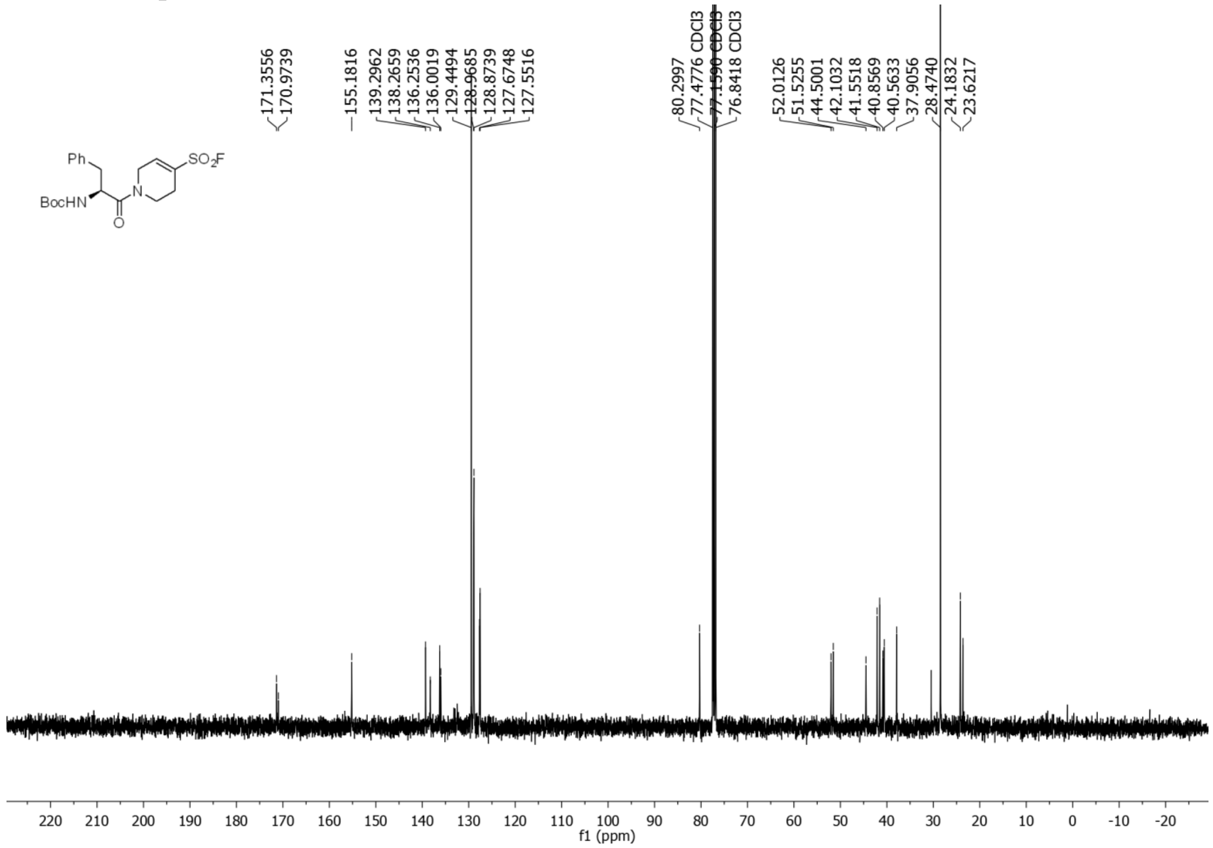

^{19}F-NMR Spectrum:

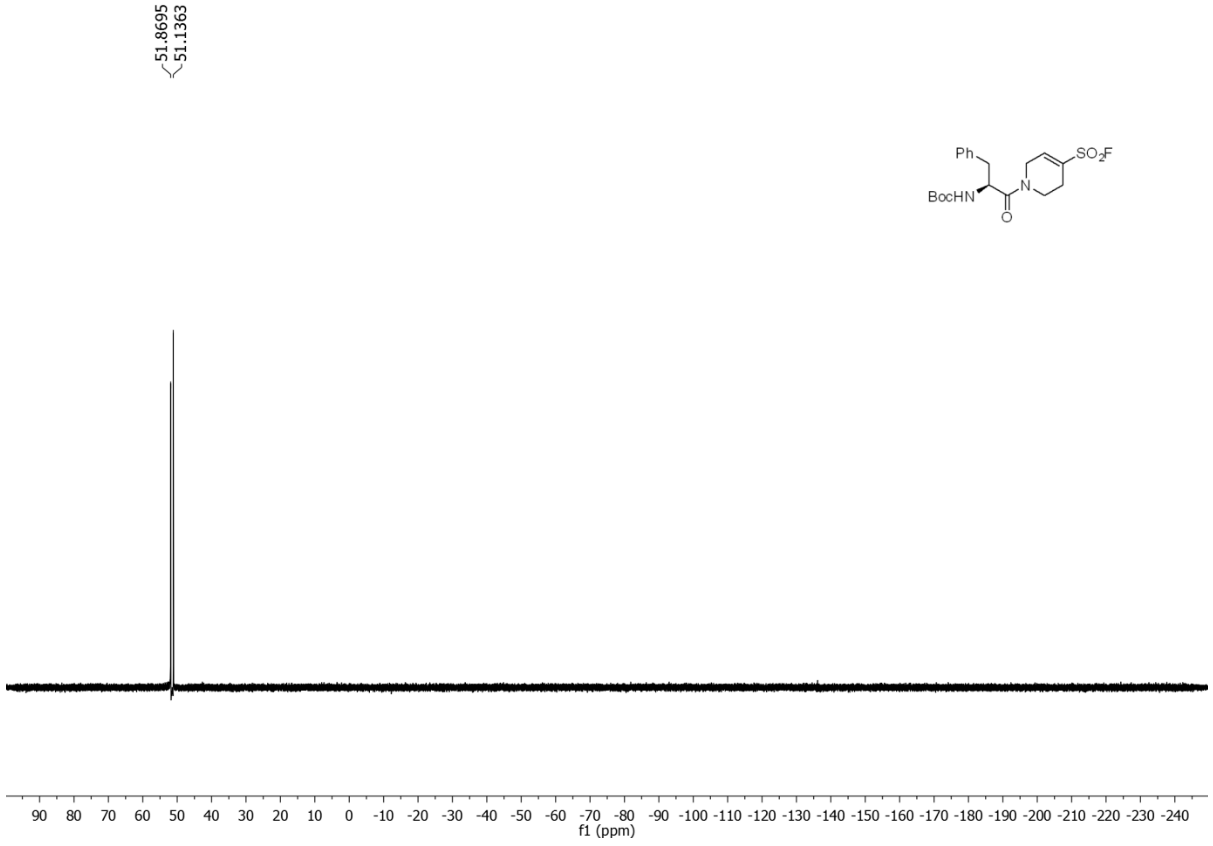

^1H-^1H COSY NMR Spectrum:

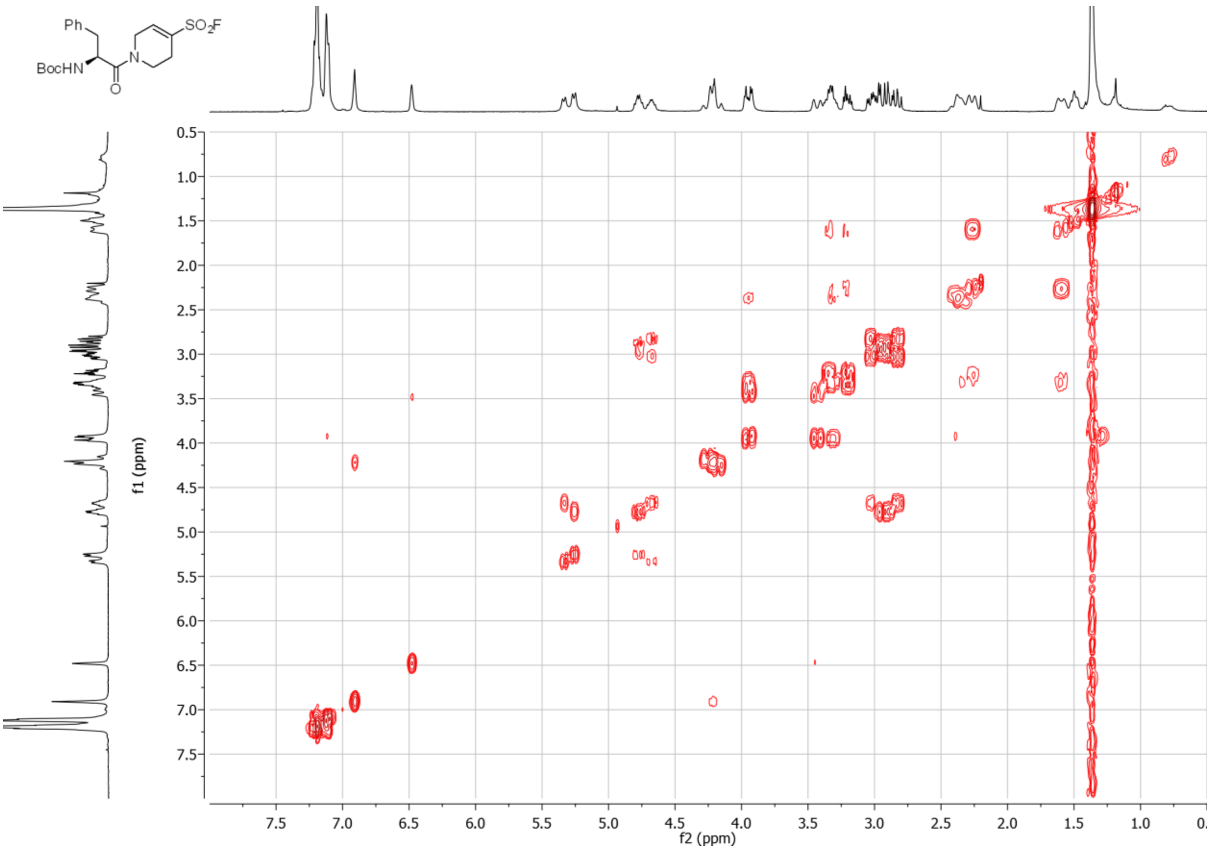

^1H-^{13}C HSQC NMR Spectrum:

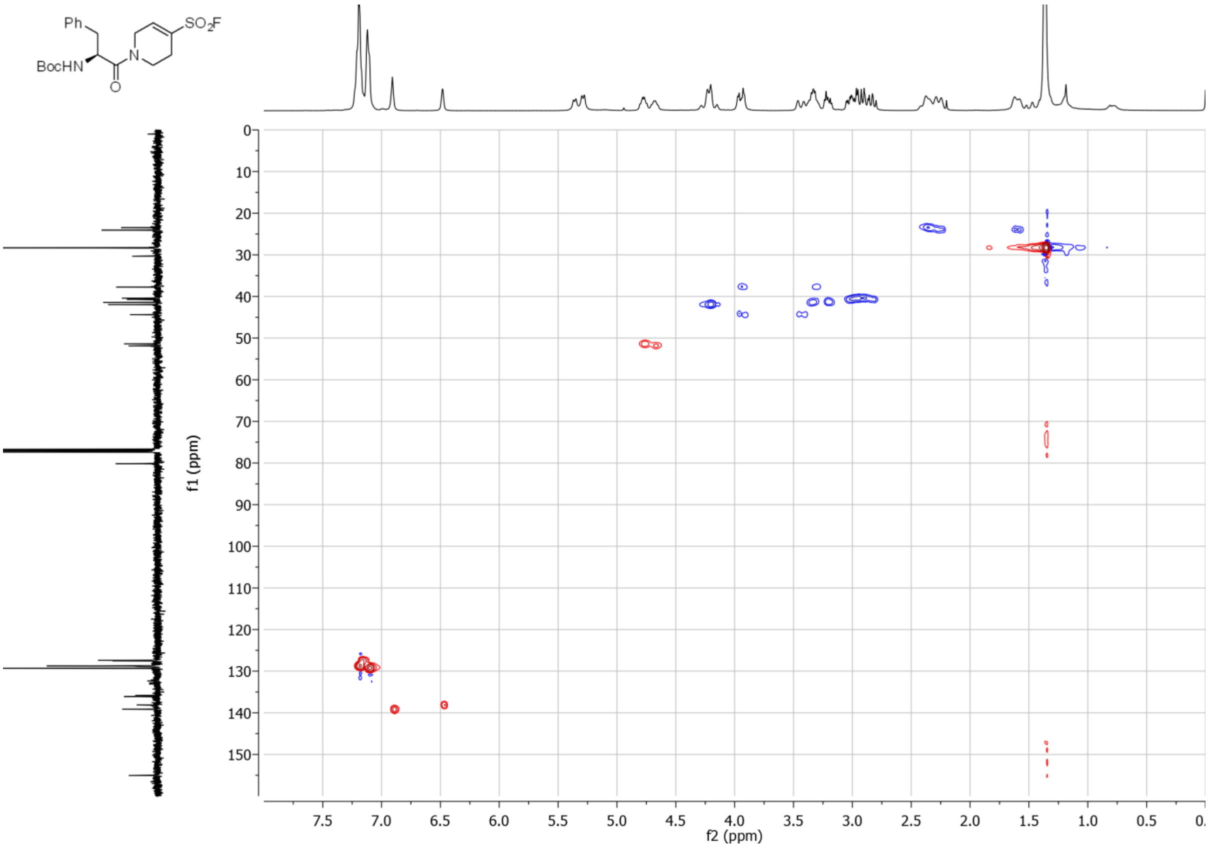

6 Chiral HPLC Traces

HPLC chromatogram of compound **20**:

(Chiralpak IC column, 4.6×250 mm, 90:10 hexane/*i*-PrOH, 1 mL/min, >99:1 er)

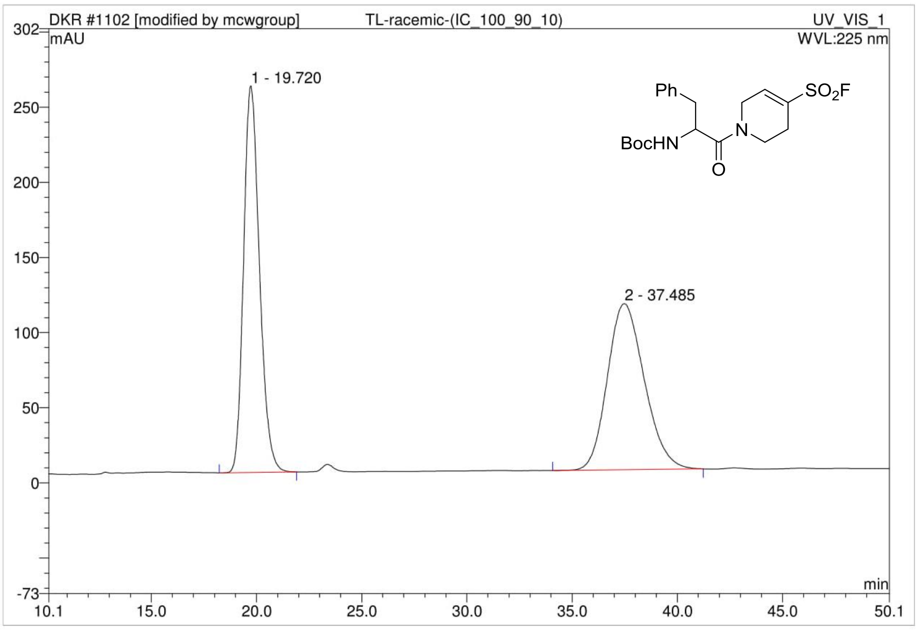

No.	Ret.Time min	Peak Name	Height mAU	Area mAU*min	Rel.Area %	Amount	Type
1	19.72	n.a.	257.229	225.509	50.05	n.a.	BMB*
2	37.49	n.a.	110.571	225.097	49.95	n.a.	BMB*
Total:			367.800	450.606	100.00	0.000	

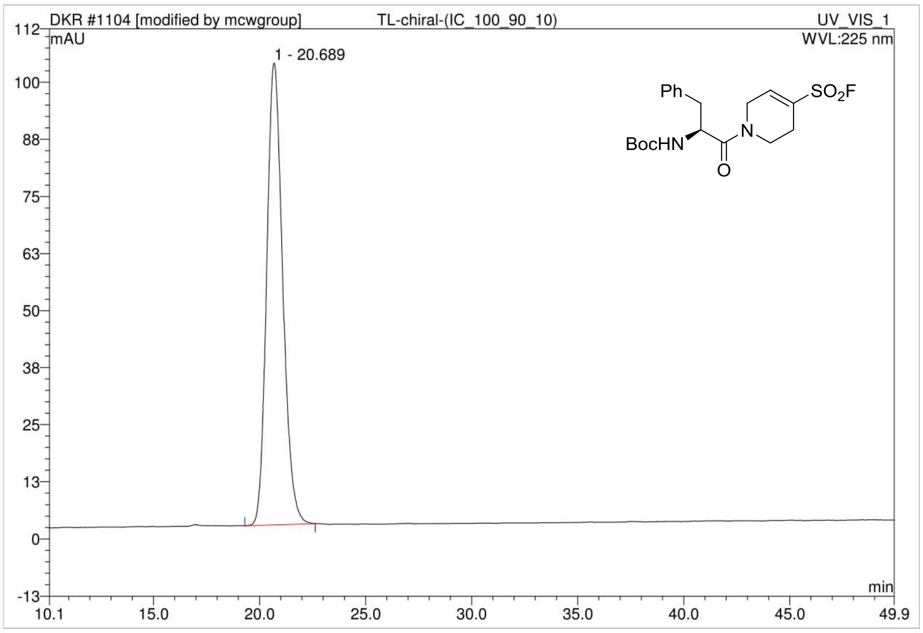

No.	Ret.Time min	Peak Name	Height mAU	Area mAU*min	Rel.Area %	Amount	Type
1	20.69	n.a.	101.155	88.447	100.00	n.a.	BMB*
Total:			101.155	88.447	100.00	0.000	

7 Reference

[1] B. Nguyen, E. J. Emmett, M. C. Willis, *J. Am. Chem. Soc.* **2010**, *132*, 16372-16373.

[2] E. J. Emmett, B. R. Hayter, M. C. Willis, *Angew. Chem. Int. Ed.* **2014**, *53*, 10204-10208.

[3] K. Lee, D. F. Wiemer, *Tetrahedron Lett.* **1993**, *34*, 2433-2436.

[4] A. Jobin-Des Lauriers, C. Y. Legault, *Org. Lett.* **2016**, *18*, 108-111.

[5] D. P. Ojha, K. R. Prabhu, *Org. Lett.* **2015**, *17*, 18-21.

[6] Y. Hioki, K. Okano, A. Mori, *Chem. Commun.* **2017**, *53*, 2614-2617.

[7] E. J. Cho, S. L. Buchwald, *Org. Lett.* **2011**, *13*, 6552-6555.

[8] X. Su, H. Huang, Y. Yuan, Y. Li, *Angew. Chem. Int. Ed.* **2017**, *56*, 1338-1341.

[9] K. Nogi, T. Fujihara, J. Terao, Y. Tsuji, *J. Org. Chem.* **2015**, *80*, 11618-11623.

[10] T. Lampe, R. Kast, M. Jeske, F. Stoll, J. Schuhmacher, E.-M. Becker, H. Beck (Bayer Schering Pharma AG), US2011166163 (A1), **2011**.

[11] N. Kotoku, Y. Sumii, T. Hayashi, S. Tamura, T. Kawachi, S. Shiomura, M. Arai, M. Kobayashi, *ACS Medicinal Chemistry Letters* **2012**, *3*, 673-677.

[12] B. Scheiper, M. Bonnekessel, H. Krause, A. Fürstner, *J. Org. Chem.* **2004**, *69*, 3943-3949.

[13] M. A. Grundl, A. Kaster, E. D. Beaulieu, D. Trauner, *Org. Lett.* **2006**, *8*, 5429-5432.

[14] S. Lessard, F. Peng, D. G. Hall, *J. Am. Chem. Soc.* **2009**, *131*, 9612-9613.

[15] N. Vicart, B. Cazes, J. Gore, *Tetrahedron* **1996**, *52*, 9101-9110.

[16] K. B. Smith, M. K. Brown, *J. Am. Chem. Soc.* **2017**, *139*, 7721-7724.

[17] M. V. Patel, T. Kolasa, K. Mortell, M. A. Matulenko, A. A. Hakeem, J. J. Rohde, S. L. Nelson, M. D. Cowart, M. Nakane, L. N. Miller, M. E. Uchic, M. A. Terranova, O. F. El-Kouhen, D. L. Donnelly-Roberts, M. T. Namovic, P. R. Hollingsworth, R. Chang, B. R. Martino, J. M. Wetter, K. C. Marsh, R. Martin, J. F. Darbyshire, G. Gintant, G. C. Hsieh, R. B. Moreland, J. P. Sullivan, J. D. Brioni, A. O. Stewart, *J. Med. Chem.* **2006**, *49*, 7450-7465.

[18] A. C. Spivey, L. J. Martin, C.-C. Tseng, G. J. Ellames, A. D. Kohler, *Organic & Biomolecular Chemistry* **2008**, *6*, 4093-4095.

[19] S. W. Dantale, B. C. G. Söderberg, *Tetrahedron* **2003**, *59*, 5507-5514.

[20] D. Chang, Y. Gu, Q. Shen, *Chem. Eur. J.* **2015**, *21*, 6074-6078.

[21] E. Richmond, N. Duguet, A. M. Z. Slawin, T. Lébl, A. D. Smith, *Org. Lett.* **2012**, *14*, 2762-2765.

[22] U. Gerlach, T. Wollmann, *Tetrahedron Lett.* **1992**, *33*, 5499-5502.
